# Dysregulation of the HSF1-Mediated UPR^mt^ Pathway in Colonic Smooth Muscle Cells Drives Motility Dysfunction in Functional Constipation

**DOI:** 10.3390/biom16060868

**Published:** 2026-06-12

**Authors:** Junpeng Yao, Wen Wang, Wei Zhang, Hang Dong, Yujun Hou, Qianhua Zheng, Ying Li, Fang Zeng

**Affiliations:** 1 School of Acupuncture and Tuina, Chengdu University of Traditional Chinese Medicine, No. 1166 Liutai Avenue, Wenjiang District, Chengdu 611137, China; yjp@cdutcm.edu.cn (J.Y.); wangwen@stu.cdutcm.edu.cn (W.W.); zhangwei@cdutcm.edu.cn (W.Z.); hangdong@stu.cdutcm.edu.cn (H.D.); houyujun@stu.cdutcm.edu.cn (Y.H.); zhengqianhua@cdutcm.edu.cn (Q.Z.); 2 Key Laboratory of Acupuncture for Senile Disease, Ministry of Education, Chengdu University of Traditional Chinese Medicine, Chengdu 611137, China; 3 Acupuncture Point Effects Key Laboratory of Sichuan Province, Chengdu University of Traditional Chinese Medicine, Chengdu 611137, China

**Keywords:** functional constipation, mitochondrial unfolded protein response, heat shock factor 1, smooth muscle cell, gut motility

## Abstract

Mitochondrial dysfunction in colonic smooth muscle cells (SMCs) is closely associated with impaired gut motility in functional constipation (FC), but the underlying molecular mechanisms remain incompletely understood. The mitochondrial unfolded protein response (UPR^mt^) is a critical pathway for maintaining mitochondrial proteostasis, and heat shock factor 1 (HSF1) acts as an important upstream regulator of this response. In the present study, we employed a loperamide-induced FC mouse model, combined with single-cell transcriptomic, molecular, and functional analyses to characterize the HSF1-UPR^mt^ pathway in colonic SMCs and to investigate its role in FC. Single-cell transcriptomic analysis of colon tissue from FC mice revealed marked downregulation of UPR^mt^-associated genes in colonic SMCs. Immunofluorescence, Western blotting, and RT-qPCR analyses of colonic tissue confirmed that HSF1 expression was reduced in colonic SMCs, along with the downregulation of the UPR^mt^ components, including HSP60, mtHSP70, and LONP1. These molecular changes were accompanied by mitochondrial structural damage, seen by transmission electron microscopy, and by functional impairments, including reduced mitochondrial membrane potential, elevated mtROS production, decreased ATP levels, and diminished activities of respiratory chain complexes I–V. AAV9-mediated overexpression of HSF1 reactivated the UPR^mt^ pathway, improved mitochondrial function, and ameliorated constipation, whereas shRNA-mediated knockdown of HSF1 further suppressed UPR^mt^ activity and aggravated mitochondrial damage, indicating that HSF1 bidirectionally regulates this pathway. Complementary experiments in primary colonic SMCs confirmed that this regulatory mechanism operates in a cell-autonomous manner, as modulation of HSF1 expression produced corresponding changes in the UPR^mt^ pathway, in the expression of mitochondrial respiratory chain complex subunits (ATP5A, NDUFA9, COX1, SDHA, UQCRC1), and in ATP production, mirroring the in vivo findings. Collectively, these results demonstrate that HSF1 plays a pivotal role in maintaining mitochondrial homeostasis in colonic SMCs through regulation of the UPR^mt^ pathway and that HSF1 dysfunction is closely associated with slowed gut motility in FC. These findings offer a new mechanistic perspective on FC and point to the HSF1–UPR^mt^ axis as a potential therapeutic target.

## 1. Introduction

Functional constipation (FC) is a functional colonic disorder distinct from irritable bowel syndrome, clinically characterized by persistent difficulty in defecation, reduced bowel movement frequency, and a sensation of incomplete evacuation [1]. Epidemiological studies estimate the global adult prevalence of FC at approximately 16% [2], and its symptoms substantially impair patients’ quality of life while imposing a heavy healthcare burden [3]. The primary pathophysiological substrate of FC is a colonic motility disorder that manifests as weakened propulsive contractions and delayed colonic transit [4,5,6]. First-line treatments predominantly rely on laxatives, but prolonged use often produces adverse effects such as drug dependence and electrolyte disturbances [7,8]. Consequently, deeper investigation of the molecular mechanisms underlying colonic motility dysfunction in FC remains essential for the development of novel targeted therapies.

Rhythmic contractions of colonic smooth muscle cells (SMCs) directly drive the propulsion of intestinal contents and therefore require a continuous mitochondrial supply of adenosine triphosphate (ATP) [9]. Previous studies have demonstrated that mitochondrial dysfunction—manifested by altered mitochondrial dynamics and impaired mitochondrial biogenesis—is closely associated with a range of gastrointestinal motility disorders [10,11,12]. In colonic SMCs derived from patients with FC and from relevant animal models, investigators have reported reduced ATP production [13,14], increased accumulation of reactive oxygen species (ROS) [15,16], and fragmentation of the mitochondrial network [17,18]. These alterations collectively implicate mitochondrial dysfunction as a major contributor to the impaired colonic motility observed in FC. However, the upstream regulatory mechanisms responsible for these mitochondrial abnormalities, particularly the core transcriptional pathways, remain incompletely understood.

The mitochondrial unfolded protein response (UPR^mt^) represented a central adaptive pathway that cells activated to restore mitochondrial proteostasis [19,20]. In mammals, the UPR^mt^ operated through multiple regulatory branches. The canonical branch involved activating transcription factor 5 (ATF5)—together with ATF4/CHOP in certain contexts—which bound to mitochondrial unfolded protein response elements (MUREs) in target gene promoters and coordinated a broad transcriptional program of chaperones, proteases, and metabolic enzymes [21,22,23]. A separate branch functioned through heat shock factor 1 (HSF1) [24]. Although HSF1 had been traditionally recognized as the main regulator of the cytosolic heat shock response, driving the expression of HSP70, HSP90, and other cytosolic chaperones during proteotoxic stress, subsequent studies revealed its direct contribution to the UPR^mt^ through a distinct mechanism. Sutandy et al. [25] demonstrated that impaired mitochondrial protein import led to the accumulation of unimported precursor proteins in the cytosol, which were subsequently detected by HSF1. This detection prompted HSF1 to translocate to the nucleus, where it activated a specific subset of mitochondrial proteostasis genes, including HSP60, mtHSP70, and LONP1, via heat shock elements (HSEs) in their promoters [25,26]. Importantly, HSF1’s involvement in the UPR^mt^ arose from mitochondrial import stress rather than heat, and its transcriptional targets favored mitochondrial chaperones over cytosolic ones [27]. In addition to its established role in the heat shock response, HSF1-mediated regulation of the UPR^mt^ facilitated cellular adaptation to metabolic challenges, such as oxidative stress. Although the HSF1–UPR^mt^ axis has been extensively implicated in diverse pathological contexts, including metabolic disorders [28], neurodegenerative diseases [29], and cardiac aging [30], its specific role and regulatory mechanisms in colonic SMCs during FC remain unexplored. We therefore hypothesized that dysfunction of the HSF1–UPR^mt^ signaling axis contributes to mitochondrial damage in colonic SMCs and thereby promotes the development of colonic motility disorders in FC.

**Figure 1:**
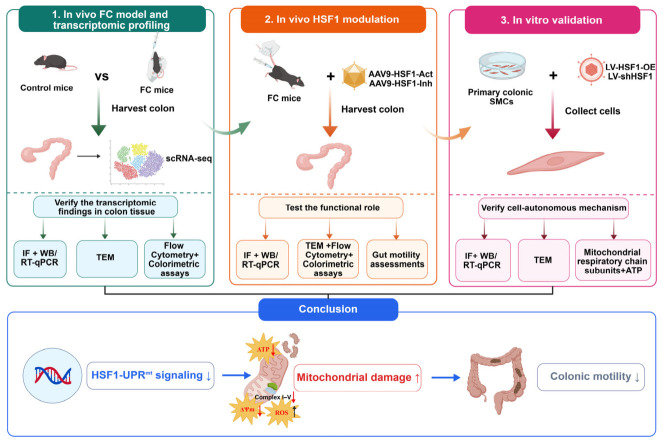
Study design and experimental workflow. (1) In vivo FC model and transcriptomic profiling: colonic SMCs from loperamide-induced FC and control mice were analyzed by single-cell transcriptomic sequencing, and findings were verified in colonic tissue. (2) In vivo HSF1 modulation: AAV9-mediated HSF1 overexpression or shRNA knockdown was performed in FC mice, followed by analysis of the UPR^mt^ pathway, mitochondrial structure and function, and gut motility. (3) In vitro validation: primary colonic SMCs were transduced with lentivirus for HSF1 overexpression or knockdown, and mitochondrial structure and function were assessed. Arrows indicate the direction of effects on mitochondrial integrity and colon motility. Abbreviations in the figure are explained in the main text (This figure was created with BioGDP.com).

To test this hypothesis, we adopted an integrated experimental strategy (Figure 1). We first established a FC mouse model by administering loperamide hydrochloride, a peripherally acting μ-opioid receptor agonist that reliably induced FC. Our group and other laboratories had extensively validated this model for investigating the molecular mechanisms underlying FC [31,32,33,34]. Using this model, we performed single-cell sequencing of colon tissue and identified transcriptomic changes in colonic SMCs associated with FC. These findings were then validated in colon tissue by immunofluorescence, Western blotting, and RT-qPCR. Mitochondrial structure was examined by transmission electron microscopy, and mitochondrial function was evaluated by flow cytometry, ATP measurement, and respiratory chain complex activity assays. After establishing the molecular and functional correlates of UPR^mt^ suppression, we asked whether HSF1 directly regulates this pathway. To address this, we bidirectionally modulated HSF1 in vivo via adeno-associated virus serotype 9 (AAV9)-mediated overexpression and shRNA knockdown in FC mice, and examined the effects on the UPR^mt^ pathway, mitochondrial homeostasis, and gut motility. In parallel, we performed experiments in primary colonic SMCs using lentivirus-mediated HSF1 overexpression and knockdown to determine whether this regulatory mechanism operates in a cell-autonomous manner. Collectively, this study aimed to elucidate previously unrecognized mechanisms underlying FC pathogenesis and to provide a theoretical framework for the development of therapeutic strategies targeting mitochondrial homeostasis.

## 2. Materials and Methods

### 2.1. Animals Care

Ninety SPF-grade male C57BL/6J mice, aged 6–8 weeks and weighing 20 ± 2 g, were purchased from Chengdu Dashuo Laboratory Animal Co., Ltd. (Chengdu, china) (license number: SCXK (Sichuan) 2025-0030). They were housed under controlled conditions at 20 ± 2 °C, 50% ± 5% relative humidity, and a 12 h/12 h light–dark cycle, with ad libitum access to standard chow diet and sterile drinking water throughout the experimental period. After seven days of acclimation, the mice were randomly assigned using a random number table into six groups (*n* = 15 per group). Group allocations and treatments are described in Section 2.3. All procedures complied with ethical guidelines and were approved by the Animal Experiment Ethics Committee of Chengdu University of Traditional Chinese Medicine (Approval No. 2025119).

### 2.2. Construction of Adeno-Associated Virus Serotype 9 (AAV9) and Lentiviral Vectors

The construction, packaging, and purification of AAV9 and lentiviral vectors were completed by Shandong Vigenebio Biotechnology Co., Ltd. (Jinan, China).

*AAV9 vectors:* All AAV9 constructs were based on the pAV backbone. To enable bidirectional modulation of HSF1 expression and to verify its transfection in colonic SMCs, we used two engineered vectors, AAV9-HSF1-act and AAV9-HSF1-inh, each co-expressing a GFP reporter with the target sequence (Table S1). The AAV9-HSF1-act vector carried the mouse HSF1 coding sequence (NM_008296.3). All viruses utilized the smooth muscle-specific Sm22a (Tagln) promoter, which facilitated selective transgene expression in vascular and visceral SMCs. The corresponding controls included AAV9-act-Veh, an empty vector control for the overexpression construct that expressed GFP alone, and AAV9-inh-Veh, a scrambled control for the shRNA construct that expressed a non-targeting shRNA along with GFP. Both controls were also driven by the Sm22a promoter.

*Lentiviral vectors:* The HSF1 overexpression lentivirus (LV-HSF1-OE) utilized the pLent-EF1a-3Flag-CMV-copGFP-P2A-Puro vector, likewise carrying the mouse HSF1 coding sequence (NM_008296.3). The HSF1 knockdown lentivirus (LV-shHSF1) was constructed with the pLent-U6-shRNA-CMV-RFP-P2A-Blasticidin vector, incorporating a specific shRNA that targeted mouse HSF1. A scrambled non-targeting shRNA served as the control (LV-shVeh). Empty vector controls (LV-Veh) included the same backbone without the HSF1 insert. Lentiviral transduction procedures are described in Section 2.11.

### 2.3. Animal Grouping, AAV Transduction, and FC Model Establishment

*Animal grouping:* After acclimation, mice were randomly assigned to six groups (n = 15 per group): (1) Control group (no AAV9 vector and no loperamide, only saline gavage); (2) FC group (no AAV9 vector, received only loperamide); (3) HSF1-Act group (received AAV9 encoding HSF1, followed by FC model induction); (4) Act-Veh group (received the corresponding empty AAV9 vector, followed by FC model induction); (5) HSF1-Inh group (received shAAV9 targeting HSF1, followed by FC model induction); and (6) Inh-Veh group (received the scrambled control AAV9 vector, followed by FC model induction).

*AAV transduction:* Two weeks before establishing the FC model, the AAV transduction was performed via rectal enema [31]. After a 24 h fast and under isoflurane anesthesia, an enema needle was inserted approximately 2.5 cm into the colon. We first perfused 300 μL of 20 mM N-acetylcysteine solution and retained it for 30 min to remove mucus. Subsequently, 200 μL of AAV solution (5 × 10^10^ vg/mouse) was injected. After perfusion, the anus was briefly occluded, and the mice were held vertically for 1 min to promote uniform viral distribution across the colonic mucosa. To verify transduction efficiency and specificity, we set aside 4 mice per group before functional experiments for fluorescence detection of GFP and for measurement of HSF1 mRNA and protein levels.

*FC model establishment:* The FC model was then established using the loperamide gavage protocol previously published by our group [31,32]. Loperamide hydrochloride (Sigma-Aldrich, 34552-83-5) was suspended in 0.9% normal saline at a concentration of 1 mg/mL. Mice in the FC, HSF1-Act, Act-Veh, HSF1-Inh, and Inh-Veh groups received oral gavage of loperamide at a dosage of 10 mg/kg twice daily for 14 consecutive days. In contrast, mice in the Control group received an equal volume of 0.9% normal saline following the same schedule.

### 2.4. Assessment of Gut Motility-Related Parameters

Following the above-described treatments, mice were fasted for 12 h and then gavaged with Evans blue solution (Solarbio, Beijing, China, E8010). The interval from gavage to the first appearance of blue feces was recorded for each mouse as the total gastrointestinal transit time (TGITT). The model was deemed successful when the TGITT in the FC group was significantly prolonged relative to that of the controls. Gut motility function was then evaluated by measuring TGITT, 6 h fecal pellet output, fecal water content, and small intestinal propulsive rate. TGITT was recorded as the time (min) from Evans blue gavage to the first appearance of a blue fecal pellet [31,33]. For 6 h fecal output, pellets excreted by each mouse were collected and counted over 6 h. Fecal water content was calculated as (wet weight − dry weight)/wet weight × 100% after drying feces at 60 °C. Small intestinal propulsive rate was determined 30 min after Evans blue gavage [34]: mice were euthanized, the small intestine was removed, and the propulsion rate was calculated as (length of blue-stained segment/total intestinal length) × 100%.

### 2.5. Sample Collection and Processing

Upon completion of gut motility assessments (TGITT, 6 h fecal pellet output, fecal water content, and small intestinal propulsive rate), mice were euthanized by cervical dislocation under isoflurane anesthesia. The small intestine was removed for measurement of the propulsive rate as described above. Colonic tissues were promptly collected, rinsed with pre-cooled phosphate-buffered saline (1× PBS; 137 mM NaCl, 2.67 mM KCl, 10 mM Na_2_HPO_4_, 2.0 mM KH_2_PO_4_, without Ca^2+^/Mg^2+^, pH 7.4). Among the 15 mice per group, 3 were used for single-cell RNA sequencing, 4 were used for verification of AAV9 transduction efficiency, the remaining 8 mice were allocated to downstream morphological and molecular assays. Colons from these 8 mice were each divided into four segments: one segment was immediately fixed in glutaraldehyde for transmission electron microscopy; one segment was immediately fixed in paraformaldehyde for H&E staining and immunofluorescence; one segment was prepared fresh as a single-cell suspension for flow cytometry; one segment was snap-frozen in liquid nitrogen and stored at −80 °C for subsequent molecular and biochemical analyses. The specific homogenization and extraction procedures for each assay are described in the corresponding sections below.

### 2.6. Single-Cell RNA Sequencing Analysis

Single-cell RNA sequencing was performed using the DNBelab C-TaiM4 platform [35]. Fresh colon tissues from the 3 mice per group designated for single-cell RNA sequencing (see Section 2.5) were collected and rinsed with pre-cooled 1× PBS. Tissues were minced and digested with 0.25% Trypsin (Thermo Fisher Scientific, Waltham, MA, USA) and 10 μg/mL DNase I (Sigma-Aldrich, St. Louis, MO, USA) in 1× PBS at 37 °C for 40 min with agitation at 50 rpm. Every 20 min, the supernatant containing released cells was collected, immediately mixed with an equal volume of RPMI-1640 medium containing 30% fetal bovine serum (FBS) to terminate digestion, and the remaining tissue was replenished with fresh digestion solution. The terminated cell suspensions from each collection were pooled. The pooled cell suspension was then filtered through a 40 μm nylon cell strainer. The filtrate was centrifuged at 300× *g* for 5 min at 4 °C. The cell pellet was resuspended in red blood cell lysis buffer (Thermo Fisher Scientific, Waltham, MA, USA, 00-4333-57) at a volume ratio of 3:1 (lysis buffer: cell suspension) and incubated for 2 min at room temperature. The lysis reaction was quenched by adding an excess volume of 1× PBS, and the cells were centrifuged at 300× *g* for 5 min at 4 °C. The resulting cell pellet was resuspended in PBS containing 5% FBS. Cell concentration and viability were assessed by 0.4% AO/PI staining using a Countstar Rigel S2 automated cell counter (Countstar, Shanghai, China). Samples with viability exceeding 80% were used for downstream processing. Cell concentrations were adjusted to 700–1200 cells/μL with PBS containing 5% FBS. The cell suspension was loaded onto a microfluidic chip to generate Gel Beads-in-Emulsion (GEMs), each containing individual cells, gel beads with unique cell barcodes, and unique molecular identifiers [36]. Intracellular mRNAs were captured and reverse-transcribed using the DNBelab Library & Gel Bead Kit v3 (MGI Tech, Shenzhen, China, 940-001818-00). After cDNA purification and amplification, sequencing libraries were constructed through fragmentation, adapter ligation, and index PCR. Library quality was assessed using a Qubit 4.0 fluorometer (Thermo Fisher Scientific, Waltham, MA, USA) and Agilent 2100 Bioanalyzer (Agilent Technologies, Santa Clara, CA, USA). Libraries passing quality control were subjected to PE150 paired-end sequencing on the DNBSEQ-T7 platform, targeting an average sequencing depth of ≥20,000 reads per cell. The DNBelab C-TaiM4 platform uses a combinatorial barcoding strategy to label individual cells, and the resulting data were processed to generate a digital gene expression matrix for each cell [35,36].

### 2.7. Data Dimensionality Reduction and Cell Clustering

Raw sequencing data underwent quality control using fastp [37] to trim adapters and filter low-quality reads. Clean reads were aligned to the mouse reference genome GRCm39 using the STAR algorithm within the DNBc4tools (v2.1.2) pipeline; gene-level quantification was also performed with DNBc4tools. Subsequent analyses were conducted in R (v4.2.0) using the Seurat package (v5.0.1) to integrate data across samples, reduce dimensionality, and identify differentially expressed genes. Low-quality cells were filtered out based on the following criteria: detected genes between 200 and 9000, mitochondrial gene proportion below 25%, and removal of putative doublets. After normalization, batch effects across samples were corrected using Seurat’s integration algorithm [38]. Principal component analysis was applied to highly variable genes for dimensionality reduction, and UMAP was used for visualization and cell clustering. Cell types were assigned based on canonical marker gene expression and cross-referenced with the SingleR database. Differential expression analysis was performed using the FindMarkers function with thresholds of |avg_log2FC| > 0.25 and adjusted *p*-value < 0.05. Finally, the AddModuleScore function [39] was used to calculate an enrichment score for each cell based on a UPR^mt^-related gene set, enabling assessment of UPR^mt^ pathway activation across different cell populations.

### 2.8. H&E Staining

Colon tissues designed for H&E staining (as described in Section 2.5) were fixed in 4% paraformaldehyde, dehydrated using standard procedures, and embedded in paraffin. Sections of 4 μm thickness were cut, deparaffinized, and rehydrated [40]. The sections were then stained with hematoxylin and eosin and coverslipped with neutral gum. Digital images were acquired using a slide-scanning system (Shenzhen Shengqiang Technology Co., Ltd., Shenzhen, China, SQS-600P). At 100× and 400× magnifications, we examined the mucosal, submucosal, muscular, and serosal layers of the colon wall and assessed pathological morphological changes.

### 2.9. Enzyme-Linked Immunosorbent Assay (ELISA)

For ELISA, a piece of frozen colon tissue was thawed on ice and homogenized in pre-cooled 1× PBS at a 1:9 (*w*/*v*) ratio using a tissue grinder (KZ-III-F, Servicebio, Wuhan, China) at 4 °C. After centrifugation at 5000× *g* for 10 min at 4 °C, the supernatants were collected. The concentrations of 5-hydroxytryptamine (5-HT), tryptophan hydroxylase 1 (TPH1), and vasoactive intestinal peptide (VIP) were quantified using commercial ELISA kits (5-HT and VIP: Shanghai Zhuocai, Shanghai, China, ZC-37715 and ZC-38836; TPH1: Jiangsu Jingmei, Yancheng, China, JM-12211M1). All assays were performed according to the manufacturers’ protocols. Quantitation of each parameter was obtained by referring to standard curves generated with the provided standard.

### 2.10. Detection of Mitochondrial Function by Flow Cytometry

Fresh colon tissue allocated to flow cytometry (see Section 2.5) was minced and then digested in RPMI-1640 medium containing 0.1% collagenase type I, 0.1% collagenase type II, 0.1% neutral protease, and 0.01% DNase I at 37 °C for 30 min with intermittent trituration. Digestion was terminated by adding ice-cold RPMI-1640 medium containing 10% FBS and 2 mM EDTA. The resulting cell suspension was filtered through a 40 μm nylon cell strainer and centrifuged at 500× *g* for 5 min. The cell pellet was resuspended in 1× PBS (pH 7.4), and cell viability and concentration were determined by 0.4% AO/PI staining using a Countstar Rigel S2 automated cell counter. Finally, the cell suspension was adjusted to a density of 5–10 × 10^5^ cells per tube for subsequent flow cytometric analysis.

Mitochondrial membrane potential was assessed using a JC-1 kit (Elabscience, Wuhan, China, E-CK-A301). JC-1 was a lipophilic cationic dye that accumulated in mitochondria in a membrane potential-dependent manner. At high membrane potential, JC-1 formed J-aggregates that emitted red fluorescence. When the membrane potential collapsed, JC-1 remained in monomeric form and emitted green fluorescence. Thus, the ratio of red to green fluorescence reflected mitochondrial membrane potential. Cells were incubated with JC-1 working solution at 37 °C for 20 min, then washed and resuspended. Red (PE-A channel) and green (FITC-A channel) fluorescence were measured using a flow cytometer (Beckman Coulter, Suzhou, China, CytoFLEX), and the red/green fluorescence ratio was calculated to represent mitochondrial membrane potential. Mitochondrial superoxide levels were measured using a MitoSOX^TM^ Red kit (Beyotime, Shanghai, China, S0061S). MitoSOX Red is a live-cell-permeant dye that selectively targets mitochondria and is oxidized specifically by superoxide, but not by other reactive oxygen species, to produce red fluorescence. Cells were incubated with MitoSOX Red working solution at 37 °C for 10–30 min, then washed and resuspended. Red fluorescence was recorded (PE channel), and superoxide levels were expressed as median and mean fluorescence intensities. Single-cell suspensions were prepared from whole colonic tissue without applying SMC-specific surface marker gating. Consequently, the JC-1 and MitoSOX data reflected the mitochondrial membrane potential and mtROS averaged across the entire dissociated cell population, rather than solely SMCs.

### 2.11. Cell Culture and Lentiviral Infection

*Cell culture:* Primary mouse colonic smooth muscle cells (Procell, Wuhan, China, CP-M044) were maintained in a ready-to-use complete smooth muscle cell medium (Procell, CM-M044; containing all components required for growth of cells) at 37 °C with 5% CO_2_. Cells between passages 3 and 5 were used for all experiments. For passaging and harvesting, cells were detached using 0.25% trypsin-EDTA (Shanghai Yuanpei, Shanghai, China) for 2–3 min at 37 °C. Then, complete medium was added to terminate digestion, and the mixture was centrifuged at 250× *g* for 5 min at room temperature. The resulting pellet was resuspended either in fresh complete medium for further culture or in 1× PBS for downstream assays.

*Lentiviral Infection:* Lentiviral constructs were obtained as described in Section 2.2. For lentiviral transduction, cells in the logarithmic growth phase were seeded into 6-well plates at two different densities according to the experimental purpose: for transduction efficiency assays, cells were seeded at 3 × 10^4^ cells/mL; for all other downstream analyses, cells were seeded at 1 × 10^5^ cells/mL. After cell adherence and upon reaching approximately 70% confluence, the medium was replaced with complete medium containing 8 μg/mL Polybrene, and the appropriate lentivirus was added at a multiplicity of infection (MOI) of 100. Following 16 h of exposure, the virus-containing medium was removed, the medium was refreshed with complete medium, and the cells were cultured for an additional 48 h. After 48 h, cells were harvested and the same pellet was split for ATP, Western blotting, RT-qPCR, with separate cultures for transmission electron microscopy and immunofluorescence.

Five experimental groups were included: (1) Control (untransduced cells), (2) LV-HSF1-OE (cells transduced with lentivirus overexpressing full-length mouse HSF1), (3) LV-Veh (cells transduced with an empty vector control), (4) LV-shHSF1 (cells transduced with lentivirus carrying an shRNA targeting mouse HSF1), and (5) LV-shVeh (cells transduced with lentivirus containing a non-targeting scrambled shRNA).

### 2.12. Transmission Electron Microscopy

For colon tissue, the segment allocated to transmission electron microscopy (see Section 2.5) was fixed in 2.5% glutaraldehyde at 4 °C, followed by post-fixation with 1% osmium tetroxide. For tissue samples, fixation was performed by immersion immediately after dissection. For cultured cells, cells were harvested as described in Section 2.11 by centrifugation at 250× *g* for 5 min and the resulting pellets were fixed as intact aggregates. After dehydration through a graded acetone series, samples were infiltrated and embedded in epoxy resin. Ultrathin sections (60–90 nm) were cut using an ultramicrotome (LEICA, Wetzlar, Germany, UC7rt) and collected on copper grids. Sections were double-stained with uranyl acetate and lead citrate. Uranyl acetate bound to nucleic acids and phospholipids, while lead citrate interacted with proteins and membrane structures. Together, these stains provided electron density to cellular compartments [41]. This staining protocol allowed for clear visualization of organelles and membrane boundaries. SMCs were identified within the muscularis propria by their spindle-shaped morphology, abundant cytoplasmic dense bodies, subsarcolemmal dense plaques, caveolae, and surrounding basal lamina [42]. In cultured SMC pellets, the same criteria applied, with additional confirmation from the homogeneous population of cells displaying SMC morphology. Mitochondrial morphology (including cristae integrity and matrix electron density) and abundance in SMCs were assessed.

### 2.13. Determination of ATP Content

ATP levels were measured in both colon tissue and cultured SMCs using a colorimetric ATP assay kit (Nanjing Jiancheng, A095-1-1, Nanjing, China). For colon tissue, frozen samples were thawed and homogenized in ice-cold normal saline (1:9, *w*/*v*) using a tissue grinder (KZ-III-F, Servicebio, Wuhan, China) at 4 °C to prepare 10% homogenates. An aliquot was taken for protein quantification, and the remaining homogenate was placed in a boiling water bath for 10 min, vortexed for 1 min, and centrifuged at 1400× *g* for 10 min at 4 °C. The supernatant was collected for ATP measurement. For cultured SMCs, cells were harvested as described in Section 2.11. The cell pellet was resuspended in 300–500 μL of ice-cold normal saline and lysed by ultrasonication on ice. An aliquot of the lysate was taken for protein quantification, and the remaining lysate was placed in a boiling water bath for 10 min, vortexed for 1 min, and centrifuged at 12,000× *g* for 10 min at 4 °C. The supernatant was used for ATP measurement.

For both tissue and cell samples, the ATP-containing supernatants were reacted with the kit working solution following the manufacturer’s protocol. Samples were incubated at 37 °C for 30 min, and absorbance was read at 636 nm using a microplate reader (Molecular Devices, Sunnyvale, CA, USA, SpectraMAX Plus 384) for tissue samples and a spectrophotometer (Shanghai Youke, Shanghai, China, UV752N) for cell samples. ATP concentrations were calculated from a standard curve and expressed as mmol/g protein.

For protein quantification, both kits are Bradford-based, but sample matrices differ. Tissue protein was measured from unboiled saline homogenate (clean matrix), optimally quantified by the Beyotime P0006 kit (Beyotime, Shanghai, China, P0006). Cultured cell protein was measured from ultrasonication lysate containing membrane components (complex matrix), for which the Jiancheng A045-2-2 kit (Nanjing jiancheng, Nanjing, China, A045-2-2) is more suitable.

### 2.14. Assessment of Mitochondrial Respiratory Chain Complexes

For colon tissue, the activities of respiratory chain complexes I–V were measured using colorimetric assay kits (Beijing Solarbio Science & Technology Co., Ltd., Beijing, China: Complex I, BC0515; Complex II, BC3235; Complex III, BC3245; Complex IV, BC0945; Complex V, BC1445). A piece of frozen colon tissue was homogenized in 1.0 mL of ice-cold extraction buffer (250 mM sucrose, 10 mM Tris-HCl, 1 mM EDTA, pH 7.4) using a tissue grinder on ice. The homogenate was centrifuged at 600× *g* for 10 min at 4 °C. The supernatant was transferred to a new tube and centrifuged at 11,000× *g* for 15 min at 4 °C. The resulting pellet (mitochondrial fraction) was resuspended in extraction buffer and disrupted by sonication. The sonicated suspension was then used for the assays following each kit’s protocol. Absorbance was measured at the wavelengths specified by the manufacturer (340 nm, 605 nm, 550 nm, 550 nm, and 660 nm for complexes I–V, respectively). Complex activities were calculated using the formulas provided with the kits and expressed as U/g mass.

For cultured colonic SMCs, the protein expression levels of mitochondrial respiratory chain complex subunits (ATP5A, NDUFA9, COX1, SDHA, and UQCRC1) were assessed by Western blotting as described in Section 2.15.

### 2.15. Immunofluorescence (IF)

Colon tissues allocated to immunofluorescence (see Section 2.5) were fixed in paraformaldehyde, embedded in paraffin, and sectioned at 4 μm thickness. Paraffin sections were deparaffinized, subjected to antigen retrieval, and blocked, followed by incubation with appropriate primary antibodies overnight at 4 °C. For tissue samples, a double-labeling approach was used: a primary antibody against α-SMA was co-incubated with a primary antibody against COX IV, HSF1, HSP60, LONP1, or mtHSP70 overnight at 4 °C, followed by CY3- and CY5-conjugated secondary antibodies. For cell samples, cells seeded on coverslips were fixed with 4% paraformaldehyde for 15 min at room temperature, permeabilized with 0.1% Triton X-100 for 10 min, and blocked. Cells were then incubated with a primary antibody against COX and one of the following primary antibodies: anti-HSF1, anti-HSP60, anti-LONP1, or anti-mtHSP70, followed by incubation with HRP-conjugated secondary antibodies. Tyramide signal amplification was performed using tyramide-conjugated fluorophores (iF647 or iF750) according to the manufacturer’s instructions; HRP catalyzes the deposition of tyramide-fluorophore conjugates onto tyrosine residues near the target protein, resulting in localized signal amplification. All samples were counterstained with DAPI for 10 min at room temperature to label nuclei. Images were acquired using an OLYMPUS VS200 slide scanner (Olympus, Tokyo, Japan). For CY3, excitation/emission wavelengths were 548/561 nm; for CY5, 650/670 nm; for iF647, 650/665 nm; for iF750, 755/780 nm; and for DAPI, 358/461 nm. Double-positive cells were quantified with Image-Pro Plus. α-SMA is also expressed in myofibroblasts and subepithelial fibroblasts. To minimize the contribution of these non-SMC populations, quantification was restricted to the muscularis propria, where SMCs are the predominant α-SMA-expressing cell type. Detailed information on the primary and secondary antibodies is provided in Table S2.

### 2.16. Western Blotting

A piece of frozen colon tissue were homogenized in RIPA lysis buffer (Beyotime, P0013) supplemented with protease inhibitors using a tissue grinder (KZ-III-F, Servicebio, Wuhan, China) at 4 °C. The homogenate was sonicated on ice and centrifuged at 13,000× *g* for 10 min at 4 °C. The supernatant was collected. For cultured SMCs, cells were harvested as described in Section 2.11 and lysed in RIPA lysis buffer supplemented with protease inhibitors. The lysate was sonicated on ice and centrifuged at 13,000× *g* for 10 min at 4 °C. Protein concentrations in both tissue and cell lysates were determined by the BCA method (Biosharp, Hefei, China, BL521C). Proteins were denatured at 95 °C for 10 min, separated by SDS–PAGE using a 10% resolving gel, and transferred onto PVDF membranes (Servicebio, G6050-0.45). Membranes were blocked with 5% nonfat milk in (Tris Buffered Saline with Twee) TBST at room temperature for 2 h and then incubated with appropriate primary antibodies at 4 °C overnight. After washing with TBST, membranes were incubated with an HRP-conjugated secondary antibody (Abclonal, Wuhan, China, AS014) at room temperature for 2 h. Blots were developed using an ECL chemiluminescent substrate (Biosharp, BL520B), and images were acquired with a Tanon 5200 Multi system. The integrated optical density of each band was measured using Gel-Pro Analyzer 4 software, and relative protein expression was calculated after normalization to β-actin (Abclonal, AC026). The following proteins were assessed: HSF1, HSP60, mtHSP70, and LONP1 in both tissue and cultured cell samples; ATP5A, NDUFA9, COX1, SDHA, and UQCRC1 in cell samples. Detailed antibody information is listed in Table S2, and the uncropped full-blot images are provided in Figure S1.

### 2.17. Reverse Transcription Quantitative Polymerase Chain Reaction (RT-qPCR)

A piece of frozen colon tissue were homogenized in RNA lysis buffer (Buffer LB, biosharp, BL1365A) using a tissue grinder (KZ-III-F, Servicebio, Wuhan, China) at 4 °C. For cultured SMCs, cells were harvested as described in Section 2.11, and the washed cell pellet was directly lysed in Buffer LB. Total RNA was extracted from both tissue homogenates and cell lysates using a Total RNA Extraction Kit (biosharp, BL1365A) according to the manufacturer’s instructions, which included phase separation by centrifugation at 13,000× *g* for 15 min at 4 °C. Genomic DNA was removed, and RNA was reverse transcribed into complementary DNA using the PrimeScript^TM^ RT Reagent Kit with gDNA Eraser (Takara, Beijing, China, RR047A). Using the resulting cDNA as a template, amplification was performed on a QuantStudio^TM^ instrument with TB Green Premix Ex Taq^TM^ II FAST qPCR (Takara, Beijing, China, CN830A). The thermal cycling conditions were: pre-denaturation at 95 °C for 1 min, followed by 45 cycles of 95 °C for 10 s, 55 °C for 30 s, and 72 °C for 10 s. β-Actin was used as the internal reference, and relative expression levels were calculated using the 2^−△△CT^ method. HSF1, HSP60, LONP1, and mtHSP70 mRNAs were assessed in both colon tissue and cultured SMCs. Primer sequences are provided in Table S3.

### 2.18. Data Analysis

All data analyses were conducted in a blinded manner. Statistical analyses were performed using SPSS 25.0 software. The number of experimental replicates (n) is indicated in the figure legends. Data are presented as the mean ± SD. Comparisons between two groups were analyzed using unpaired two-tailed Student’s *t*-tests, and comparisons among multiple groups were analyzed using one-way or two-way ANOVA, as appropriate, followed by Tukey’s post hoc test. Statistical significance was defined as follows: * *p* < 0.05, ** *p* < 0.01, *** *p* < 0.001 for overexpression vs. control, and ^#^ *p* < 0.05, ^##^ *p* < 0.01, ^###^ *p* < 0.001 for inhibition vs. control. This definition applied to both in vivo and in vitro experiments.

## 3. Results

### 3.1. Loperamide Administration Established an FC Mouse Model Characterized by Slowed Gut Motility

**Figure 2:**
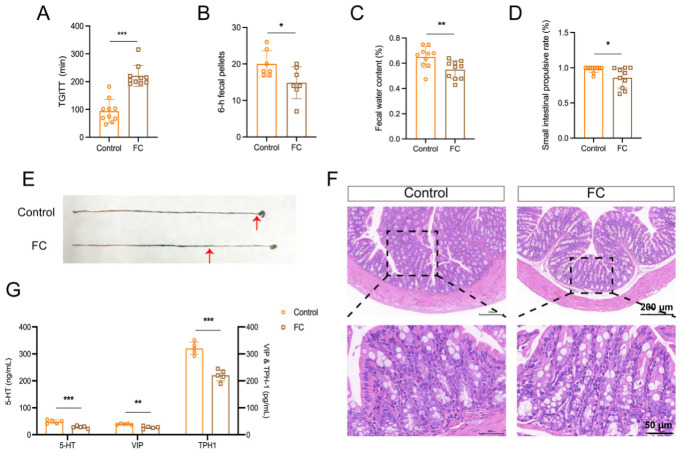
Loperamide treatment induced constipation in mice. (**A**–**D**) Assessment of gut motility function in FC versus control mice (n = 7–10). (**A**) TGITT, (**B**) 6-h fecal pellets, (**C**) fecal water content, and (**D**) small intestinal propulsive rate. (**E**) Representative images of small intestinal propulsive rate from control and FC mice, the red arrows indicated the terminus of the blue-stained segment. (**F**) Representative images of H&E-stained colon sections showing histological changes (n = 3). Scale bar, 200 µm (upper, X100) and 50 µm (lower, X400). (**G**) ELISA quantification of gut motility-related markers (n = 5): 5-HT, VIP, and TPH-1. Data are presented as the mean ± SD, * *p* < 0.05, ** *p* < 0.01, *** *p* < 0.001 vs. Control (unpaired two-tailed Student’s *t*-test). For detailed statistical information, see Table S4.

To evaluate gut motility function, an FC mouse model was first established using a well-characterized loperamide-induced constipation protocol that has been extensively validated by our group and other laboratories [31,32,33,34]. Mice received intragastric administration of loperamide hydrochloride suspension for 14 days, whereas control mice received 0.9% saline. Several physiology parameters associated with gut motility were subsequently measured, including TGITT, 6-h fecal pellet output, fecal water content, and the small intestinal propulsive rate [31,32,33,34]. Compared with the control group, mice in the FC group exhibited a pronounced constipation phenotype, characterized by a significant prolongation of TGITT (*p* < 0.001) accompanied by marked reductions in 6-h fecal pellet output, fecal water content (*p* < 0.05, *p* < 0.01), and the small intestinal propulsive rate (*p* < 0.05; Figure 2A–E). Histological examination using H&E staining demonstrated that the overall architecture of the colonic mucosa, muscularis, and serosa remained intact in both groups, with no apparent organic damage, indicating that the model represents a functional motility disorder rather than an organic structural abnormality (Figure 2F). In addition, ELISA measurements revealed that colonic levels of 5-HT (produced primarily by enterochromaffin cells), VIP (released by enteric inhibitory motor neurons), and TPH-1 (the rate-limiting enzyme for 5-HT synthesis in enterochromaffin cells) were significantly reduced in FC mice (*p* < 0.001 for 5-HT and TPH-1, *p* < 0.01 for VIP; Figure 2G). Taken together, these results confirmed the successful establishment of an FC mouse model characterized primarily by slowed gut motility.

### 3.2. Single-Cell Transcriptomics Indicated Suppression of the UPR^mt^ Pathway in Colonic SMCs of FC Mice

**Figure 3:**
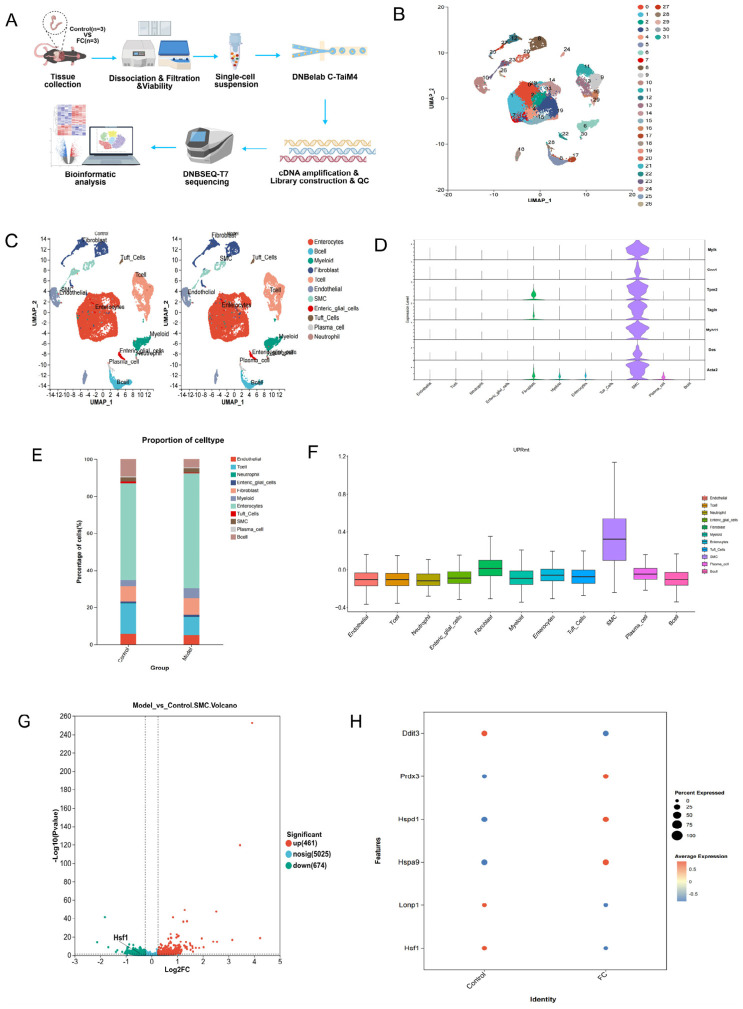
Single-cell transcriptomics identified UPR^mt^ suppression in colonic smooth muscle cells of FC mice. (**A**) Workflow for single-cell seq (n = 3 per group). (**B**) UMAP projection of 32 transcriptionally distinct clusters integrated from all samples and (**C**) 11 major cell types between groups. (**D**) Violin plots showing expression levels of smooth muscle cell (SMC)-specific markers across identified cell types. (**E**) Composition of cell types in control versus FC mice. (**F**) UPR^mt^ gene signature scores across cell types, presented as box plots. (**G**) Volcano plot of differentially expressed genes in SMCs from FC mice compared with controls. (**H**) Bubble plot visualizing the expression of UPR^mt^-related genes in SMCs of control and FC mice.

To comprehensively characterize cellular changes associated with FC, unbiased single-cell RNA sequencing was performed on colon tissues from both control and FC mice (Figure 3A), generating a detailed cellular atlas that resolved 11 major cell types within the colon (Figure 3B,C). Among these populations, SMCs were identified based on strong expression of established markers of contractile and cytoskeletal function, including myosin light chain kinase (Mylk), calponin 1 (Cnn1), tropomyosin 2 (Tpm2), transgelin (Tagln), myosin heavy chain 11 (Myh11), desmin (Des), and alpha-smooth muscle actin (Acta2) (Figure 3D and Figure S2). Comparison of cell-type proportions between groups revealed a modest reduction in the relative abundance of SMCs in FC mice (Figure 3E), suggesting that alterations in SMC integrity or function may have contributed to the development of hypomotility-associated constipation. To extend these transcriptomic findings, the AddModuleScore function was applied using a predefined UPR^mt^ gene set to calculate an enrichment score for each cell, enabling assessment of UPR^mt^ pathway activation across different cell populations. Notably, SMCs displayed the highest module scores among all identified cell populations (Figure 3F), indicating that these cells may play a central role in maintaining mitochondrial proteostasis within the colon. Differential expression analysis between control and FC groups revealed significant downregulation of several genes encoding core components of the UPR^mt^ pathway in FC mice, including the transcription factor HSF1 and the mitochondrial protease LONP1 (Figure 3G,H). Collectively, these transcriptomic findings suggest that the UPR^mt^ pathway is suppressed in colonic SMCs under FC conditions.

### 3.3. Downregulation of the UPR^mt^ Pathway Was Associated with Mitochondrial Dysfunction in Colonic SMCs of FC Mice

**Figure 4:**
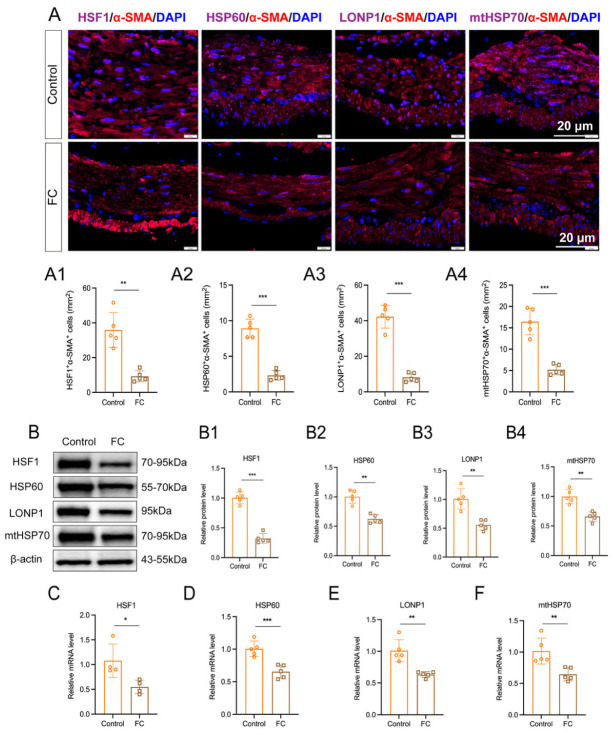
UPR^mt^ pathway components were downregulated in colonic SMCs of FC mice. (**A**) Representative immunofluorescence images of UPR^mt^-related proteins (HSF1, HSP60, mtHSP70, and LONP1, purple), α-smooth muscle actin (α-SMA, red), and DAPI (blue) in the colon tissues of control and FC groups. Scale bar, 20 μm, X400. Single-channel images for the merged panels are shown in Figure S3. (**A1**–**A4**) Quantification of the number of α-SMA-positive cells that were co-positive for HSF1, HSP60, mtHSP70, and LONP1 in control and FC groups (n = 5). (**B**) Representative Western blot images of HSF1, HSP60, mtHSP70, and LONP1 protein levels in colon tissues. (**B1**–**B4**) Quantification of the Western blot band intensities for these proteins, normalized to β-actin (n = 5). (Original Western Blot Images see Supplementary Figure S1.) (**C**–**F**) RT-qPCR analysis of HSF1, HSP60, mtHSP70, and LONP1 mRNA levels in colon tissues (n = 4–5). Data are presented as the mean ± SD. * *p* < 0.05, ** *p* < 0.01, *** *p* < 0.001 vs. Control (unpaired two-tailed Student’s *t*-test). For detailed statistical information, see Table S5.

To validate the transcriptomic findings, the established FC mouse model was used to examine the expression of UPR^mt^-related components specifically within colonic smooth muscle. Immunofluorescence (IF) co-localization analysis was performed on colon tissue sections to assess the distribution of the regulatory factor HSF1, the molecular chaperones HSP60 and mtHSP70, and the protease LONP1 relative to the smooth muscle marker α-SMA within the muscularis propria. In control mice, strong fluorescence signals for HSF1, HSP60, mtHSP70, and LONP1 were predominantly localized within the muscular layer and showed clear co-localization with α-SMA-positive cells. In contrast, FC mice exhibited markedly reduced fluorescence intensity for all four proteins within α-SMA-positive regions (Figure 4A and Figure S3). Quantitative analysis further confirmed these observations, demonstrating that the numbers of α-SMA^+^HSF1^+^, α-SMA^+^HSP60^+^, α-SMA^+^mtHSP70^+^, and α-SMA^+^LONP1^+^ cells were significantly lower in FC mice compared with controls (*p* < 0.01 for HSF1, *p* < 0.001 for the other three; Figure 4A1–A4). Consistent with these findings, Western blot and RT-qPCR analyses revealed corresponding decreases in the protein and mRNA levels of all four molecules in the FC group (*p* < 0.05 for HSF1 protein, *p* < 0.001 for the other three proteins; *p* < 0.05 for HSF1 mRNA, *p* < 0.001 for HSP60 mRNA, and *p* < 0.01 for mtHSP70 and LONP1 mRNA; Figure 4B–F).

**Figure 5:**
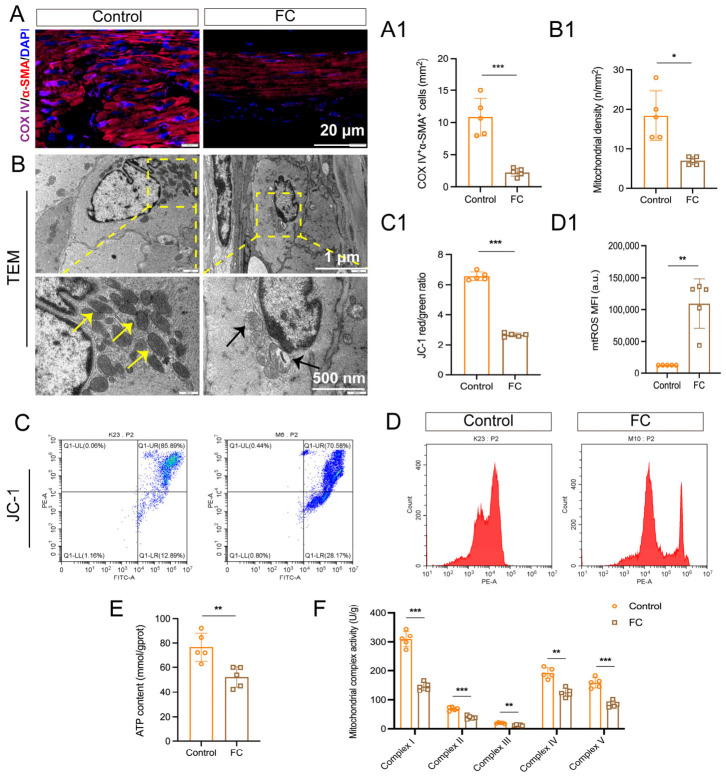
UPR^mt^ suppression was accompanied by mitochondrial dysfunction in colonic SMCs of FC mice. (**A**) Representative IF images of COX IV (purple), α-SMA (red), and DAPI (blue) in the colon tissues from control and FC mice. Scale bar, 20 μm, X400. Single-channel images are shown in Figure S4. (**A1**) Quantification of the number of α-SMA-positive cells that were co-positive for COX IV (n = 5). (**B**) Representative TEM images of colonic SMCs from control and FC mice. Yellow arrows indicate normal mitochondria, and black arrows indicate damaged mitochondria with fragmented cristae. Scale bar, 1 μm (upper panels, X1200) and 500 nm (lower panels, X3000). (**B1**) Quantitative analysis of mitochondrial density, expressed as the number of mitochondria per mm^2^ of cytoplasmic area (3 fields per mouse, n = 5). (**C**) Representative flow cytometry density plots of JC-1 staining. The *x*-axis (FITC-A) represents the green fluorescence intensity (JC-1 monomers), the *y*-axis (PE-A) represents the red fluorescence intensity (JC-1 aggregates). (**C1**) Quantitative analysis of the JC-1 red/green fluorescence ratio (n = 5). (**D**) Representative flow cytometry histograms of MitoSOX Red staining. The *x*-axis represents the fluorescence intensity (PE-A channel), and the *y*-axis represents the cell count. (**D1**) Quantitative analysis of the mean fluorescence intensity (MFI) of mtROS (n = 5). (**E**) ATP levels and (**F**) the activities of complexes I–V in colon tissues (n = 5 for ATP, n = 4–5 for complexes), as measured by colorimetric assays. All data are presented as the mean ± SD. * *p* < 0.05, ** *p* < 0.01, *** *p* < 0.001 vs. Control (unpaired two-tailed Student’s *t*-test was used for comparisons). For detailed statistical information, see Table S6.

Given the suppression of key UPR^mt^ components, mitochondrial morphology and function in colonic SMCs were subsequently examined. Co-localization analysis of α-SMA with the mitochondrial marker COX IV in colon tissue sections revealed a weakened mitochondrial network signal in SMCs from FC mice (Figure 5A and Figure S4), accompanied by a significant reduction in the number of co-labeled cells, suggesting a loss of mitochondrial mass or abundance (Figure 5A1). Transmission electron microscopy further confirmed these structural abnormalities. Mitochondria within FC SMCs displayed disrupted cristae architecture, prominent vacuolization (Figure 5B), and a significant reduction in mitochondrial density per unit area (*p* < 0.05; Figure 5B1). Functional assays demonstrated parallel impairments in mitochondrial activity. Flow cytometry analysis with JC-1 and MitoSOX Red revealed a shift toward lower membrane potential and higher superoxide levels in the FC group (Figure 5C,D). Compared with controls, FC mice exhibited a reduced mitochondrial membrane potential (JC-1 red/green ratio, *p* < 0.001; Figure 5C1), elevated mtROS production (*p* < 0.01; Figure 5D1), decreased ATP content (*p* < 0.01; Figure 5E), as well as markedly diminished activities of respiratory chain complexes I–V, both measured by colorimetric assays (*p* < 0.001 for complexes I, II, and V, *p* < 0.01 for complexes III and IV; Figure 5F). Taken together, these findings indicate that suppression of the UPR^mt^ pathway is closely associated with both structural and functional mitochondrial damage in colonic SMCs of FC mice.

### 3.4. Bidirectional Manipulation of HSF1 Bidirectionally Altered UPR^mt^ Pathway Expression in Colonic SMCs of FC Mice

**Figure 6:**
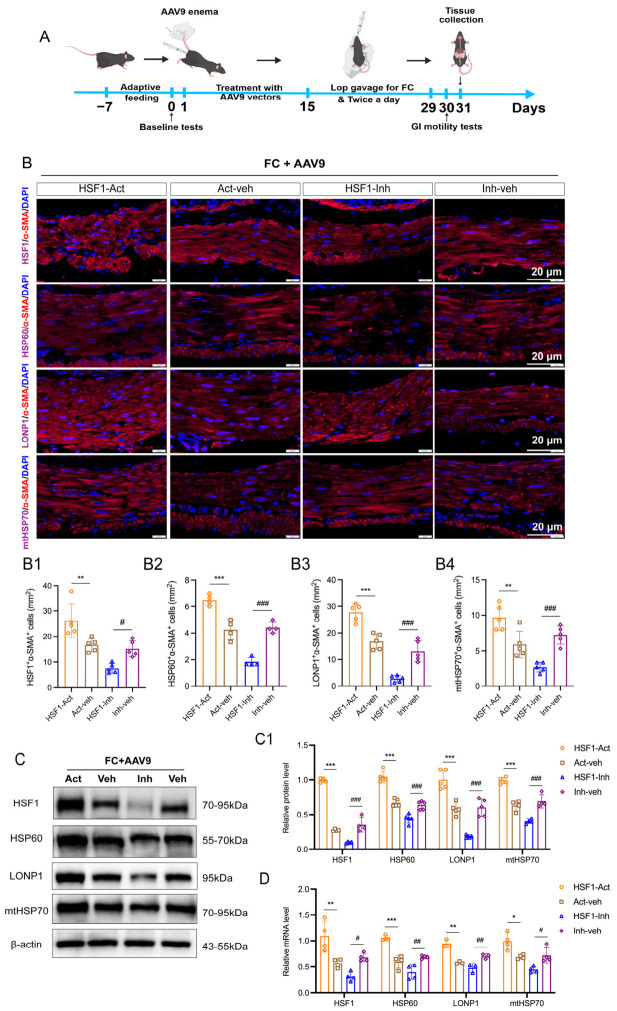
Modulation of HSF1 bidirectionally altered UPR^mt^ pathway expression in colonic SMCs of FC mice. (**A**) Scheme of the experimental timeline for mice exposure to AAV9 vectors. (**B**) Representative IF images of UPR^mt^-related proteins (HSF1, HSP60, mtHSP70, and LONP1, purple), α-SMA (red), and DAPI (blue) in the colon tissues across the four groups: Act-Veh, HSF1+Act, Inh-Veh, and HSF1+Inh. Scale bar, 20 μm, X400. Single-channel images for the merged panels are shown in Figure S6. (**B1**–**B4**) Quantification of the number of α-SMA-positive cells that were co-positive for HSF1, HSP60, mtHSP70, and LONP1 in the four groups (n = 4–5). (**C**) Representative Western blot images of HSF1, HSP60, mtHSP70, and LONP1 in colon tissues from the indicated groups. (**C1**) Quantification of the Western blot band intensities for these proteins, normalized to β-actin (n = 4–5). (Original Western Blot Images see Supplementary Figure S1.) (**D**) RT-qPCR analysis of HSF1, HSP60, mtHSP70 and LONP1 mRNA levels in colon tissues (n = 3–4). Data are presented as the mean ± SD, * *p* < 0.05, ** *p* < 0.01, *** *p* < 0.001 for HSF1+Act vs. Act-Veh or ^#^ *p* < 0.05, ^##^ *p* < 0.01, ^###^ *p* < 0.001 for HSF1+Inh vs. Inh-Veh (one-way ANOVA with Tukey’s post hoc test). HSF1-Act: FC mice instilled with AAV9 encoding HSF1; Act-Veh: FC instilled with HSF1-activator AAV9 empty vector; HSF1+Inh: FC mice instilled with shAAV9 targeting HSF1; Inh-Veh: FC treated with HSF1-inhibitor AAV9 scrambled control. For detailed statistical information, see Table S7.

To further investigate the functional role of the UPR^mt^ pathway in FC, HSF1–an essential upstream transcriptional regulator of the pathway–was selected for bidirectional manipulation in vivo (Figure 6A). AAV9 vectors designed to target colonic SMCs were delivered to FC mice by enema, and corresponding empty-vector control groups were established. To evaluate tissue distribution of the viral vectors, randomly selected mice from each group (n = 3 per group) were examined; these mice were excluded from subsequent functional experiments. GFP fluorescence imaging demonstrated that viral transduction occurred predominantly within the colonic muscularis layer, whereas only weak fluorescence signals were observed in the mucosal layer (Figure S5A). Successful viral expression was further confirmed by Western blot and RT-qPCR analyses (both *p* < 0.05; Figure S5B–D). Importantly, HSF1 expression did not differ significantly between empty-vector control mice and the FC group, indicating that the AAV vector itself did not substantially affect basal HSF1 expression (Figure S5B–D). Overall, GFP fluorescence was confined to the muscularis layer, and HSF1 mRNA and protein levels were bidirectionally modulated in the expected directions, confirming that AAV9 delivery reached colonic SMCs.

Subsequently, the effects of HSF1 manipulation on core components of the UPR^mt^ pathway were evaluated. IF analysis of colon tissue sections revealed that, compared with the Act-Veh group, the HSF1-Act group exhibited markedly increased fluorescence signals for HSF1, HSP60, mtHSP70, and LONP1 within the colonic smooth muscle layer (Figure 6B and Figure S6). Quantitative analysis confirmed a significant increase in the numbers of double-positive cells for each protein with α-SMA in the HSF1-Act group compared with Act-Veh controls (*p* < 0.001 for HSP60 and mtHSP70, *p* < 0.01 for HSF1 and LONP1; Figure 6B1–B4). In contrast, the HSF1-Inh group exhibited reduced fluorescence signals (Figure 6B) and fewer double-positive cells than the Inh-Veh group (*p* < 0.05 for HSF1, *p* < 0.001 for the others; Figure 6B1–B4).

Western blot and RT-qPCR analyses consistently confirmed these regulatory effects at both the protein and transcriptional levels (Figure 6C,D). Compared with their respective empty-vector controls, HSF1 activation significantly increased the protein (all *p* < 0.001) and mRNA levels of HSF1, HSP60, mtHSP70, and LONP1 (*p* < 0.001 for HSP60, *p* < 0.05 to *p* < 0.01 for the others), whereas HSF1 knockdown significantly decreased the protein (all *p* < 0.001) and mRNA expressions (*p* < 0.01 for HSP60 and mtHSP70, *p* < 0.05 for HSF1 and LONP1) of these molecules. Collectively, these findings demonstrate that bidirectional manipulation of HSF1 directly regulates the activity of the UPR^mt^ pathway in colonic SMCs under FC conditions.

### 3.5. Activation of the UPR^mt^ Pathway Preserved Mitochondrial Homeostasis and Improved Gut Motility in FC Mice

**Figure 7:**
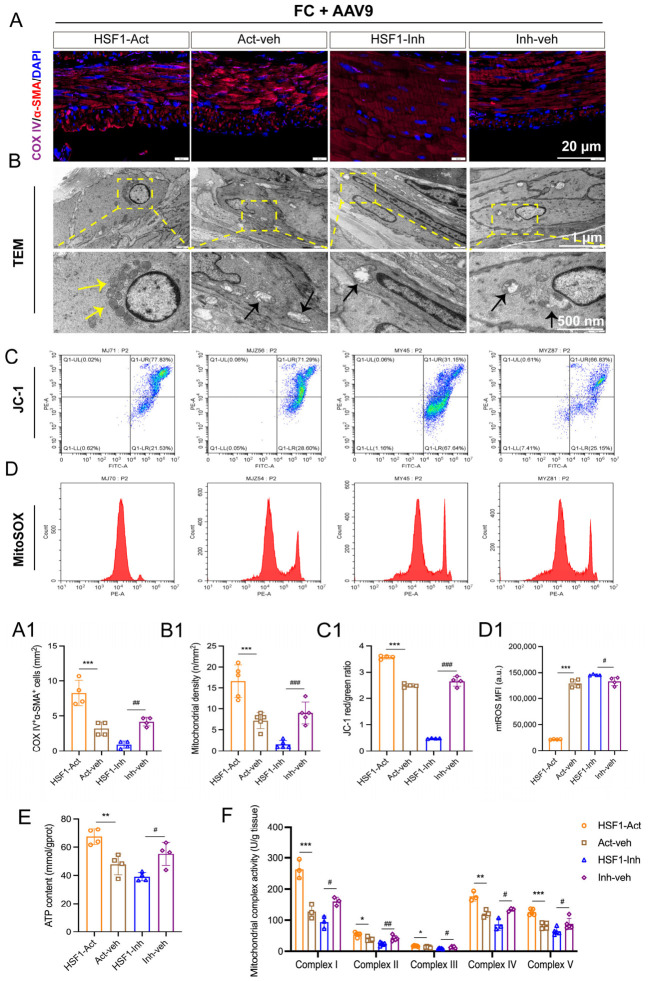
Activation of the UPR^mt^ pathway preserved mitochondrial homeostasis in FC mice. (**A**) Representative IF images of COX IV (purple), α-SMA (red), and DAPI (blue) in the colon tissues across the four groups: Act-Veh, HSF1+Act, Inh-Veh, and HSF1+Inh. Scale bar, 20 μm, X400. Single-channel images are shown in Figure S7. (**A1**) Quantification of the number of α-SMA-positive cells that were co-positive for COX IV (n = 4). (**B**) Representative TEM images of colonic SMCs from mouse tissue. Yellow arrows indicate normal mitochondria, and black arrows indicate damaged mitochondria with fragmented cristae. Scale bar, 1 μm (upper panels, X1200) and 500 nm (lower panels, X3000). (**B1**) Quantitative analysis of mitochondrial density, expressed as the number of mitochondria per mm^2^ of cytoplasmic area (3 fields per mouse, n = 5). (**C**) Representative flow cytometry density plots of JC-1 staining and (**D**) histograms of MitoSOX Red staining in colonic tissue from the Act-Veh, HSF1+Act, Inh-Veh, and HSF1+Inh groups, with (**C1**) quantitative data shown as the JC-1 red/green fluorescence ratio and (**D1**) the mean fluorescence intensity (MFI) of mitochondrial ROS (mtROS), respectively (n = 4). (**E**) ATP levels and (**F**) the activities of complexes I–V in colon tissues (n = 4 for ATP, n = 3–4 for complexes), as measured by colorimetric assays. Data are presented as the mean ± SD, * *p* < 0.05, ** *p* < 0.01, *** *p* < 0.001 for HSF1+Act vs. Act-Veh or ^#^ *p* < 0.05, ^##^ *p* < 0.01, ^###^ *p* < 0.001 for HSF1+Inh vs. Inh-Veh (one-way ANOVA with Tukey’s post hoc test was used). For the full names of the groups, see the note above. For detailed statistical information, see Table S8.

The physiological consequences of UPR^mt^ modulation by HSF1 overexpression were next evaluated with respect to mitochondrial homeostasis and gut motility. IF staining of colon tissue showed that COX IV signals within the colonic smooth muscle layer were markedly increased in the HSF1-Act group versus Act-Veh controls, whereas a reduction in COX IV fluorescence was observed in the HSF1-Inh group versus Inh-Veh controls (Figure 7A). Quantitative analysis demonstrated a significant increase in α-SMA^+^COX IV^+^ co-labeled cells in the HSF1-Act group (*p* < 0.001), whereas the HSF1-Inh group exhibited a significant decrease in these cells (*p* < 0.01; Figure 7A1 and Figure S7). Transmission electron microscopy provided further confirmation of these mitochondrial changes. Compared with their respective vehicle controls, mitochondria in the HSF1-Act group exhibited well-preserved morphology, with clearly defined cristae structures and a significantly higher mitochondrial density per unit area (*p* < 0.001; Figure 7B,B1). In contrast, mitochondria in the HSF1-Inh group displayed pronounced vacuolization, disrupted cristae architecture, and a marked reduction in mitochondrial density (*p* < 0.001; Figure 7B,B1).

Functional assays supported these structural observations. Compared with vehicle-treated controls, mice in the HSF1-Act group showed a significant increase in mitochondrial membrane potential (*p* < 0.001) and ATP production (*p* < 0.01), together with a significant reduction in mtROS levels (*p* < 0.001). Conversely, the HSF1-Inh group exhibited further declines in mitochondrial membrane potential and ATP levels accompanied by increased mtROS production (*p* < 0.001, *p* < 0.05, *p* < 0.05; Figure 7C–E). Consistently, the activities of respiratory chain complexes I–V were substantially restored in the HSF1-Act group (*p* < 0.001 for complexes I and V, *p* < 0.05 to *p* < 0.01 for complexes II-IV; Figure 7F) but were further suppressed in the HSF1-Inh group (all *p* < 0.05; Figure 7F).

**Figure 8:**
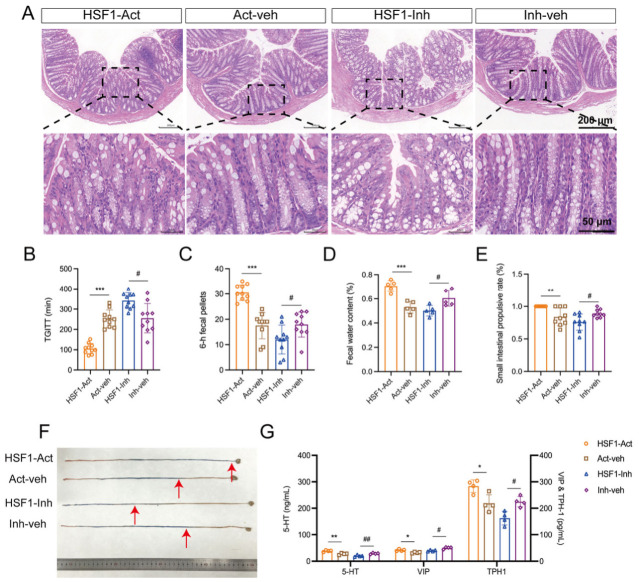
Activation of the UPR^mt^ pathway alleviated gut motility dysfunction in FC mice. (**A**) Representative images of H&E-stained colon sections from the Act-Veh, HSF1+Act, Inh-Veh, and HSF1+Inh groups (n = 3). Scale bar, 200 µm (upper, X100) and 50 µm (lower, X400). (**B**–**E**) Assessment of constipation-related behavioral phenotypes (n = 5–10). (**B**) TGITT, (**C**) 6-h fecal pallets, (**D**) fecal water content, and (**E**) small intestinal propulsive rate. (**F**) Representative images of small intestinal transit rate in mice from each group, the red arrows indicated the terminus of the blue-stained segment. (**G**) ELISA quantification of 5-HT, VIP, and TPH1 (n = 5). Data are presented as the mean ± SD, * *p* < 0.05, ** *p* < 0.01, *** *p* < 0.001 for HSF1+Act vs. Act-Veh or ^#^ *p* < 0.05, ^##^ *p* < 0.01 for HSF1+Inh vs. Inh-Veh (one-way ANOVA with Tukey’s post hoc test was used). For the full names of the groups, see the note above. For detailed statistical information, see Table S9.

Importantly, these mitochondrial changes translated into clear physiological effects on gut motility. Histological analysis after H&E staining (Figure 8A) confirmed that colonic tissue architecture remained intact in all experimental groups, with no detectable structural damage in the mucosa, muscularis, or serosa, indicating that the observed phenotypic changes reflected functional regulation rather than structural injury. Behavioral assessments demonstrated that mice in the HSF1-Act group showed significant improvement in constipation-related parameters, including shortened TGITT, increased 6-h fecal pellet output, higher fecal water content, and enhanced small intestinal propulsive rate (all *p* < 0.01; Figure 8B–F). In contrast, mice in the HSF1-Inh group exhibited further deterioration of gut motility (all *p* < 0.05; Figure 8B–F).

Consistent with these physiological findings, ELISA measurements revealed that colon tissues from HSF1-Act mice contained significantly higher levels of 5-HT, VIP, and TPH-1 compared with Act-Veh controls (*p* < 0.01 for 5-HT, *p* < 0.05 for VIP and TPH-1; Figure 8G), whereas all three factors were further reduced in the HSF1-Inh group relative to the Inh-Veh group (*p* < 0.01 for 5-HT, *p* < 0.05 for VIP and TPH-1; Figure 8G). Collectively, these results demonstrate that activation of the UPR^mt^ pathway in colonic SMCs plays a critical role in maintaining mitochondrial integrity and regulating gut motility in the FC model.

### 3.6. HSF1 Regulated UPR^mt^ Pathway Expression in Primary Colonic SMCs

**Figure 9:**
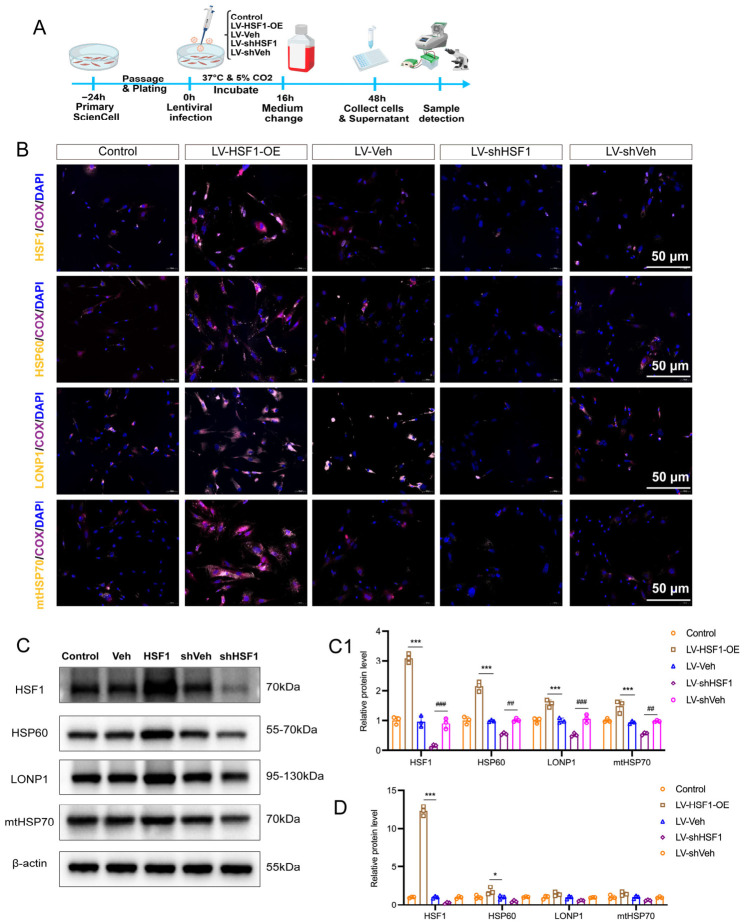
HSF1 regulated UPR^mt^ pathway expression in primary colonic SMCs. (**A**) Schematic diagram of the invitro-experimental design. (**B**) Representative immunofluorescence images of UPR^mt^-related proteins (HSF1, HSP60, mtHSP70, or LONP1, yellow), COX (purple), and DAPI (blue) in the primary SMCs across the five groups: Control, LV-HSF1-OE, LV-Veh, LV-shHSF1, and LV-shVeh. Scale bar, 50 μm, X20. Single-channel images are shown in Figure S8. (**C**) Representative Western blot images of HSF1, HSP60, mtHSP70, LONP1, and β-actin. (**C1**) Quantification of the Western blot band intensities for these proteins, normalized to β-actin (n = 3). (Original Western Blot Images see Supplementary Figure S1.) (**D**) RT-qPCR analysis of HSF1, HSP60, LONP1, and mtHSP70 mRNA levels (n = 3). All data are presented as the mean ± SD, * *p* < 0.05, *** *p* < 0.001 for LV-HSF1-OE vs. LV-veh or ^##^ *p* < 0.01, ^###^ *p* < 0.001 LV-shHSF1 vs. LV-shVeh (one-way ANOVA with Tukey’s post hoc test). LV-HSF1-OE: cells infected with HSF1-overexpressing lentivirus; LV-Veh: cells infected with HSF1-overexpressing empty vector; LV-shHSF1: cells infected with HSF1-targeting shRNA lentivirus; LV-shVeh: cells infected with HSF1-targeting shRNA scrambled control. For detailed statistical information, see Table S10.

To determine whether the regulatory mechanism observed in vivo operates in a cell-autonomous manner, primary mouse colonic SMCs (Procell, CP-M044) were used for in vitro experiments, thereby minimizing potential confounding influences from the in vivo environment. Cells were divided into five experimental groups: a Control group (untransduced cells), and four groups transduced with lentiviral vectors—LV-HSF1-OE, LV-Veh, LV-shHSF1, and LV-shVeh (Figure 9A). Fluorescence microscopy confirmed efficient viral infection in the cultured cells (Figure S8). IF analysis demonstrated that overexpression of HSF1 (LV-HSF1-OE) markedly increased the fluorescence signals of HSF1, HSP60, LONP1, and mtHSP70 compared with the LV-Veh group (Figure 9B). Conversely, knockdown of HSF1 (LV-shHSF1) significantly reduced the fluorescence intensity of these proteins versus the LV-shVeh group (Figure 9B). Western blot analysis further confirmed these observations, showing that protein levels of all four targets were significantly upregulated following LV-HSF1-OE (all *p* < 0.001; Figure 9C) and downregulated following LV-shHSF1 (*p* < 0.001 for HSF1 and LONP1, *p* < 0.01 for HSP60 and mtHSP70; Figure 9C). RT-qPCR analysis confirmed that HSF1 and HSP60 mRNA levels were significantly increased following LV-HSF1-OE (*p* < 0.001 and *p* < 0.05, respectively; Figure 9D), whereas their reduction following LV-shHSF1 did not reach statistical significance at the mRNA level (*p* = 0.08 and *p* = 0.17, respectively; Figure 9D), despite robust protein knockdown; LONP1 and mtHSP70 mRNA levels showed trends consistent with their corresponding protein changes, although these differences did not reach statistical significance (Figure 9D). Overall, these in vitro findings recapitulated the results observed in vivo and indicate that regulation of the UPR^mt^ pathway by HSF1 represents an intrinsic property of colonic SMCs that operates independently of the complex in vivo microenvironment.

### 3.7. HSF1 Regulated Mitochondrial Structure and Function in Primary Colonic SMCs

**Figure 10:**
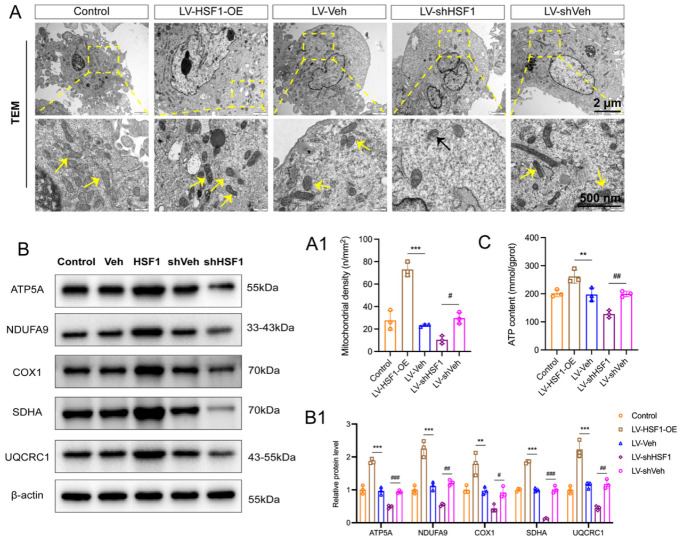
HSF1 regulated mitochondrial structure and function in primary colonic SMCs. (**A**) Representative TEM images of cultured colonic SMCs. Yellow arrows indicate normal mitochondria, and black arrows indicate damaged mitochondria with fragmented cristae. Scale bar, 2 μm (upper panels) and 500 nm (lower panels). (**A1**) Quantitative analysis of mitochondrial density, expressed as the number of mitochondria per mm^2^ of cytoplasmic area (3 fields per cell, n = 3). (**B**) Representative Western blot images of ATP5A, NDUFA9, COX1, SDHA, UQCRC1, and β-actin in cultured colonic SMCs. (**B1**) Quantification of the Western blot band intensities for these proteins, normalized to β-actin (n = 3). (Original Western Blot Images see Supplementary Figure S1.) (**C**) Intracellular ATP levels measured using a colorimetric ATP assay kit, (n = 3). Data are presented as the mean ± SD, ** *p* < 0.01, *** *p* < 0.001 for LV-HSF1-OE vs. LV-veh or ^#^ *p* < 0.05, ^##^ *p* < 0.01, ^###^ *p* < 0.001 LV-shHSF1 vs. LV-shVeh (one-way ANOVA with Tukey’s post hoc test). For the full names of the groups, see the note above. For detailed statistical information, see Table S11.

To further investigate mitochondrial responses downstream of UPR^mt^ modulation, mitochondrial ultrastructure and functional markers were examined in primary colonic SMCs. Transmission electron microscopy revealed that cells transduced with LV-HSF1-OE displayed well-preserved mitochondrial morphology, characterized by intact cristae and increased mitochondrial density per unit area compared with the LV-Veh group (*p* < 0.001; Figure 10A). In contrast, cells subjected to LV-shHSF1 exhibited pronounced mitochondrial vacuolization, disruption of cristae architecture, and a reduction in mitochondrial number (*p* < 0.05; Figure 10A). The functional consequences of these structural alterations were then evaluated. Compared with their respective empty-vector controls, protein levels of mitochondrial respiratory chain complex subunits—including ATP5A (Complex V), NDUFA9 (Complex I), COX1 (Complex IV), SDHA (Complex II), and UQCRC1 (Complex III)—were significantly increased following LV-HSF1-OE (*p* < 0.01 for COX1, *p* < 0.001 for the others; Figure 10B) and decreased following LV-shHSF1 (*p* < 0.001 for ATP5A and SDHA, *p* < 0.01 for NDUFA9 and UQCRC1, *p* < 0.05 for COX1; Figure 10B). Consistent with these molecular changes, intracellular ATP levels were significantly elevated in the LV-HSF1-OE group and reduced in the LV-shHSF1 group compared with their respective controls (both *p* < 0.01; Figure 10C). These in vitro findings closely mirrored the observations obtained from the in vivo mouse experiments. Taken together, the results demonstrate that colonic SMCs possess an intrinsic regulatory mechanism in which the HSF1-UPR^mt^ pathway controls mitochondrial structure and energy metabolism. Disruption of this cell-autonomous pathway therefore represents a key mechanistic link contributing to the gut motility deficits observed in FC.

## 4. Discussion

**Figure 11:**
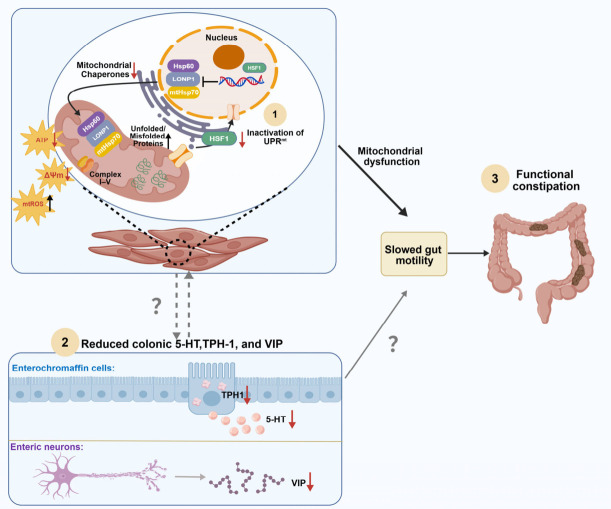
Schematic summary of HSF1-mediated UPR^mt^ regulation in colonic SMCs during FC. ① In FC, downregulation of HSF1 in colonic SMCs suppresses the expression of UPR^mt^-related proteins, including HSP60, mtHSP70, and LONP1. This leads to mitochondrial structural damage, reduced mitochondrial number, increased mtROS production, decreased mitochondrial membrane potential, diminished ATP synthesis, and impaired respiratory chain activity. ② Colonic levels of 5-HT, TPH-1, and VIP are reduced in FC. 5-HT and TPH-1 are produced by enterochromaffin cells, while VIP is produced by enteric neurons, not by colonic SMCs. The relationship between SMC mitochondrial dysfunction and these neurohumoral changes, and the extent to which these changes contribute to constipation, remain to be fully elucidated (indicated by dashed arrows and question marks). ③ These changes together ultimately lead to slowed gut motility and functional constipation (This figure was created with BioGDP.com). HSP60: heat shock protein 60; mtHSP70: mitochondrial heat shock protein 70; LONP1: Lon peptidase 1; mtROS: mitochondrial reactive oxygen species; 5-HT: 5-hydroxytryptamine; TPH-1: tryptophan hydroxylase 1; VIP: vasoactive intestinal peptide.

This study demonstrates that the UPR^mt^ in colonic SMCs is not merely a passive stress-protection program but rather a determinant of gut motility outcomes. In the loperamide-induced FC mouse model, HSF1 expression in colonic SMCs was markedly downregulated, leading to suppression of the UPR^mt^. This dysregulation correlated with mitochondrial structural damage, disrupted energy metabolism, and worsening of the gut motility phenotype. Bidirectional manipulation of HSF1—via in vivo AAV9 or in vitro LV interventions—either restored or further impaired UPR^mt^ activity, mitochondrial function, and motility performance. Importantly, this regulatory mechanism operated in a cell-autonomous manner in primary colonic SMCs. Together, these findings indicate that the HSF1–UPR^mt^ signaling axis is critical for maintaining mitochondrial homeostasis in colonic SMCs and for preserving normal gut motility, and that its dysfunction constitutes an intrinsic mechanism underlying motility disorder in FC (Figure 11).

Mitochondrial dysfunction in FC was documented from multiple perspectives, including oxidative damage, disturbances in energy metabolism, and mtDNA mutations [43,44,45]. However, less attention focused on whether the mitochondrial proteostasis machinery itself was compromised in this context. The UPR^mt^ serves as the primary transcriptional program that maintains mitochondrial proteostasis by inducing chaperones and proteases to refold or degrade misfolded proteins [23,25,46]. In contrast to mitophagy, which eliminates damaged mitochondria, the UPR^mt^ repairs mitochondrial proteostasis by upregulating chaperones and proteases [47]. This distinction is particularly relevant in FC, where the functional disturbances are considered reversible rather than degenerative. Sun et al. reported that mitochondrial dysfunction and enteric neuropathy underlie the pathogenesis of functional constipation, and that restoring mitochondrial membrane fusion via a targeted nanoplatform significantly improved colonic motility in preclinical models [48]. Additionally, rotenone, a complex I inhibitor that activated the UPR^mt^, improved constipation symptoms [49]. Our single-cell transcriptomic data extend these observations by showing consistent downregulation of UPR^mt^ genes in colonic SMCs of FC mice. This finding places UPR^mt^ dysfunction not only in epithelial and stem cell compartments, as previously studied, but also in the smooth muscle layer, directly linking it to the gut’s motility apparatus.

Recent studies have established that the UPR^mt^ plays a critical role in preserving intestinal homeostasis, although they examined different cell types and stressors [50,51]. Berger et al. [52] used an intestinal epithelial-specific HSP60 knockout and showed that UPR^mt^ activation caused epithelial stem cell loss and proliferation arrest; paradoxically, it also induced compensatory overproliferation of escaper cells via paracrine WNT signaling, indicating that mitochondrial stress can non-cell-autonomously reshape epithelial regeneration. Yang et al. [53] corroborated and extended these findings in a POLG mutant mouse: an increased mtDNA mutation load depleted NAD^+^, activated ATF5-dependent UPR^mt^, suppressed Wnt/β-catenin signaling, and precipitated exhaustion of Lgr5^+^ intestinal stem cells and premature intestinal aging. Together, these studies placed the UPR^mt^ at the center of epithelial and stem cell regulation in the intestine, but they left open whether the UPR^mt^ influences SMCs and gut motility. The present study addresses that gap: in the FC state, the UPR^mt^ in colonic SMCs is repressed, and this repression does not result from HSP60 deficiency or excessive mtDNA mutations but is directly associated with downregulation of HSF1 expression. Unlike the compensatory proliferation linked to UPR^mt^ activation reported by Berger et al. [52], the suppression of the UPR^mt^ in our study is closely associated with disrupted energy metabolism and impaired gut motility, and these mitochondrial deficits are likely to contribute to reduced contractile performance of SMCs in the FC state, indicating that the same pathway can produce markedly different pathological outcomes across cell types. Consistent with Yang et al. [53], our data also show a positive correlation between UPR^mt^ activity and cellular functional status; however, we targeted the transcription factor HSF1 rather than NAD^+^, thereby identifying a new molecular entry point for UPR^mt^ regulation. Hao et al. reported that 4,6-diamino-2-pyrimidinethiol-modified gold nanoparticles improved intestinal mitochondrial function and relieved constipation via AMPK/PGC-1α activation [54]. That study demonstrated the therapeutic value of enhancing mitochondrial metabolism through exogenous pharmacological intervention, whereas our work reveals that the HSF1–UPR^mt^ axis acts as a key downstream mechanism mediating those effects through endogenous transcriptional regulation. In sum, the present study extends UPR^mt^ research beyond epithelial homeostasis and aging into smooth muscle dynamics and identifies HSF1 as a novel molecular node linking mitochondrial quality control to gut motility.

HSF1, a canonical heat shock transcription factor, has recently been shown to contribute to the mitochondrial stress response and to directly activate multiple UPR^mt^ target genes, including HSP60 [55,56]. In the present study, we employed HSF1 as a mechanistic probe and used both gain- and loss-of-function approaches to define its upstream regulatory role in the UPR^mt^ pathway in colonic SMCs. In vivo overexpression of HSF1 restored expression of UPR^mt^ components, improved mitochondrial morphology and function, and ameliorated the constipation phenotype. By contrast, inhibition of HSF1 further suppressed the pathway and aggravated mitochondrial damage. Primary cell experiments further verified this bidirectional regulatory relationship and established that the HSF1–UPR^mt^ axis operates in a cell-autonomous manner in SMCs. We do not claim, however, that HSF1 is the sole initiating factor in FC; rather, we consider it a regulatory node that highlights the central role of the UPR^mt^ in FC pathophysiology. Within the complex etiological network of FC [57], multiple upstream factors may converge on mitochondrial stress and, by altering HSF1 expression or transcriptional activity, diminish the repair capacity of the UPR^mt^ pathway and thereby provoke energy metabolic disturbances in SMCs. Our present study is the first to demonstrate, through bidirectional genetic manipulation, a direct link between functional inhibition of the HSF1–UPR^mt^ axis and reduced intestinal motility, providing new evidence supporting an energy-metabolic origin for FC.

The mammalian UPR^mt^ was not controlled by a single transcription factor. The most well-characterized branch involved ATF5, and in some contexts, ATF4/CHOP, which bound to MUREs and drove a broad transcriptional response that included chaperones, proteases, and metabolic enzymes [21,22]. HSF1 regulated a narrower set of targets, most notably HSP60 and mtHSP70, through a distinct mechanism by binding to HSEs in their promoters [25,26]. These two branches were likely not redundant. The ATF5 branch coordinated a wide stress response, while HSF1 maintained the basal and stress-induced expression of key mitochondrial chaperones [27]. In our scRNA-seq data, HSF1 downregulated significantly in colonic SMCs of FC mice, while ATF5 exhibited no notable change. This pattern indicated a selective loss of the HSF1 branch in this disease context. Its loss alone, even with intact ATF5 signaling still compromised mitochondrial proteostasis. The potential for the two branches to compensate for one another, and the conditions under which this might occur, remained an open question. Future research should investigate whether HSF1 directly binds to HSEs in the HSP60 and mtHSP70 promoters in colonic SMCs, for instance, through ChIP-qPCR. Additionally, it should assess whether HSF1 functionally cooperates with ATF5 or other signaling intermediates, such as the SIRT3–PGC-1α axis, in this tissue.

In the present study, colonic tissue from FC mice exhibited reduced mitochondrial membrane potential, increased mtROS production, decreased ATP levels, and diminished activities of respiratory chain complexes I through V. The changes in all measured functional parameters were consistent. Overexpression of HSF1 led to improvements in these indicators, while knockdown of HSF1 resulted in further deterioration. One interpretation suggested that HSF1-dependent UPR^mt^ activity sustained a baseline of mitochondrial function in colonic SMCs. The loss of this baseline created a bioenergetic state inadequate for normal contractile activity. The JC-1 and MitoSOX data originated from whole-tissue single-cell suspensions, reflecting tissue-level mitochondrial function rather than specific measurements from SMCs. Nonetheless, TEM revealed a similar pattern of reduced membrane potential and increased ROS within SMCs, identified by their ultrastructure. Additionally, IF demonstrated co-localization of COX IV with α-SMA in the muscularis propria. We note that α-SMA is not exclusive to SMCs; however, restricting quantification to the muscularis propria provides positional specificity. The consistency across these methods suggests that the flow cytometry results were not predominantly influenced by non-SMC cell types. Future studies should incorporate SMC-specific surface marker gating or genetic labeling strategies to enable direct isolation of colonic SMCs for functional profiling, yielding a more precise readout.

An unresolved question from our data pertained to the link between SMC mitochondrial dysfunction and the neurohumoral alterations in the FC colon. While 5-HT is mainly produced by enterochromaffin cells and VIP is primarily released by inhibitory enteric motor neurons, neither of these neuroactive substances originates directly from SMCs. The diminished levels of these molecules in the FC condition might result from the direct effects of loperamide on μ-opioid receptors in enteroendocrine and neuronal cells, rather than being a consequence of SMC mitochondrial dysfunction. Another possibility is that reduced SMC contractility could indirectly impact enteroendocrine and enteric neuronal function through mechanosensory feedback pathways, including those involving Piezo channels or other mechanotransducers in the intestinal wall [31,58]. Future studies that utilize enterochromaffin cell-specific or enteric neuron-specific genetic manipulations will be necessary to distinguish between these possibilities. Understanding this interplay represents a crucial area for future research, as it may determine whether mitochondrial-targeted therapies can indirectly restore neurohumoral balance and provide additional prokinetic effects in FC, beyond merely improving SMC contractility. The question of whether the HSF1–UPR^mt^ axis functions similarly in female animals remains unanswered. FC is significantly more prevalent in women than in men [2], and sex-dependent differences in mitochondrial biology are well documented [59]. The current study involved only male mice, leaving it unclear whether HSF1-mediated regulation of mitochondrial proteostasis in colonic SMCs varies by sex. Investigating this question could clarify whether the female predominance of FC has a mitochondrial basis.

These unresolved questions also point to several boundaries of what the present study can conclude. First, although the loperamide-induced constipation model is widely used to mimic FC, it cannot fully recapitulate the complex etiology of human FC. The conclusions therefore require validation in additional etiological models, such as those involving low-fiber diet, aging, or genetic modifications. Second, the JC-1 and MitoSOX flow cytometry assays were performed on whole-tissue single-cell suspensions without SMC-specific surface marker gating, and therefore reflect tissue-level rather than SMC-specific mitochondrial function. Incorporating SMC-specific surface markers or genetic labeling strategies in future studies would allow direct isolation of colonic SMCs for functional mitochondrial assays. Third, the bidirectional modulation experiments do not establish whether HSF1 downregulation precedes or follows the onset of FC. It remains possible that reduced contractile activity leads to secondary mitochondrial changes. Resolving the causal direction will require both longitudinal tracking of HSF1 expression during FC development and SMC-specific HSF1 deletion prior to FC induction.

Nevertheless, this study proposes that interventions restoring UPR^mt^ function could represent a new therapeutic direction for FC. Currently, first-line therapies for FC (such as polyethylene glycol [60] and prucalopride [61]) primarily target intestinal secretion or neurotransmitter receptors. Although these agents improve defecation frequency in the short term, they rarely reverse intrinsic contraction dysfunction of SMCs [62], and long-term use commonly leads to diminished efficacy and tolerance [63]. By contrast, moderate activation of HSF1 or enhancement of the UPR^mt^ pathway repairs the energetic basis of the contractile apparatus at the level of cellular energy metabolism, rather than merely replacing or stimulating residual function. Targeting mitochondrial quality control thereby addresses the metabolic root of smooth muscle motility disorders and has the potential to reverse cellular functional decline, representing a more fundamental therapeutic concept.

## 5. Conclusions

This study combines complementary in vivo and in vitro approaches to investigate the role of the HSF1-UPR^mt^ pathway in the pathogenesis of FC. Our findings show that HSF1 expression significantly decreases in colonic SMCs of FC mice, and this reduction correlates with suppression of the UPR^mt^ pathway. The loss of HSF1 activity closely associates with mitochondrial structural damage, impaired mitochondrial function, and reduced gut motility. Restoring HSF1 activity reactivates the UPR^mt^ pathway, preserves mitochondrial homeostasis, and improves gut motility in a cell-autonomous manner. Collectively, these results identify HSF1 as a critical regulator of gut motility in FC through its regulation of the UPR^mt^ pathway. It remains to be determined whether HSF1 downregulation is an initiating event or a secondary response to reduced contractile activity, and further validation in additional etiological models and human tissue samples is necessary. Nonetheless, these findings suggest that therapeutic strategies targeting the HSF1–UPR^mt^ axis may offer a promising approach for treating FC.

## Data Availability

The data supporting the findings of this study are available within the article and the Supplementary Materials. The original uncropped Western blot images are available at Figshare (https://doi.org/10.6084/m9.figshare.32287419). The single-cell RNA sequencing data generated and analyzed during this study are available in the NCBI Gene Expression Omnibus repository (http://www.ncbi.nlm.nih.gov/bioproject/1441204, accessed on 31 December 2025). The accession number for the dataset is PRJNA1441204.

## Supplementary Materials

The following supporting information can be downloaded at: https://www.mdpi.com/article/10.3390/biom16060868/s1, Figure S1. The full, uncropped Western blot images. Figure S2. Violin plots showing expression levels of specific markers across all identified cell types. Figure S3. Representative IF images of individual staining channels for UPR^mt^-related proteins and α-SMA in the control and FC mice. Figure S4. Representative IF images of individual staining channels for COX IV in the control and FC mice. Figure S5. The confirmation of successful AAV9-mediated HSF1 modulation in colonic tissue from FC mice. Figure S6. Representative IF images of individual staining channels for UPR^mt^-related proteins in the AAV9-treated groups. Figure S7. Representative IF images of individual staining channels for COX IV and α-SMA in the AAV9-treated groups. Figure S8. Lentiviral transfection efficiency images in primary SMCs. Figure S9. Representative IF images of individual staining channels for UPR^mt^-related proteins in the LV-transfected primary SMCs. Table S1. AAV9 vectors used in this study. Table S2. List of primary and secondary antibodies. Table S3. PCR primers. Tables S4–S11. Detailed statistical information corresponding to Figure 2, Figure 3, Figure 4, Figure 5, Figure 6, Figure 7, Figure 8, Figure 9 and Figure 10.

## Abbreviations

FC, functional constipation; SMCs, smooth muscle cells; ATP, adenosine triphosphate; ROS, reactive oxygen species; mtROS, mitochondrial reactive oxygen species; UPR^mt^, mitochondrial unfolded protein response; HSP60, heat shock protein 60; mtHSP70, mitochondrial heat shock protein 70; LONP1, lon peptidase 1; HSF1, heat shock factor 1; ATF5, activating transcription factor 5; ATF4, activating transcription factor 4; AAV, adeno-associated virus; LV, lentivirus; shRNA, short hairpin RNA; GFP, green fluorescent protein; Sm22a, smooth muscle 22 alpha; TGITT, total gastrointestinal transit time; 5-HT, 5-hydroxytryptamine; TPH1, tryptophan hydroxylase 1; VIP, vasoactive intestinal peptide; MitoSOX, mitochondria-targeted superoxide indicator; IF, immunofluorescence; TEM, transmission electron microscopy; WB, Western blotting; RT-qPCR, reverse transcription quantitative polymerase chain reaction; α-SMA, alpha-smooth muscle actin; COX IV, cytochrome c oxidase subunit IV; HSE, heat shock element; MURE, mitochondrial unfolded protein response element; DPBS, Dulbecco’s phosphate-buffered saline; FBS, fetal bovine serum; BCA, bicinchoninic acid; UMAP, uniform manifold approximation and projection; scRNA-seq, single-cell RNA sequencing; SD, standard deviation; ANOVA, analysis of variance.
