# Supplementary figures and images for: Dysregulation of the HSF1-Mediated UPRmt Pathway in Colonic Smooth Muscle Cells Drives Motility Dysfunction in Functional Constipation (part 2 of 2)
Source: Biomolecules. 2026 Jun 12;16(6):868. doi: 10.3390/biom16060868 (PMC13296460; doi:10.3390/biom16060868)

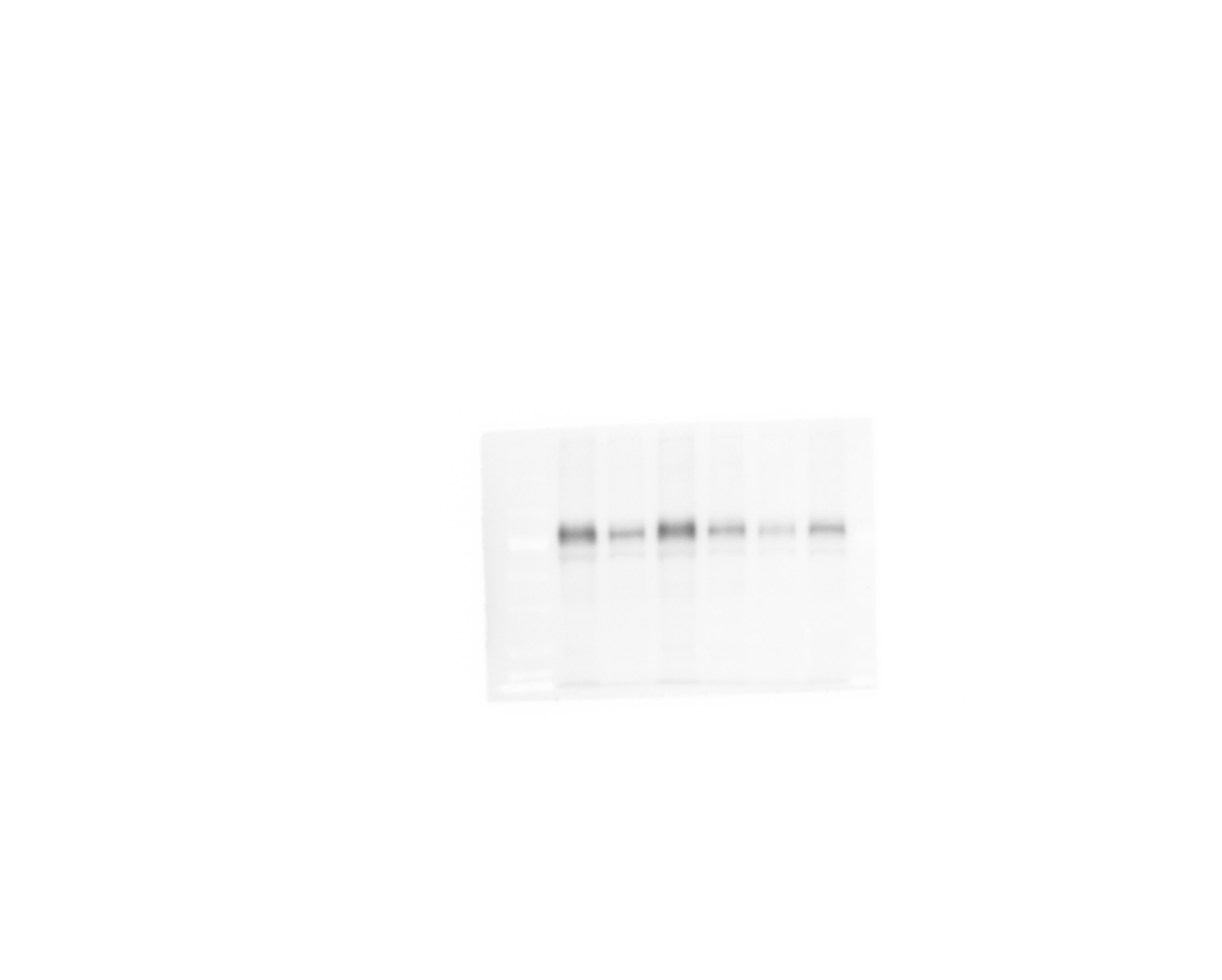

Supplement: Supplementary file 1 [file biomolecules-16-00868-s001.zip › FigureS1 the full, uncropped western blot images/The vivo mice study/HSF1/3-0.4s.Tif]

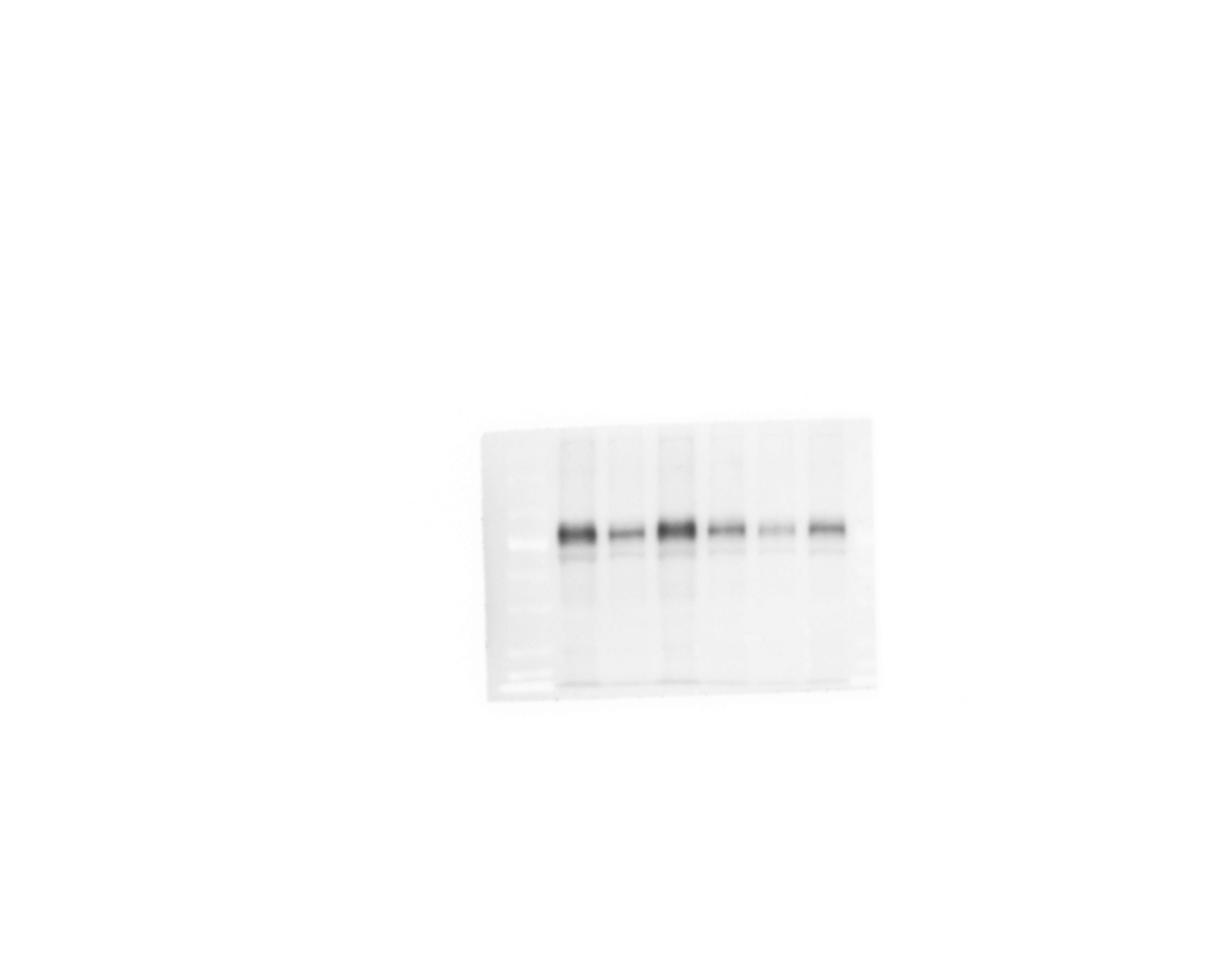

Supplement: Supplementary file 1 [file biomolecules-16-00868-s001.zip › FigureS1 the full, uncropped western blot images/The vivo mice study/HSF1/3-2s.Tif]

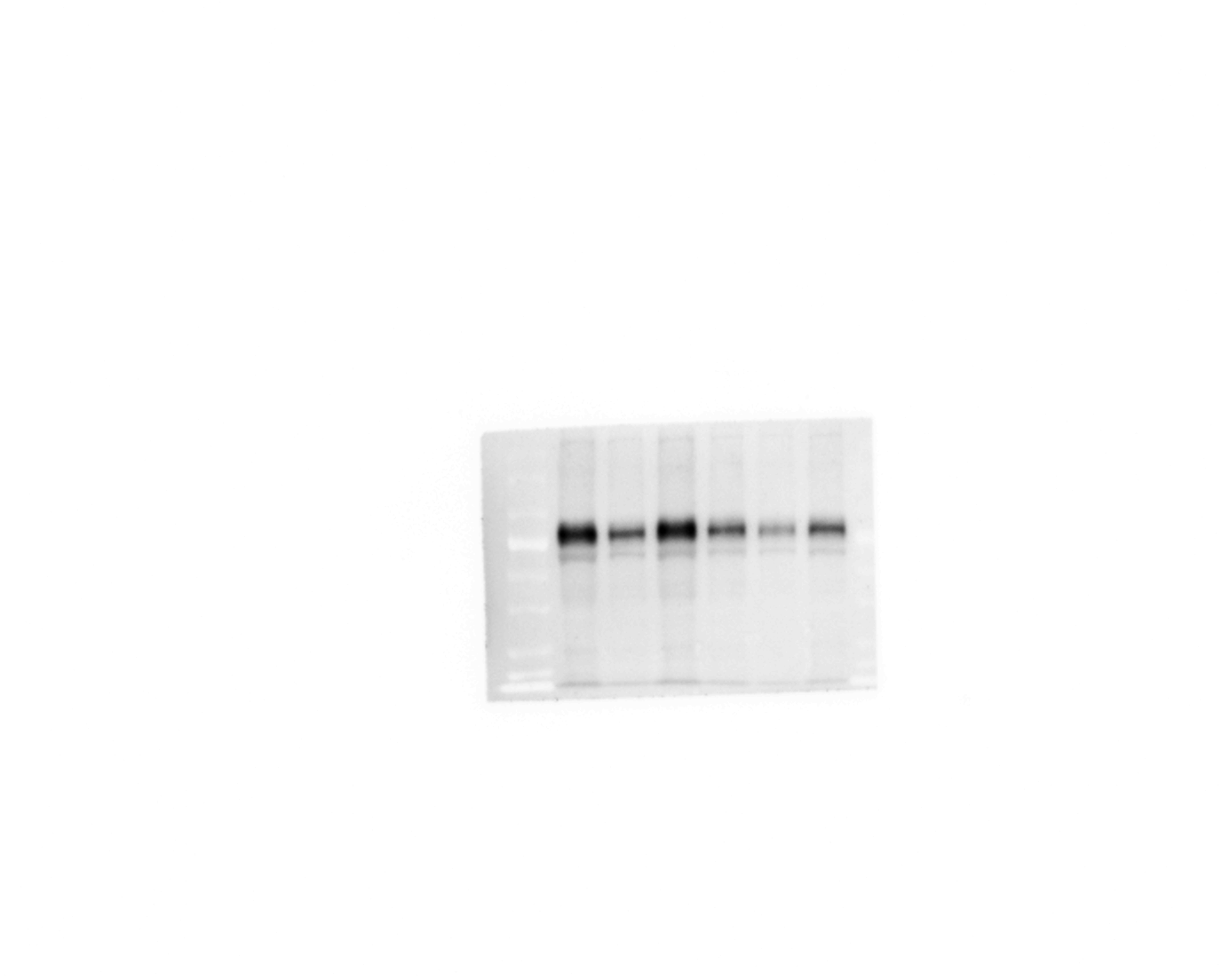

Supplement: Supplementary file 1 [file biomolecules-16-00868-s001.zip › FigureS1 the full, uncropped western blot images/The vivo mice study/HSF1/3-5s.Tif]

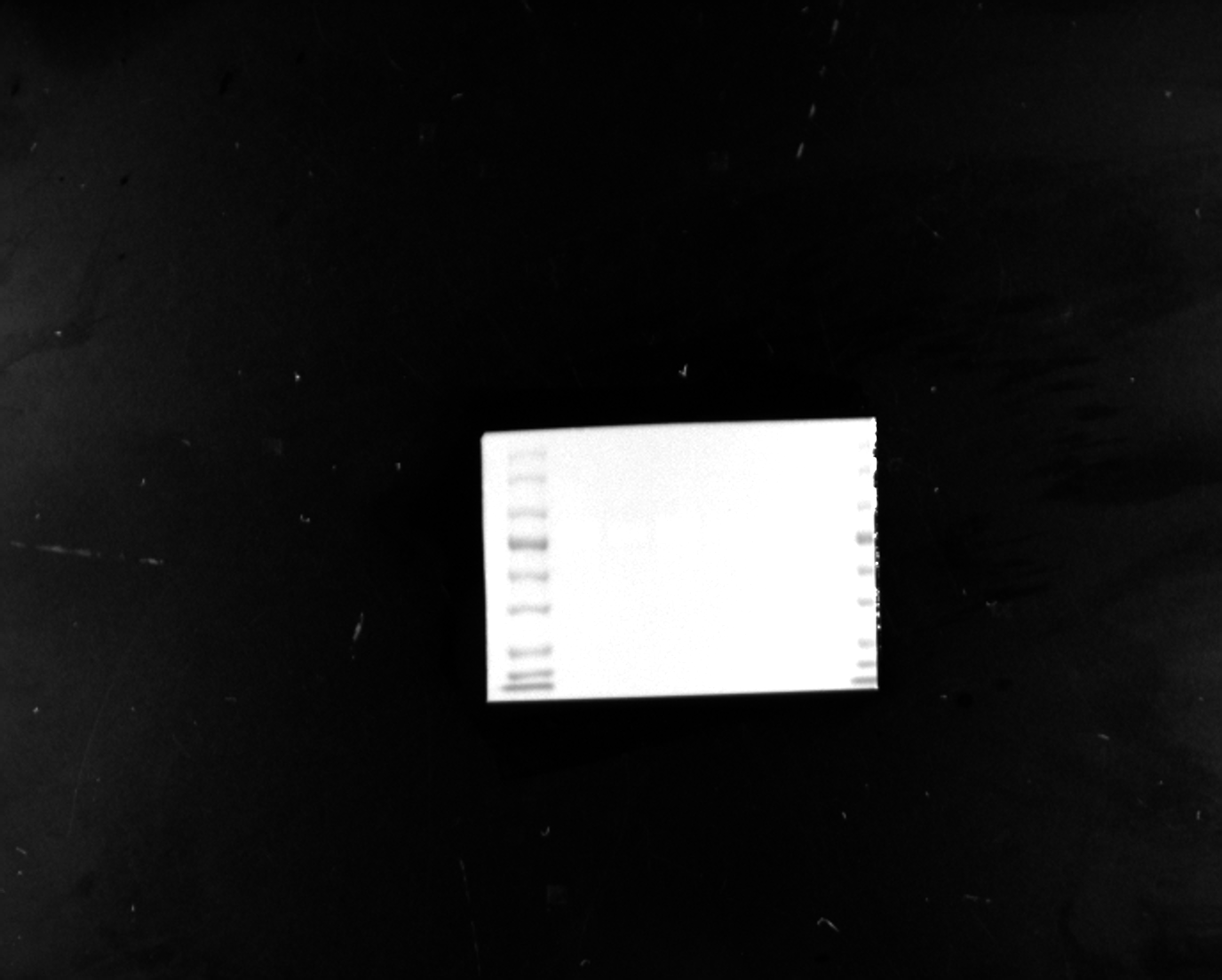

Supplement: Supplementary file 1 [file biomolecules-16-00868-s001.zip › FigureS1 the full, uncropped western blot images/The vivo mice study/HSF1/3-t.Tif]

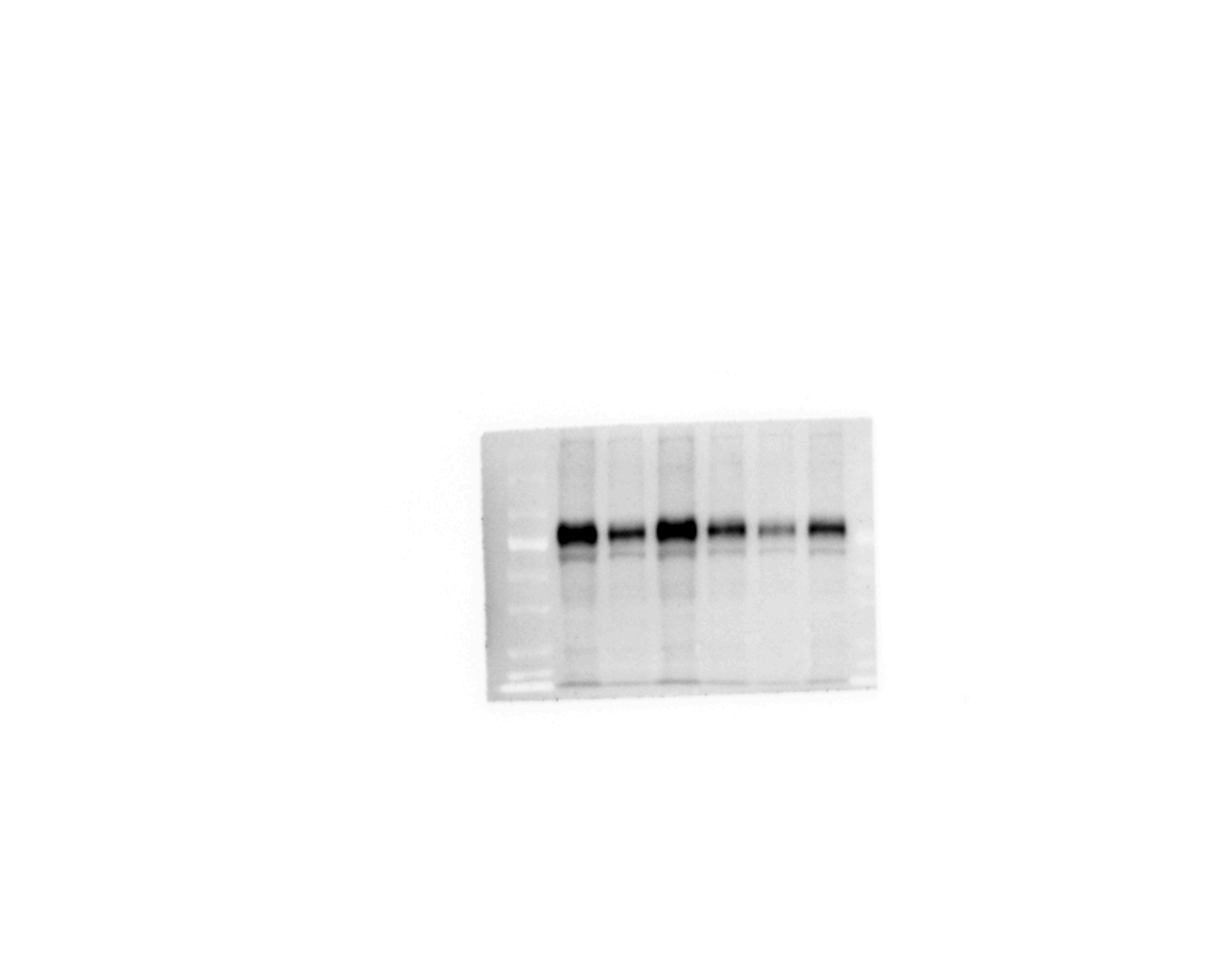

Supplement: Supplementary file 1 [file biomolecules-16-00868-s001.zip › FigureS1 the full, uncropped western blot images/The vivo mice study/HSF1/3.Tif]

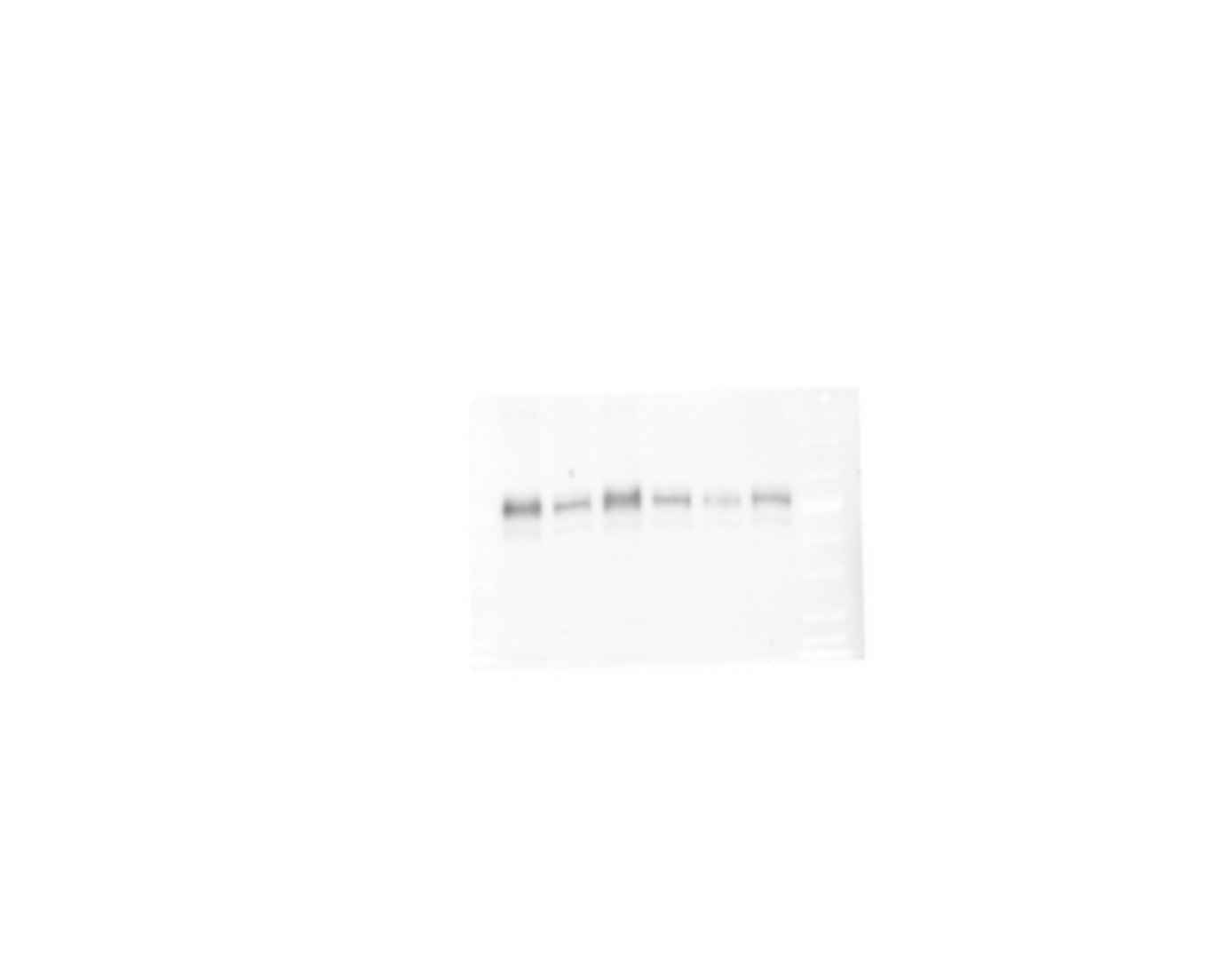

Supplement: Supplementary file 1 [file biomolecules-16-00868-s001.zip › FigureS1 the full, uncropped western blot images/The vivo mice study/HSF1/4-0.6s.Tif]

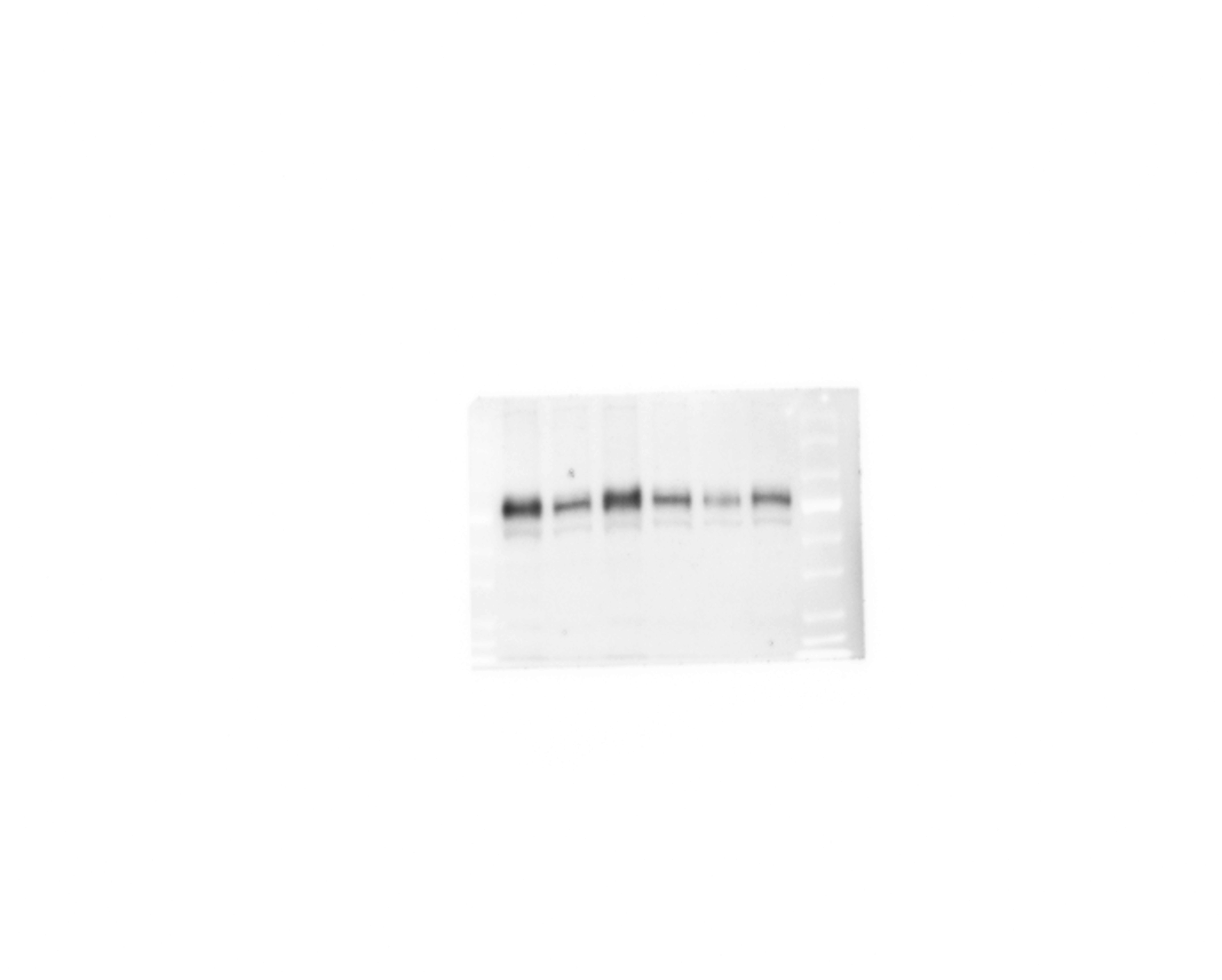

Supplement: Supplementary file 1 [file biomolecules-16-00868-s001.zip › FigureS1 the full, uncropped western blot images/The vivo mice study/HSF1/4-2s.Tif]

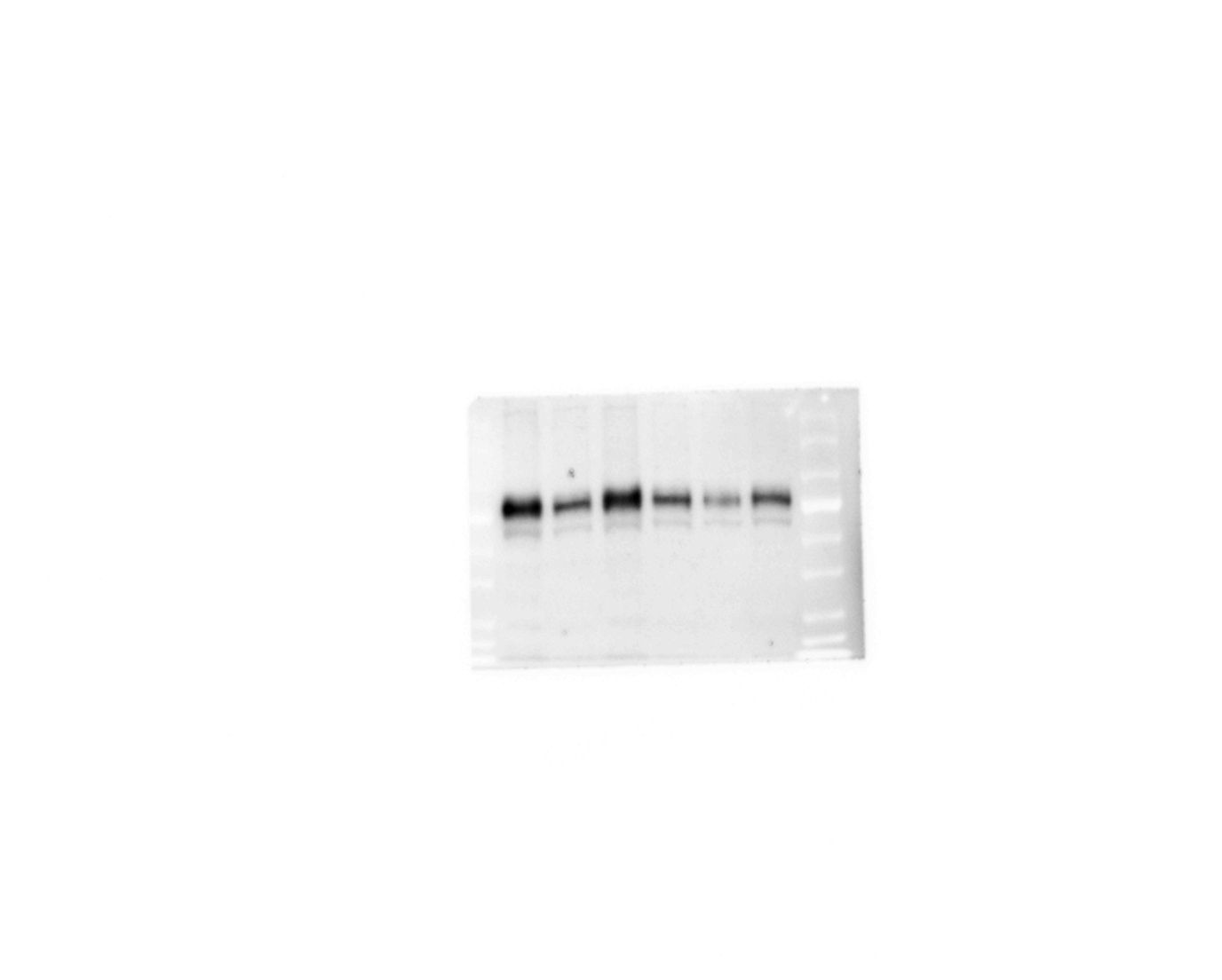

Supplement: Supplementary file 1 [file biomolecules-16-00868-s001.zip › FigureS1 the full, uncropped western blot images/The vivo mice study/HSF1/4-5s.Tif]

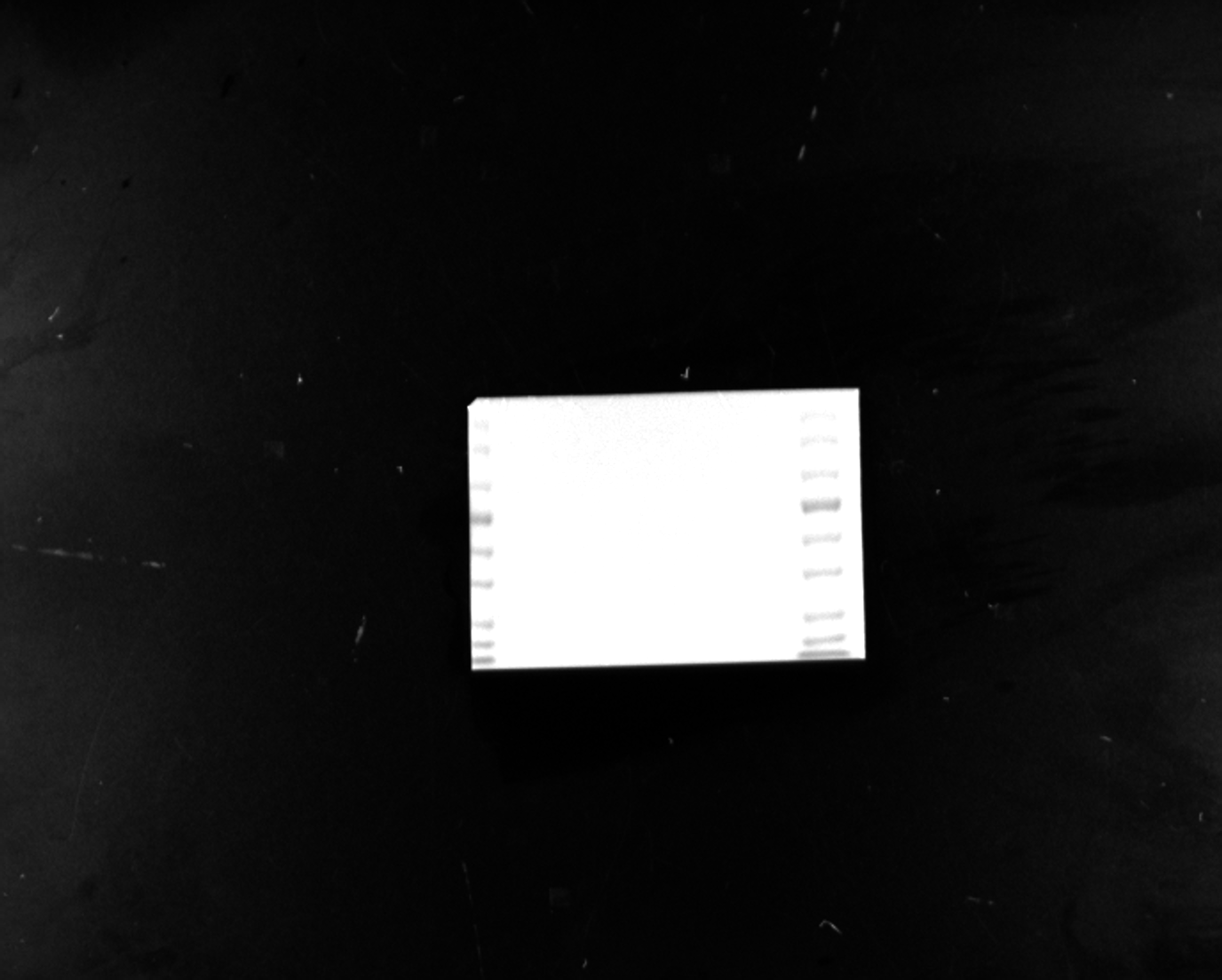

Supplement: Supplementary file 1 [file biomolecules-16-00868-s001.zip › FigureS1 the full, uncropped western blot images/The vivo mice study/HSF1/4-t.Tif]

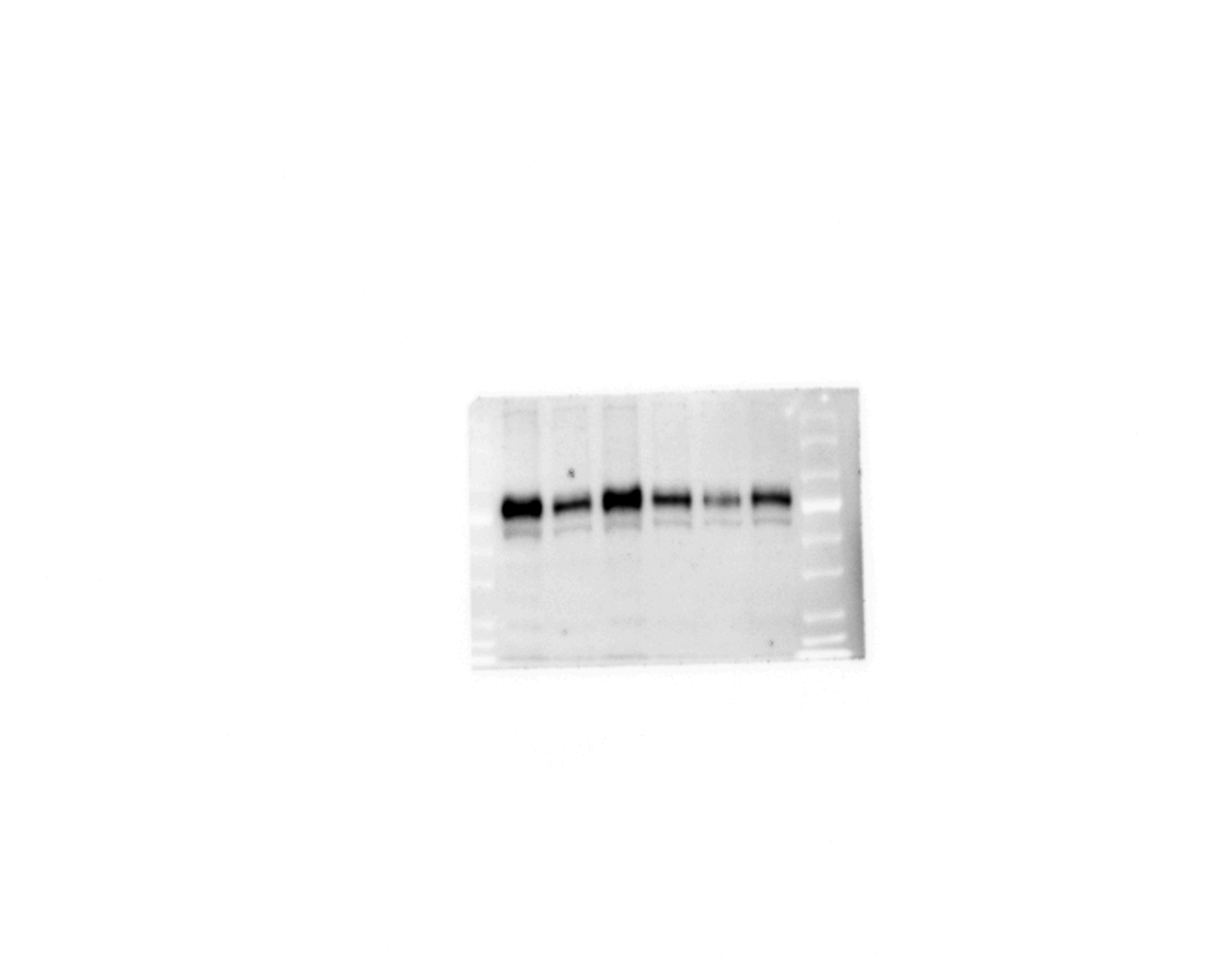

Supplement: Supplementary file 1 [file biomolecules-16-00868-s001.zip › FigureS1 the full, uncropped western blot images/The vivo mice study/HSF1/4.Tif]

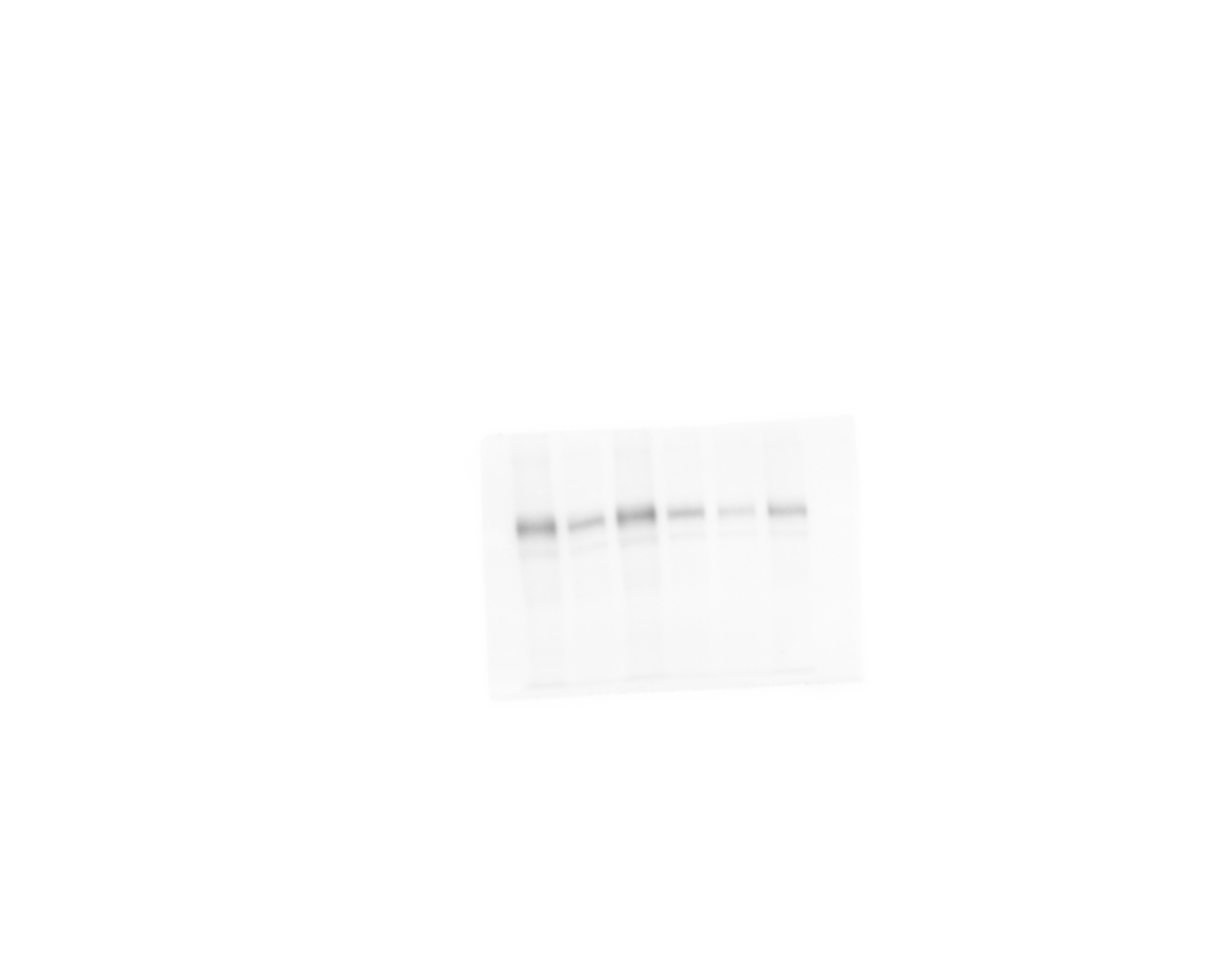

Supplement: Supplementary file 1 [file biomolecules-16-00868-s001.zip › FigureS1 the full, uncropped western blot images/The vivo mice study/HSF1/5-0.5s.Tif]

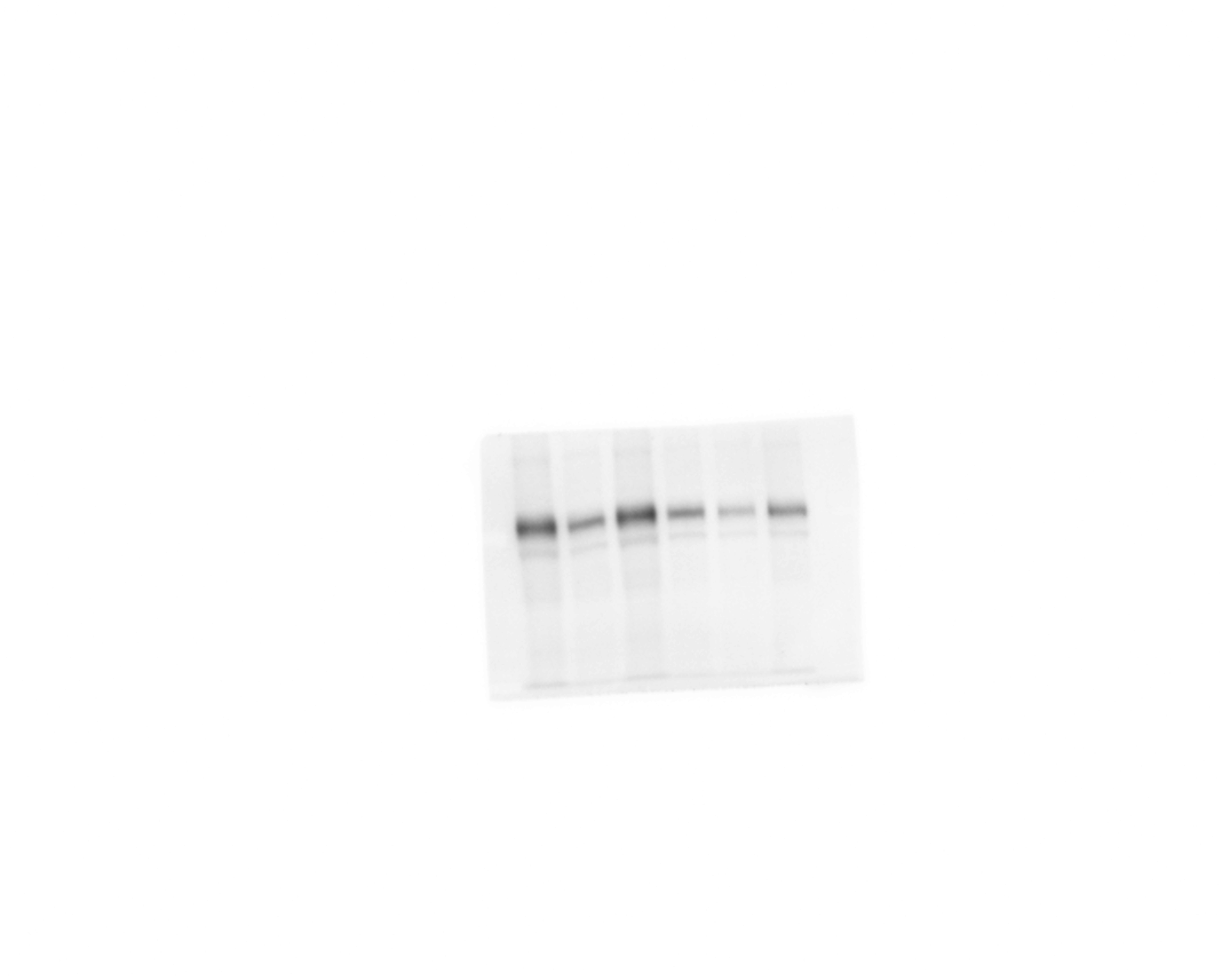

Supplement: Supplementary file 1 [file biomolecules-16-00868-s001.zip › FigureS1 the full, uncropped western blot images/The vivo mice study/HSF1/5-3s.Tif]

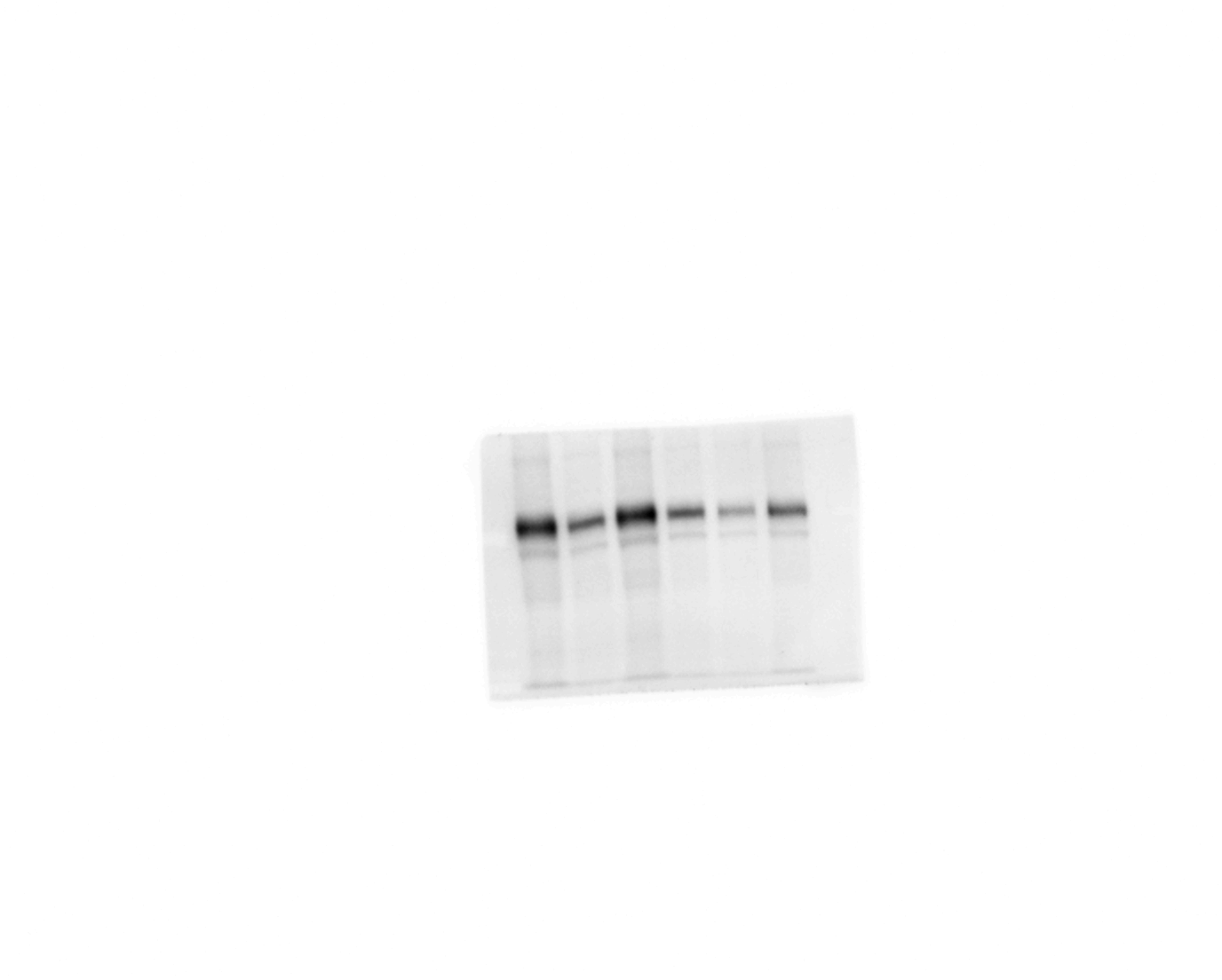

Supplement: Supplementary file 1 [file biomolecules-16-00868-s001.zip › FigureS1 the full, uncropped western blot images/The vivo mice study/HSF1/5-6s.Tif]

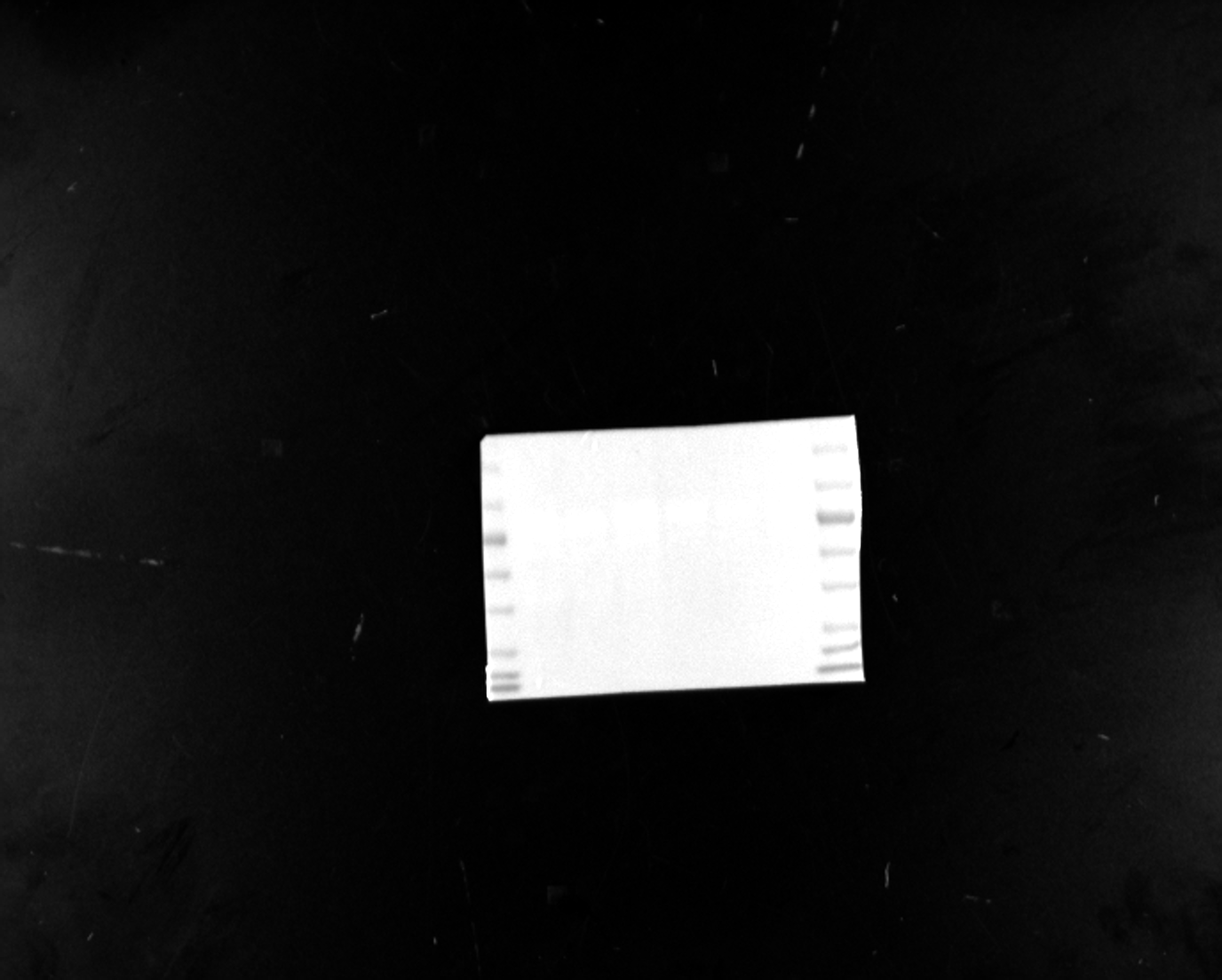

Supplement: Supplementary file 1 [file biomolecules-16-00868-s001.zip › FigureS1 the full, uncropped western blot images/The vivo mice study/HSF1/5-t.Tif]

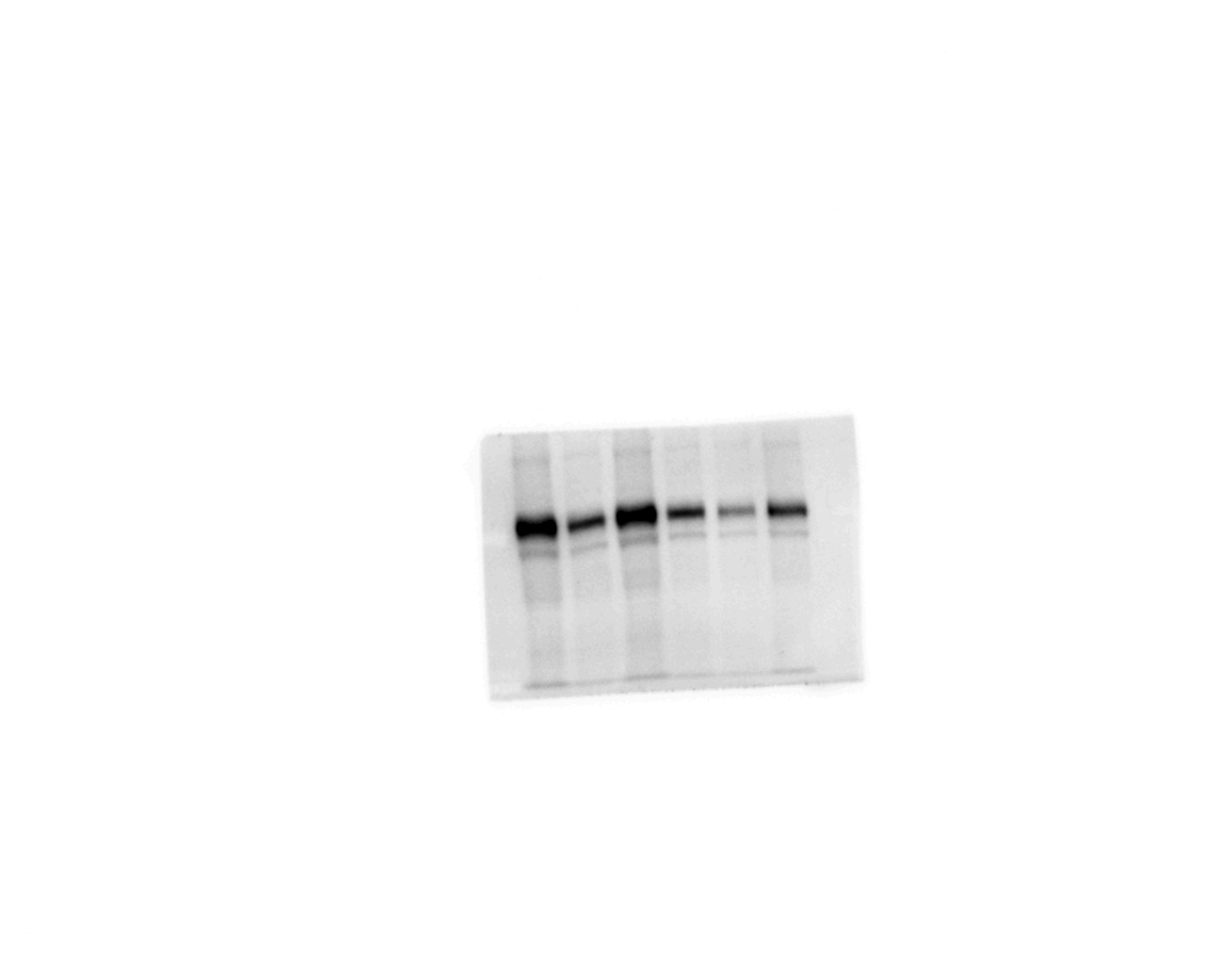

Supplement: Supplementary file 1 [file biomolecules-16-00868-s001.zip › FigureS1 the full, uncropped western blot images/The vivo mice study/HSF1/5.Tif]

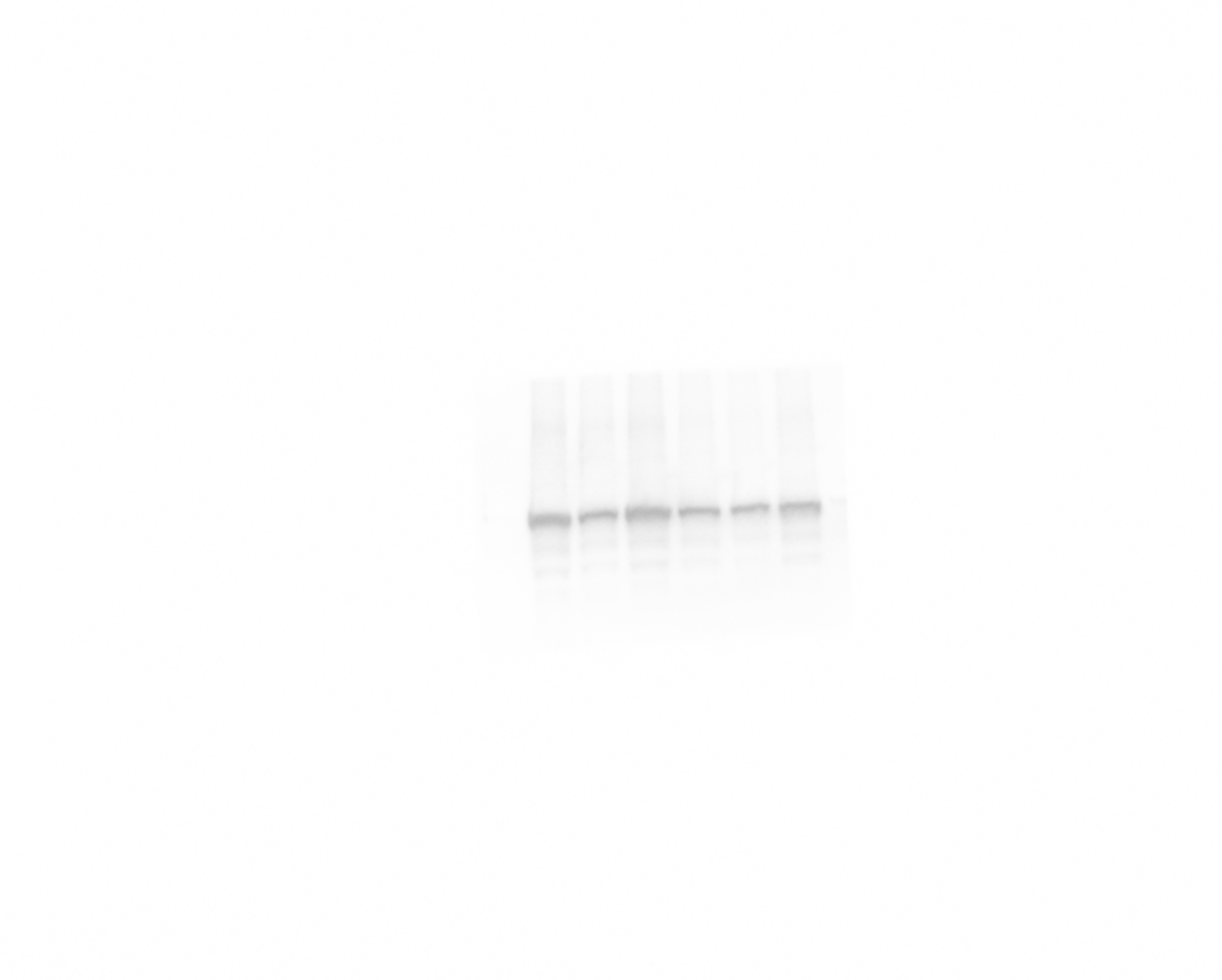

Supplement: Supplementary file 1 [file biomolecules-16-00868-s001.zip › FigureS1 the full, uncropped western blot images/The vivo mice study/HSP60/1-0.5s.Tif]

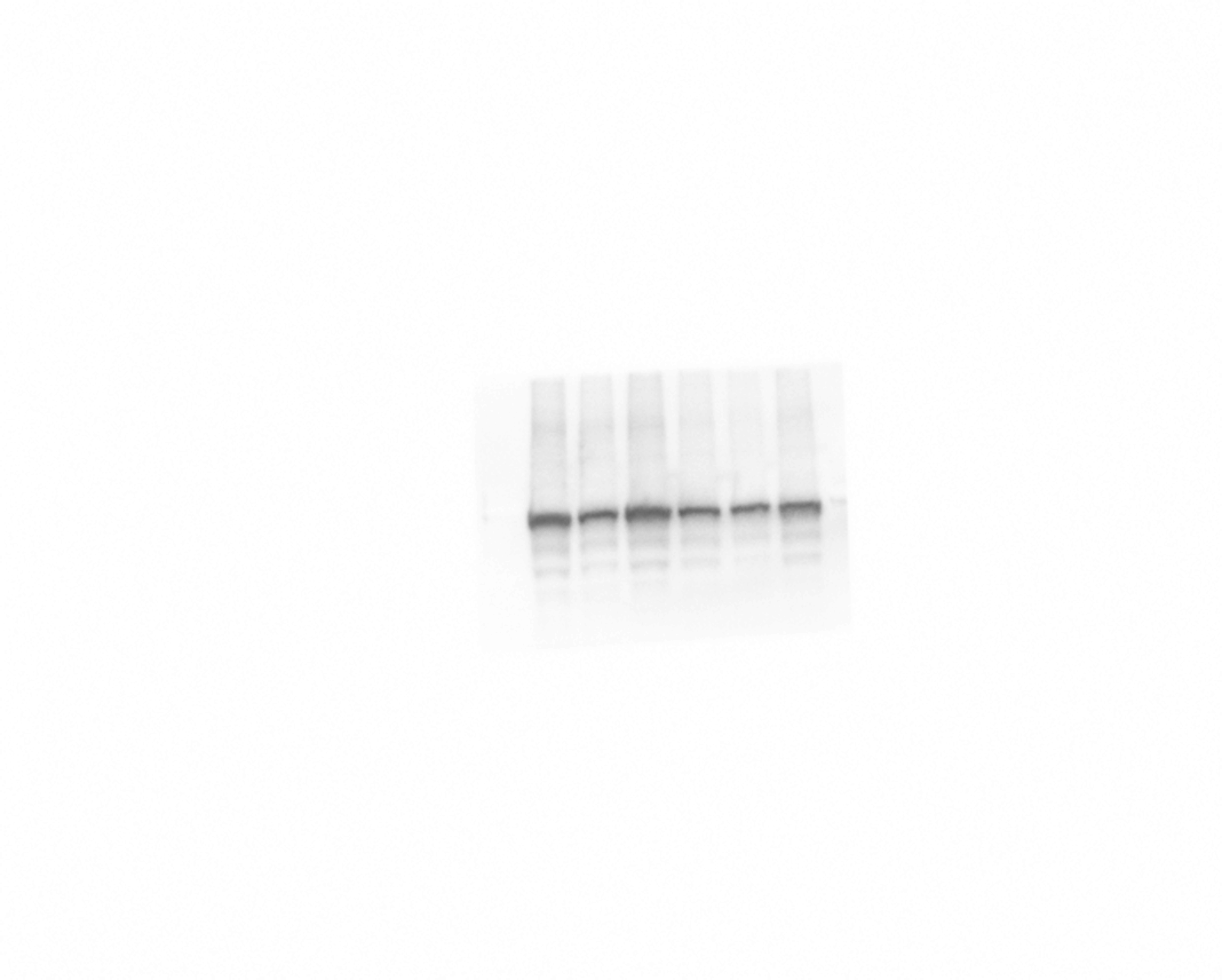

Supplement: Supplementary file 1 [file biomolecules-16-00868-s001.zip › FigureS1 the full, uncropped western blot images/The vivo mice study/HSP60/1-2s.Tif]

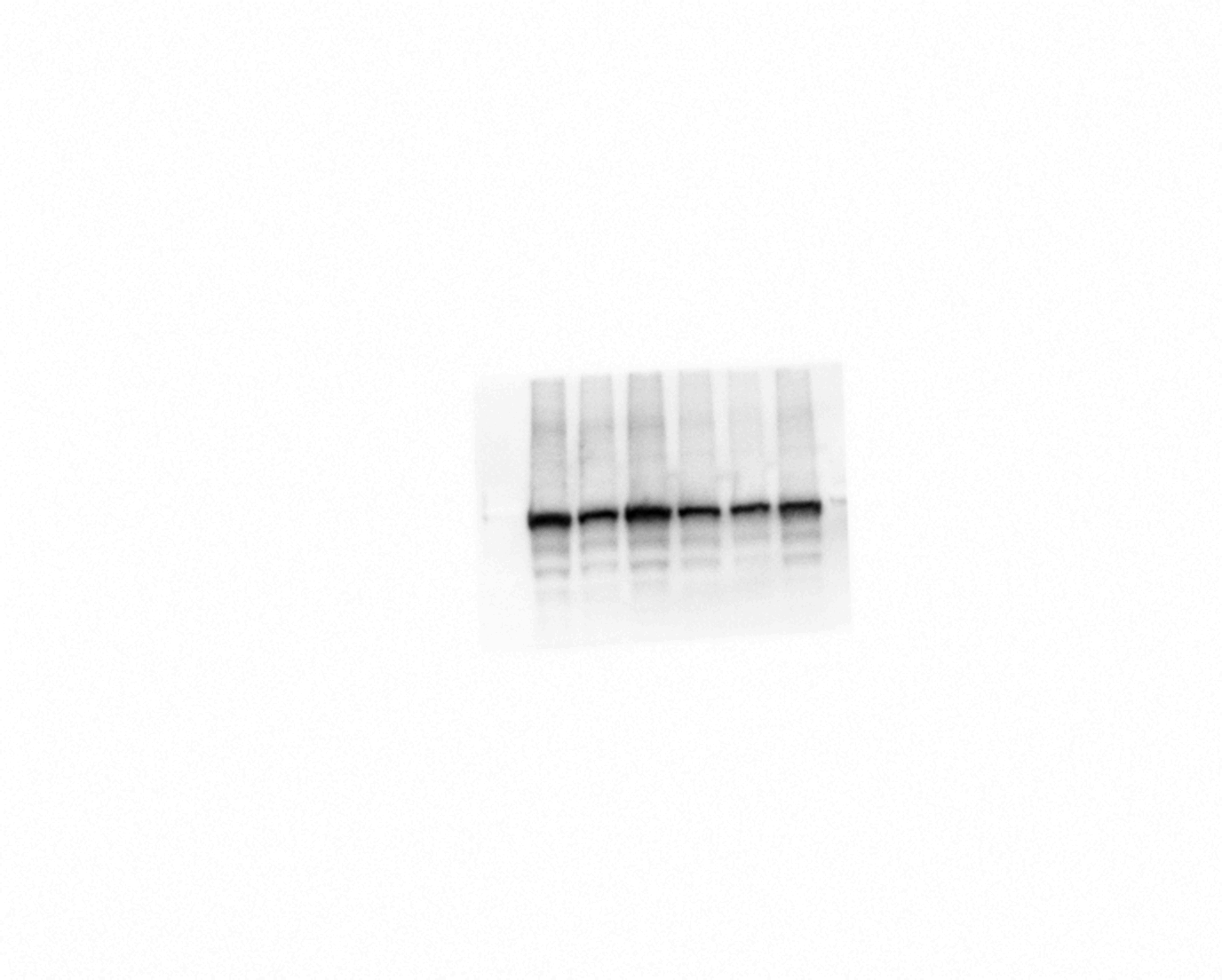

Supplement: Supplementary file 1 [file biomolecules-16-00868-s001.zip › FigureS1 the full, uncropped western blot images/The vivo mice study/HSP60/1-6s.Tif]

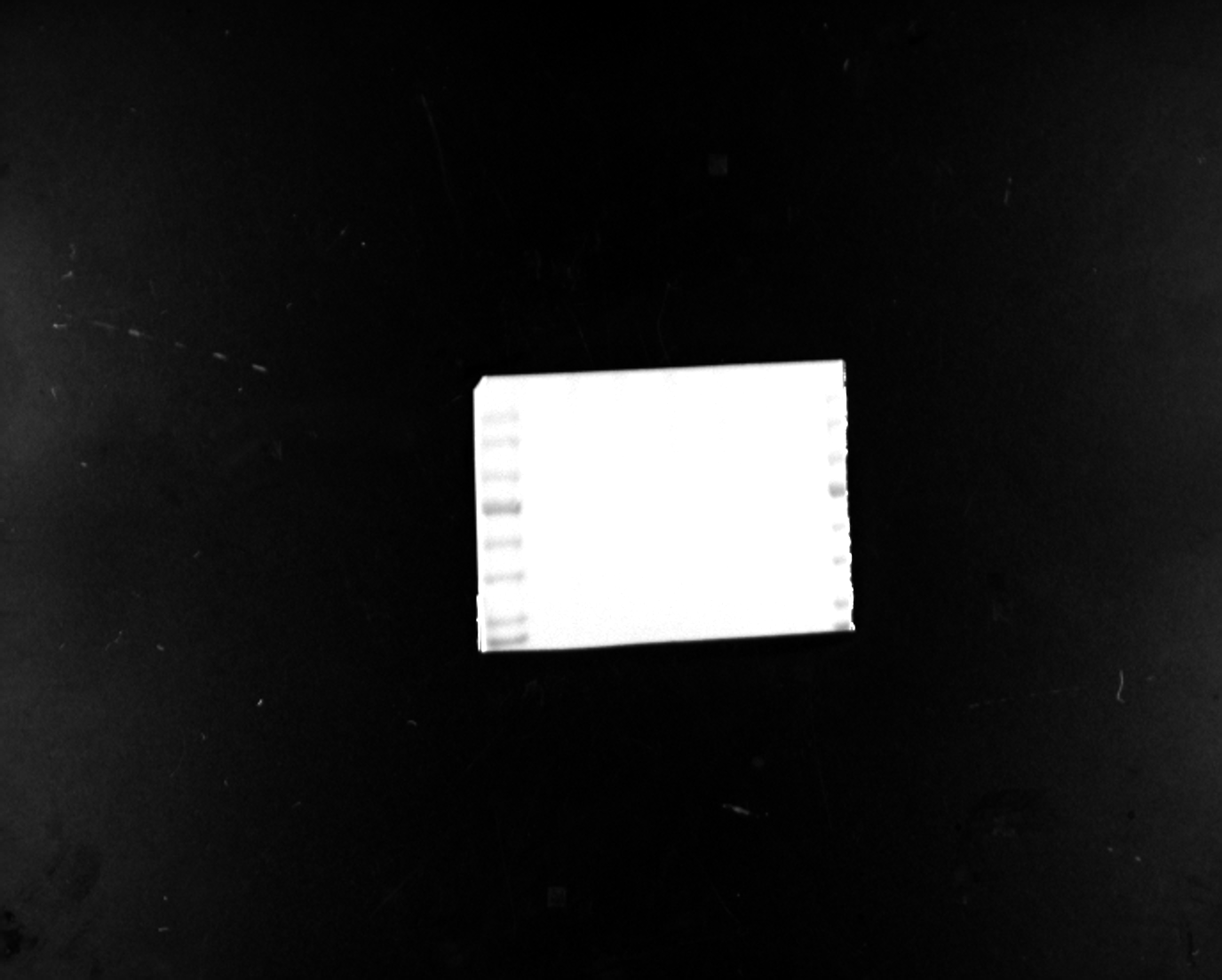

Supplement: Supplementary file 1 [file biomolecules-16-00868-s001.zip › FigureS1 the full, uncropped western blot images/The vivo mice study/HSP60/1-t.Tif]

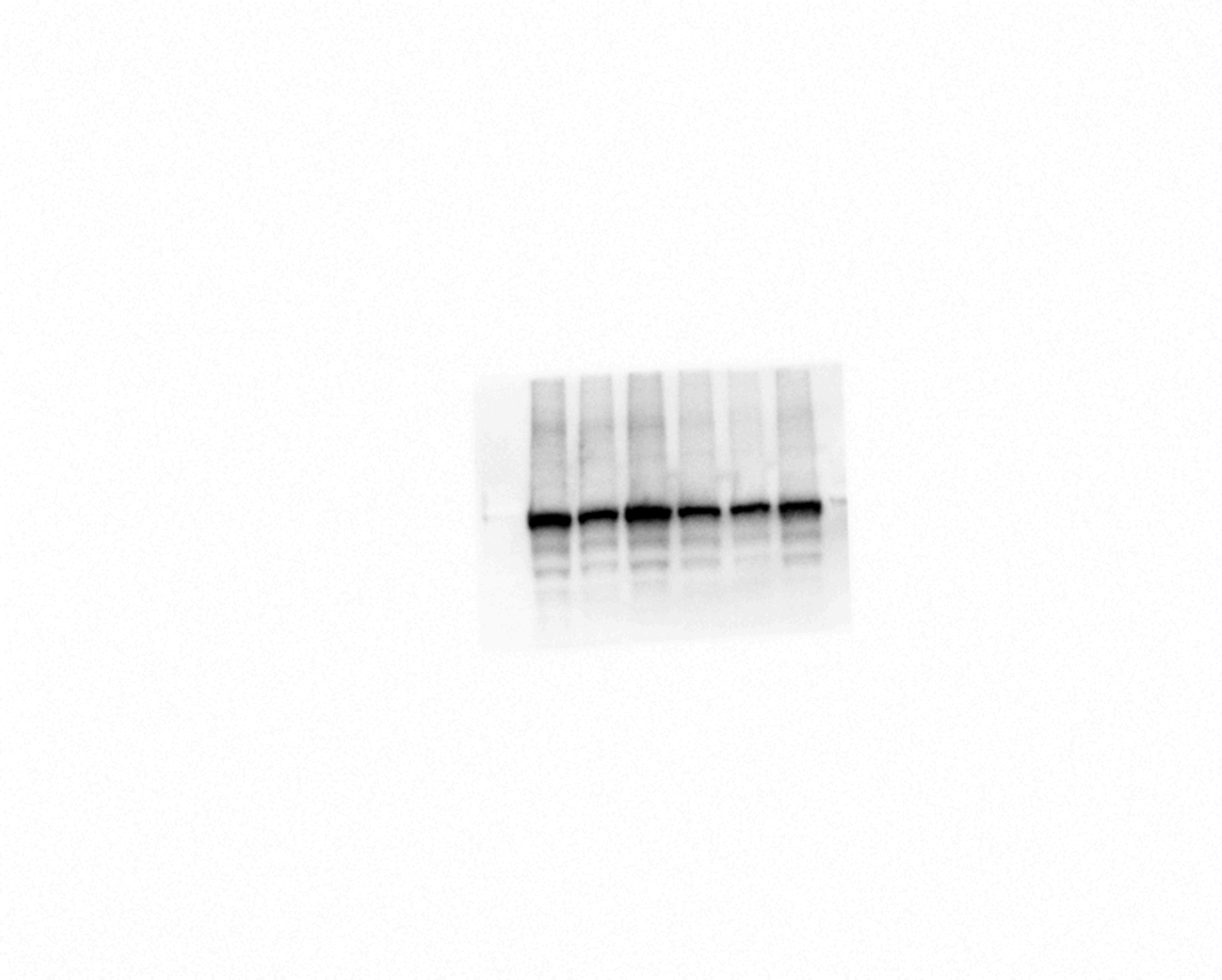

Supplement: Supplementary file 1 [file biomolecules-16-00868-s001.zip › FigureS1 the full, uncropped western blot images/The vivo mice study/HSP60/1.Tif]

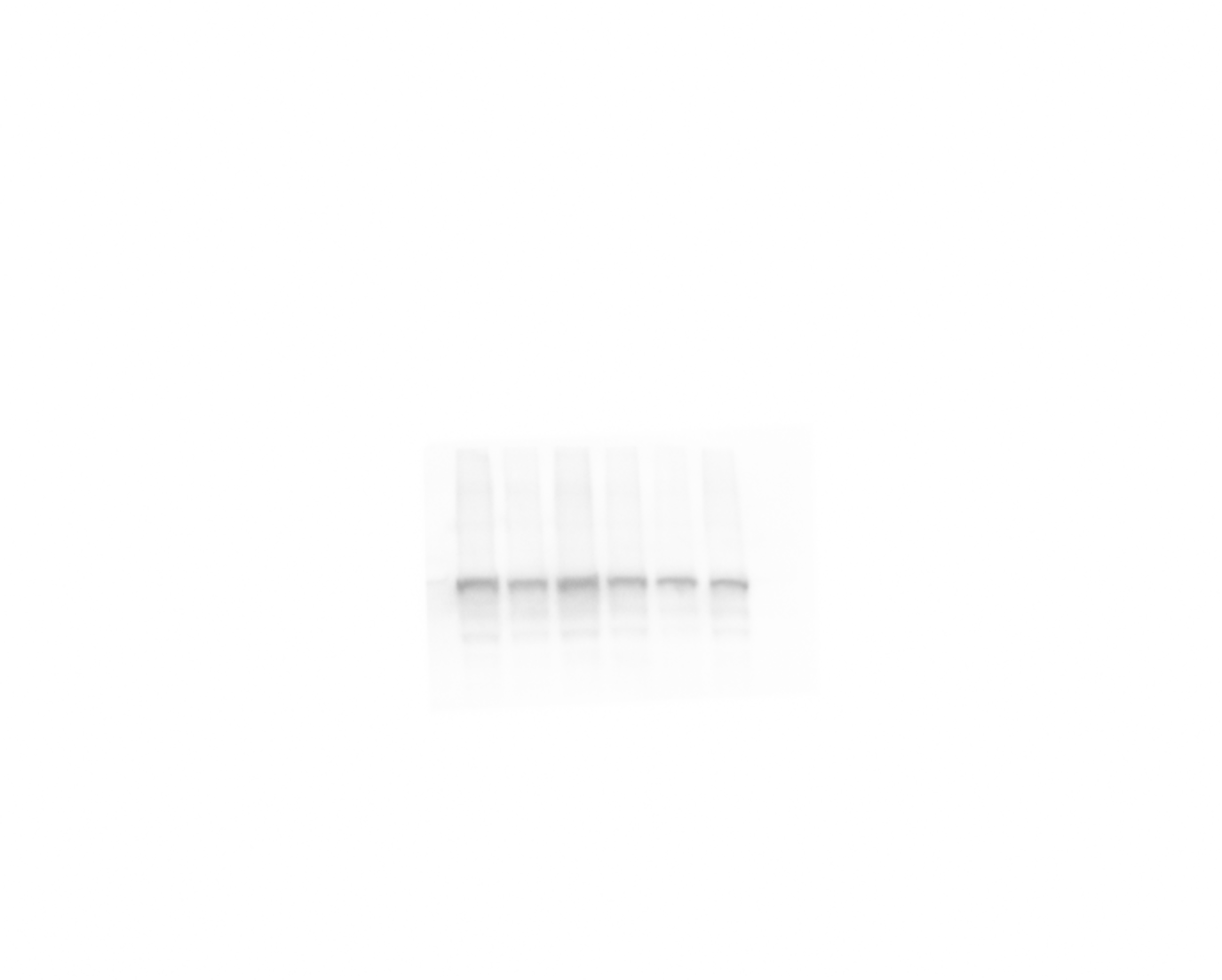

Supplement: Supplementary file 1 [file biomolecules-16-00868-s001.zip › FigureS1 the full, uncropped western blot images/The vivo mice study/HSP60/2-0.4s.Tif]

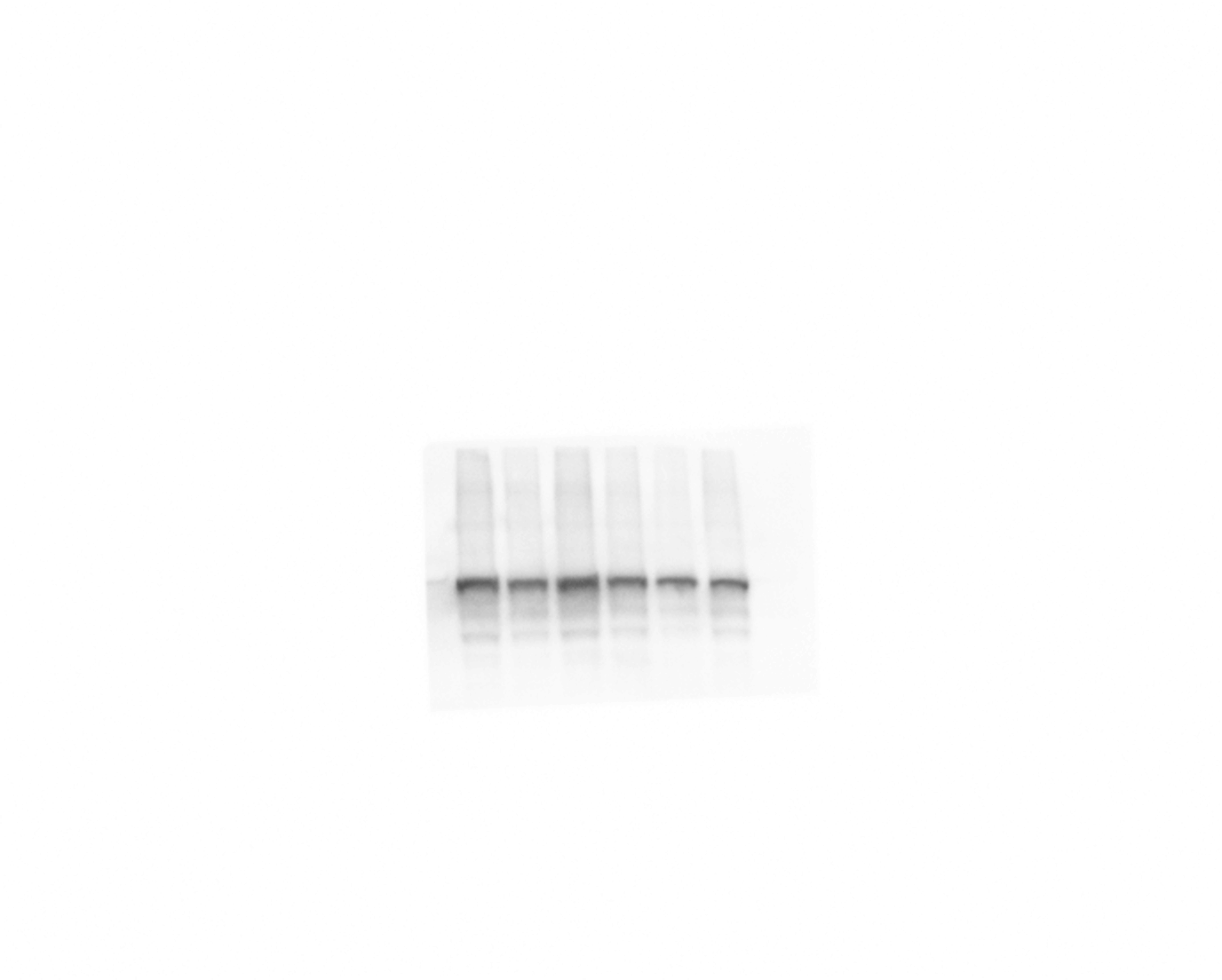

Supplement: Supplementary file 1 [file biomolecules-16-00868-s001.zip › FigureS1 the full, uncropped western blot images/The vivo mice study/HSP60/2-1.2s.Tif]

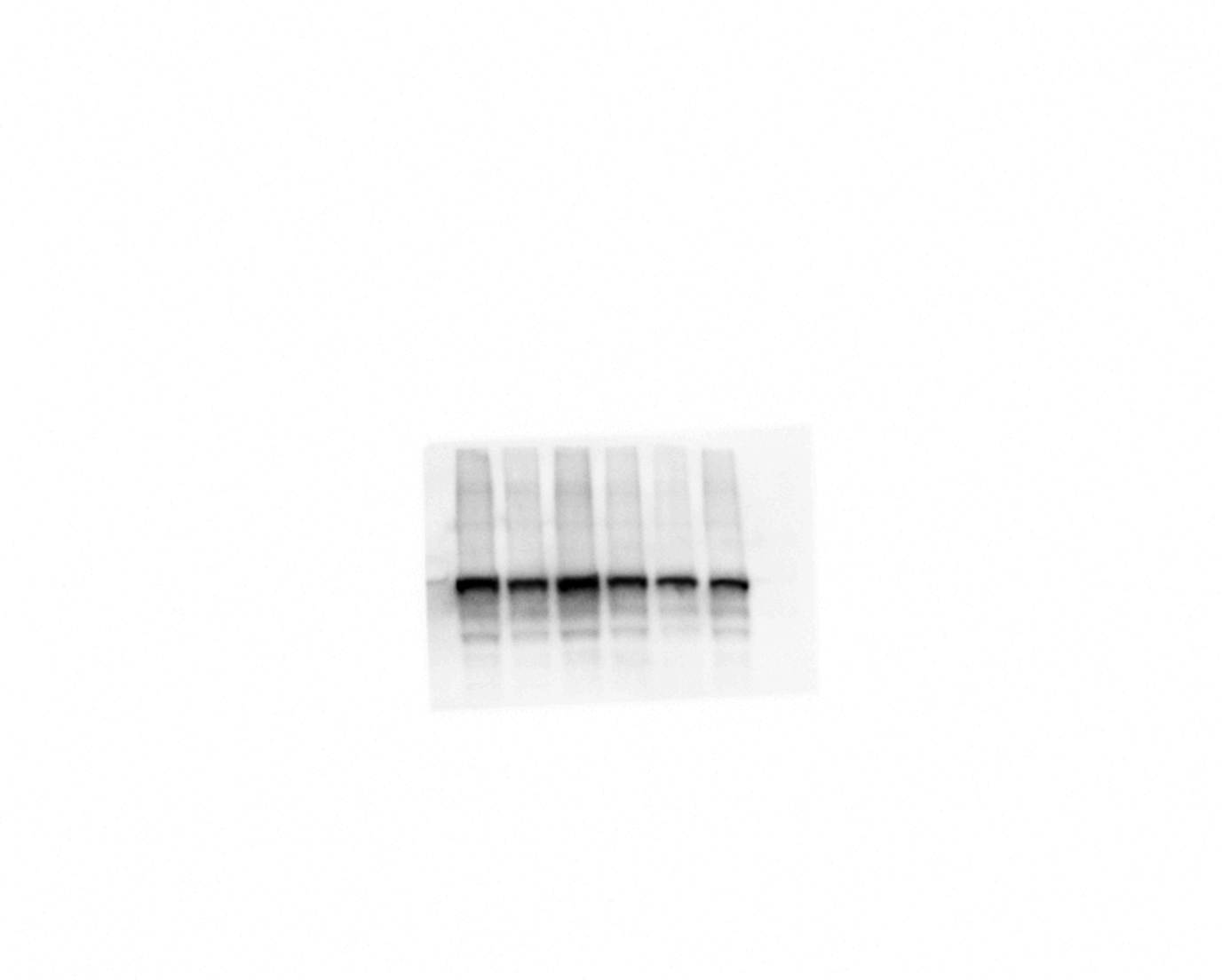

Supplement: Supplementary file 1 [file biomolecules-16-00868-s001.zip › FigureS1 the full, uncropped western blot images/The vivo mice study/HSP60/2-3s.Tif]

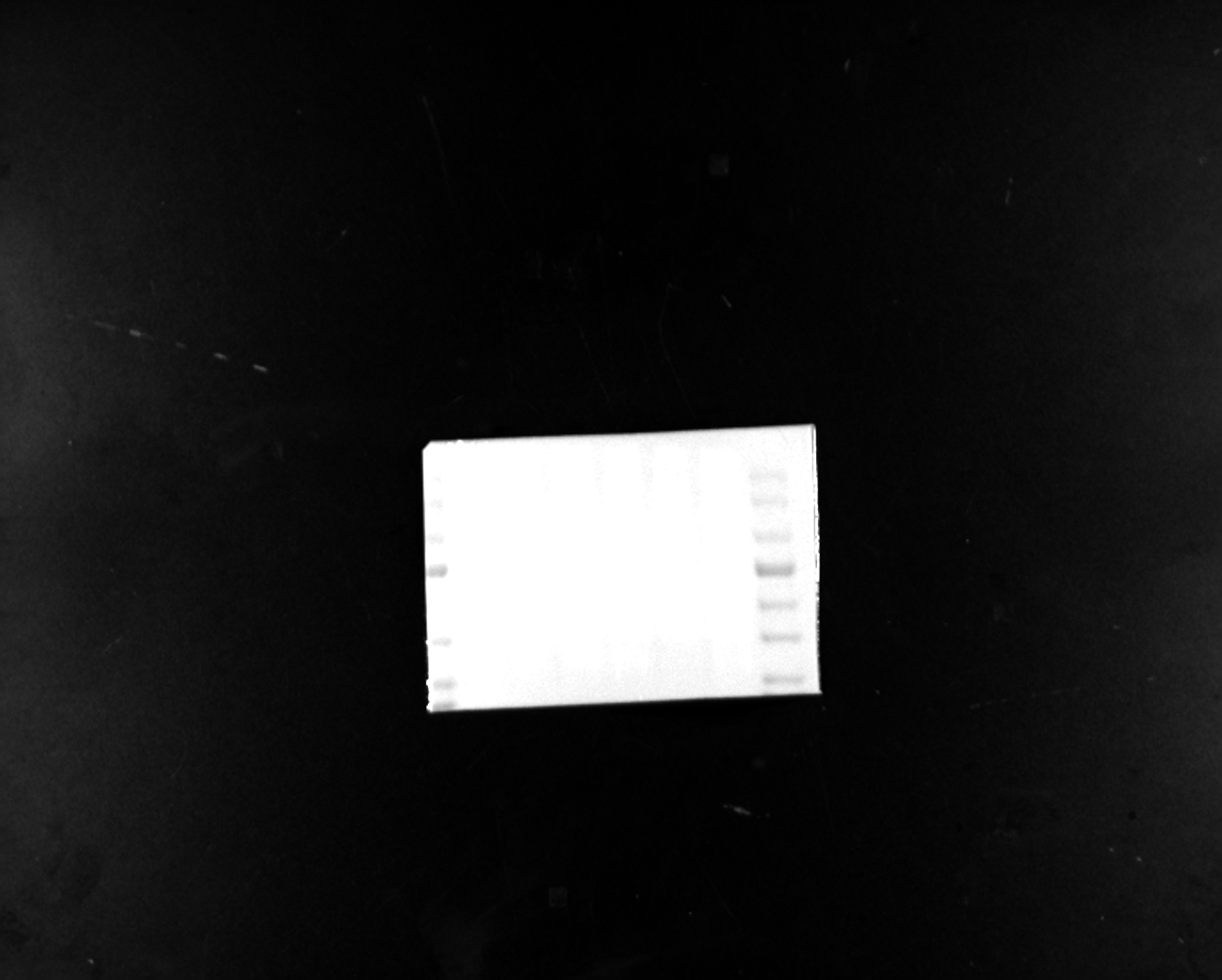

Supplement: Supplementary file 1 [file biomolecules-16-00868-s001.zip › FigureS1 the full, uncropped western blot images/The vivo mice study/HSP60/2-t.Tif]

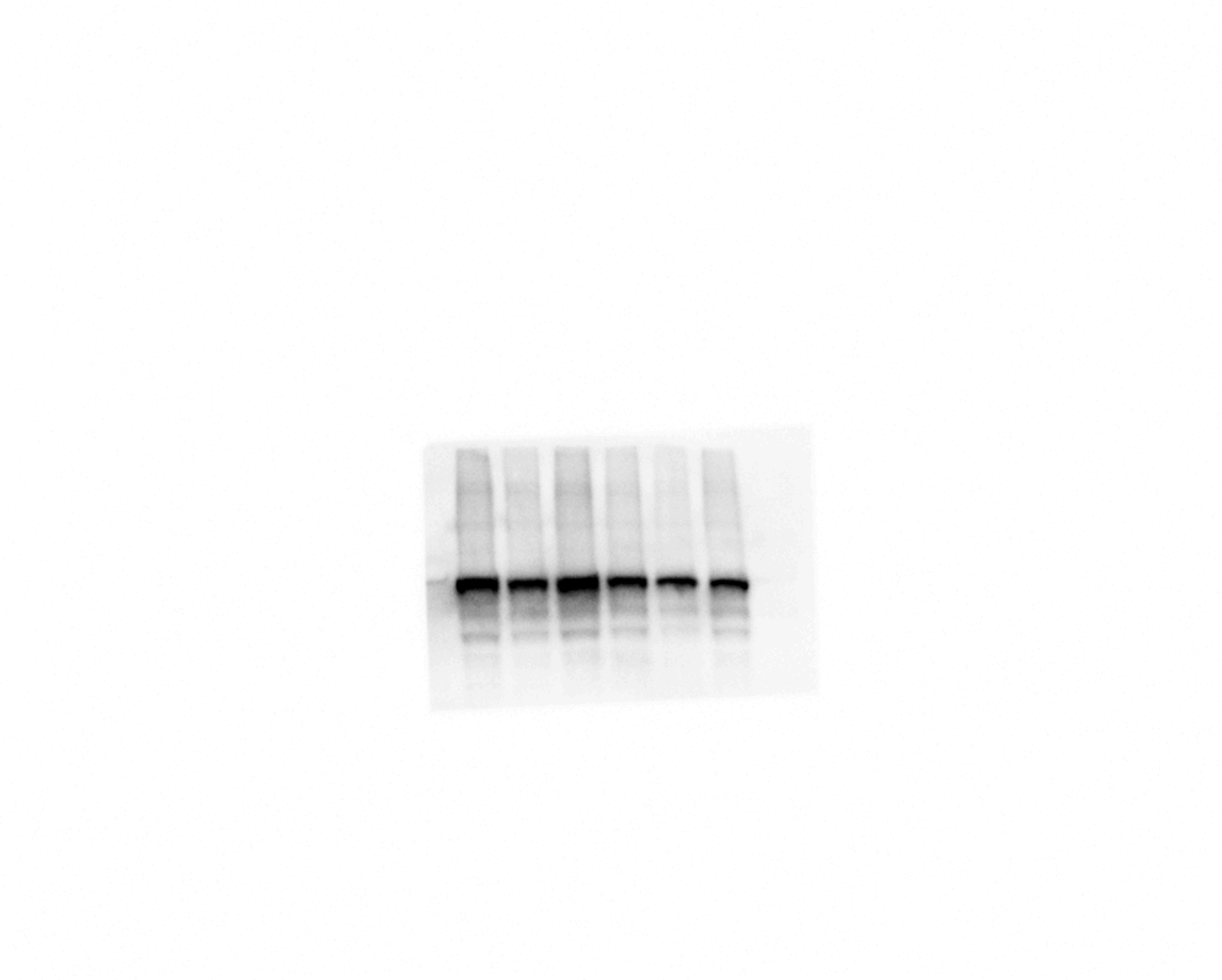

Supplement: Supplementary file 1 [file biomolecules-16-00868-s001.zip › FigureS1 the full, uncropped western blot images/The vivo mice study/HSP60/2.Tif]

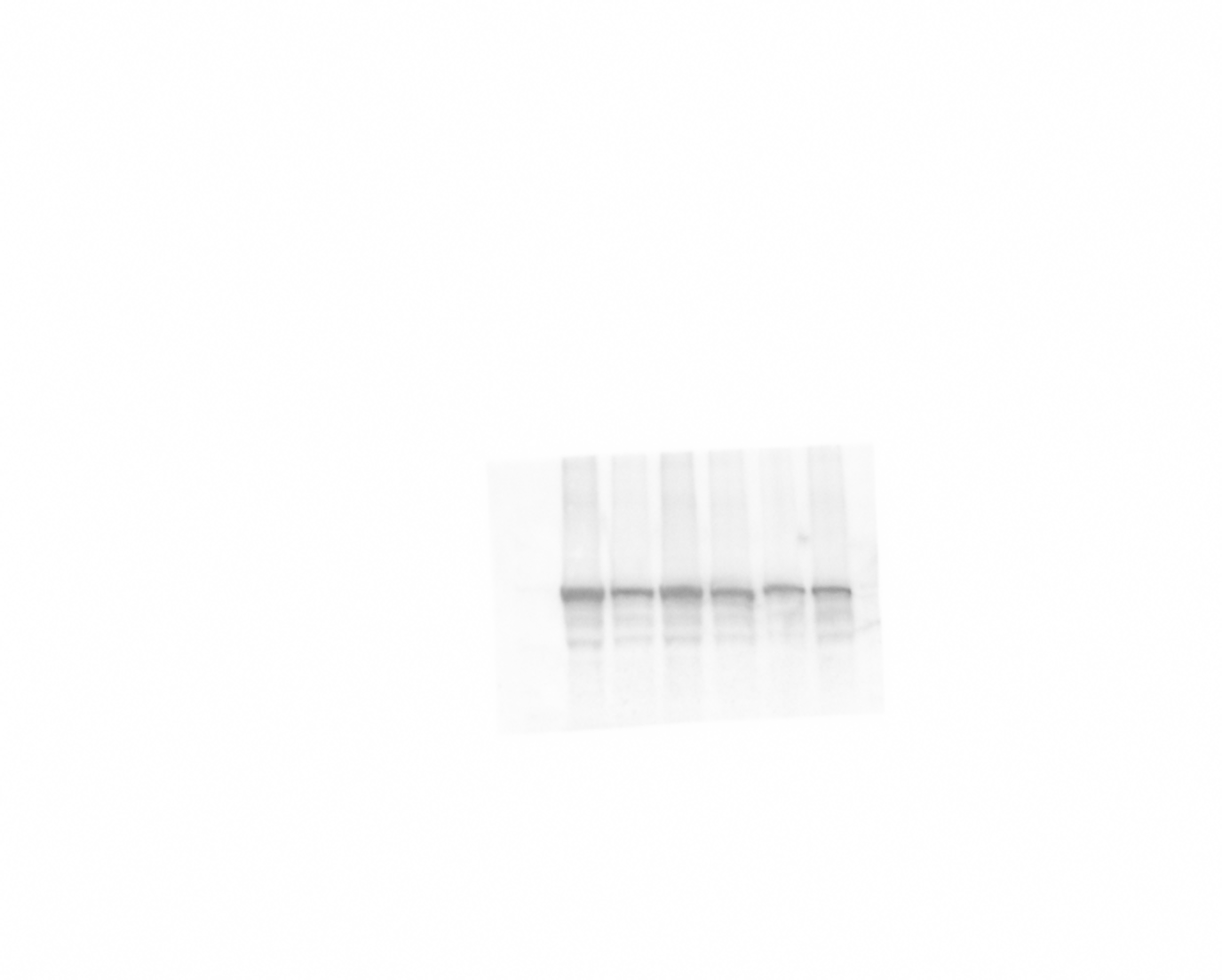

Supplement: Supplementary file 1 [file biomolecules-16-00868-s001.zip › FigureS1 the full, uncropped western blot images/The vivo mice study/HSP60/3-0.5s.Tif]

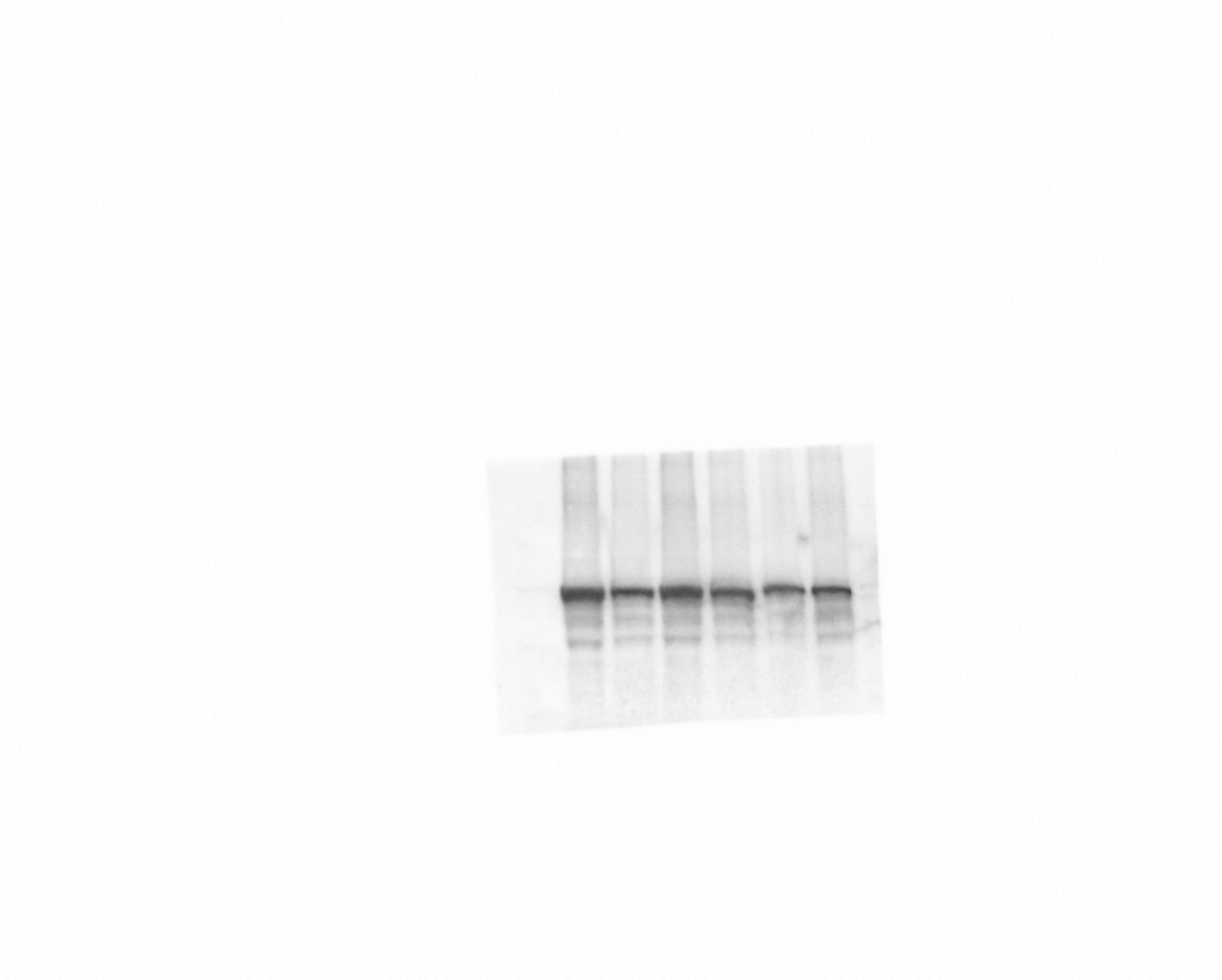

Supplement: Supplementary file 1 [file biomolecules-16-00868-s001.zip › FigureS1 the full, uncropped western blot images/The vivo mice study/HSP60/3-2s.Tif]

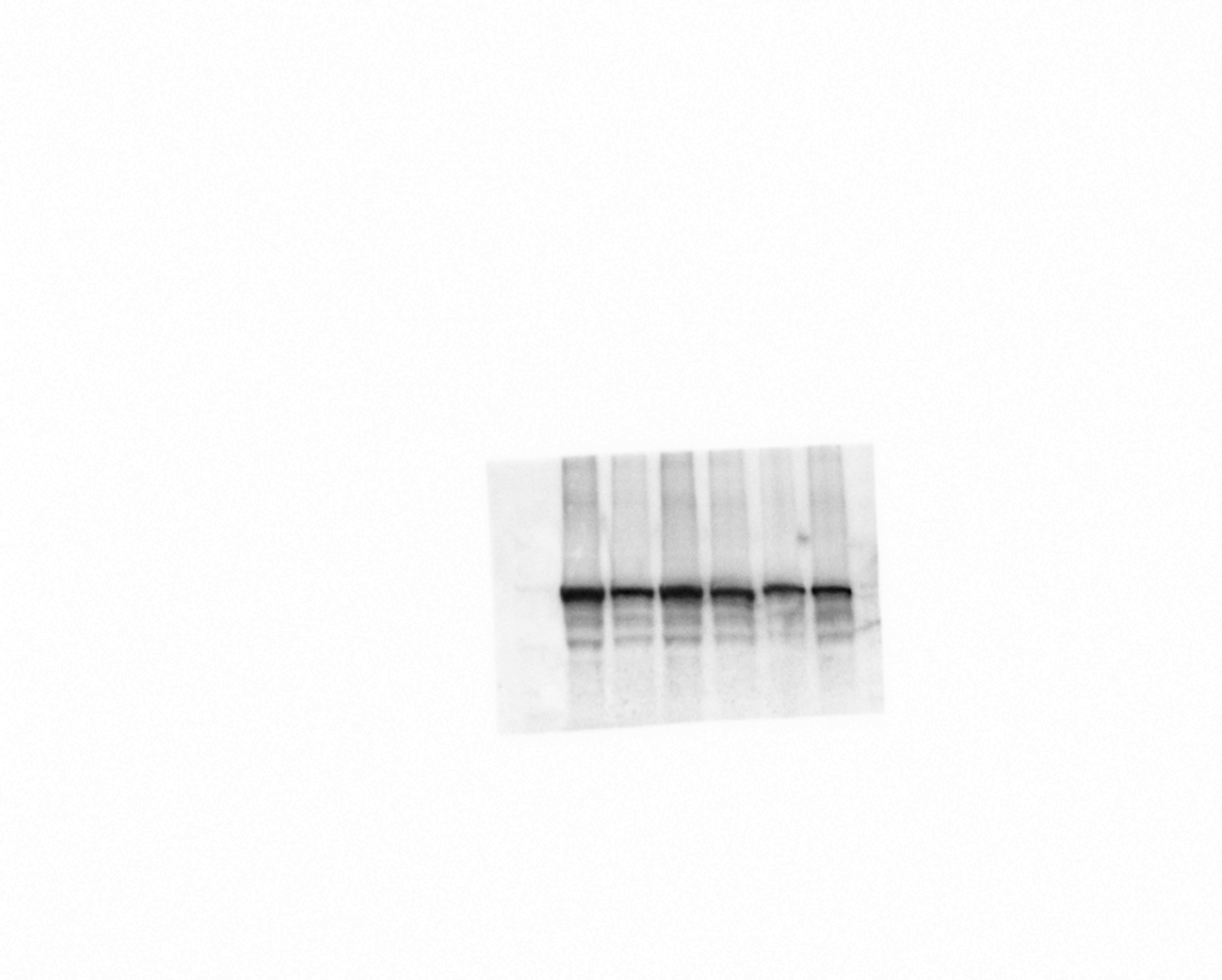

Supplement: Supplementary file 1 [file biomolecules-16-00868-s001.zip › FigureS1 the full, uncropped western blot images/The vivo mice study/HSP60/3-6s.Tif]

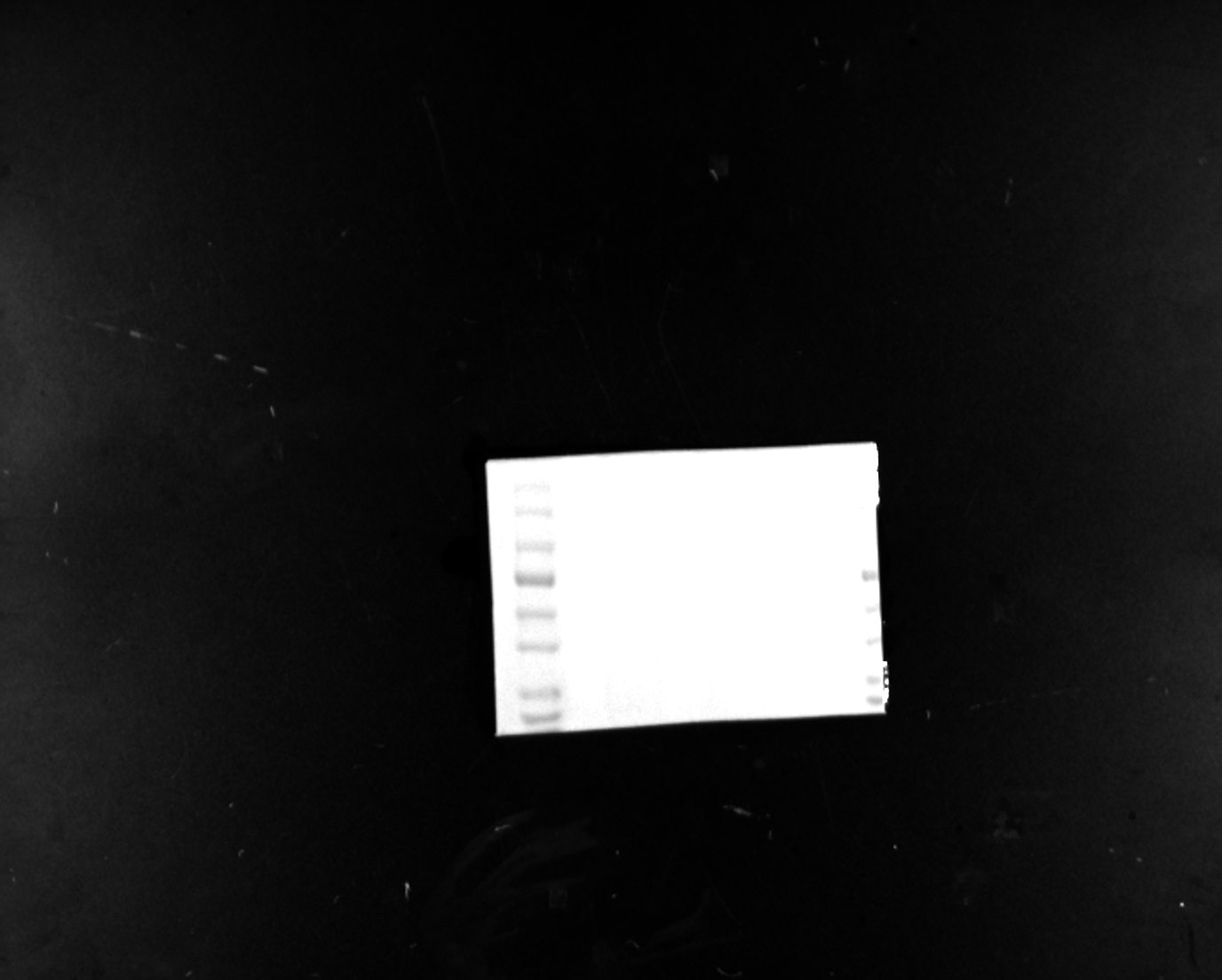

Supplement: Supplementary file 1 [file biomolecules-16-00868-s001.zip › FigureS1 the full, uncropped western blot images/The vivo mice study/HSP60/3-t.Tif]

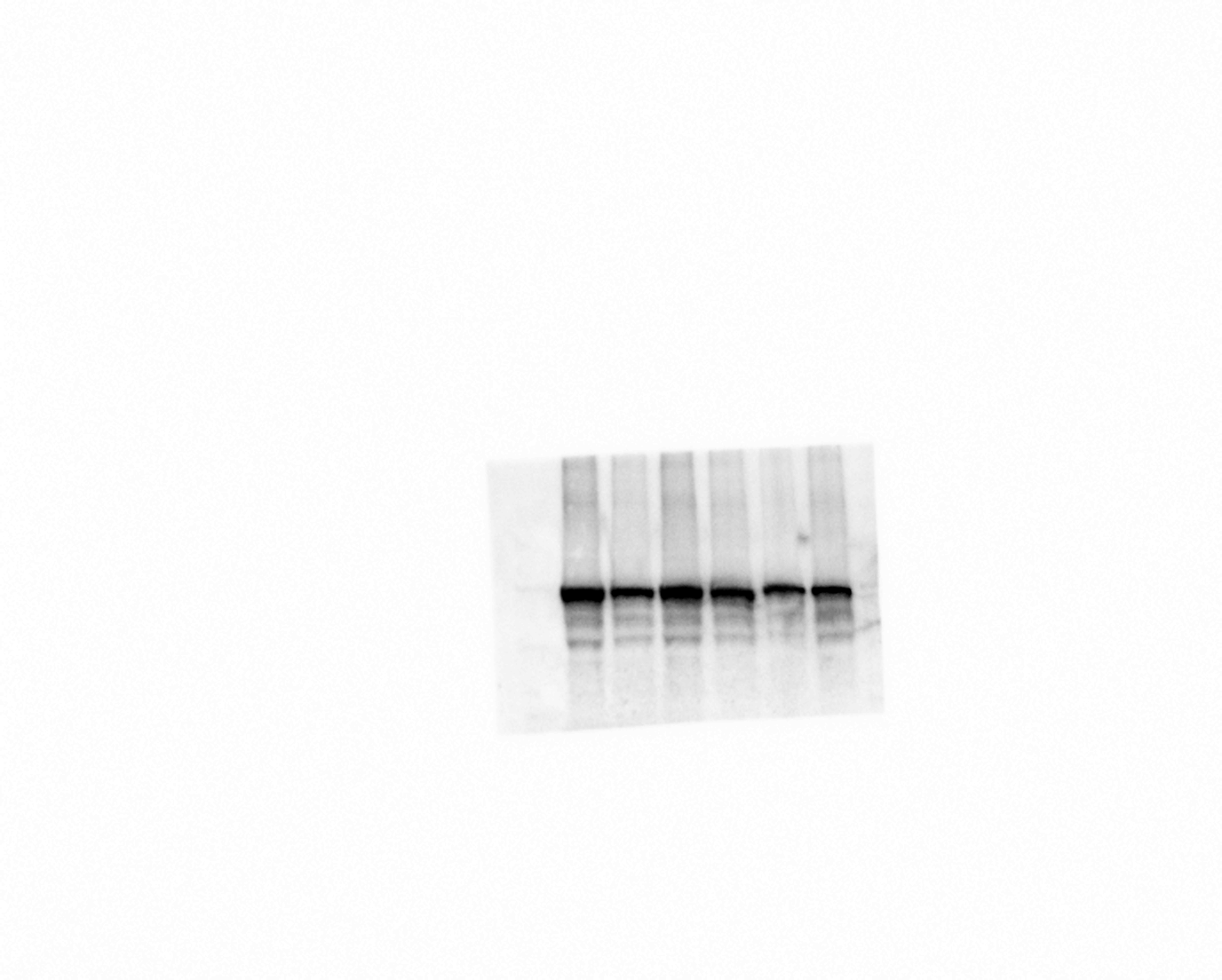

Supplement: Supplementary file 1 [file biomolecules-16-00868-s001.zip › FigureS1 the full, uncropped western blot images/The vivo mice study/HSP60/3.Tif]

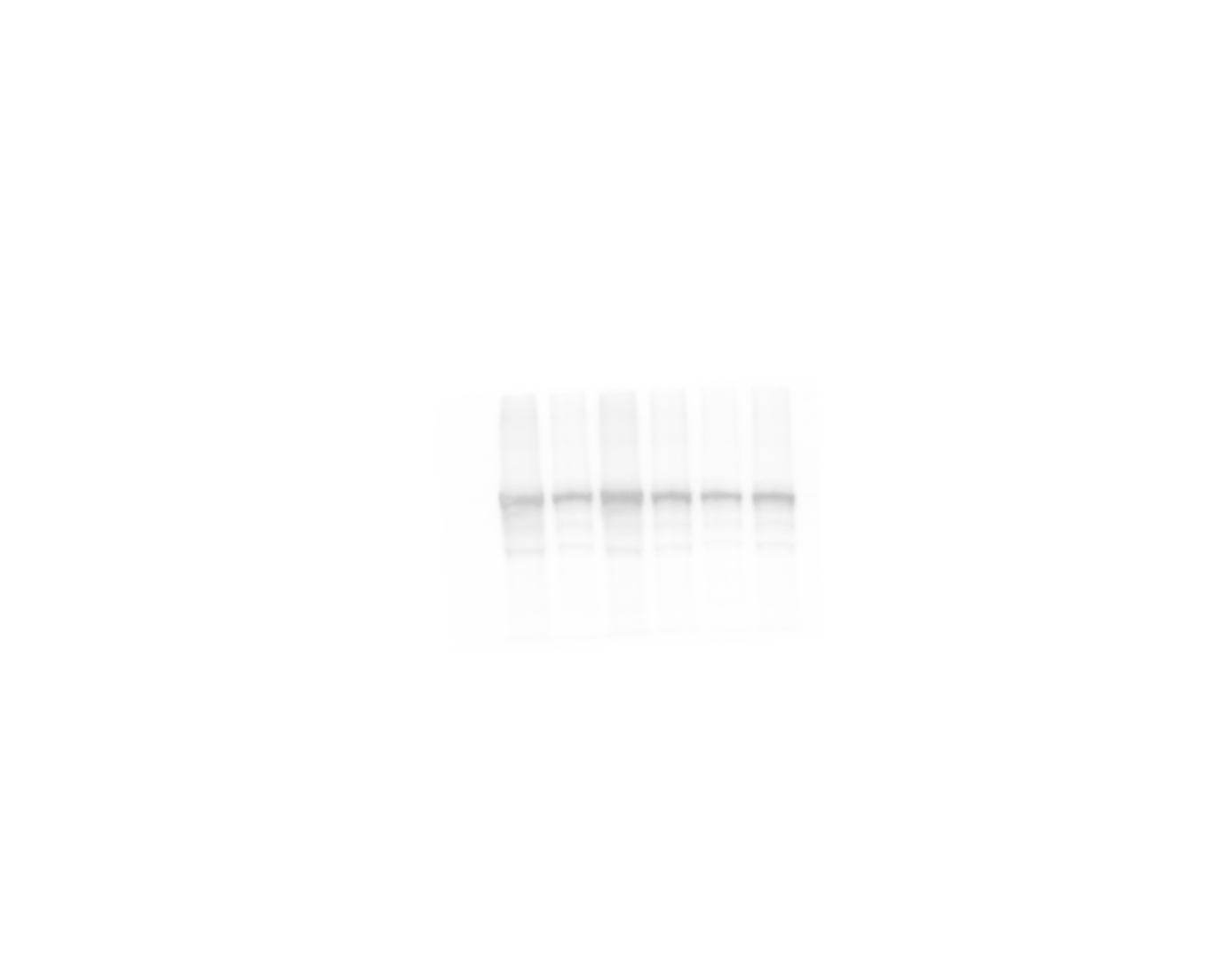

Supplement: Supplementary file 1 [file biomolecules-16-00868-s001.zip › FigureS1 the full, uncropped western blot images/The vivo mice study/HSP60/4-0.4s.Tif]

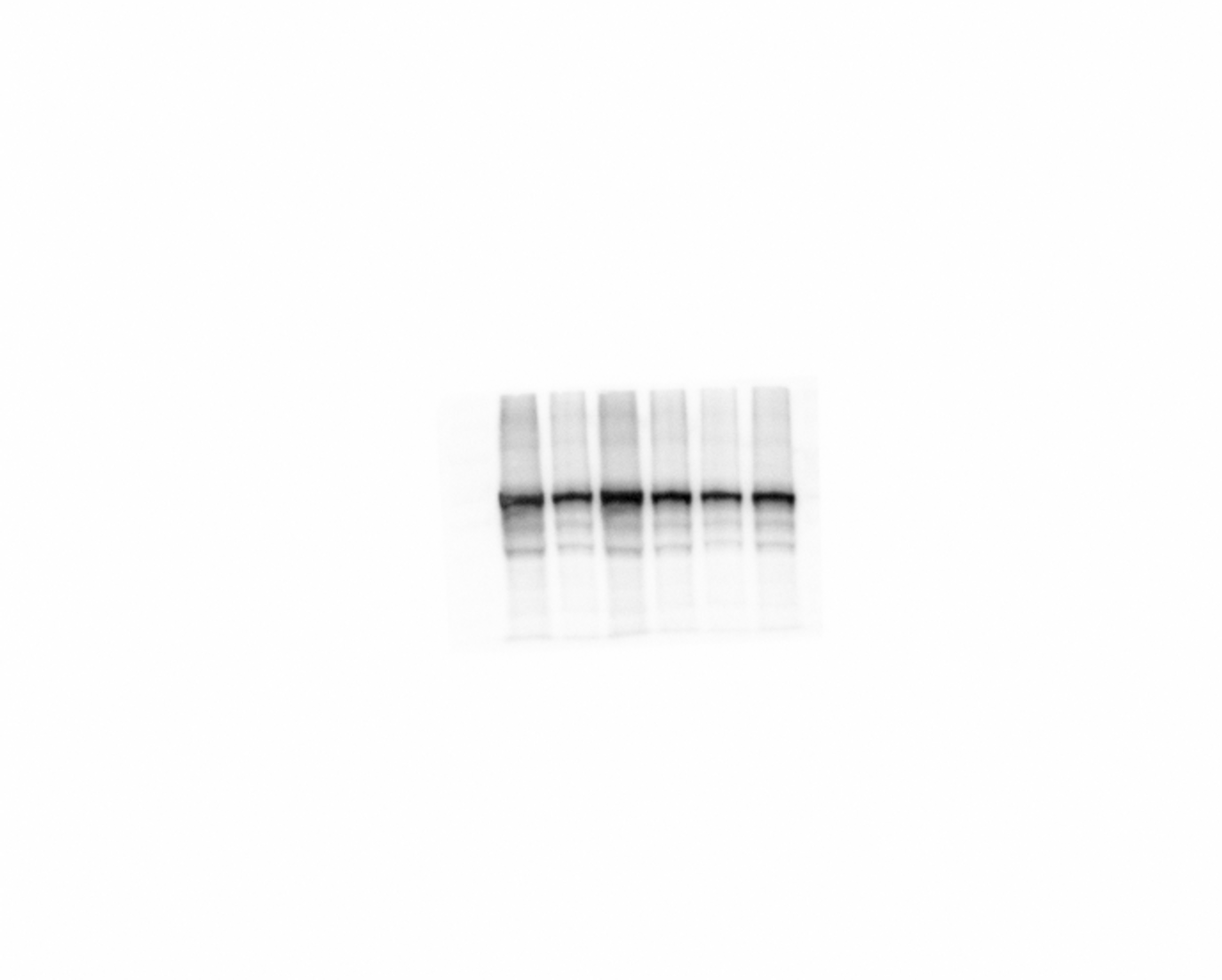

Supplement: Supplementary file 1 [file biomolecules-16-00868-s001.zip › FigureS1 the full, uncropped western blot images/The vivo mice study/HSP60/4-2s.Tif]

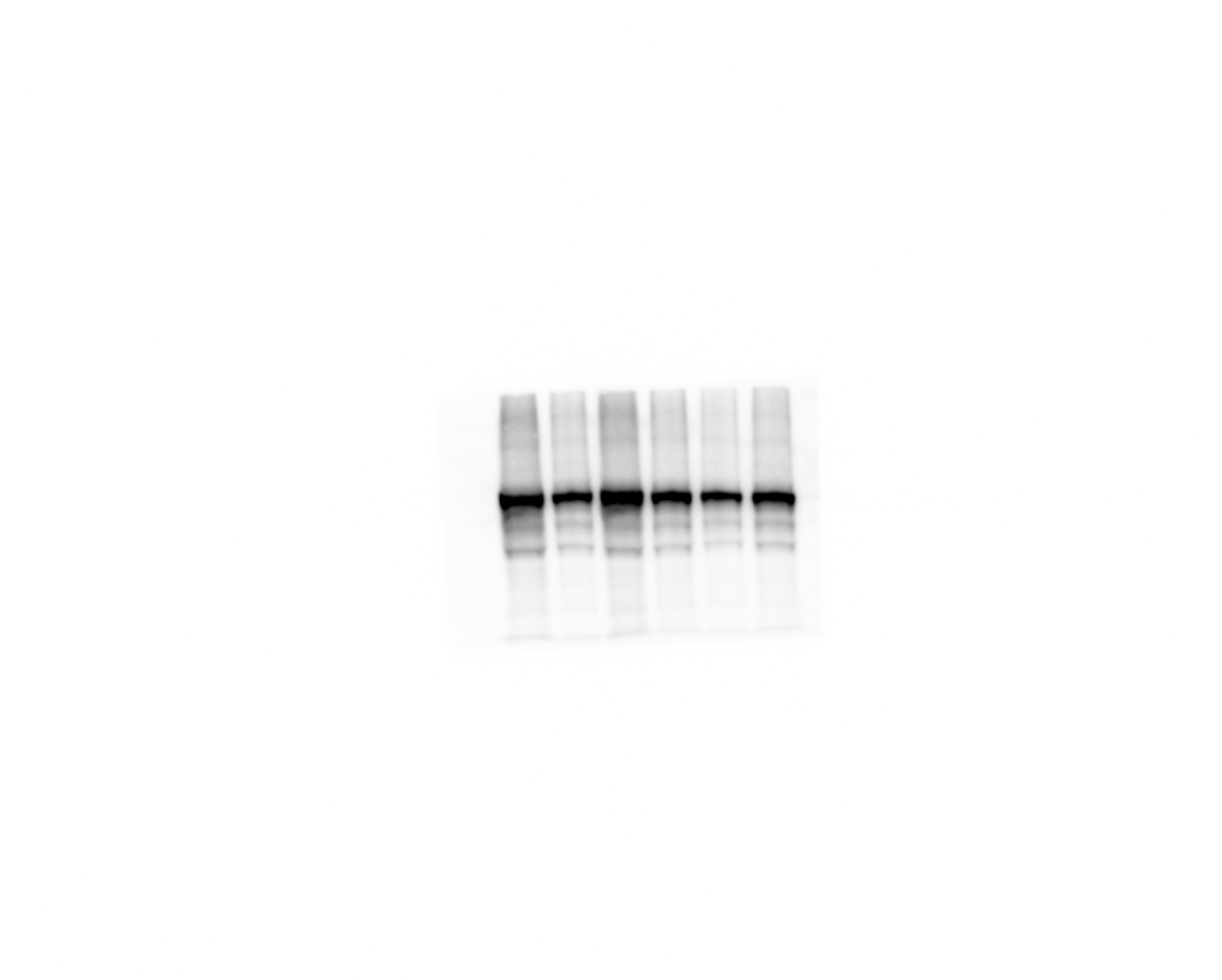

Supplement: Supplementary file 1 [file biomolecules-16-00868-s001.zip › FigureS1 the full, uncropped western blot images/The vivo mice study/HSP60/4-4s.Tif]

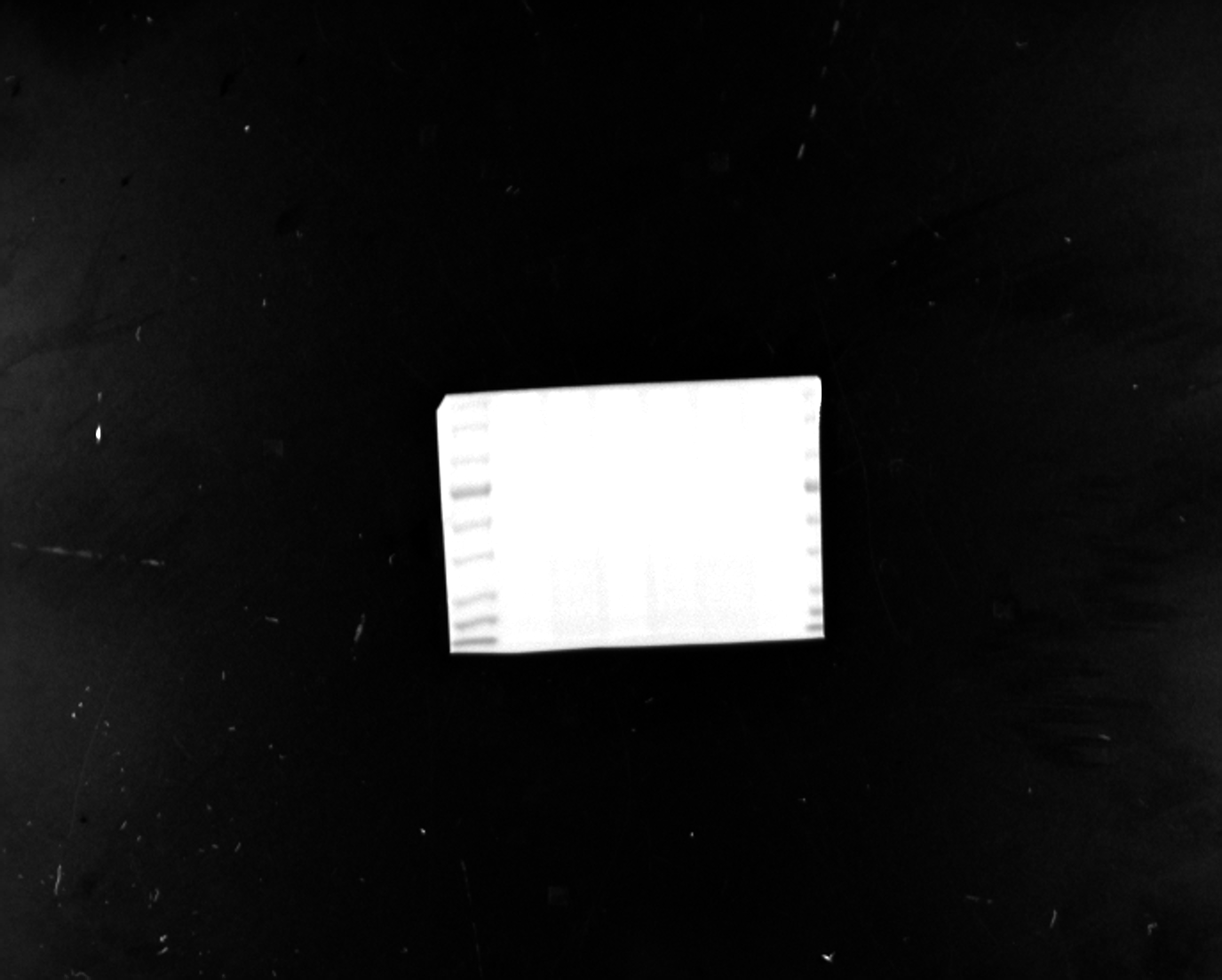

Supplement: Supplementary file 1 [file biomolecules-16-00868-s001.zip › FigureS1 the full, uncropped western blot images/The vivo mice study/HSP60/4-t.Tif]

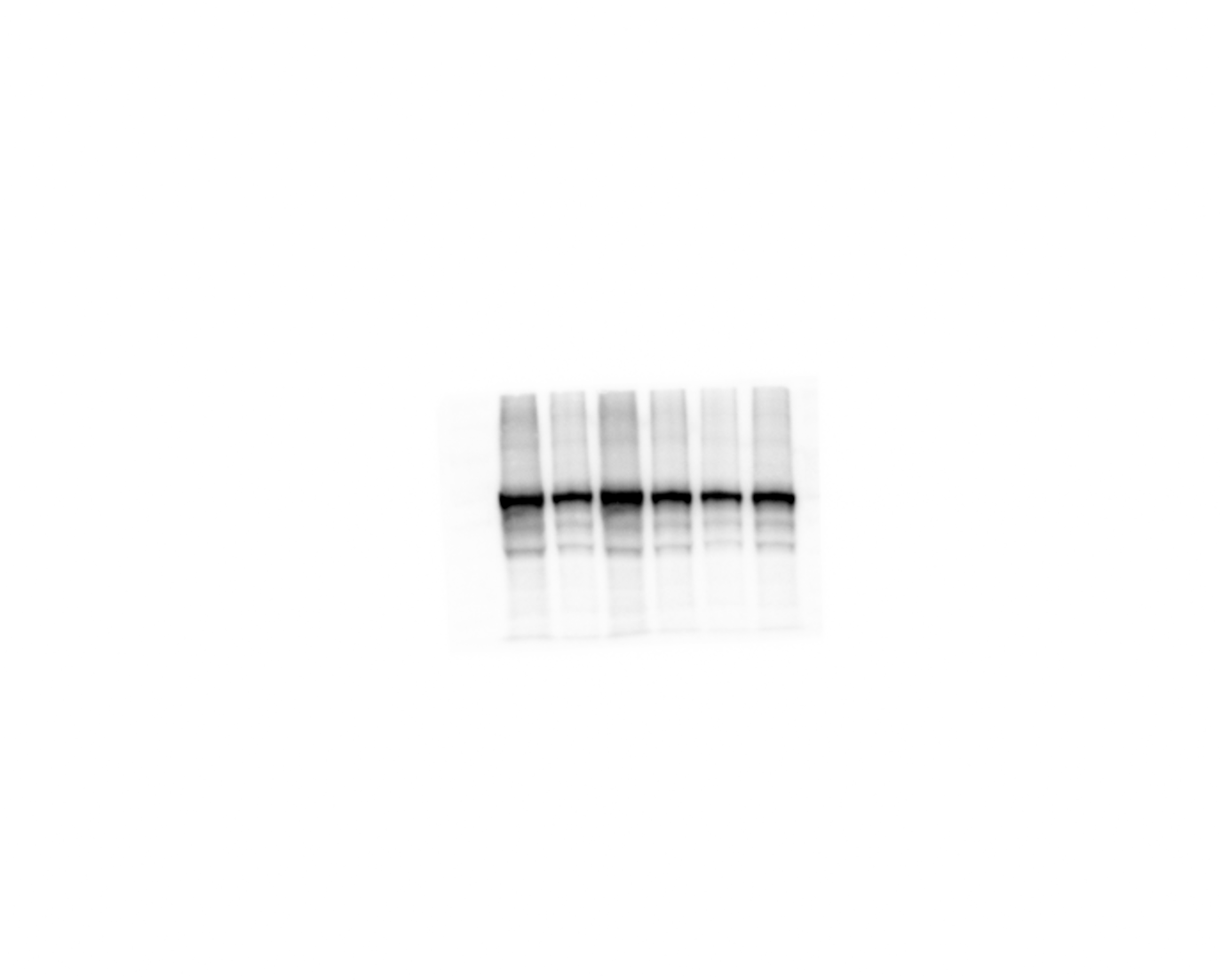

Supplement: Supplementary file 1 [file biomolecules-16-00868-s001.zip › FigureS1 the full, uncropped western blot images/The vivo mice study/HSP60/4.Tif]

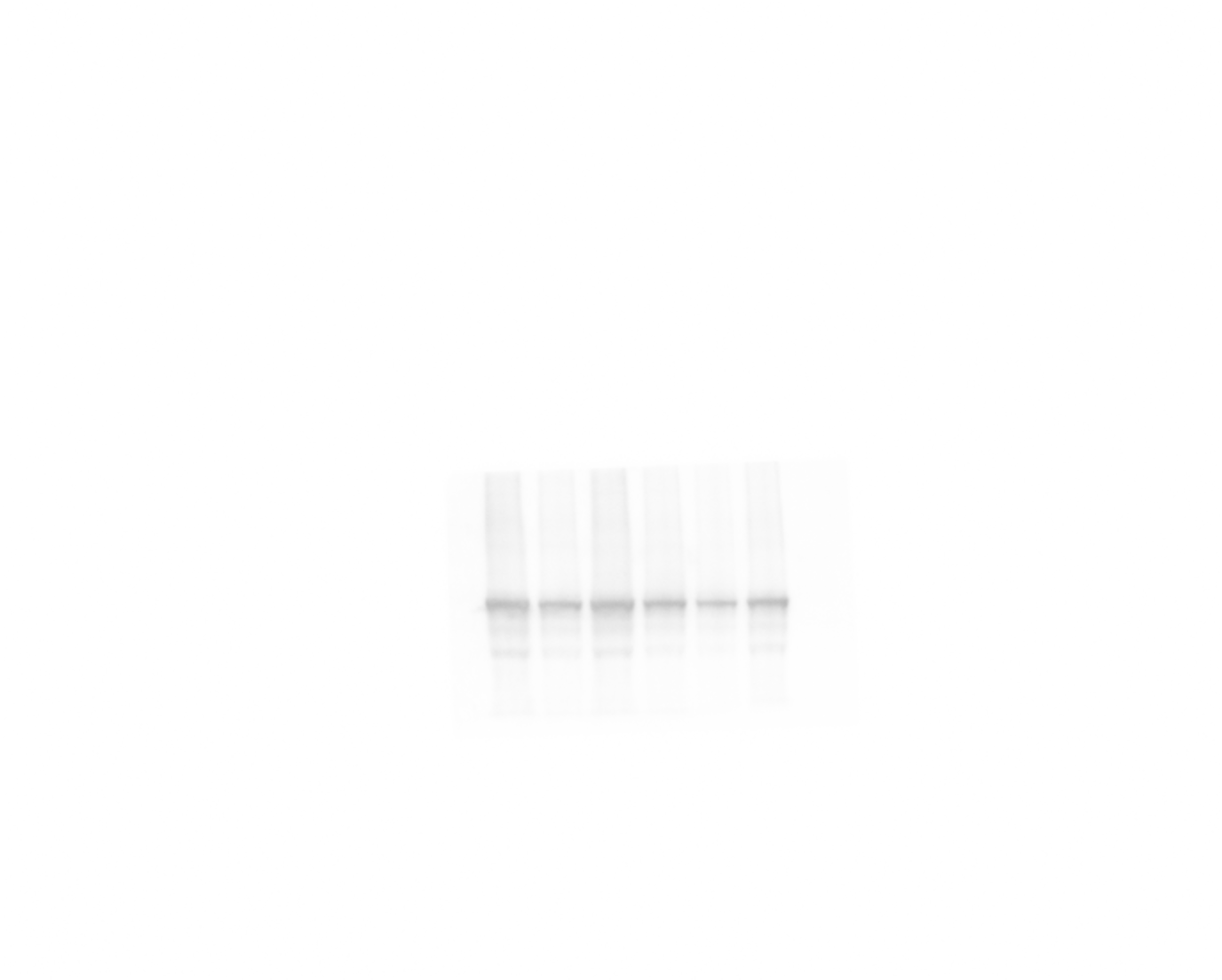

Supplement: Supplementary file 1 [file biomolecules-16-00868-s001.zip › FigureS1 the full, uncropped western blot images/The vivo mice study/HSP60/5-0.5s.Tif]

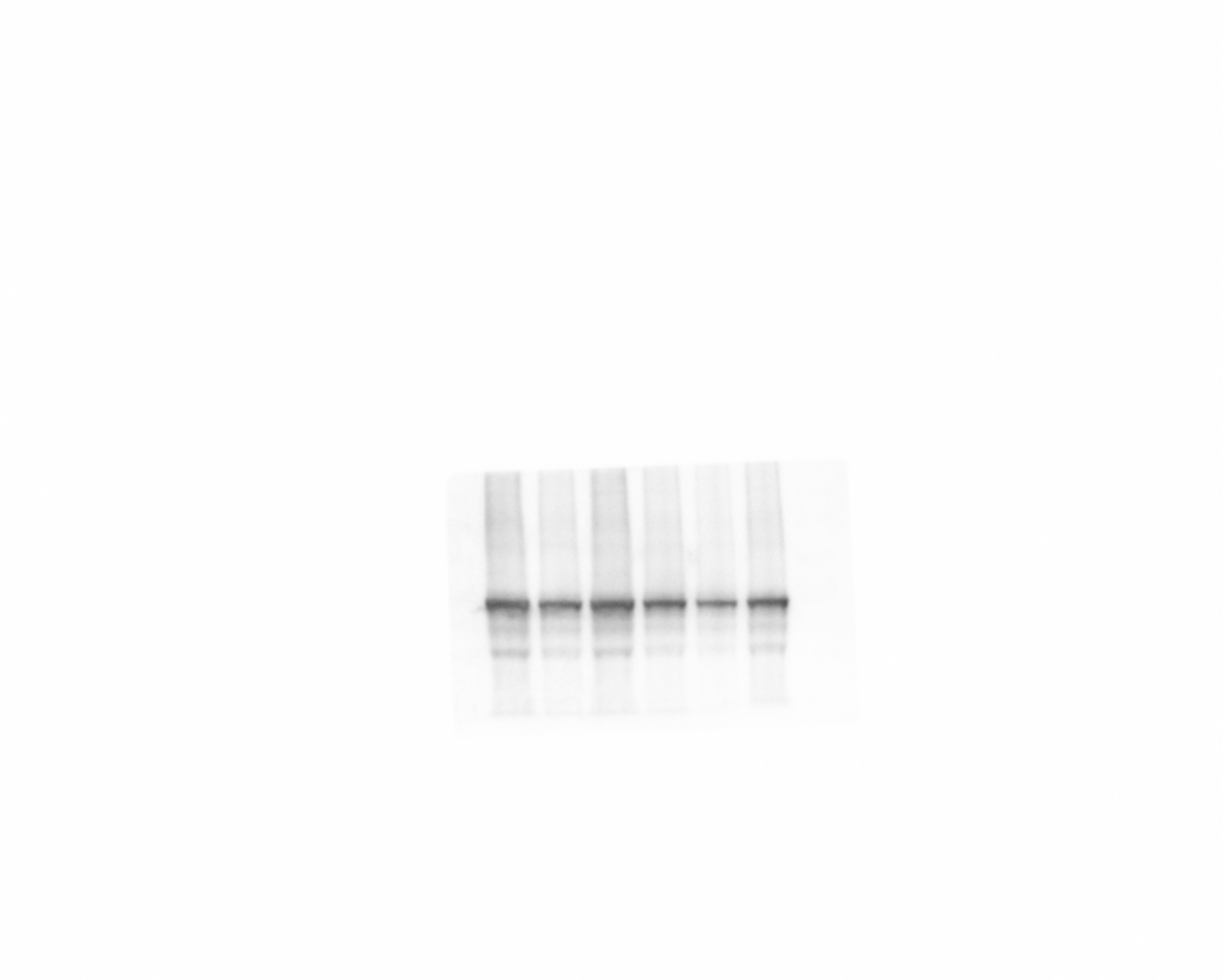

Supplement: Supplementary file 1 [file biomolecules-16-00868-s001.zip › FigureS1 the full, uncropped western blot images/The vivo mice study/HSP60/5-2s.Tif]

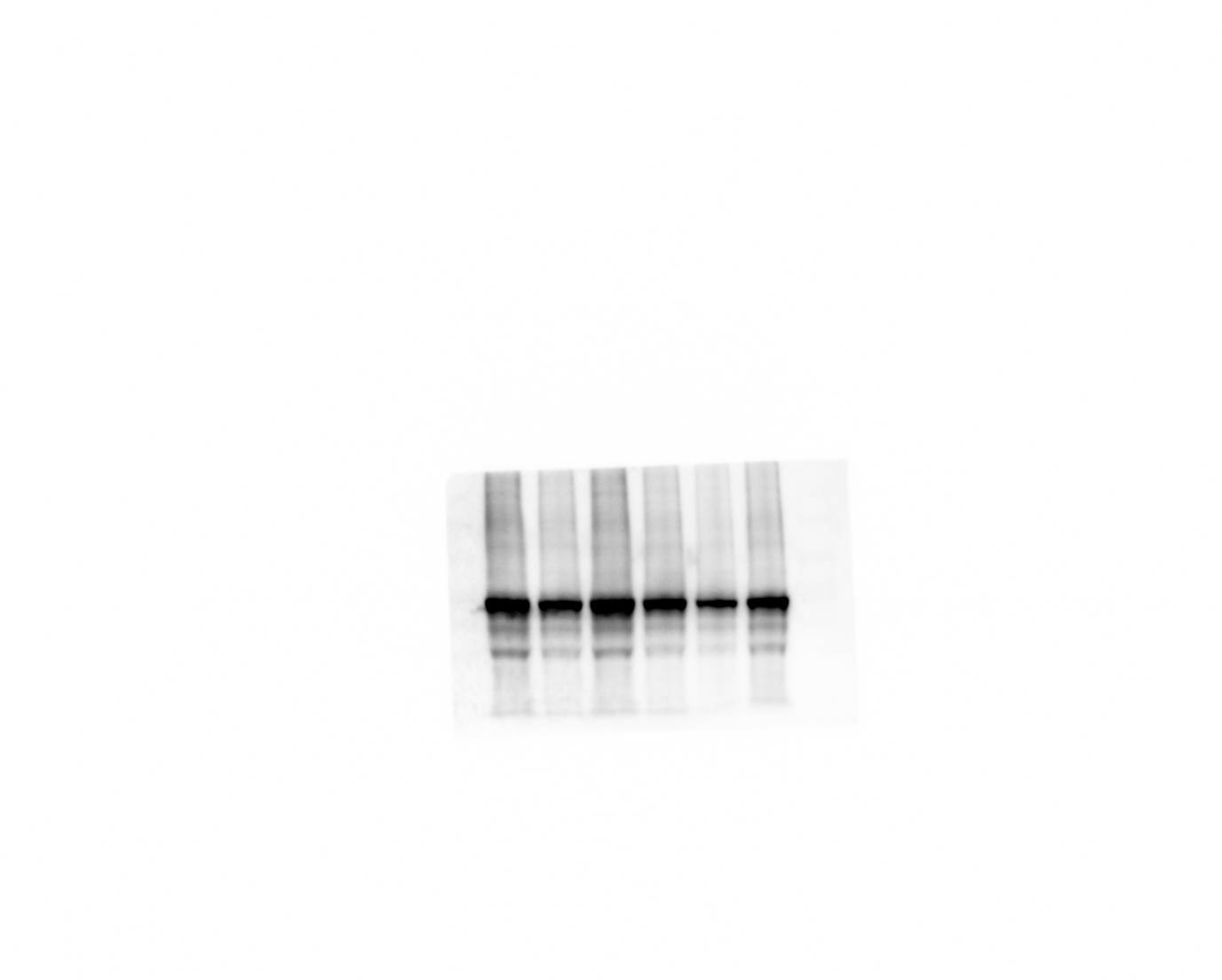

Supplement: Supplementary file 1 [file biomolecules-16-00868-s001.zip › FigureS1 the full, uncropped western blot images/The vivo mice study/HSP60/5-5s.Tif]

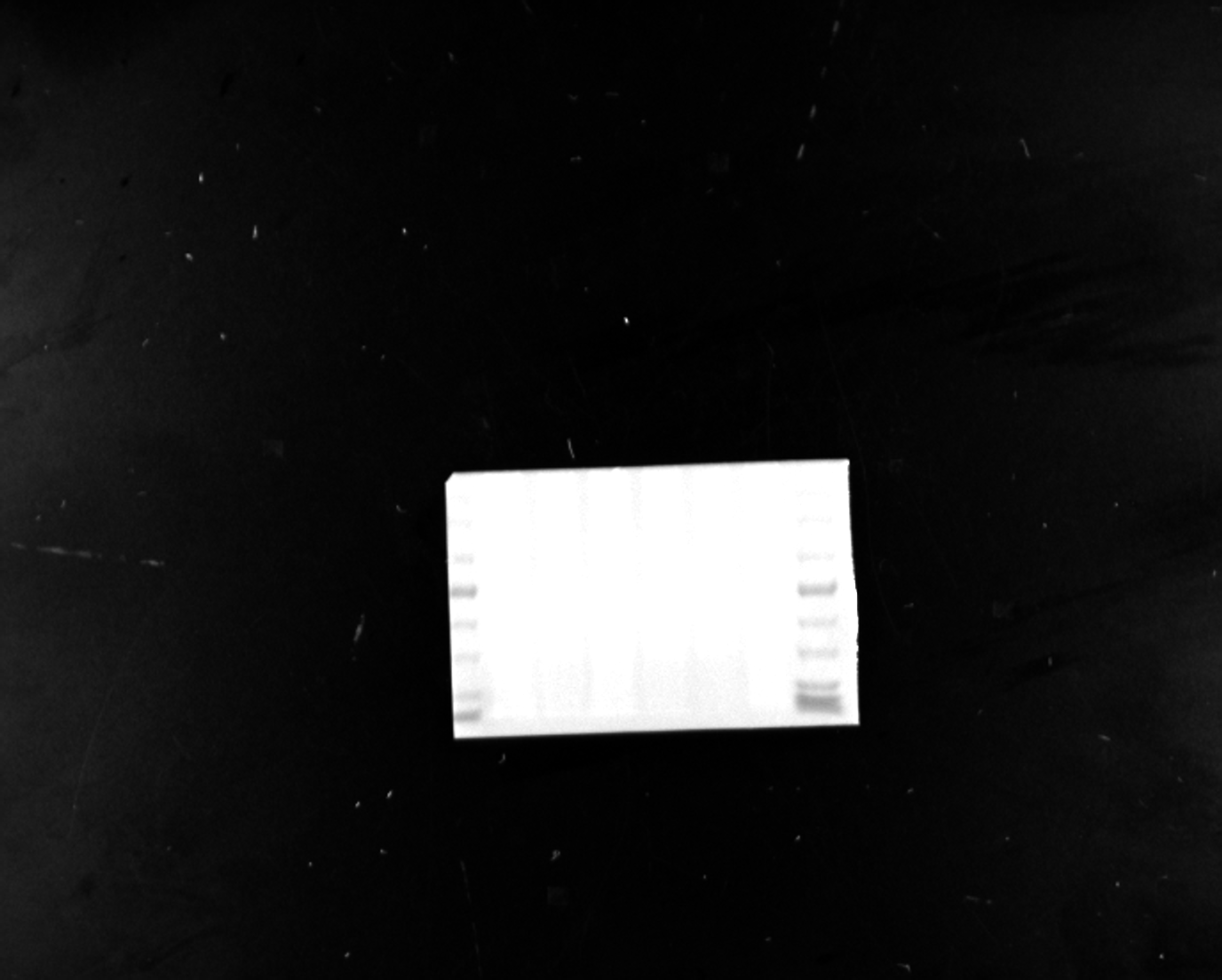

Supplement: Supplementary file 1 [file biomolecules-16-00868-s001.zip › FigureS1 the full, uncropped western blot images/The vivo mice study/HSP60/5-t.Tif]

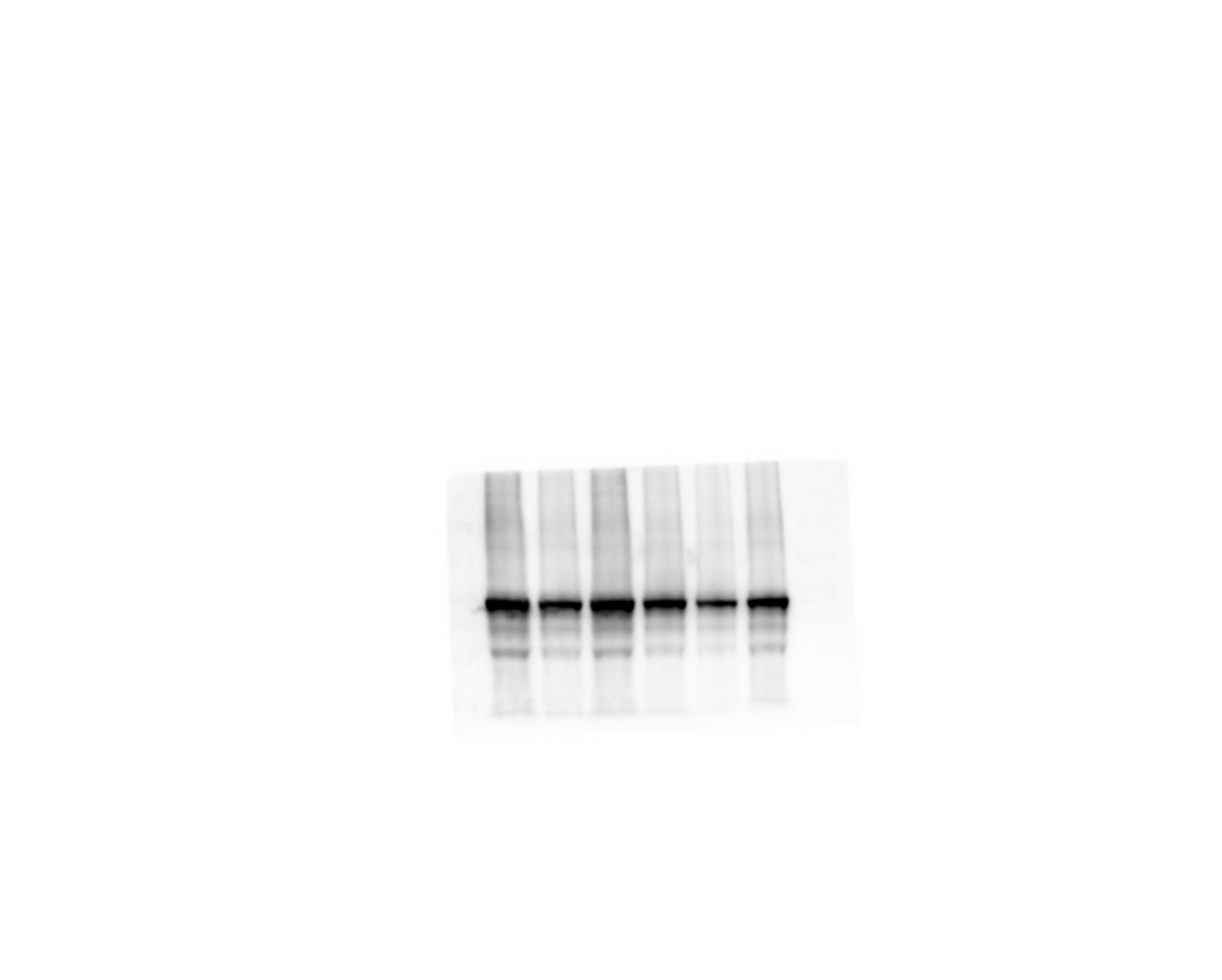

Supplement: Supplementary file 1 [file biomolecules-16-00868-s001.zip › FigureS1 the full, uncropped western blot images/The vivo mice study/HSP60/5.Tif]

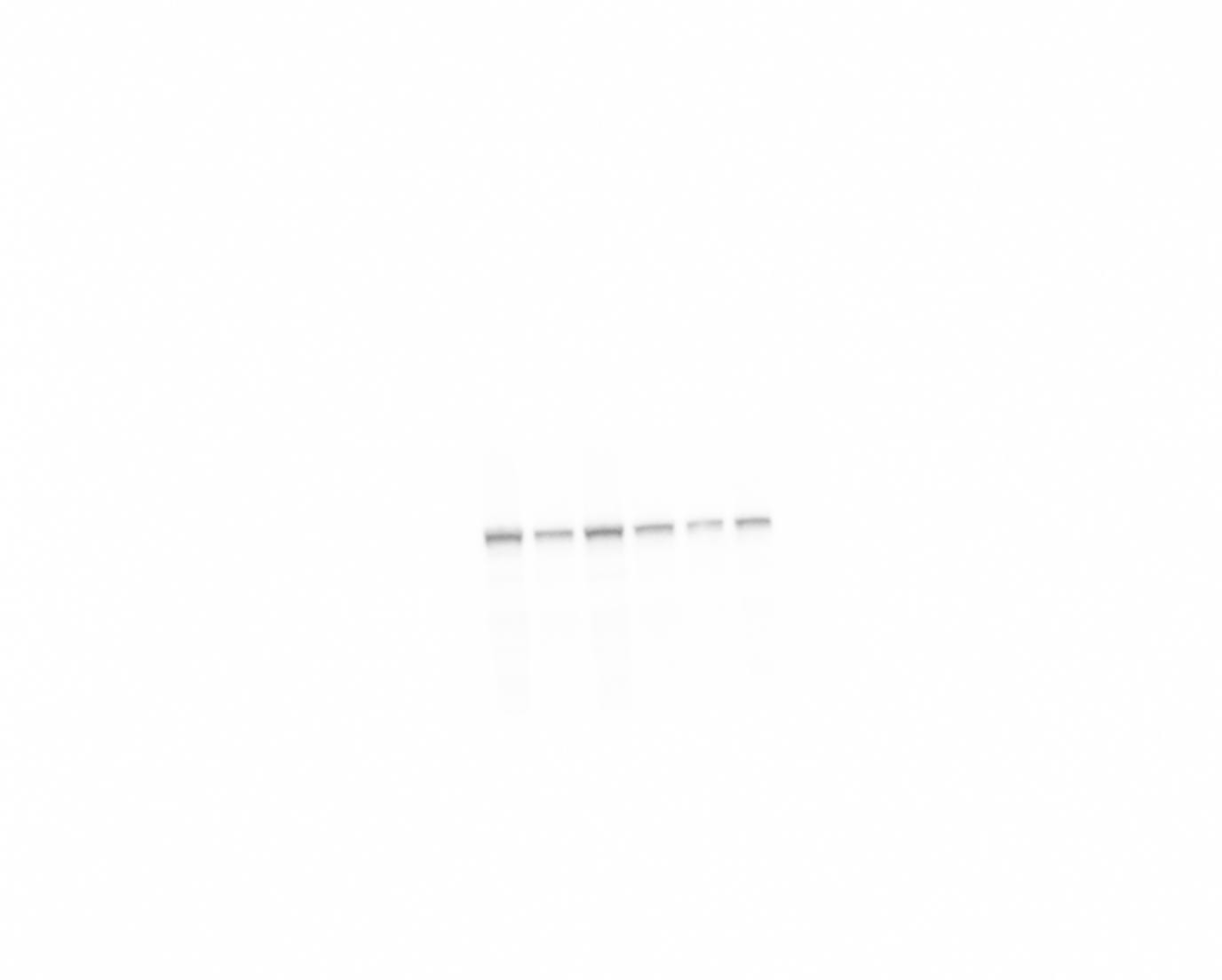

Supplement: Supplementary file 1 [file biomolecules-16-00868-s001.zip › FigureS1 the full, uncropped western blot images/The vivo mice study/LONP1/1-0.4s.Tif]

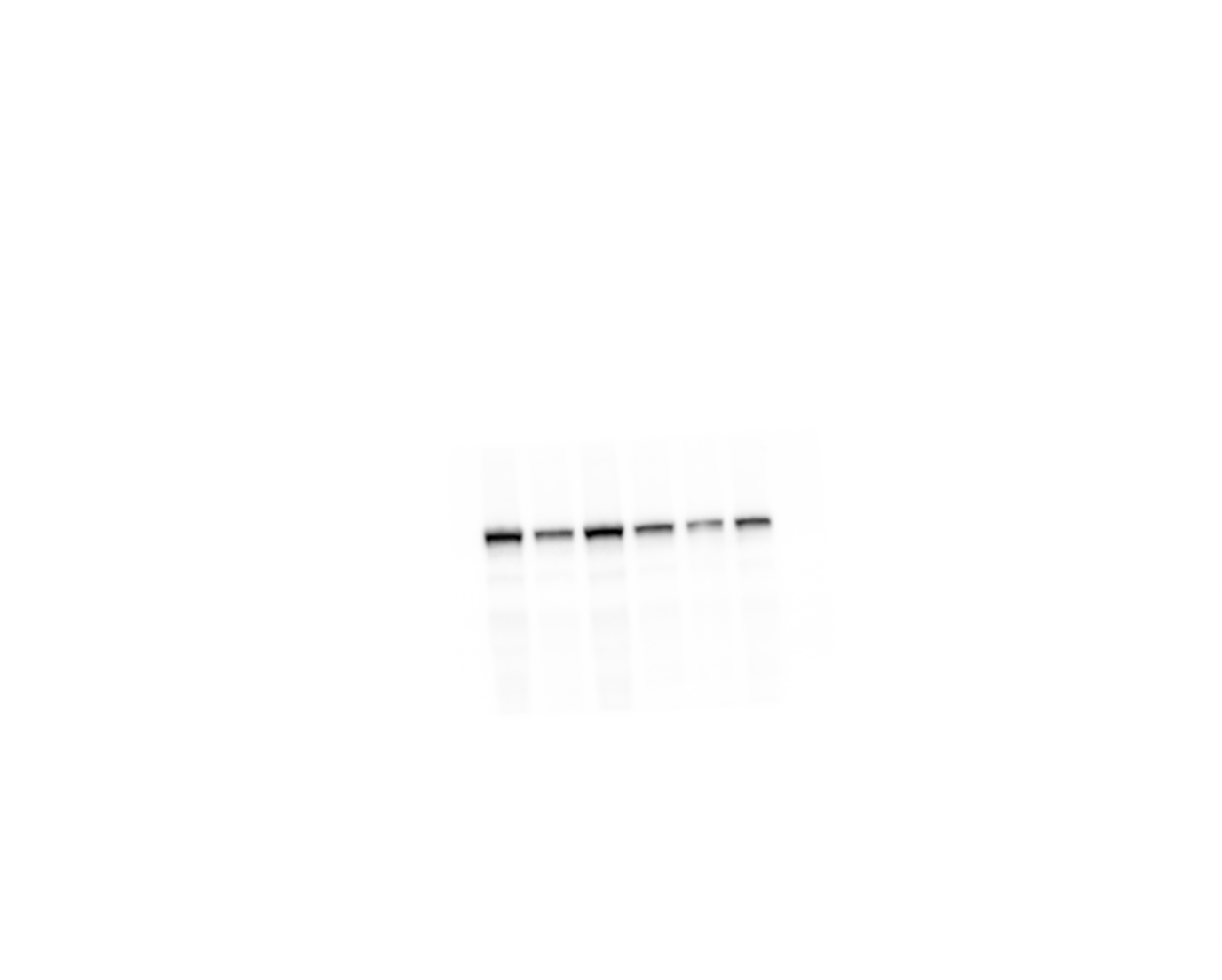

Supplement: Supplementary file 1 [file biomolecules-16-00868-s001.zip › FigureS1 the full, uncropped western blot images/The vivo mice study/LONP1/1-3s.Tif]

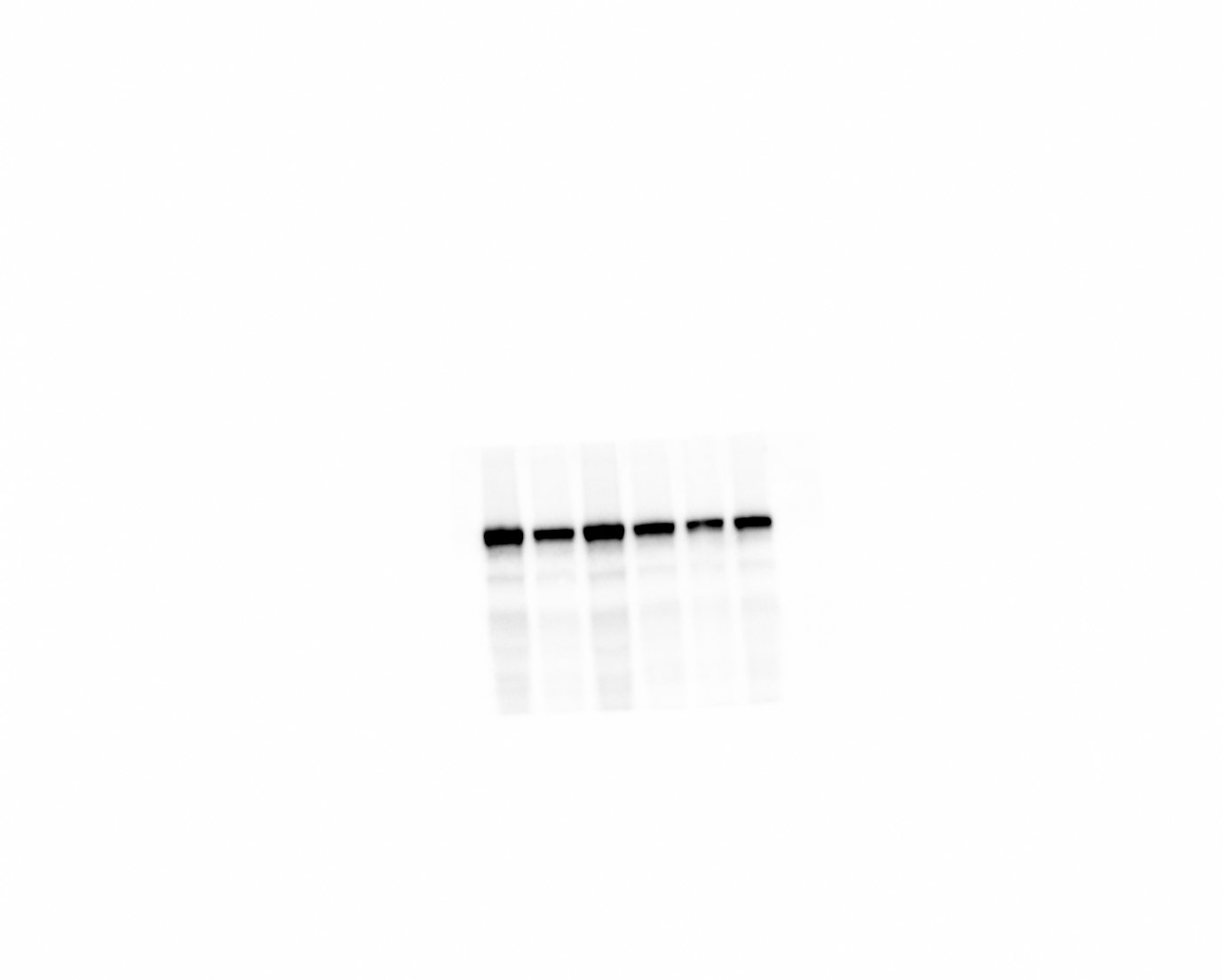

Supplement: Supplementary file 1 [file biomolecules-16-00868-s001.zip › FigureS1 the full, uncropped western blot images/The vivo mice study/LONP1/1-8s.Tif]

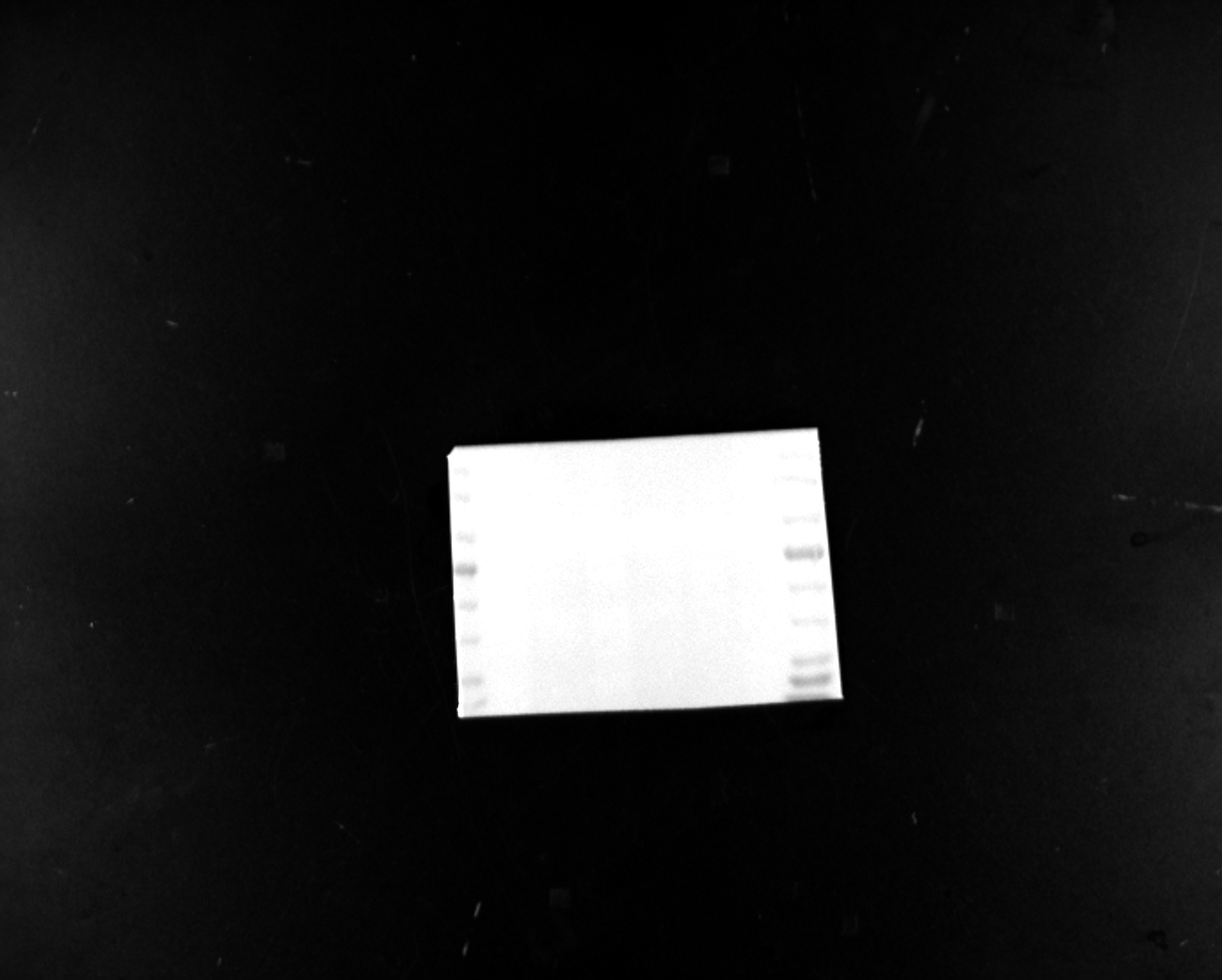

Supplement: Supplementary file 1 [file biomolecules-16-00868-s001.zip › FigureS1 the full, uncropped western blot images/The vivo mice study/LONP1/1-t.Tif]

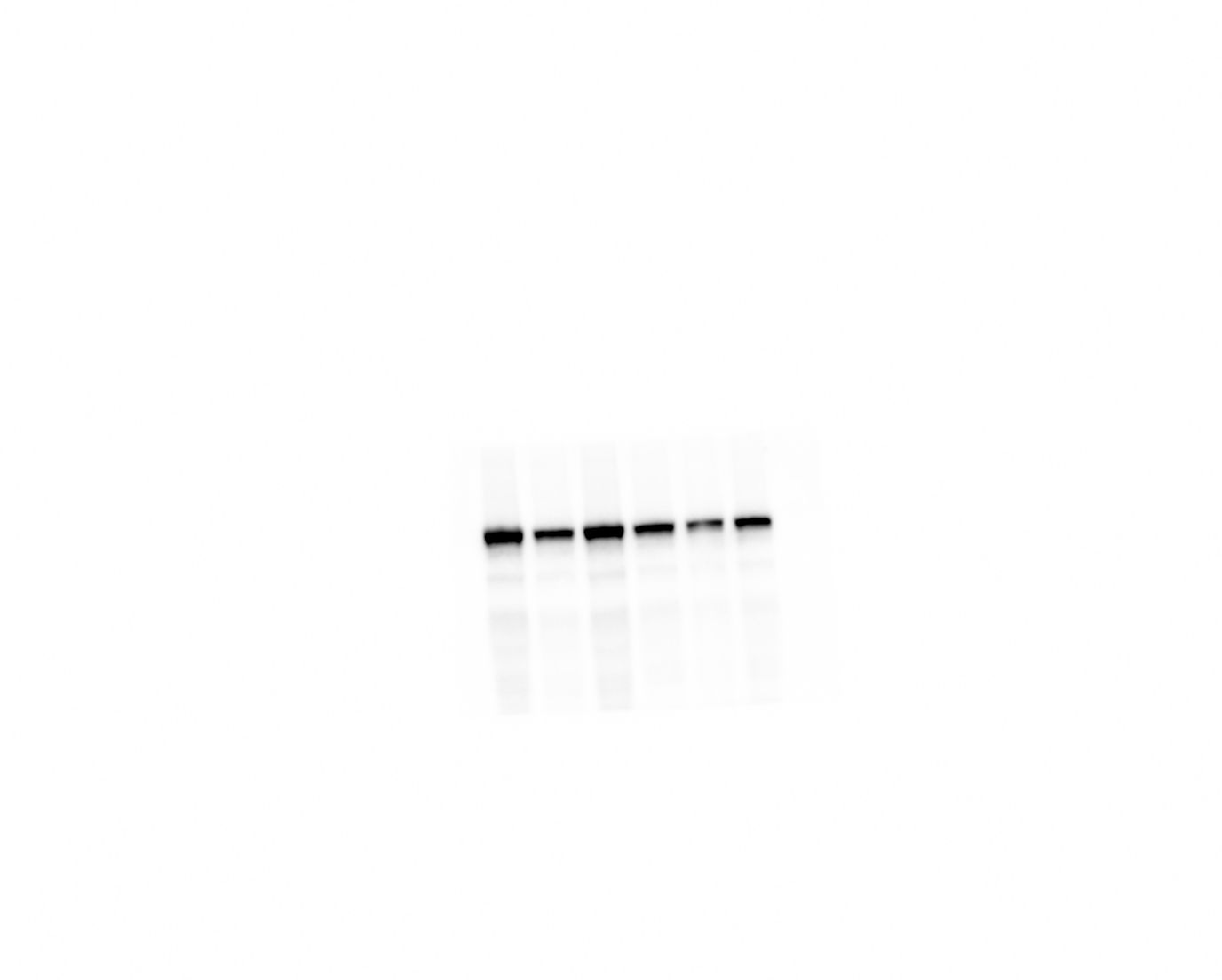

Supplement: Supplementary file 1 [file biomolecules-16-00868-s001.zip › FigureS1 the full, uncropped western blot images/The vivo mice study/LONP1/1.Tif]

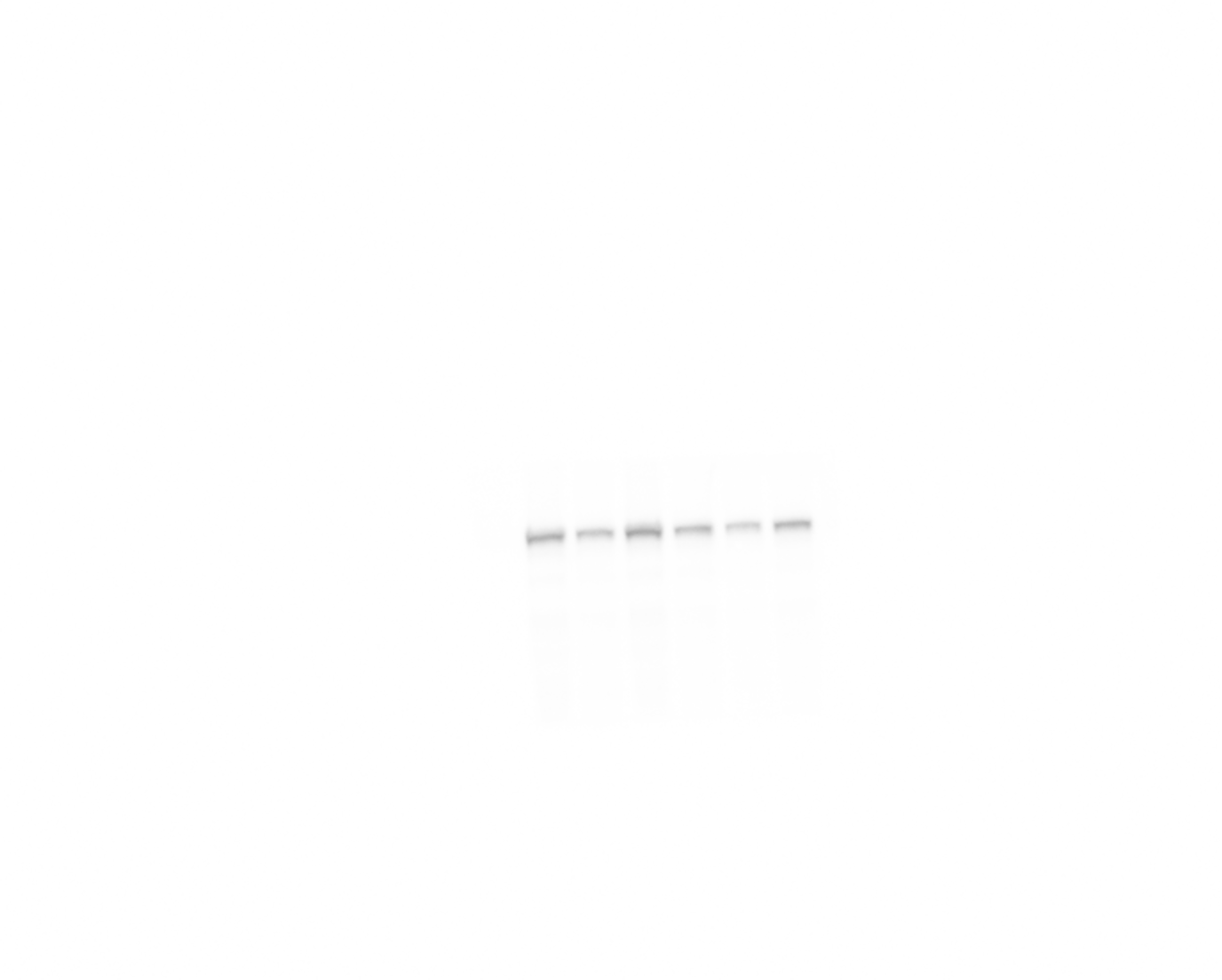

Supplement: Supplementary file 1 [file biomolecules-16-00868-s001.zip › FigureS1 the full, uncropped western blot images/The vivo mice study/LONP1/2-0.8s.Tif]

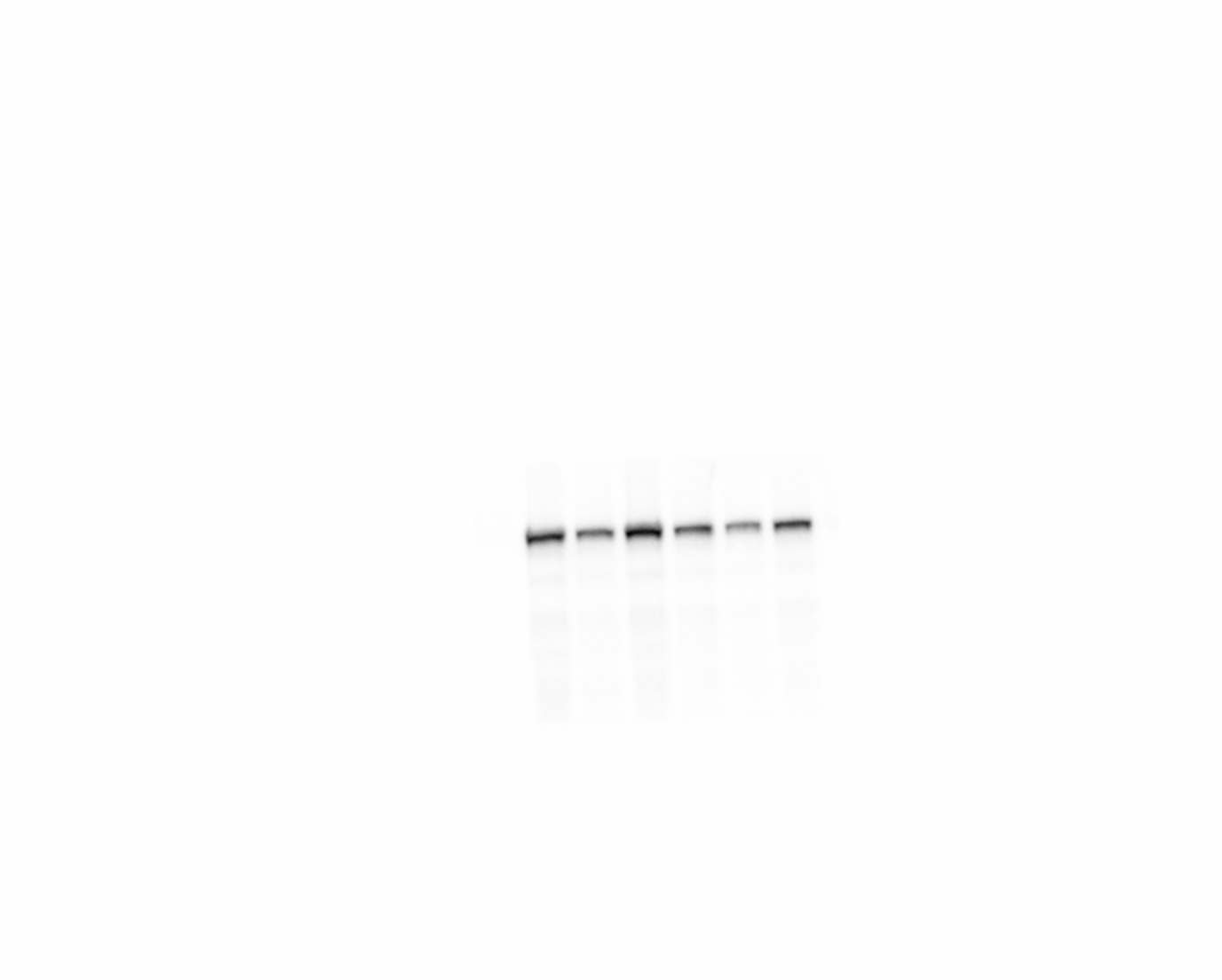

Supplement: Supplementary file 1 [file biomolecules-16-00868-s001.zip › FigureS1 the full, uncropped western blot images/The vivo mice study/LONP1/2-3s.Tif]

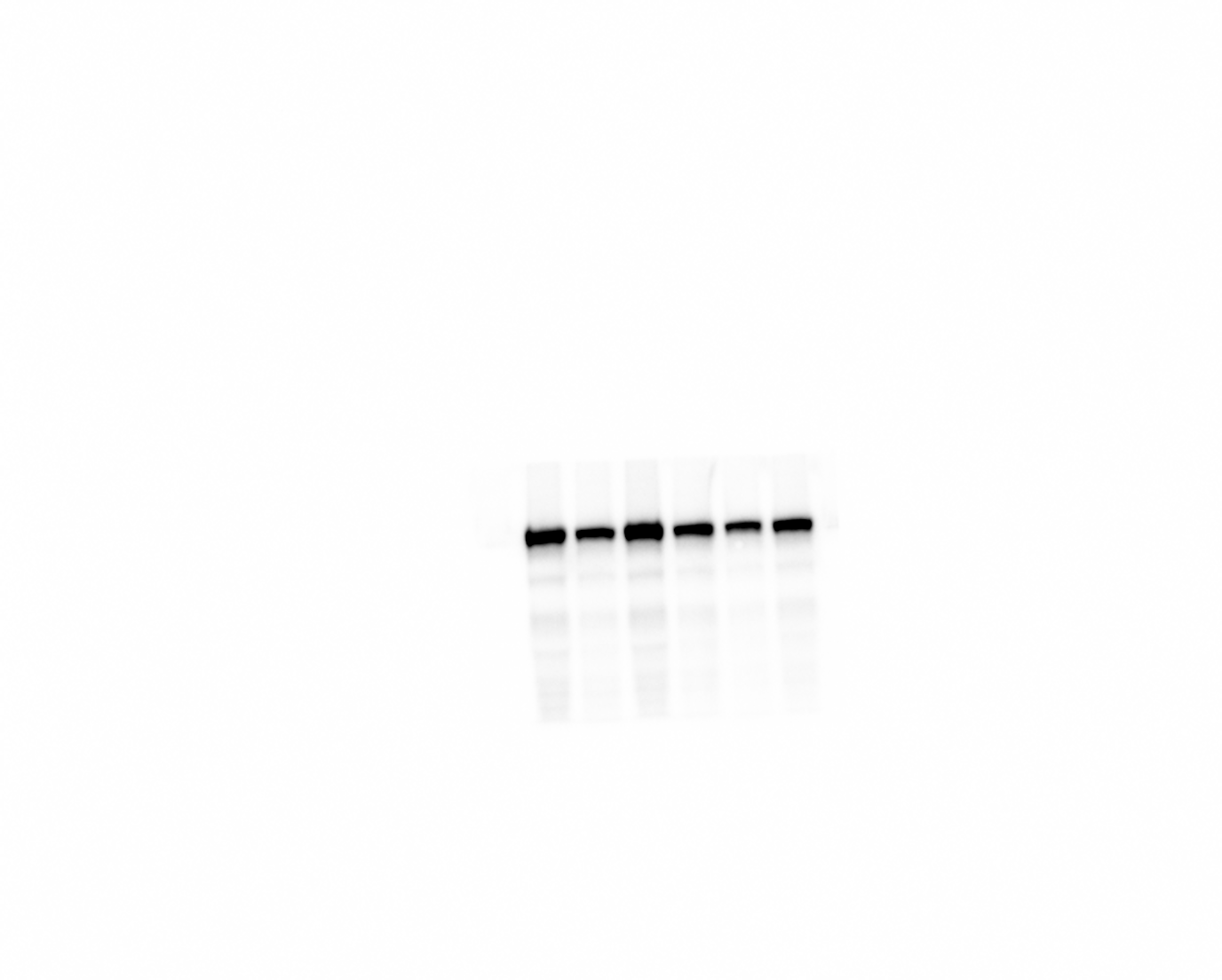

Supplement: Supplementary file 1 [file biomolecules-16-00868-s001.zip › FigureS1 the full, uncropped western blot images/The vivo mice study/LONP1/2-6s.Tif]

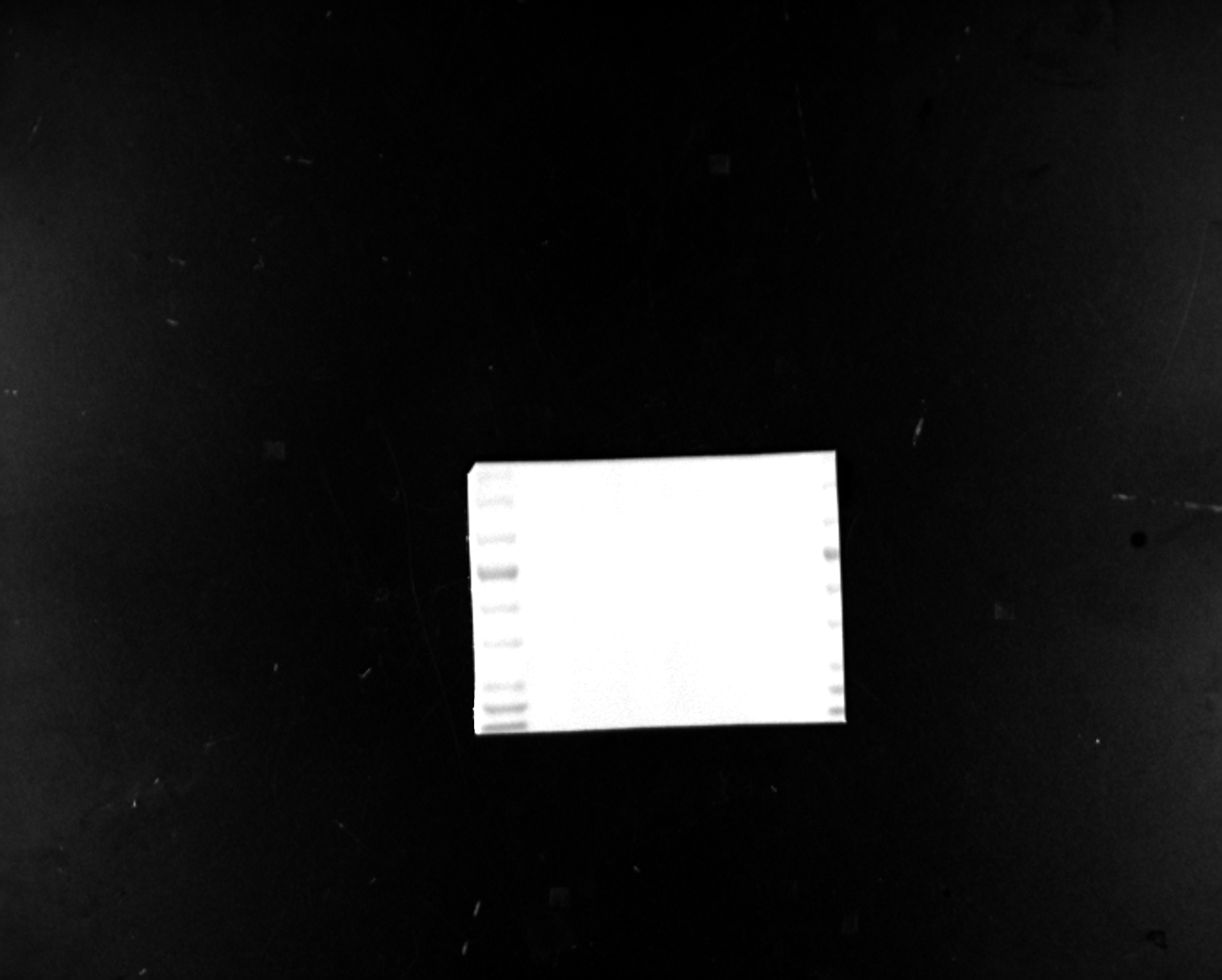

Supplement: Supplementary file 1 [file biomolecules-16-00868-s001.zip › FigureS1 the full, uncropped western blot images/The vivo mice study/LONP1/2-t.Tif]

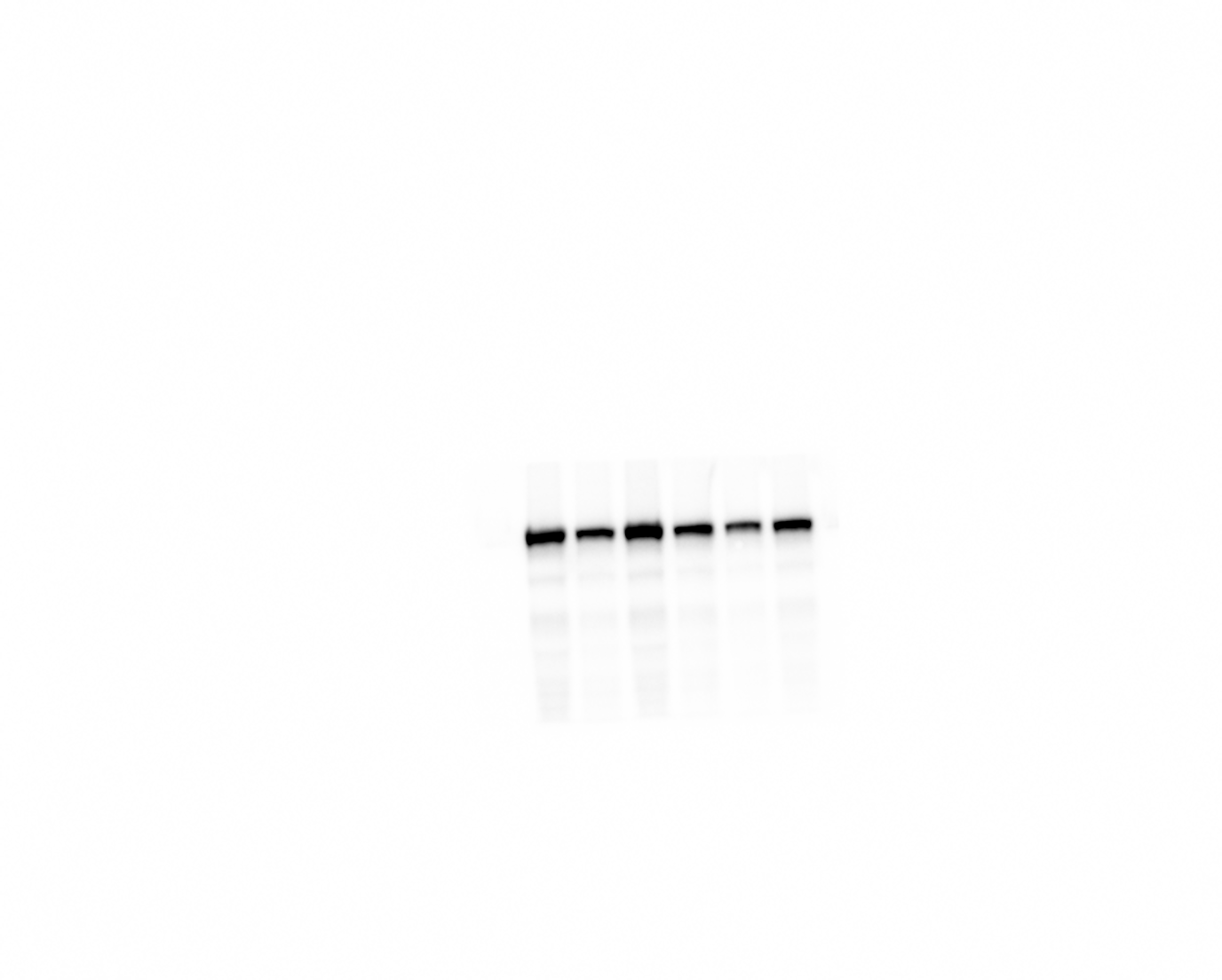

Supplement: Supplementary file 1 [file biomolecules-16-00868-s001.zip › FigureS1 the full, uncropped western blot images/The vivo mice study/LONP1/2.Tif]

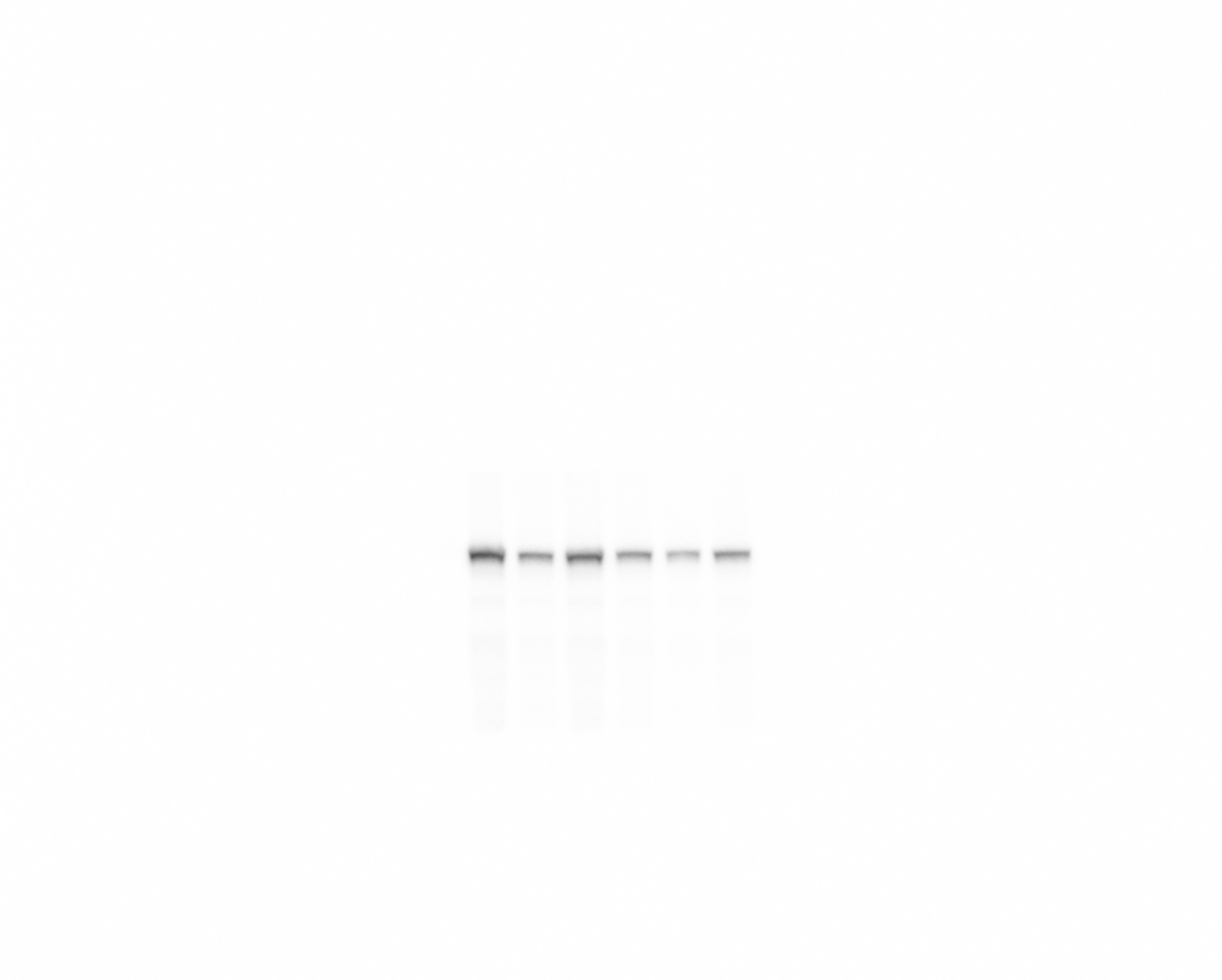

Supplement: Supplementary file 1 [file biomolecules-16-00868-s001.zip › FigureS1 the full, uncropped western blot images/The vivo mice study/LONP1/3-0.6s.Tif]

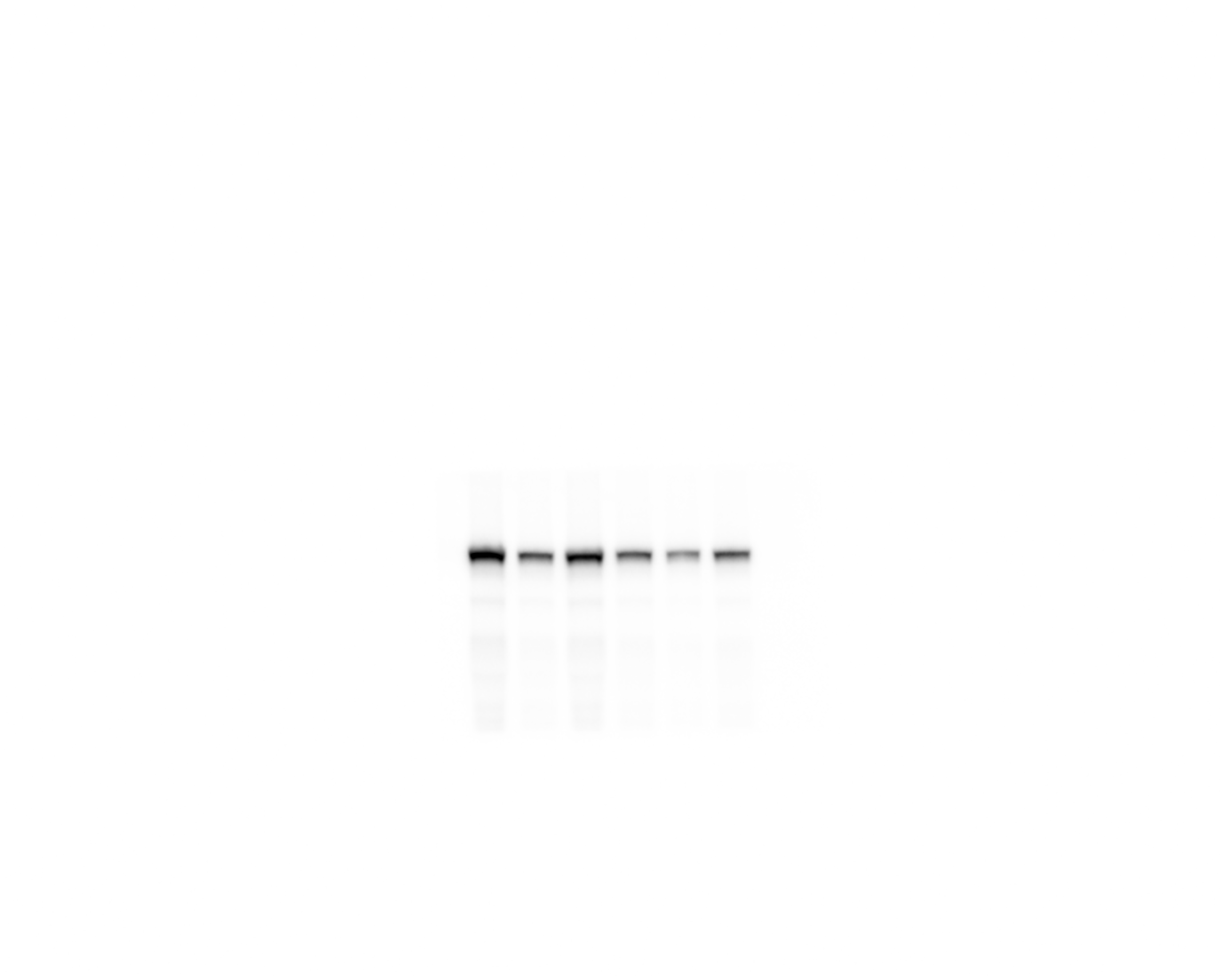

Supplement: Supplementary file 1 [file biomolecules-16-00868-s001.zip › FigureS1 the full, uncropped western blot images/The vivo mice study/LONP1/3-3s.Tif]

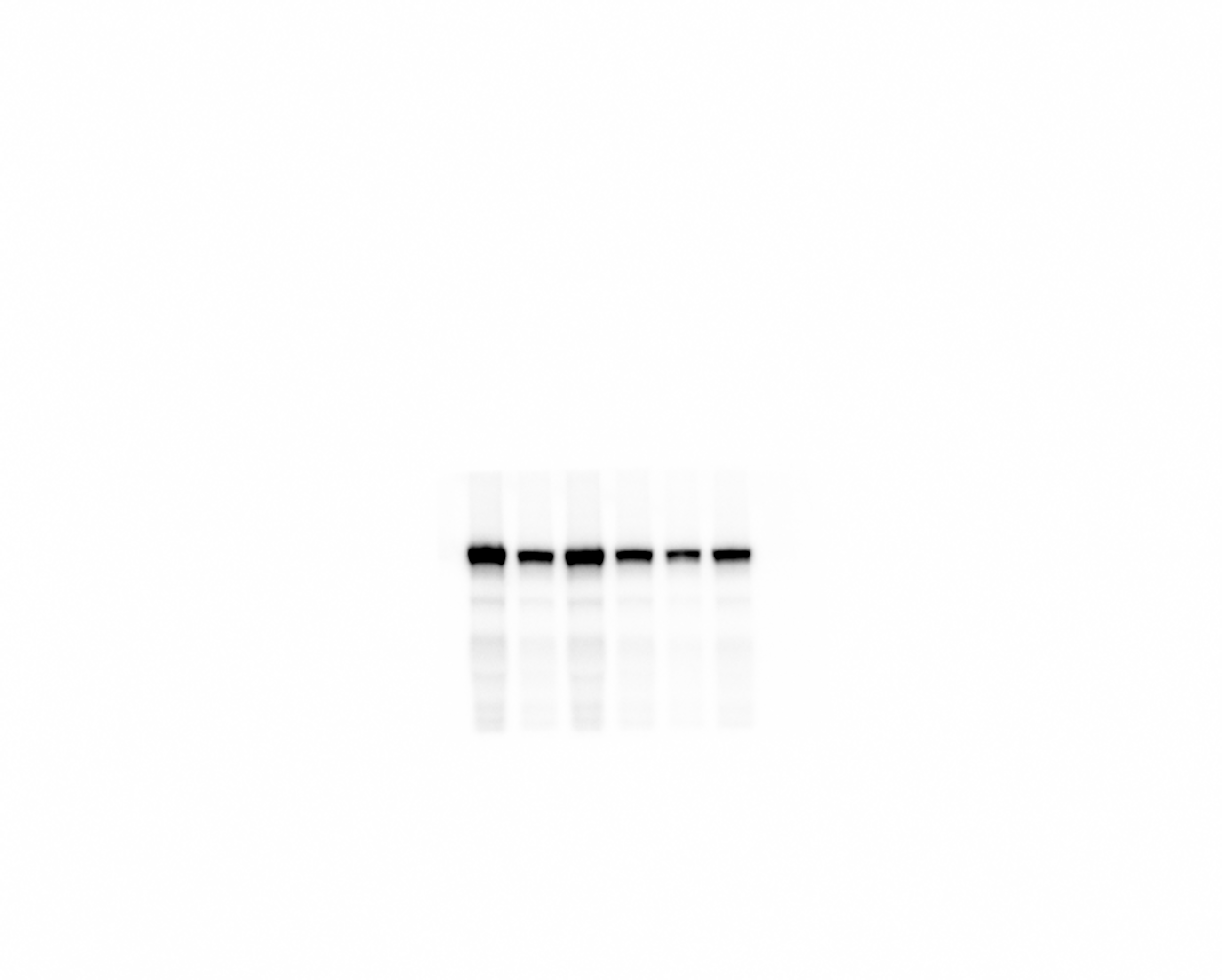

Supplement: Supplementary file 1 [file biomolecules-16-00868-s001.zip › FigureS1 the full, uncropped western blot images/The vivo mice study/LONP1/3-7s.Tif]

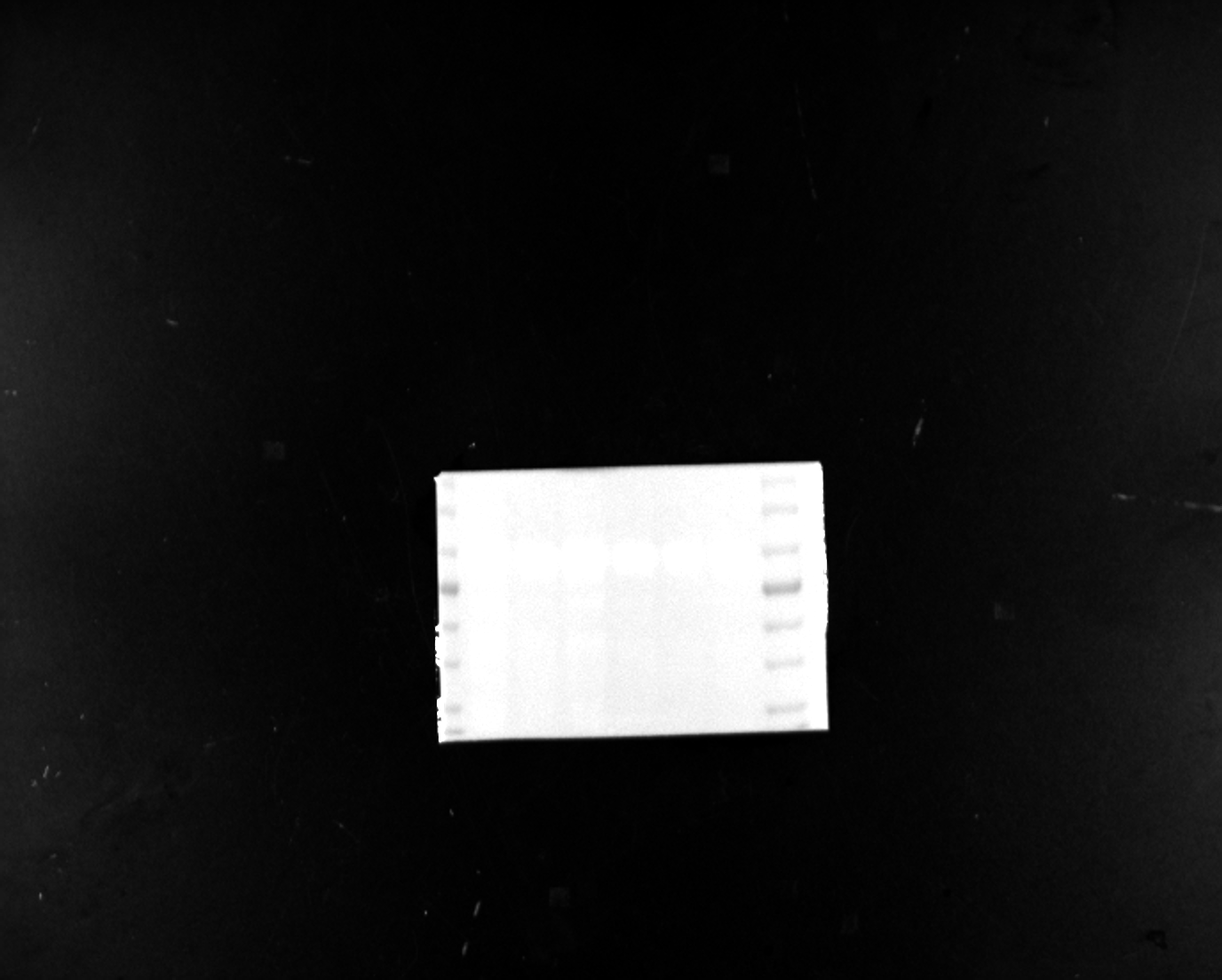

Supplement: Supplementary file 1 [file biomolecules-16-00868-s001.zip › FigureS1 the full, uncropped western blot images/The vivo mice study/LONP1/3-t.Tif]

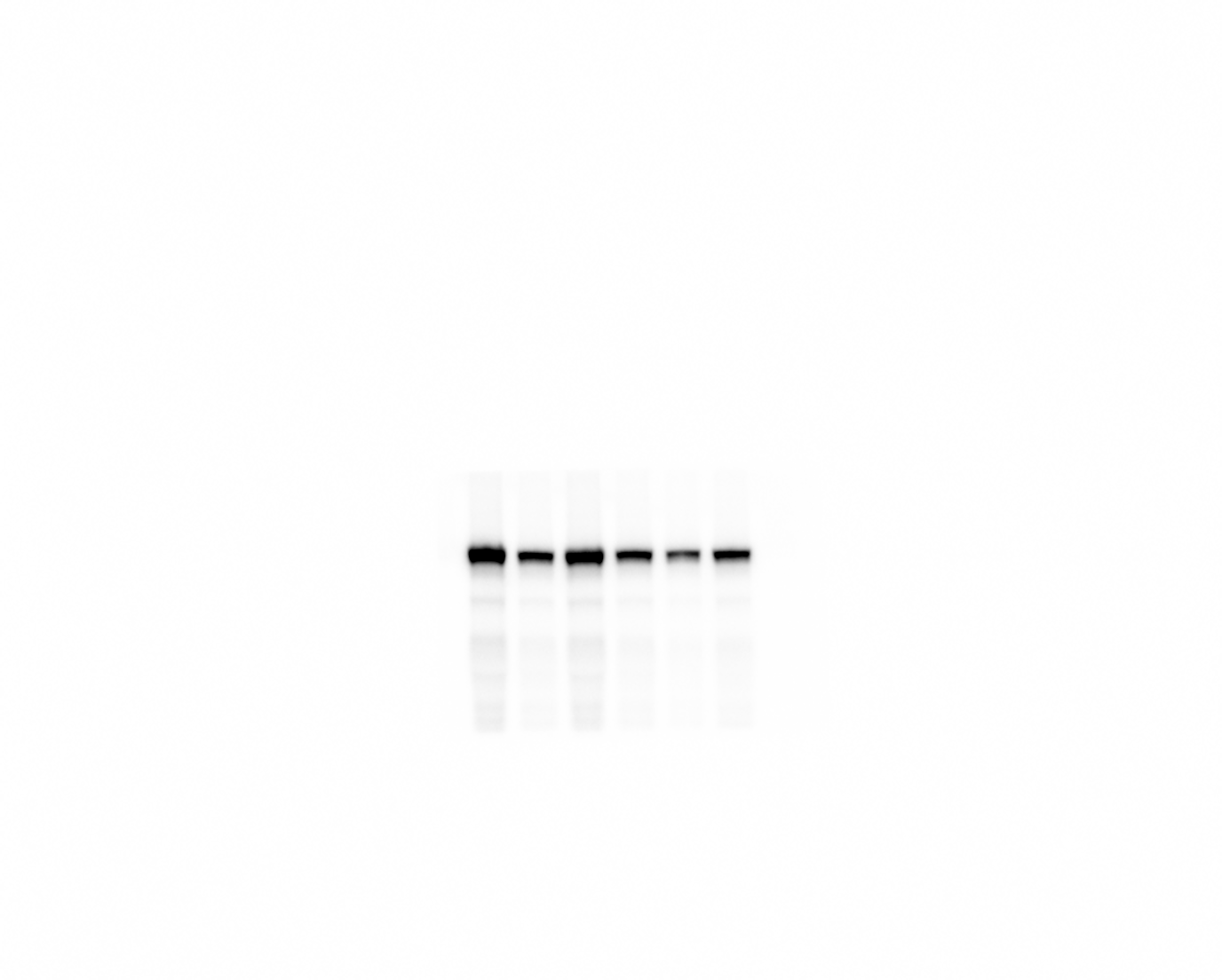

Supplement: Supplementary file 1 [file biomolecules-16-00868-s001.zip › FigureS1 the full, uncropped western blot images/The vivo mice study/LONP1/3.Tif]

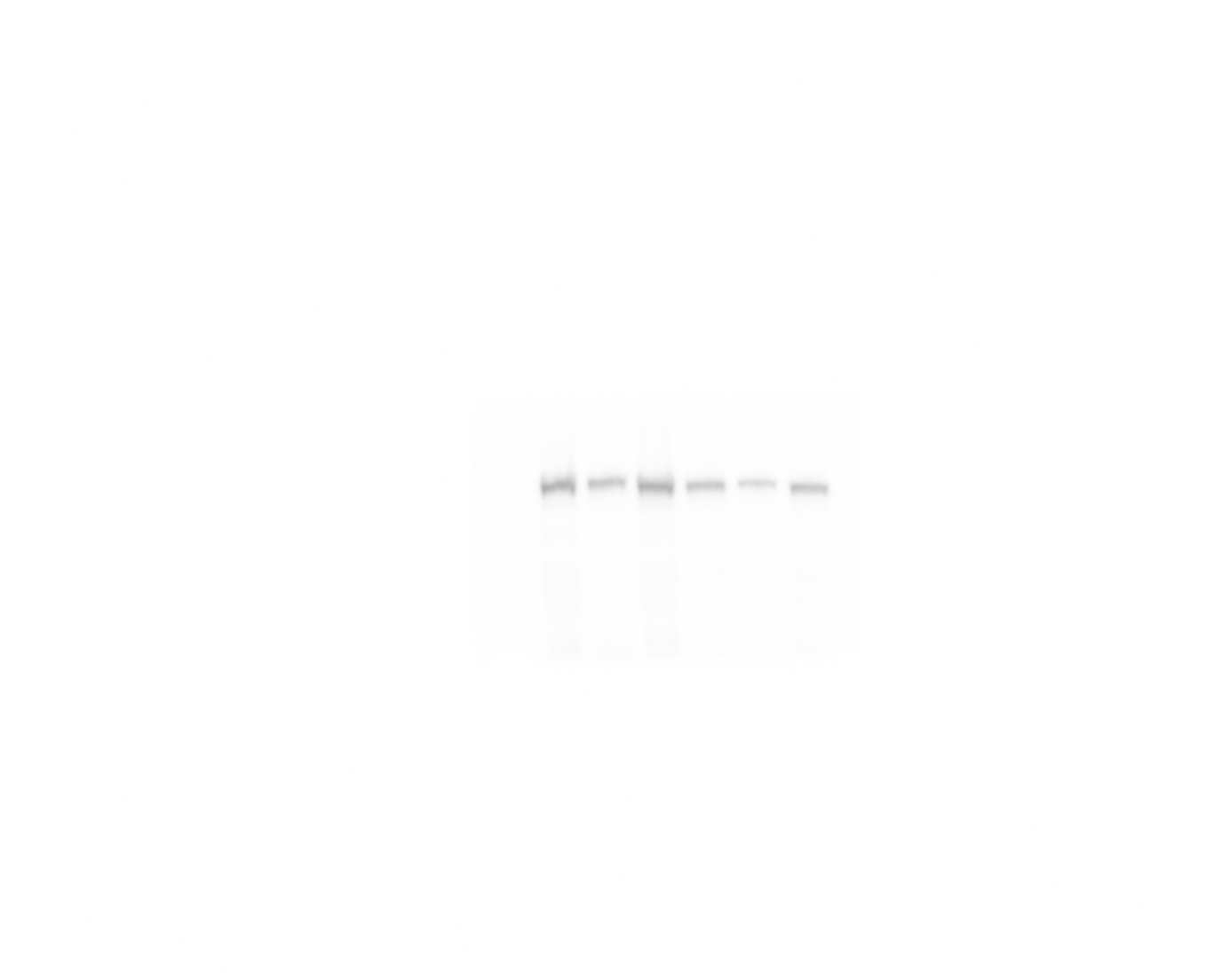

Supplement: Supplementary file 1 [file biomolecules-16-00868-s001.zip › FigureS1 the full, uncropped western blot images/The vivo mice study/LONP1/4-0.4s.Tif]

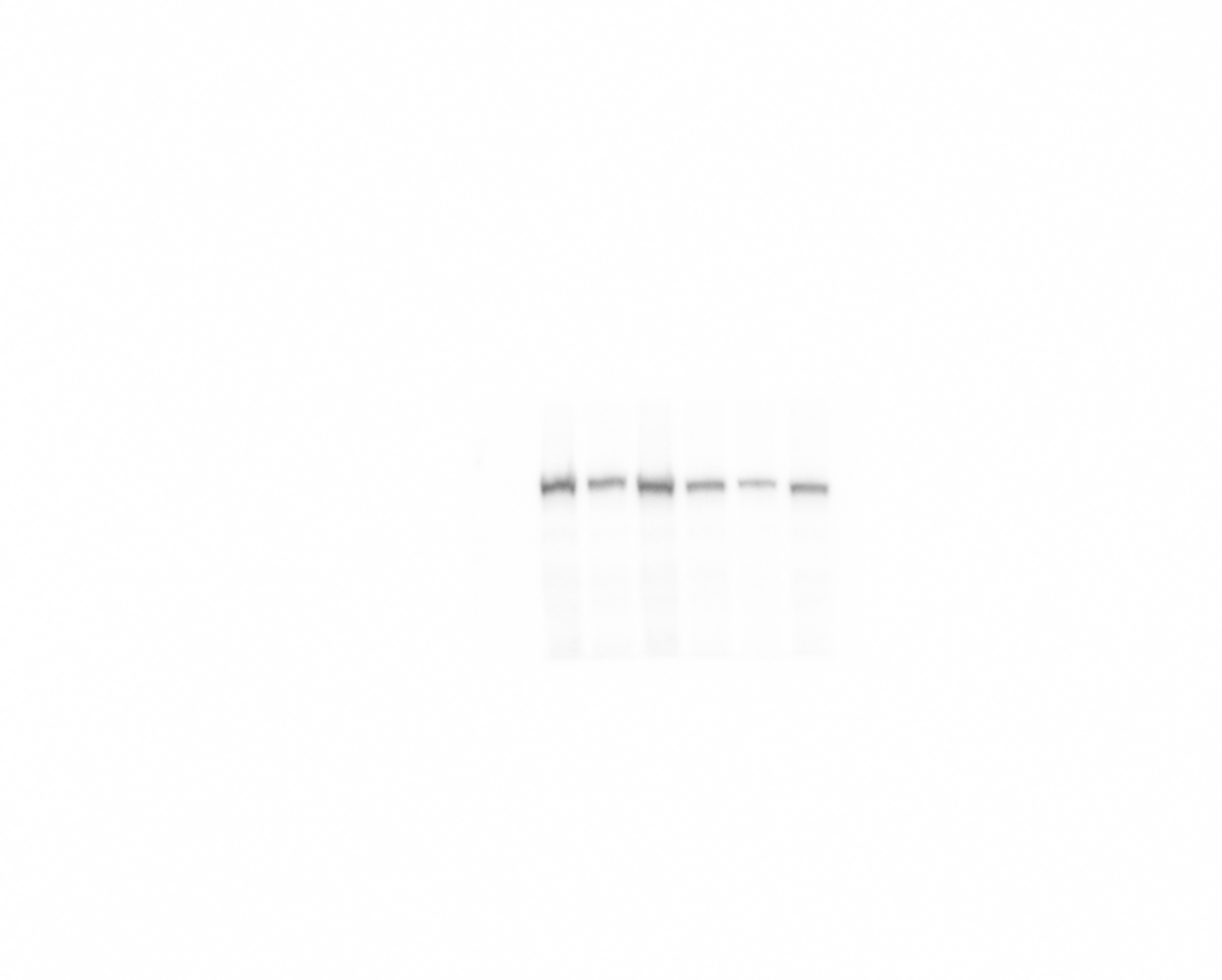

Supplement: Supplementary file 1 [file biomolecules-16-00868-s001.zip › FigureS1 the full, uncropped western blot images/The vivo mice study/LONP1/4-1.1s.Tif]

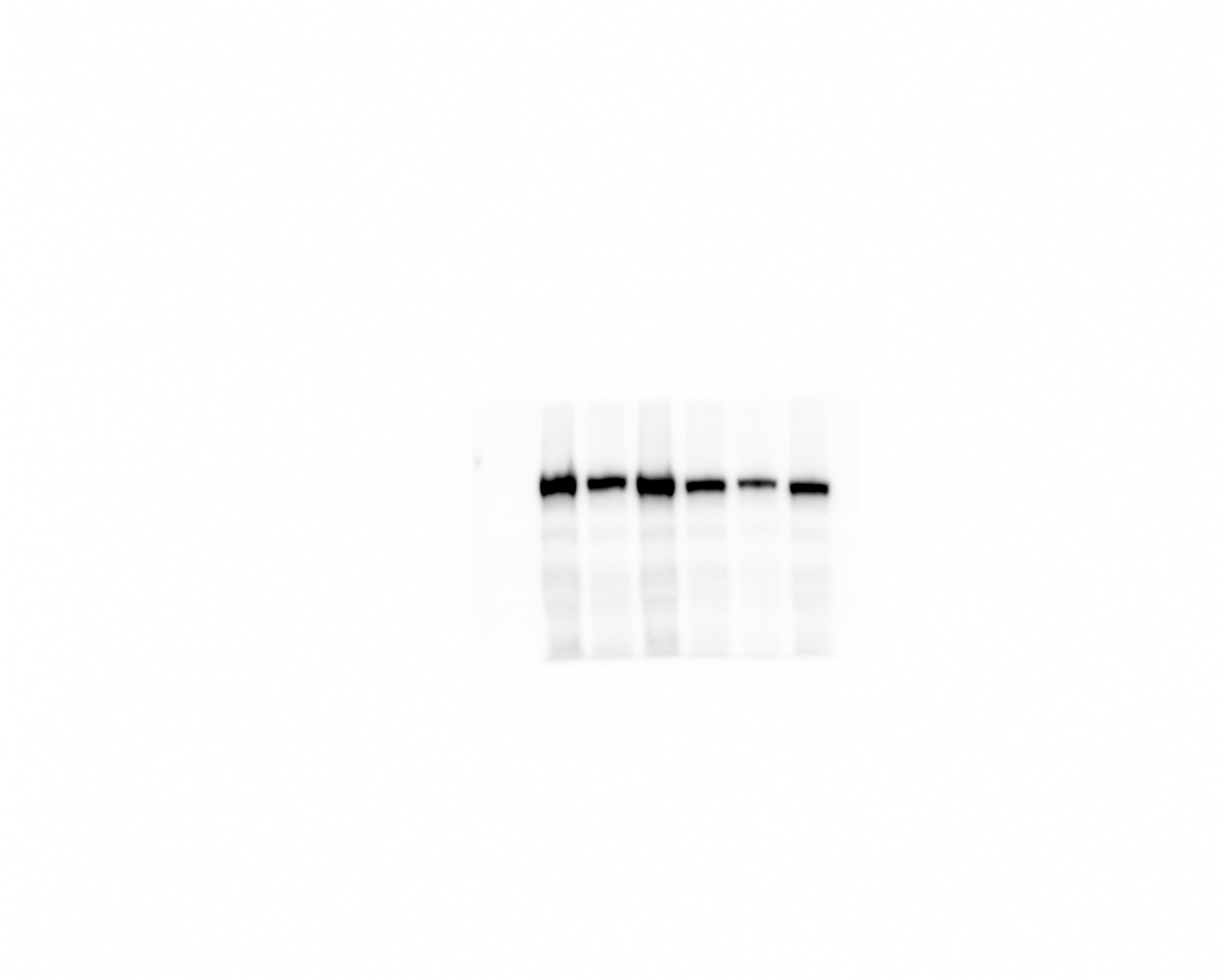

Supplement: Supplementary file 1 [file biomolecules-16-00868-s001.zip › FigureS1 the full, uncropped western blot images/The vivo mice study/LONP1/4-5s.Tif]

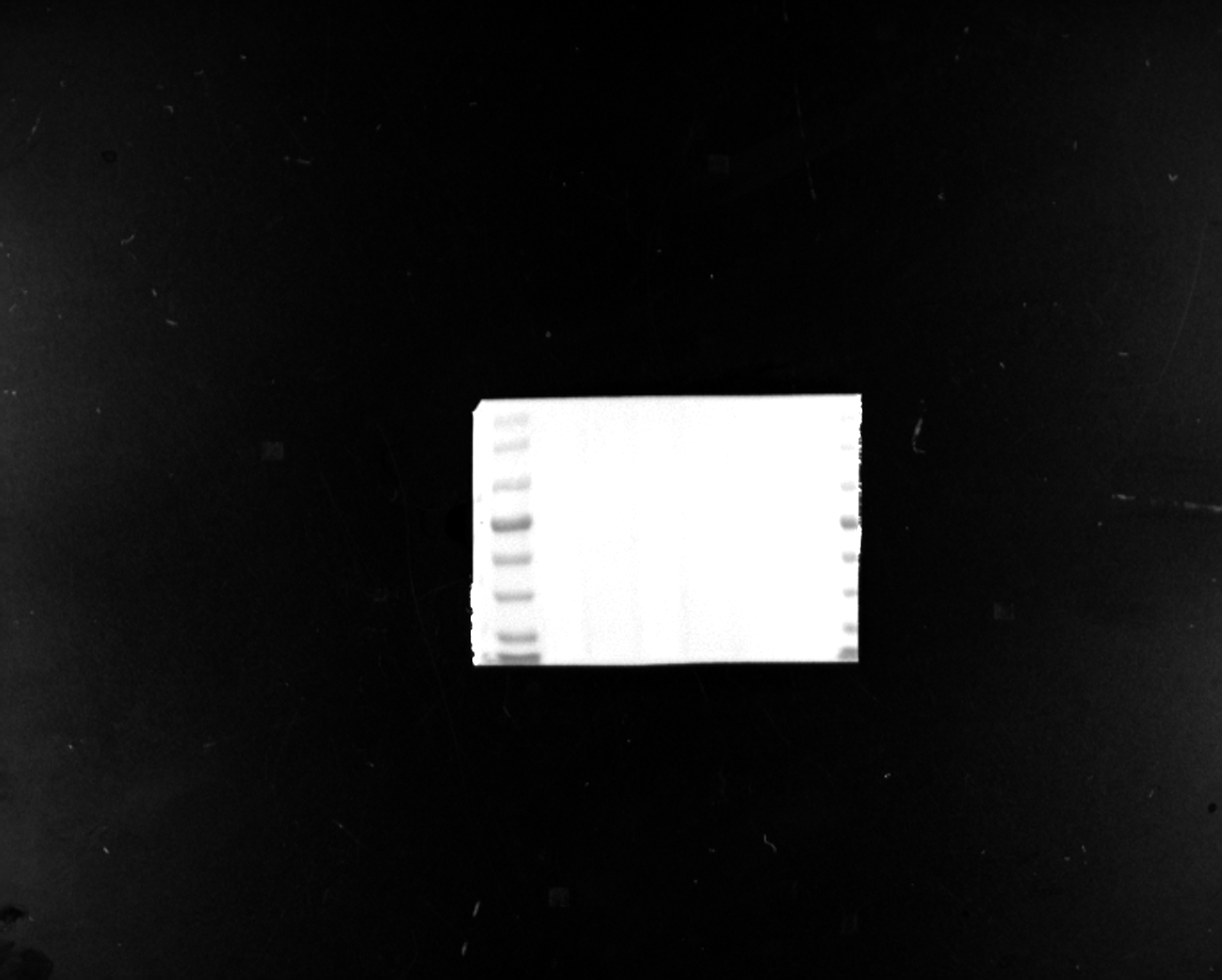

Supplement: Supplementary file 1 [file biomolecules-16-00868-s001.zip › FigureS1 the full, uncropped western blot images/The vivo mice study/LONP1/4-t.Tif]

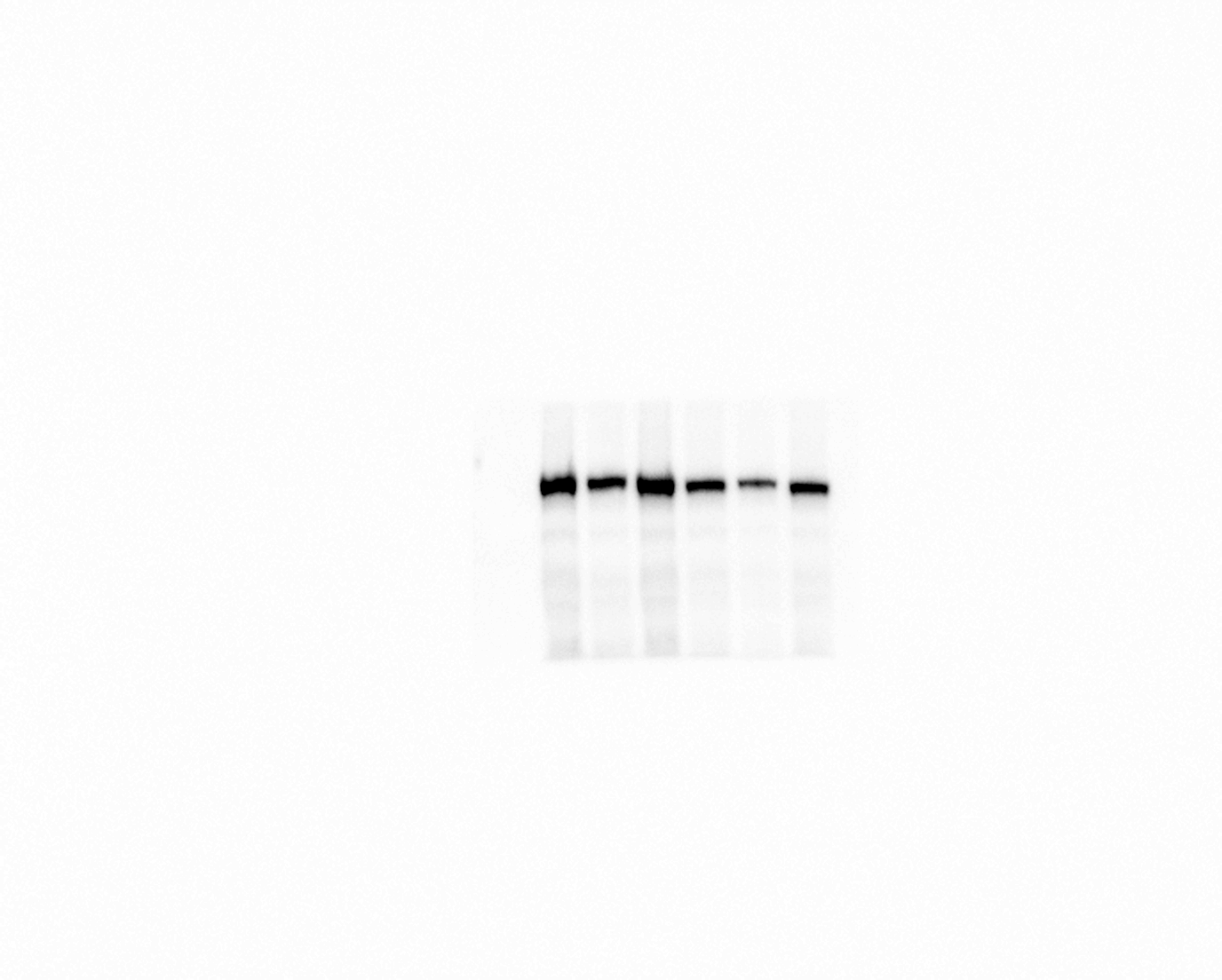

Supplement: Supplementary file 1 [file biomolecules-16-00868-s001.zip › FigureS1 the full, uncropped western blot images/The vivo mice study/LONP1/4.Tif]

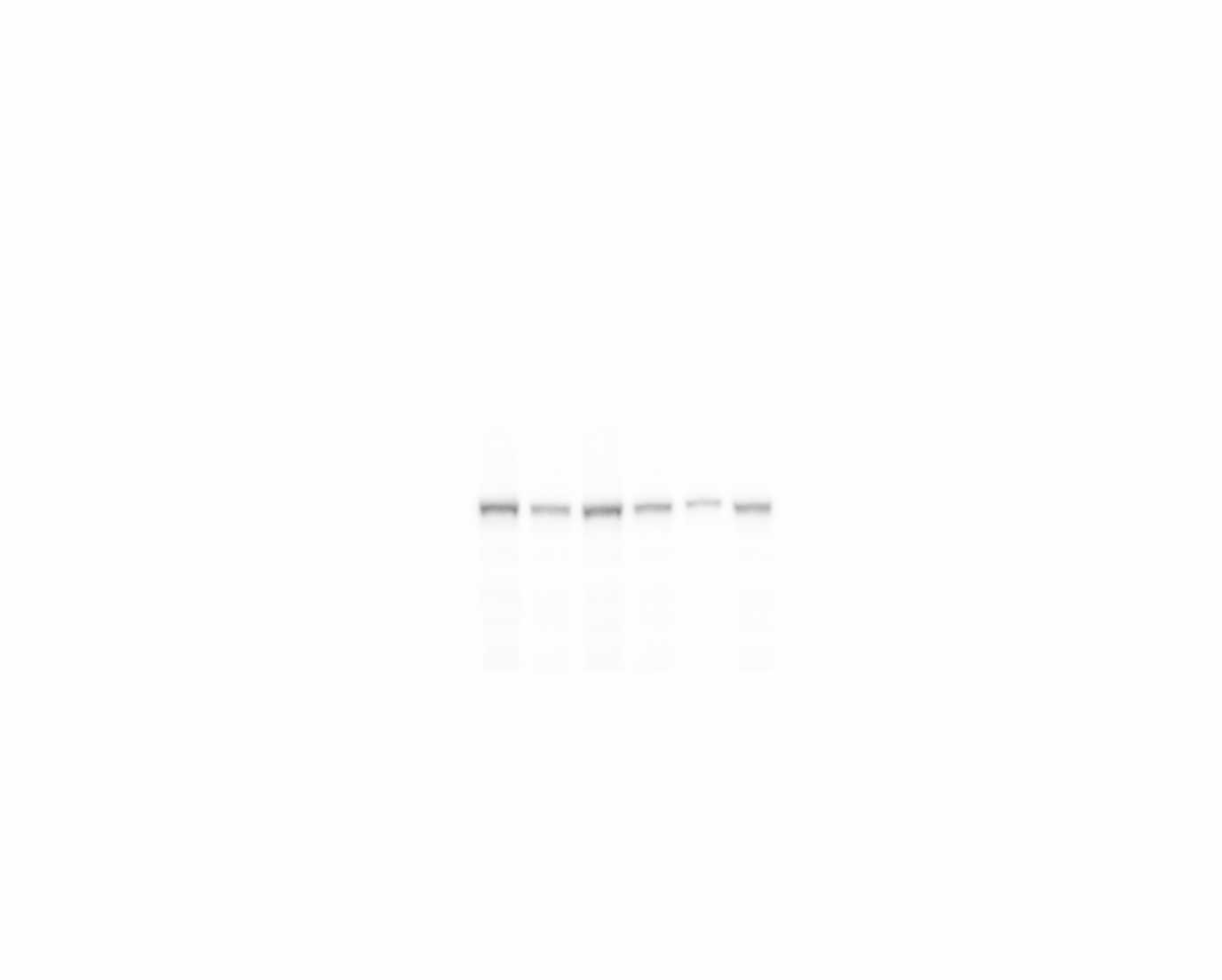

Supplement: Supplementary file 1 [file biomolecules-16-00868-s001.zip › FigureS1 the full, uncropped western blot images/The vivo mice study/LONP1/5-0.4s.Tif]

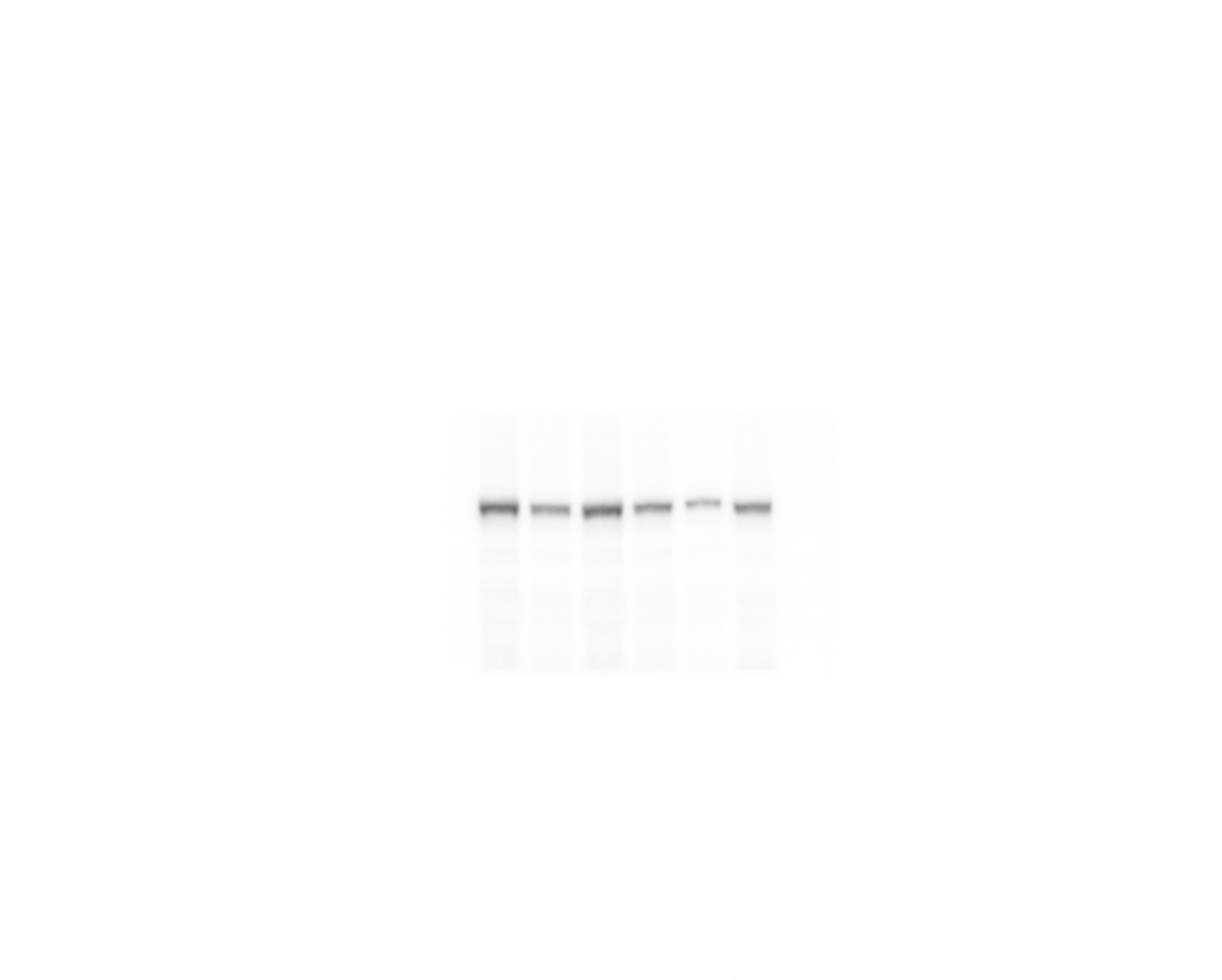

Supplement: Supplementary file 1 [file biomolecules-16-00868-s001.zip › FigureS1 the full, uncropped western blot images/The vivo mice study/LONP1/5-1.1s.Tif]

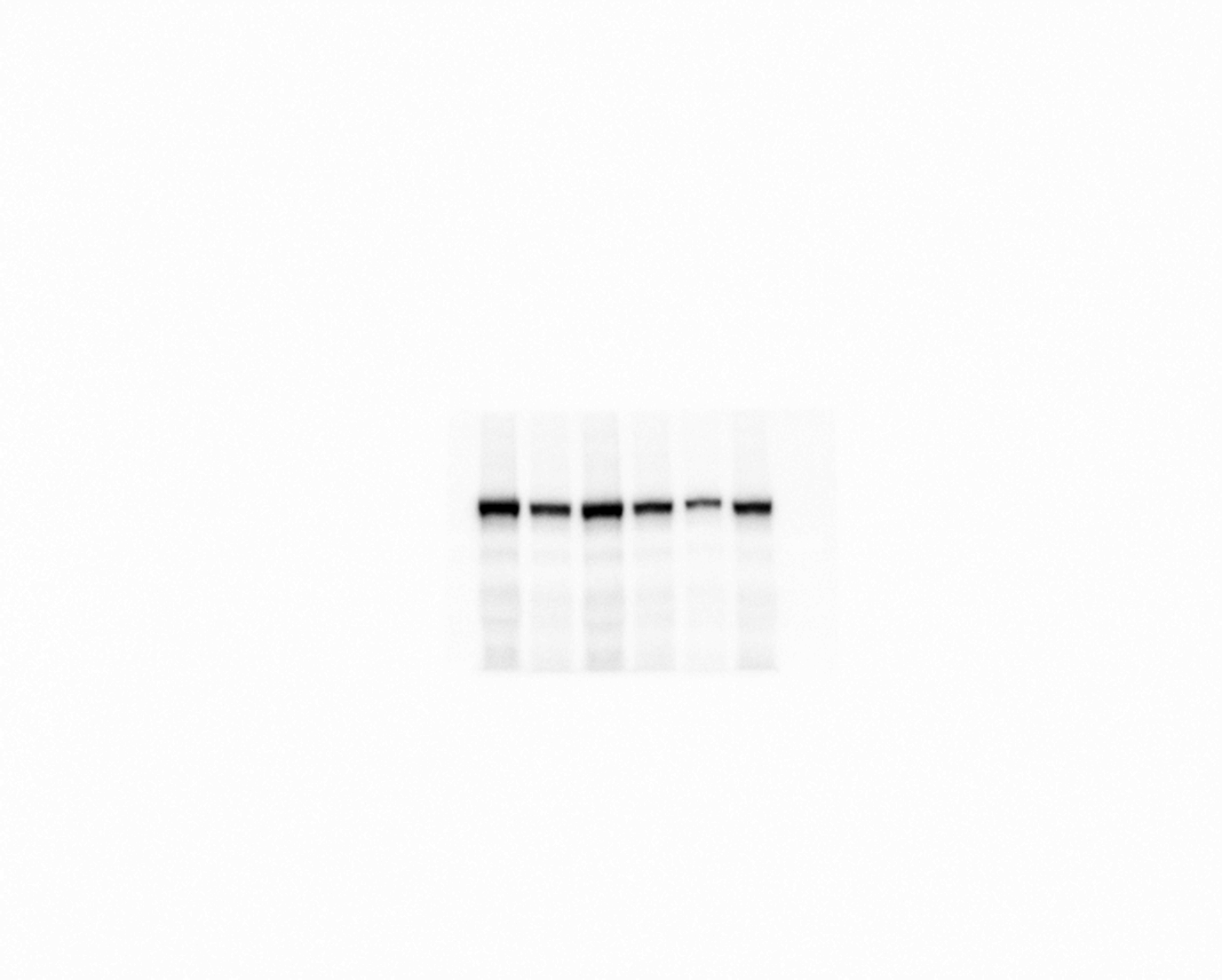

Supplement: Supplementary file 1 [file biomolecules-16-00868-s001.zip › FigureS1 the full, uncropped western blot images/The vivo mice study/LONP1/5-3.5s.Tif]

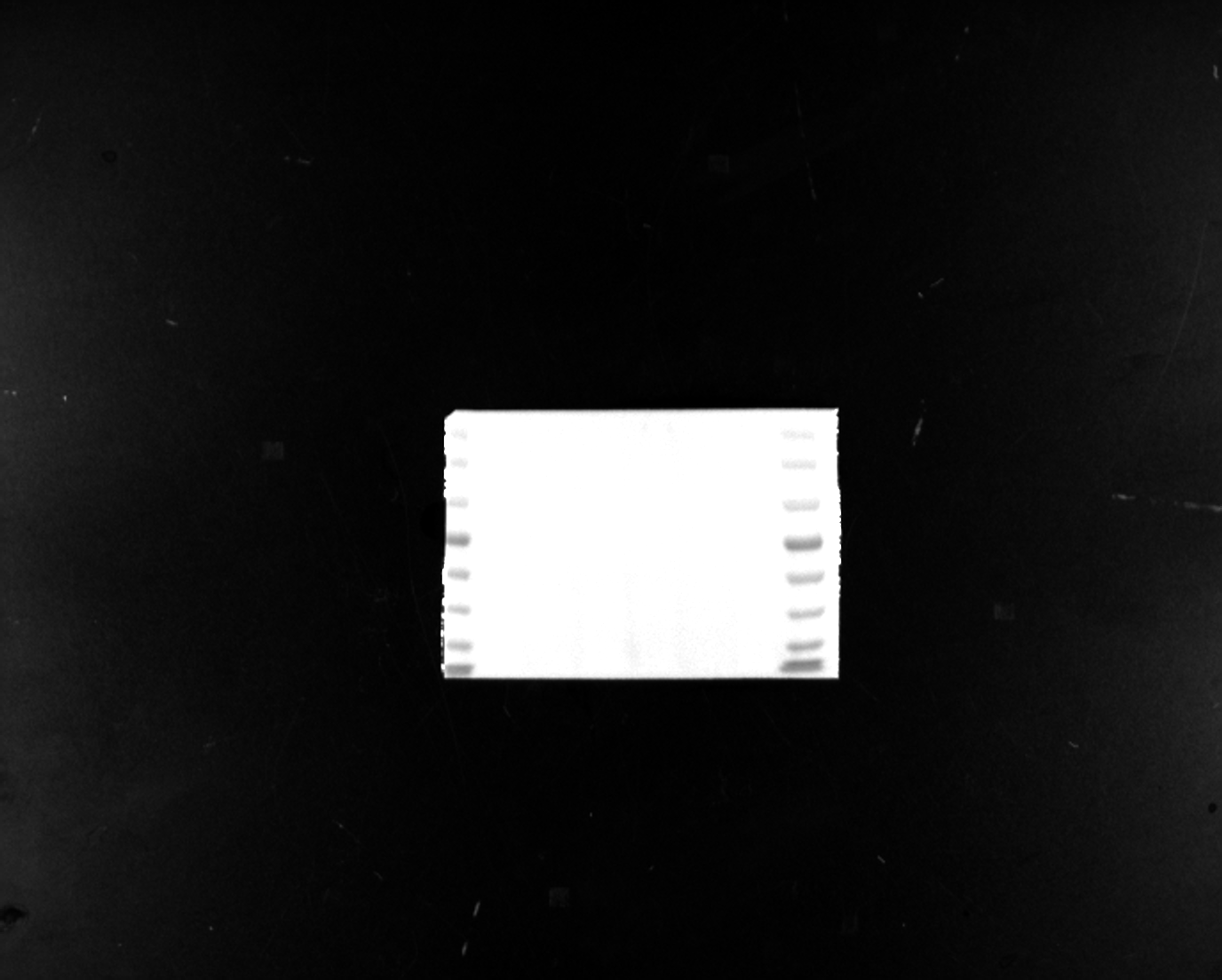

Supplement: Supplementary file 1 [file biomolecules-16-00868-s001.zip › FigureS1 the full, uncropped western blot images/The vivo mice study/LONP1/5-t.Tif]

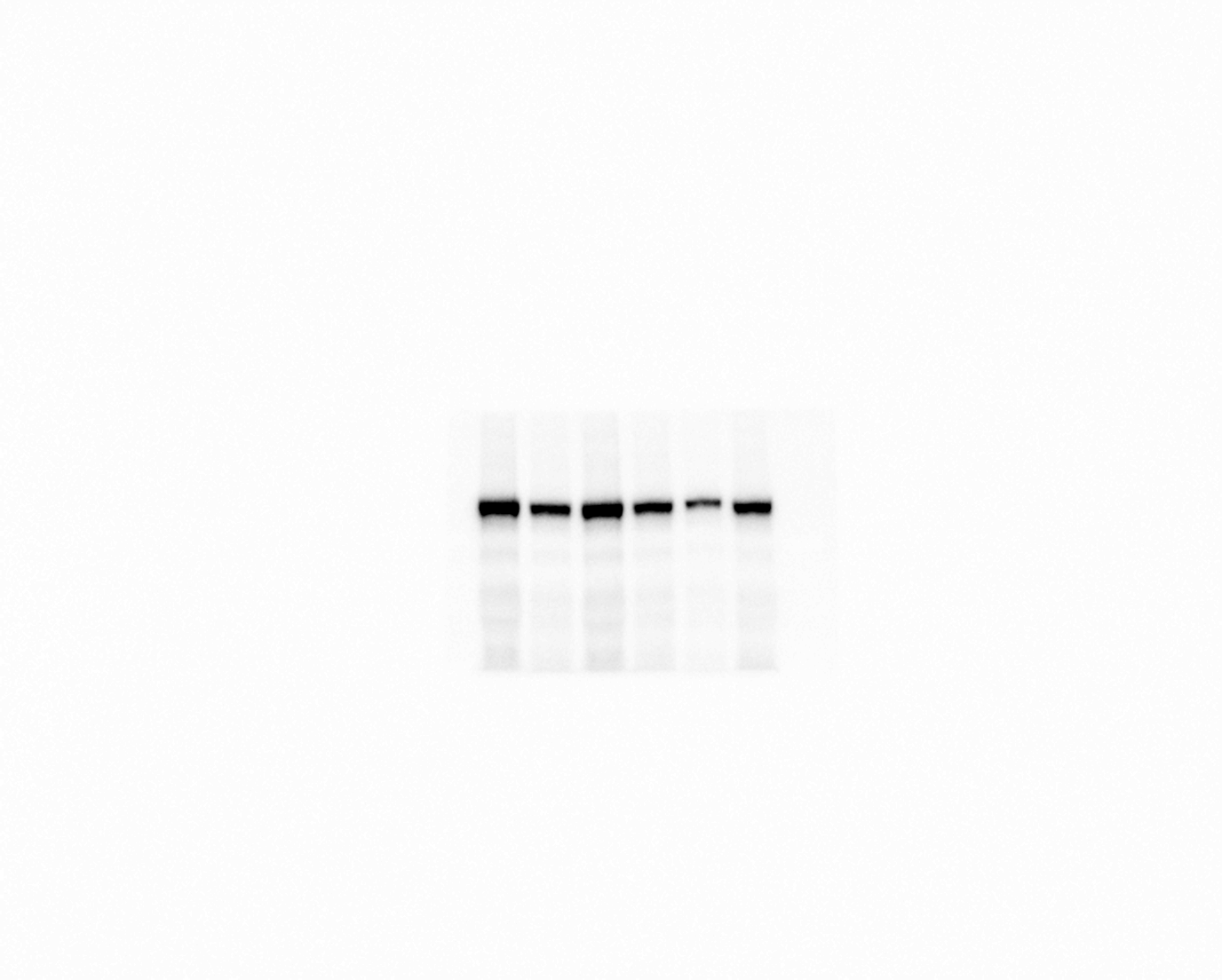

Supplement: Supplementary file 1 [file biomolecules-16-00868-s001.zip › FigureS1 the full, uncropped western blot images/The vivo mice study/LONP1/5.Tif]

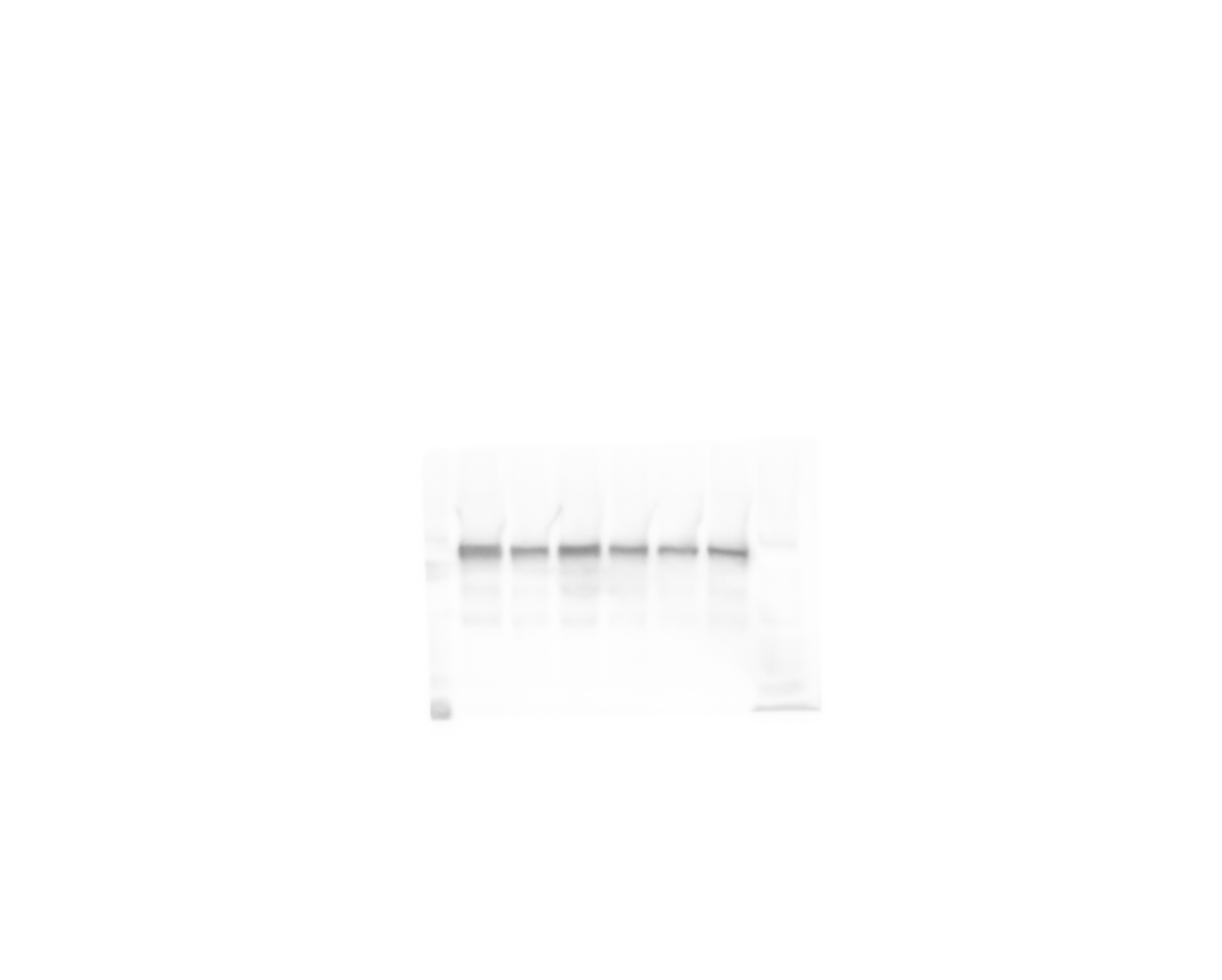

Supplement: Supplementary file 1 [file biomolecules-16-00868-s001.zip › FigureS1 the full, uncropped western blot images/The vivo mice study/mtHSP70/1-0.3s.Tif]

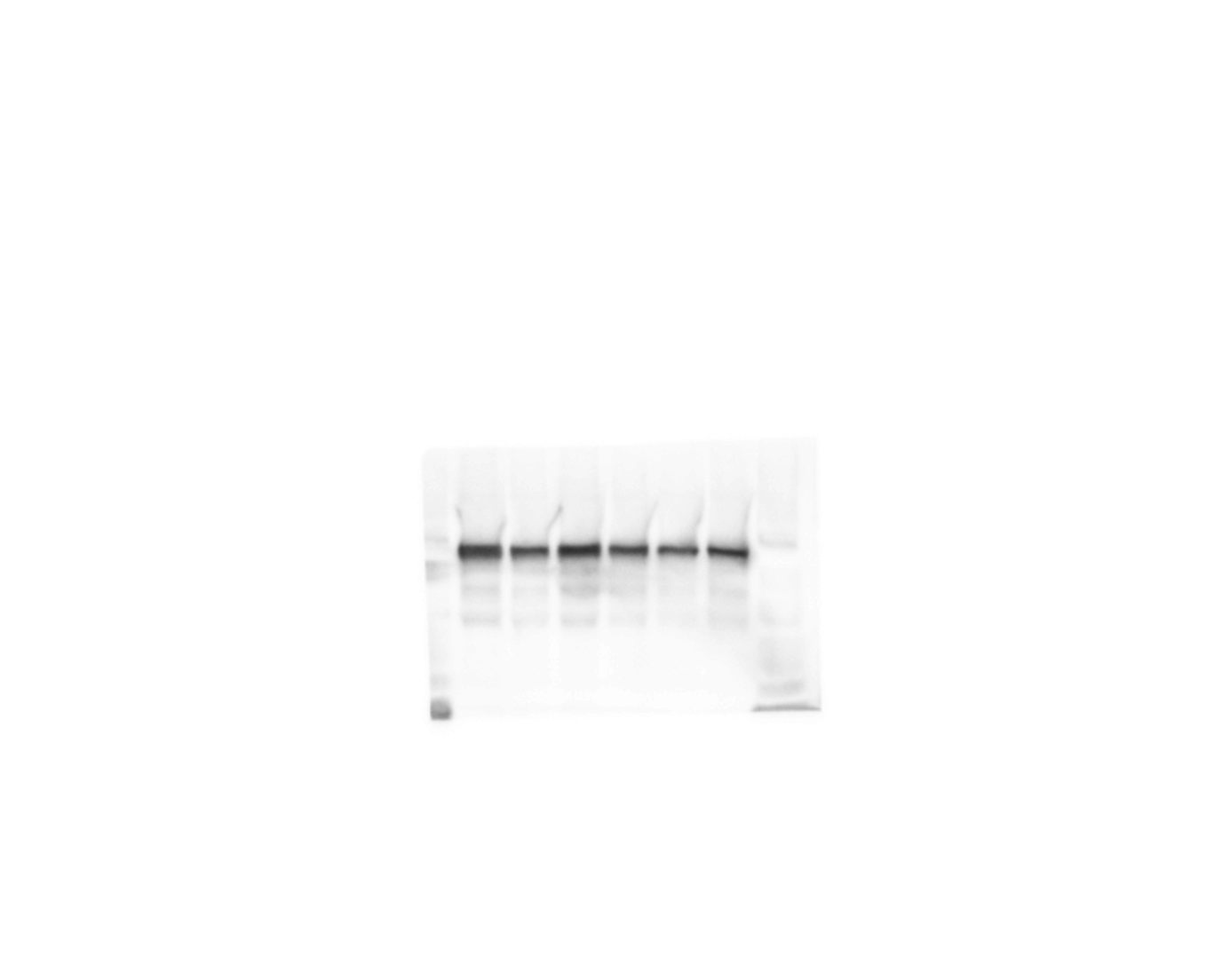

Supplement: Supplementary file 1 [file biomolecules-16-00868-s001.zip › FigureS1 the full, uncropped western blot images/The vivo mice study/mtHSP70/1-2s.Tif]

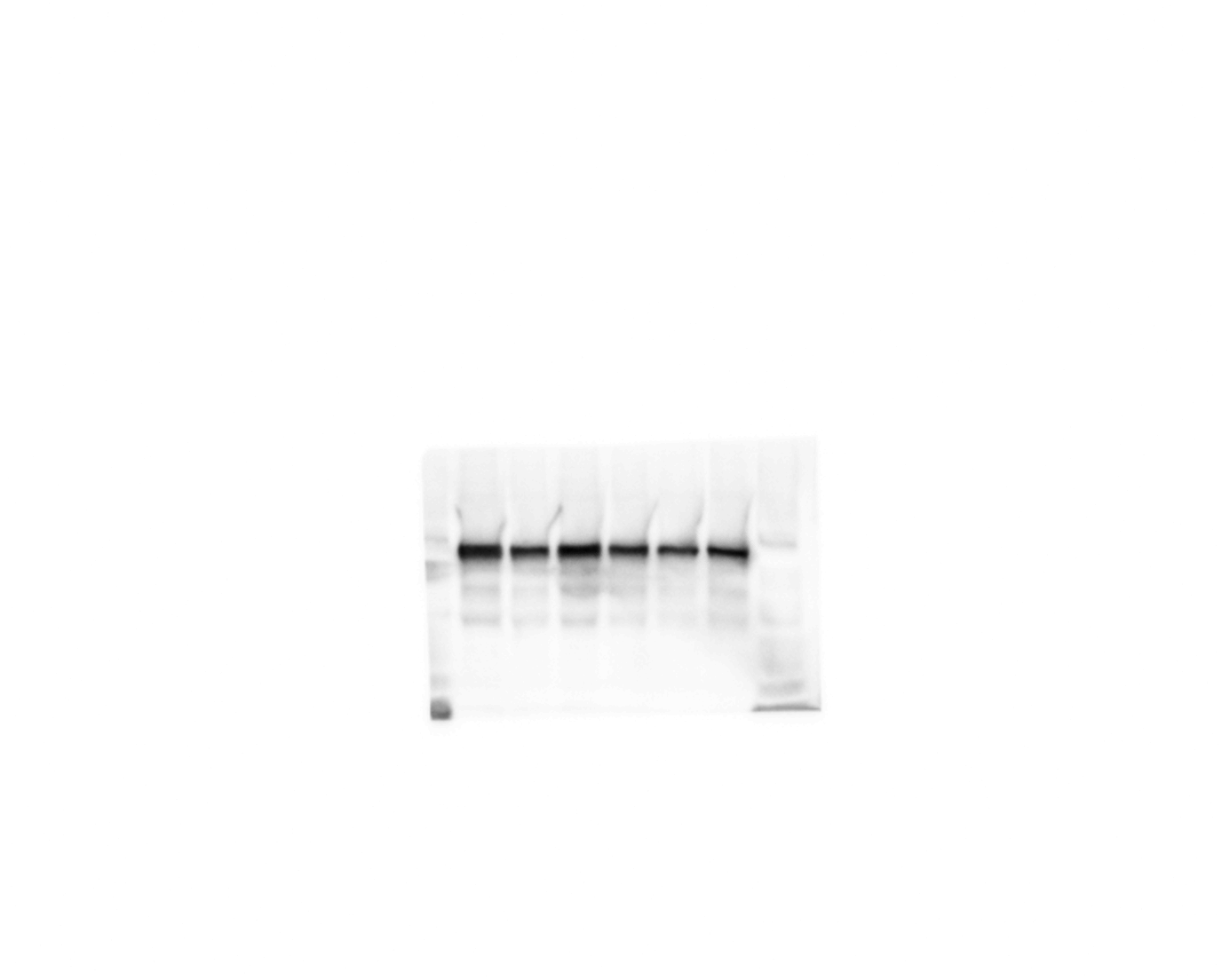

Supplement: Supplementary file 1 [file biomolecules-16-00868-s001.zip › FigureS1 the full, uncropped western blot images/The vivo mice study/mtHSP70/1-5s.Tif]

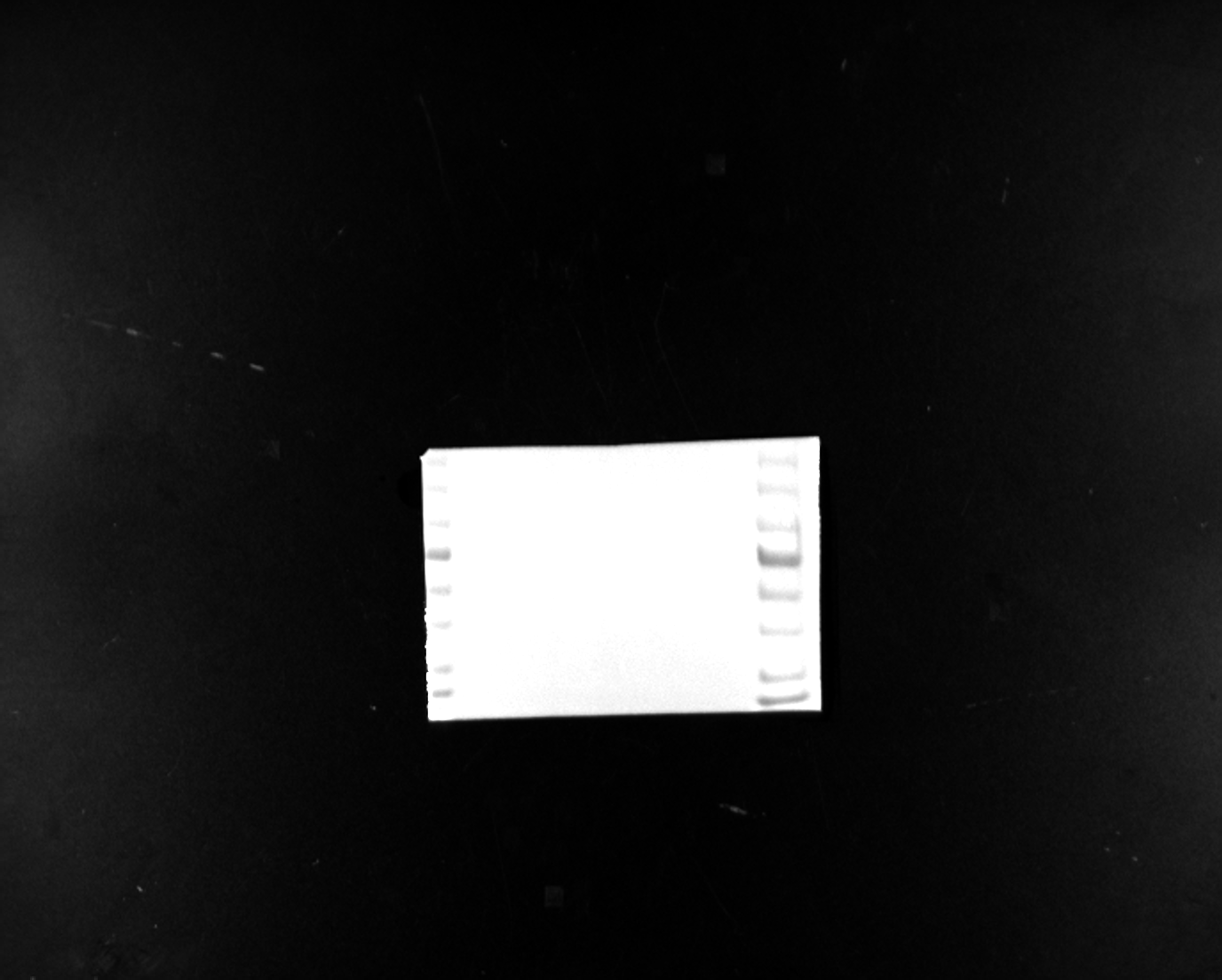

Supplement: Supplementary file 1 [file biomolecules-16-00868-s001.zip › FigureS1 the full, uncropped western blot images/The vivo mice study/mtHSP70/1-t.Tif]

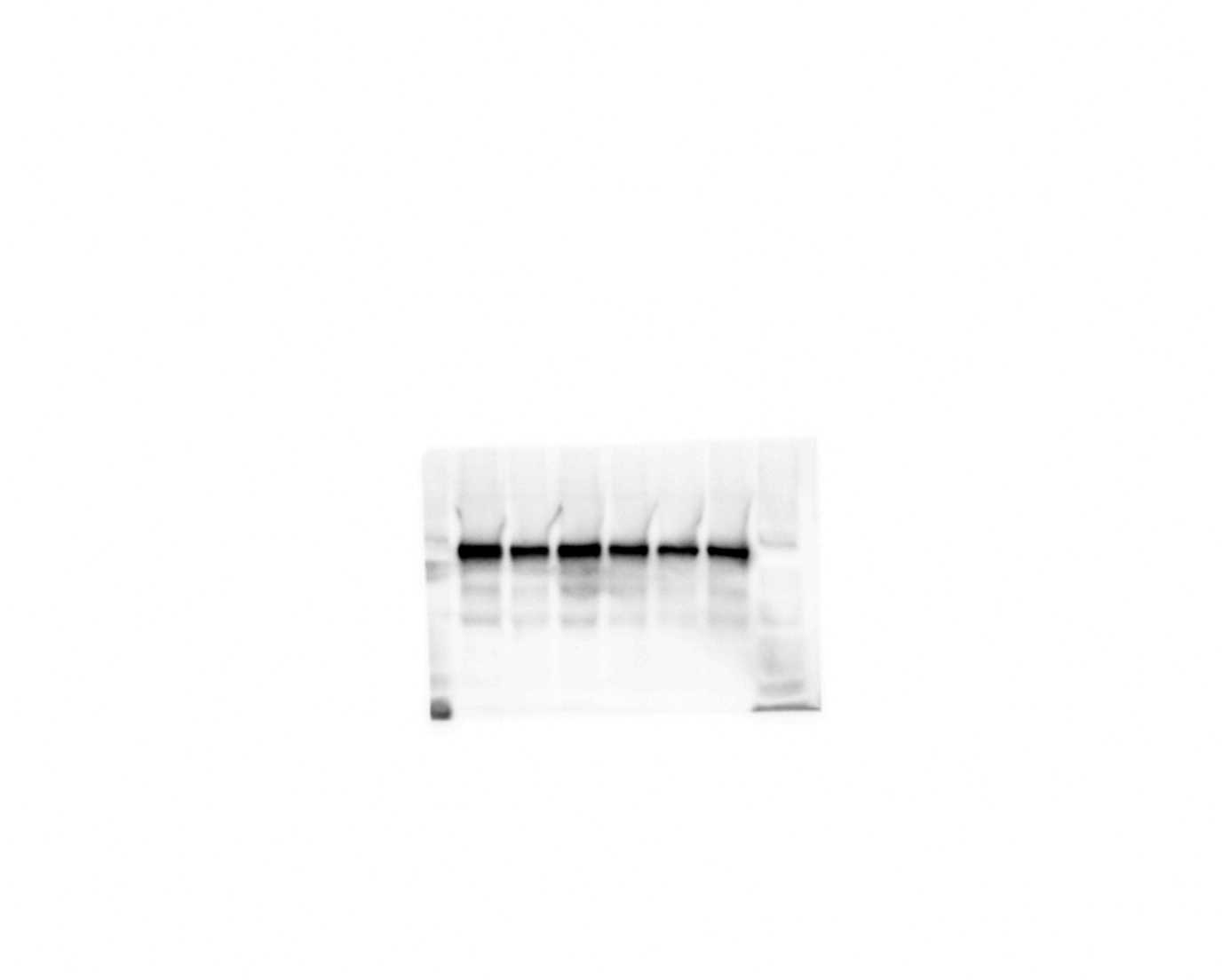

Supplement: Supplementary file 1 [file biomolecules-16-00868-s001.zip › FigureS1 the full, uncropped western blot images/The vivo mice study/mtHSP70/1.Tif]

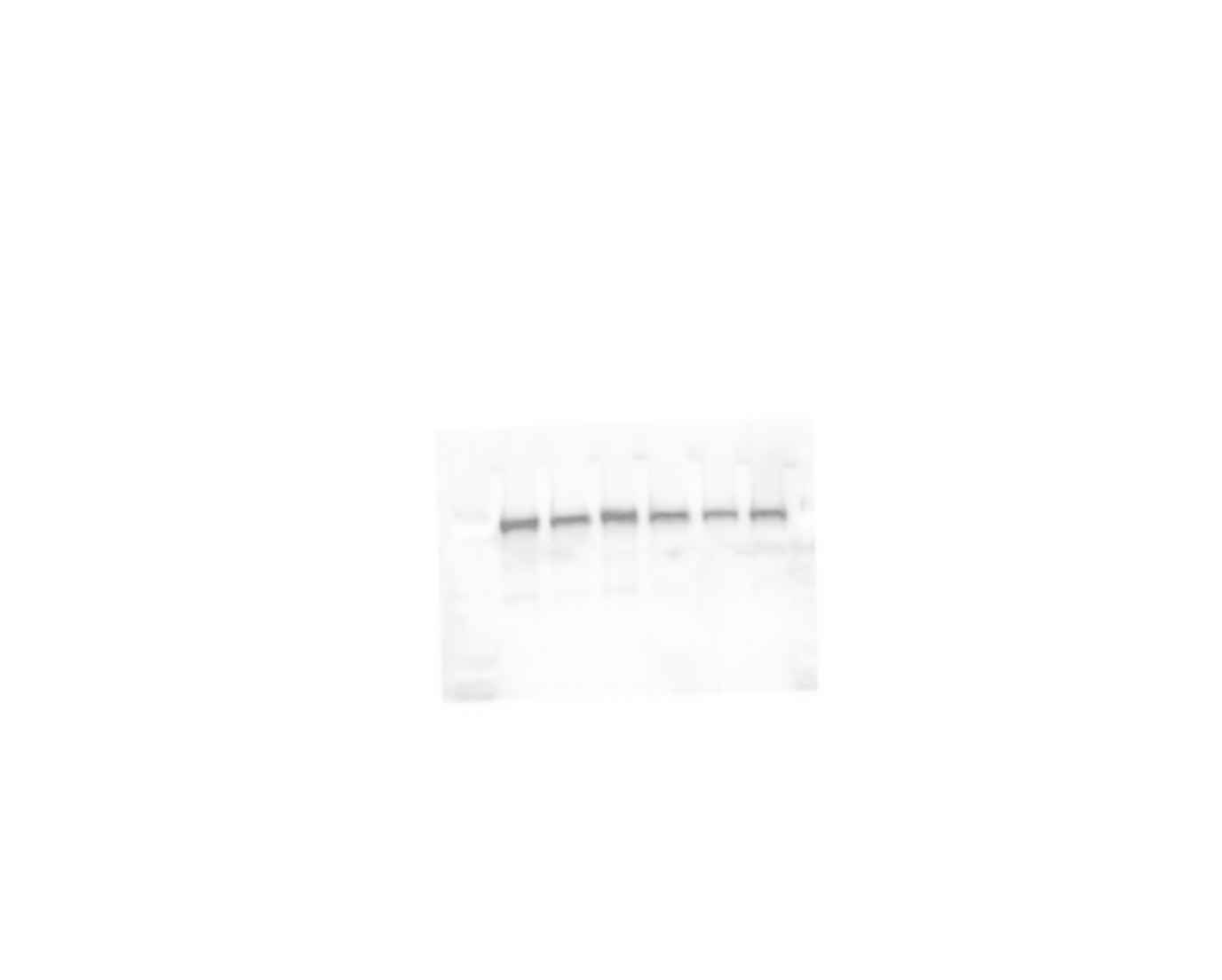

Supplement: Supplementary file 1 [file biomolecules-16-00868-s001.zip › FigureS1 the full, uncropped western blot images/The vivo mice study/mtHSP70/2-0.6s.Tif]

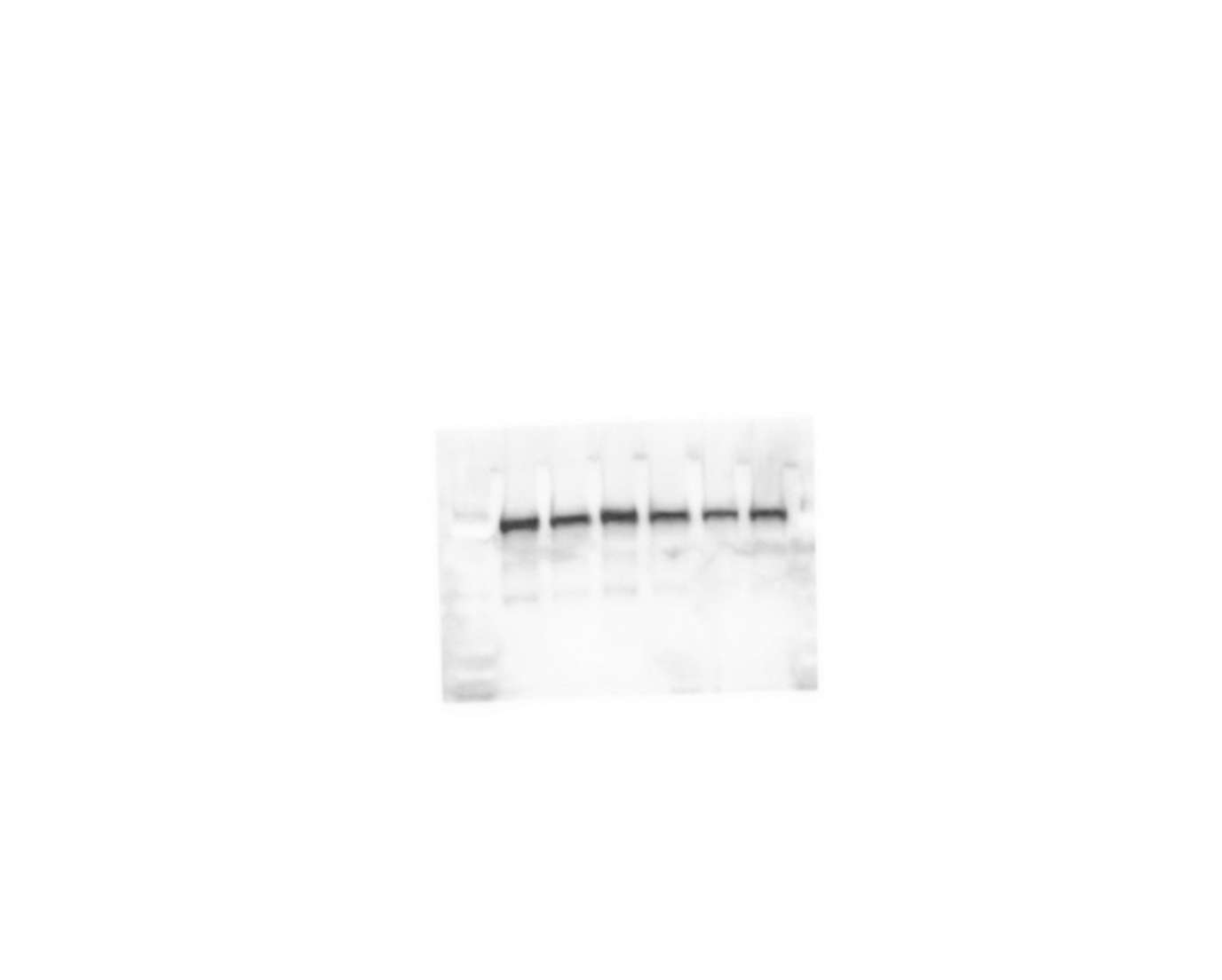

Supplement: Supplementary file 1 [file biomolecules-16-00868-s001.zip › FigureS1 the full, uncropped western blot images/The vivo mice study/mtHSP70/2-2s.Tif]

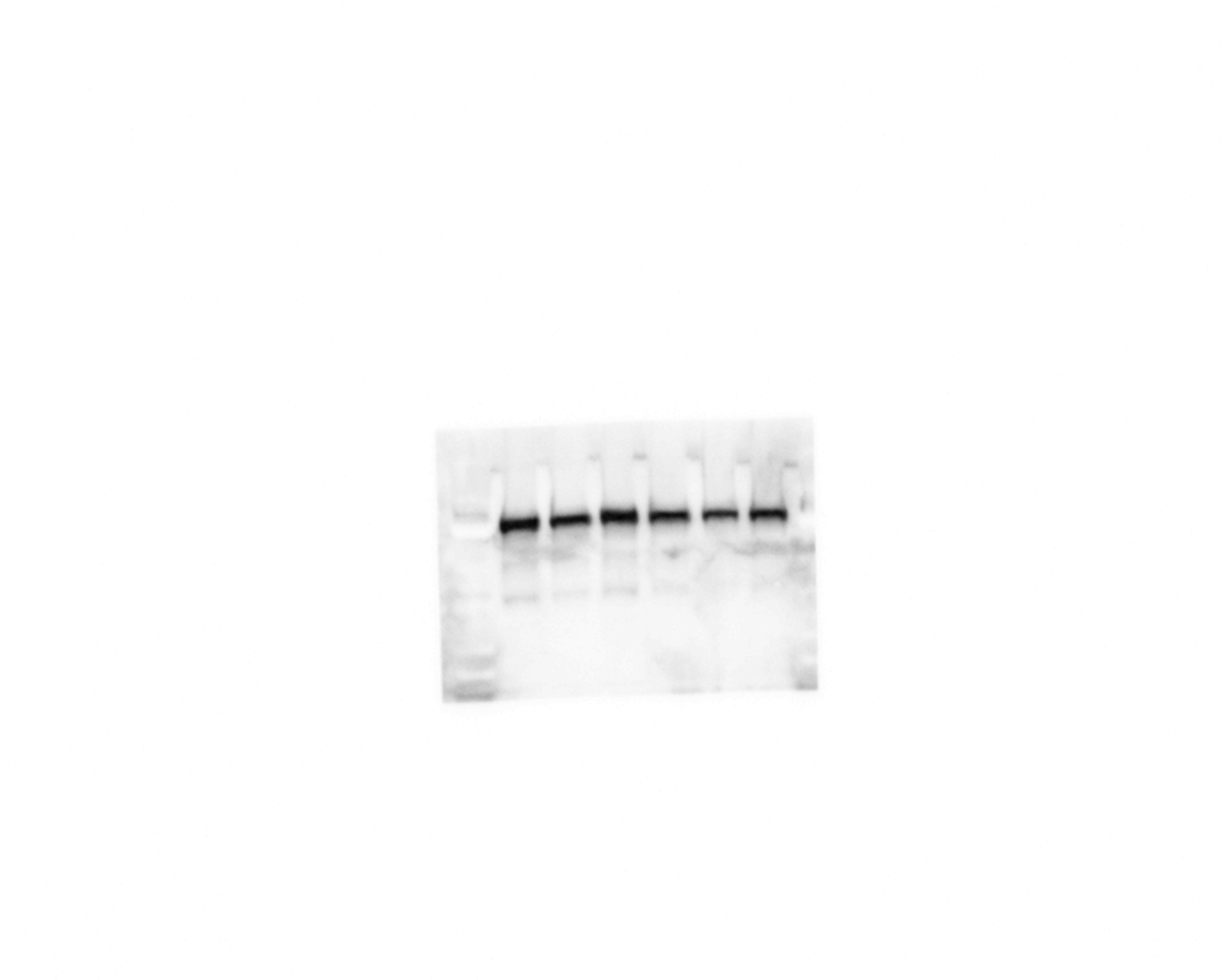

Supplement: Supplementary file 1 [file biomolecules-16-00868-s001.zip › FigureS1 the full, uncropped western blot images/The vivo mice study/mtHSP70/2-4s.Tif]

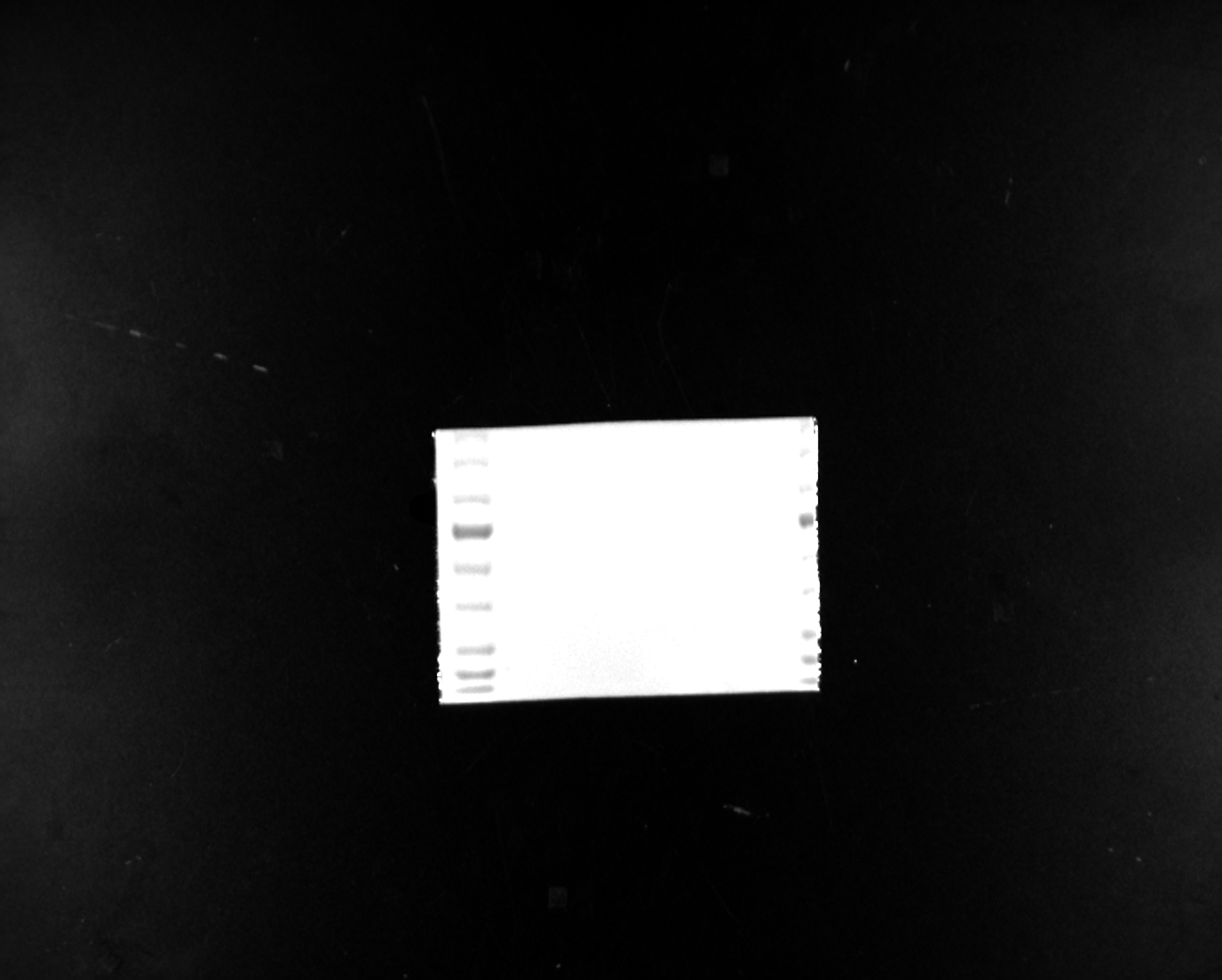

Supplement: Supplementary file 1 [file biomolecules-16-00868-s001.zip › FigureS1 the full, uncropped western blot images/The vivo mice study/mtHSP70/2-t.Tif]

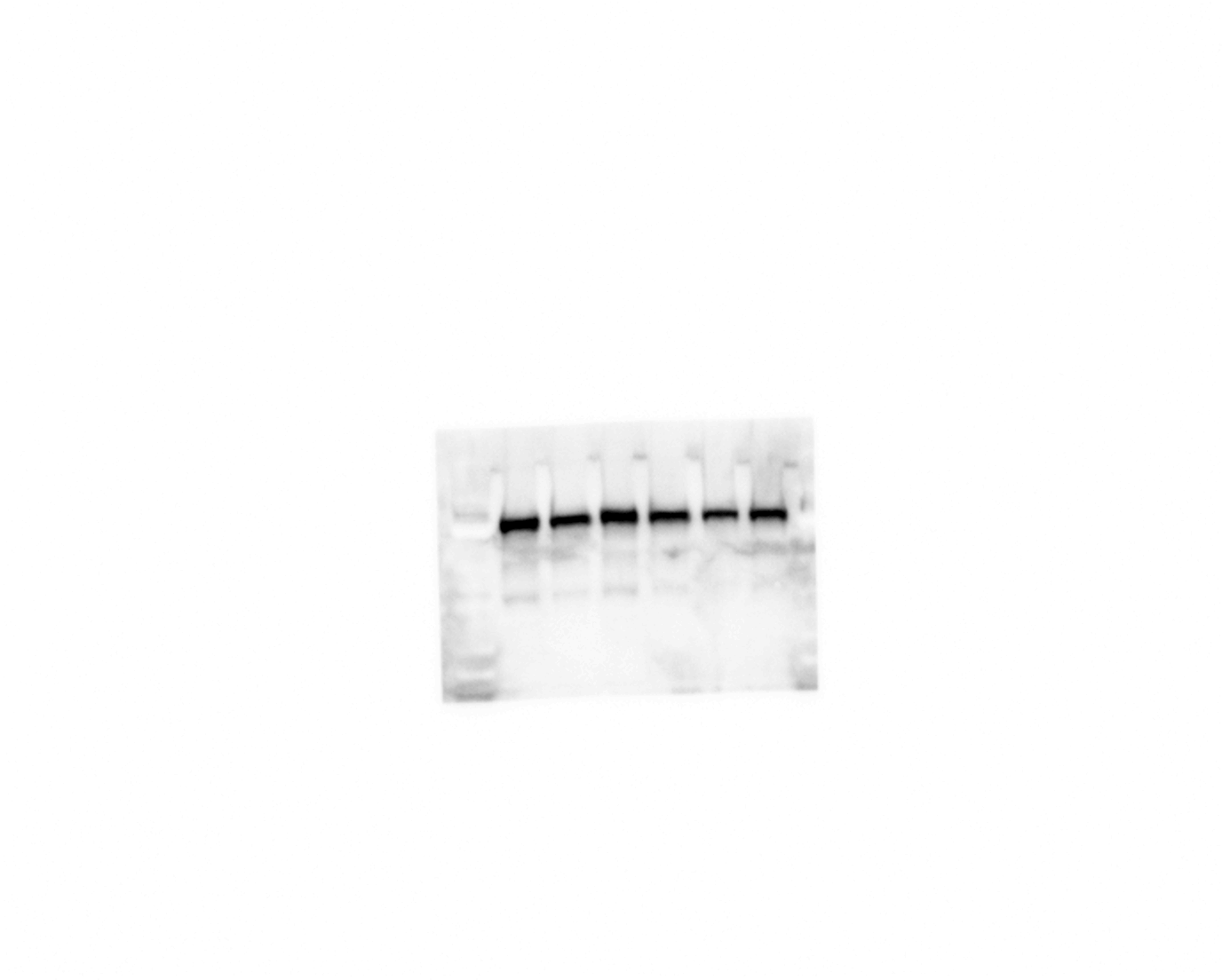

Supplement: Supplementary file 1 [file biomolecules-16-00868-s001.zip › FigureS1 the full, uncropped western blot images/The vivo mice study/mtHSP70/2.Tif]

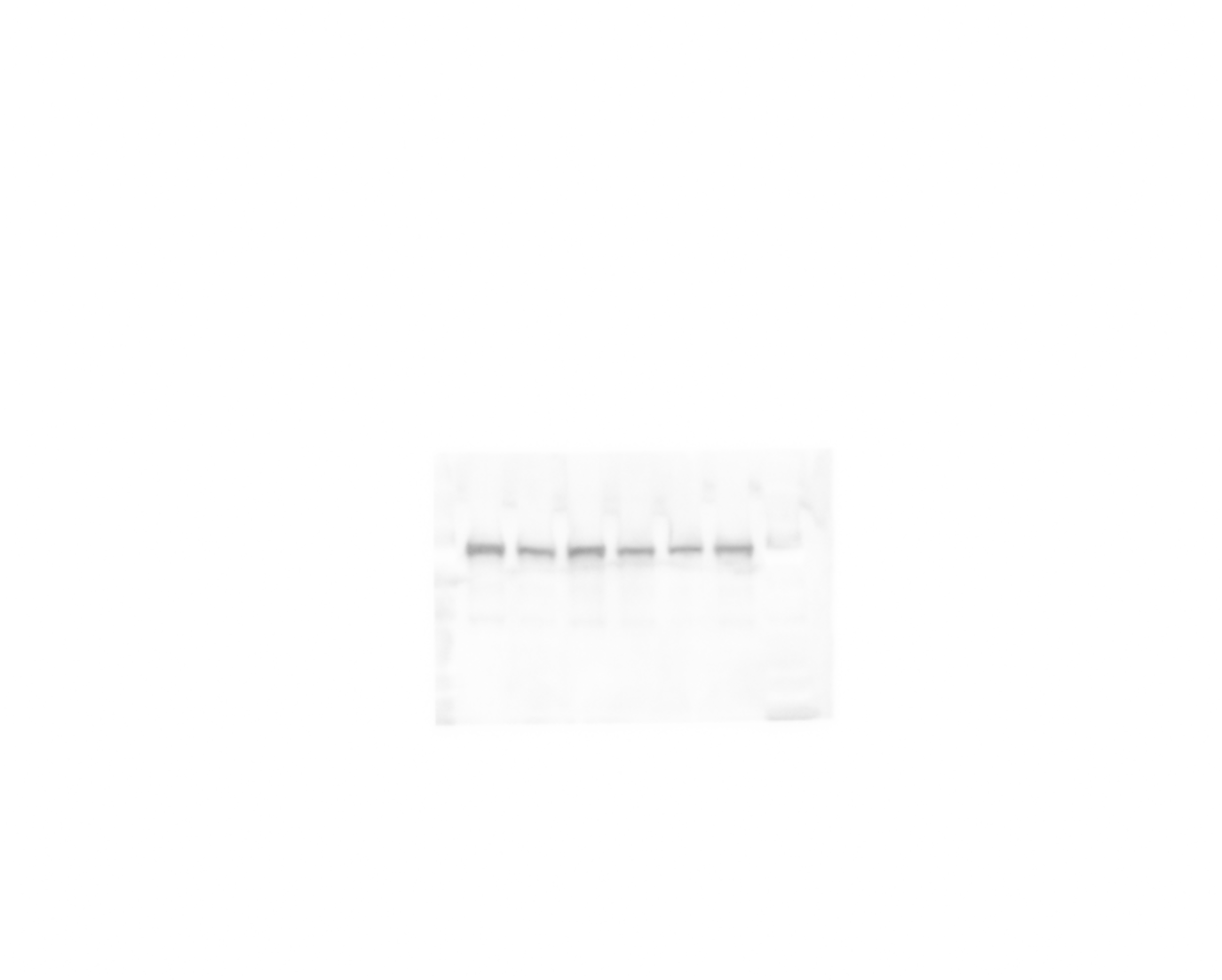

Supplement: Supplementary file 1 [file biomolecules-16-00868-s001.zip › FigureS1 the full, uncropped western blot images/The vivo mice study/mtHSP70/3-0.5s.Tif]

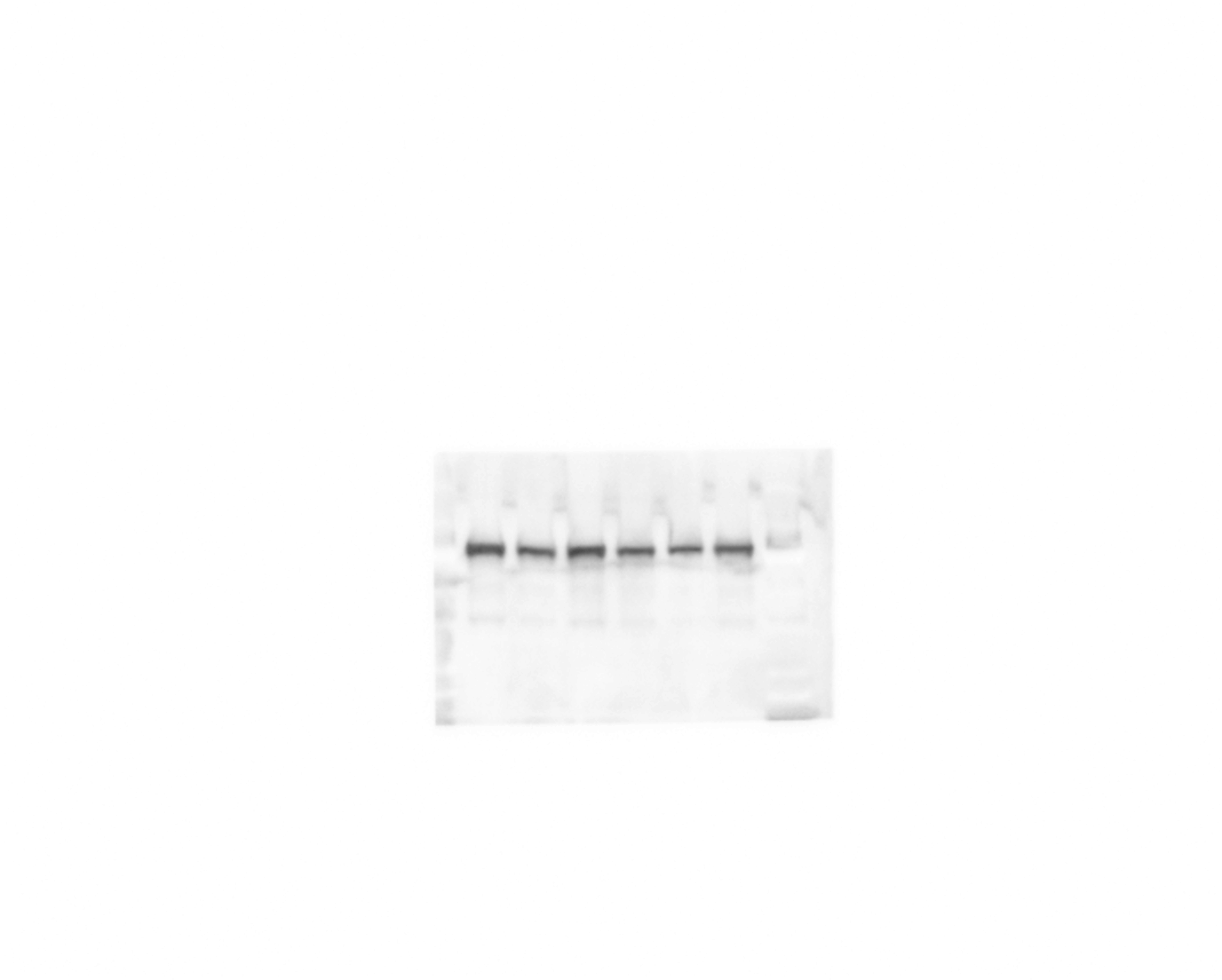

Supplement: Supplementary file 1 [file biomolecules-16-00868-s001.zip › FigureS1 the full, uncropped western blot images/The vivo mice study/mtHSP70/3-2s.Tif]

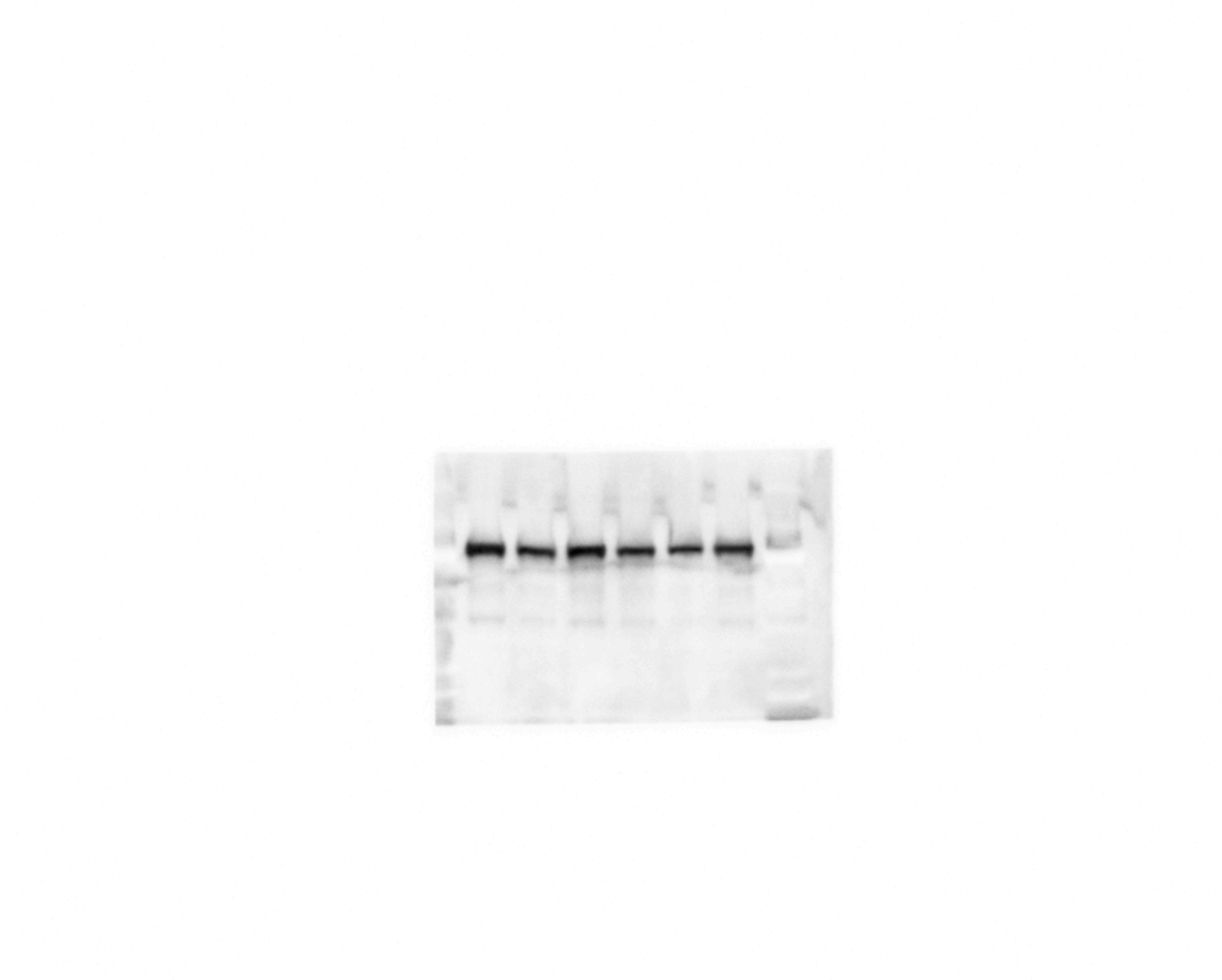

Supplement: Supplementary file 1 [file biomolecules-16-00868-s001.zip › FigureS1 the full, uncropped western blot images/The vivo mice study/mtHSP70/3-4s.Tif]

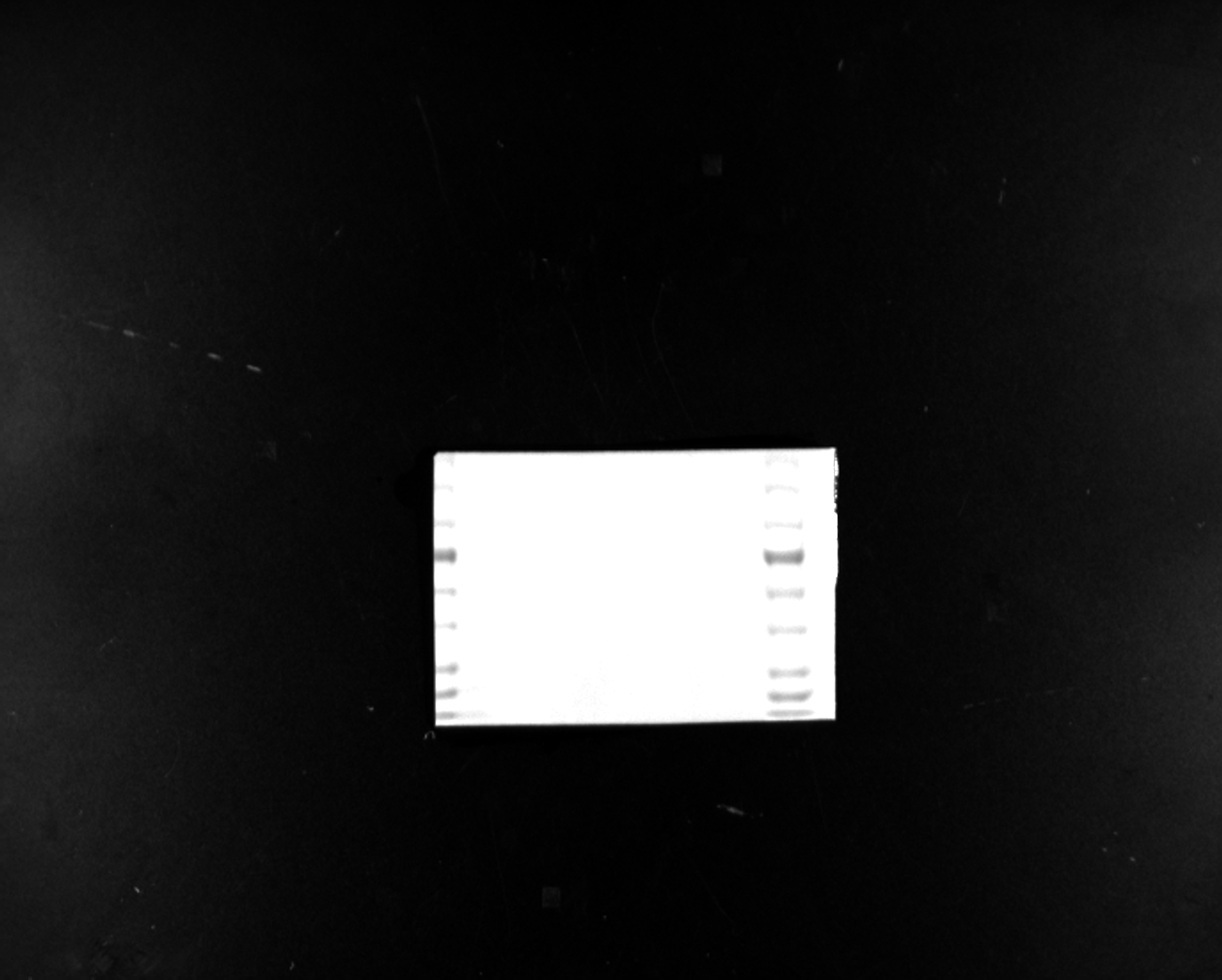

Supplement: Supplementary file 1 [file biomolecules-16-00868-s001.zip › FigureS1 the full, uncropped western blot images/The vivo mice study/mtHSP70/3-t.Tif]

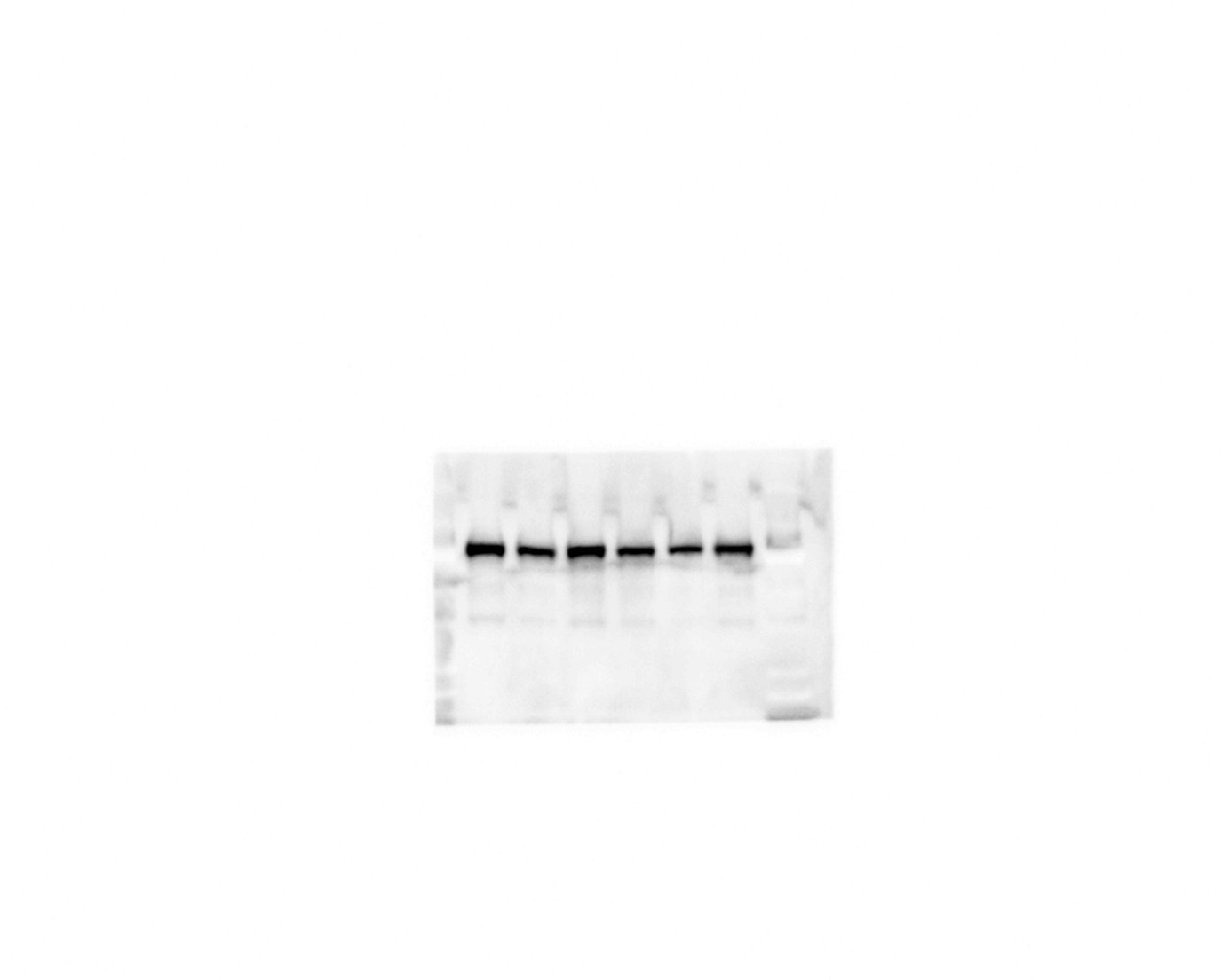

Supplement: Supplementary file 1 [file biomolecules-16-00868-s001.zip › FigureS1 the full, uncropped western blot images/The vivo mice study/mtHSP70/3.Tif]

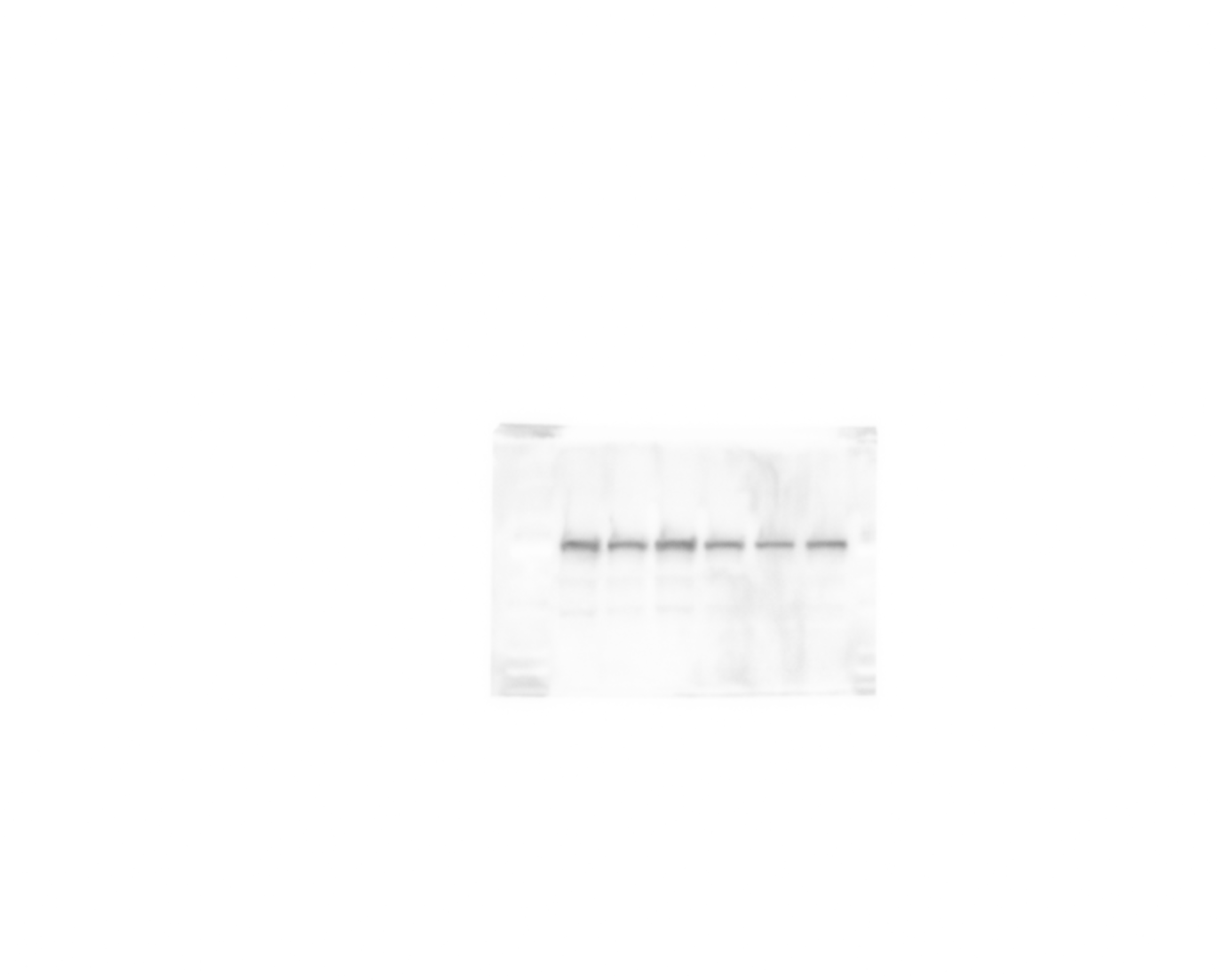

Supplement: Supplementary file 1 [file biomolecules-16-00868-s001.zip › FigureS1 the full, uncropped western blot images/The vivo mice study/mtHSP70/4-0.5s.Tif]

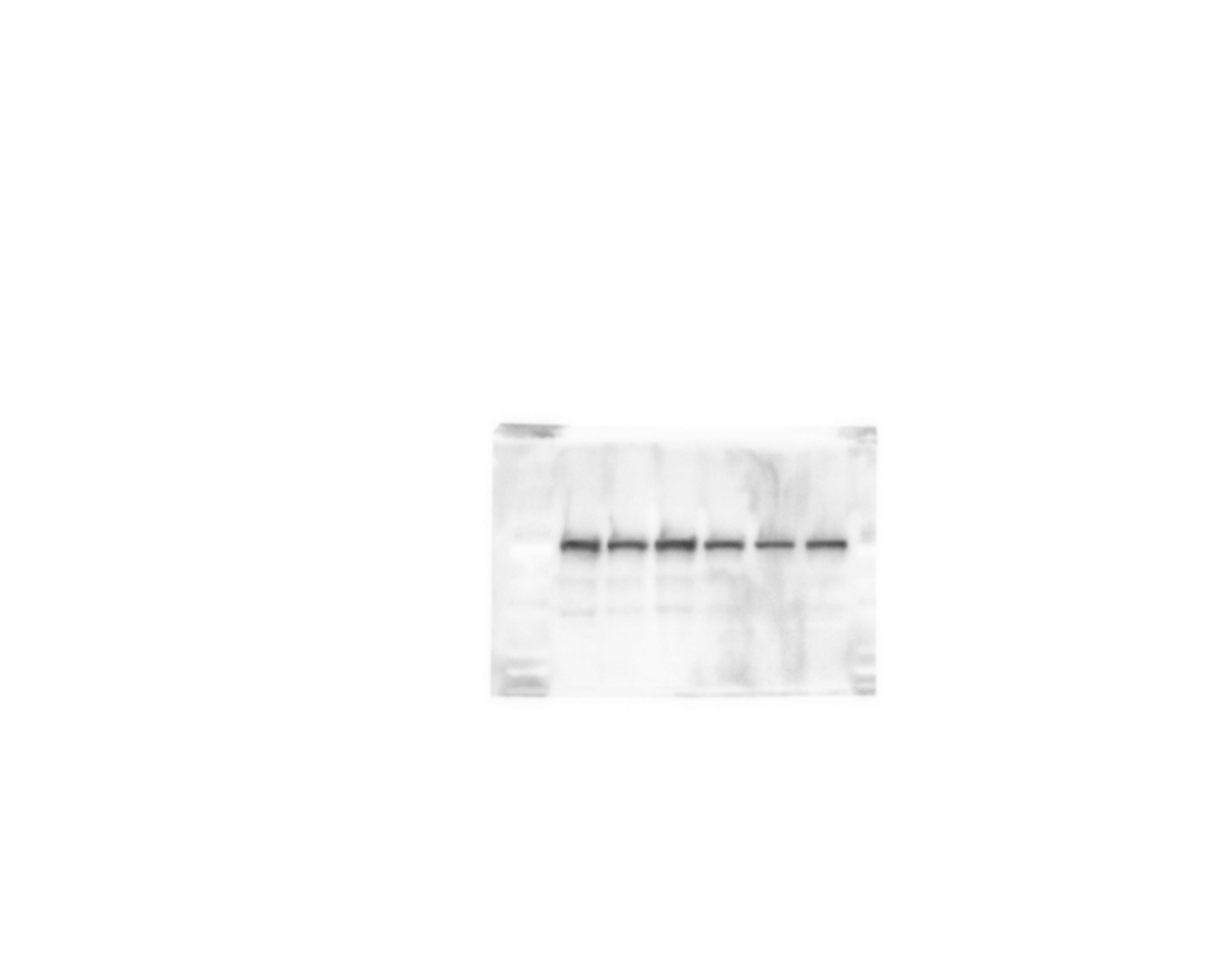

Supplement: Supplementary file 1 [file biomolecules-16-00868-s001.zip › FigureS1 the full, uncropped western blot images/The vivo mice study/mtHSP70/4-2s.Tif]

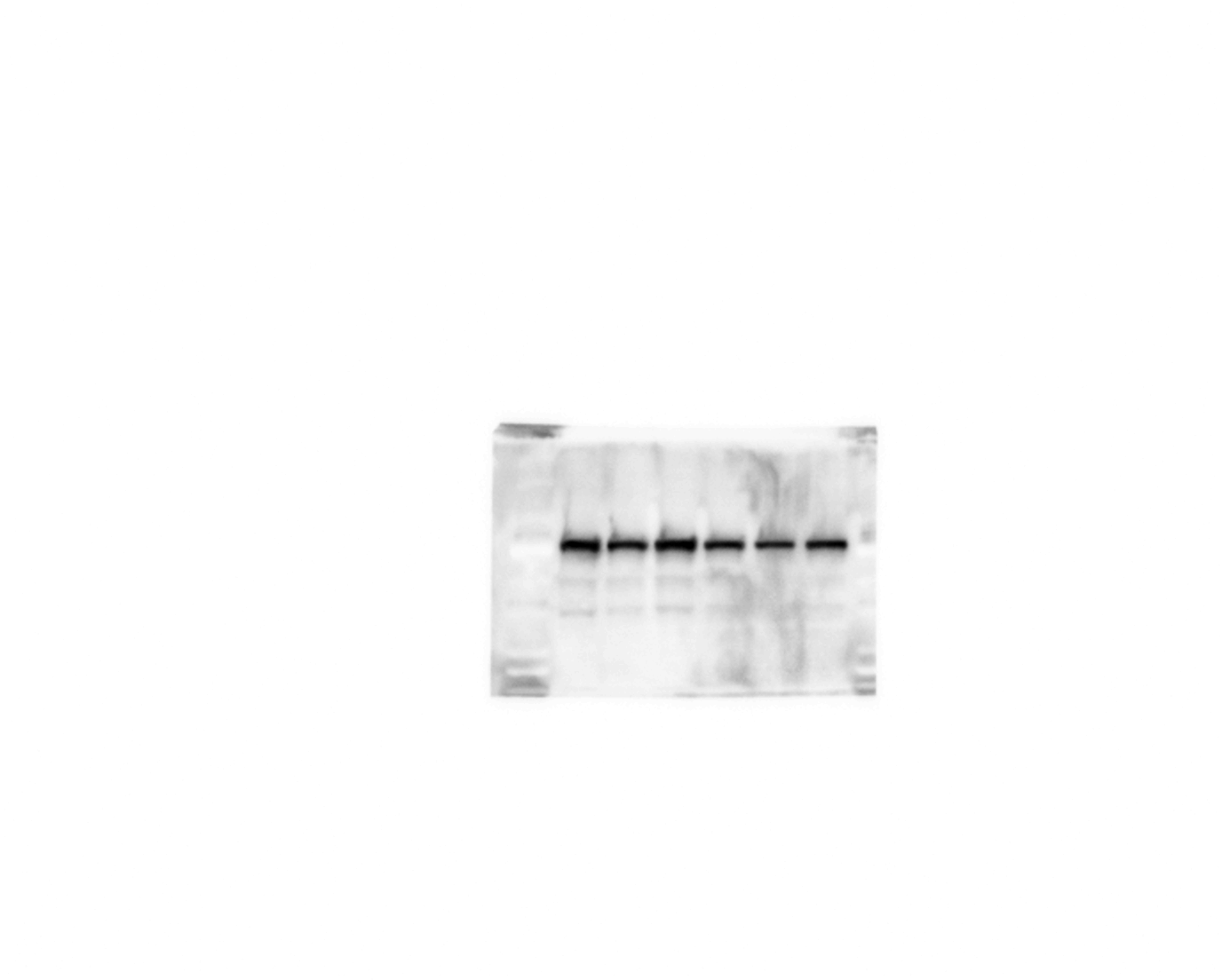

Supplement: Supplementary file 1 [file biomolecules-16-00868-s001.zip › FigureS1 the full, uncropped western blot images/The vivo mice study/mtHSP70/4-4s.Tif]

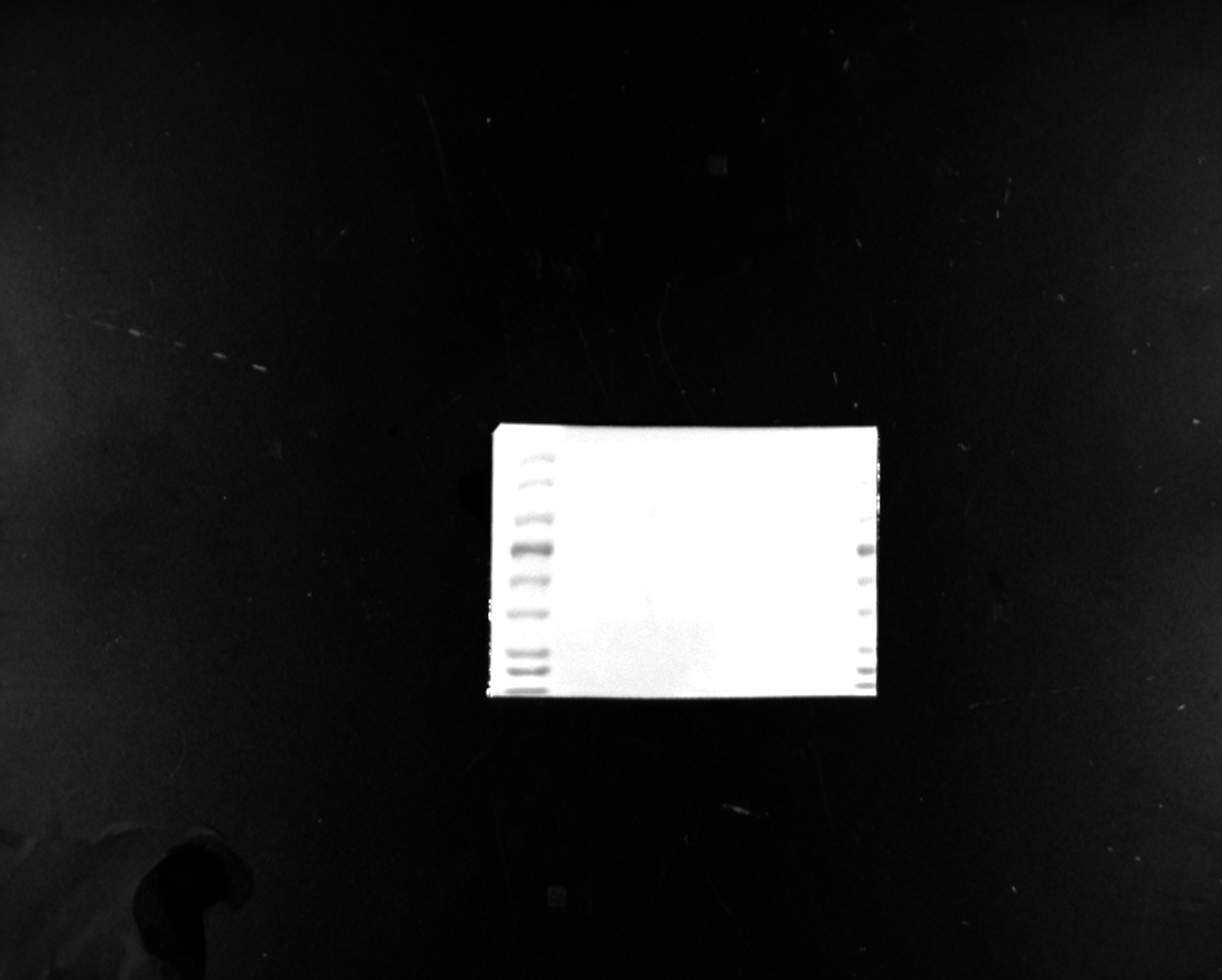

Supplement: Supplementary file 1 [file biomolecules-16-00868-s001.zip › FigureS1 the full, uncropped western blot images/The vivo mice study/mtHSP70/4-t.Tif]

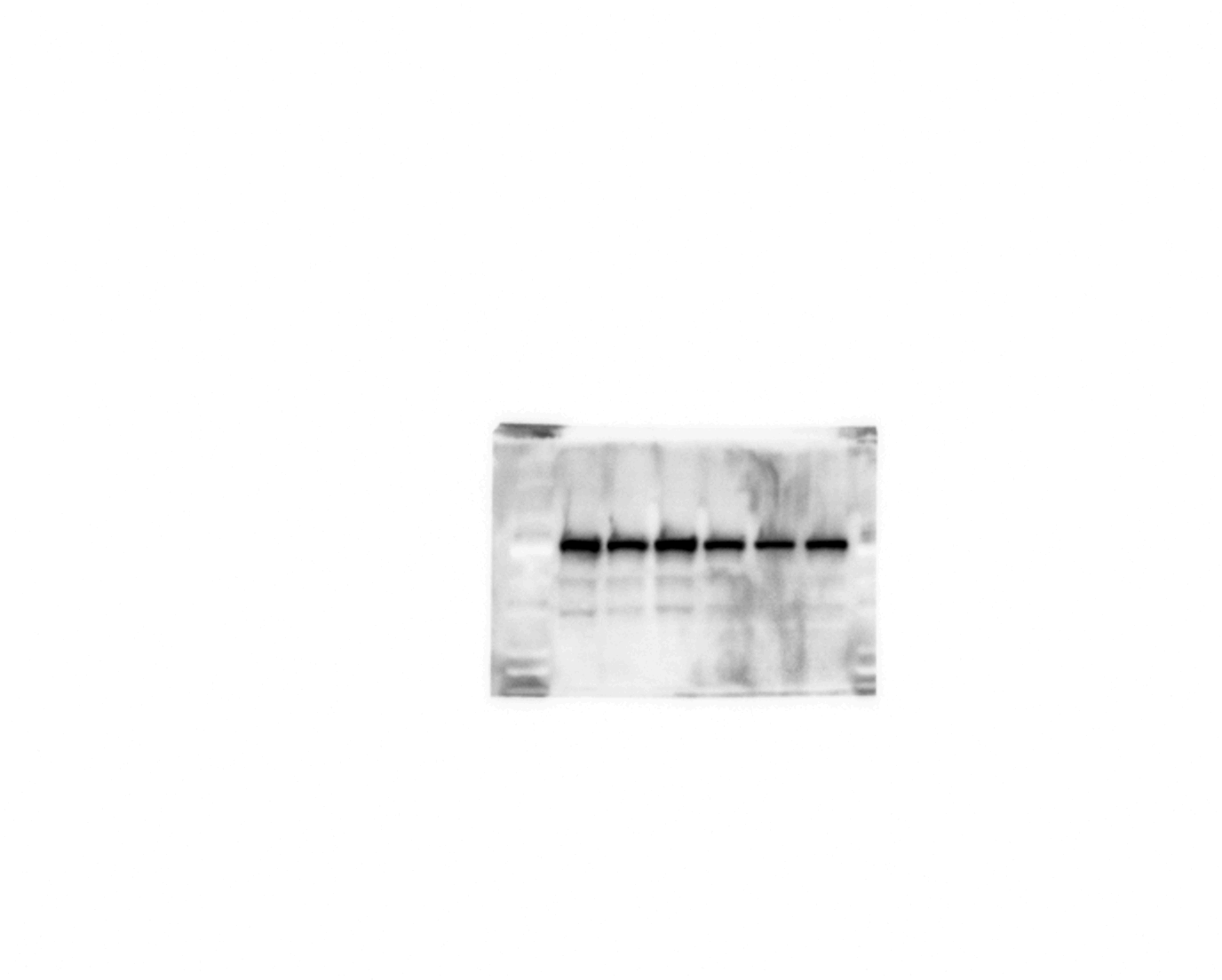

Supplement: Supplementary file 1 [file biomolecules-16-00868-s001.zip › FigureS1 the full, uncropped western blot images/The vivo mice study/mtHSP70/4.Tif]

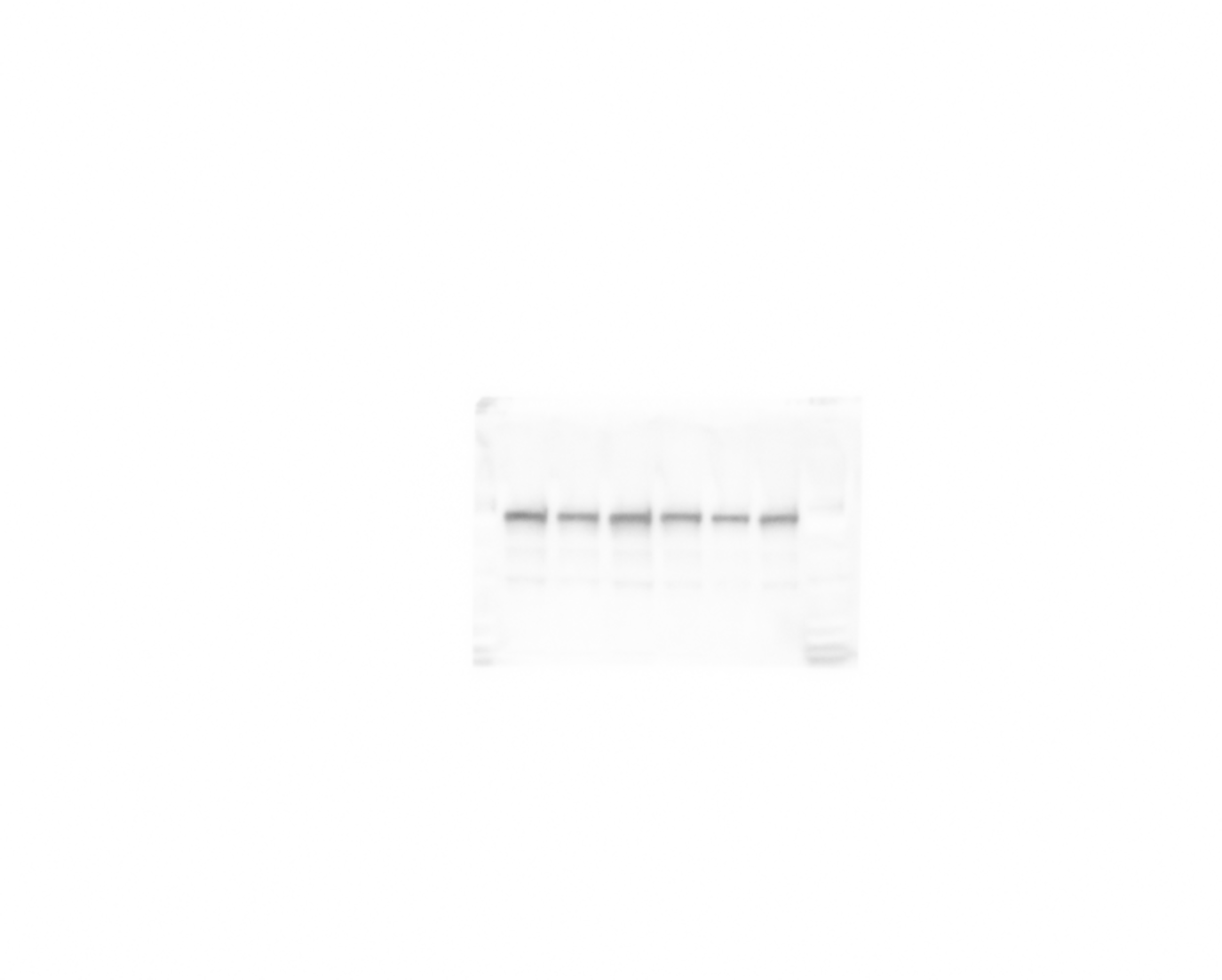

Supplement: Supplementary file 1 [file biomolecules-16-00868-s001.zip › FigureS1 the full, uncropped western blot images/The vivo mice study/mtHSP70/5-0.3s.Tif]

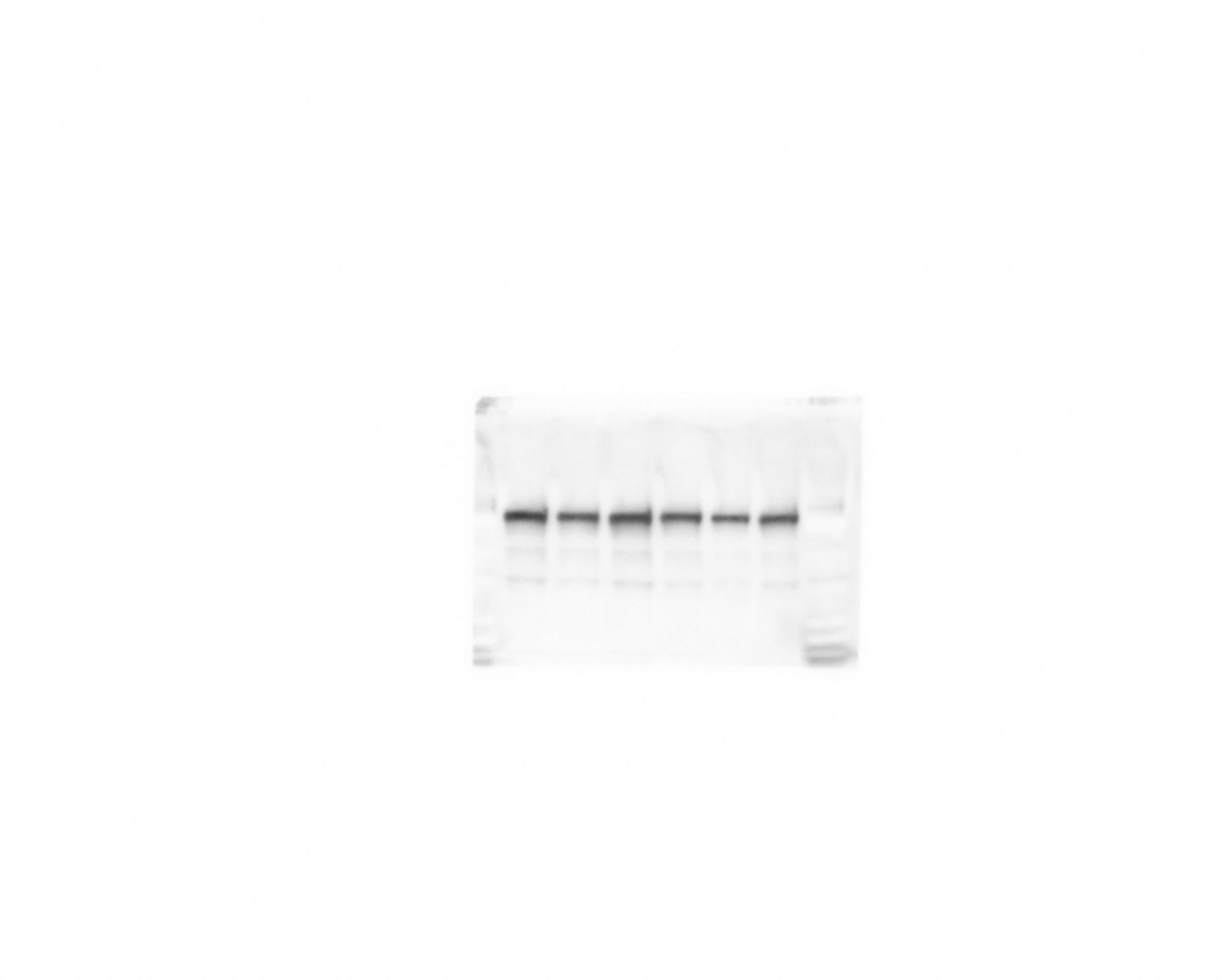

Supplement: Supplementary file 1 [file biomolecules-16-00868-s001.zip › FigureS1 the full, uncropped western blot images/The vivo mice study/mtHSP70/5-1s.Tif]

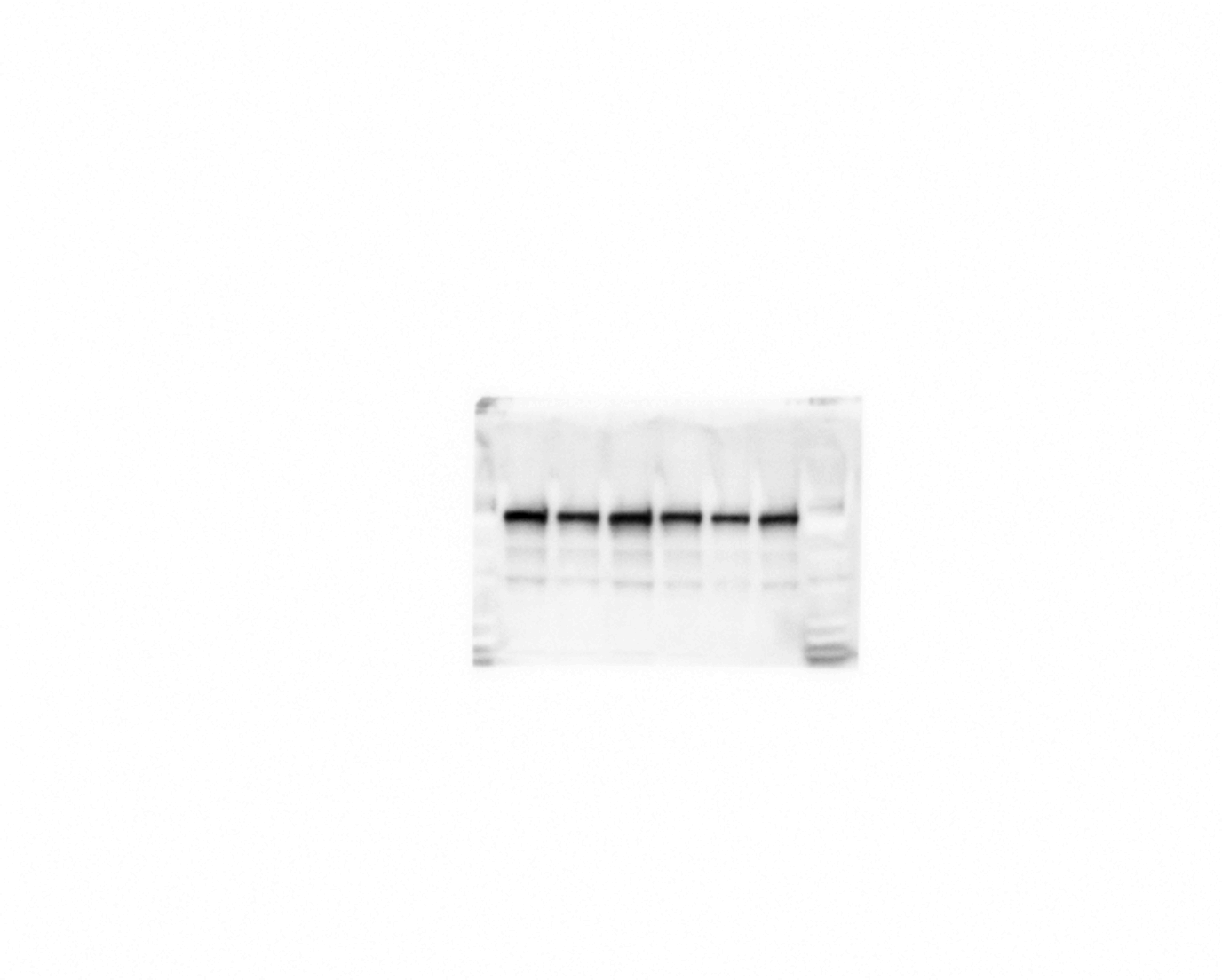

Supplement: Supplementary file 1 [file biomolecules-16-00868-s001.zip › FigureS1 the full, uncropped western blot images/The vivo mice study/mtHSP70/5-4s.Tif]

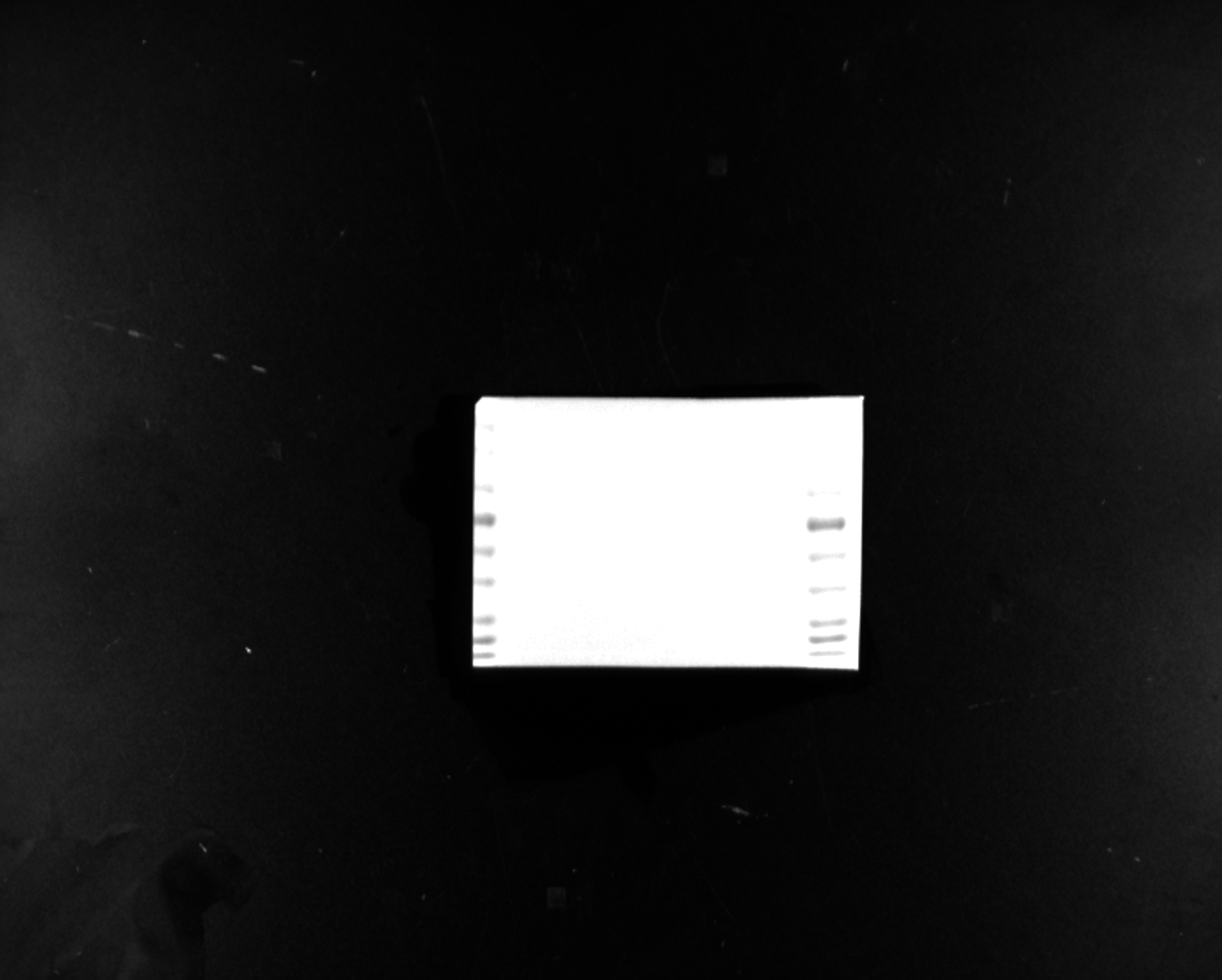

Supplement: Supplementary file 1 [file biomolecules-16-00868-s001.zip › FigureS1 the full, uncropped western blot images/The vivo mice study/mtHSP70/5-t.Tif]

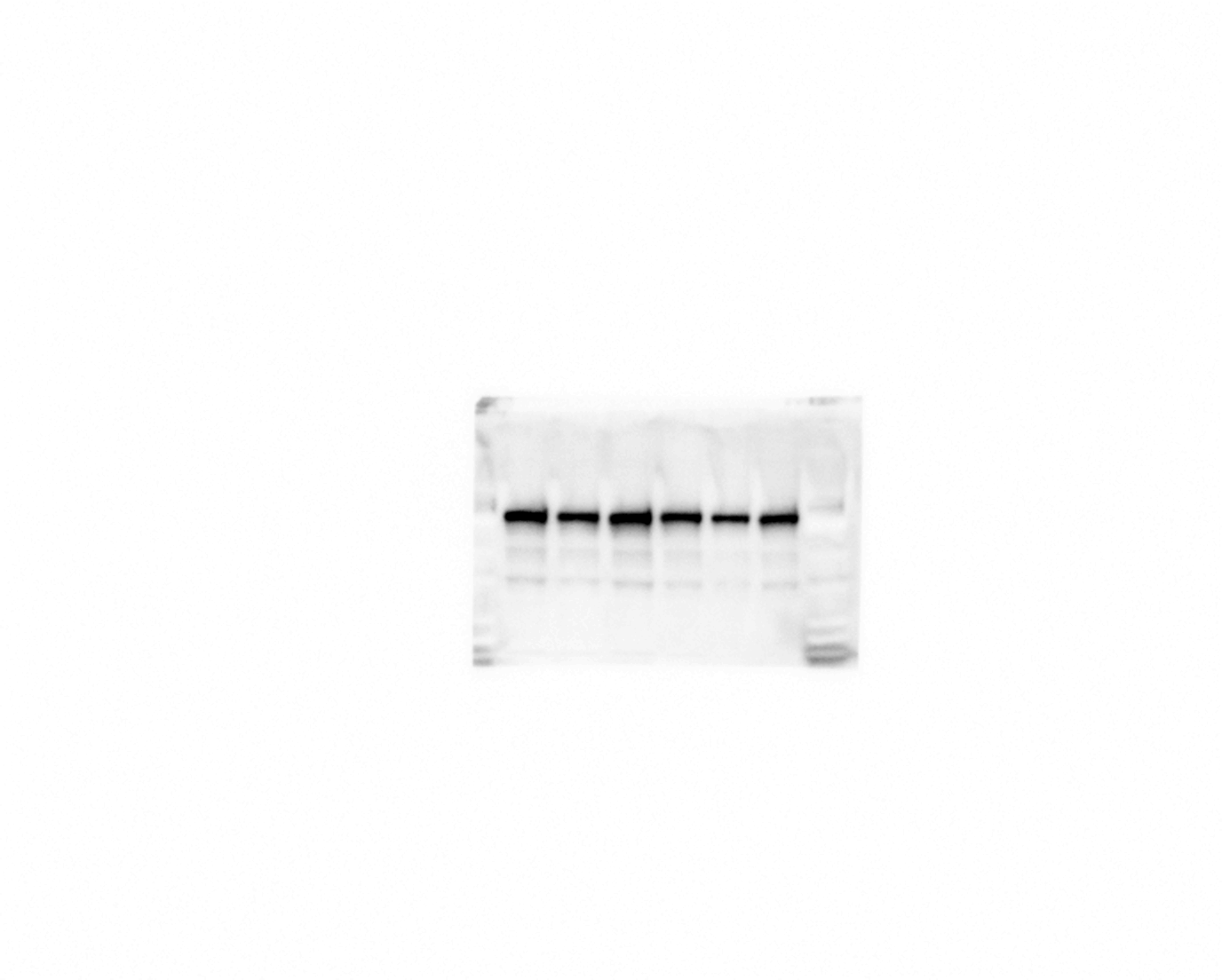

Supplement: Supplementary file 1 [file biomolecules-16-00868-s001.zip › FigureS1 the full, uncropped western blot images/The vivo mice study/mtHSP70/5.Tif]

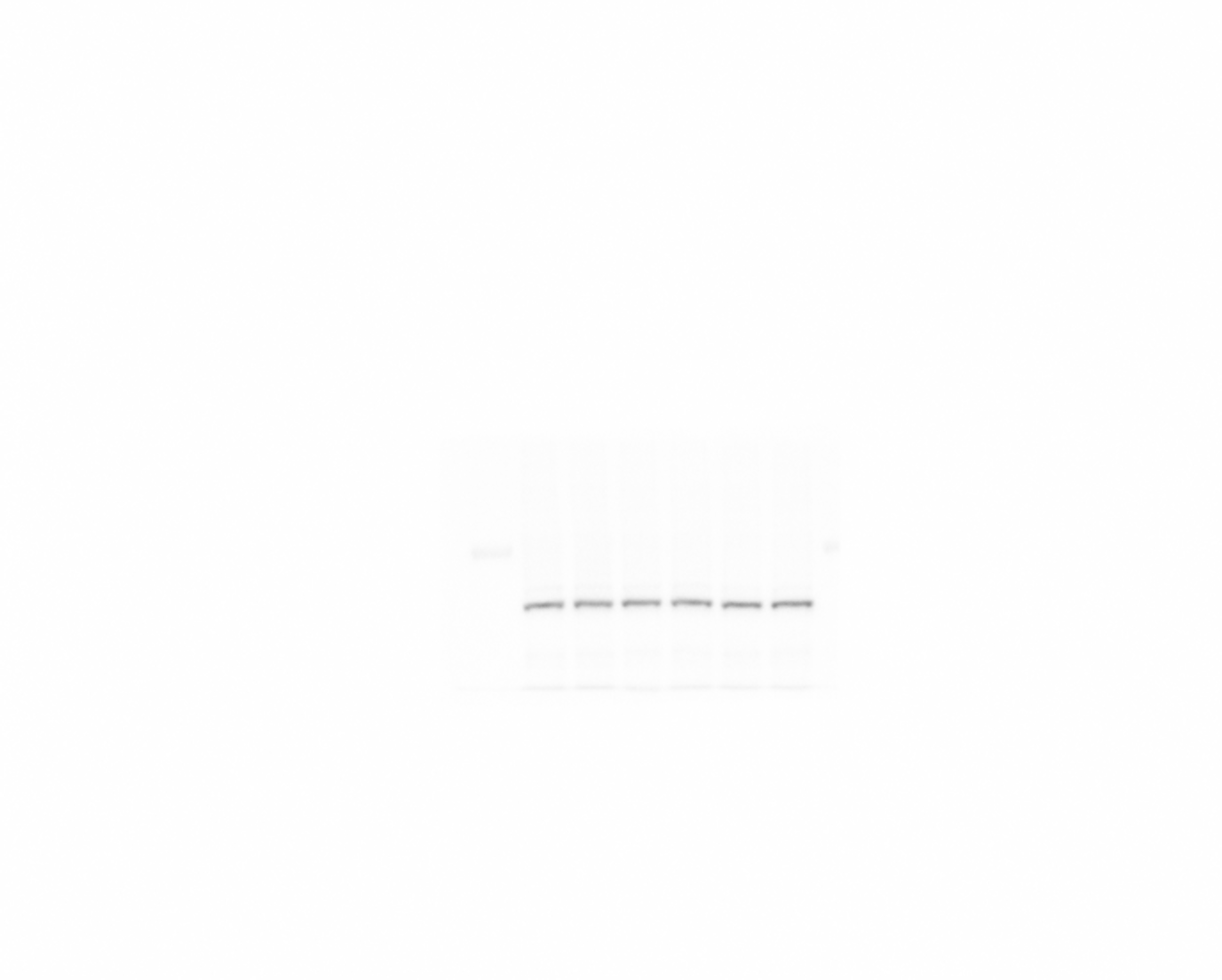

Supplement: Supplementary file 1 [file biomolecules-16-00868-s001.zip › FigureS1 the full, uncropped western blot images/The vivo mice study/β-actin/1-0.4s.Tif]

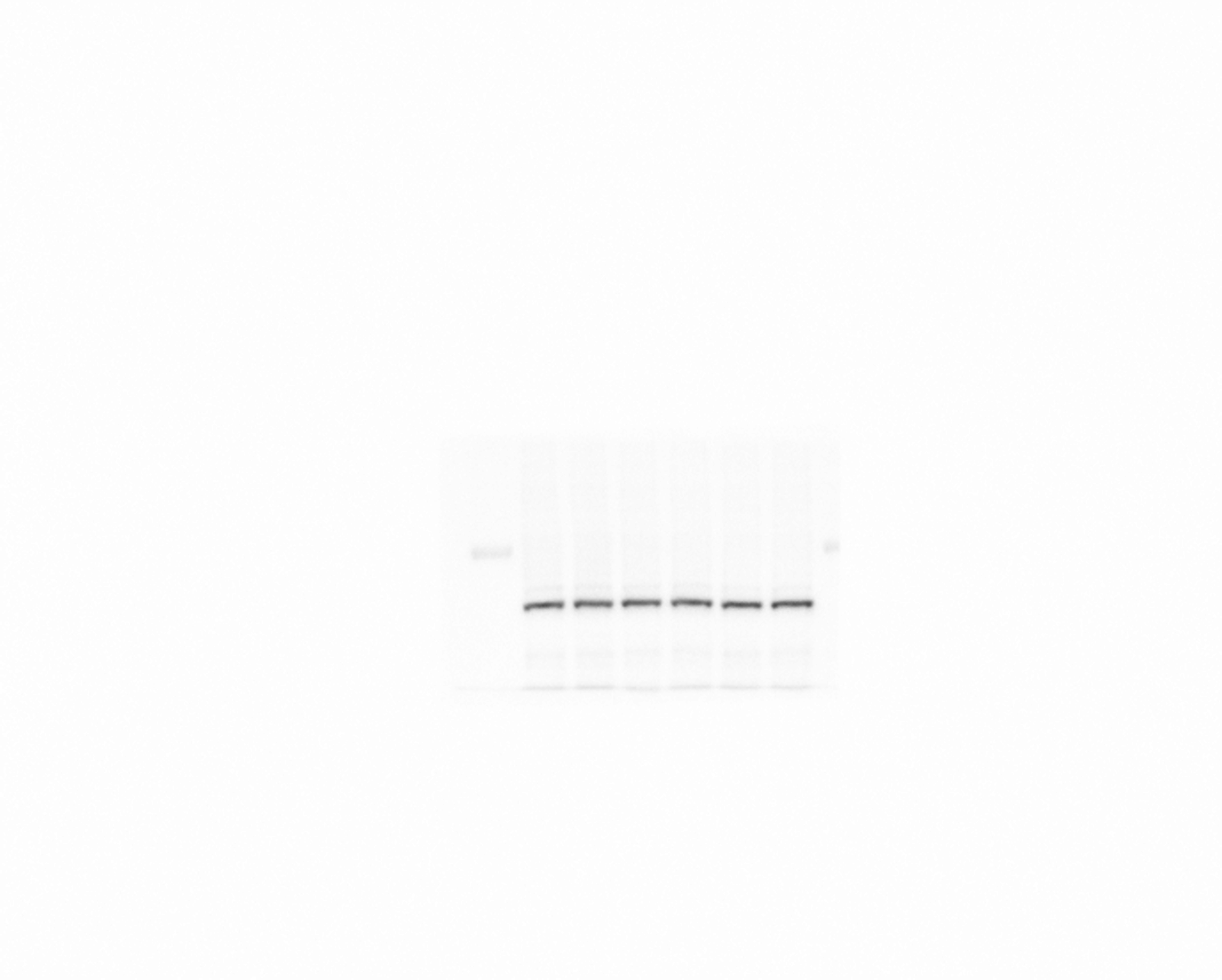

Supplement: Supplementary file 1 [file biomolecules-16-00868-s001.zip › FigureS1 the full, uncropped western blot images/The vivo mice study/β-actin/1-3s.Tif]

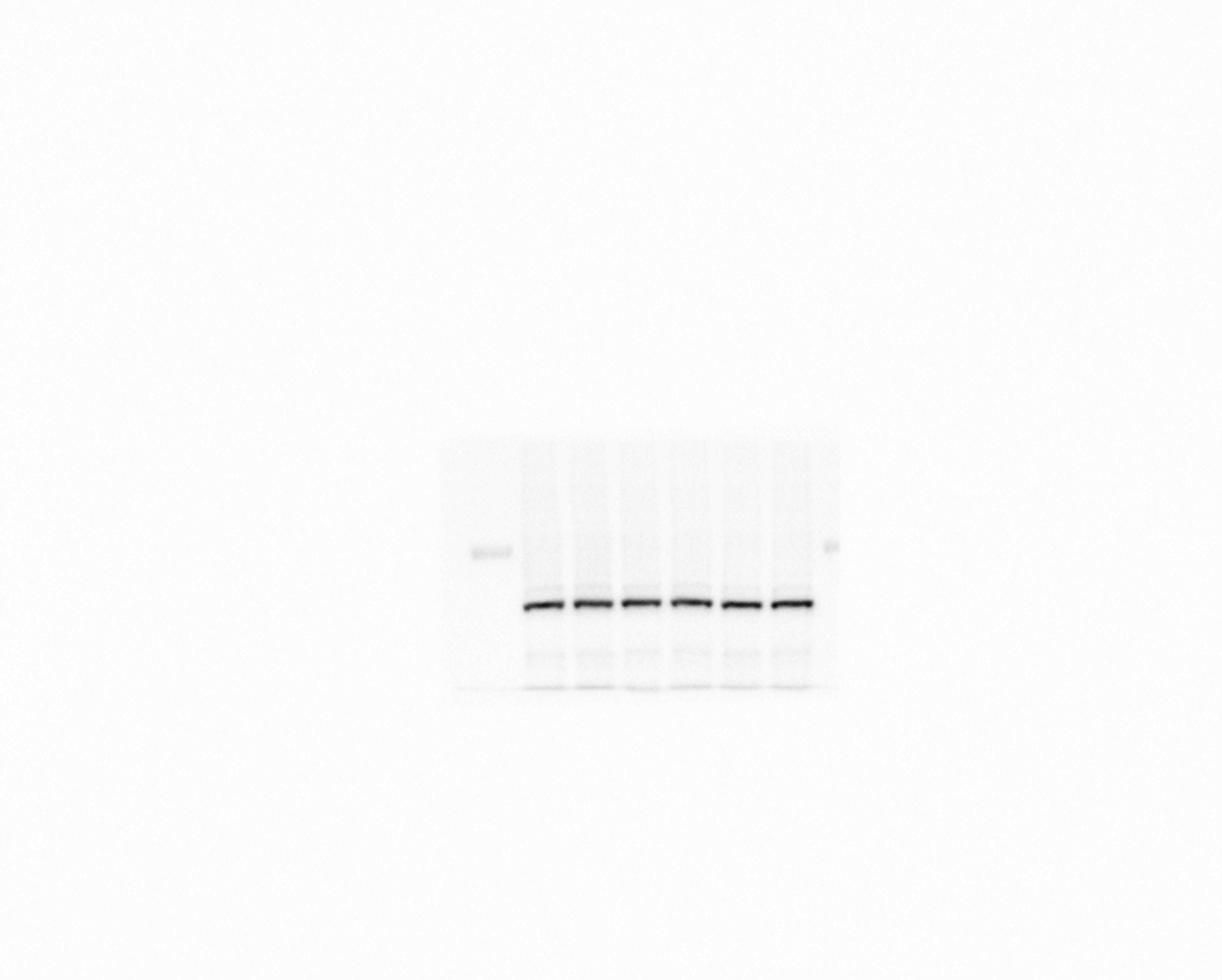

Supplement: Supplementary file 1 [file biomolecules-16-00868-s001.zip › FigureS1 the full, uncropped western blot images/The vivo mice study/β-actin/1-6s.Tif]

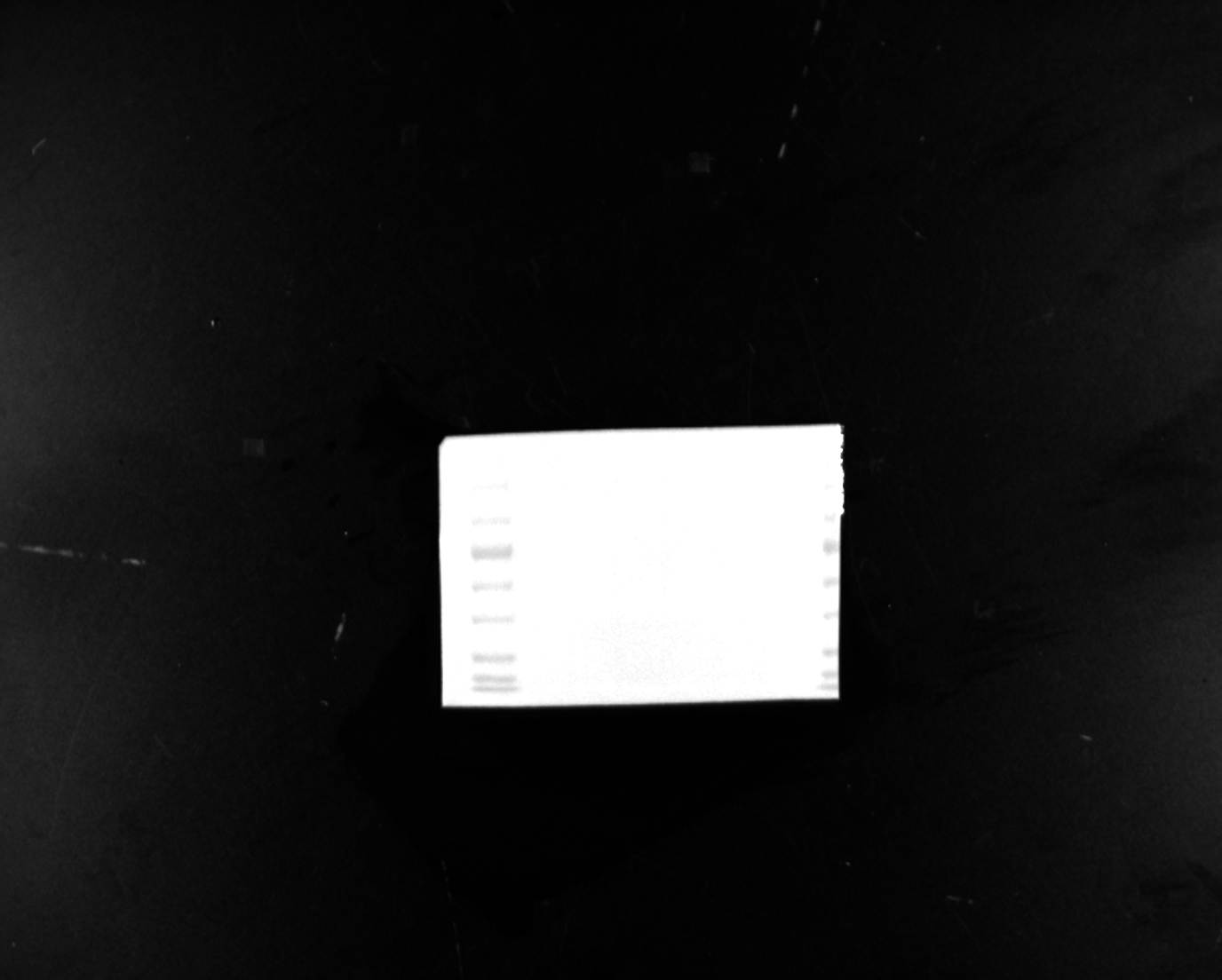

Supplement: Supplementary file 1 [file biomolecules-16-00868-s001.zip › FigureS1 the full, uncropped western blot images/The vivo mice study/β-actin/1-t.Tif]

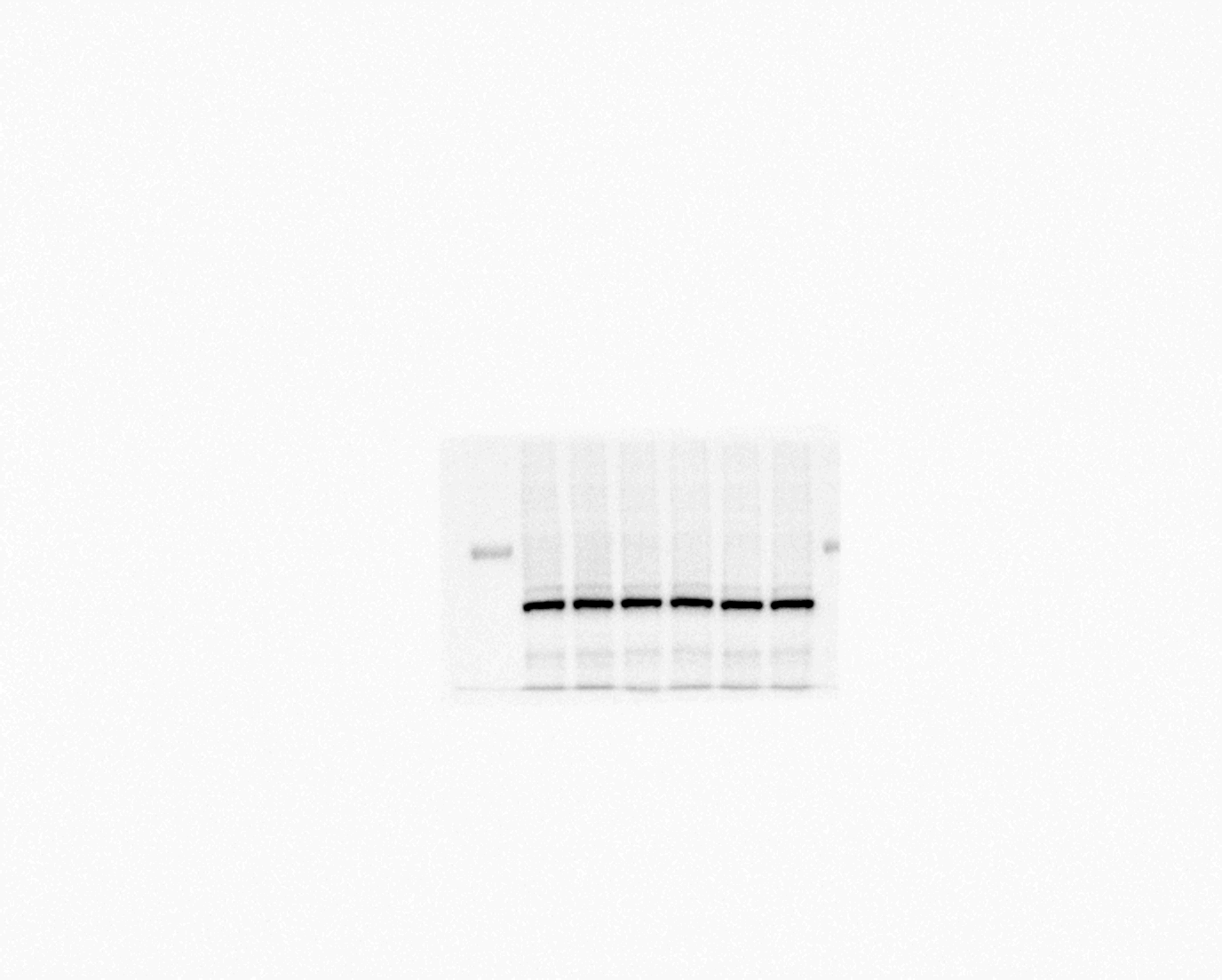

Supplement: Supplementary file 1 [file biomolecules-16-00868-s001.zip › FigureS1 the full, uncropped western blot images/The vivo mice study/β-actin/1.Tif]

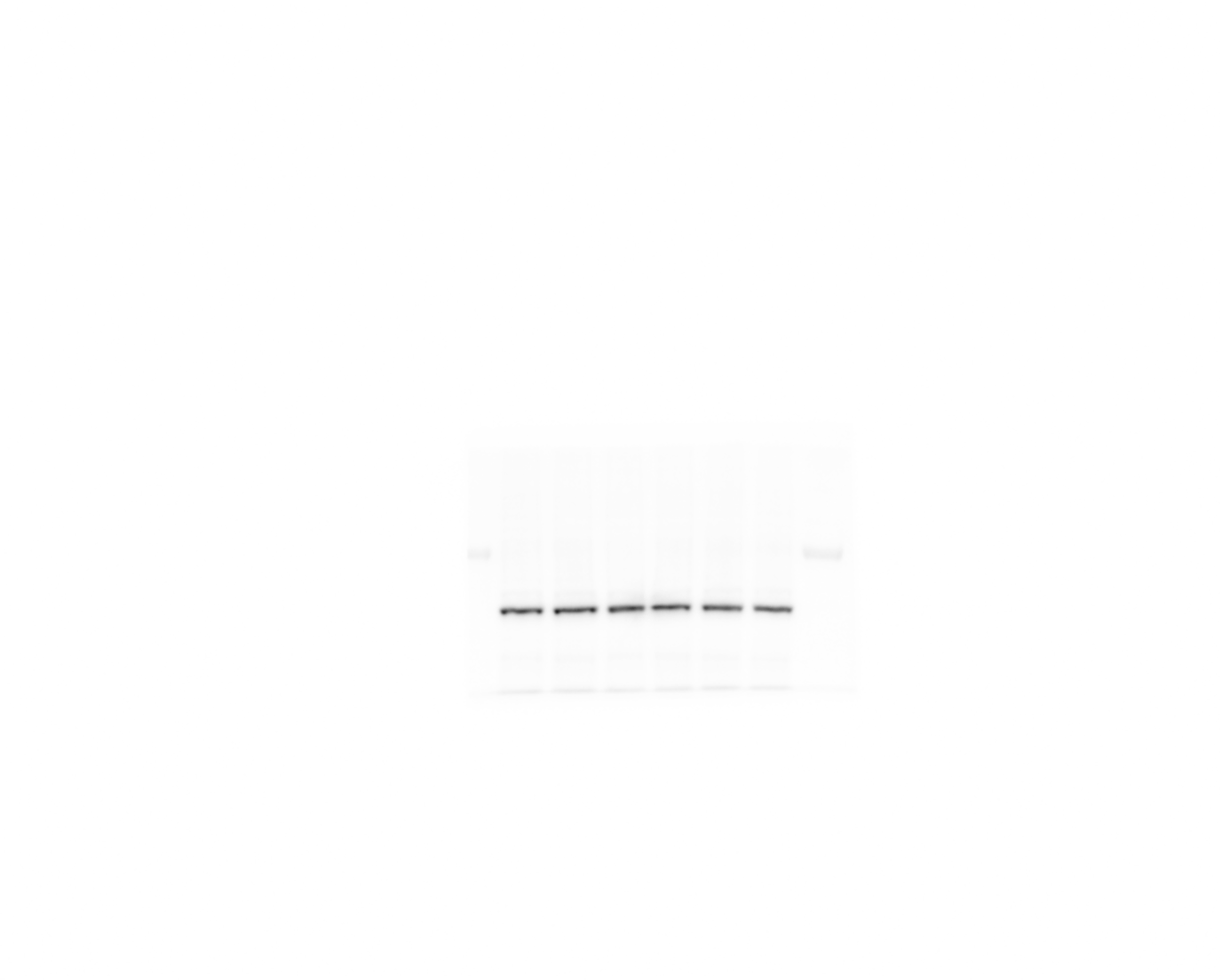

Supplement: Supplementary file 1 [file biomolecules-16-00868-s001.zip › FigureS1 the full, uncropped western blot images/The vivo mice study/β-actin/2-0.3s.Tif]

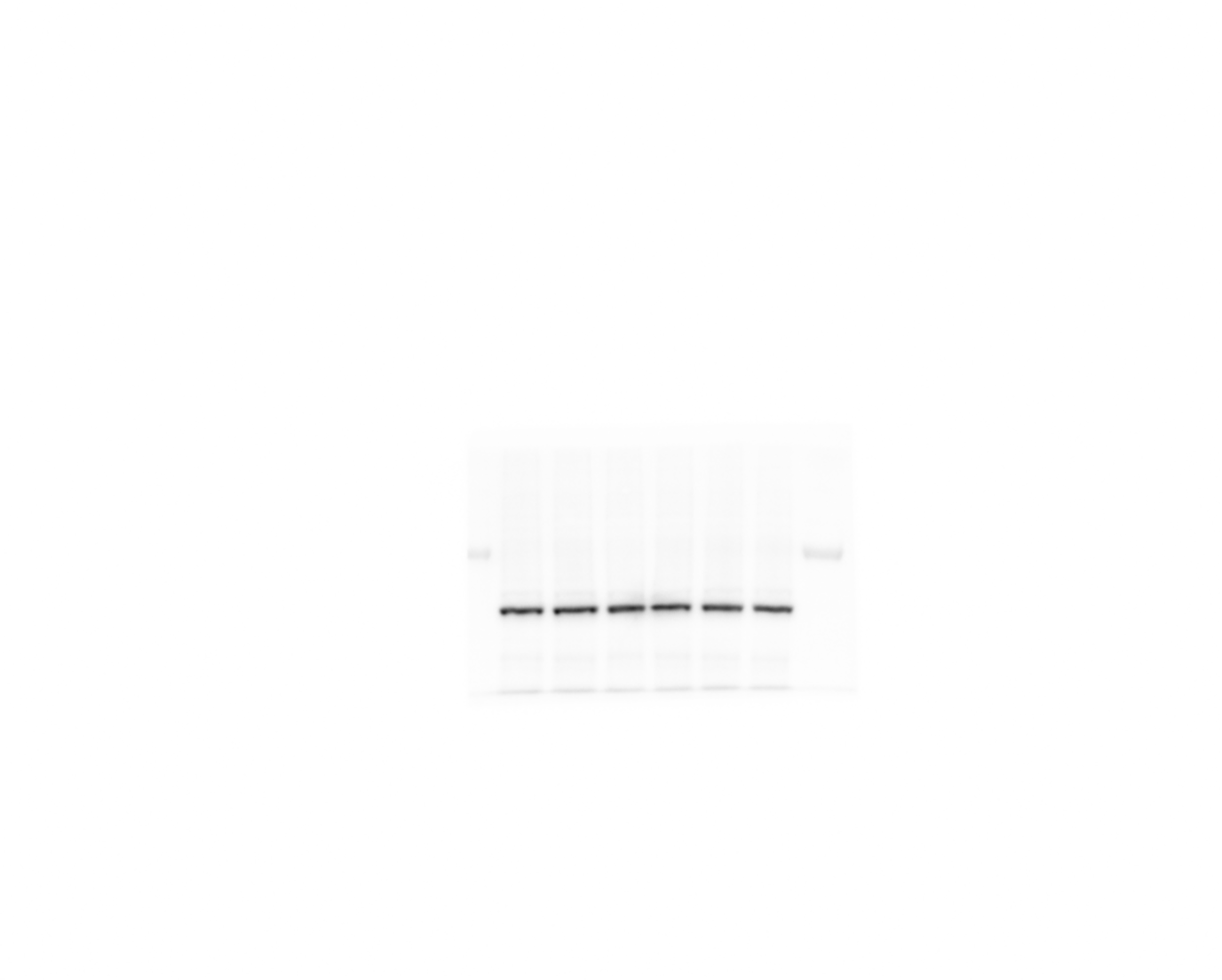

Supplement: Supplementary file 1 [file biomolecules-16-00868-s001.zip › FigureS1 the full, uncropped western blot images/The vivo mice study/β-actin/2-3s.Tif]

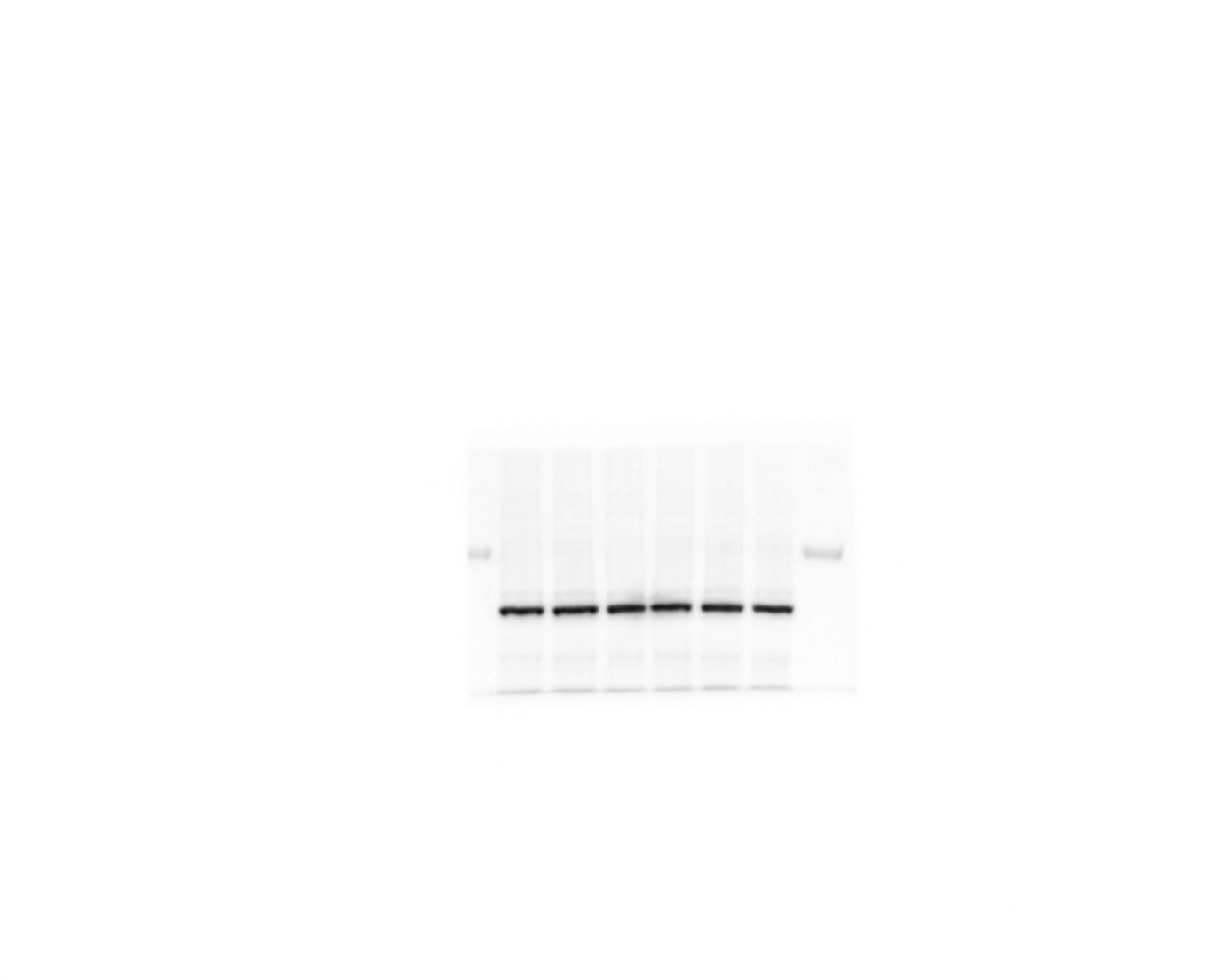

Supplement: Supplementary file 1 [file biomolecules-16-00868-s001.zip › FigureS1 the full, uncropped western blot images/The vivo mice study/β-actin/2-6s.Tif]

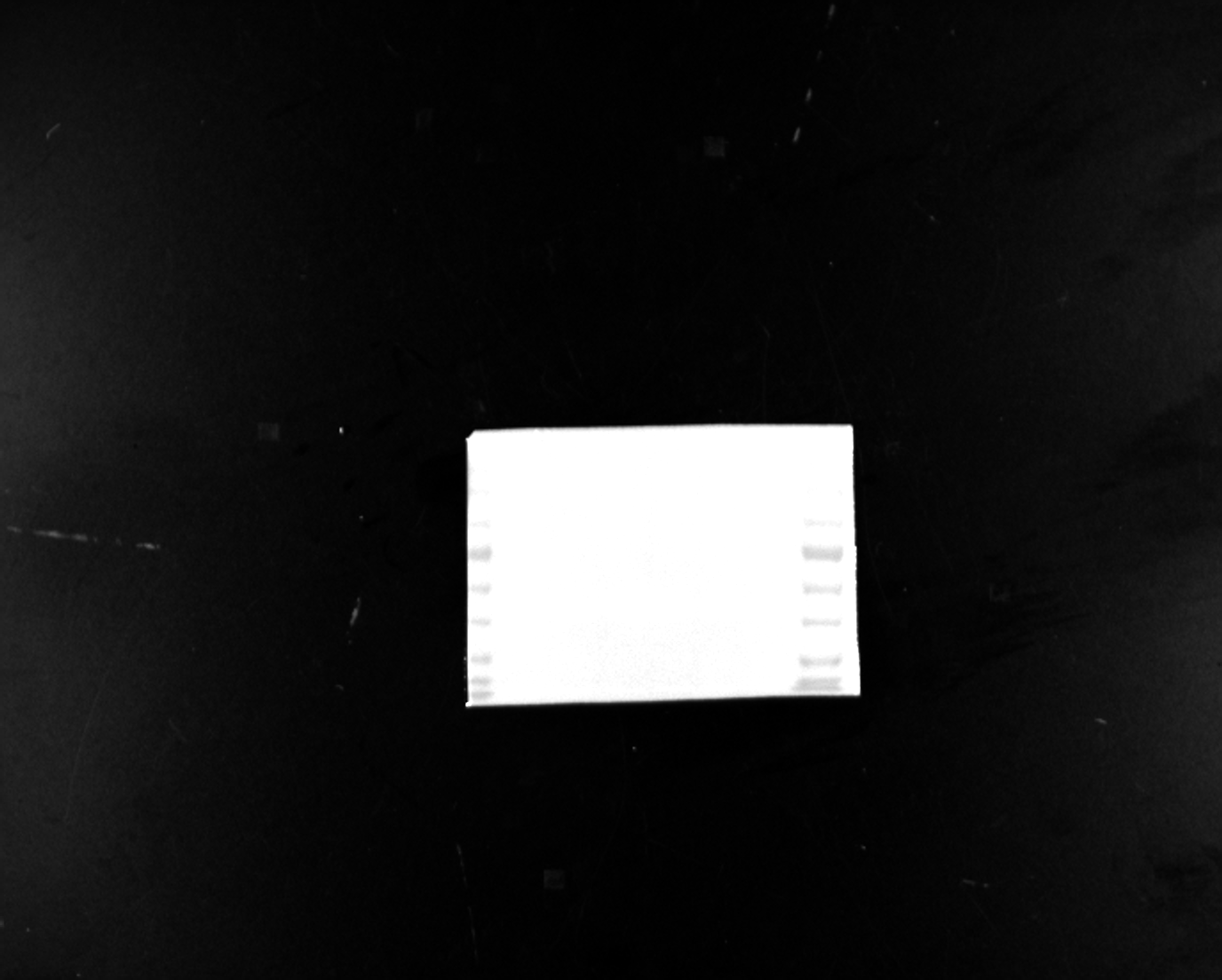

Supplement: Supplementary file 1 [file biomolecules-16-00868-s001.zip › FigureS1 the full, uncropped western blot images/The vivo mice study/β-actin/2-t.Tif]

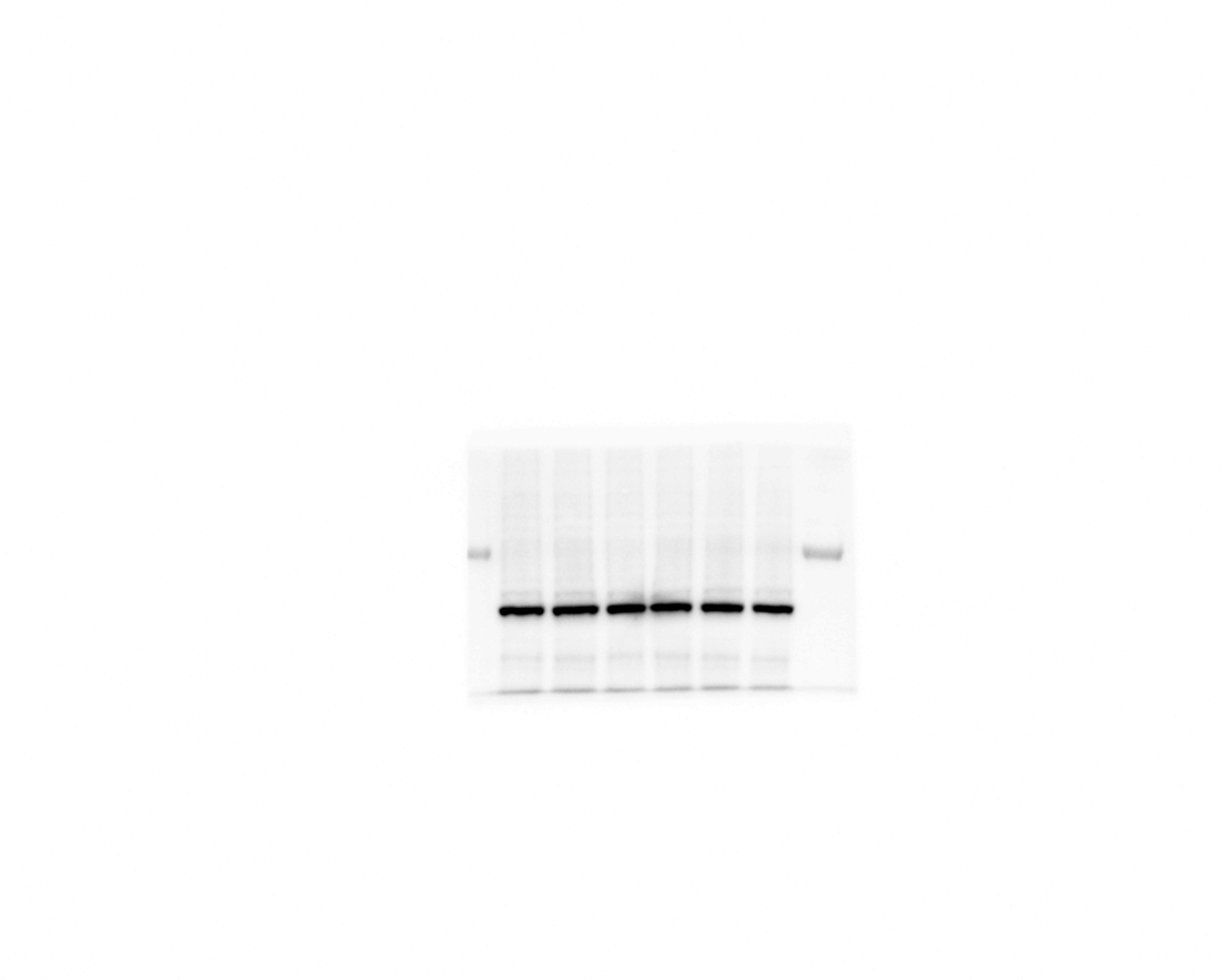

Supplement: Supplementary file 1 [file biomolecules-16-00868-s001.zip › FigureS1 the full, uncropped western blot images/The vivo mice study/β-actin/2.Tif]
