# Supplementary figures and images for: Dysregulation of the HSF1-Mediated UPRmt Pathway in Colonic Smooth Muscle Cells Drives Motility Dysfunction in Functional Constipation (part 1 of 2)
Source: Biomolecules. 2026 Jun 12;16(6):868. doi: 10.3390/biom16060868 (PMC13296460; doi:10.3390/biom16060868)

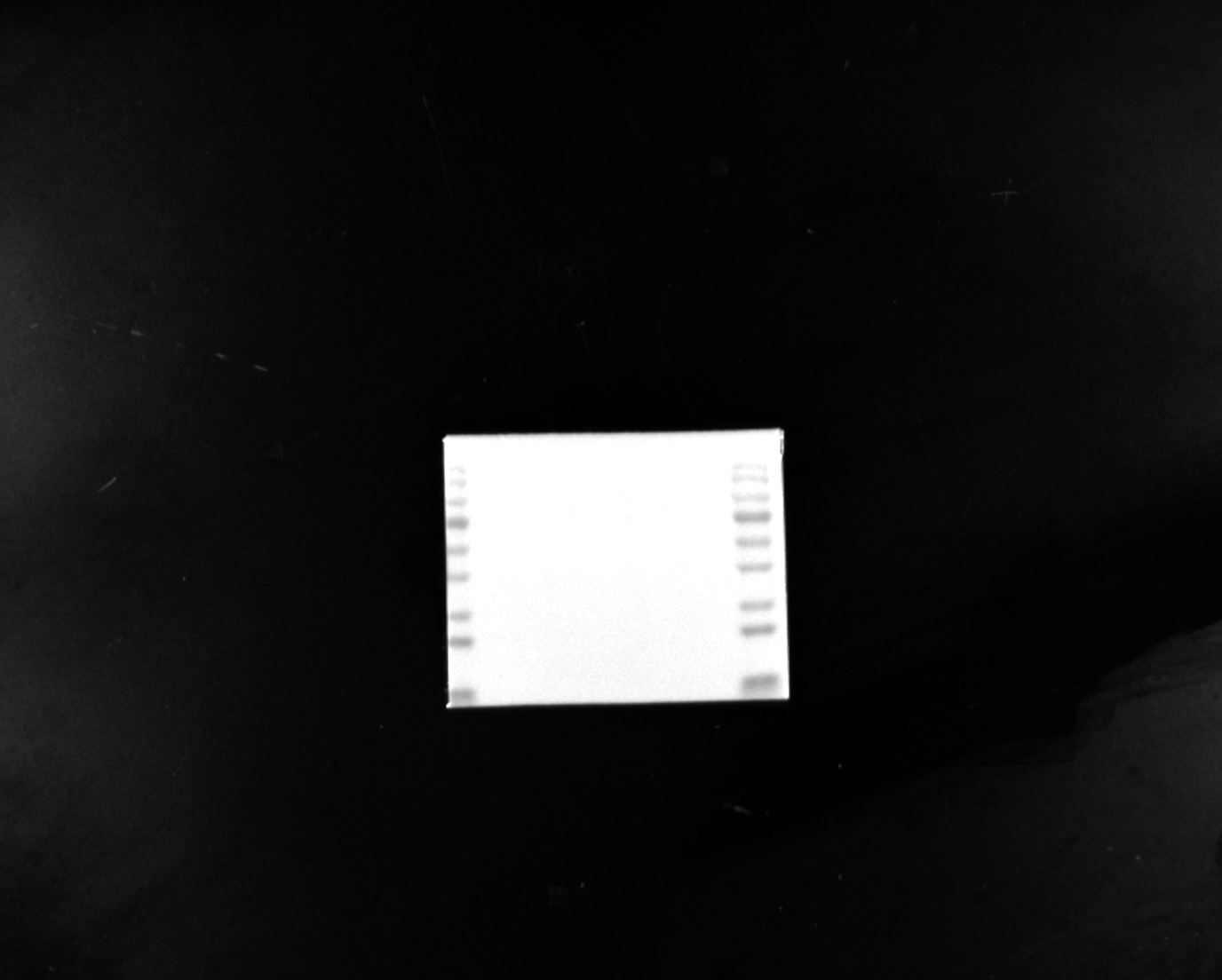

Supplement: Supplementary file 1 [file biomolecules-16-00868-s001.zip › FigureS1 the full, uncropped western blot images/The vitro primary SMCs/ATP5A/1-t.Tif]

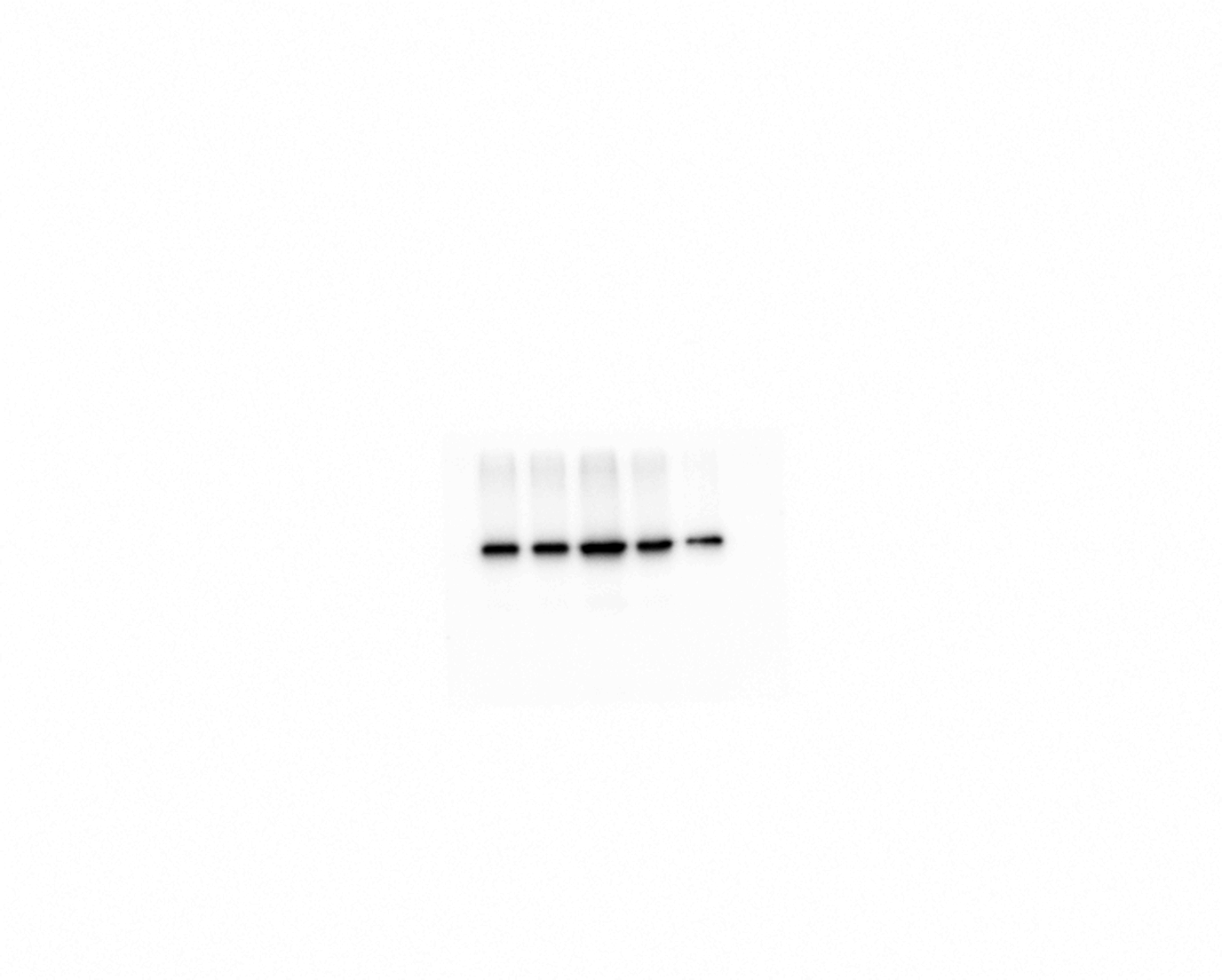

Supplement: Supplementary file 1 [file biomolecules-16-00868-s001.zip › FigureS1 the full, uncropped western blot images/The vitro primary SMCs/ATP5A/1.Tif]

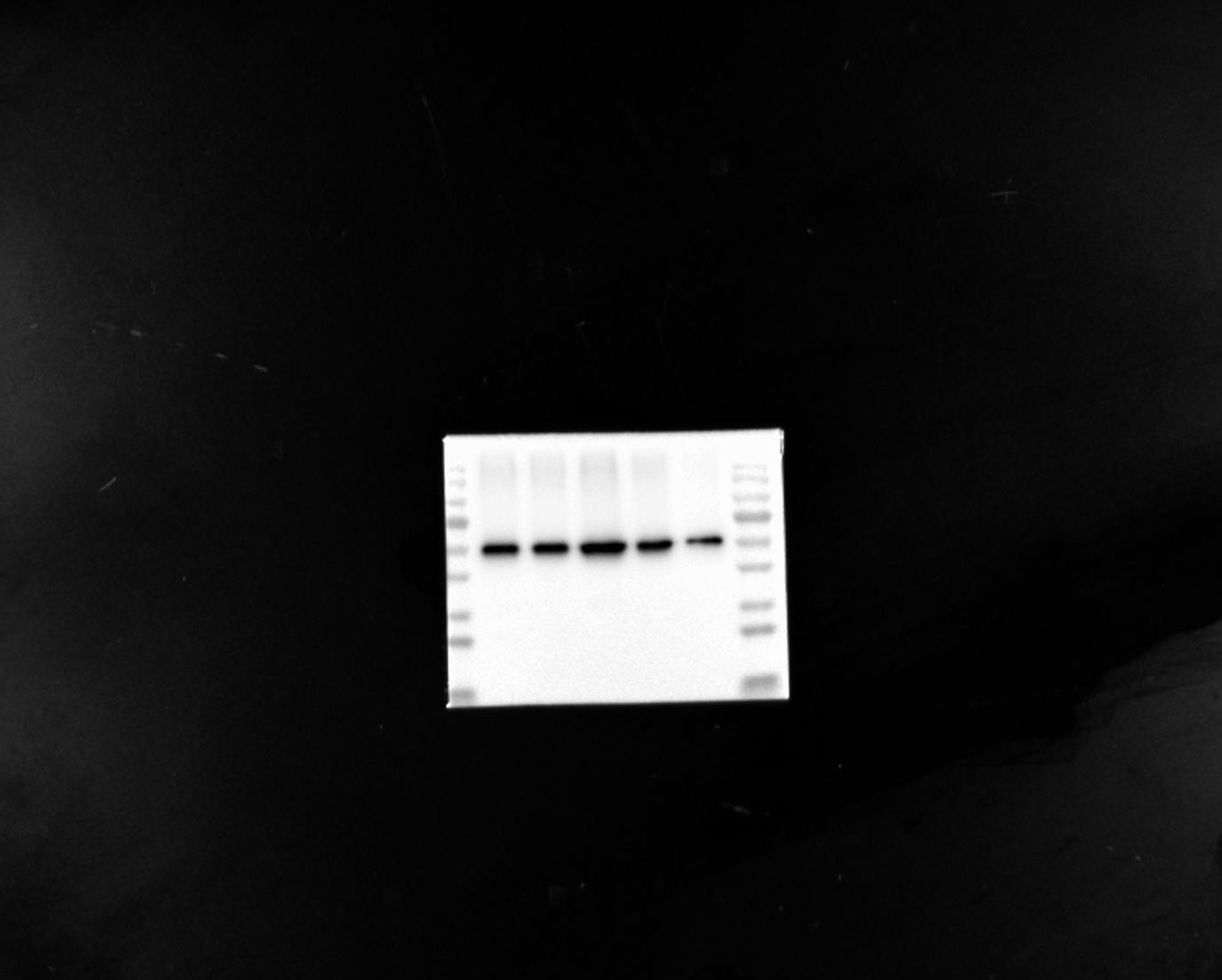

Supplement: Supplementary file 1 [file biomolecules-16-00868-s001.zip › FigureS1 the full, uncropped western blot images/The vitro primary SMCs/ATP5A/1副本.Tif]

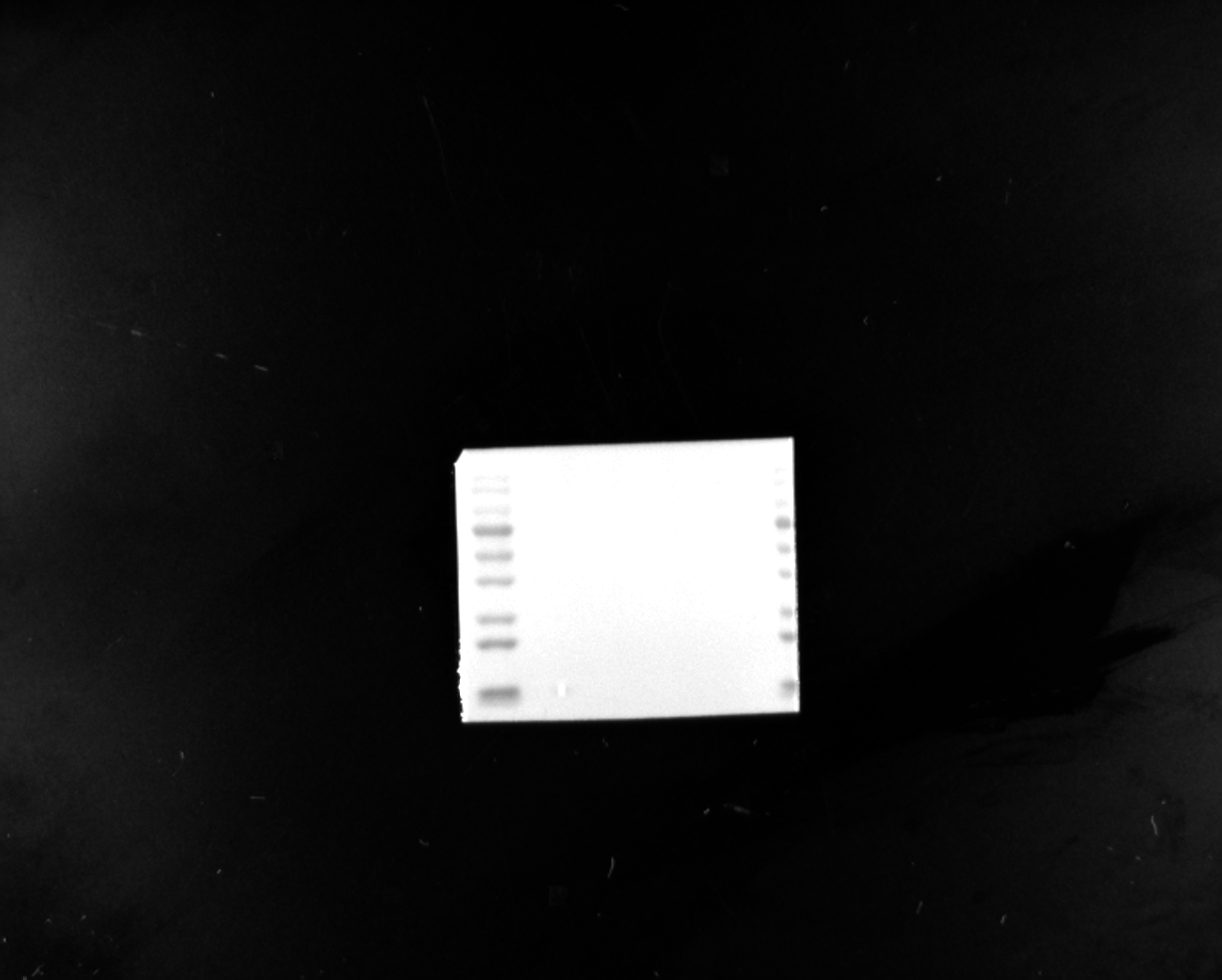

Supplement: Supplementary file 1 [file biomolecules-16-00868-s001.zip › FigureS1 the full, uncropped western blot images/The vitro primary SMCs/ATP5A/2-t.Tif]

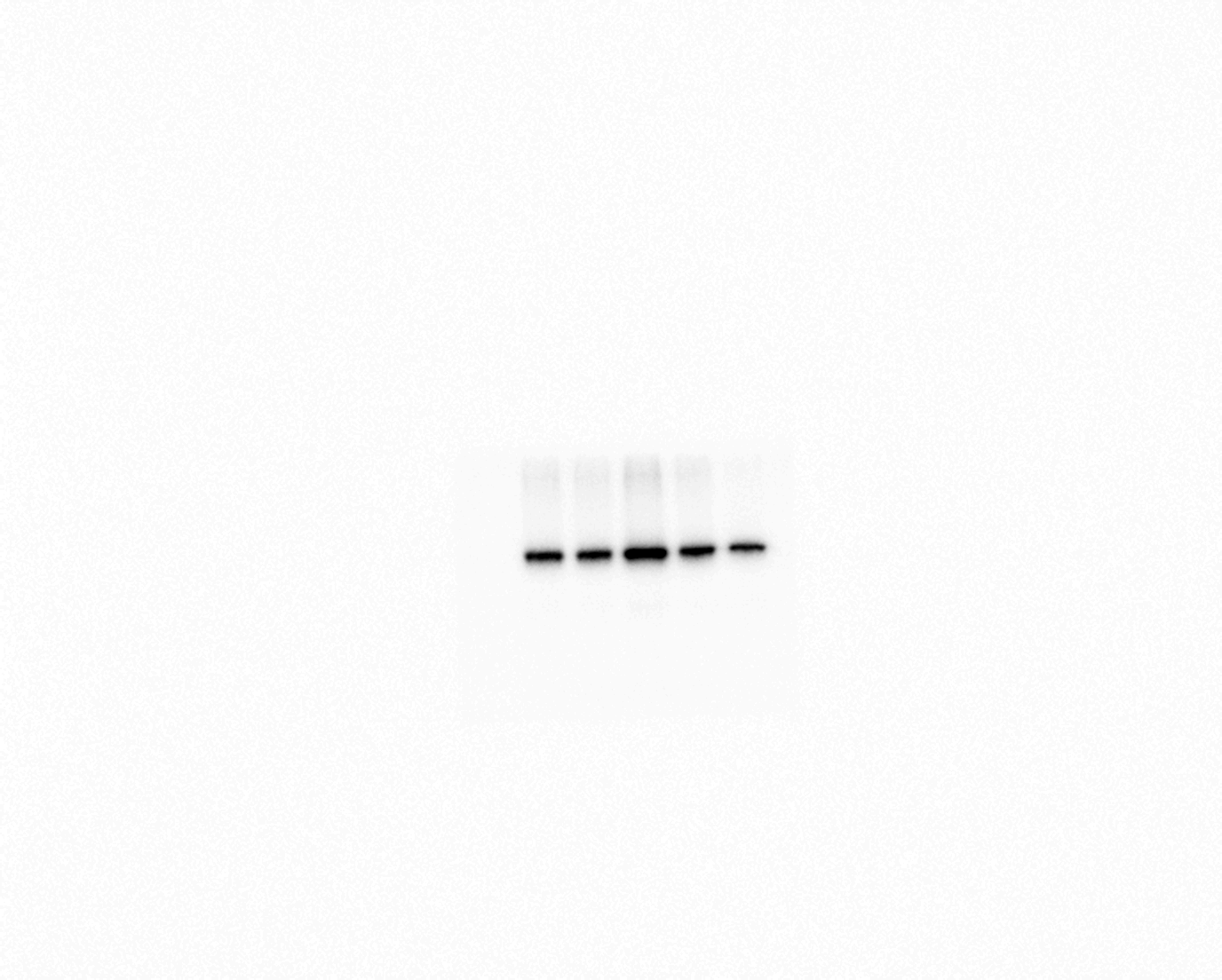

Supplement: Supplementary file 1 [file biomolecules-16-00868-s001.zip › FigureS1 the full, uncropped western blot images/The vitro primary SMCs/ATP5A/2.Tif]

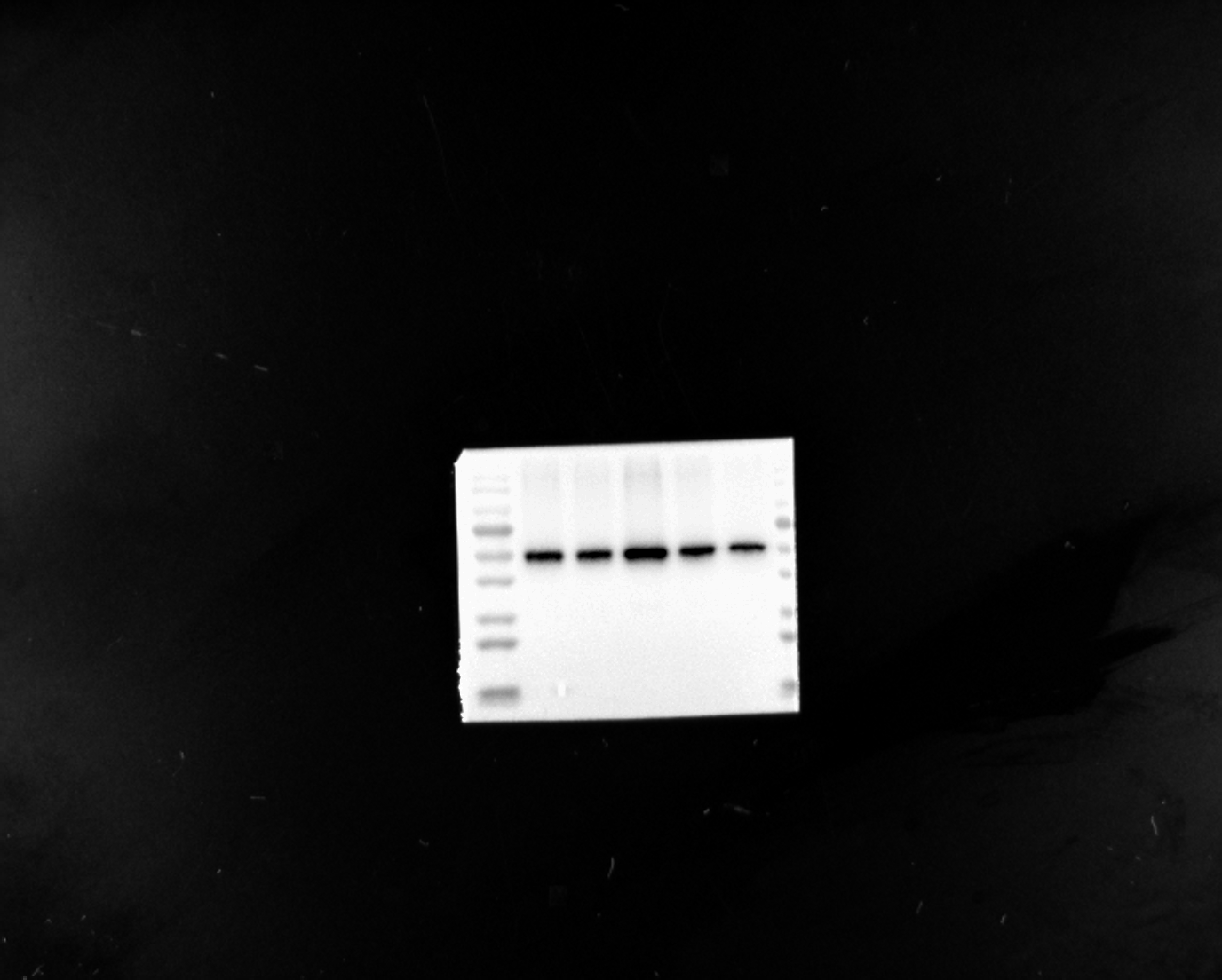

Supplement: Supplementary file 1 [file biomolecules-16-00868-s001.zip › FigureS1 the full, uncropped western blot images/The vitro primary SMCs/ATP5A/2副本.Tif]

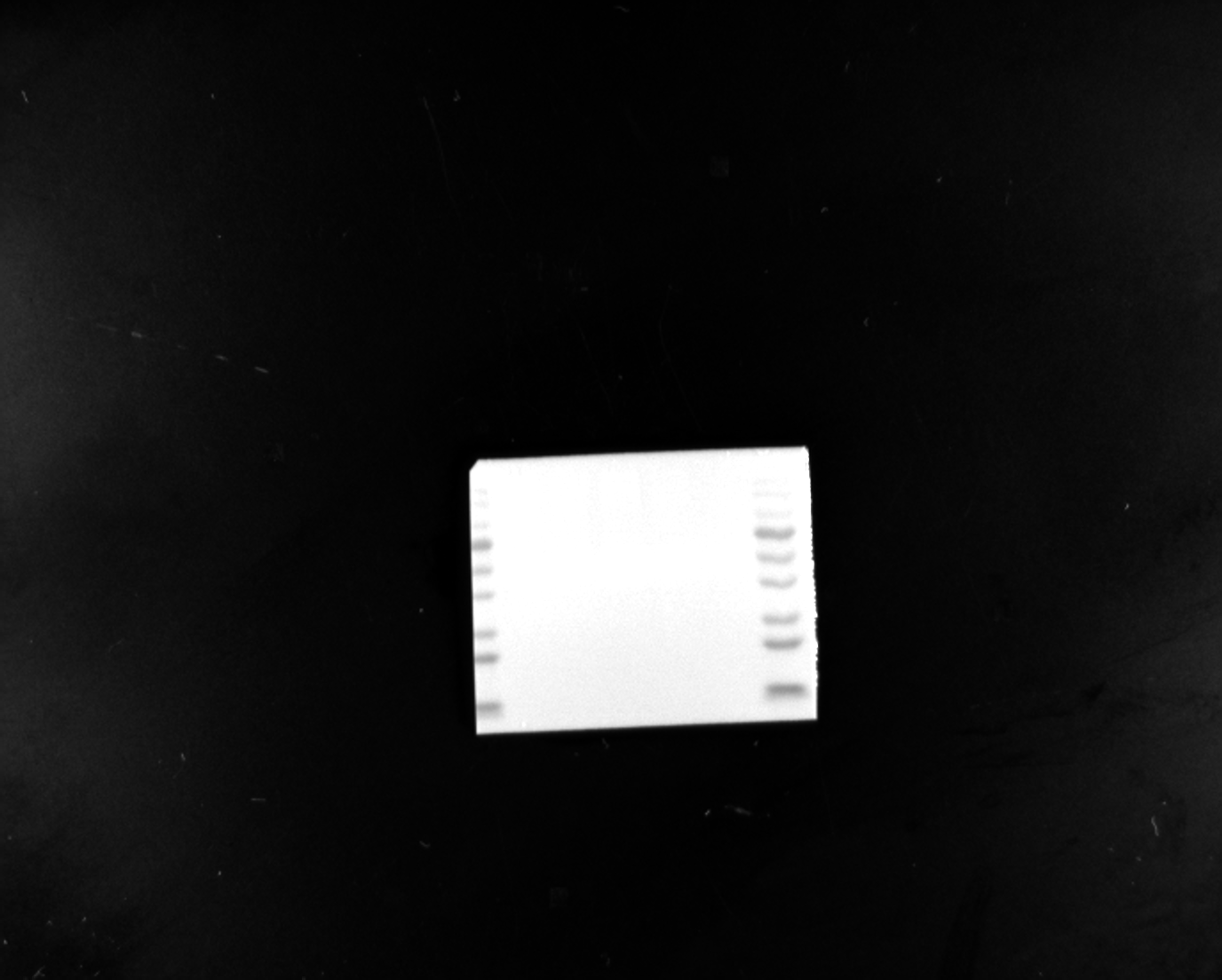

Supplement: Supplementary file 1 [file biomolecules-16-00868-s001.zip › FigureS1 the full, uncropped western blot images/The vitro primary SMCs/ATP5A/3-t.Tif]

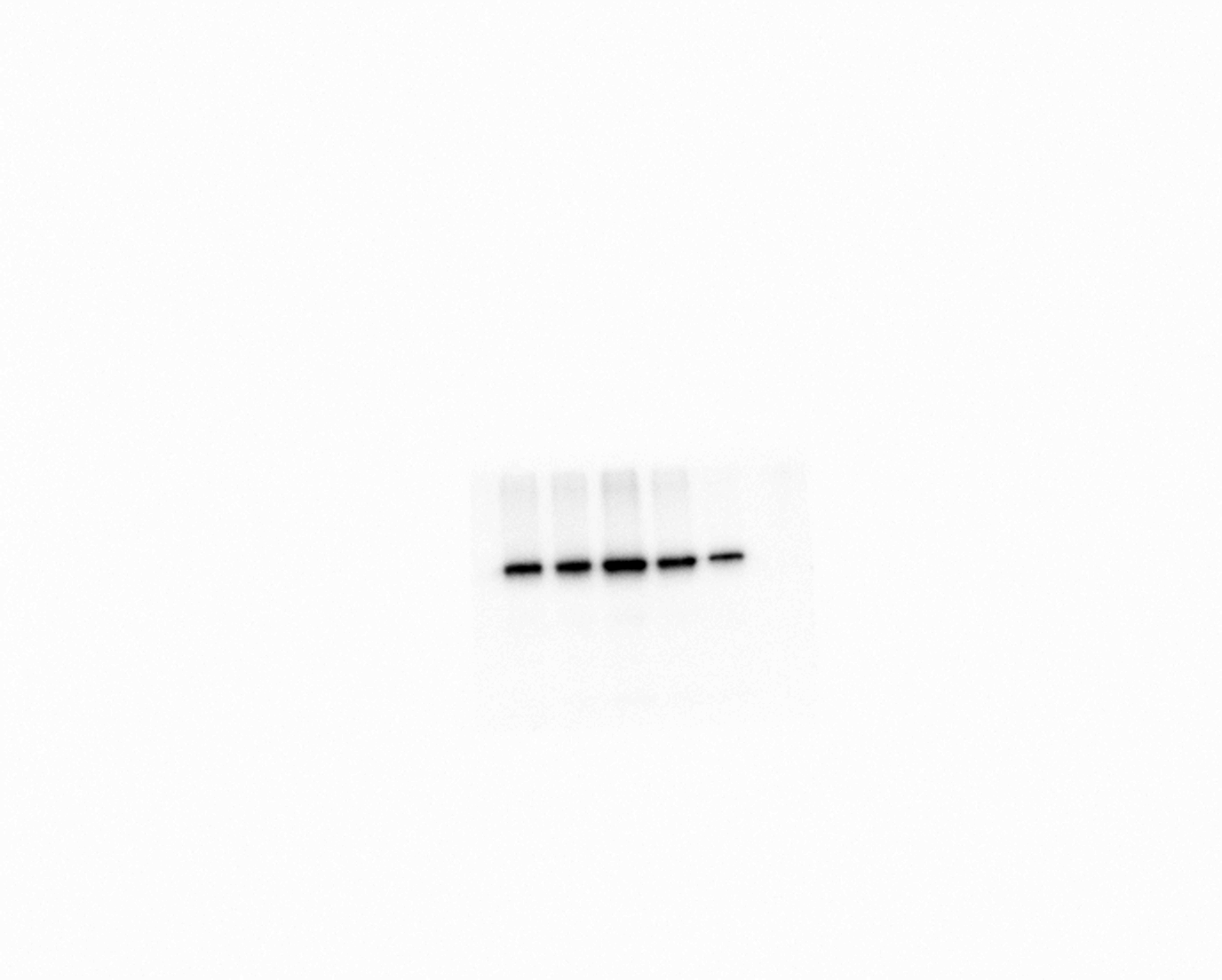

Supplement: Supplementary file 1 [file biomolecules-16-00868-s001.zip › FigureS1 the full, uncropped western blot images/The vitro primary SMCs/ATP5A/3.Tif]

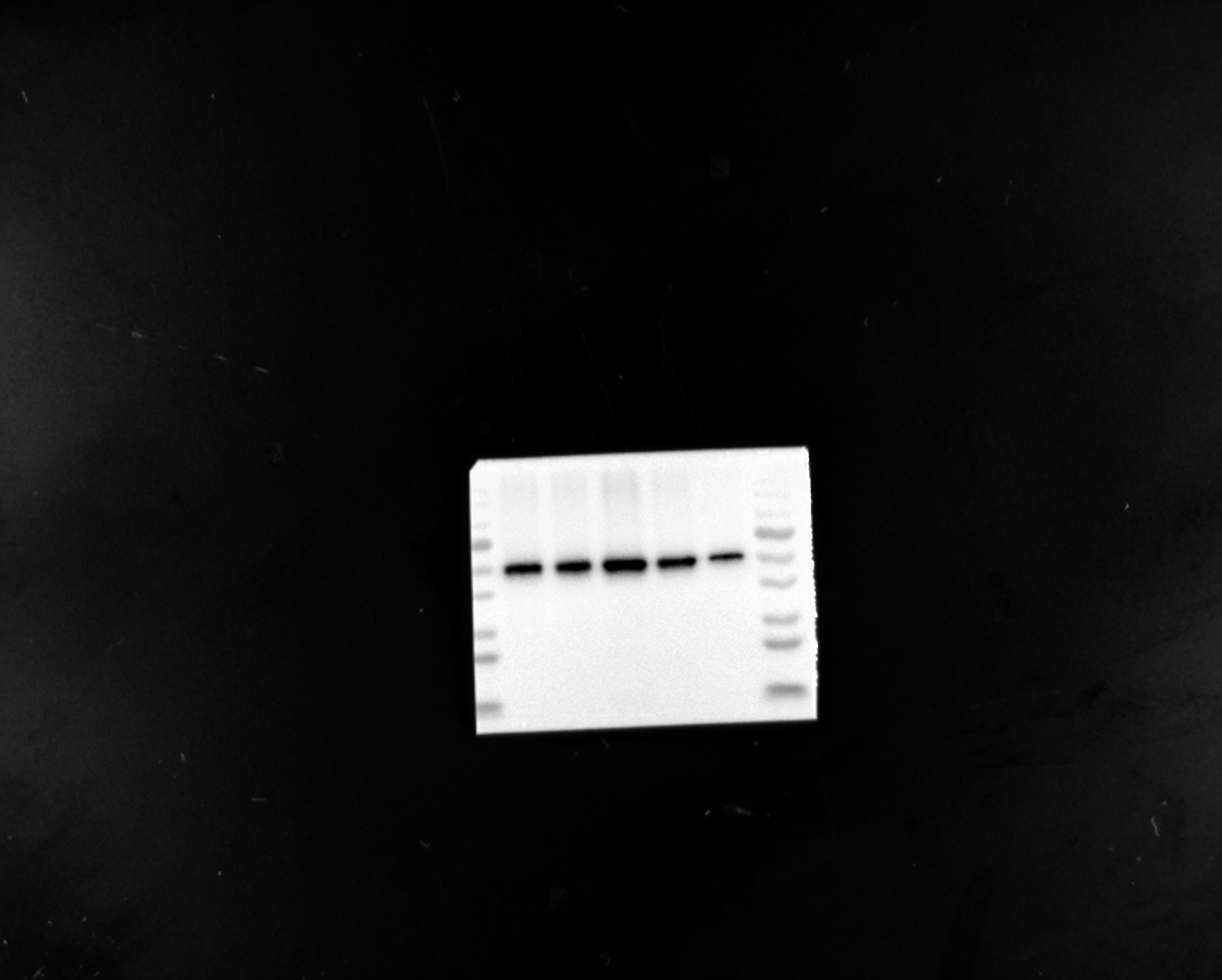

Supplement: Supplementary file 1 [file biomolecules-16-00868-s001.zip › FigureS1 the full, uncropped western blot images/The vitro primary SMCs/ATP5A/3副本.Tif]

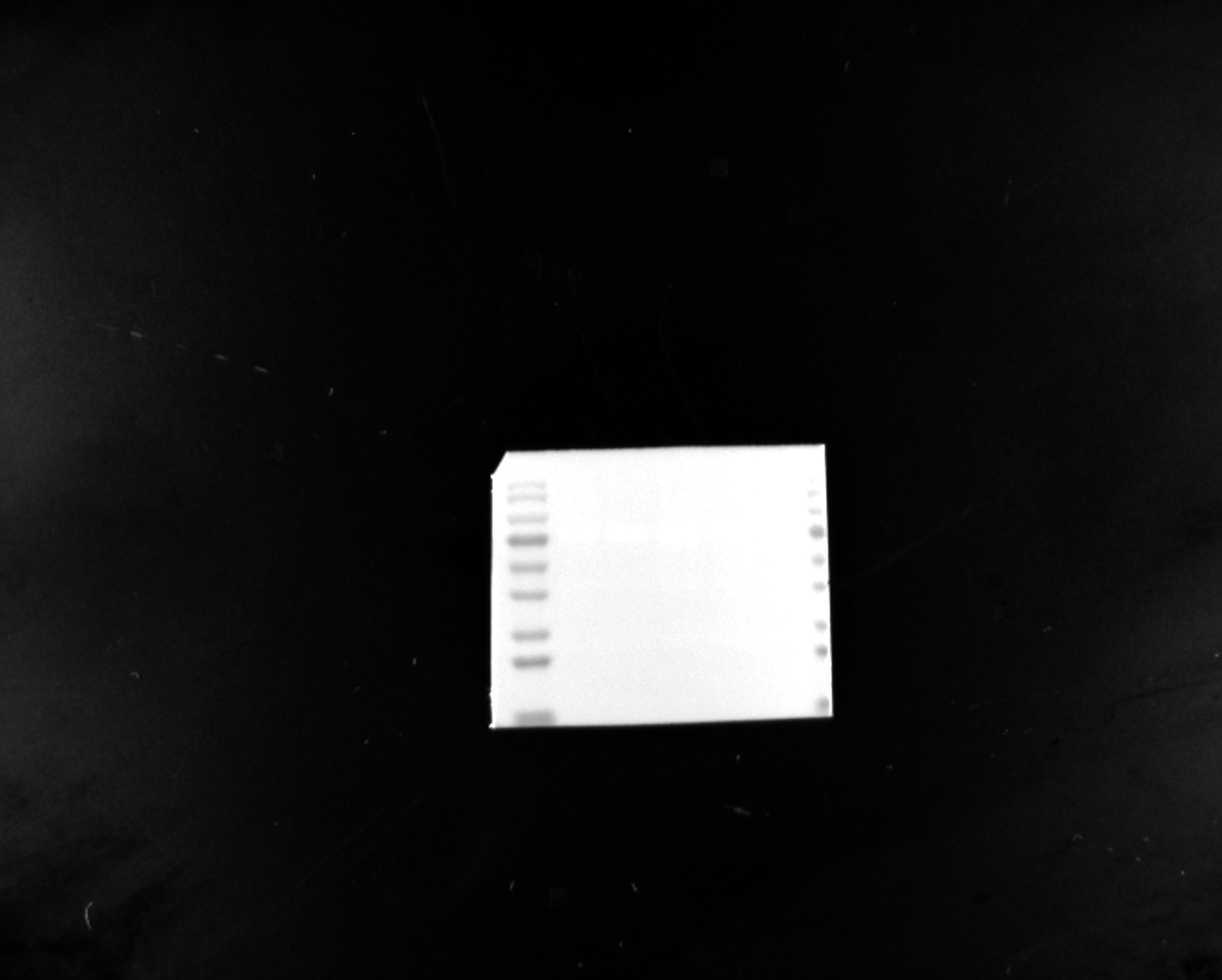

Supplement: Supplementary file 1 [file biomolecules-16-00868-s001.zip › FigureS1 the full, uncropped western blot images/The vitro primary SMCs/COX1/1-t.Tif]

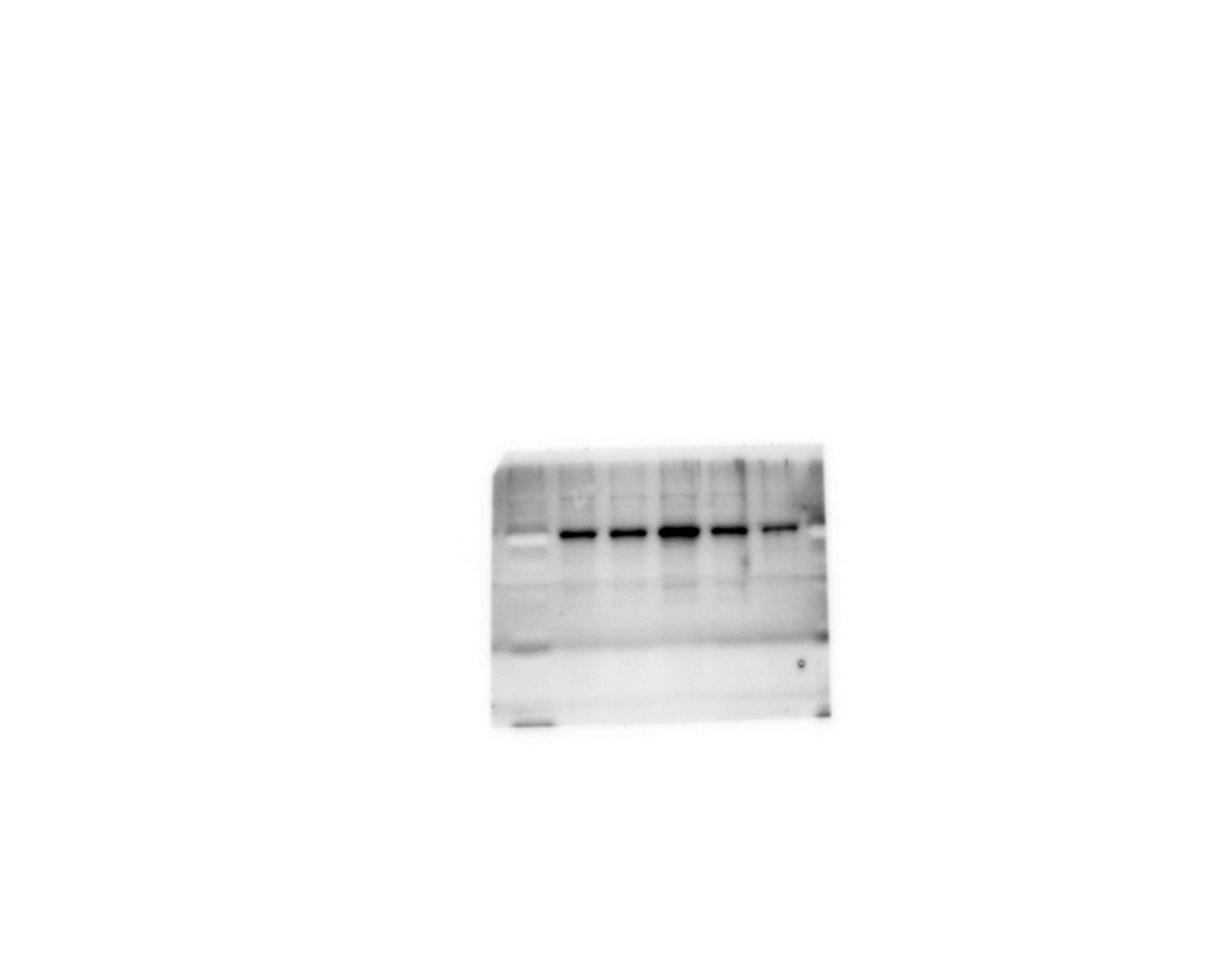

Supplement: Supplementary file 1 [file biomolecules-16-00868-s001.zip › FigureS1 the full, uncropped western blot images/The vitro primary SMCs/COX1/1.Tif]

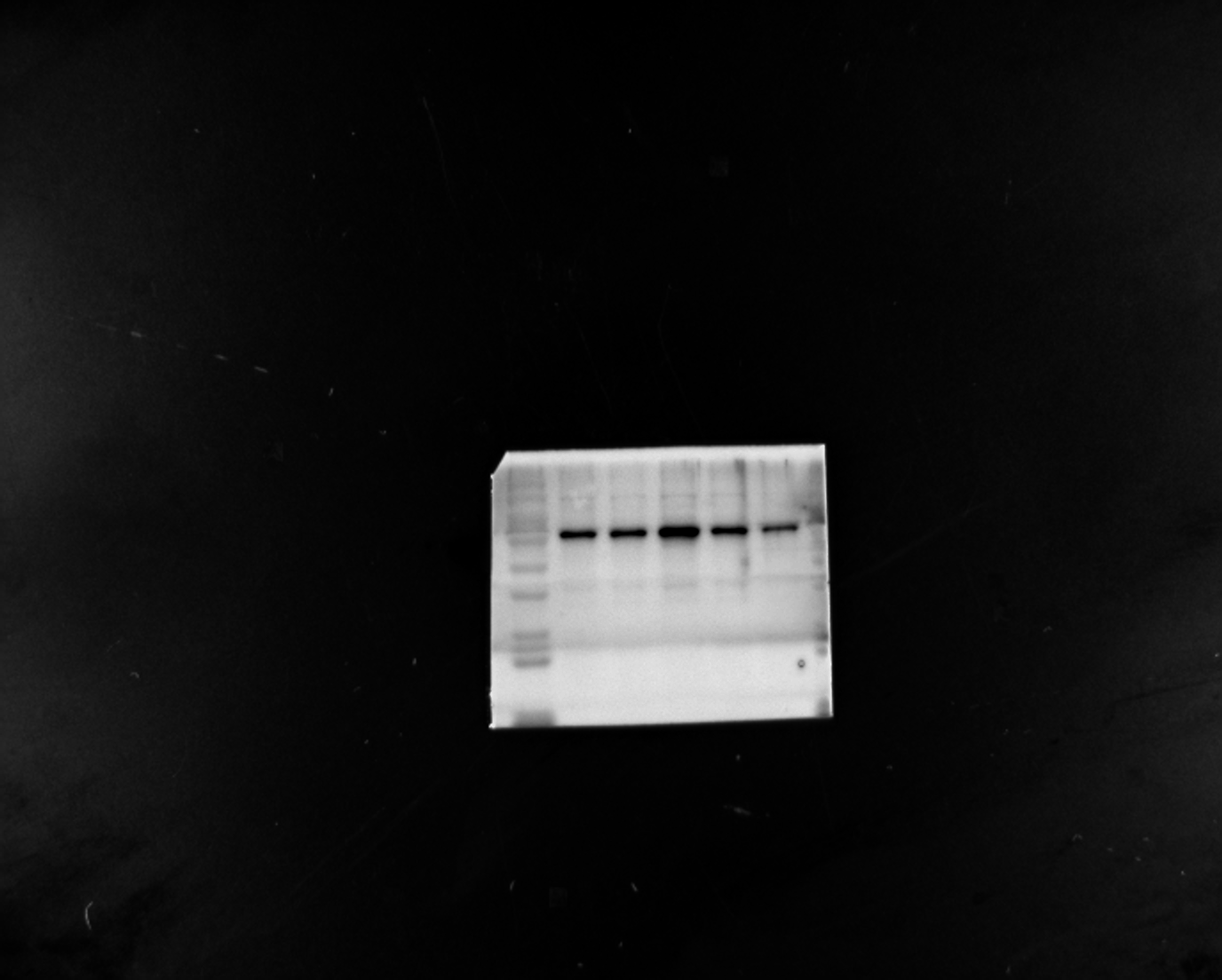

Supplement: Supplementary file 1 [file biomolecules-16-00868-s001.zip › FigureS1 the full, uncropped western blot images/The vitro primary SMCs/COX1/1副本.Tif]

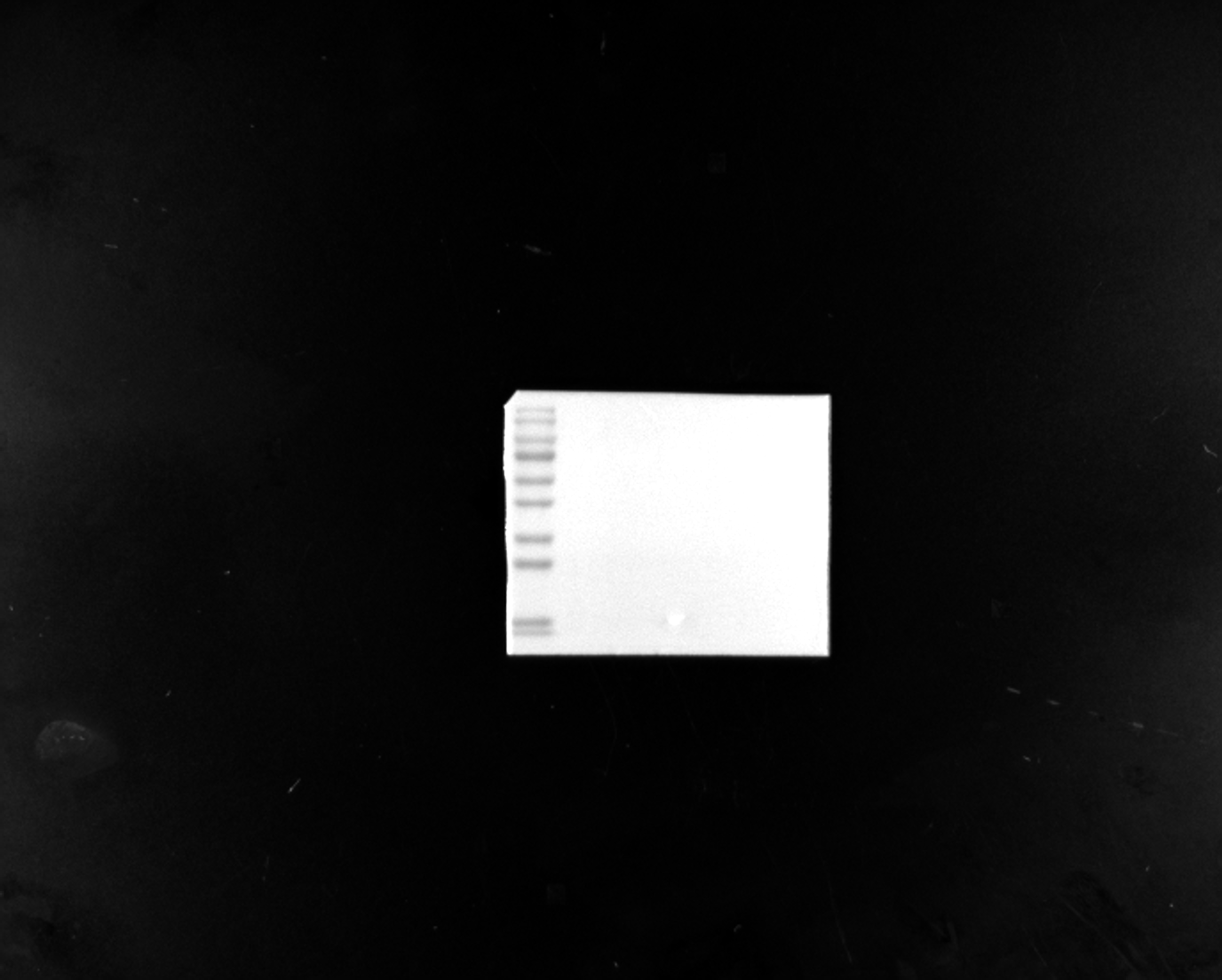

Supplement: Supplementary file 1 [file biomolecules-16-00868-s001.zip › FigureS1 the full, uncropped western blot images/The vitro primary SMCs/COX1/2-t.Tif]

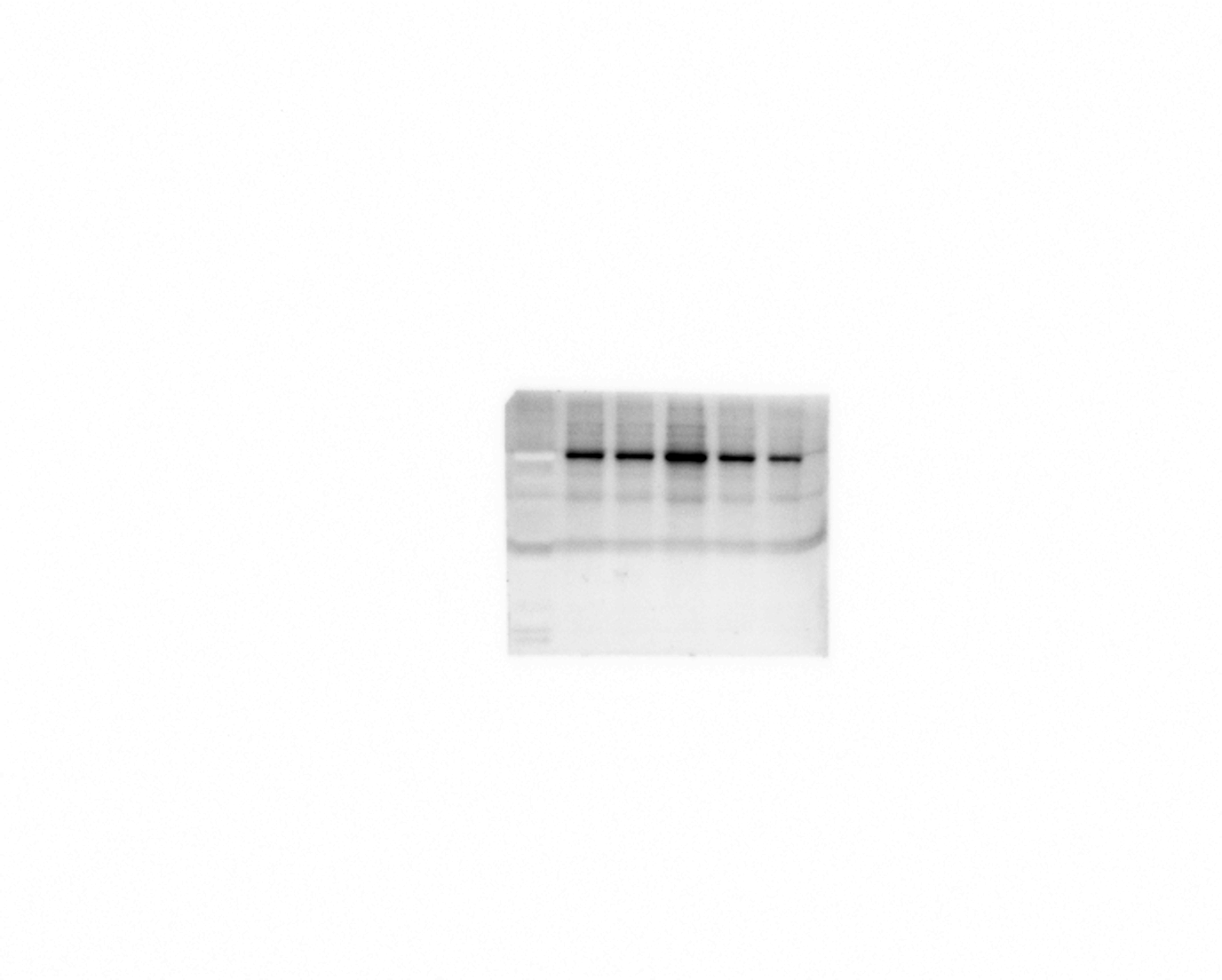

Supplement: Supplementary file 1 [file biomolecules-16-00868-s001.zip › FigureS1 the full, uncropped western blot images/The vitro primary SMCs/COX1/2.Tif]

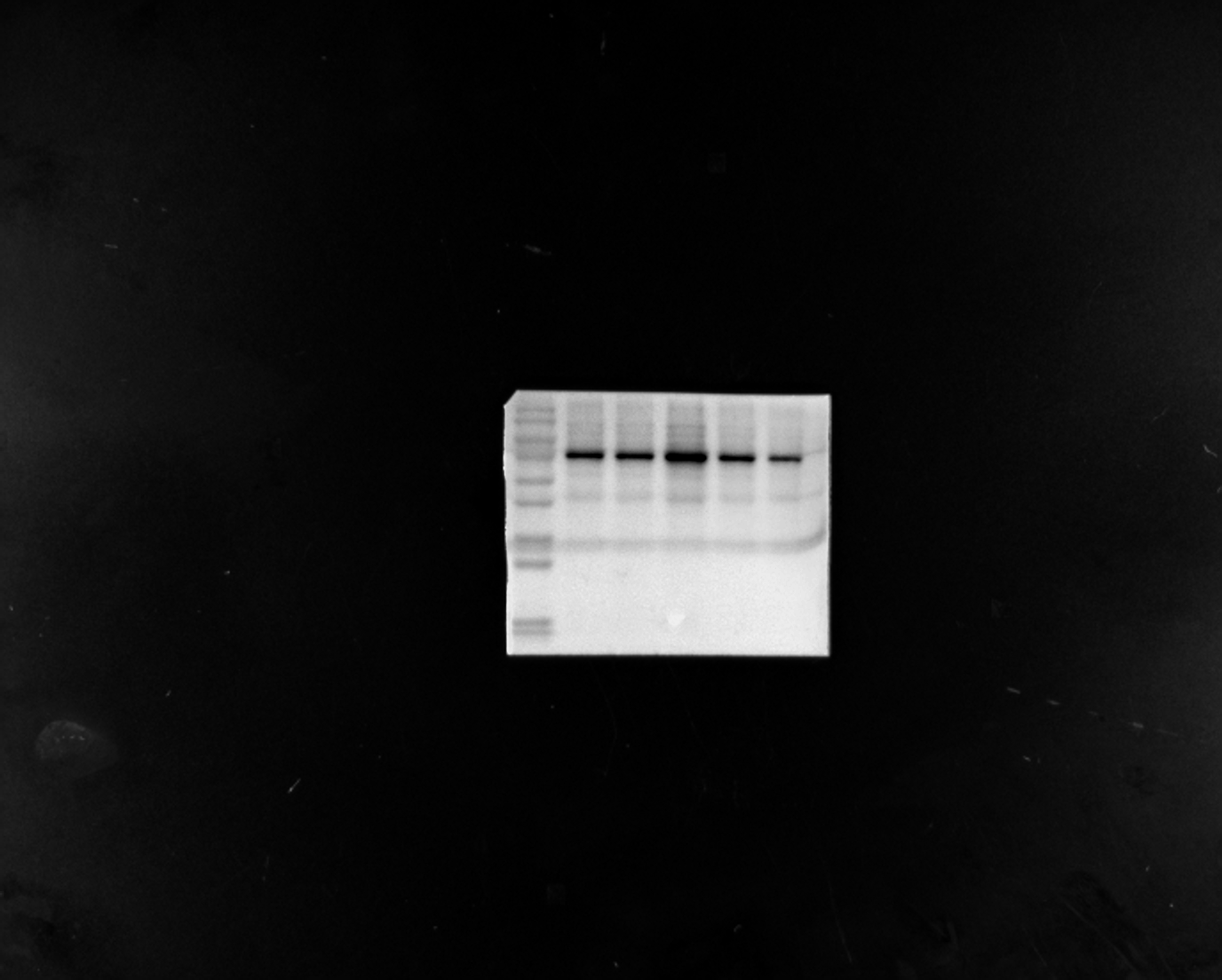

Supplement: Supplementary file 1 [file biomolecules-16-00868-s001.zip › FigureS1 the full, uncropped western blot images/The vitro primary SMCs/COX1/2副本.Tif]

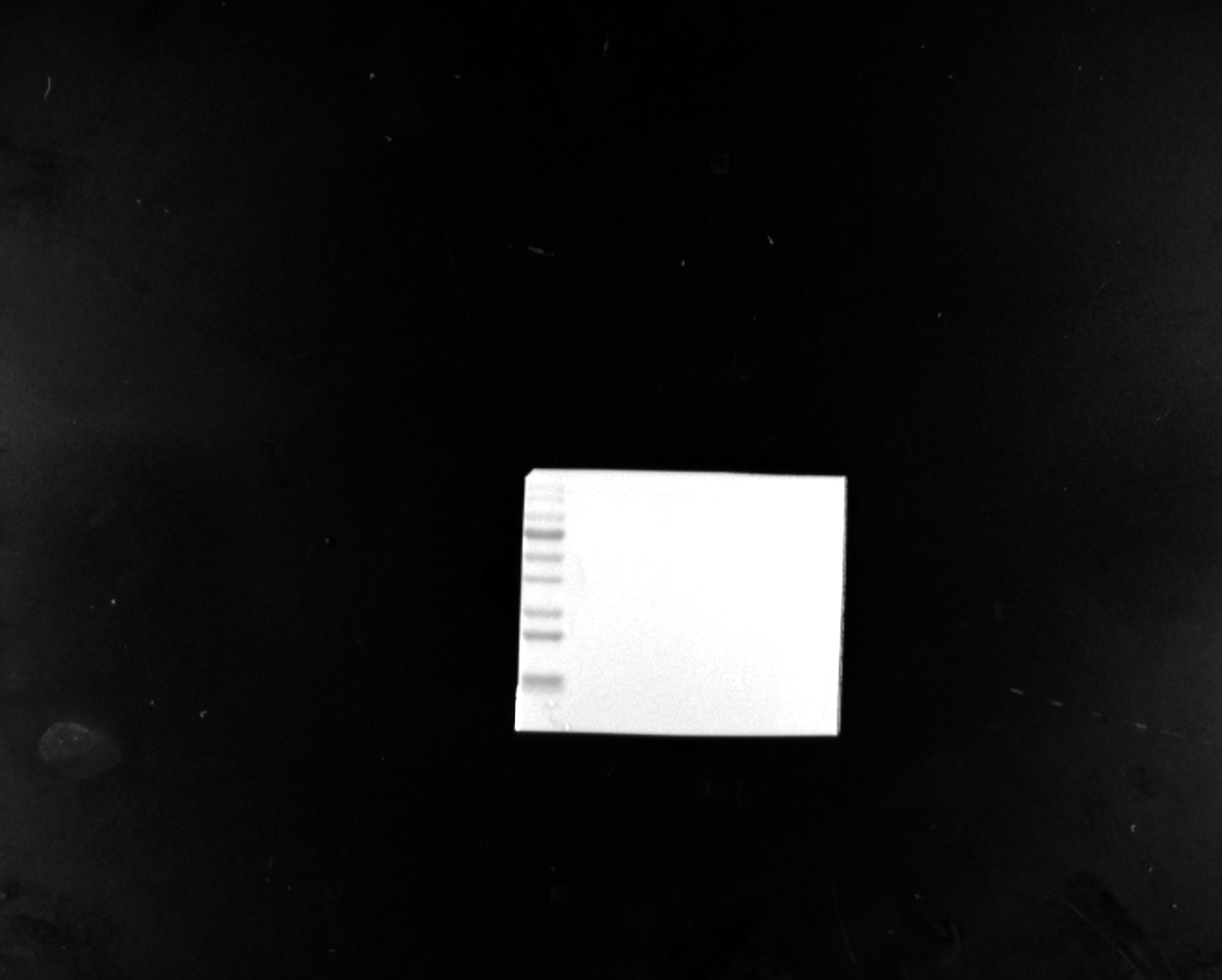

Supplement: Supplementary file 1 [file biomolecules-16-00868-s001.zip › FigureS1 the full, uncropped western blot images/The vitro primary SMCs/COX1/3-t.Tif]

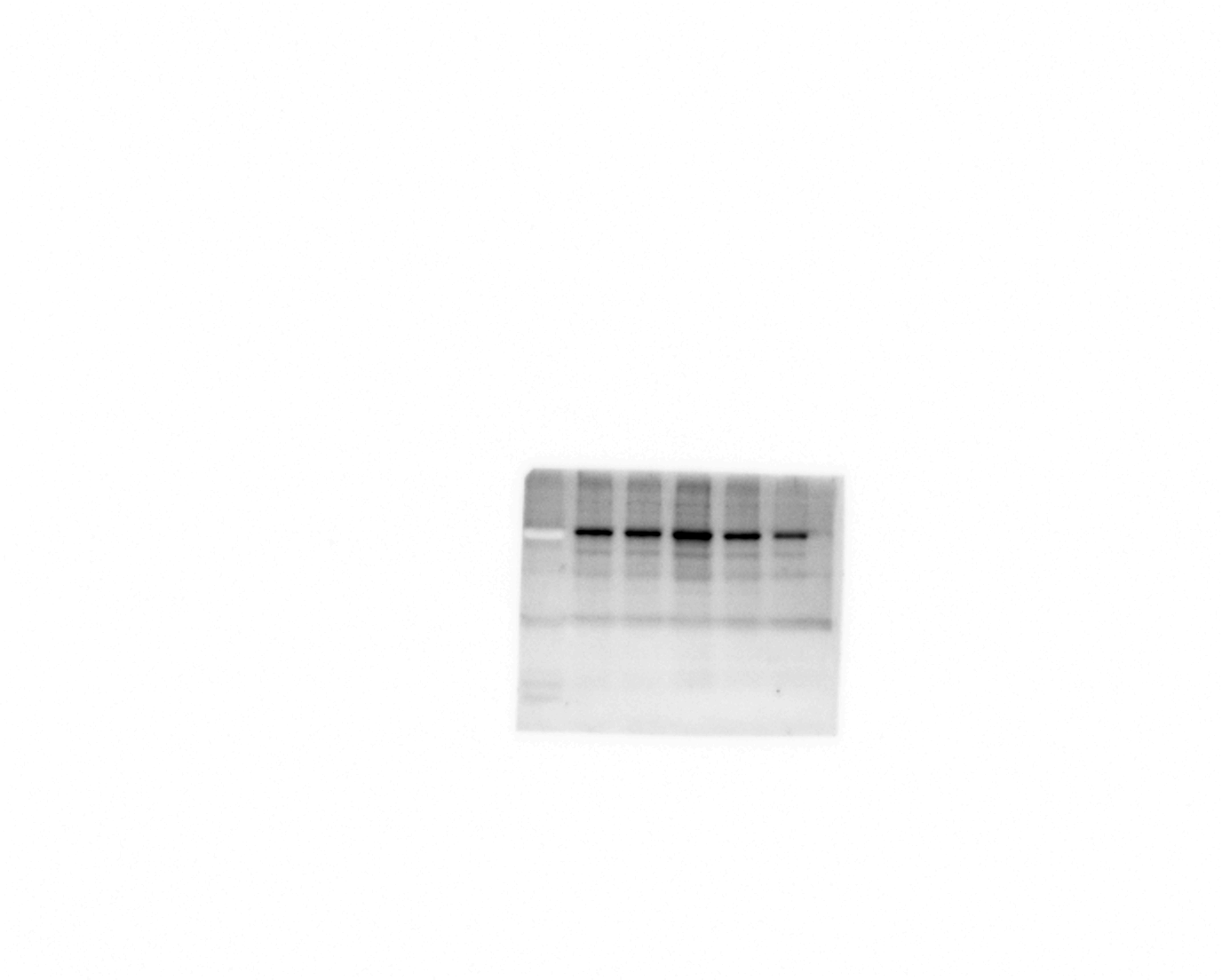

Supplement: Supplementary file 1 [file biomolecules-16-00868-s001.zip › FigureS1 the full, uncropped western blot images/The vitro primary SMCs/COX1/3.Tif]

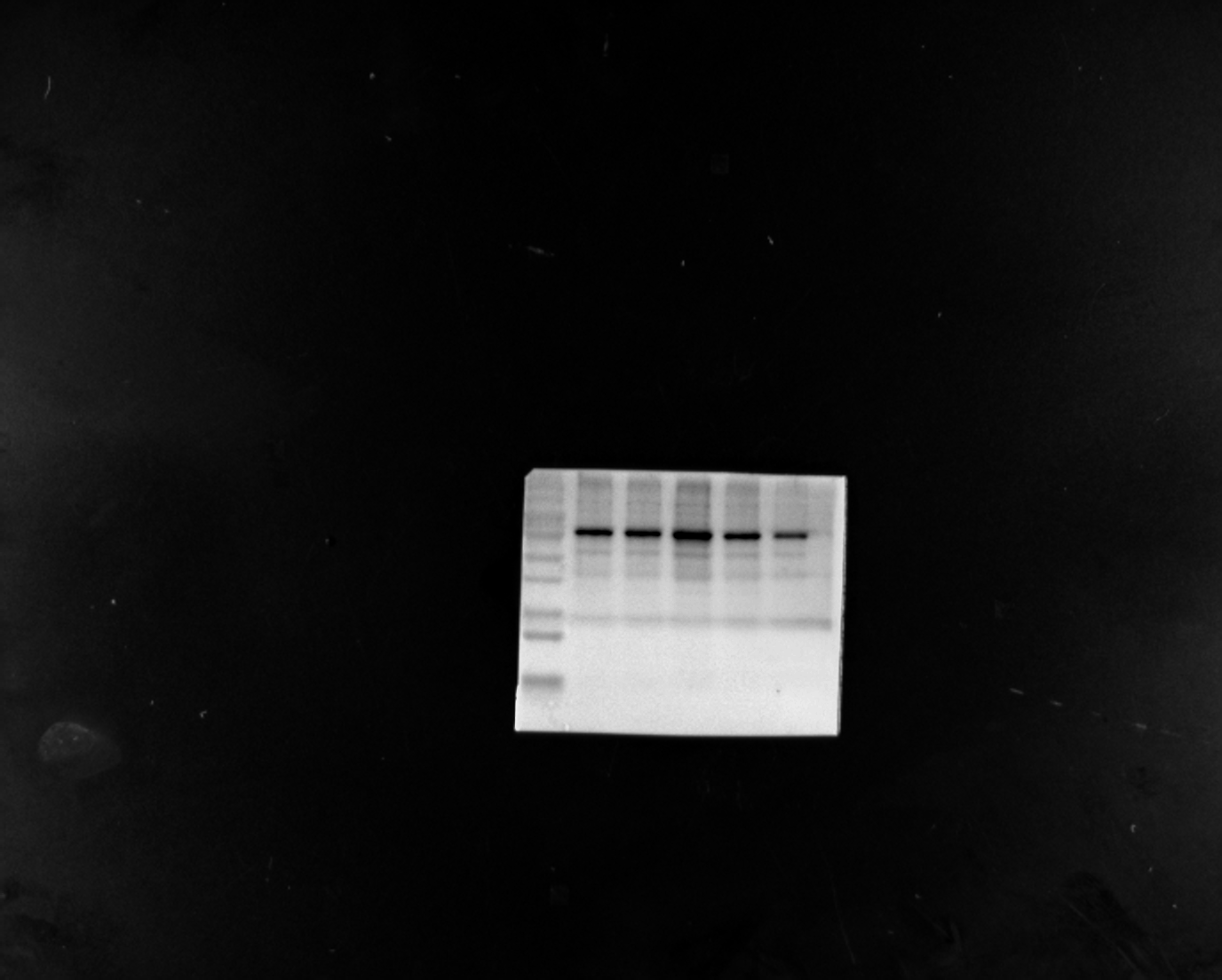

Supplement: Supplementary file 1 [file biomolecules-16-00868-s001.zip › FigureS1 the full, uncropped western blot images/The vitro primary SMCs/COX1/3副本.Tif]

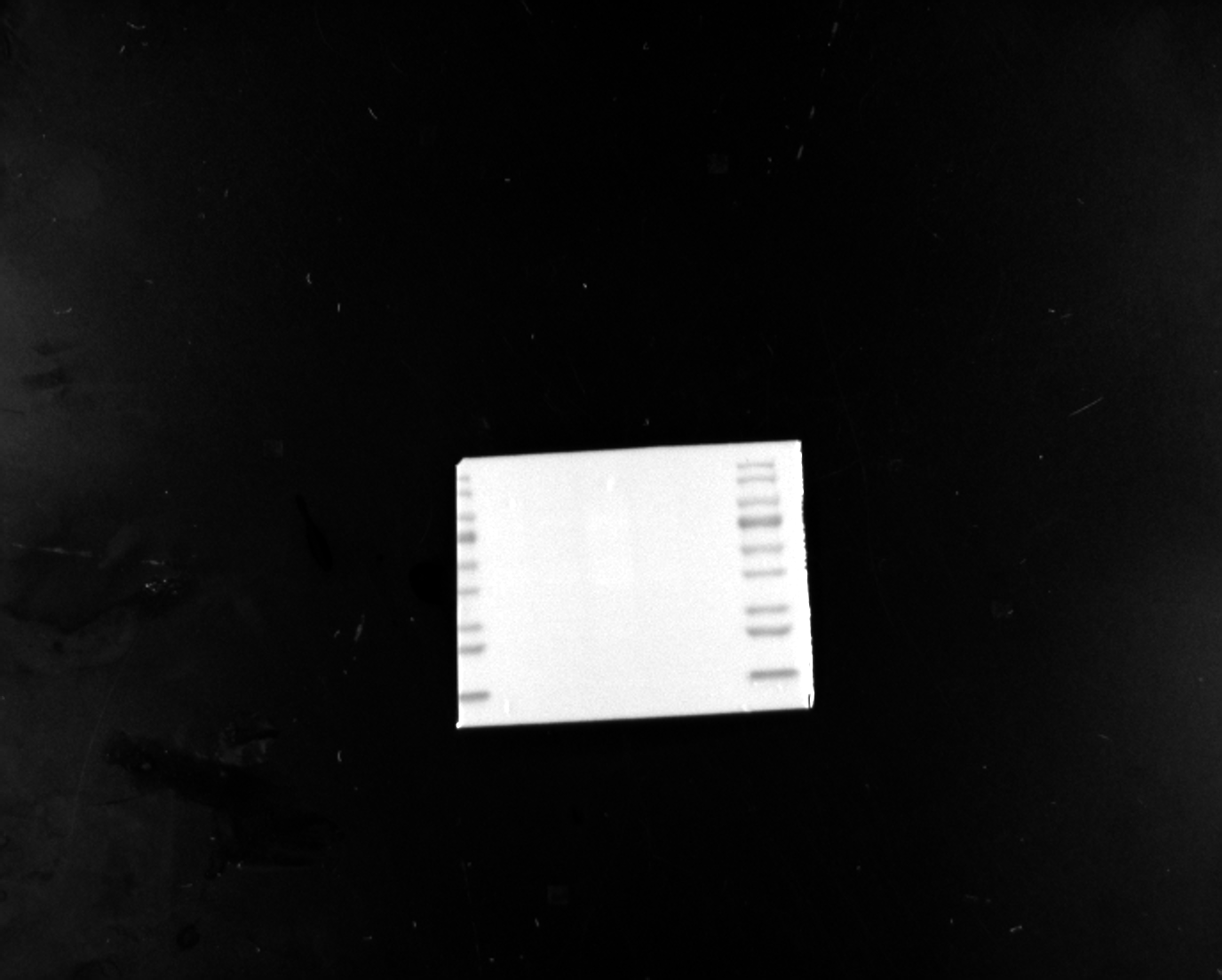

Supplement: Supplementary file 1 [file biomolecules-16-00868-s001.zip › FigureS1 the full, uncropped western blot images/The vitro primary SMCs/HSF1/1-t.Tif]

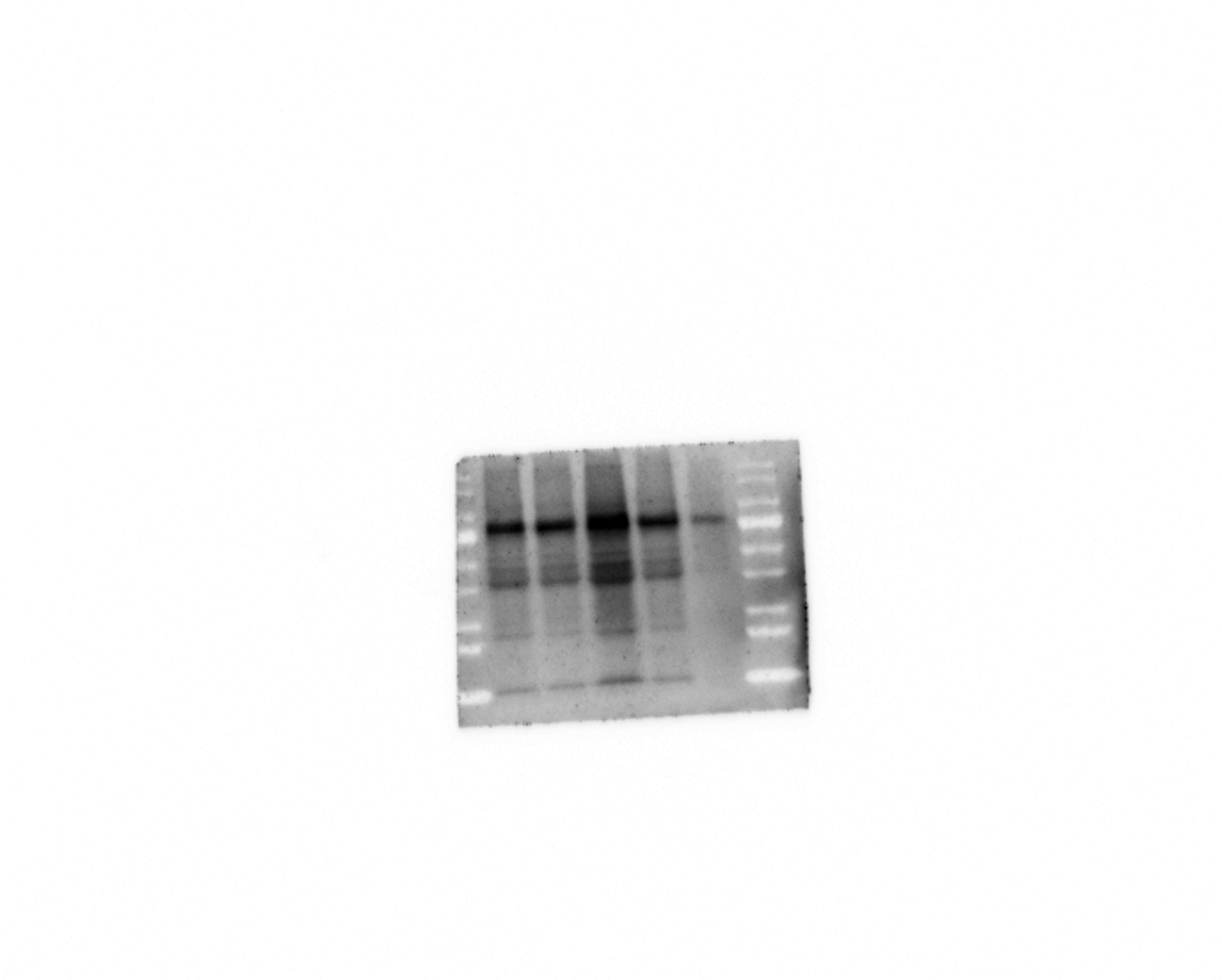

Supplement: Supplementary file 1 [file biomolecules-16-00868-s001.zip › FigureS1 the full, uncropped western blot images/The vitro primary SMCs/HSF1/1.Tif]

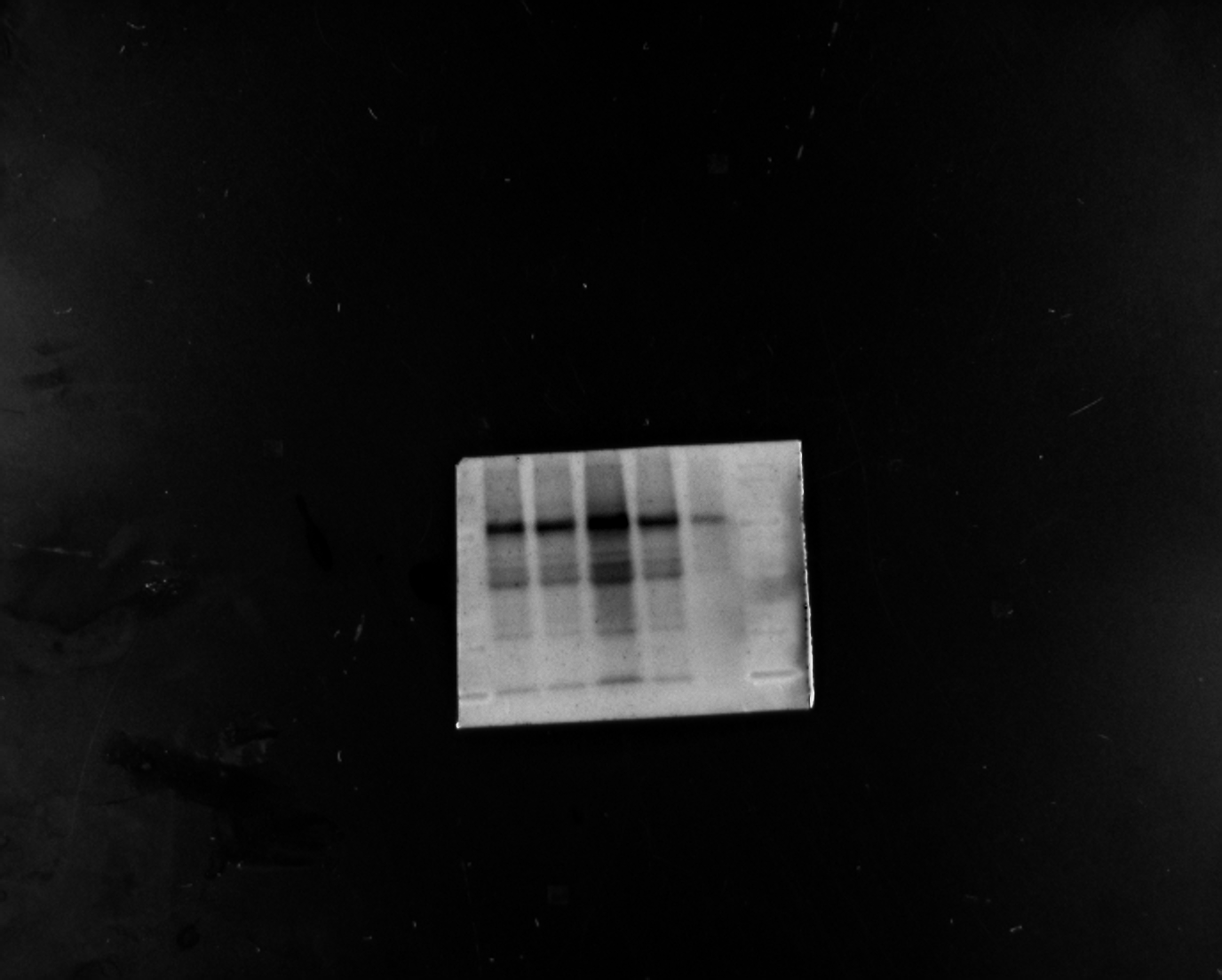

Supplement: Supplementary file 1 [file biomolecules-16-00868-s001.zip › FigureS1 the full, uncropped western blot images/The vitro primary SMCs/HSF1/1副本.Tif]

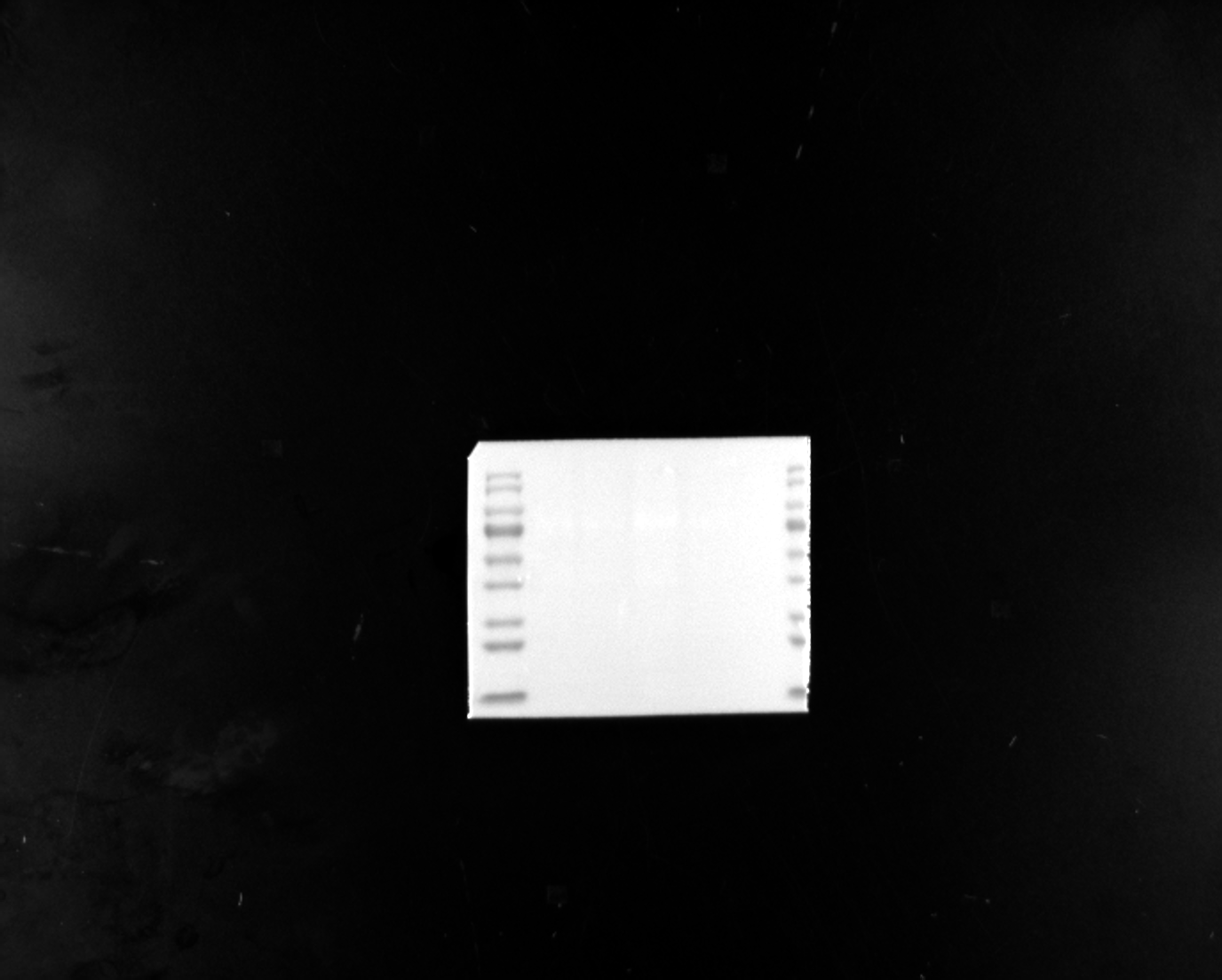

Supplement: Supplementary file 1 [file biomolecules-16-00868-s001.zip › FigureS1 the full, uncropped western blot images/The vitro primary SMCs/HSF1/2-t.Tif]

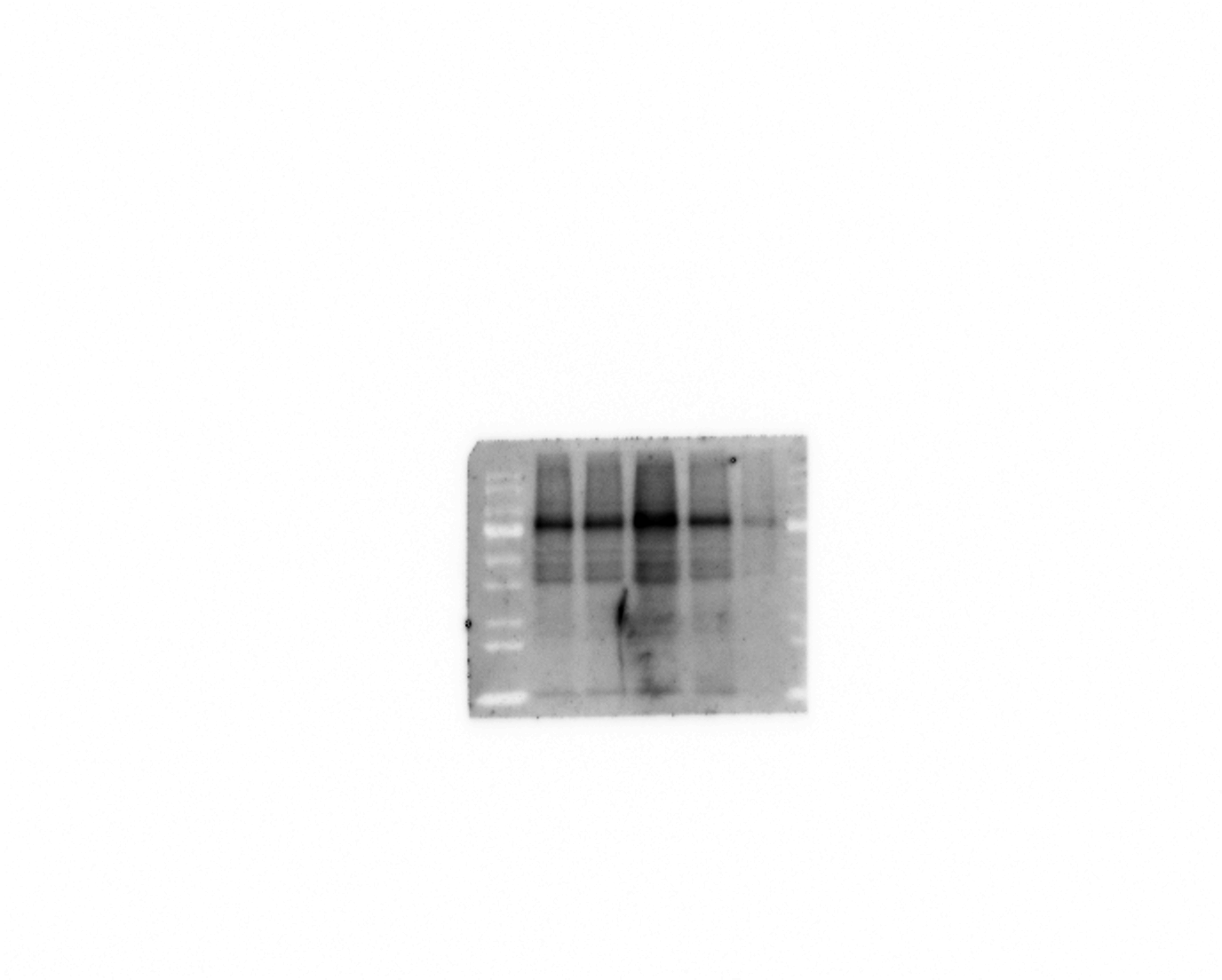

Supplement: Supplementary file 1 [file biomolecules-16-00868-s001.zip › FigureS1 the full, uncropped western blot images/The vitro primary SMCs/HSF1/2.Tif]

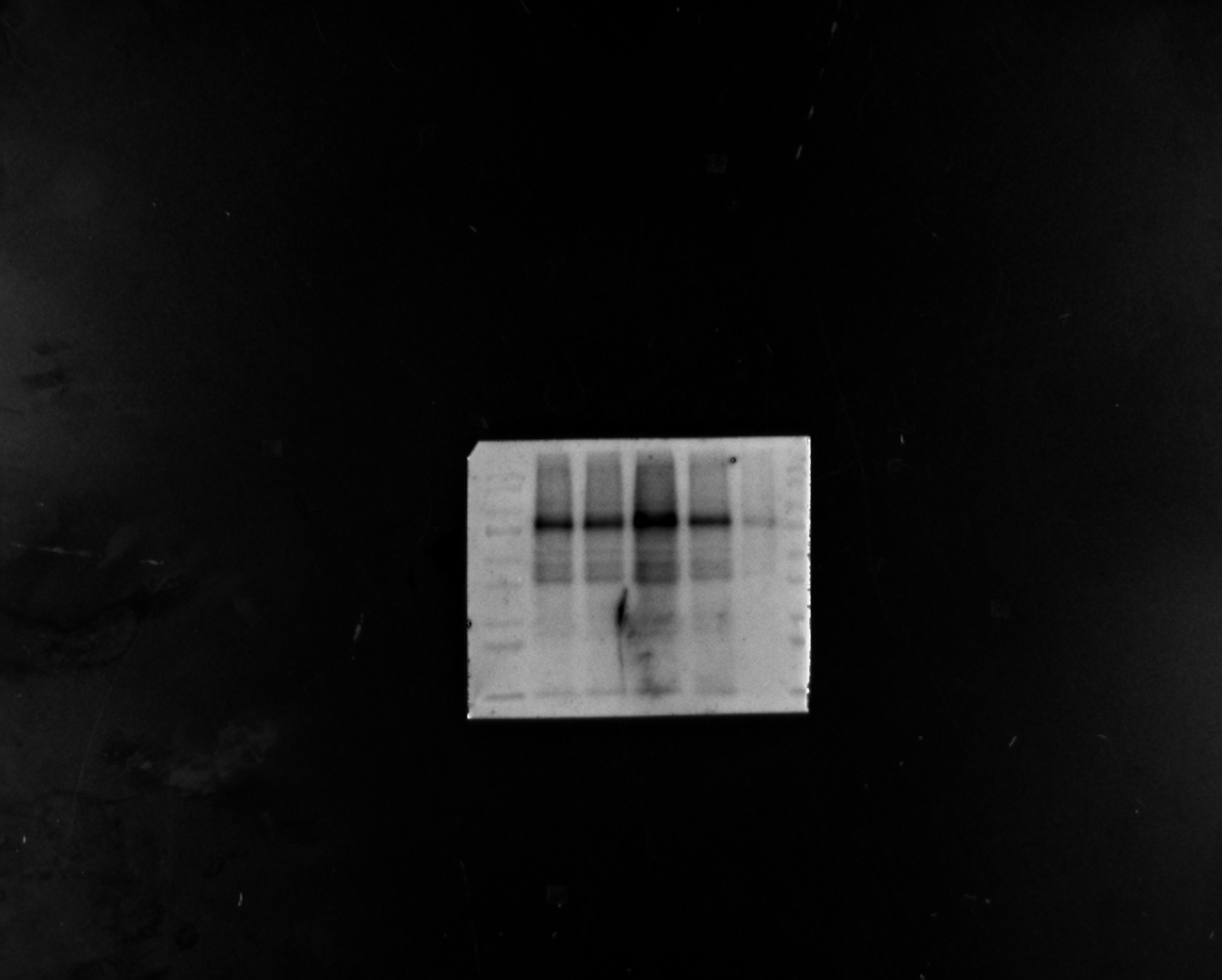

Supplement: Supplementary file 1 [file biomolecules-16-00868-s001.zip › FigureS1 the full, uncropped western blot images/The vitro primary SMCs/HSF1/2副本.Tif]

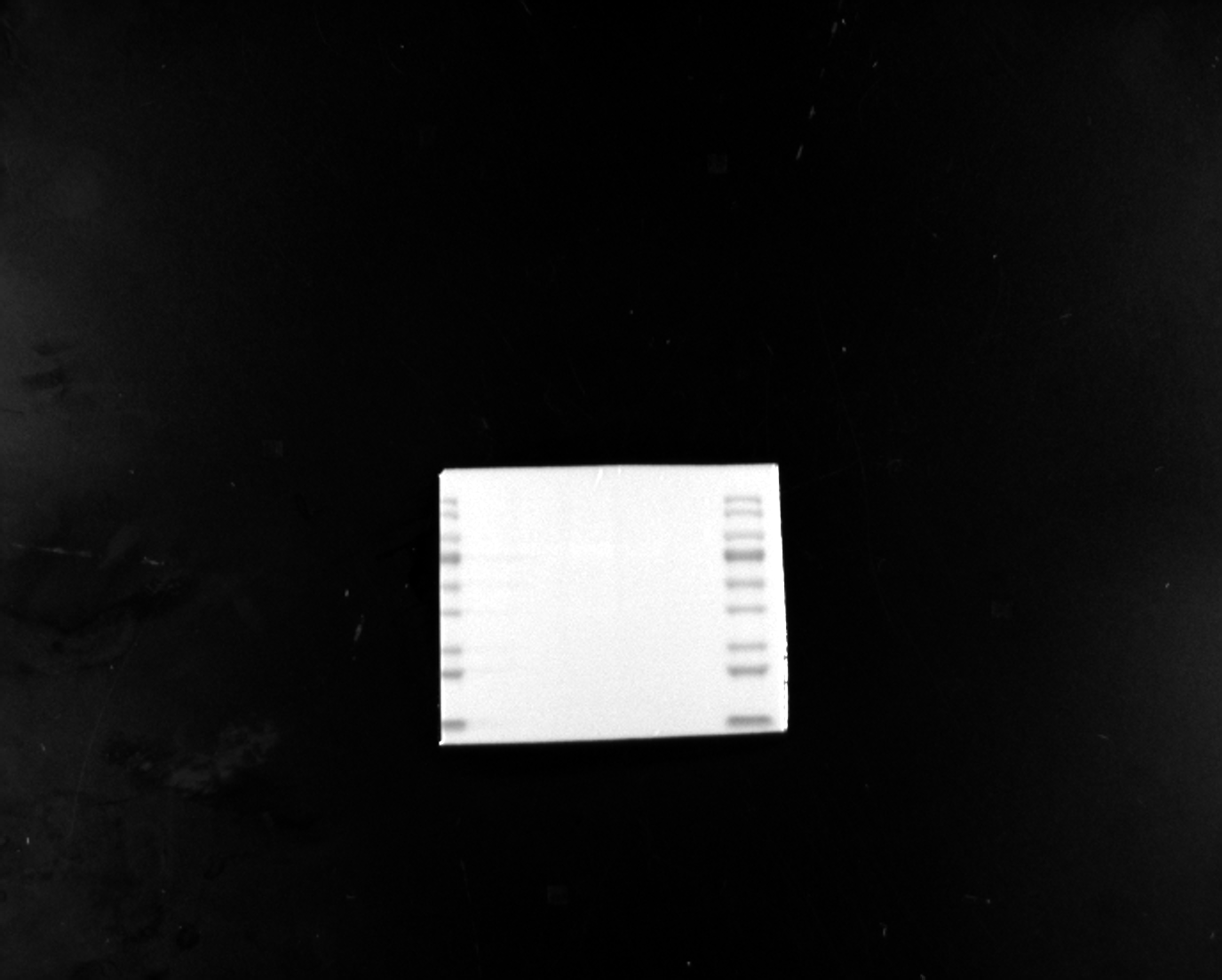

Supplement: Supplementary file 1 [file biomolecules-16-00868-s001.zip › FigureS1 the full, uncropped western blot images/The vitro primary SMCs/HSF1/3-t.Tif]

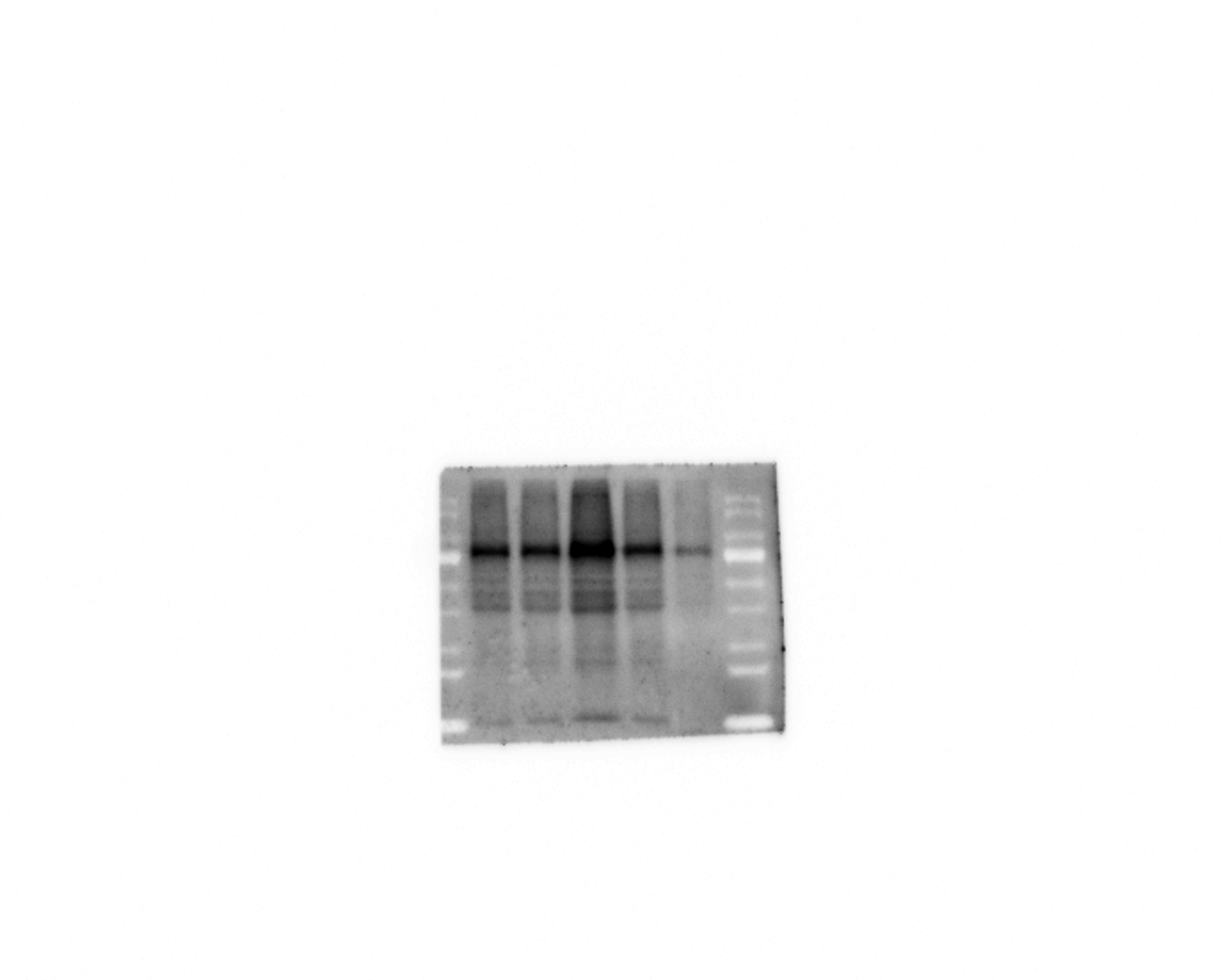

Supplement: Supplementary file 1 [file biomolecules-16-00868-s001.zip › FigureS1 the full, uncropped western blot images/The vitro primary SMCs/HSF1/3.Tif]

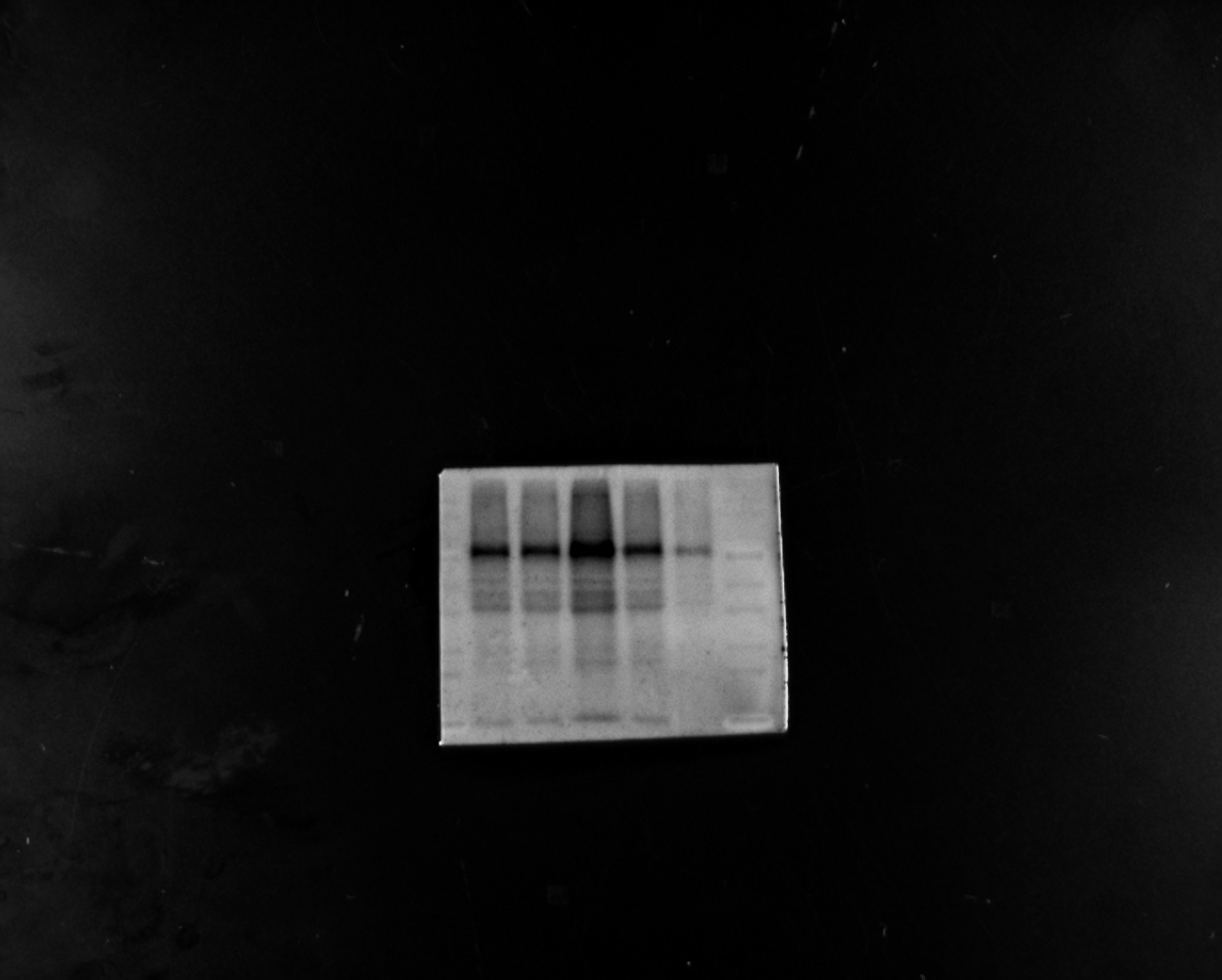

Supplement: Supplementary file 1 [file biomolecules-16-00868-s001.zip › FigureS1 the full, uncropped western blot images/The vitro primary SMCs/HSF1/3副本.Tif]

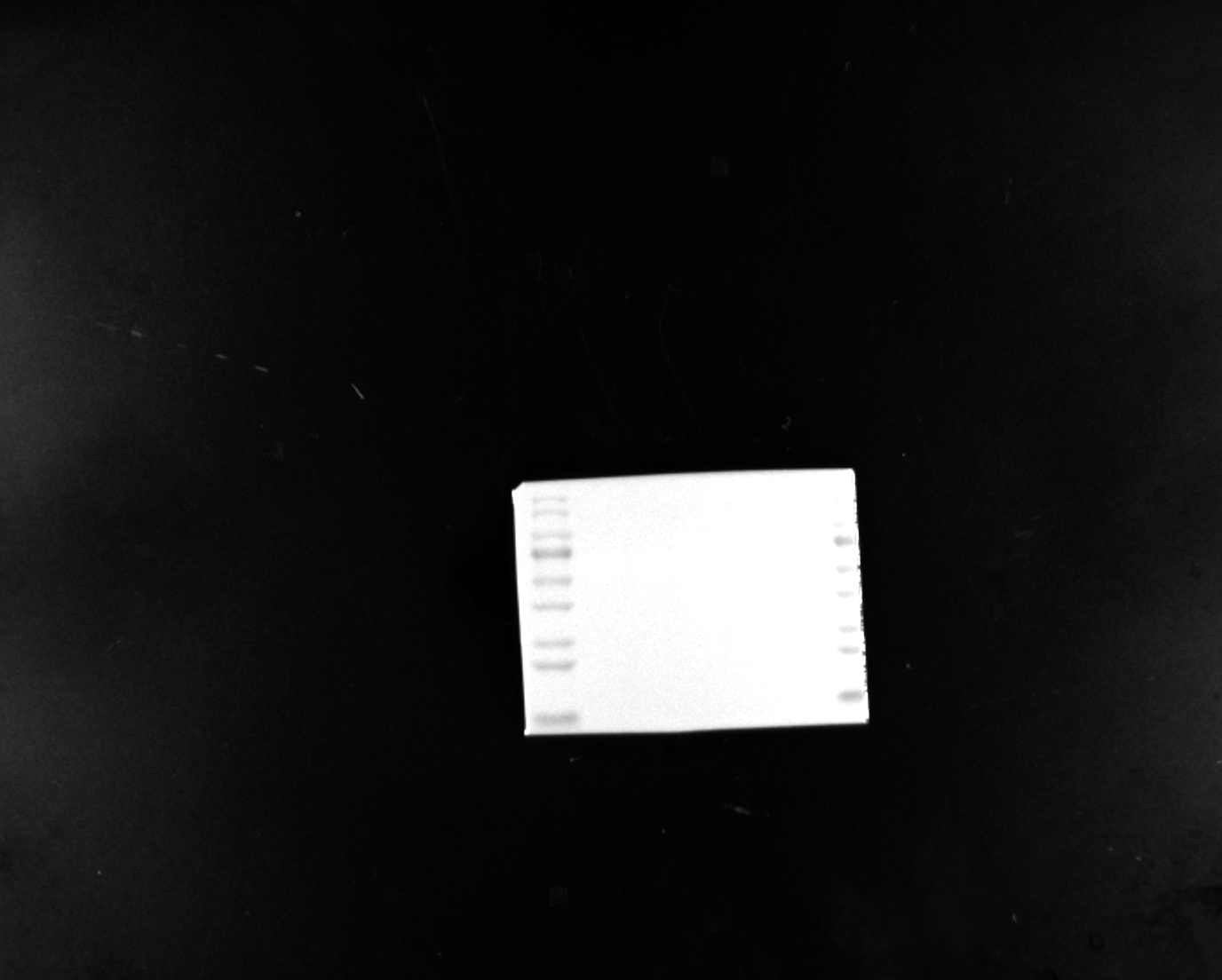

Supplement: Supplementary file 1 [file biomolecules-16-00868-s001.zip › FigureS1 the full, uncropped western blot images/The vitro primary SMCs/HSP60/1-t.Tif]

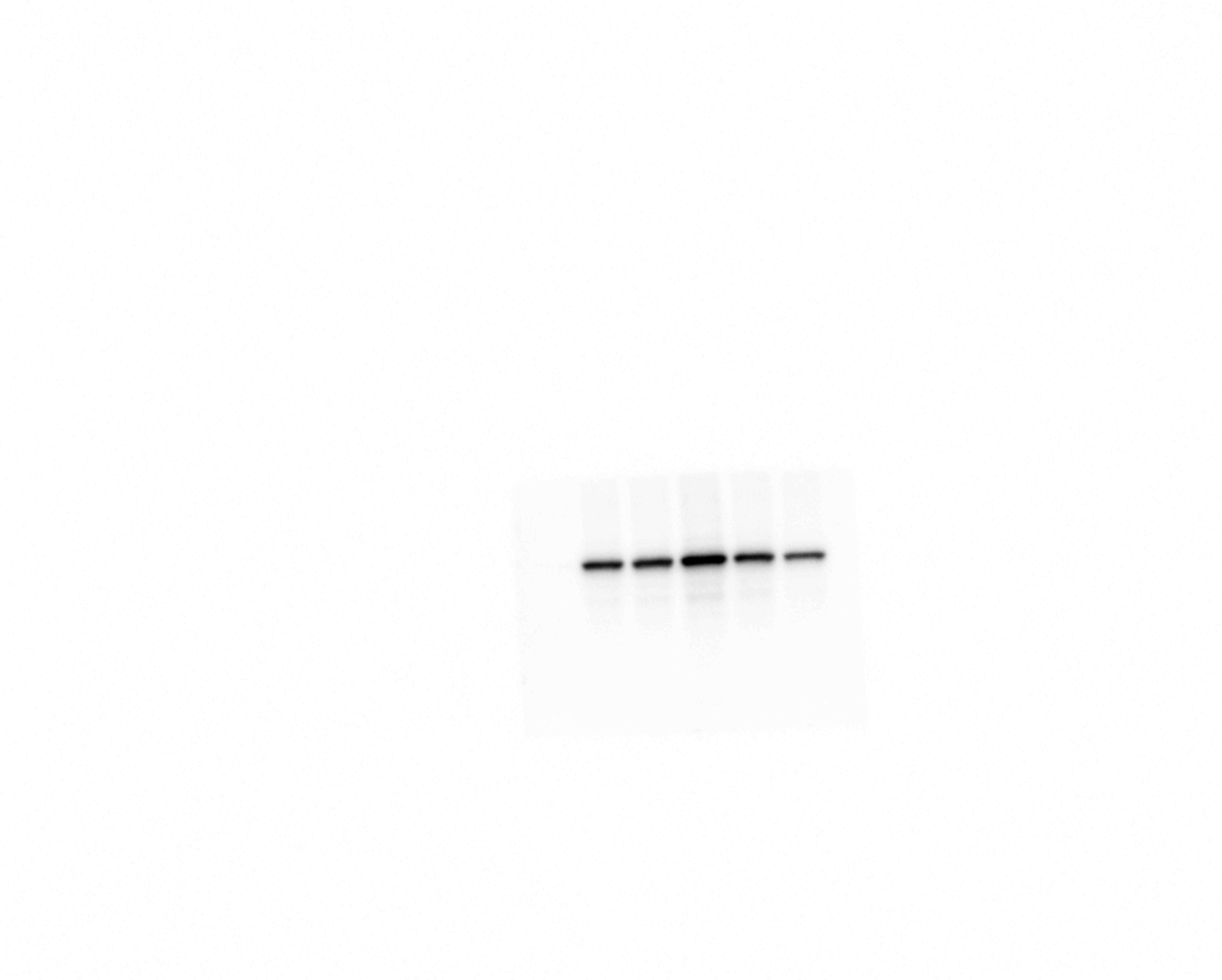

Supplement: Supplementary file 1 [file biomolecules-16-00868-s001.zip › FigureS1 the full, uncropped western blot images/The vitro primary SMCs/HSP60/1.Tif]

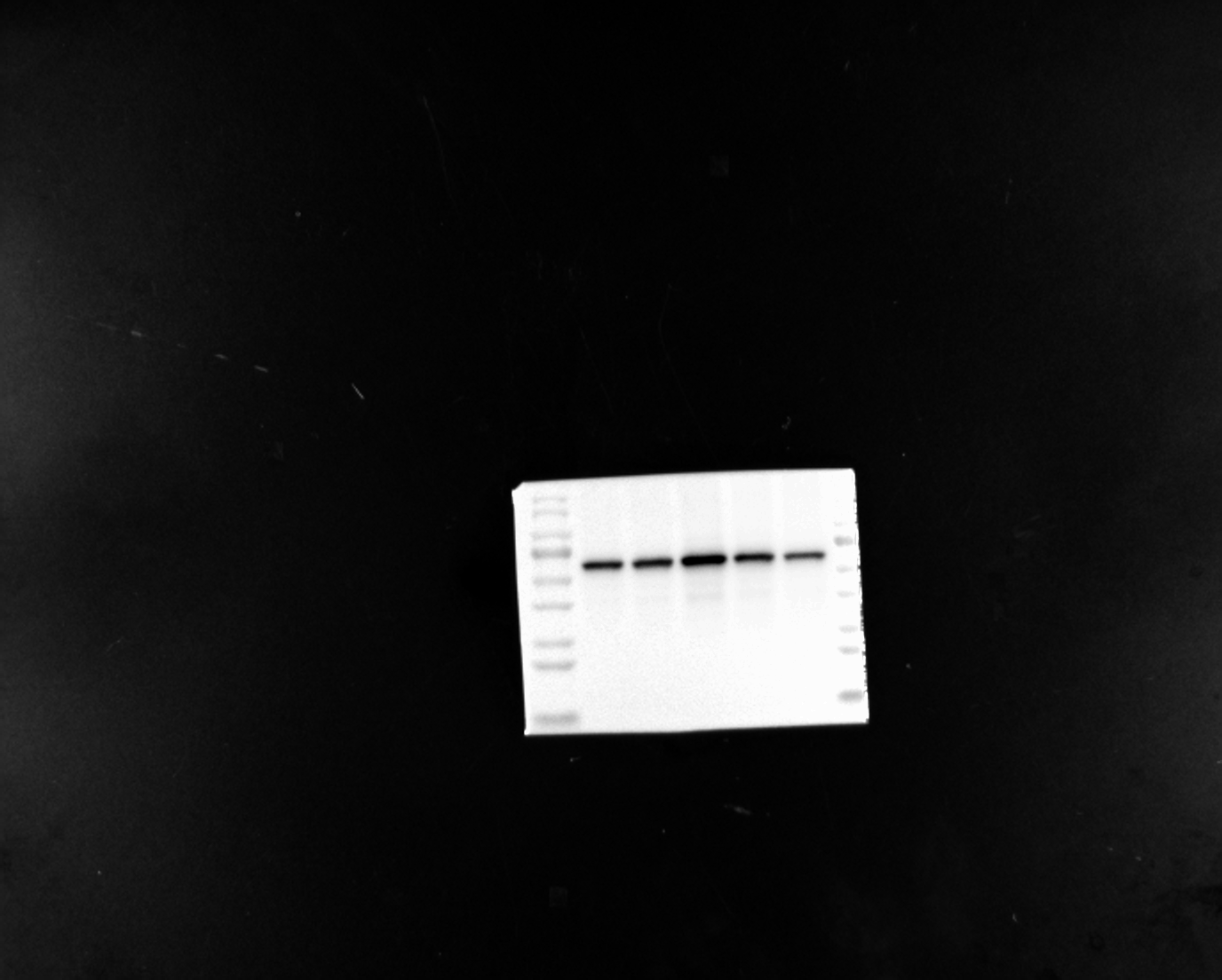

Supplement: Supplementary file 1 [file biomolecules-16-00868-s001.zip › FigureS1 the full, uncropped western blot images/The vitro primary SMCs/HSP60/1副本.Tif]

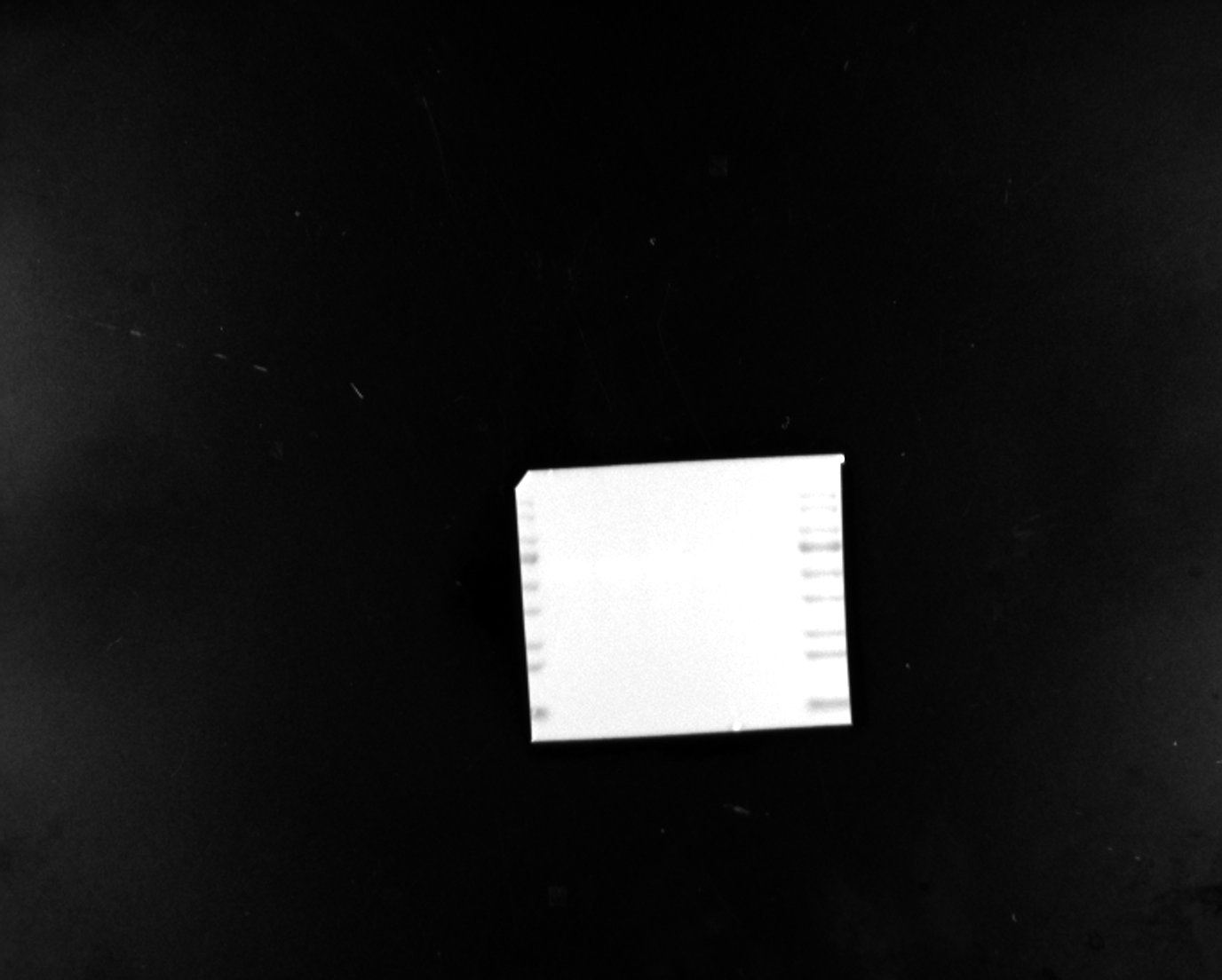

Supplement: Supplementary file 1 [file biomolecules-16-00868-s001.zip › FigureS1 the full, uncropped western blot images/The vitro primary SMCs/HSP60/2-t.Tif]

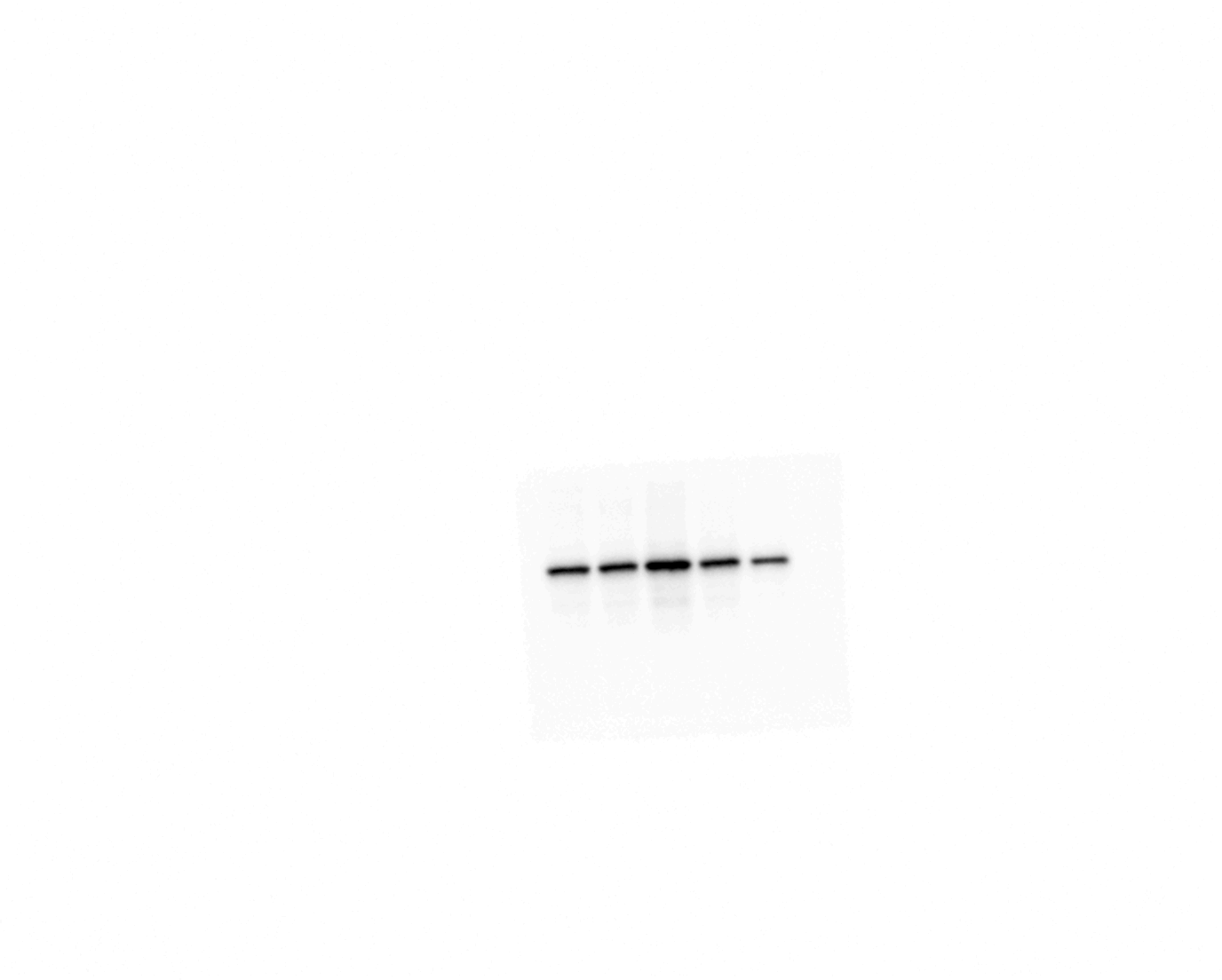

Supplement: Supplementary file 1 [file biomolecules-16-00868-s001.zip › FigureS1 the full, uncropped western blot images/The vitro primary SMCs/HSP60/2.Tif]

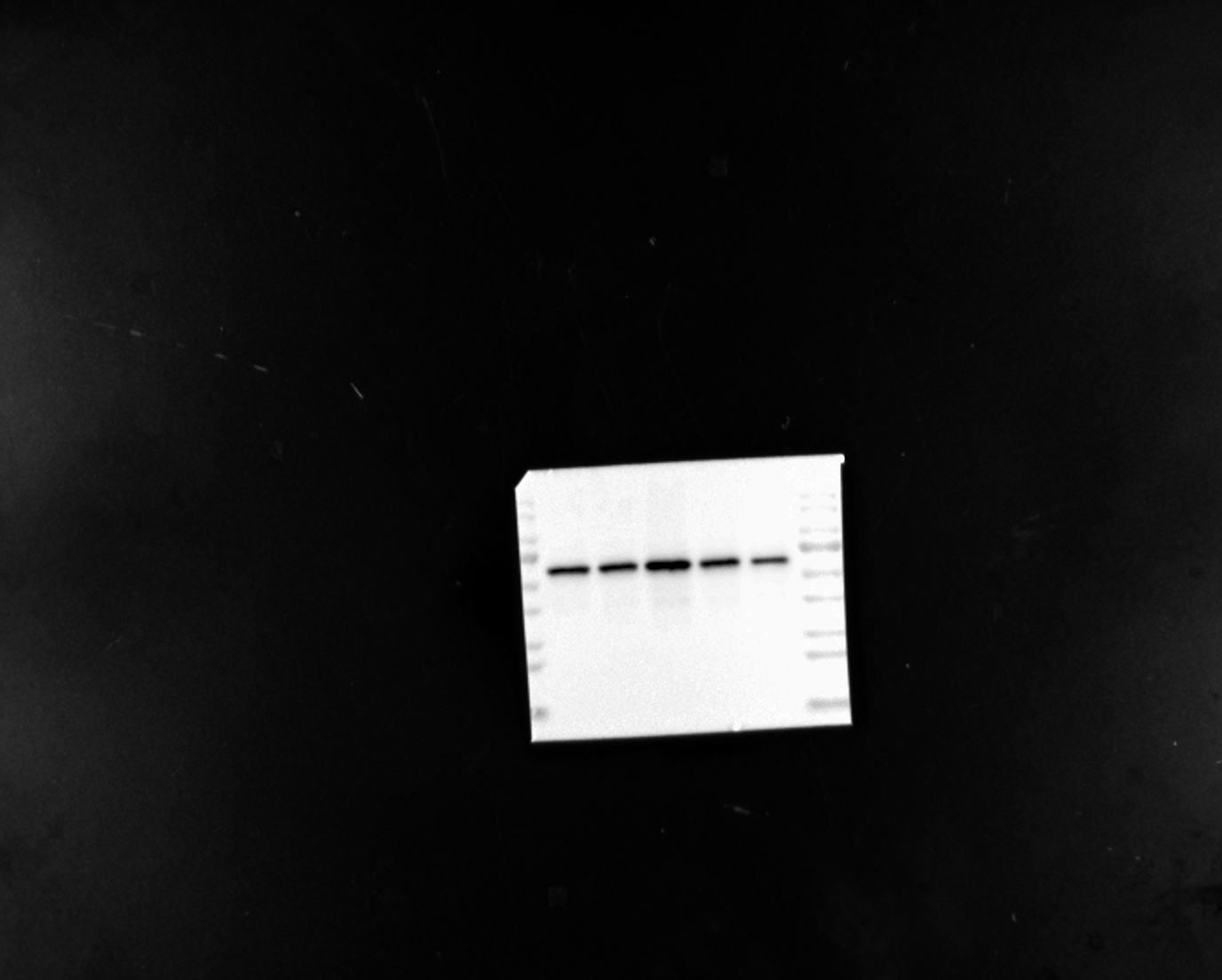

Supplement: Supplementary file 1 [file biomolecules-16-00868-s001.zip › FigureS1 the full, uncropped western blot images/The vitro primary SMCs/HSP60/2副本.Tif]

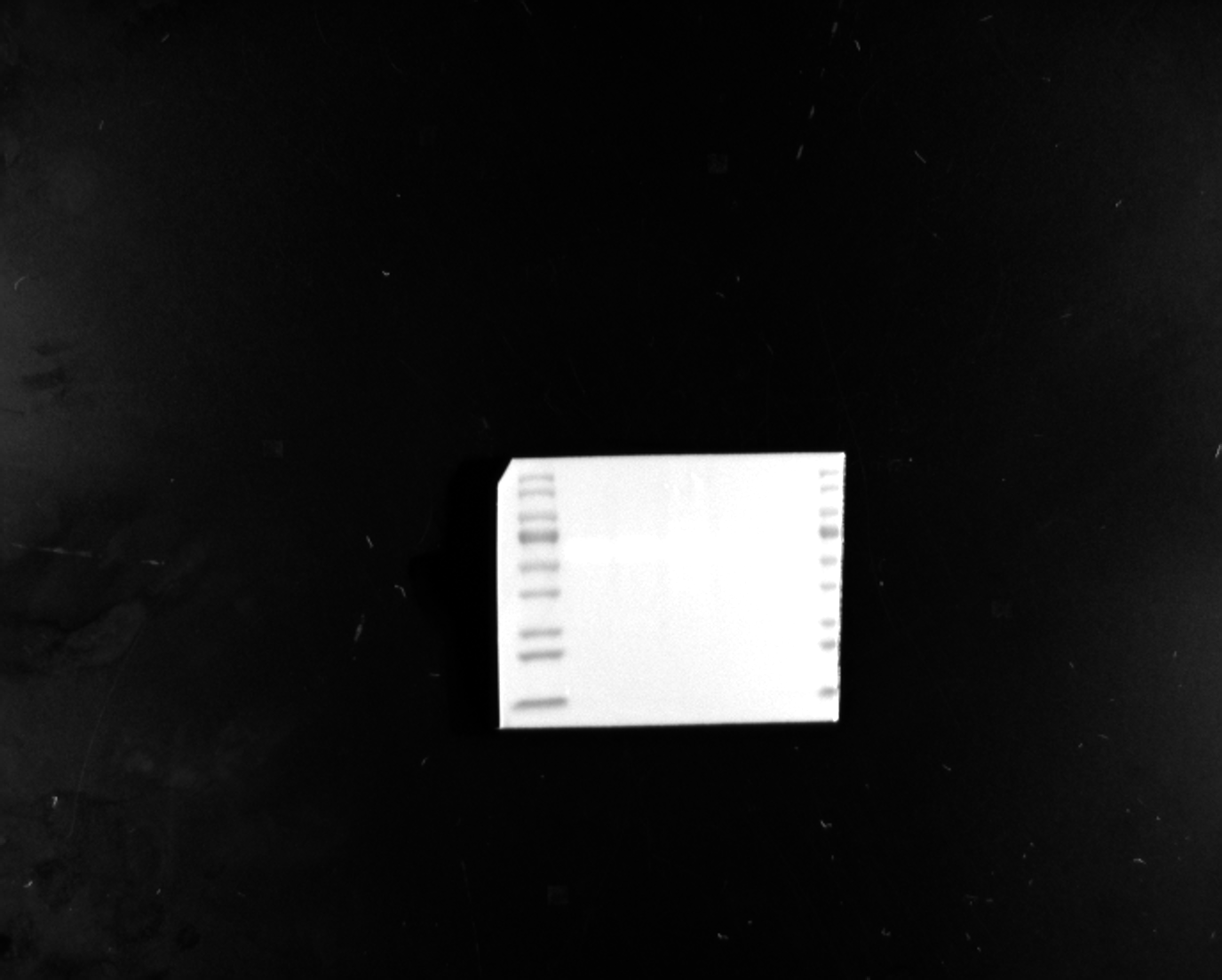

Supplement: Supplementary file 1 [file biomolecules-16-00868-s001.zip › FigureS1 the full, uncropped western blot images/The vitro primary SMCs/HSP60/3-t.Tif]

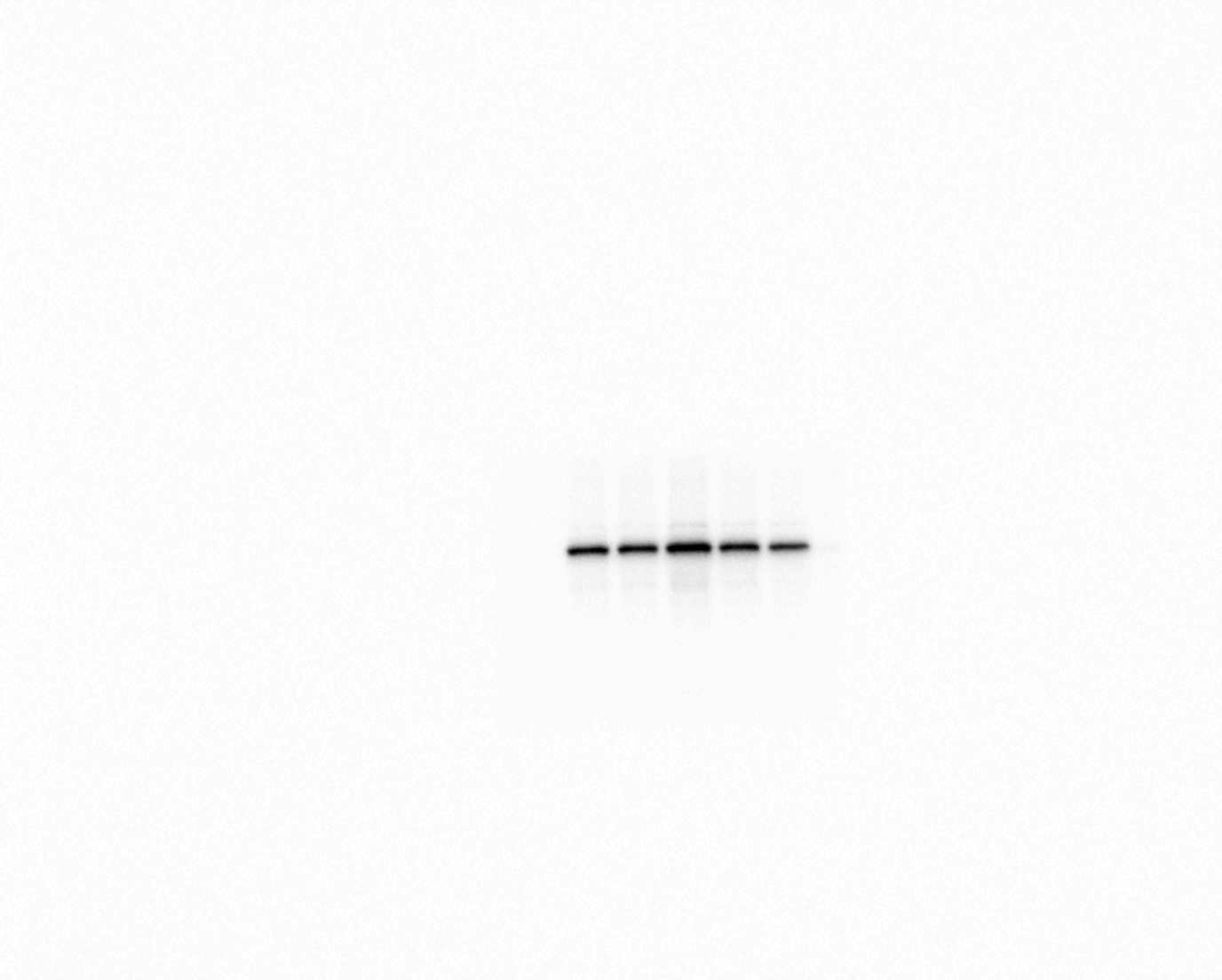

Supplement: Supplementary file 1 [file biomolecules-16-00868-s001.zip › FigureS1 the full, uncropped western blot images/The vitro primary SMCs/HSP60/3.Tif]

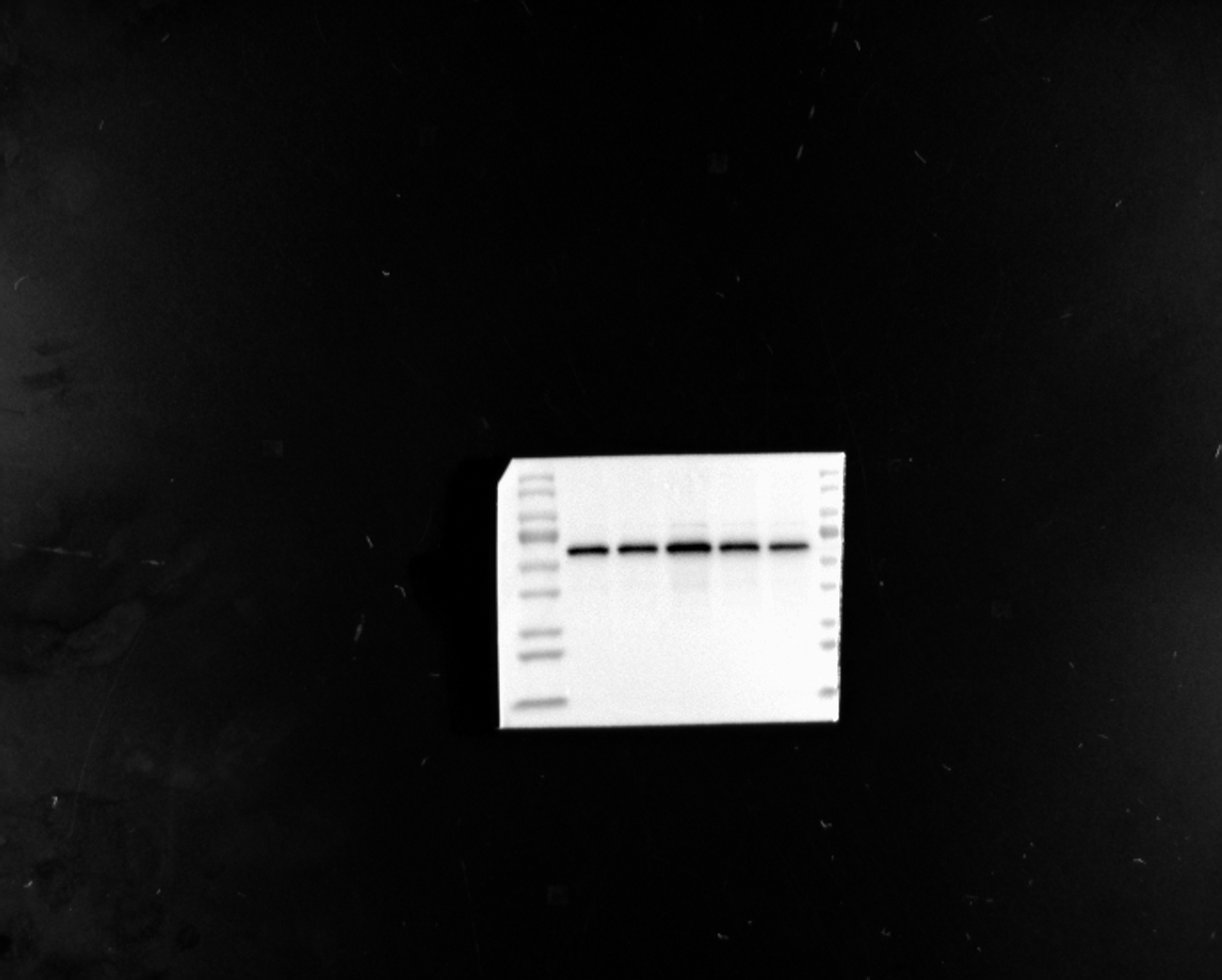

Supplement: Supplementary file 1 [file biomolecules-16-00868-s001.zip › FigureS1 the full, uncropped western blot images/The vitro primary SMCs/HSP60/3副本.Tif]

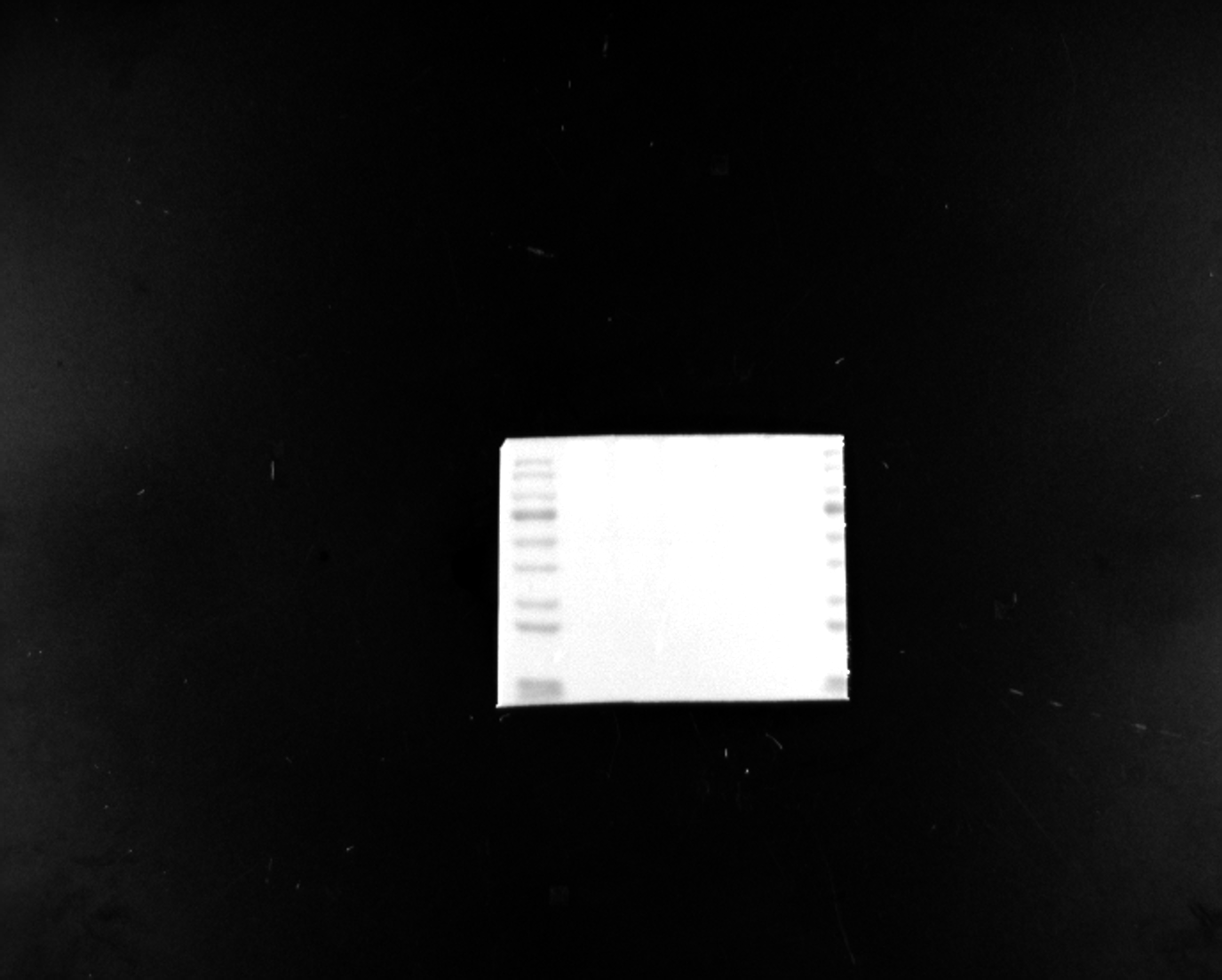

Supplement: Supplementary file 1 [file biomolecules-16-00868-s001.zip › FigureS1 the full, uncropped western blot images/The vitro primary SMCs/LONP1/1-t.Tif]

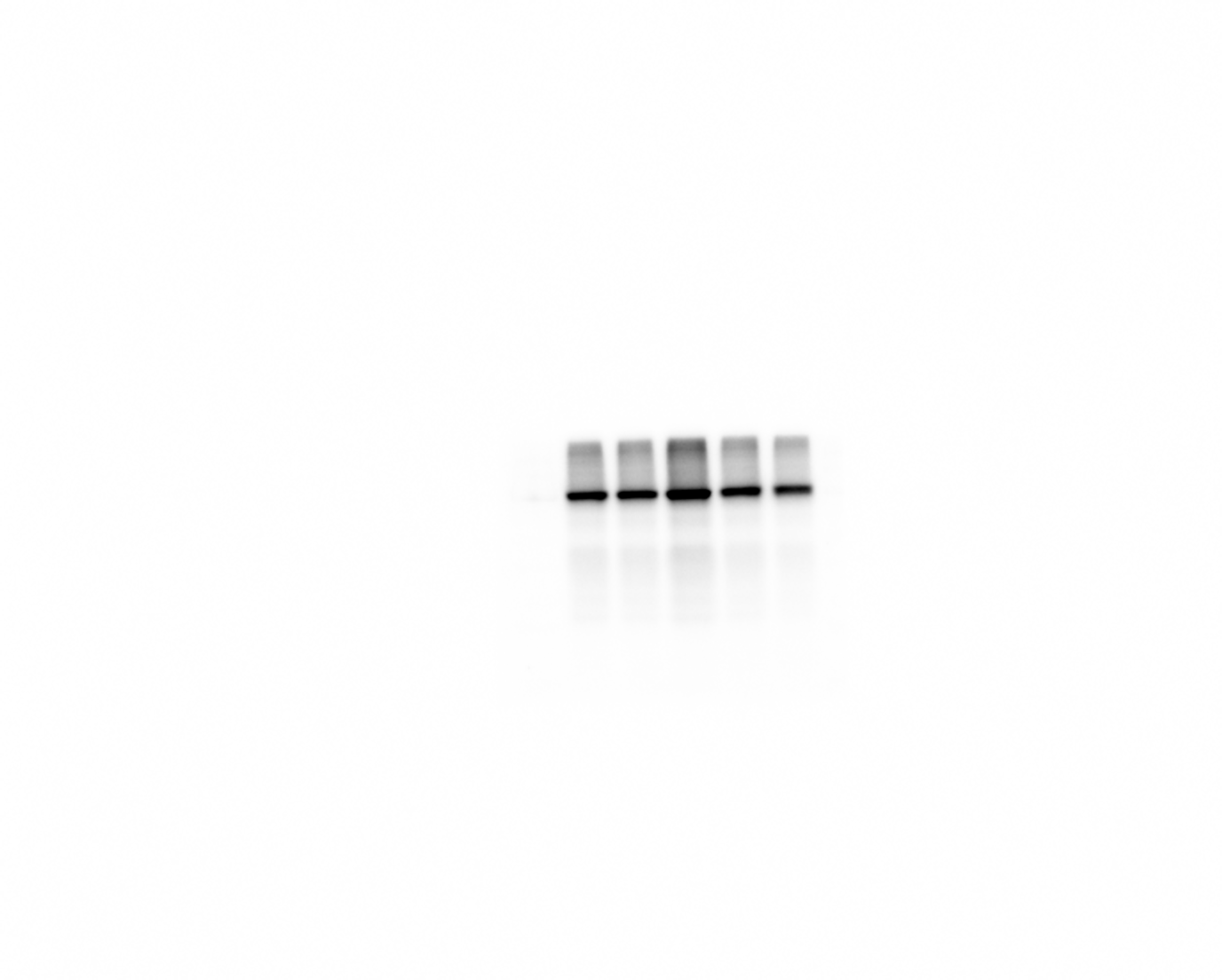

Supplement: Supplementary file 1 [file biomolecules-16-00868-s001.zip › FigureS1 the full, uncropped western blot images/The vitro primary SMCs/LONP1/1.Tif]

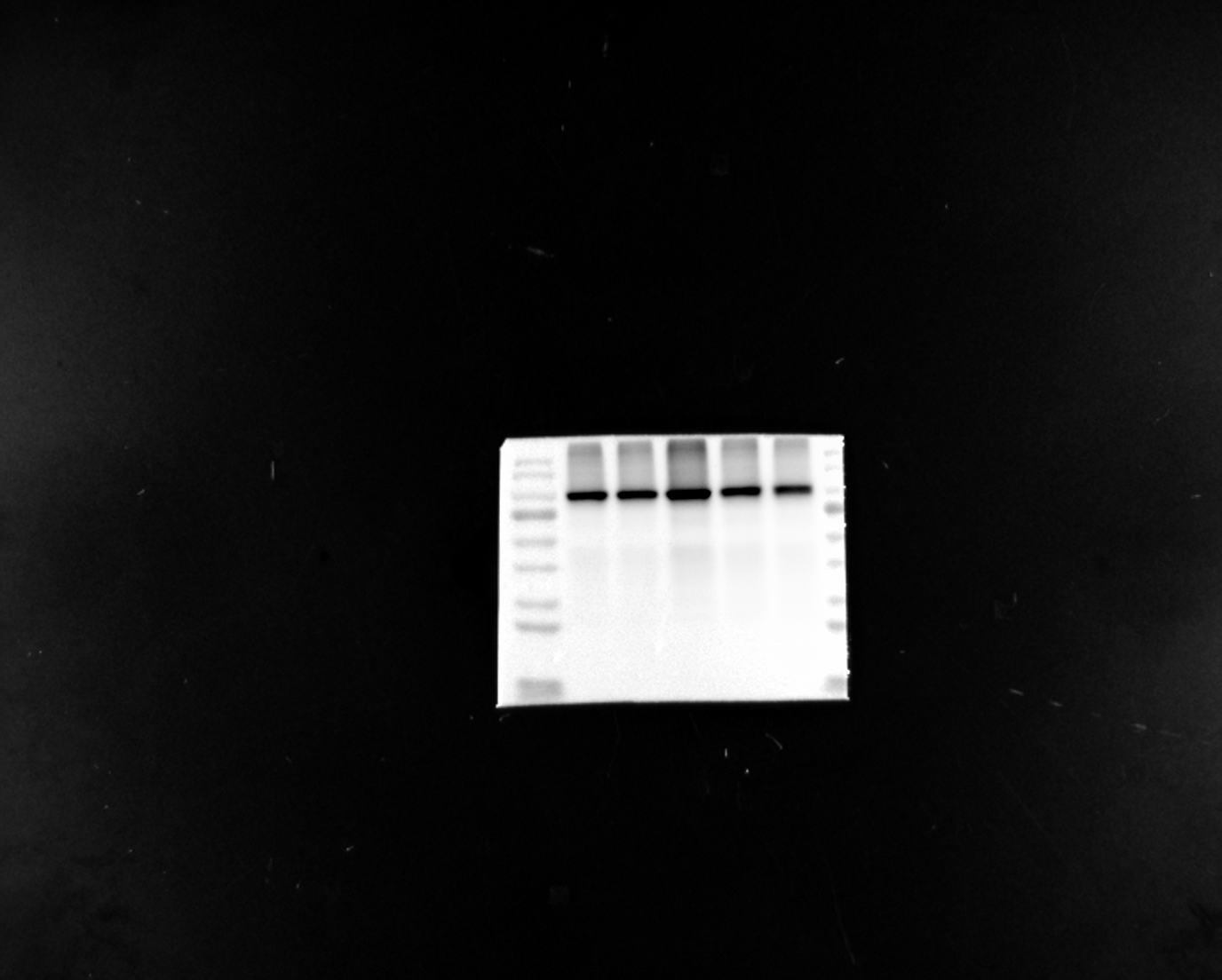

Supplement: Supplementary file 1 [file biomolecules-16-00868-s001.zip › FigureS1 the full, uncropped western blot images/The vitro primary SMCs/LONP1/1副本.Tif]

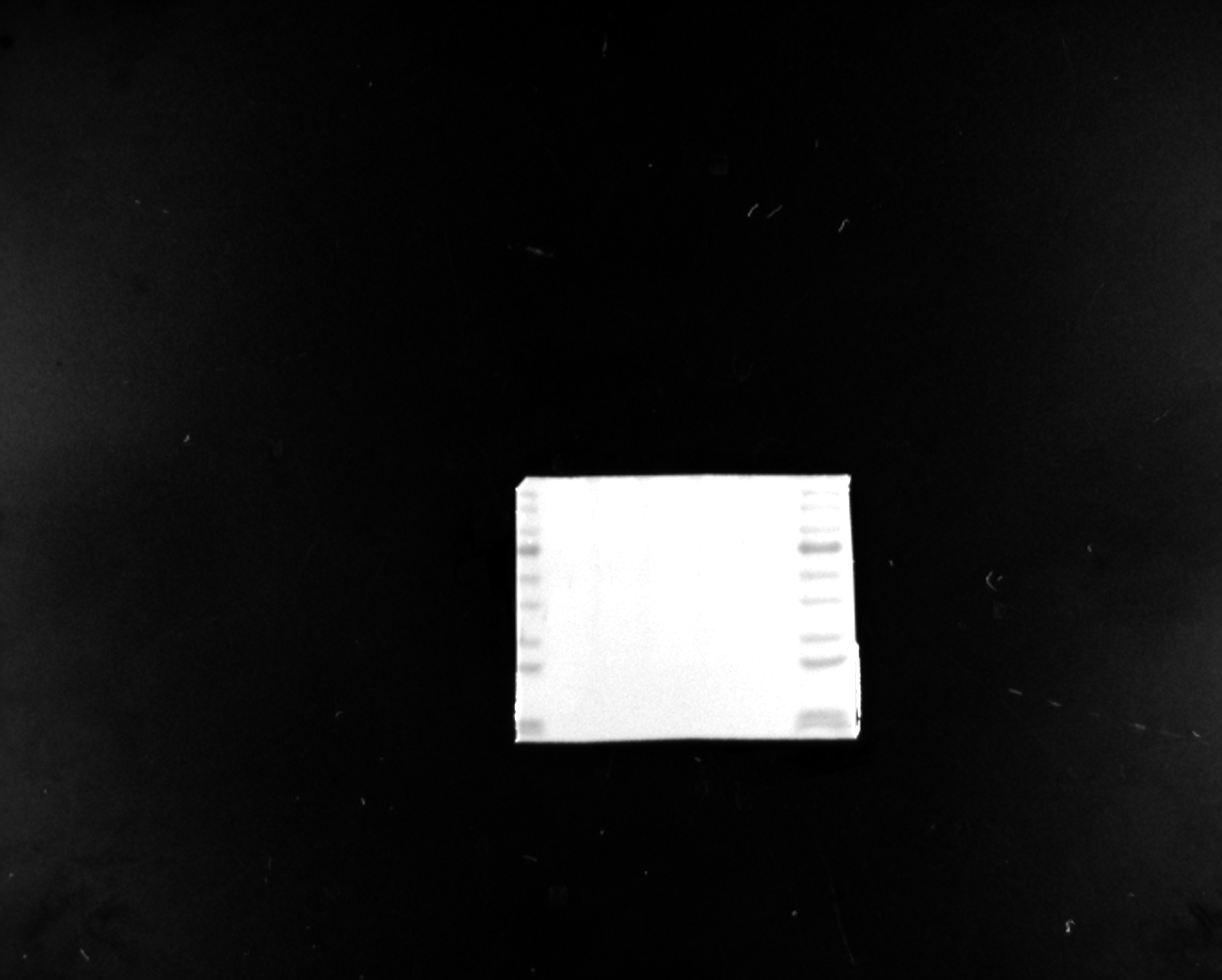

Supplement: Supplementary file 1 [file biomolecules-16-00868-s001.zip › FigureS1 the full, uncropped western blot images/The vitro primary SMCs/LONP1/2-t.Tif]

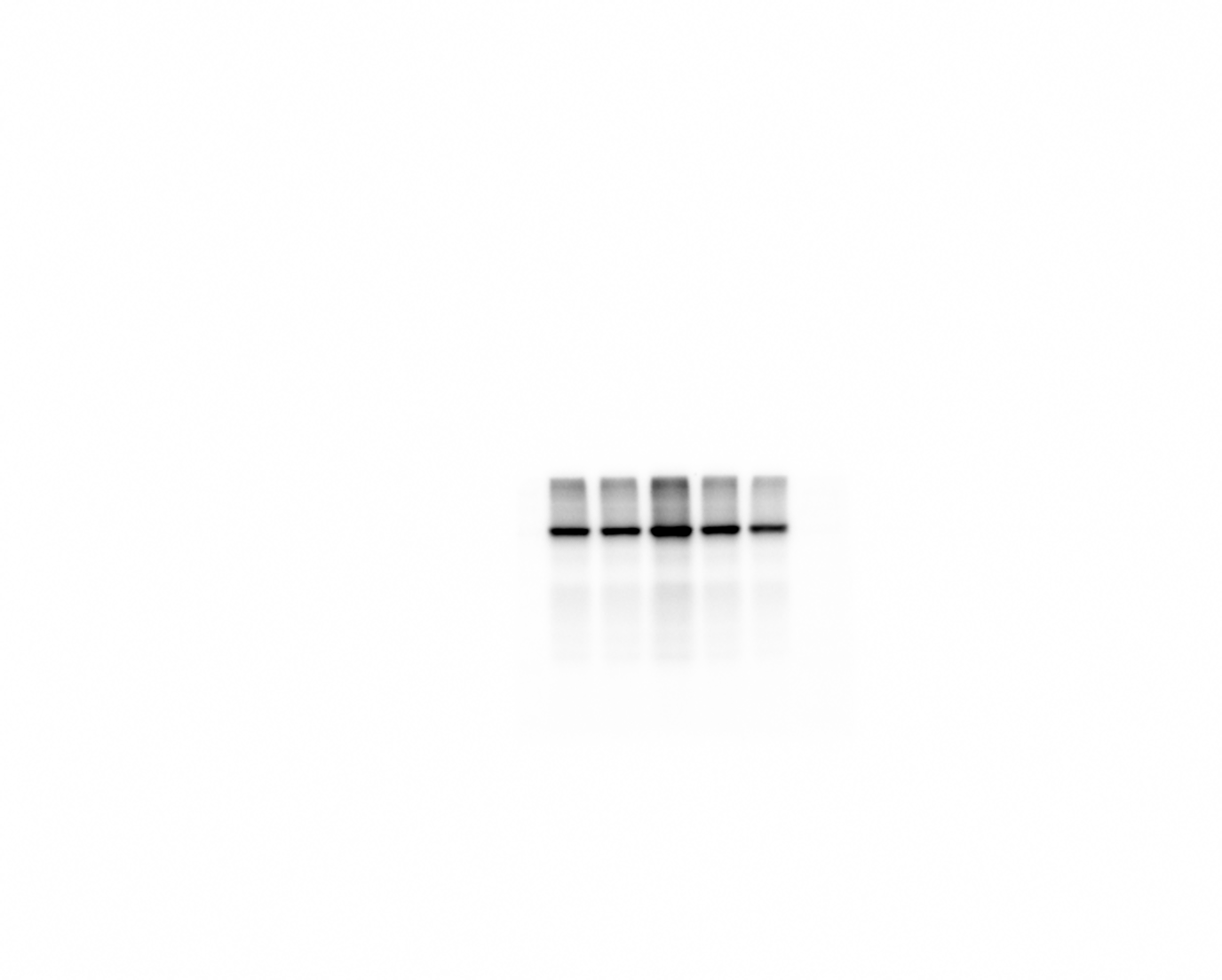

Supplement: Supplementary file 1 [file biomolecules-16-00868-s001.zip › FigureS1 the full, uncropped western blot images/The vitro primary SMCs/LONP1/2.Tif]

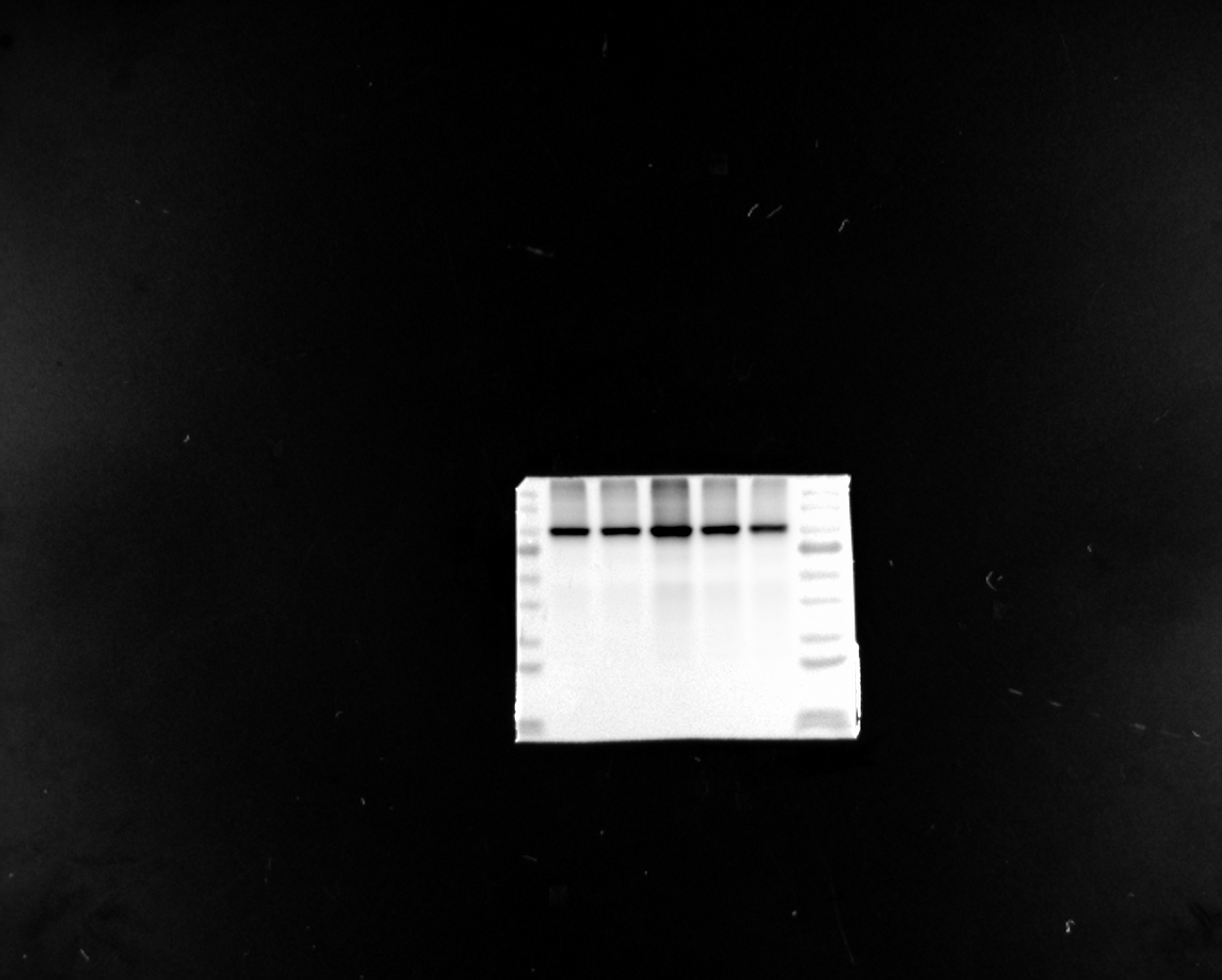

Supplement: Supplementary file 1 [file biomolecules-16-00868-s001.zip › FigureS1 the full, uncropped western blot images/The vitro primary SMCs/LONP1/2副本.Tif]

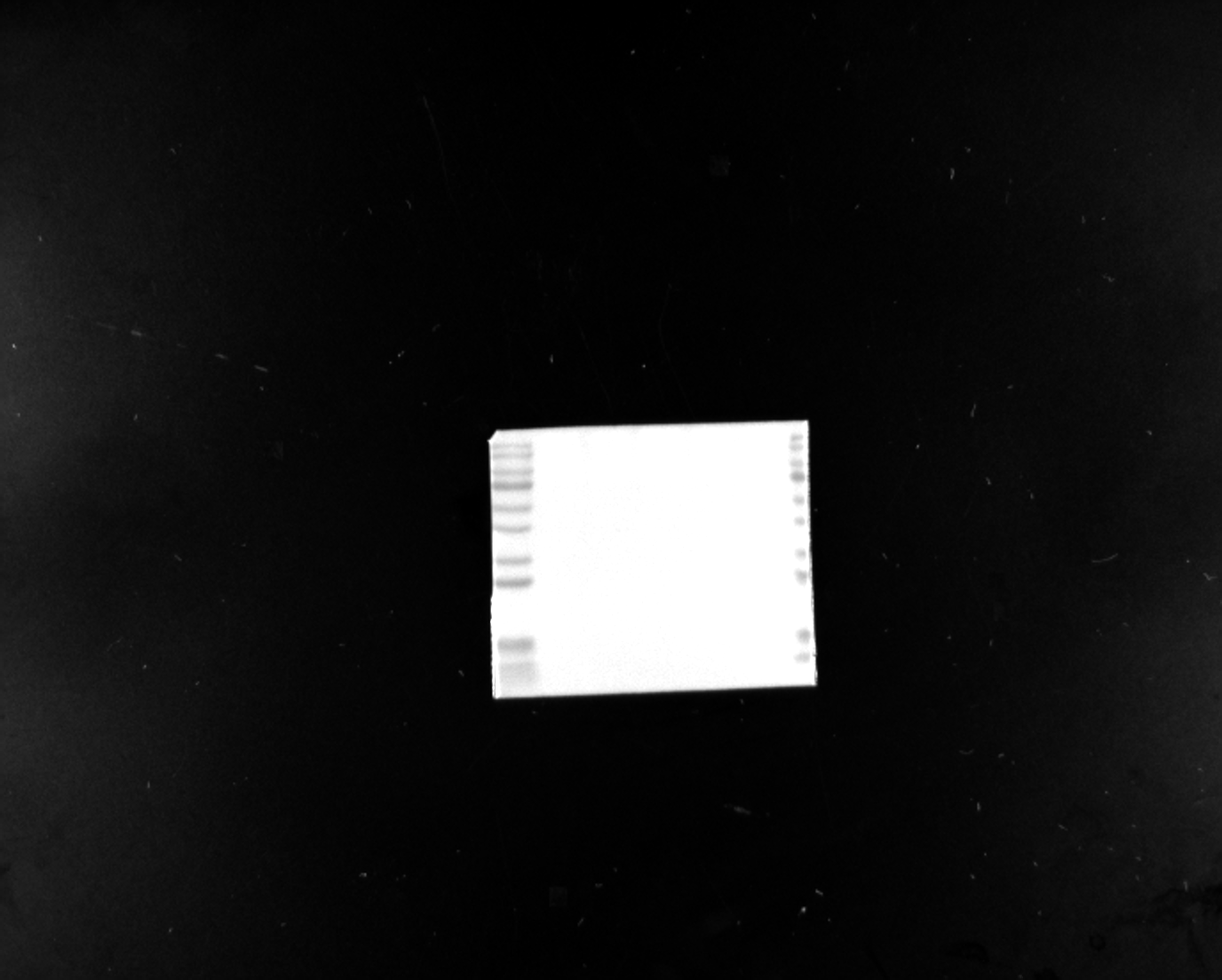

Supplement: Supplementary file 1 [file biomolecules-16-00868-s001.zip › FigureS1 the full, uncropped western blot images/The vitro primary SMCs/LONP1/3-t.Tif]

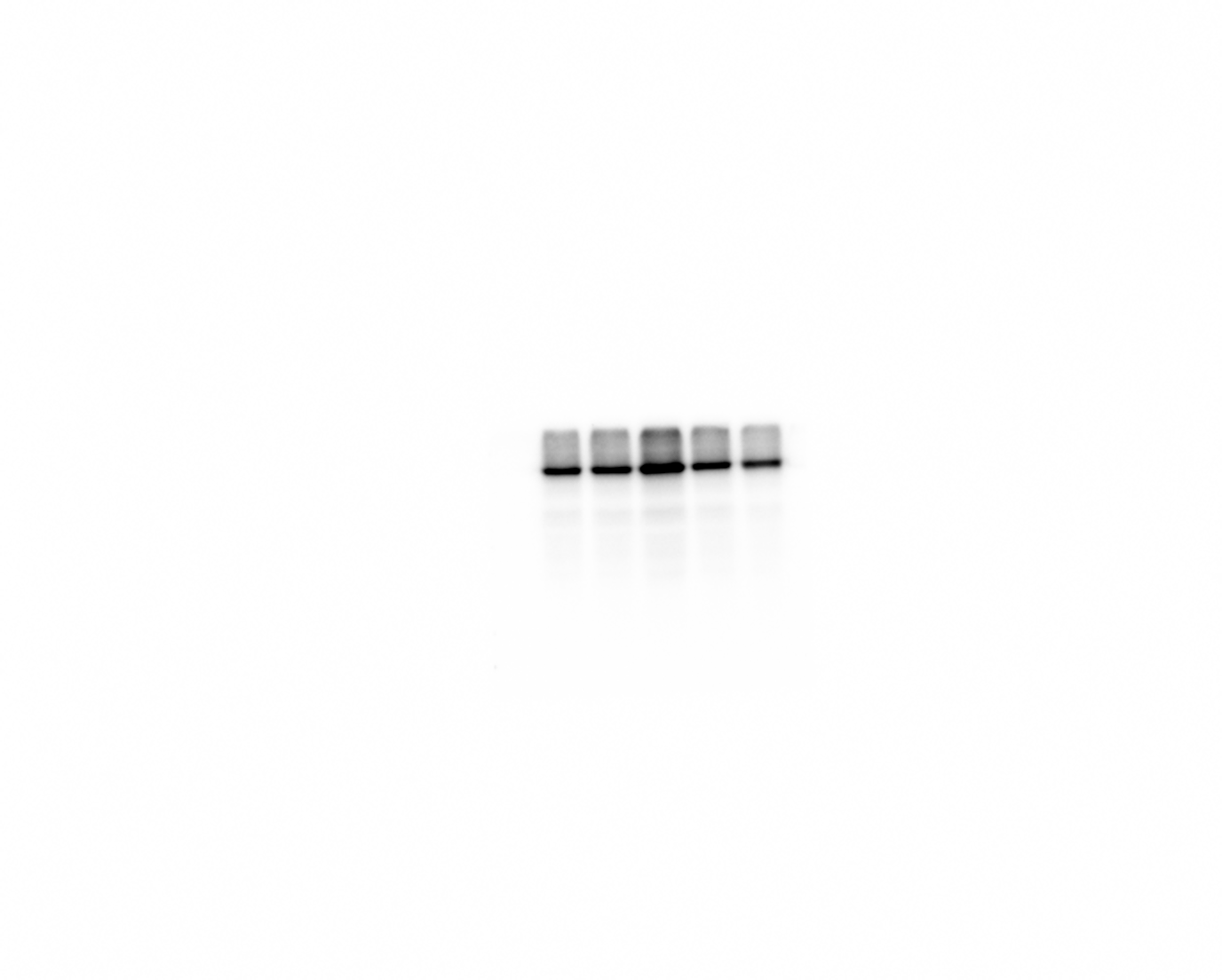

Supplement: Supplementary file 1 [file biomolecules-16-00868-s001.zip › FigureS1 the full, uncropped western blot images/The vitro primary SMCs/LONP1/3.Tif]

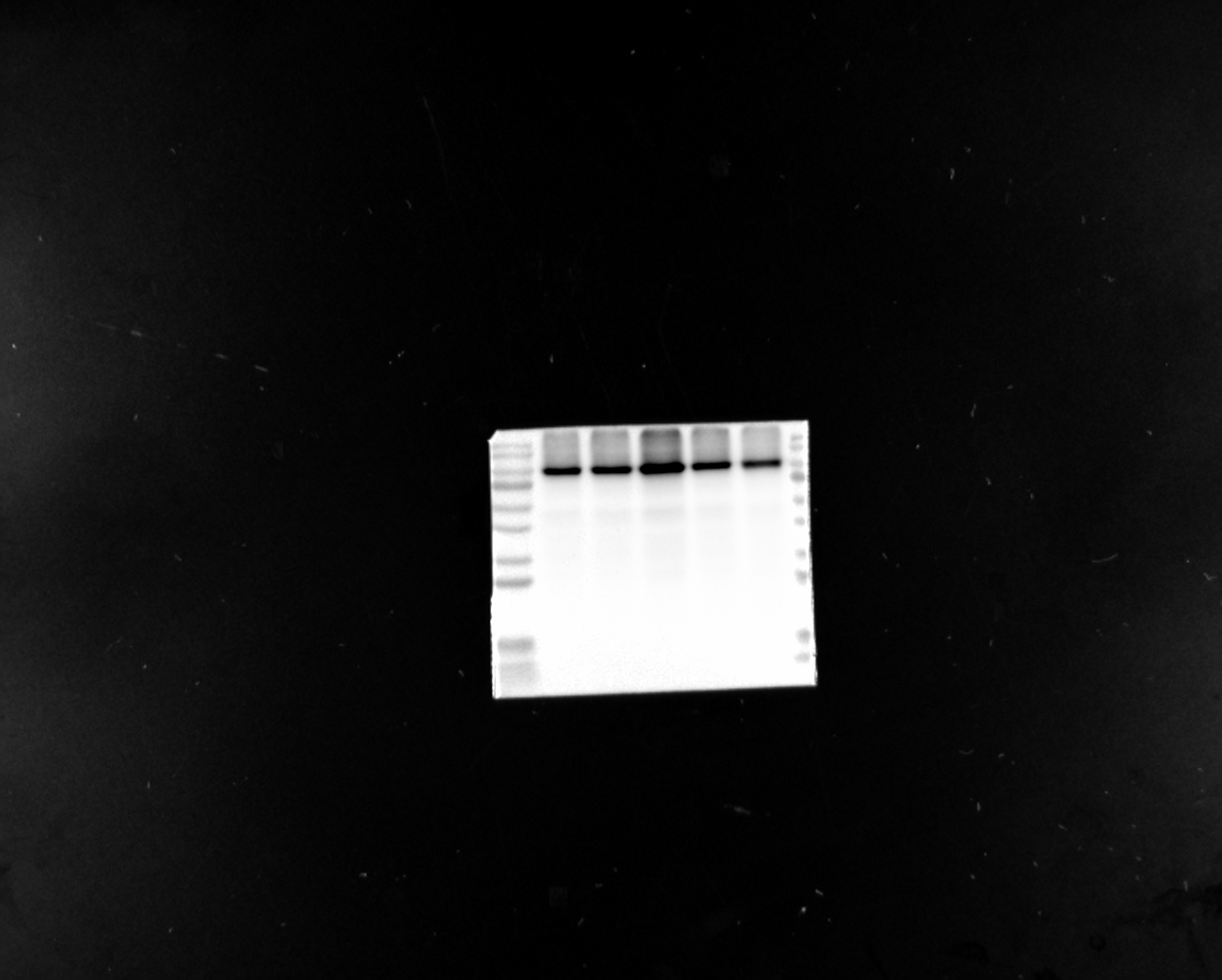

Supplement: Supplementary file 1 [file biomolecules-16-00868-s001.zip › FigureS1 the full, uncropped western blot images/The vitro primary SMCs/LONP1/3副本.Tif]

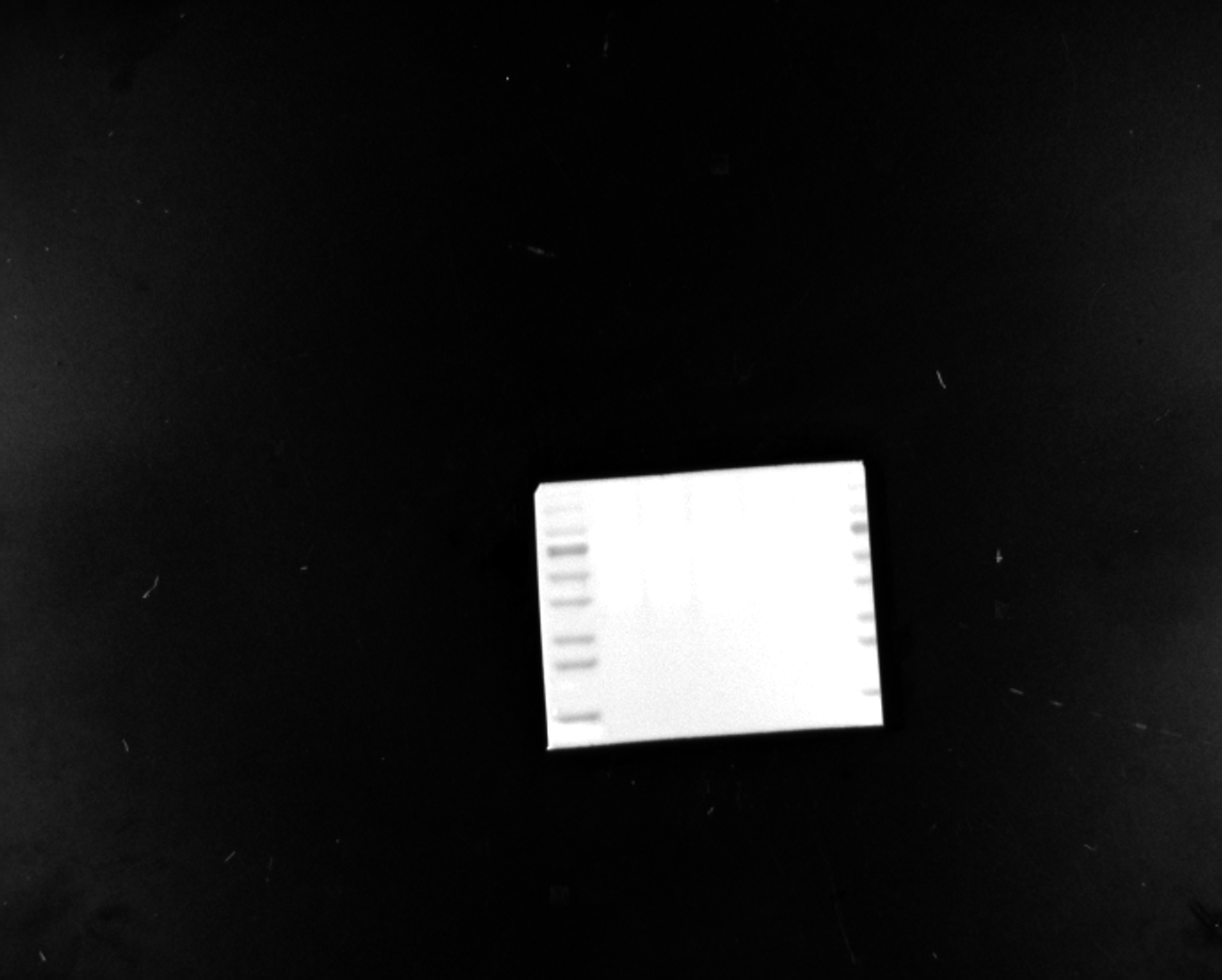

Supplement: Supplementary file 1 [file biomolecules-16-00868-s001.zip › FigureS1 the full, uncropped western blot images/The vitro primary SMCs/mtHSP70/1-t.Tif]

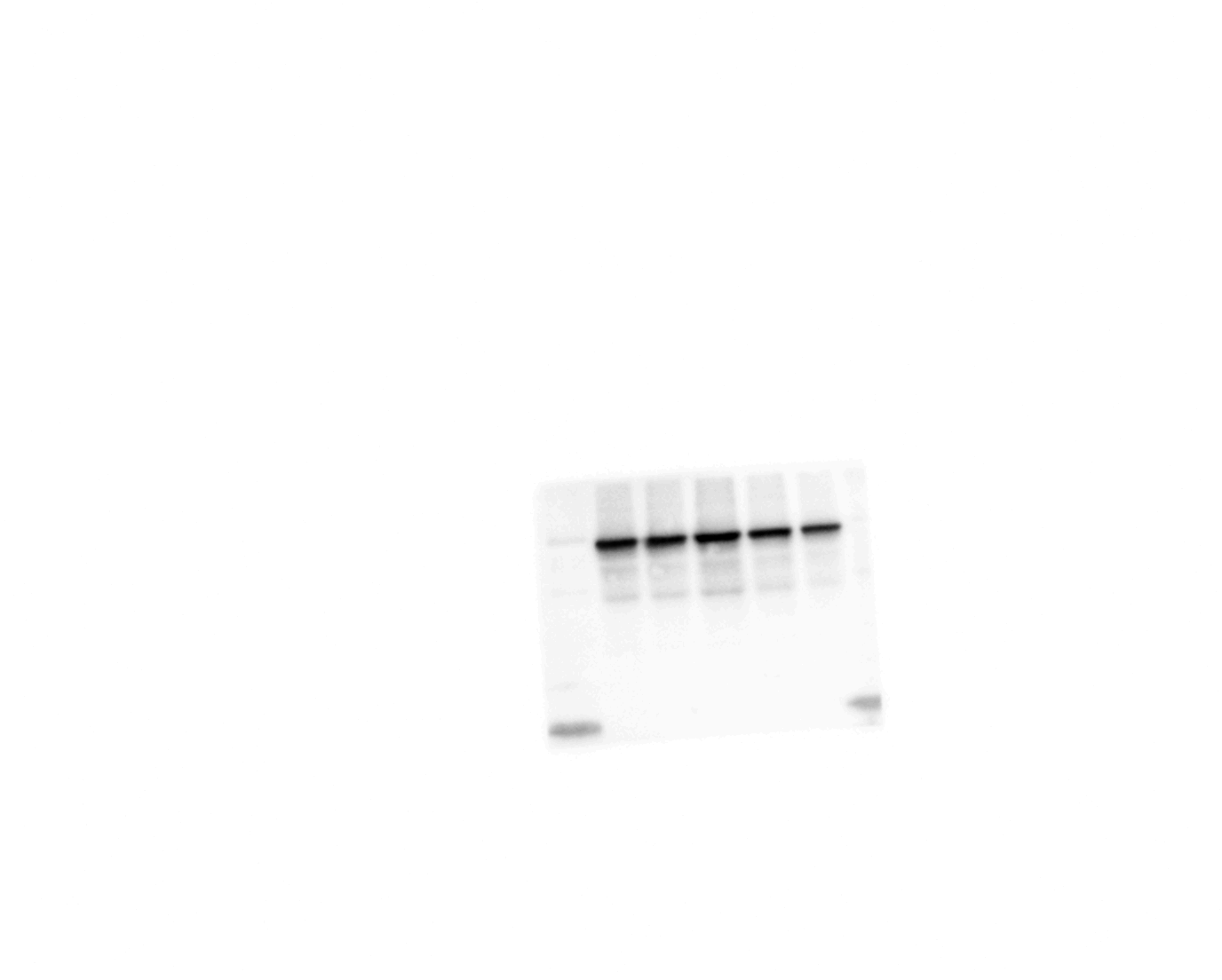

Supplement: Supplementary file 1 [file biomolecules-16-00868-s001.zip › FigureS1 the full, uncropped western blot images/The vitro primary SMCs/mtHSP70/1.Tif]

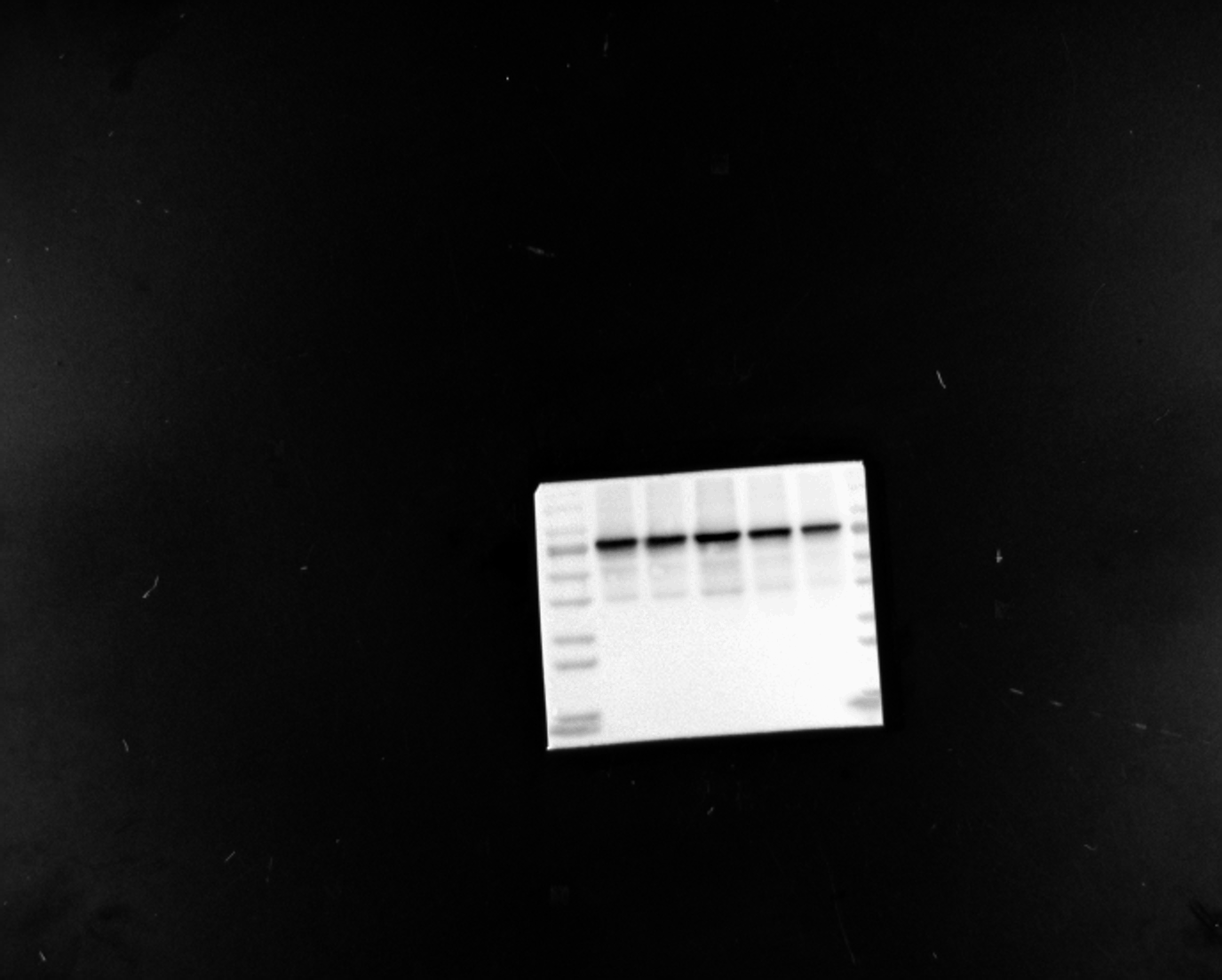

Supplement: Supplementary file 1 [file biomolecules-16-00868-s001.zip › FigureS1 the full, uncropped western blot images/The vitro primary SMCs/mtHSP70/1副本.Tif]

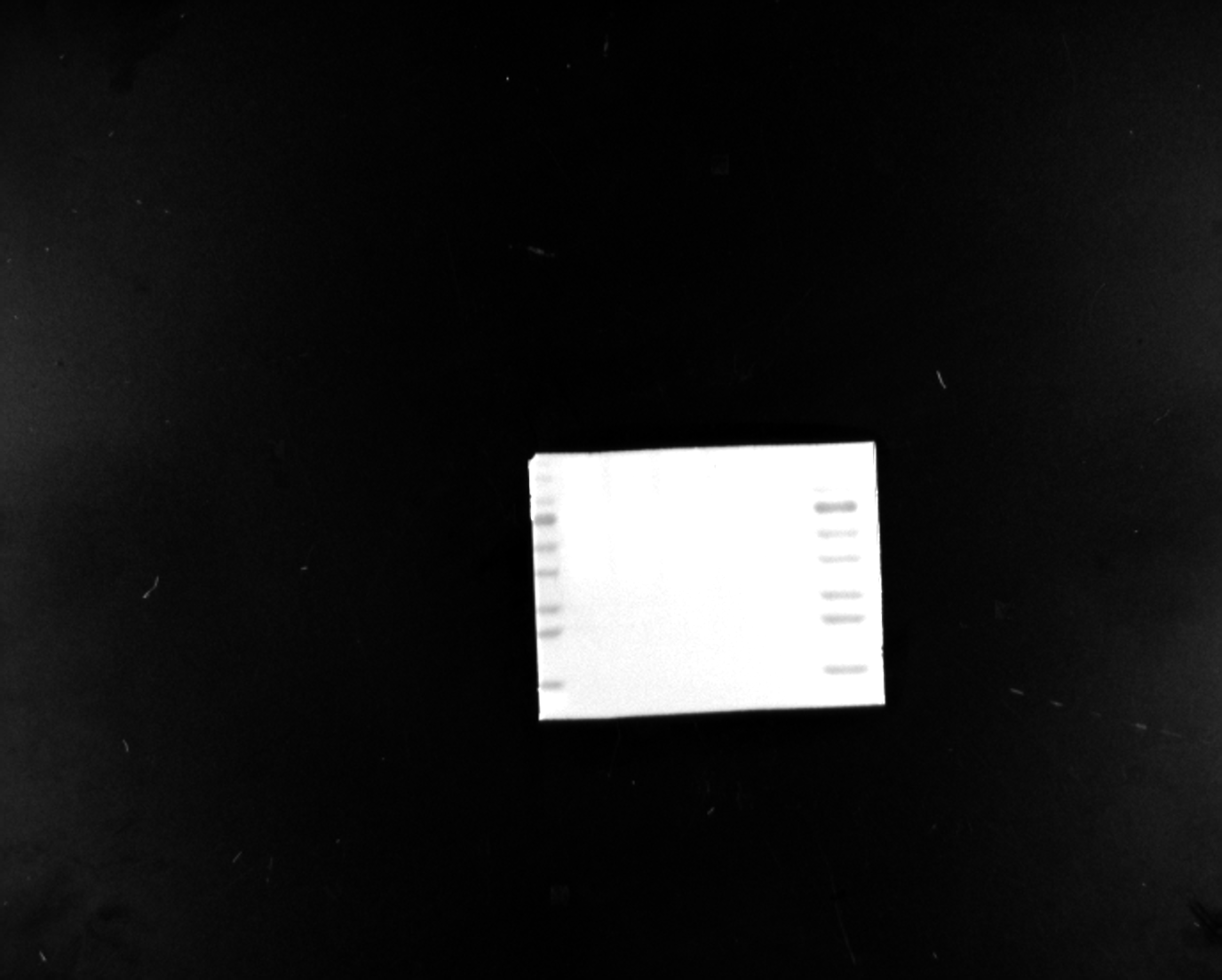

Supplement: Supplementary file 1 [file biomolecules-16-00868-s001.zip › FigureS1 the full, uncropped western blot images/The vitro primary SMCs/mtHSP70/2-t.Tif]

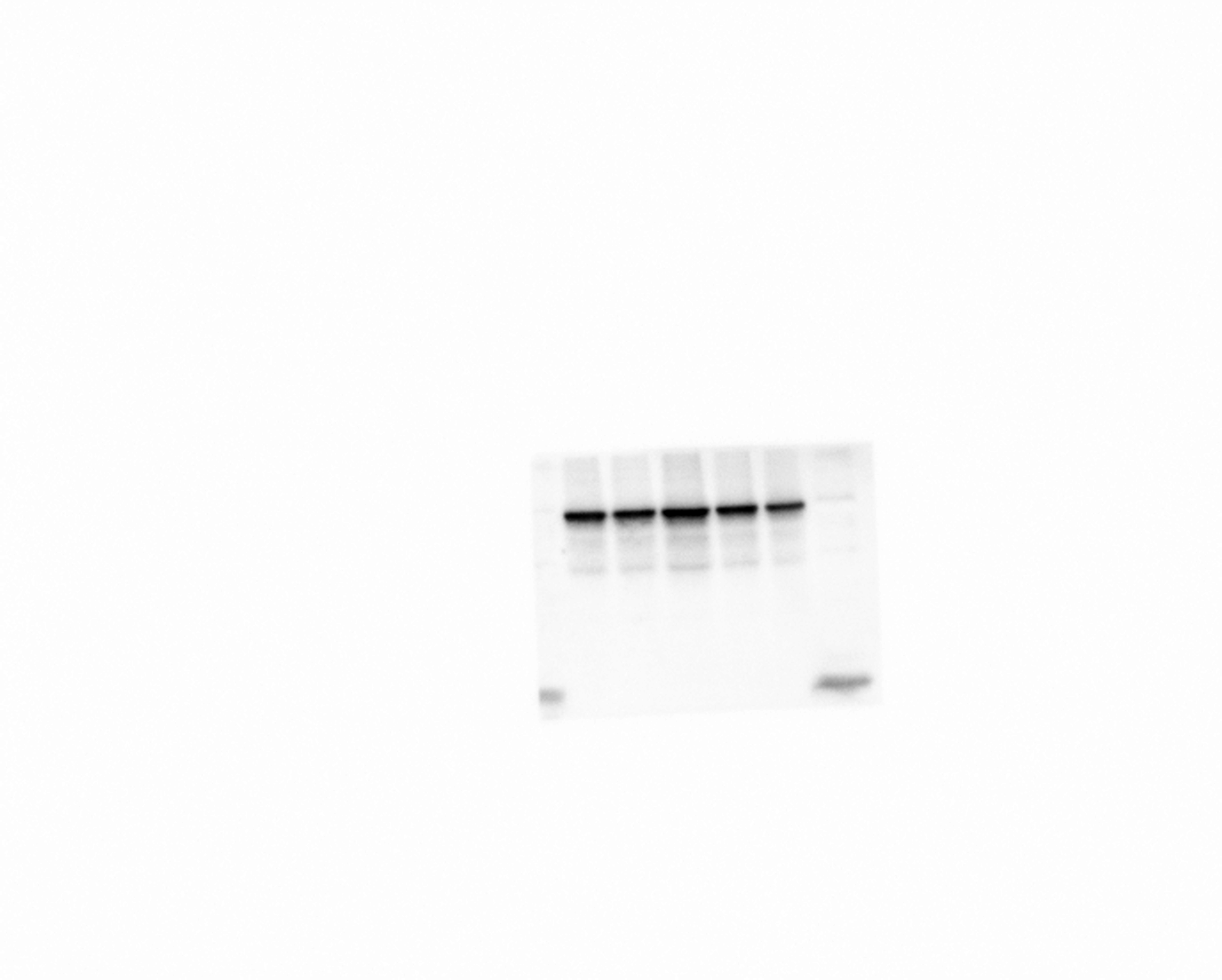

Supplement: Supplementary file 1 [file biomolecules-16-00868-s001.zip › FigureS1 the full, uncropped western blot images/The vitro primary SMCs/mtHSP70/2.Tif]

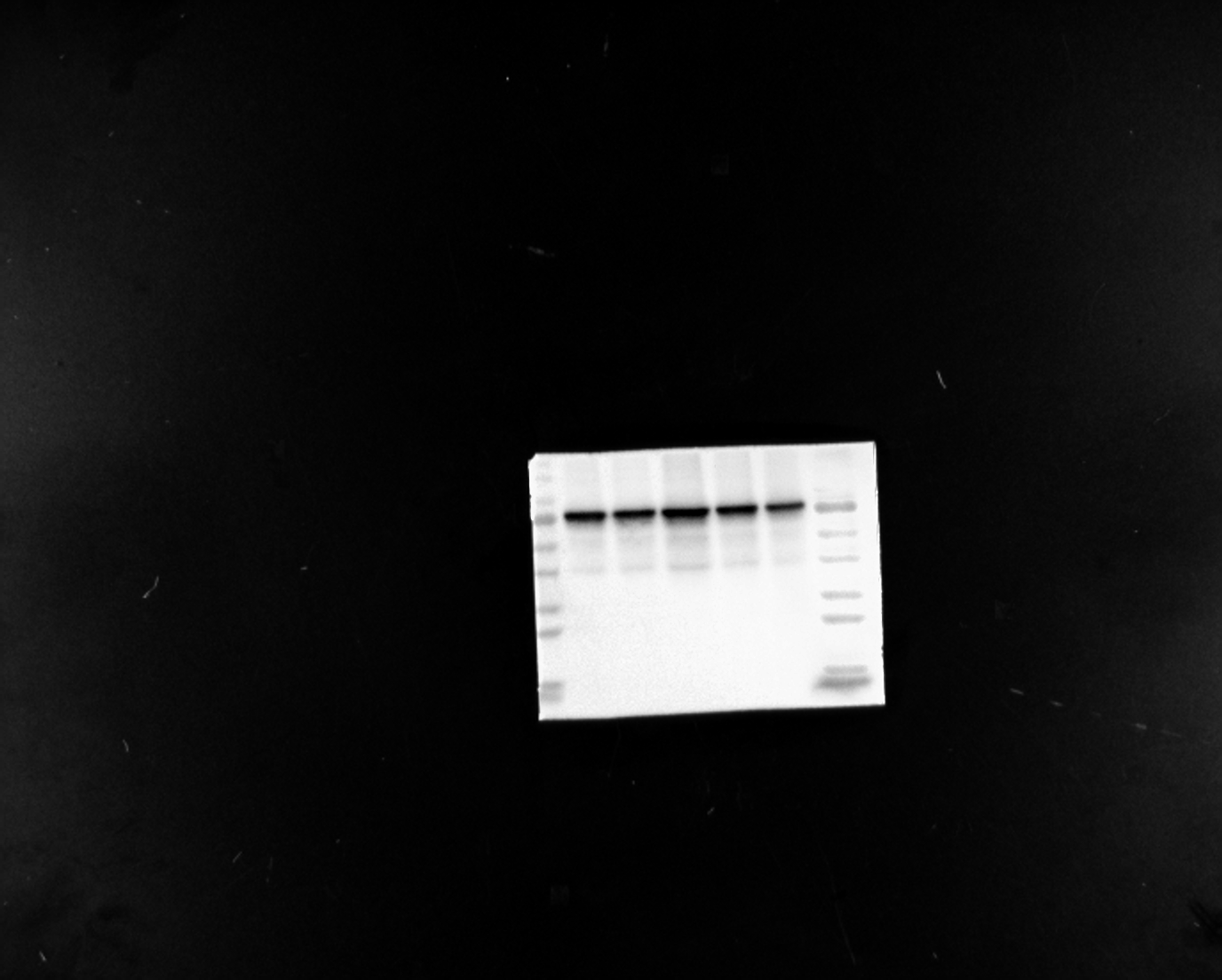

Supplement: Supplementary file 1 [file biomolecules-16-00868-s001.zip › FigureS1 the full, uncropped western blot images/The vitro primary SMCs/mtHSP70/2副本.Tif]

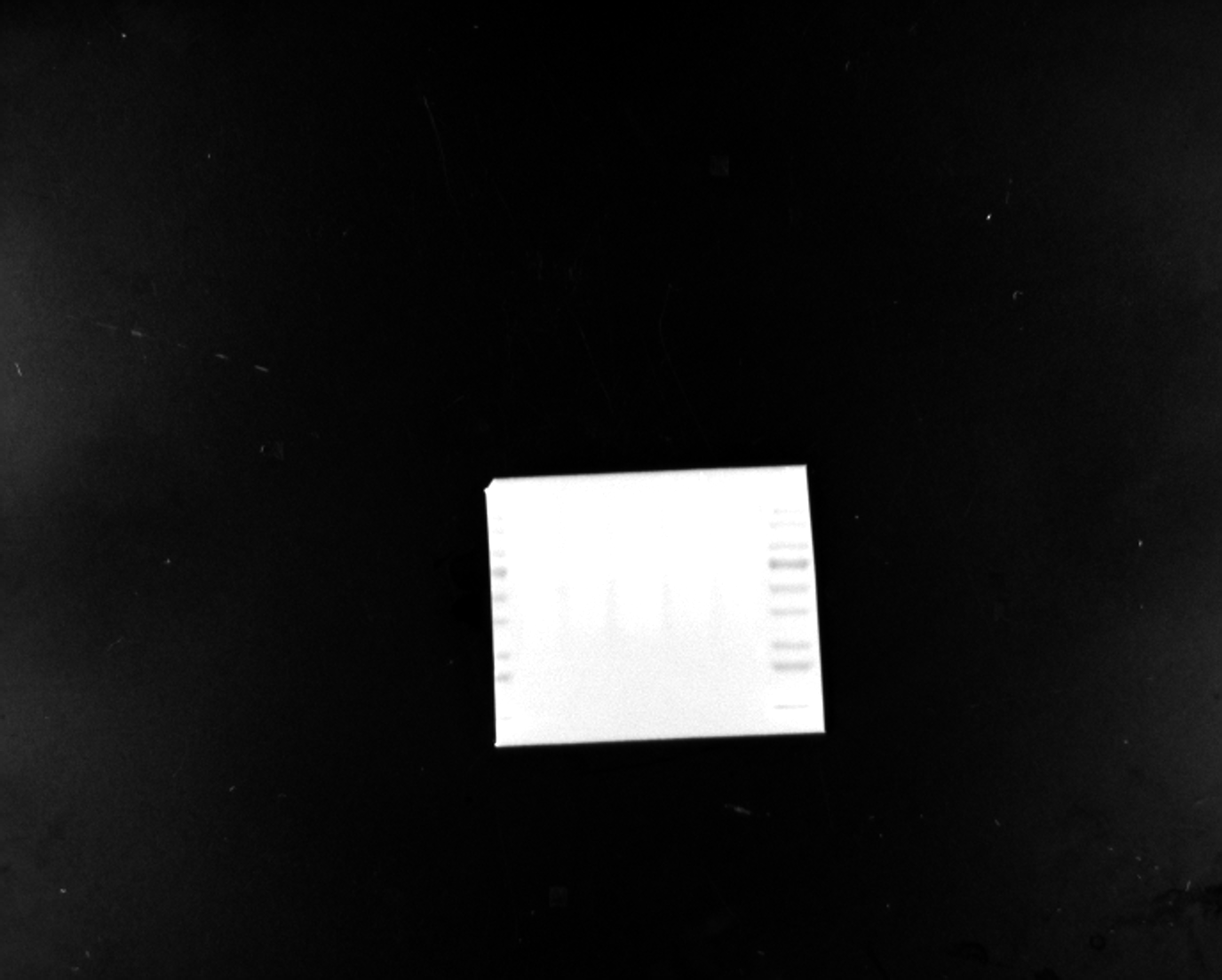

Supplement: Supplementary file 1 [file biomolecules-16-00868-s001.zip › FigureS1 the full, uncropped western blot images/The vitro primary SMCs/mtHSP70/3-t.Tif]

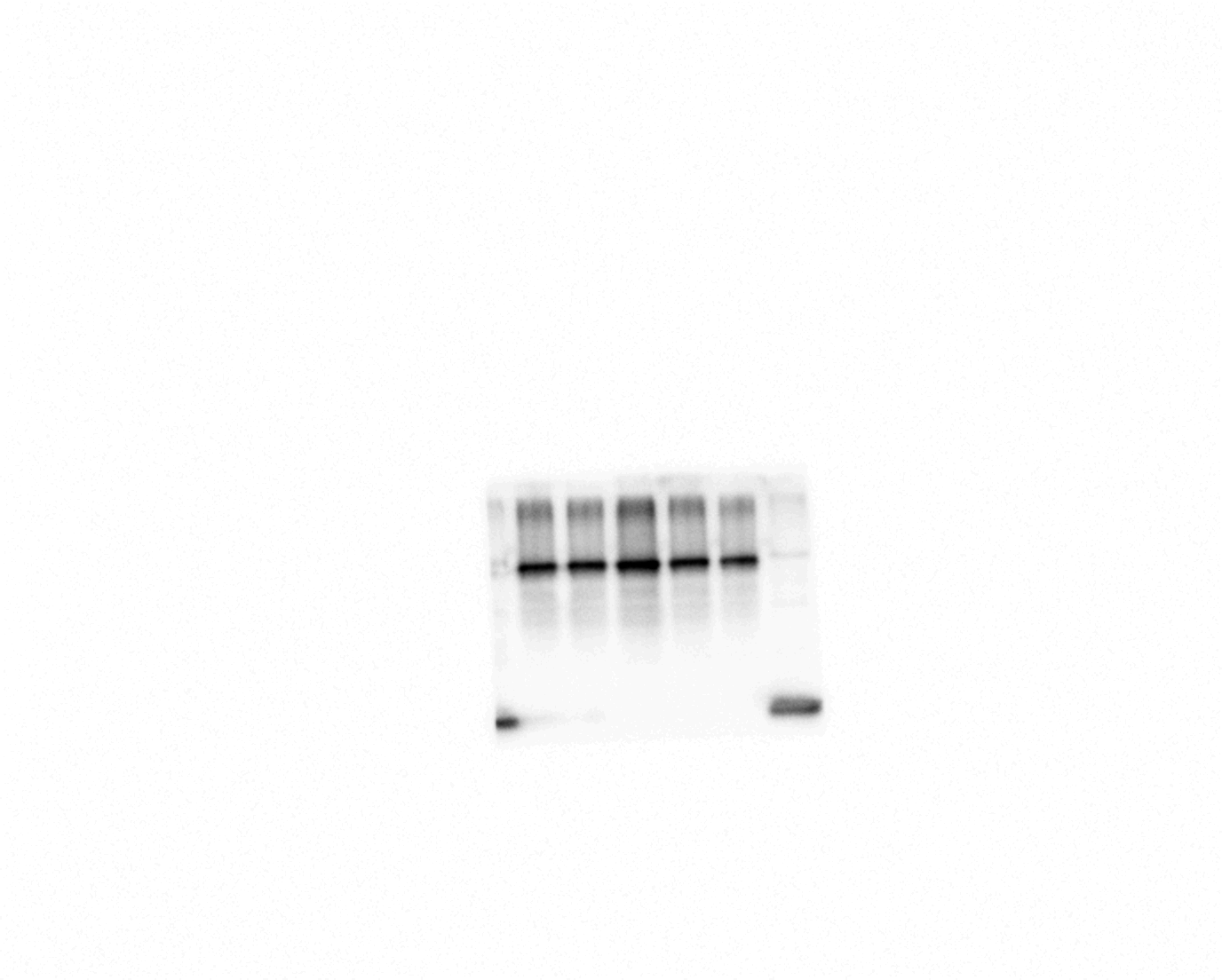

Supplement: Supplementary file 1 [file biomolecules-16-00868-s001.zip › FigureS1 the full, uncropped western blot images/The vitro primary SMCs/mtHSP70/3.Tif]

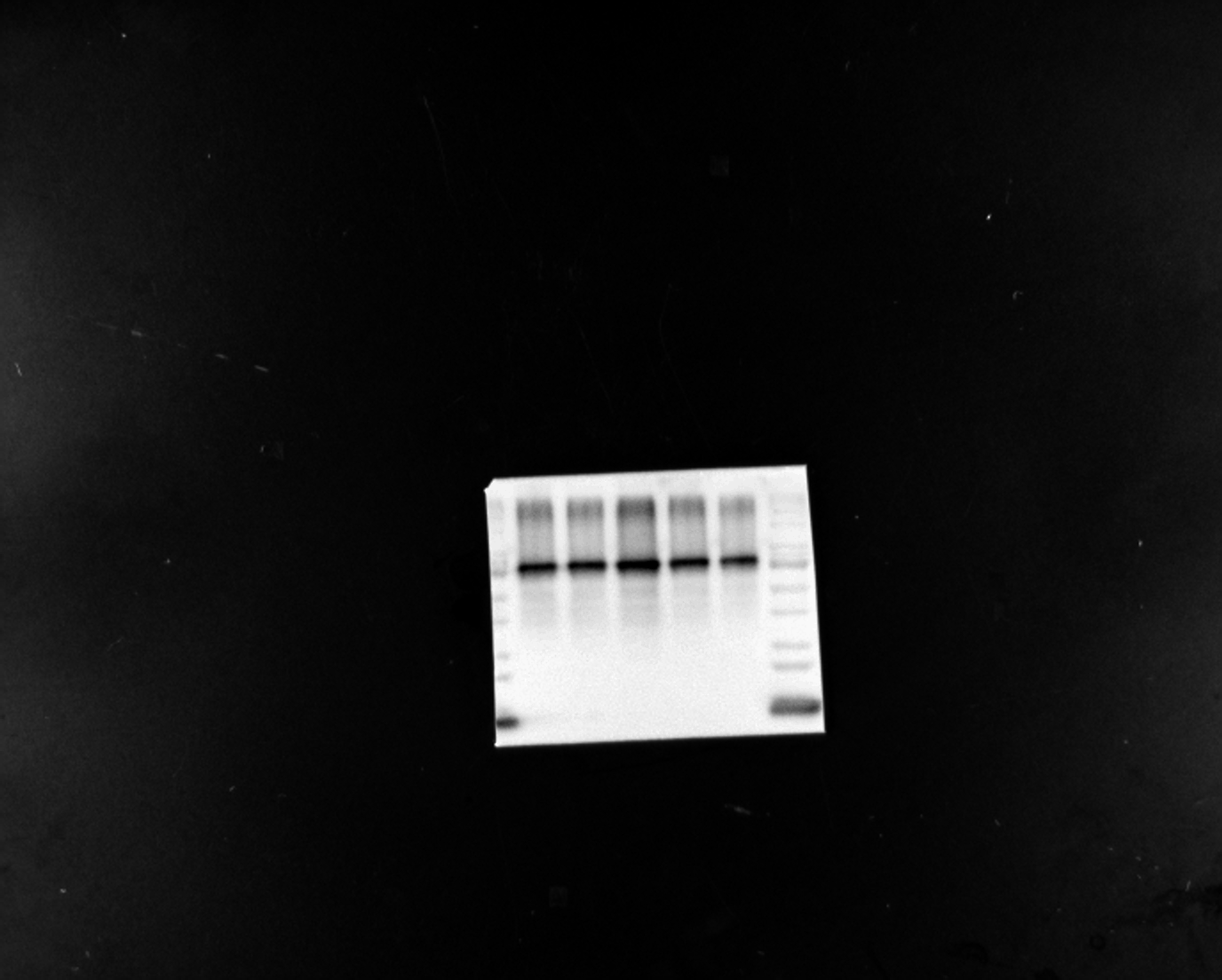

Supplement: Supplementary file 1 [file biomolecules-16-00868-s001.zip › FigureS1 the full, uncropped western blot images/The vitro primary SMCs/mtHSP70/3副本.Tif]

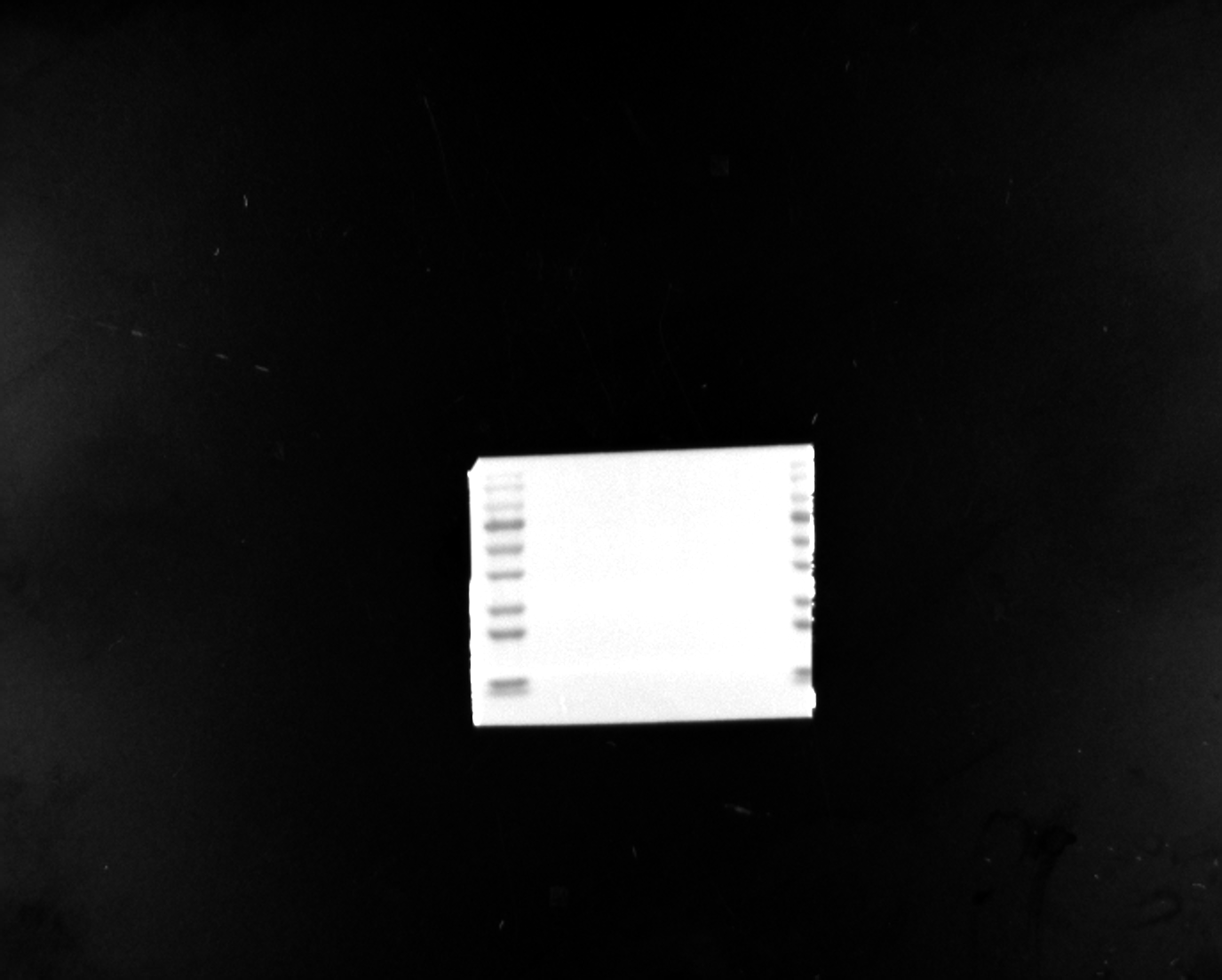

Supplement: Supplementary file 1 [file biomolecules-16-00868-s001.zip › FigureS1 the full, uncropped western blot images/The vitro primary SMCs/NDUFA9/1-t.Tif]

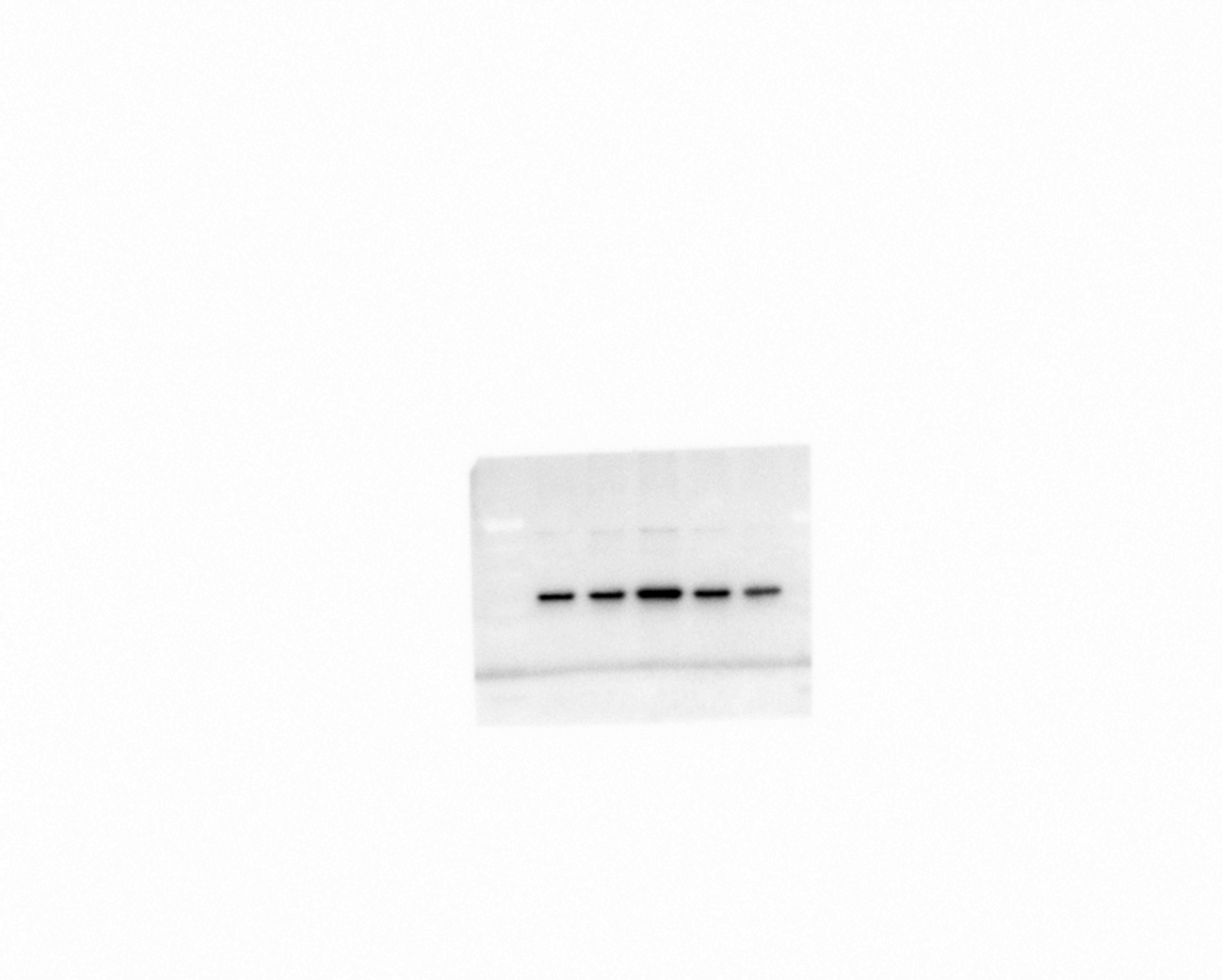

Supplement: Supplementary file 1 [file biomolecules-16-00868-s001.zip › FigureS1 the full, uncropped western blot images/The vitro primary SMCs/NDUFA9/1.Tif]

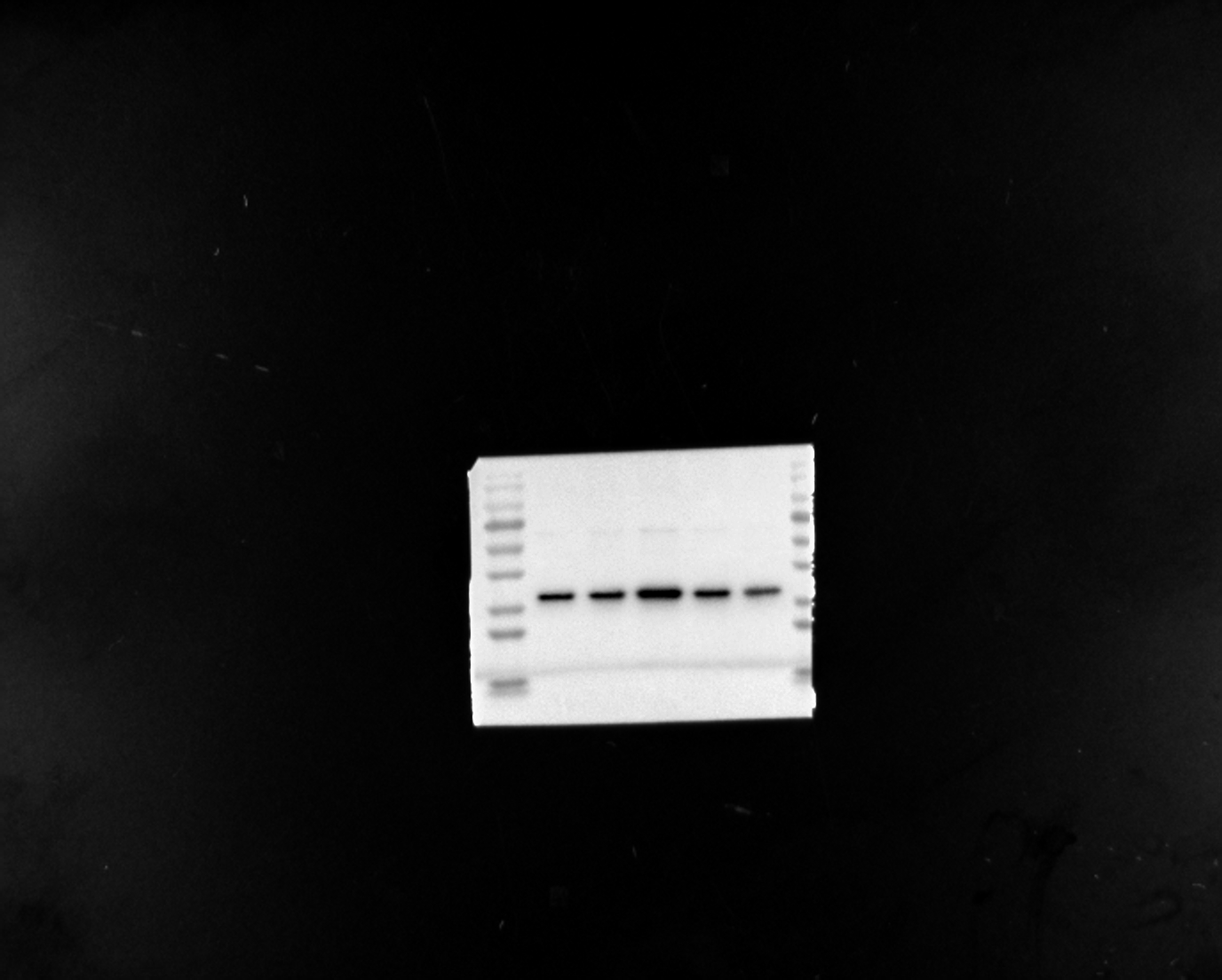

Supplement: Supplementary file 1 [file biomolecules-16-00868-s001.zip › FigureS1 the full, uncropped western blot images/The vitro primary SMCs/NDUFA9/1副本.Tif]

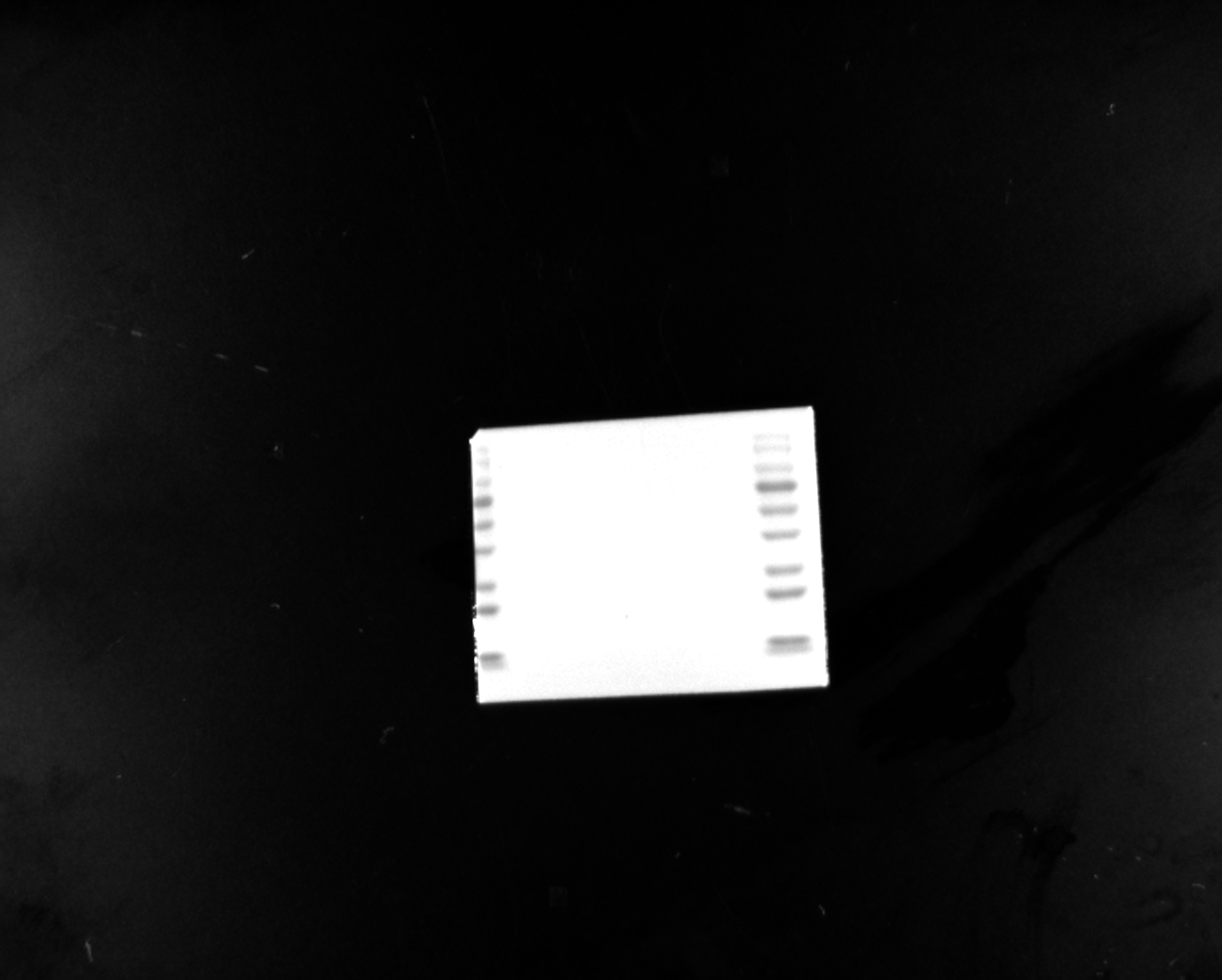

Supplement: Supplementary file 1 [file biomolecules-16-00868-s001.zip › FigureS1 the full, uncropped western blot images/The vitro primary SMCs/NDUFA9/2-t.Tif]

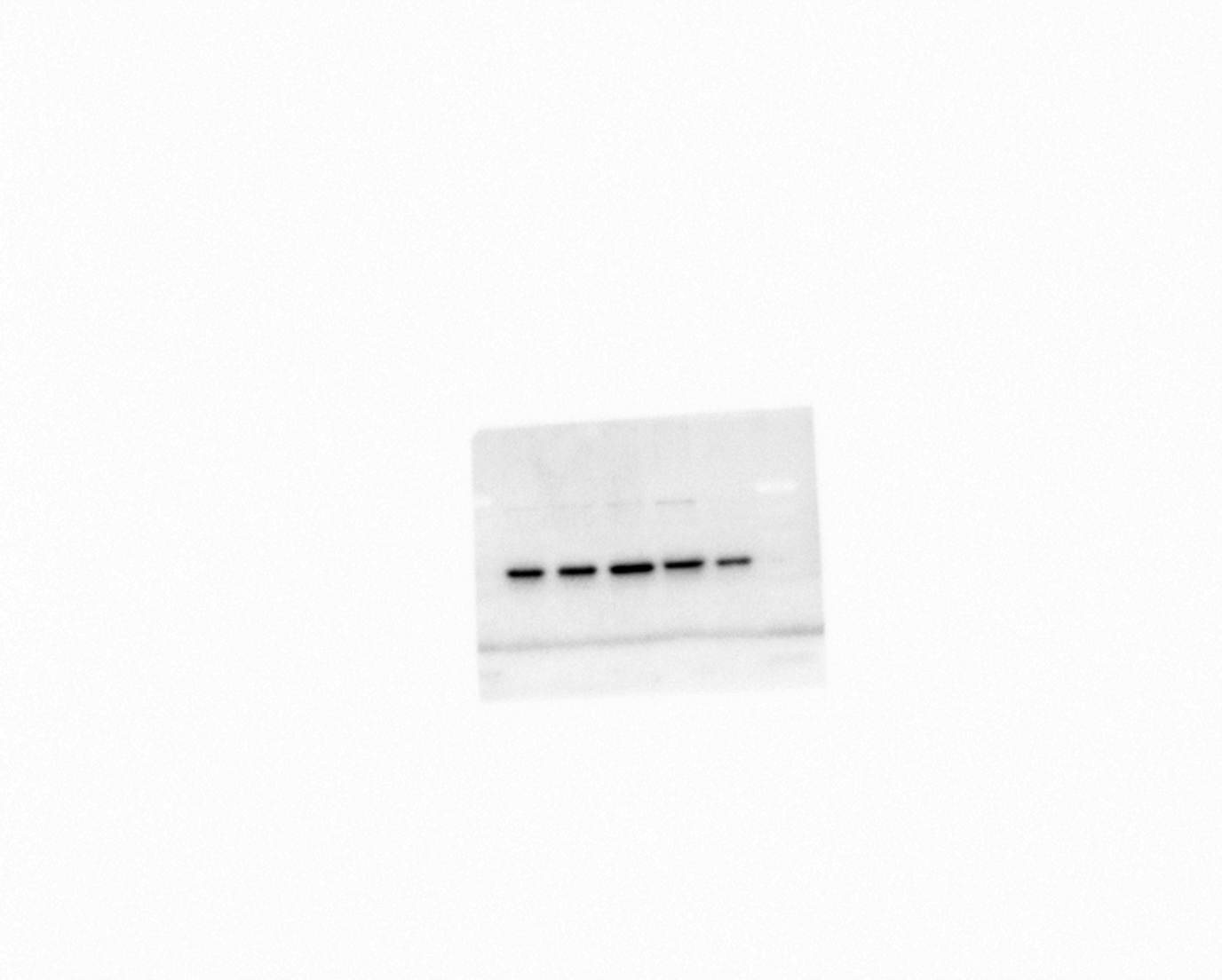

Supplement: Supplementary file 1 [file biomolecules-16-00868-s001.zip › FigureS1 the full, uncropped western blot images/The vitro primary SMCs/NDUFA9/2.Tif]

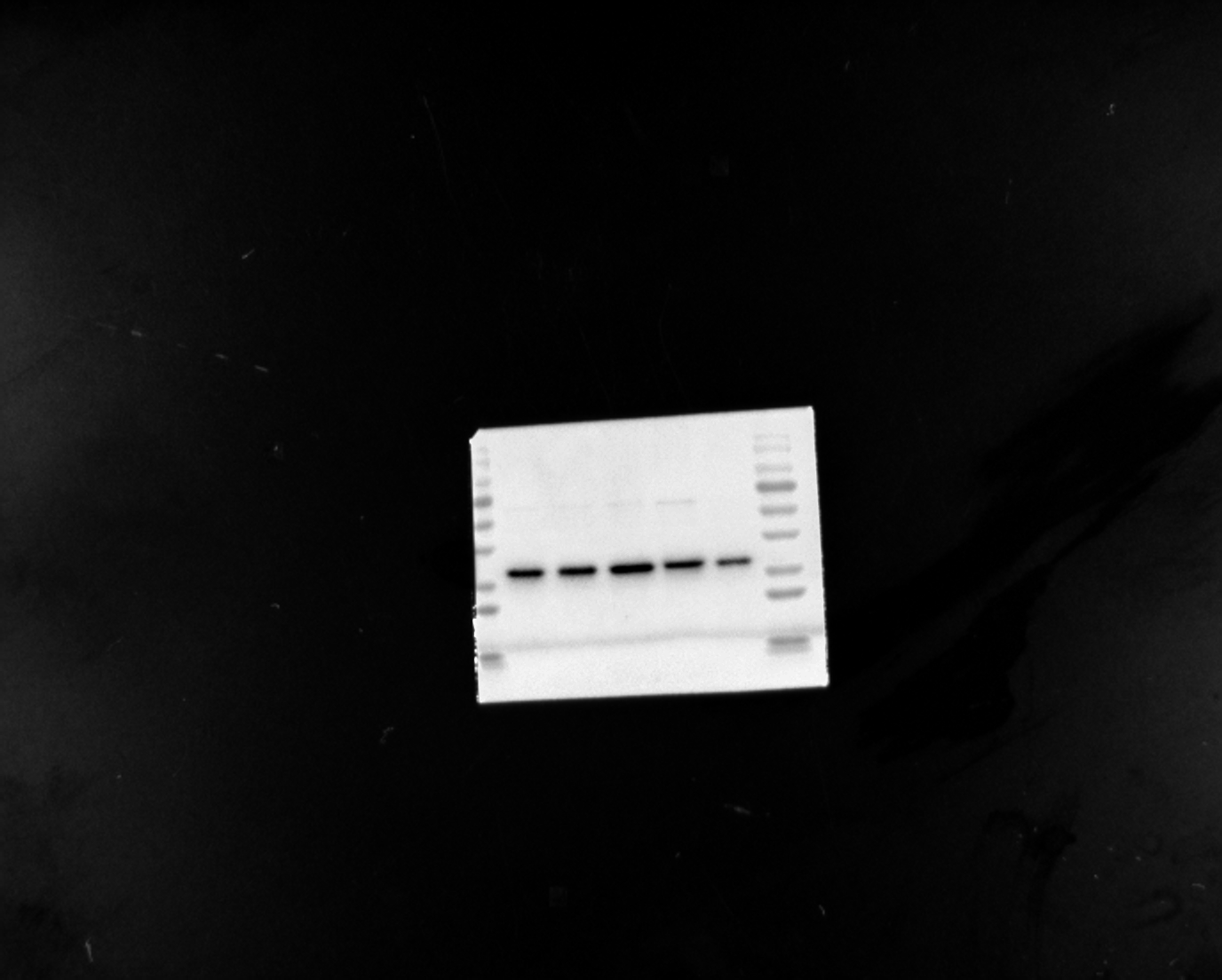

Supplement: Supplementary file 1 [file biomolecules-16-00868-s001.zip › FigureS1 the full, uncropped western blot images/The vitro primary SMCs/NDUFA9/2副本.Tif]

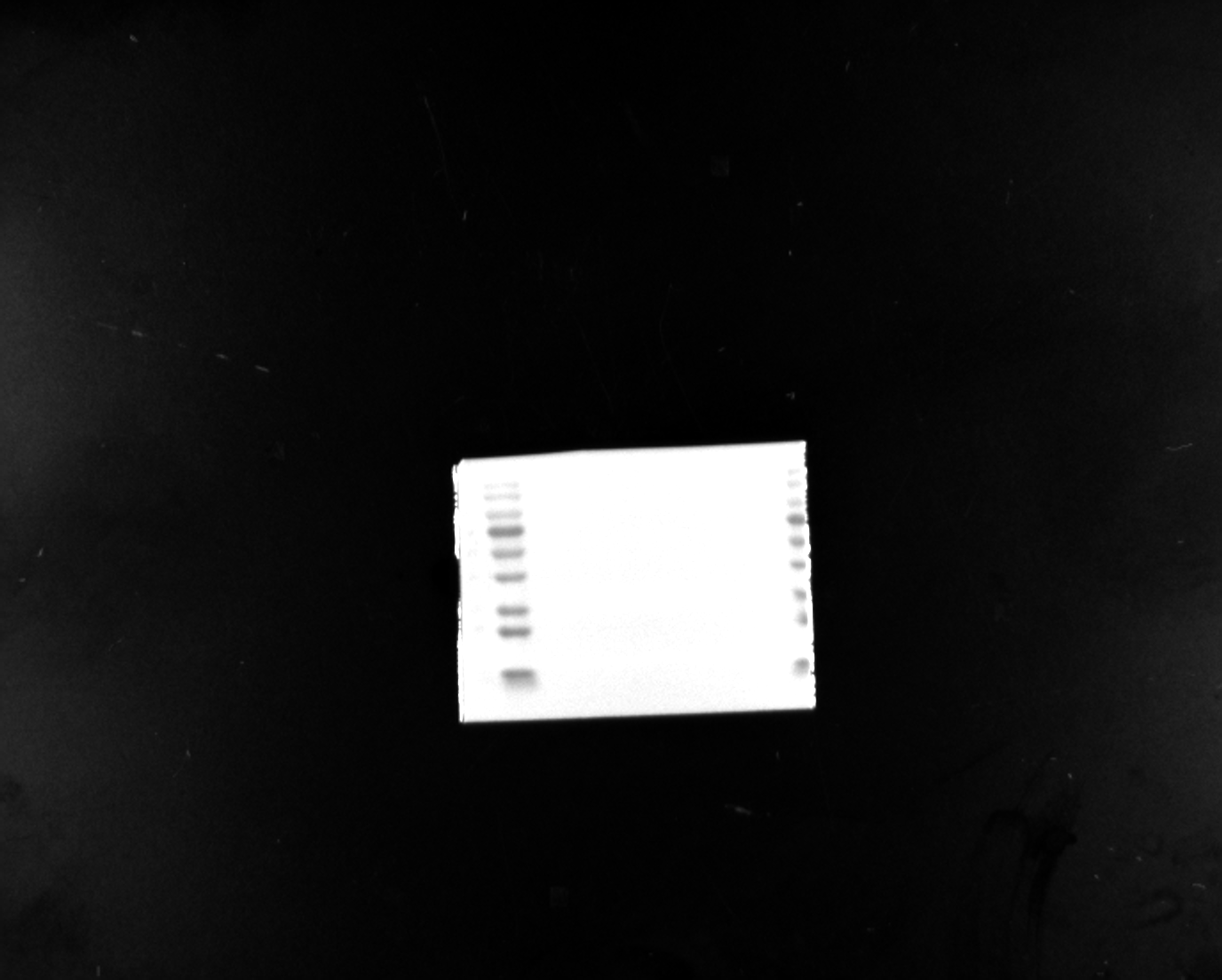

Supplement: Supplementary file 1 [file biomolecules-16-00868-s001.zip › FigureS1 the full, uncropped western blot images/The vitro primary SMCs/NDUFA9/3-t.Tif]

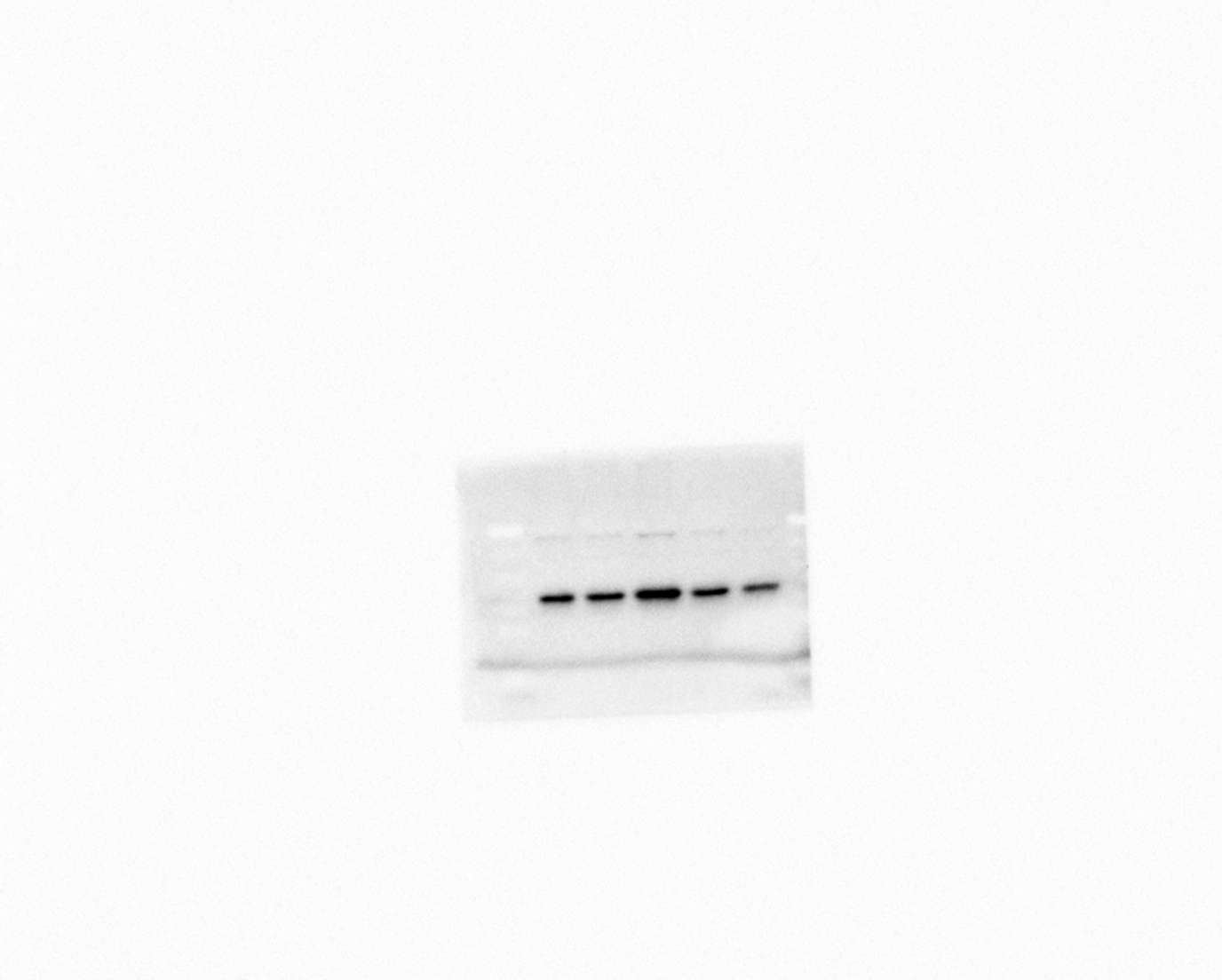

Supplement: Supplementary file 1 [file biomolecules-16-00868-s001.zip › FigureS1 the full, uncropped western blot images/The vitro primary SMCs/NDUFA9/3.Tif]

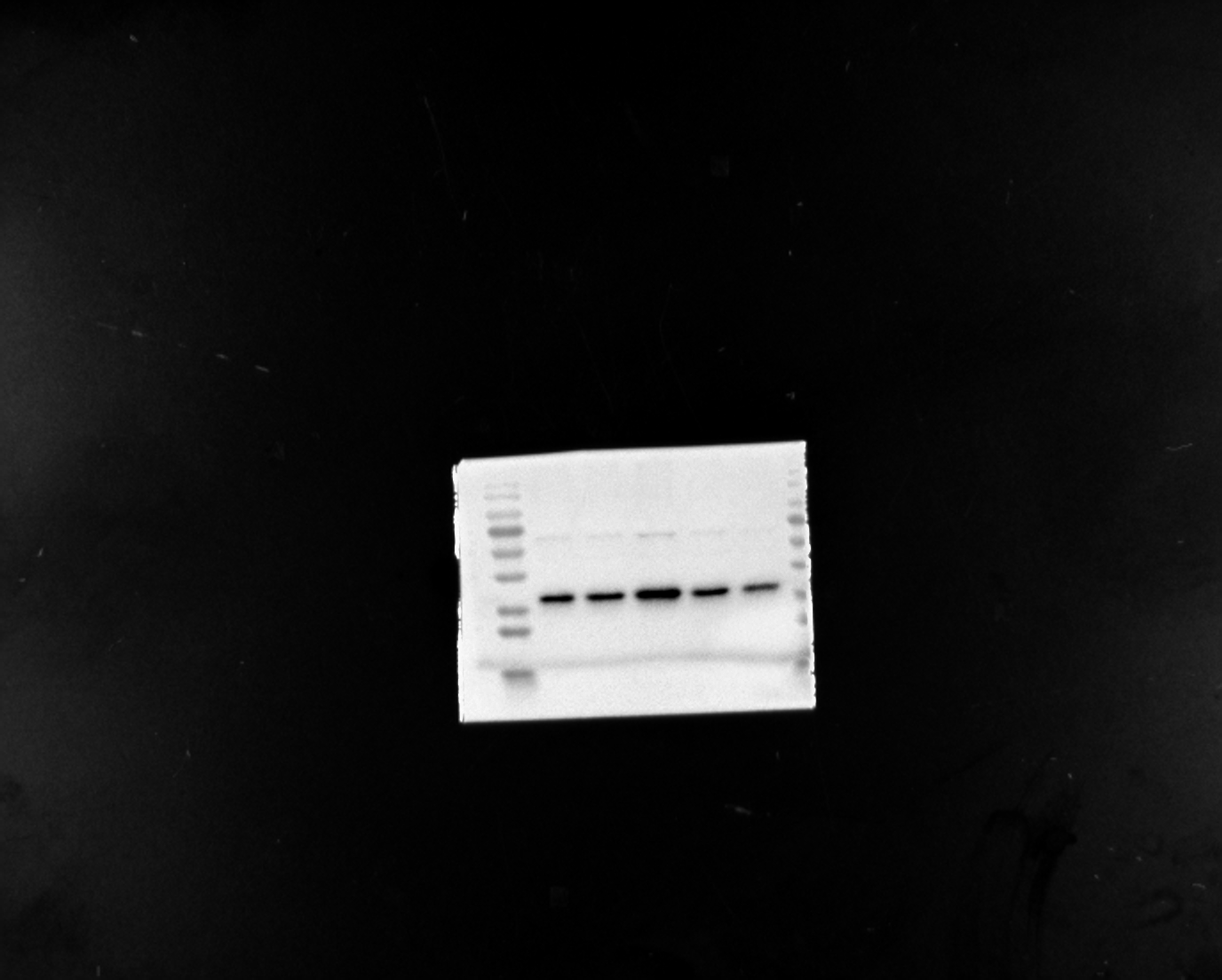

Supplement: Supplementary file 1 [file biomolecules-16-00868-s001.zip › FigureS1 the full, uncropped western blot images/The vitro primary SMCs/NDUFA9/3副本.Tif]

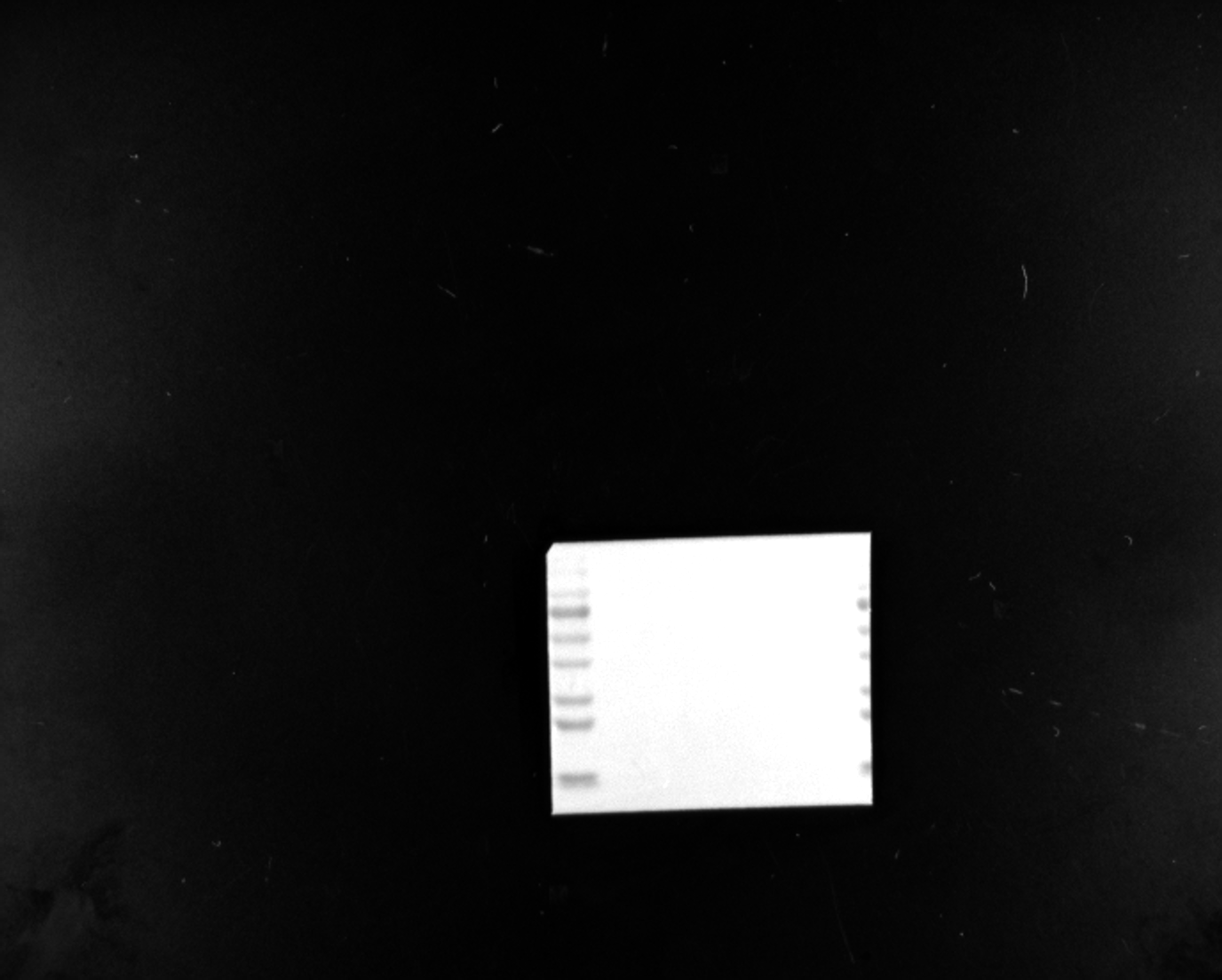

Supplement: Supplementary file 1 [file biomolecules-16-00868-s001.zip › FigureS1 the full, uncropped western blot images/The vitro primary SMCs/SDHA/1-t.Tif]

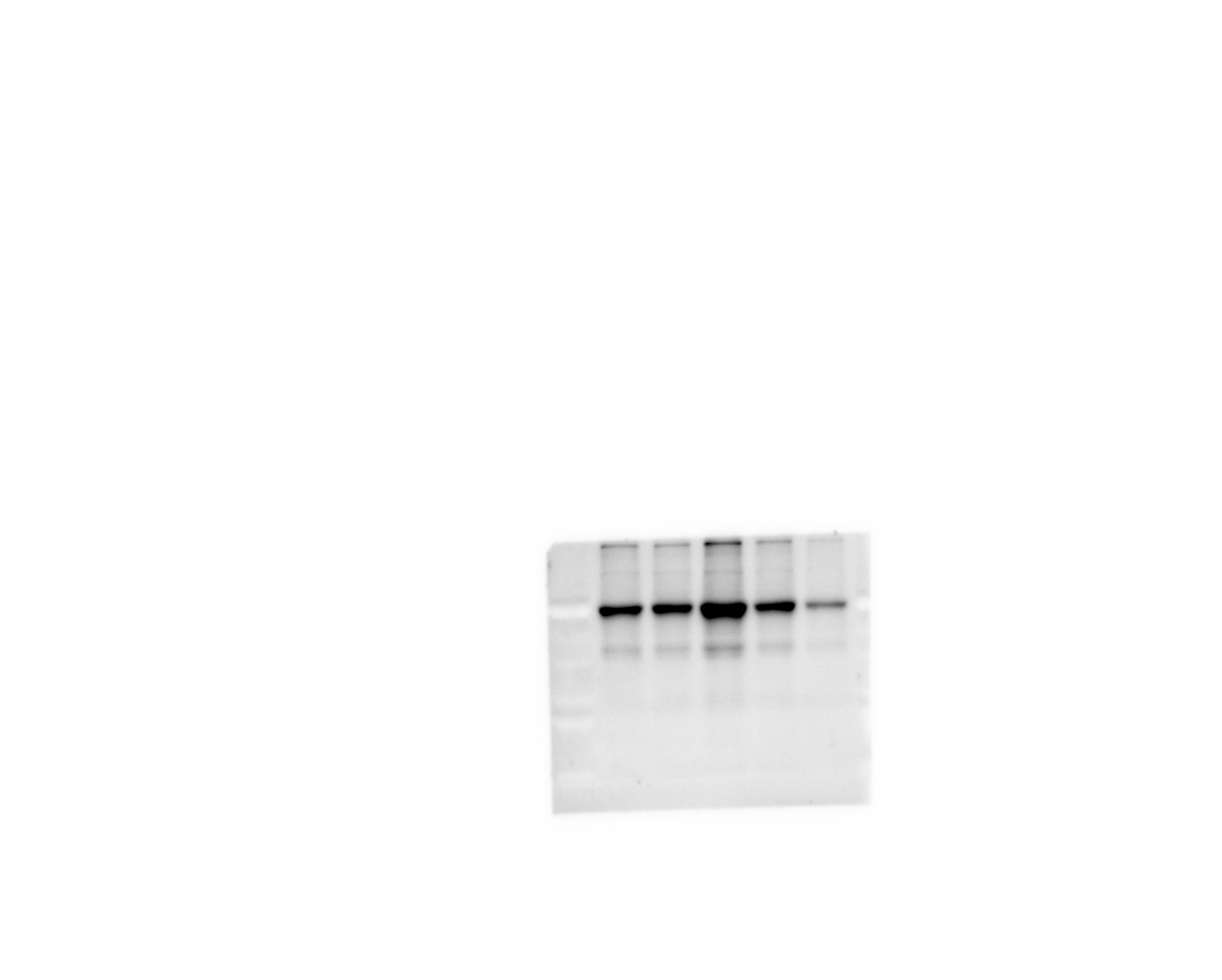

Supplement: Supplementary file 1 [file biomolecules-16-00868-s001.zip › FigureS1 the full, uncropped western blot images/The vitro primary SMCs/SDHA/1.Tif]

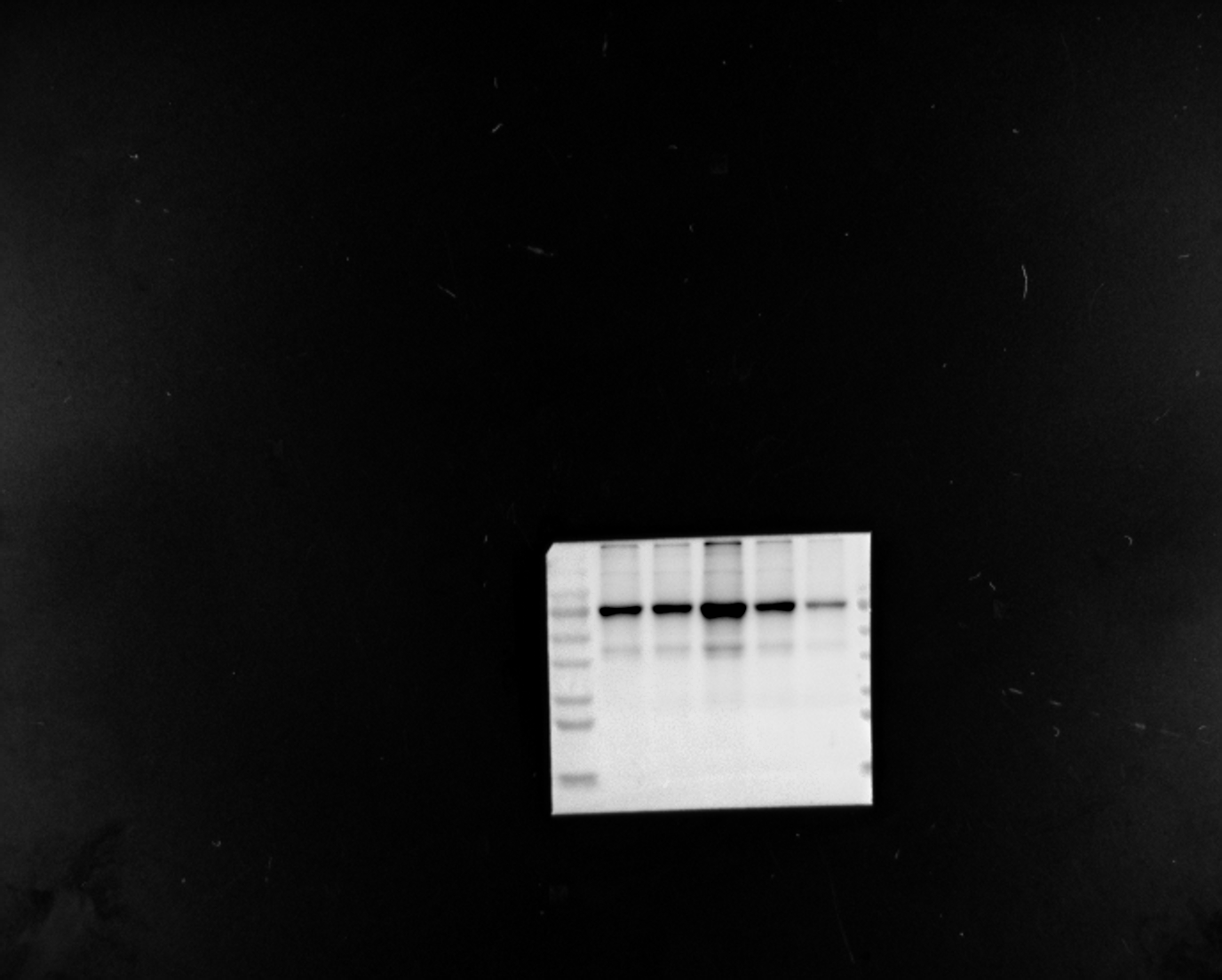

Supplement: Supplementary file 1 [file biomolecules-16-00868-s001.zip › FigureS1 the full, uncropped western blot images/The vitro primary SMCs/SDHA/1副本.Tif]

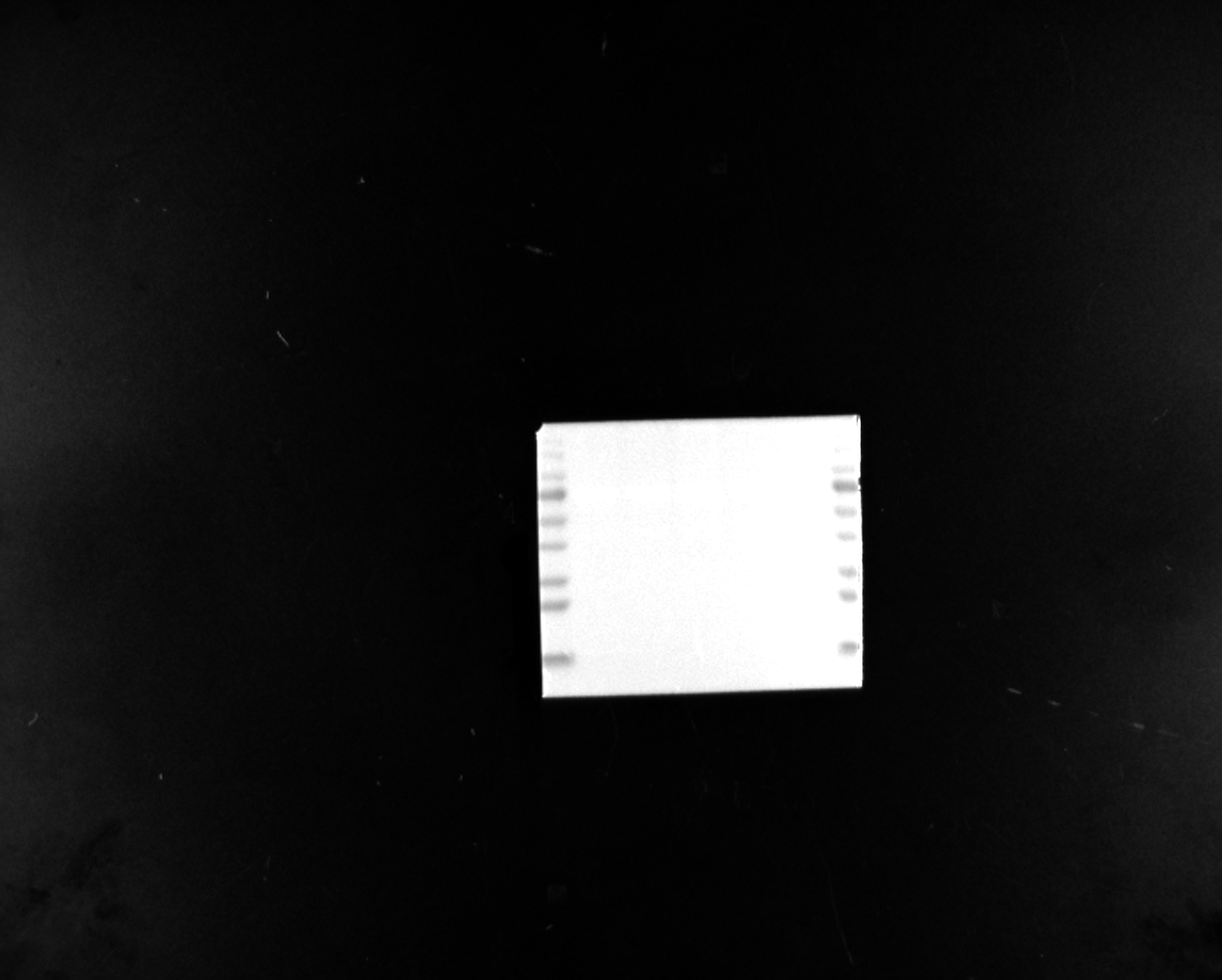

Supplement: Supplementary file 1 [file biomolecules-16-00868-s001.zip › FigureS1 the full, uncropped western blot images/The vitro primary SMCs/SDHA/2-t.Tif]

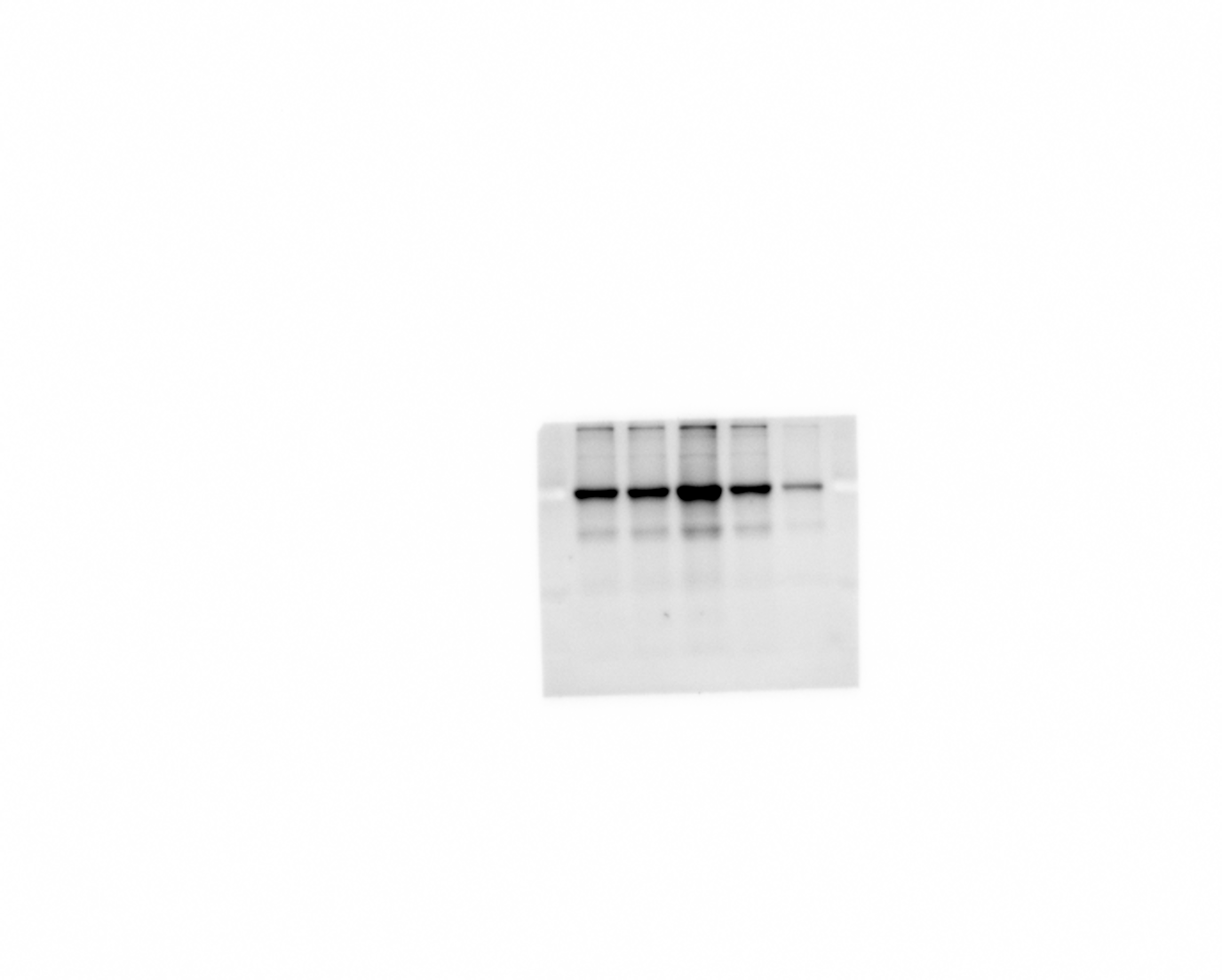

Supplement: Supplementary file 1 [file biomolecules-16-00868-s001.zip › FigureS1 the full, uncropped western blot images/The vitro primary SMCs/SDHA/2.Tif]

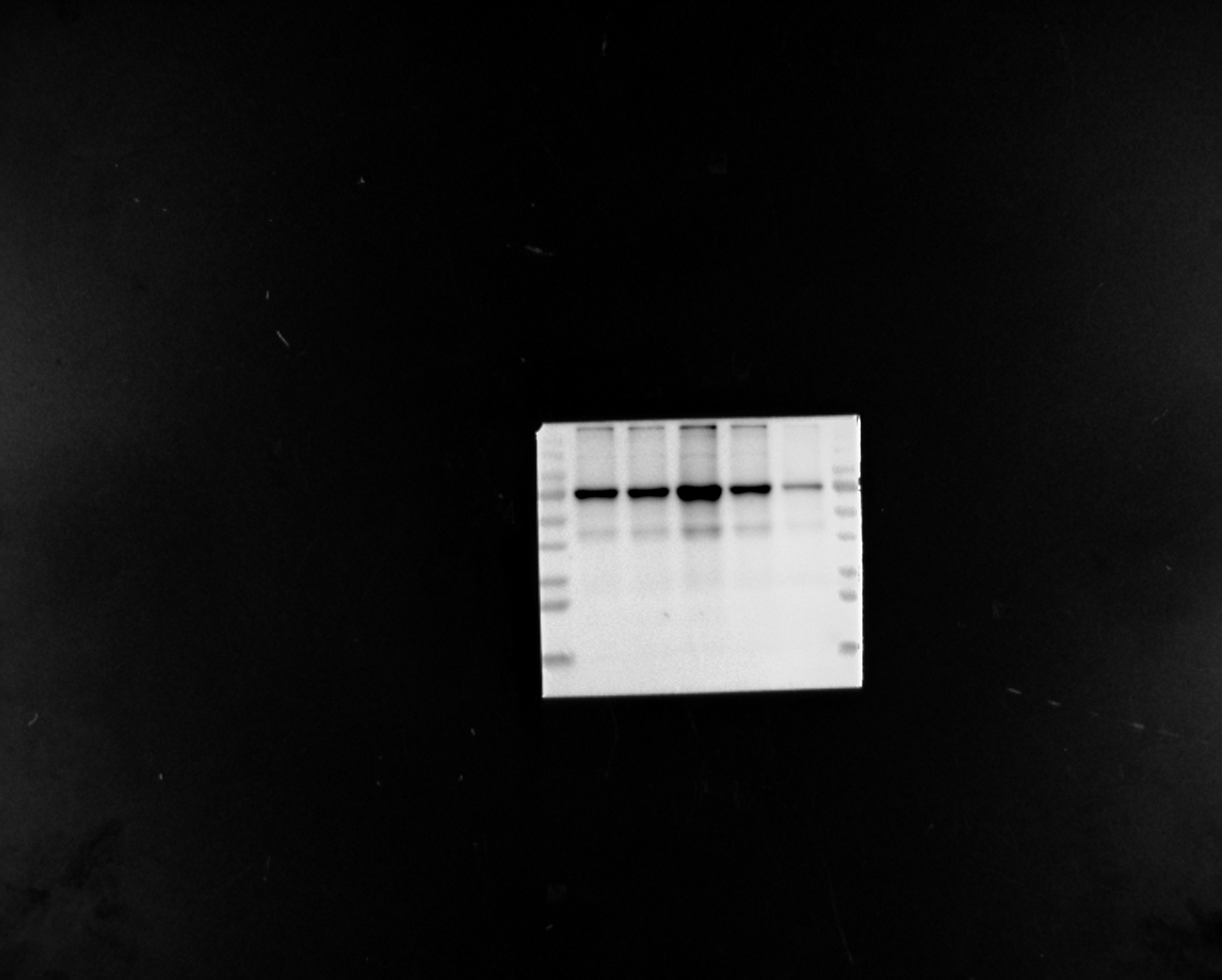

Supplement: Supplementary file 1 [file biomolecules-16-00868-s001.zip › FigureS1 the full, uncropped western blot images/The vitro primary SMCs/SDHA/2副本.Tif]

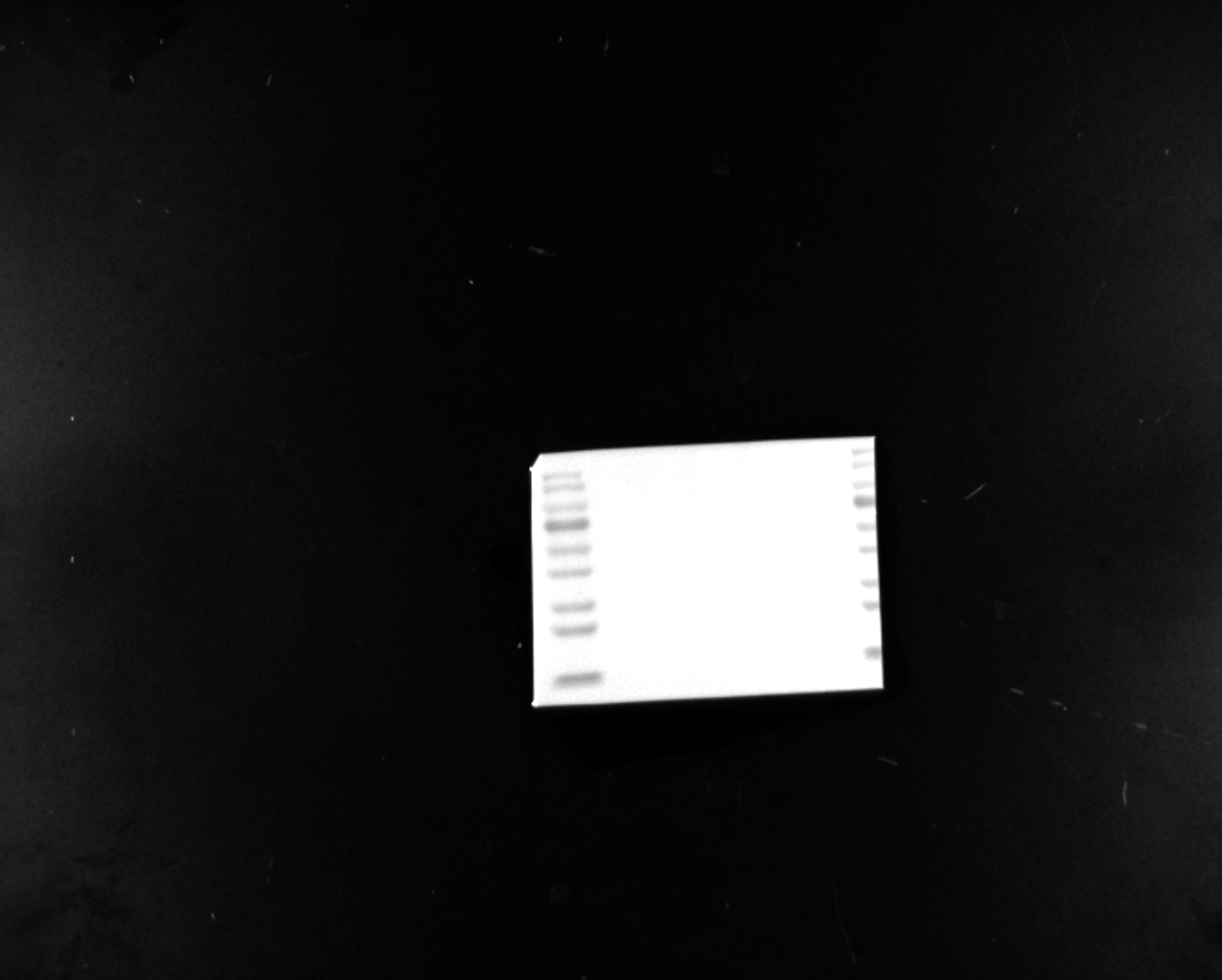

Supplement: Supplementary file 1 [file biomolecules-16-00868-s001.zip › FigureS1 the full, uncropped western blot images/The vitro primary SMCs/SDHA/3-t.Tif]

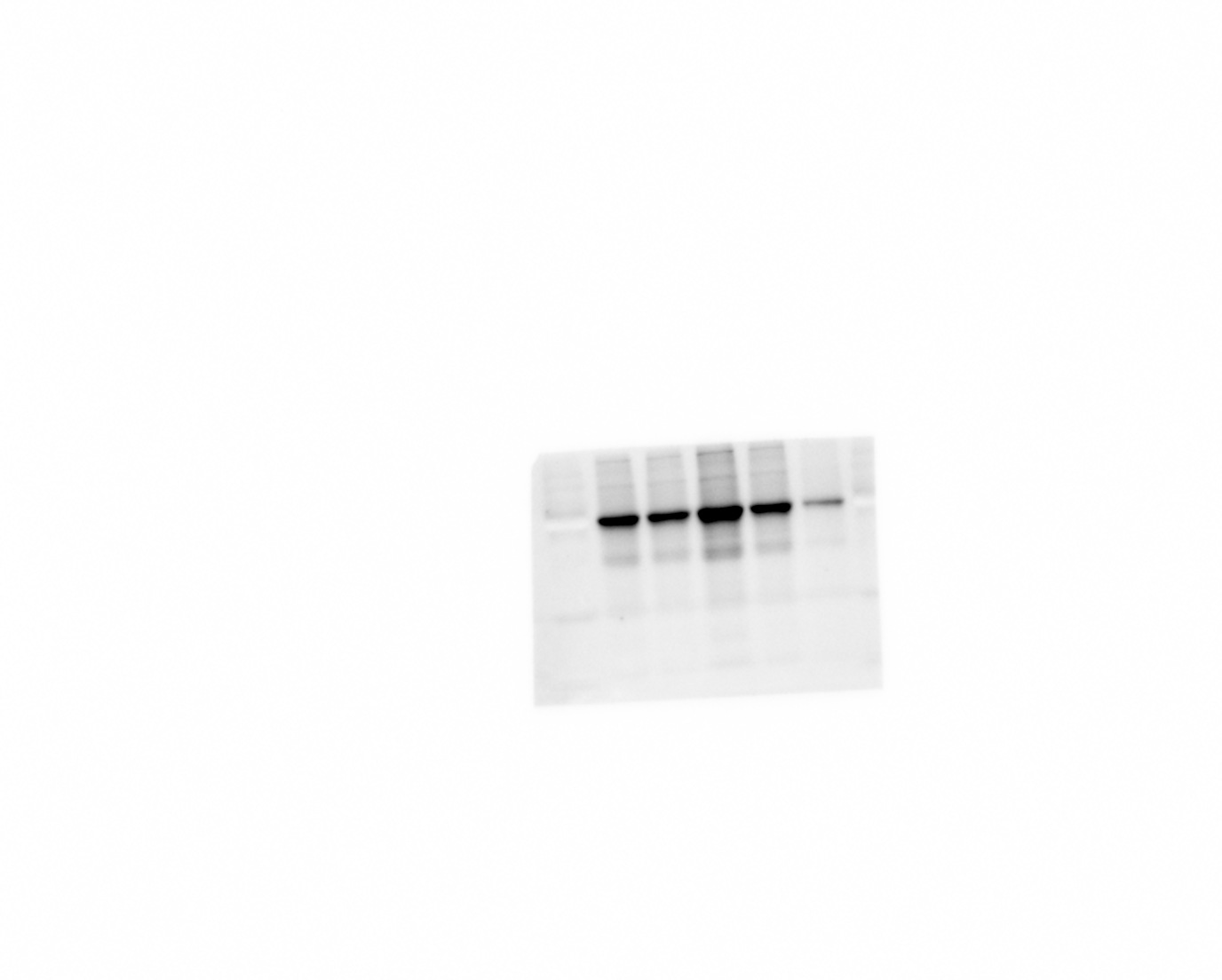

Supplement: Supplementary file 1 [file biomolecules-16-00868-s001.zip › FigureS1 the full, uncropped western blot images/The vitro primary SMCs/SDHA/3.Tif]

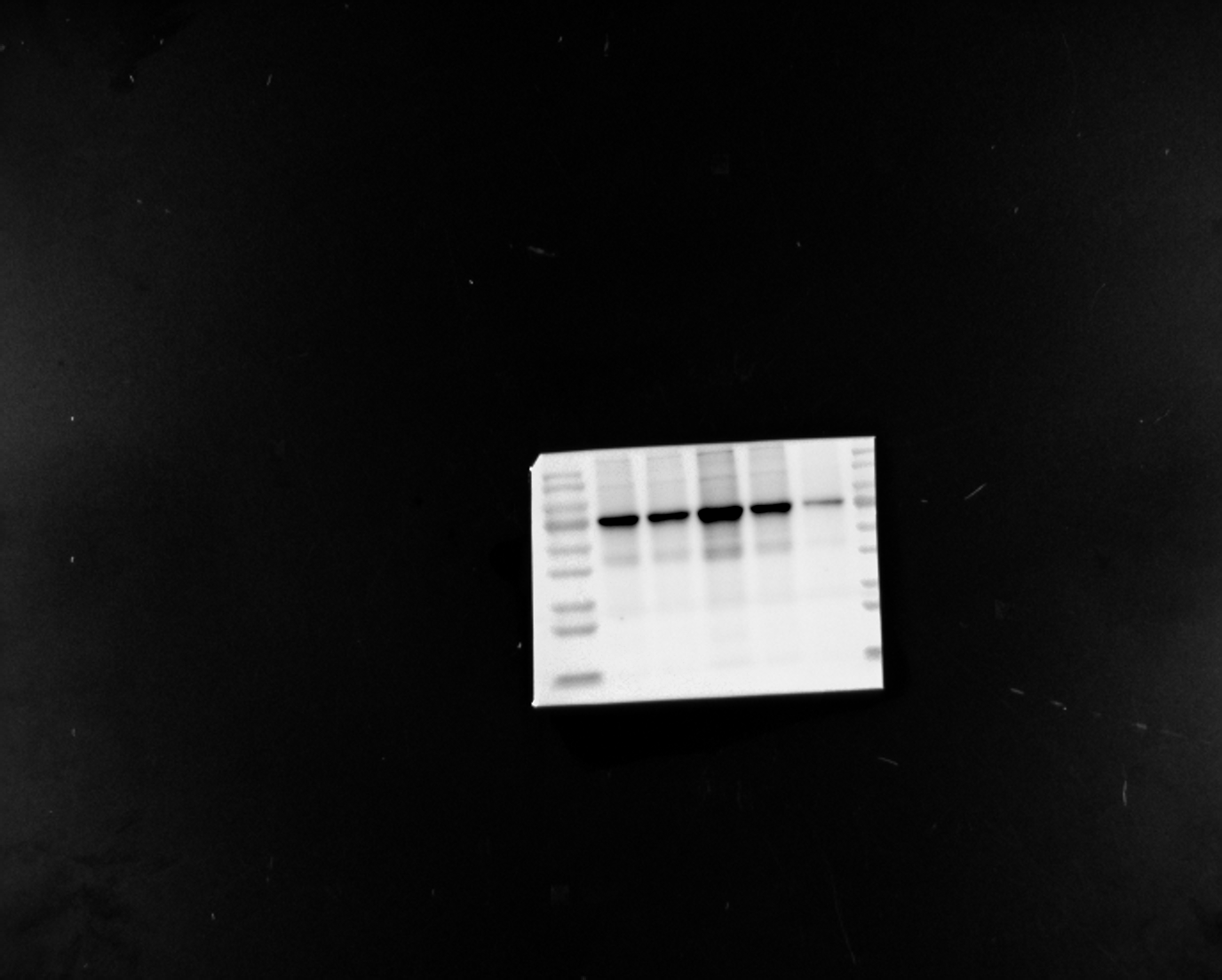

Supplement: Supplementary file 1 [file biomolecules-16-00868-s001.zip › FigureS1 the full, uncropped western blot images/The vitro primary SMCs/SDHA/3副本.Tif]

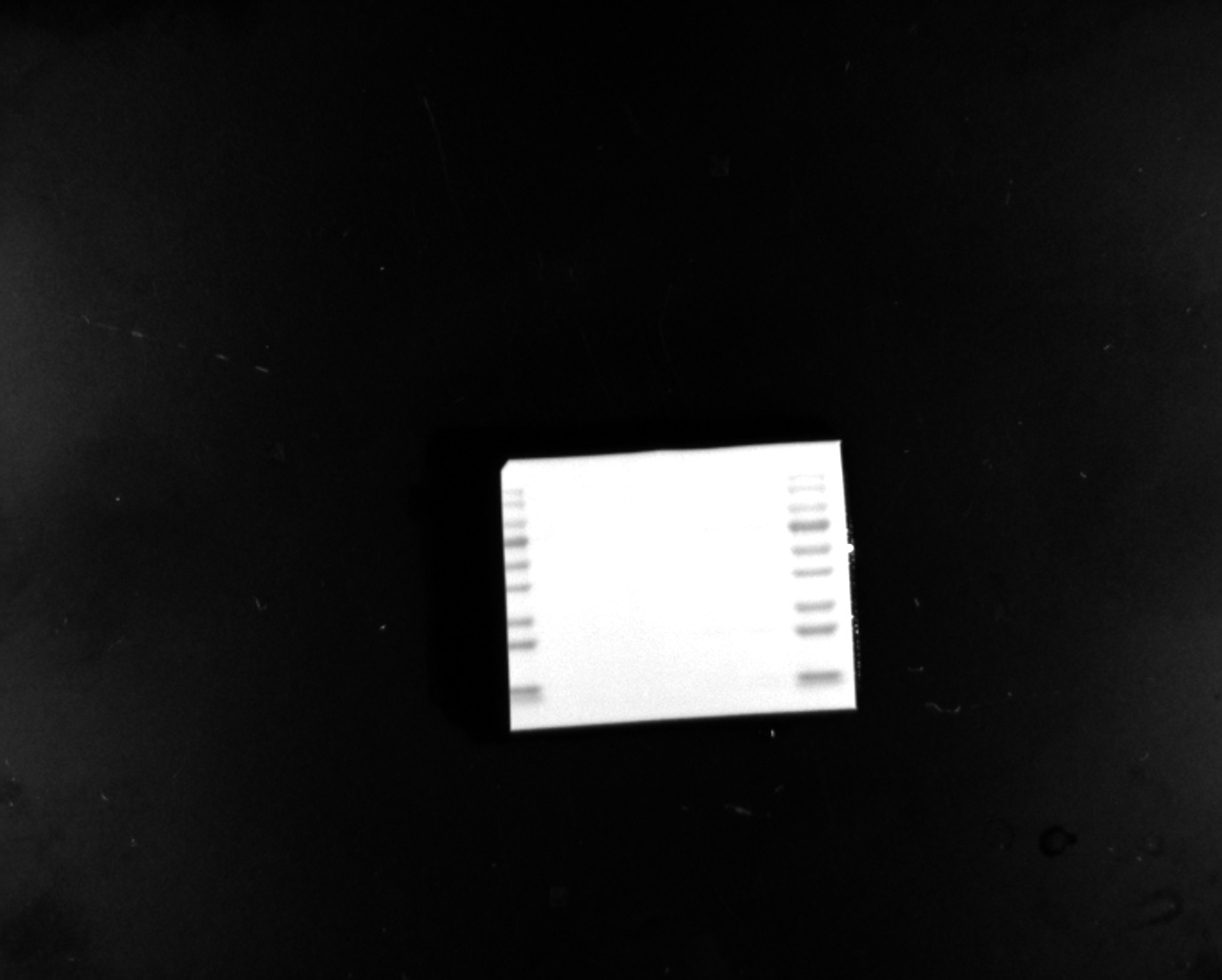

Supplement: Supplementary file 1 [file biomolecules-16-00868-s001.zip › FigureS1 the full, uncropped western blot images/The vitro primary SMCs/UQCRC1/1-t.Tif]

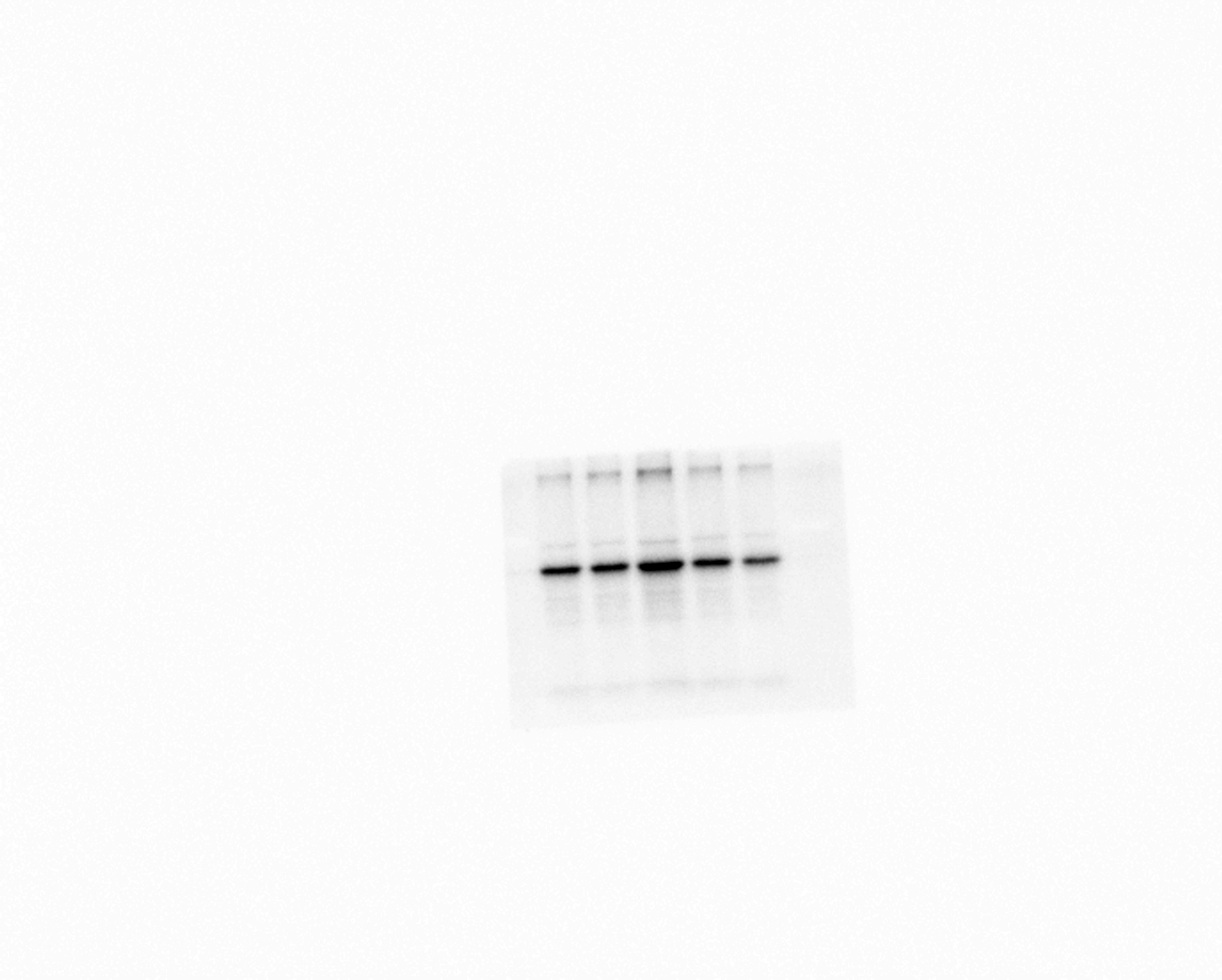

Supplement: Supplementary file 1 [file biomolecules-16-00868-s001.zip › FigureS1 the full, uncropped western blot images/The vitro primary SMCs/UQCRC1/1.Tif]

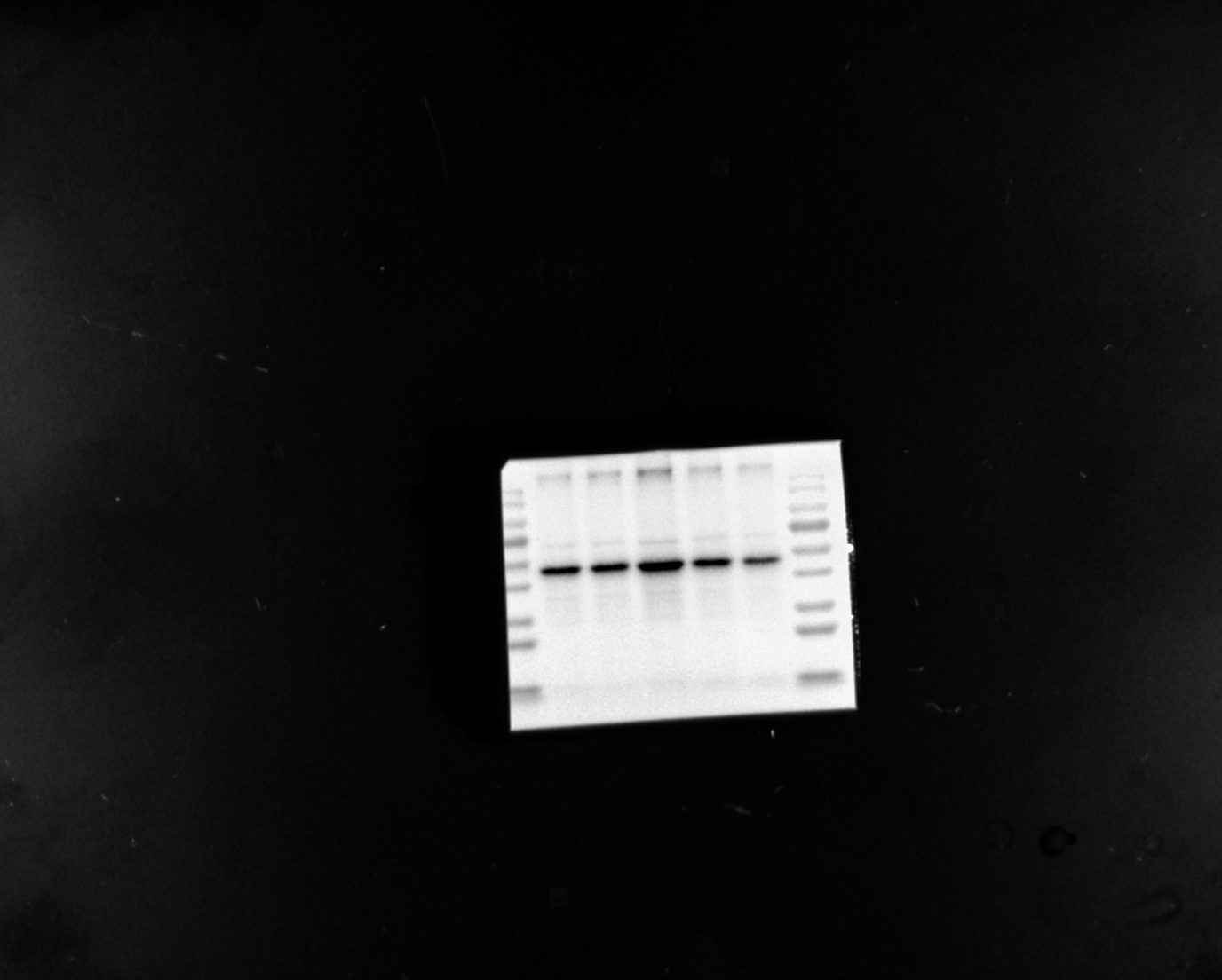

Supplement: Supplementary file 1 [file biomolecules-16-00868-s001.zip › FigureS1 the full, uncropped western blot images/The vitro primary SMCs/UQCRC1/1副本.Tif]

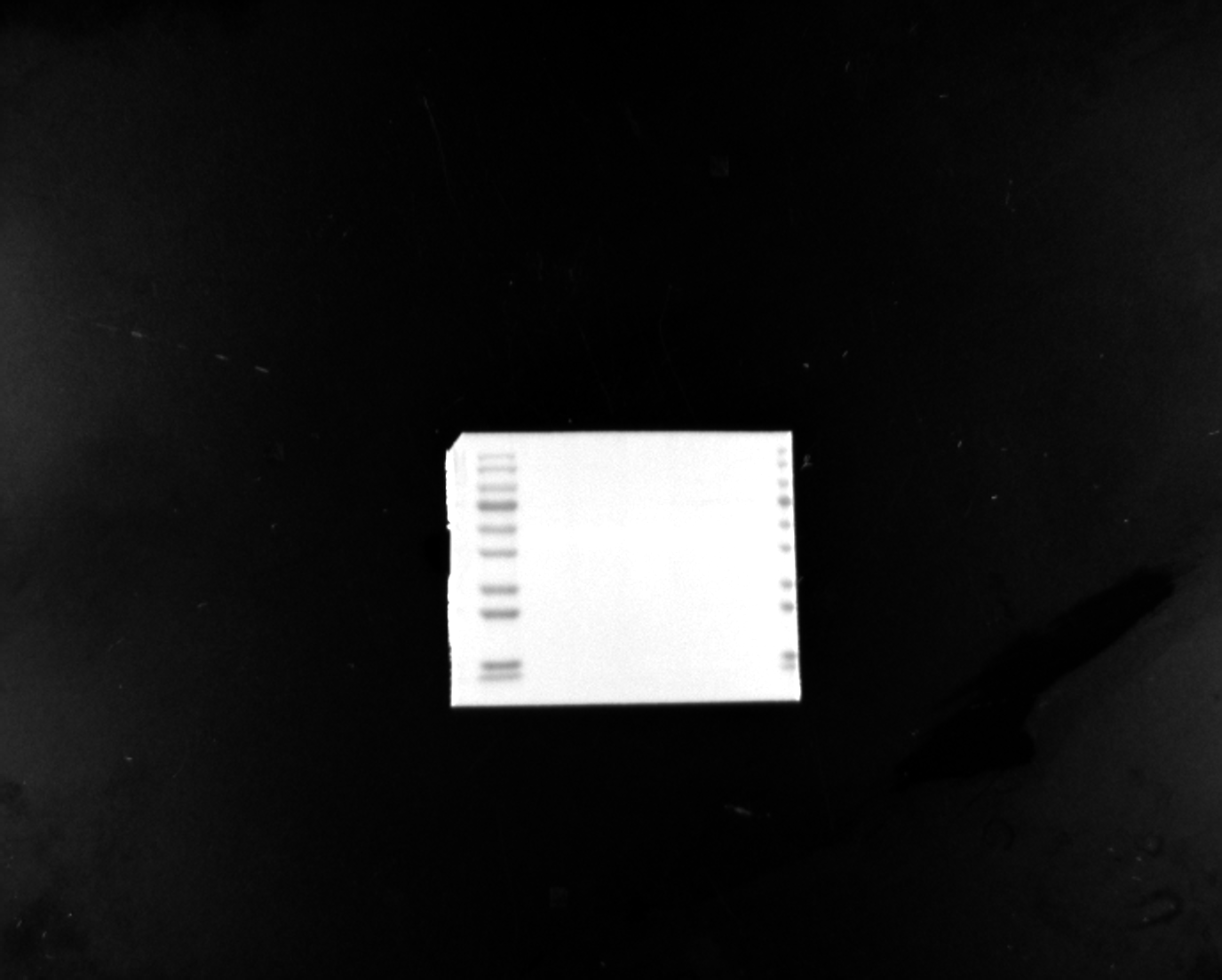

Supplement: Supplementary file 1 [file biomolecules-16-00868-s001.zip › FigureS1 the full, uncropped western blot images/The vitro primary SMCs/UQCRC1/2-t.Tif]

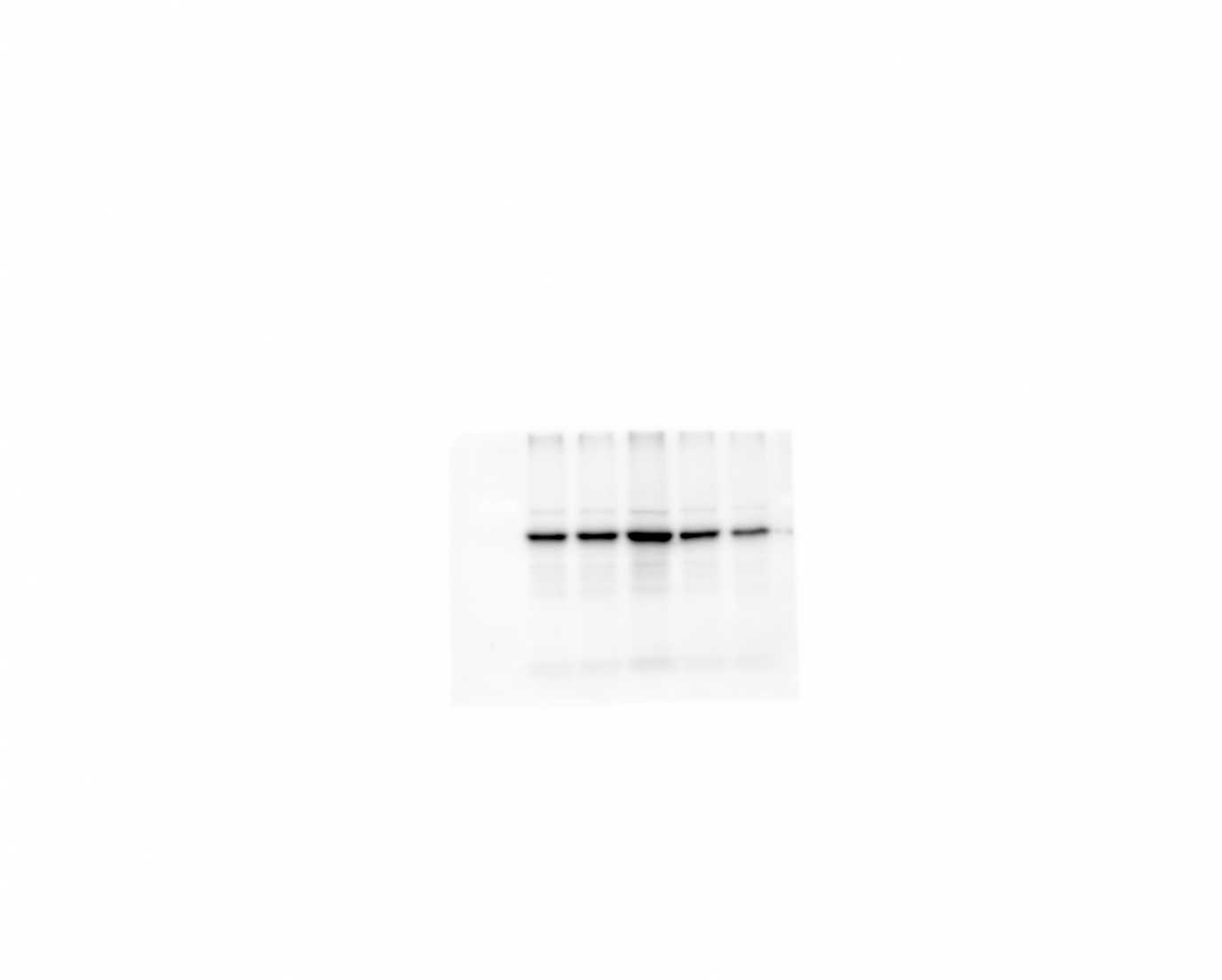

Supplement: Supplementary file 1 [file biomolecules-16-00868-s001.zip › FigureS1 the full, uncropped western blot images/The vitro primary SMCs/UQCRC1/2.Tif]

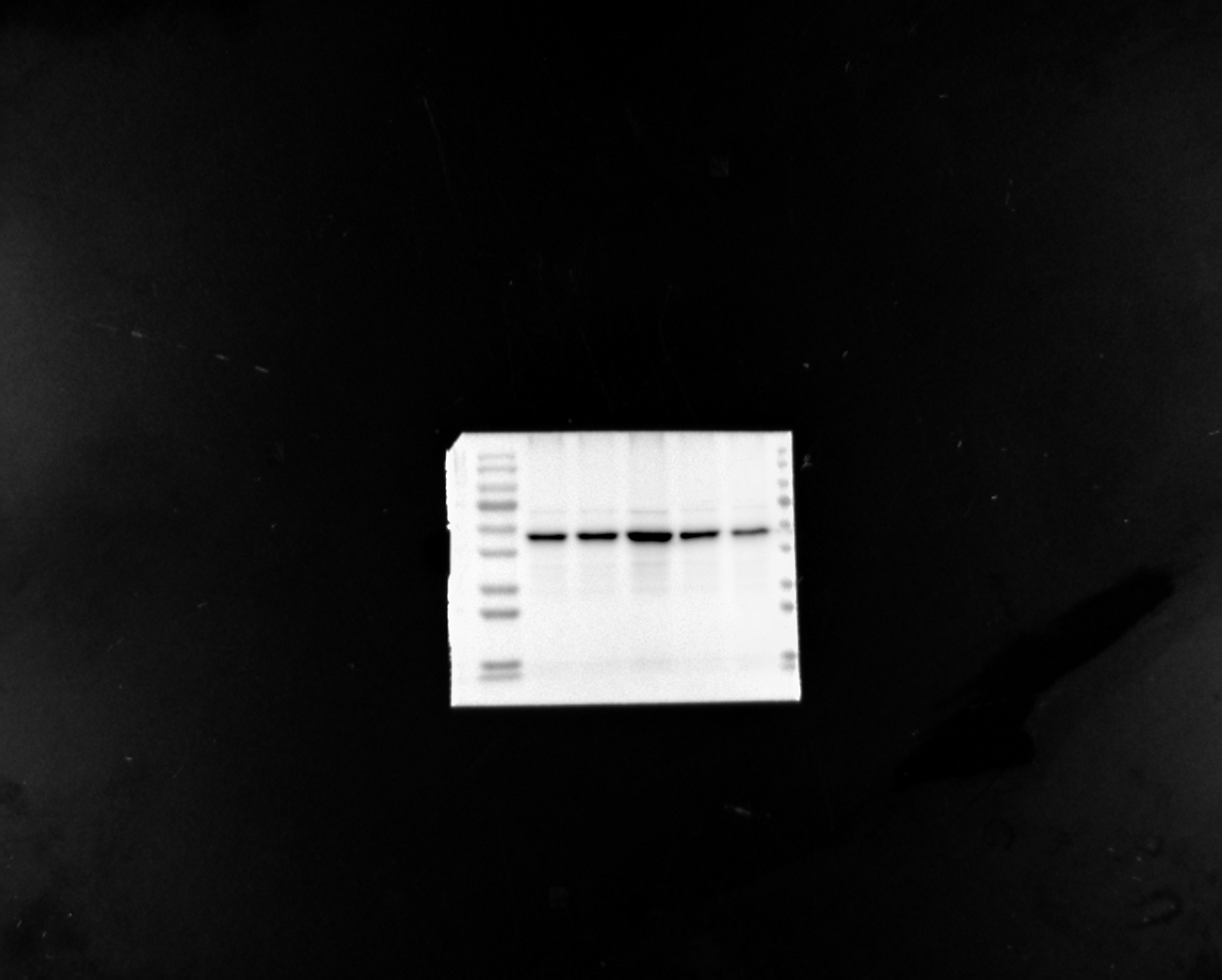

Supplement: Supplementary file 1 [file biomolecules-16-00868-s001.zip › FigureS1 the full, uncropped western blot images/The vitro primary SMCs/UQCRC1/2副本.Tif]

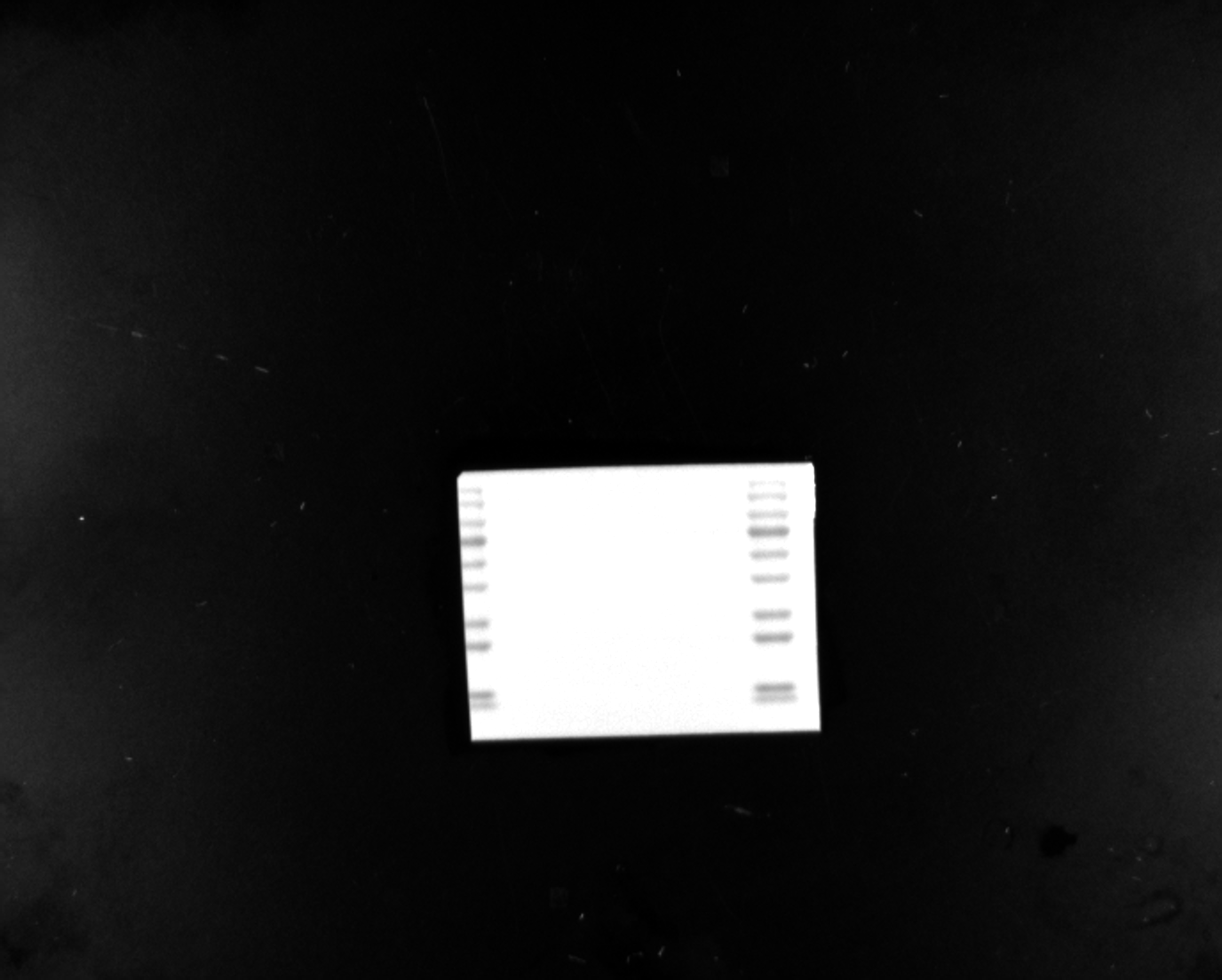

Supplement: Supplementary file 1 [file biomolecules-16-00868-s001.zip › FigureS1 the full, uncropped western blot images/The vitro primary SMCs/UQCRC1/3-t.Tif]

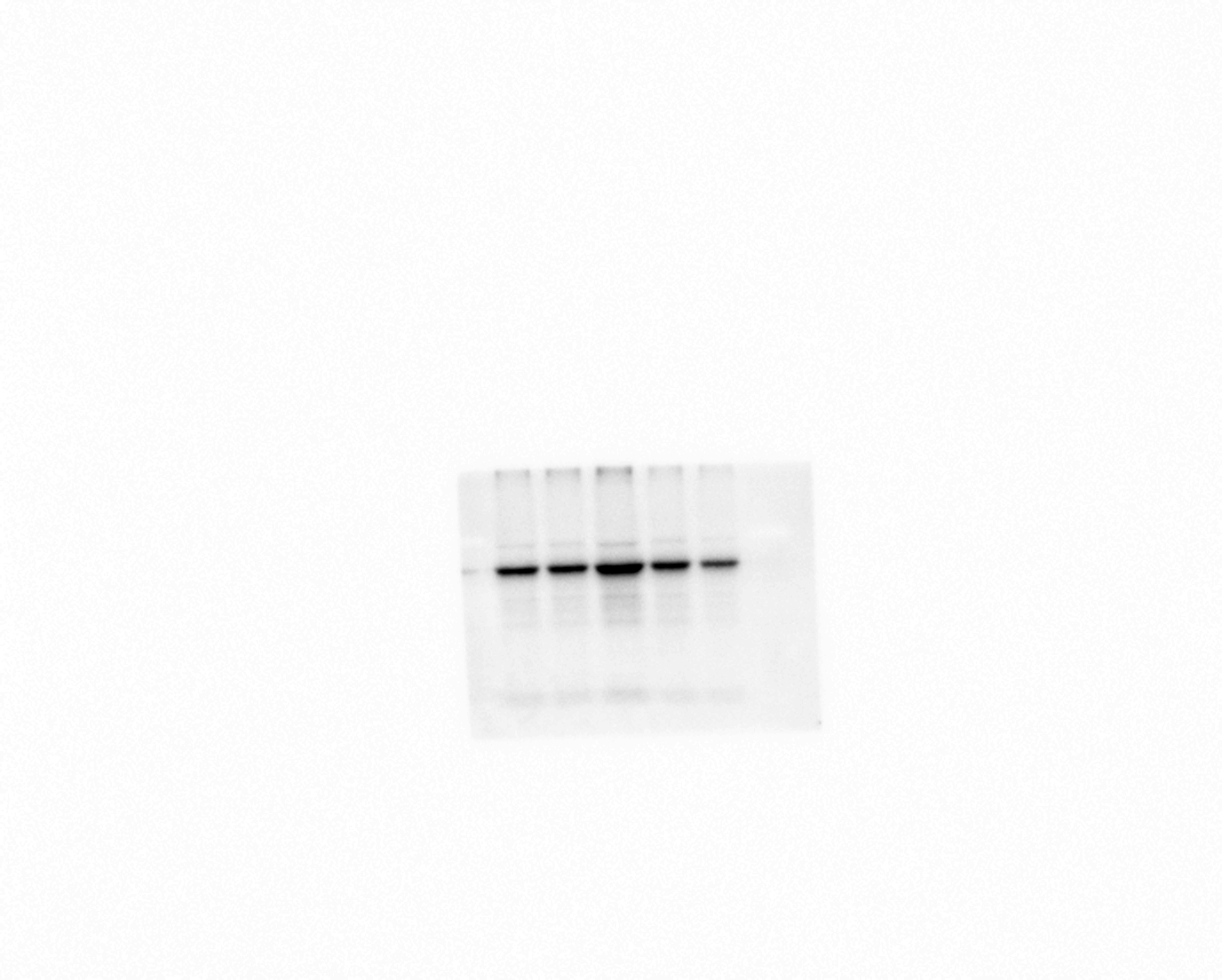

Supplement: Supplementary file 1 [file biomolecules-16-00868-s001.zip › FigureS1 the full, uncropped western blot images/The vitro primary SMCs/UQCRC1/3.Tif]

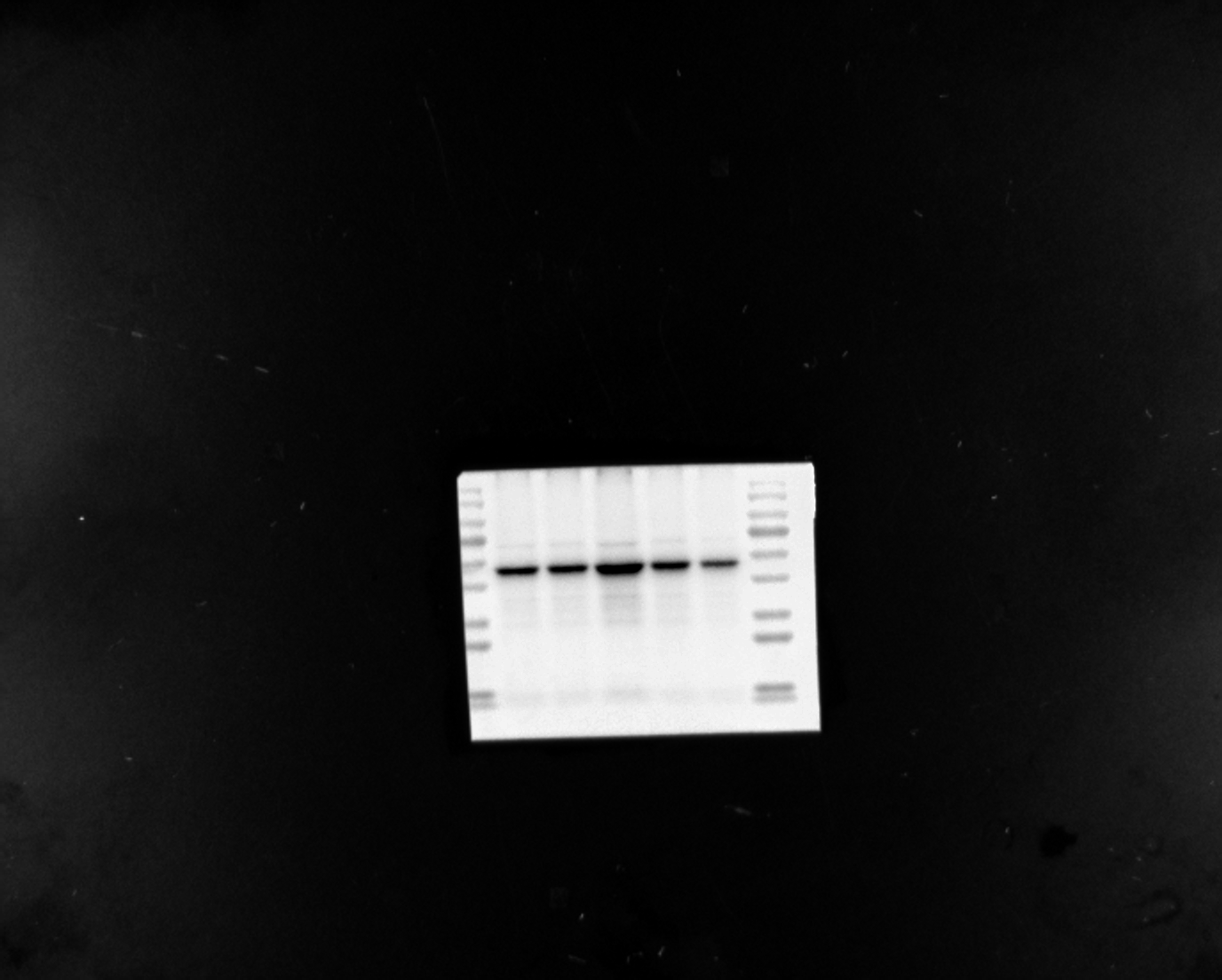

Supplement: Supplementary file 1 [file biomolecules-16-00868-s001.zip › FigureS1 the full, uncropped western blot images/The vitro primary SMCs/UQCRC1/3副本.Tif]

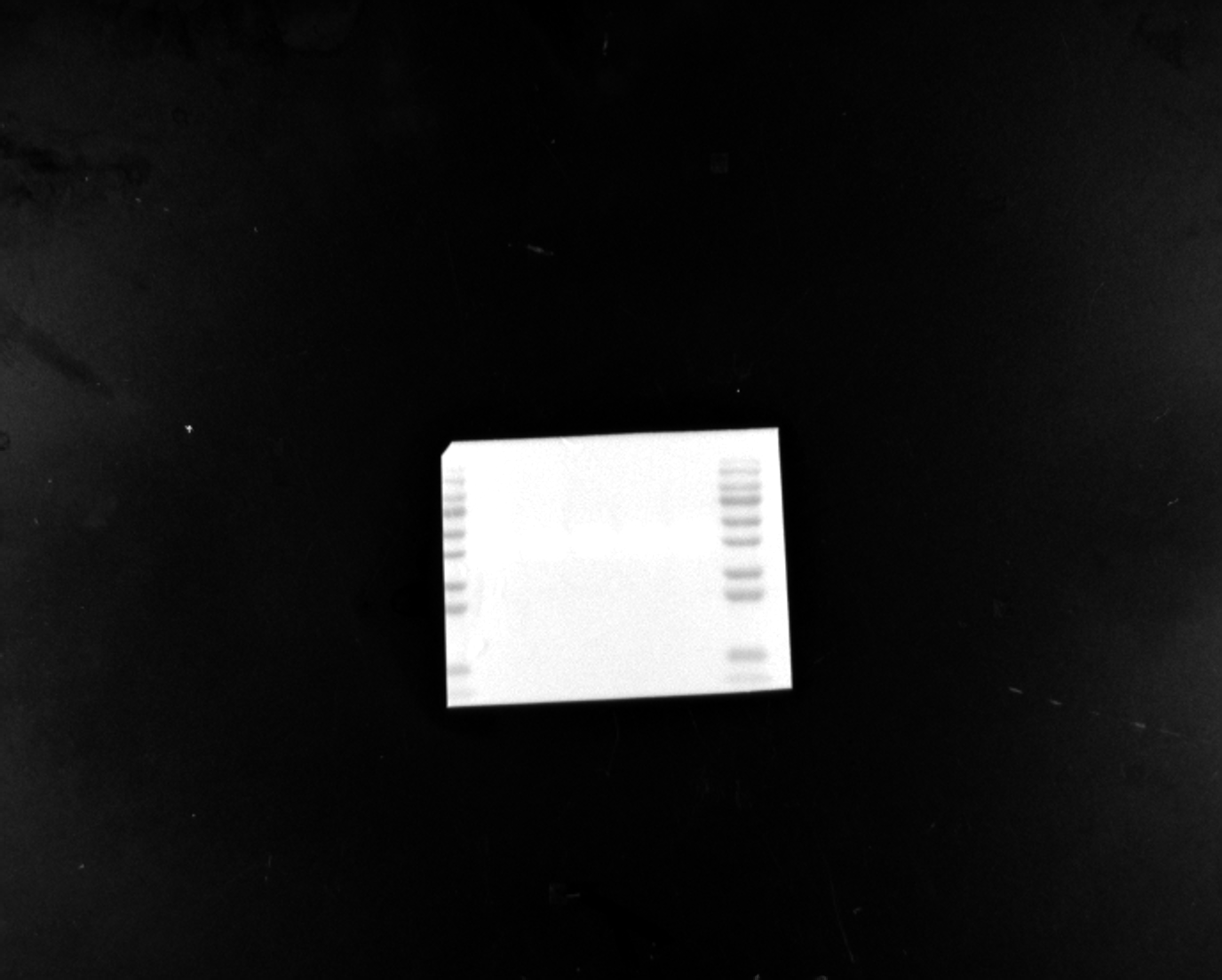

Supplement: Supplementary file 1 [file biomolecules-16-00868-s001.zip › FigureS1 the full, uncropped western blot images/The vitro primary SMCs/β-actin/1-t.Tif]

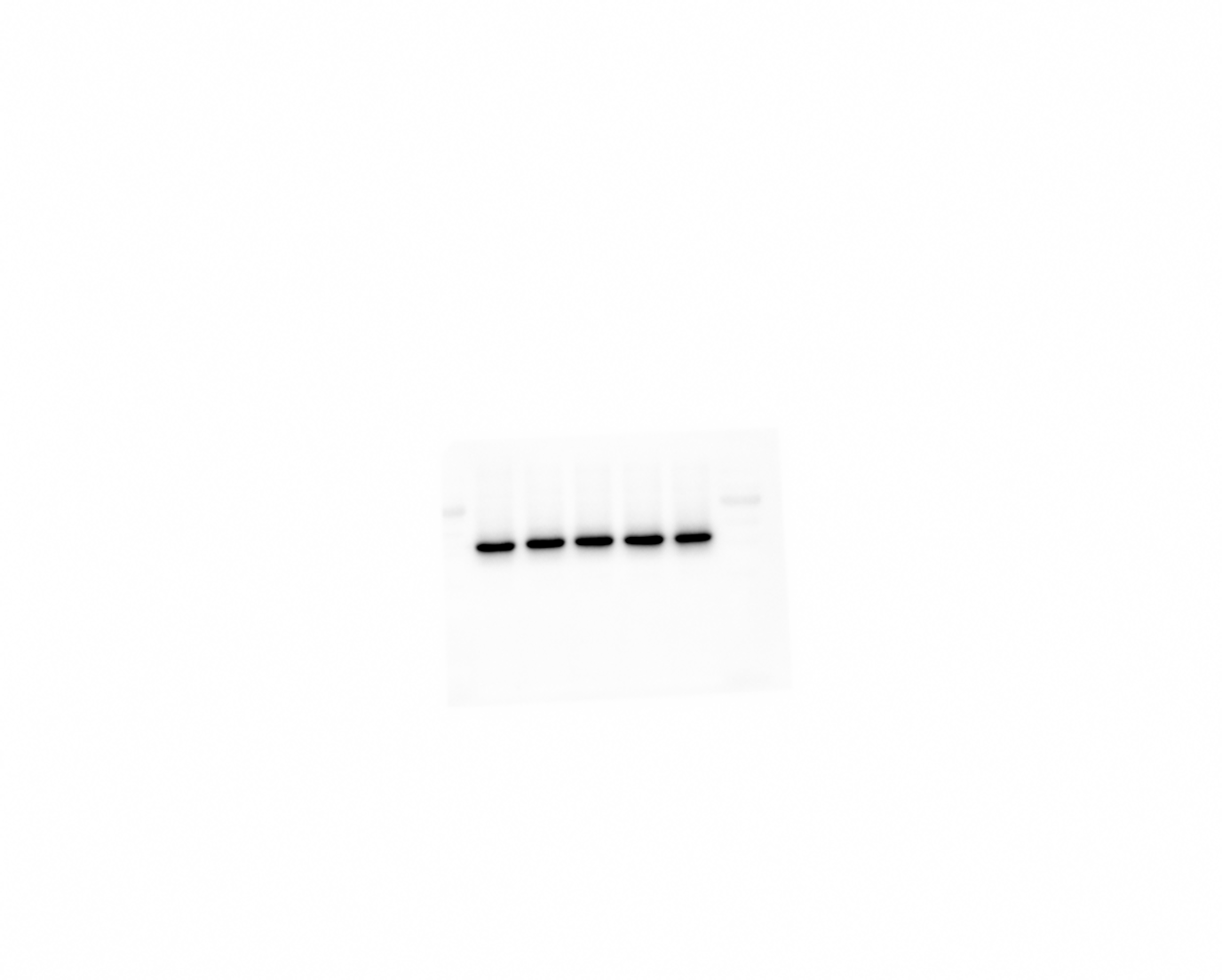

Supplement: Supplementary file 1 [file biomolecules-16-00868-s001.zip › FigureS1 the full, uncropped western blot images/The vitro primary SMCs/β-actin/1.Tif]

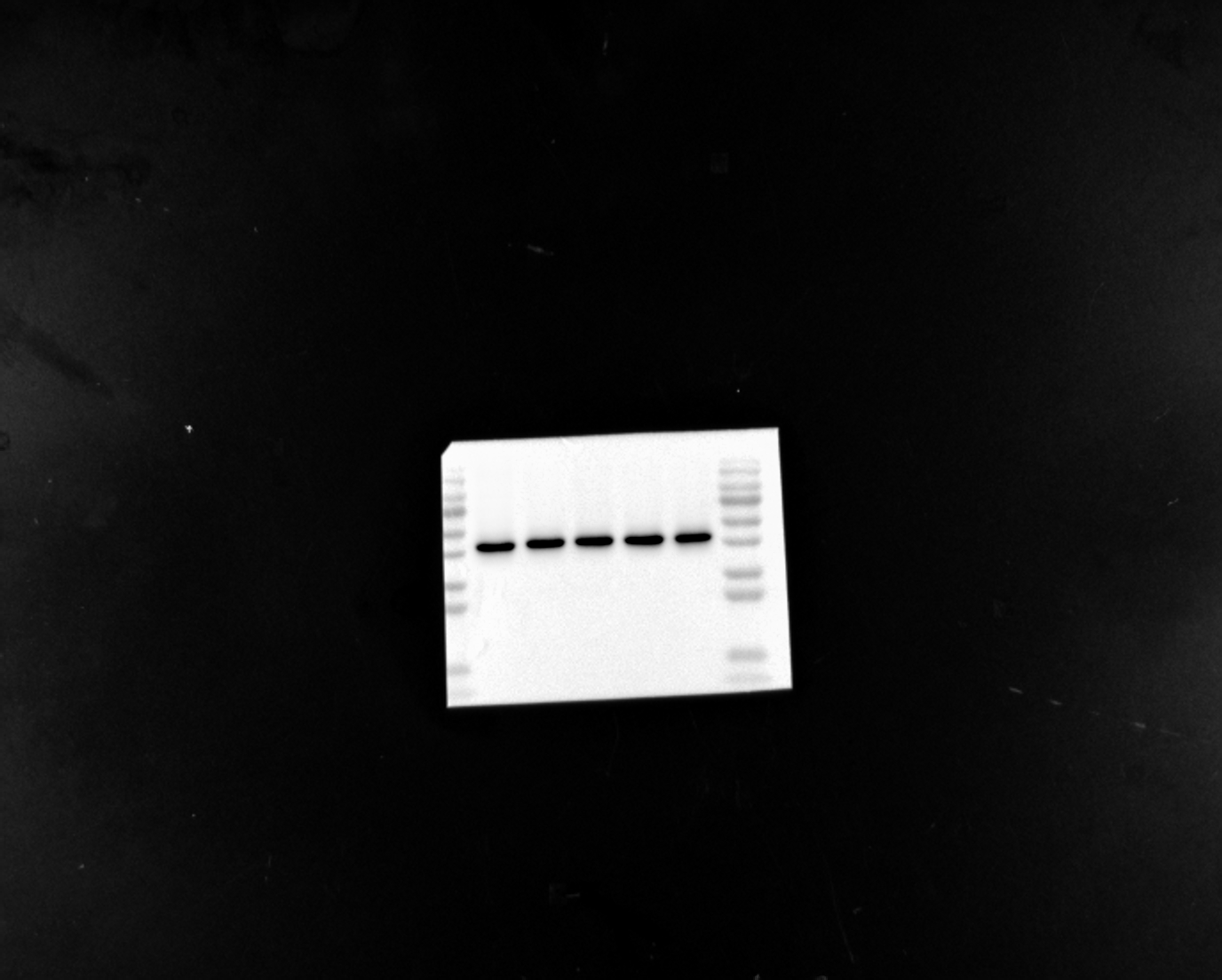

Supplement: Supplementary file 1 [file biomolecules-16-00868-s001.zip › FigureS1 the full, uncropped western blot images/The vitro primary SMCs/β-actin/1副本.Tif]

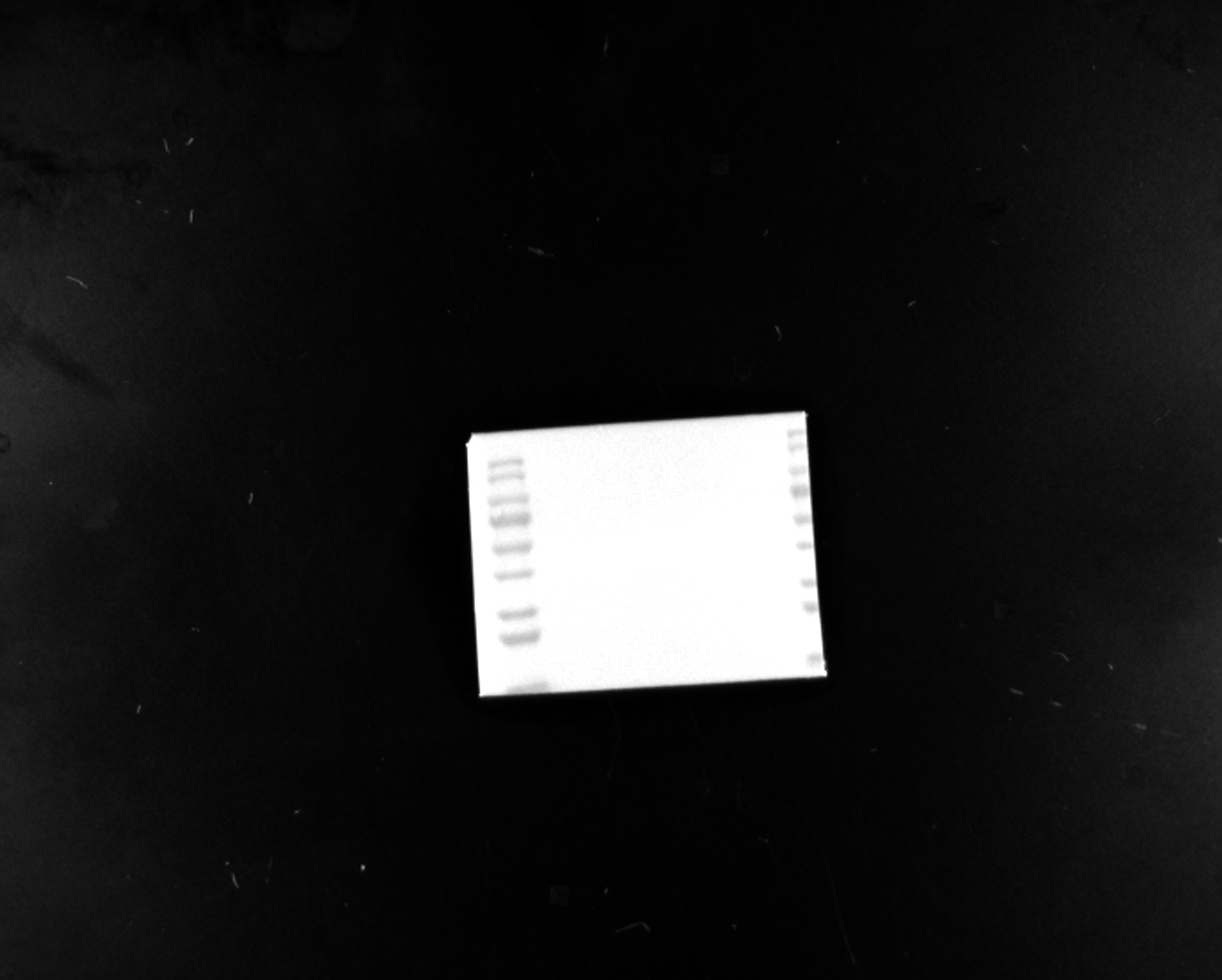

Supplement: Supplementary file 1 [file biomolecules-16-00868-s001.zip › FigureS1 the full, uncropped western blot images/The vitro primary SMCs/β-actin/2-t.Tif]

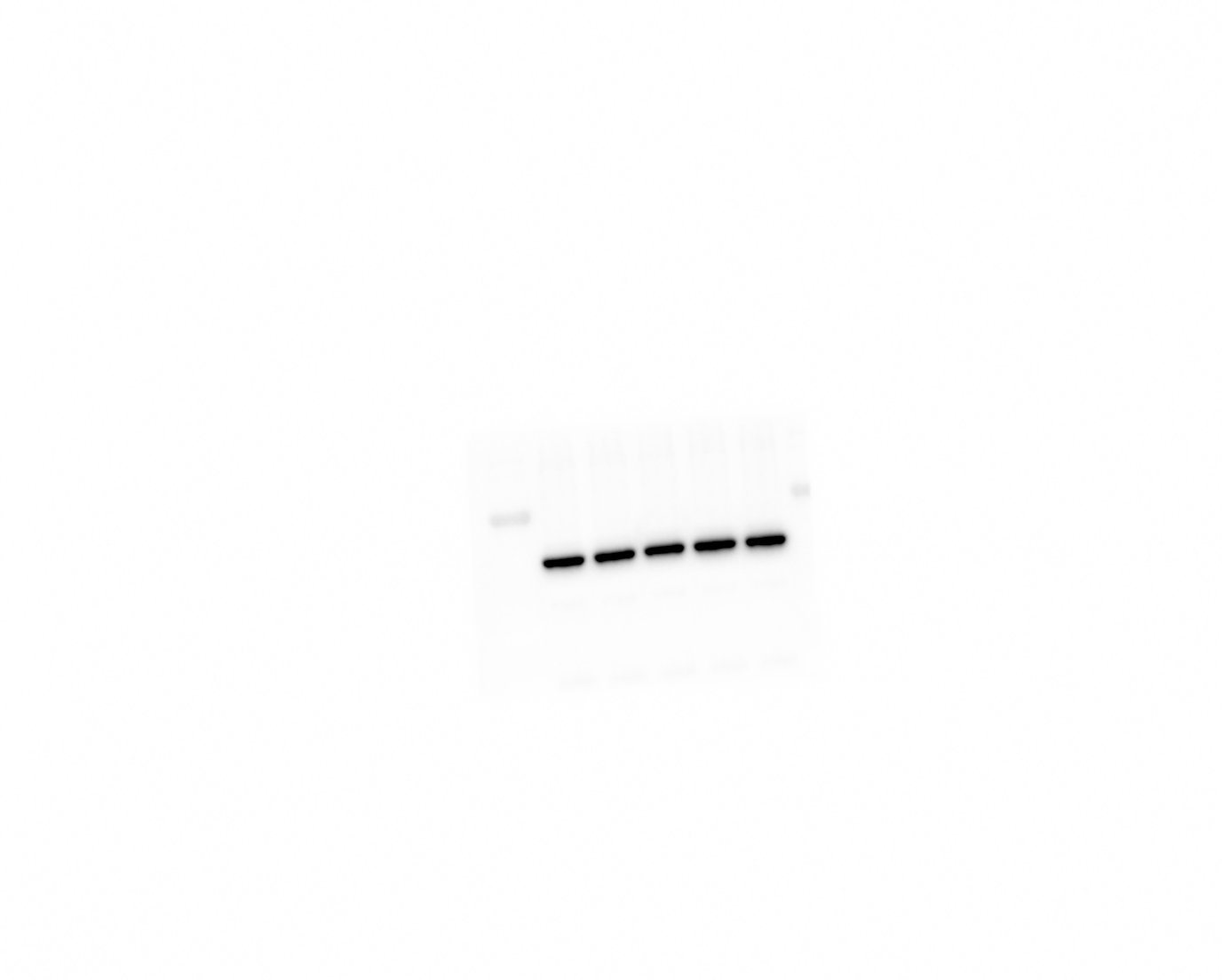

Supplement: Supplementary file 1 [file biomolecules-16-00868-s001.zip › FigureS1 the full, uncropped western blot images/The vitro primary SMCs/β-actin/2.Tif]

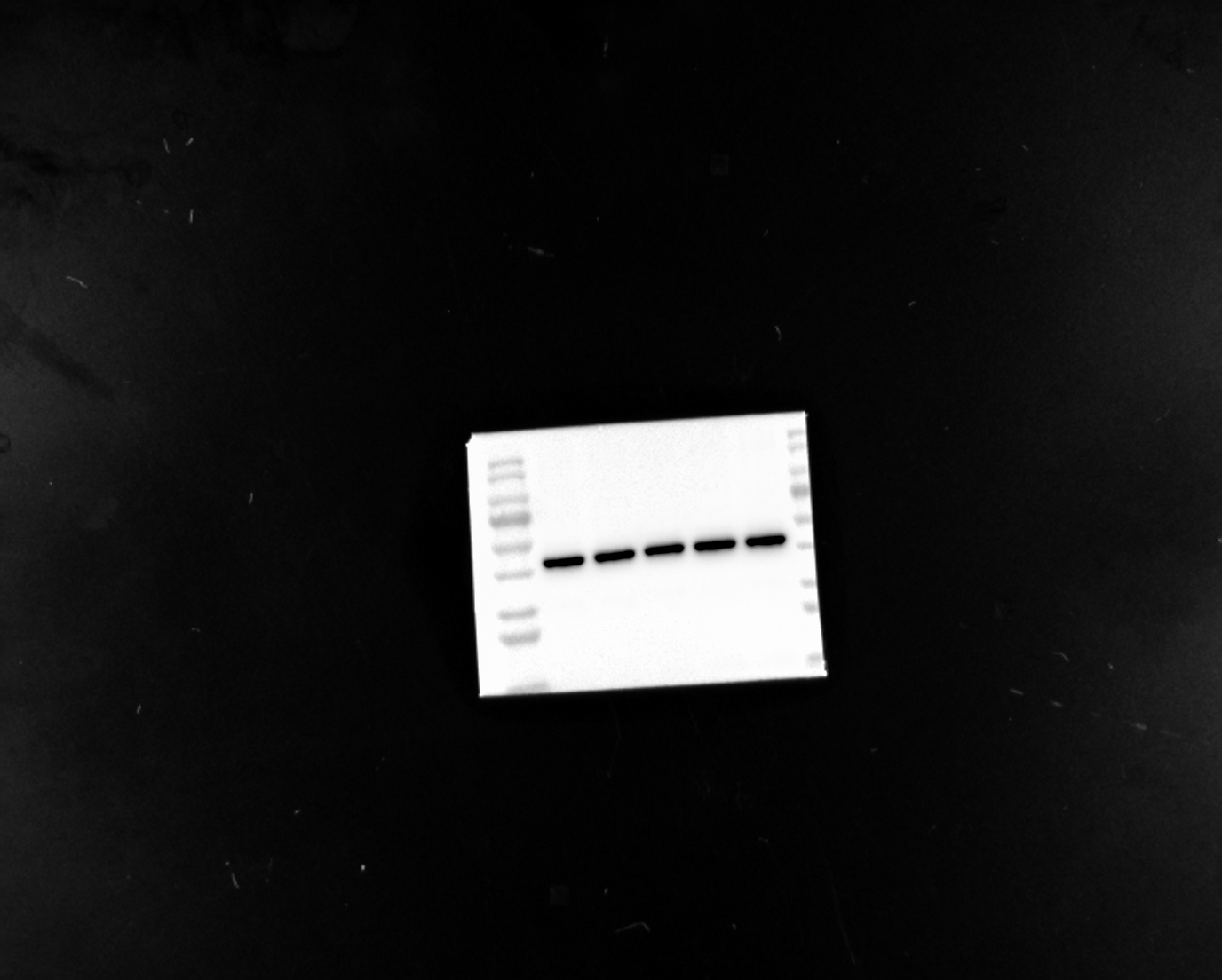

Supplement: Supplementary file 1 [file biomolecules-16-00868-s001.zip › FigureS1 the full, uncropped western blot images/The vitro primary SMCs/β-actin/2副本.Tif]

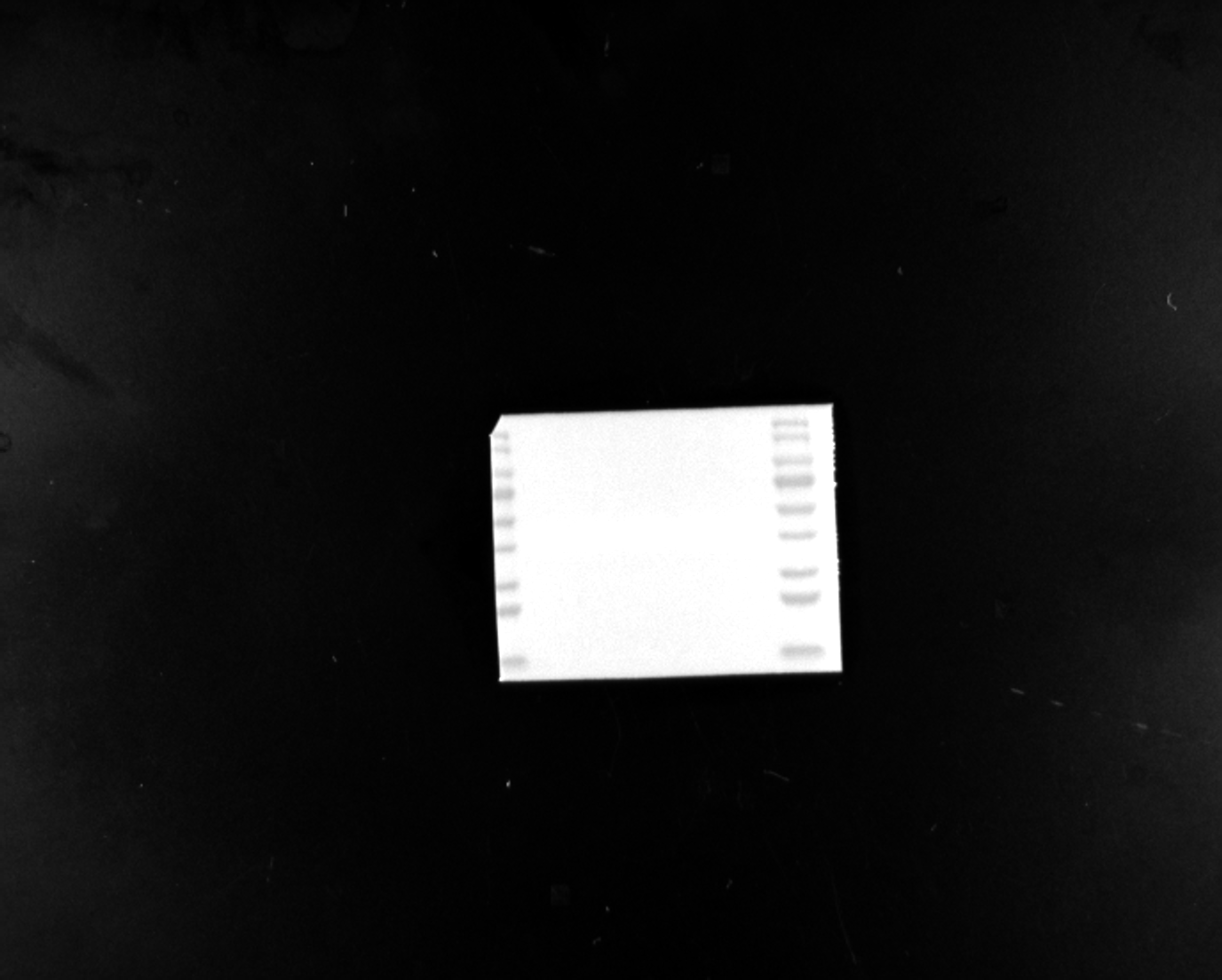

Supplement: Supplementary file 1 [file biomolecules-16-00868-s001.zip › FigureS1 the full, uncropped western blot images/The vitro primary SMCs/β-actin/3-t.Tif]

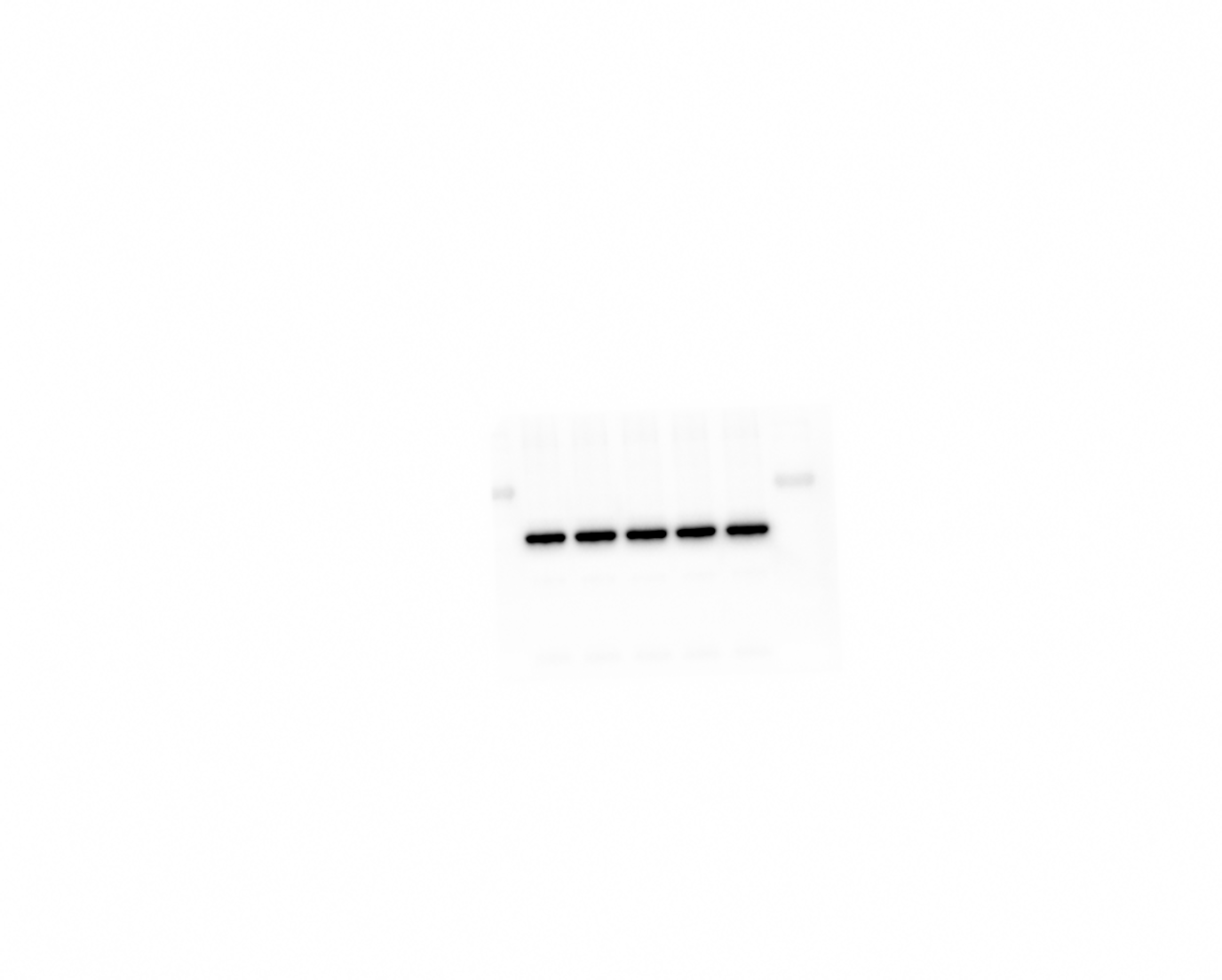

Supplement: Supplementary file 1 [file biomolecules-16-00868-s001.zip › FigureS1 the full, uncropped western blot images/The vitro primary SMCs/β-actin/3.Tif]

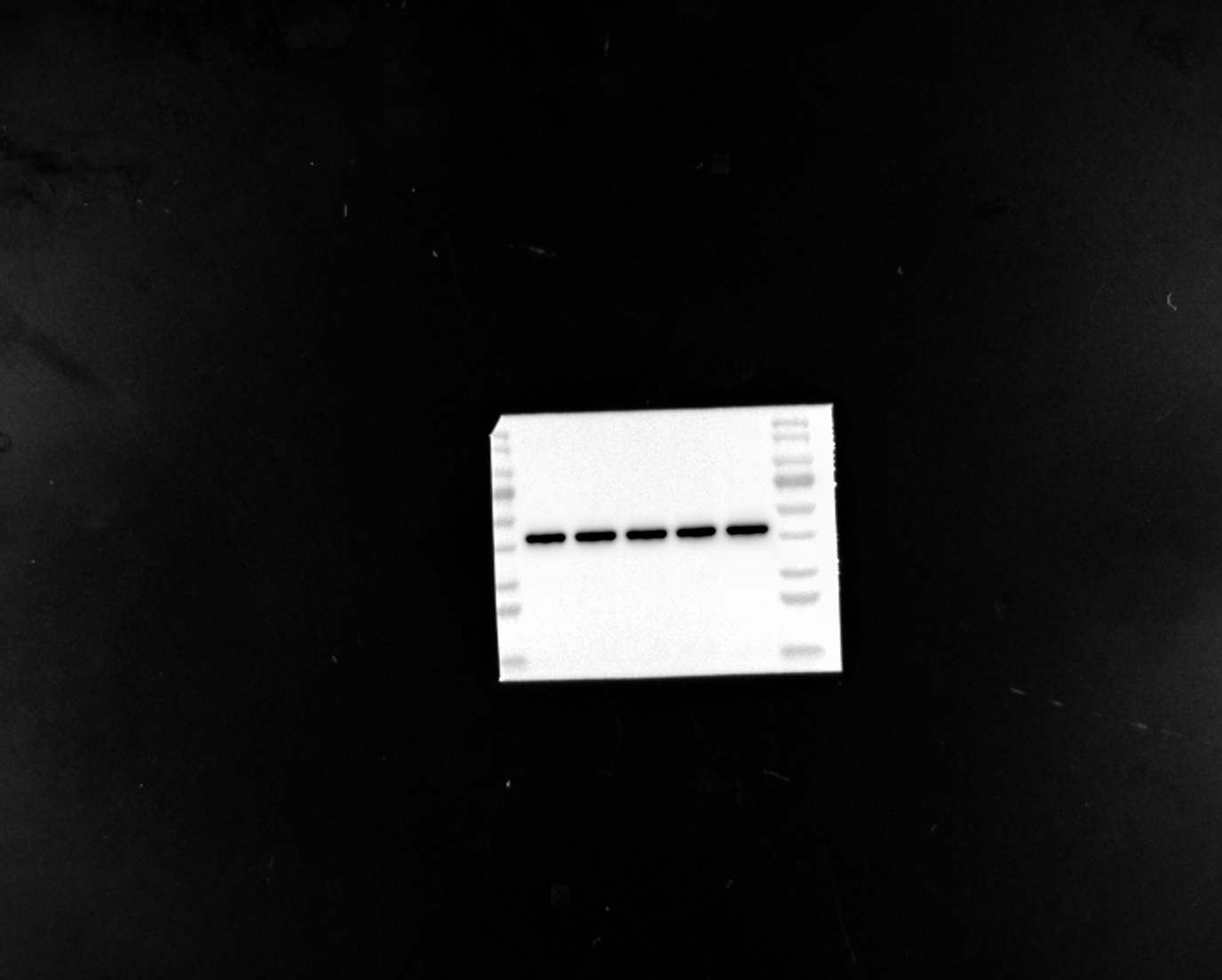

Supplement: Supplementary file 1 [file biomolecules-16-00868-s001.zip › FigureS1 the full, uncropped western blot images/The vitro primary SMCs/β-actin/3副本.Tif]

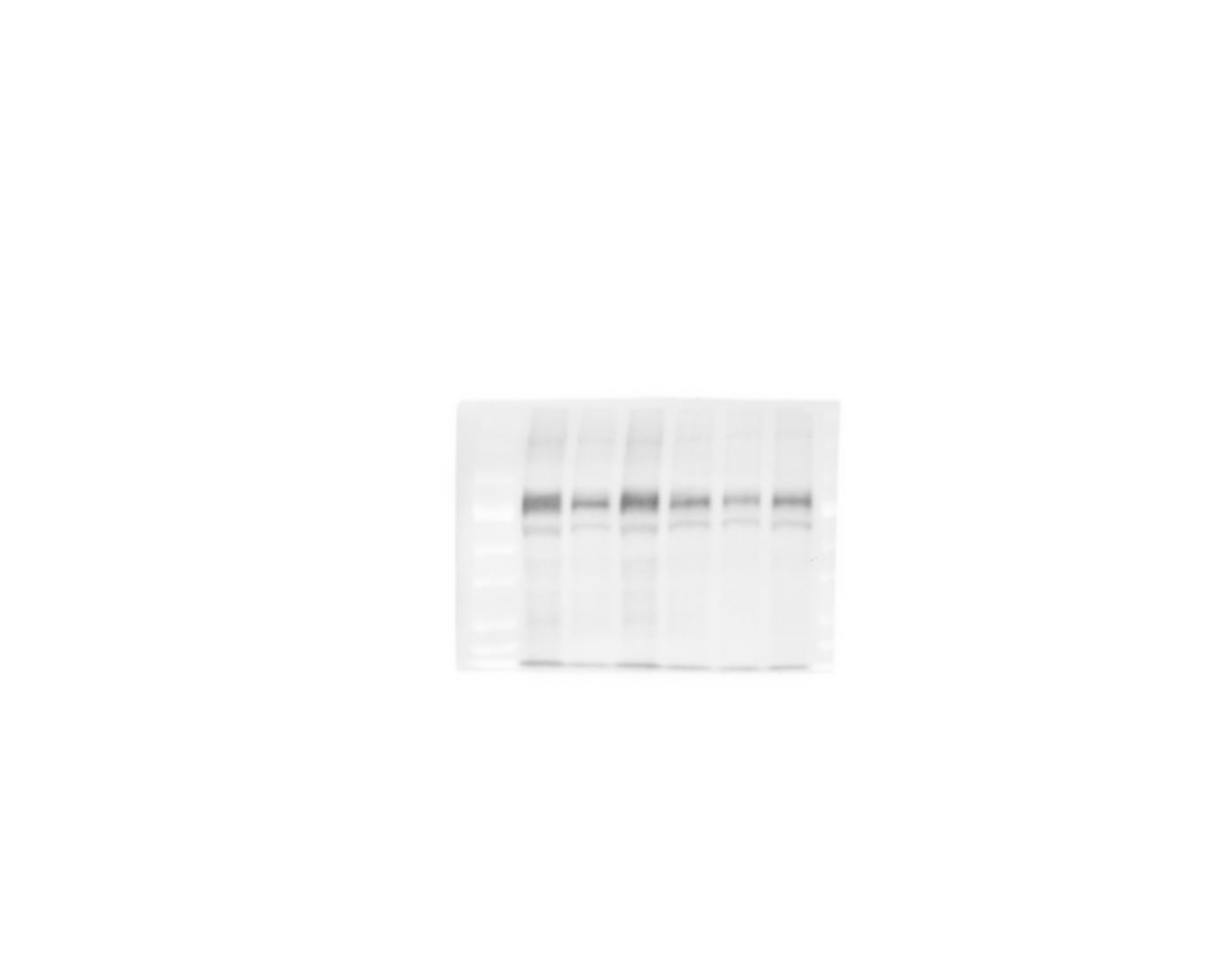

Supplement: Supplementary file 1 [file biomolecules-16-00868-s001.zip › FigureS1 the full, uncropped western blot images/The vivo mice study/HSF1/1-0.6s.Tif]

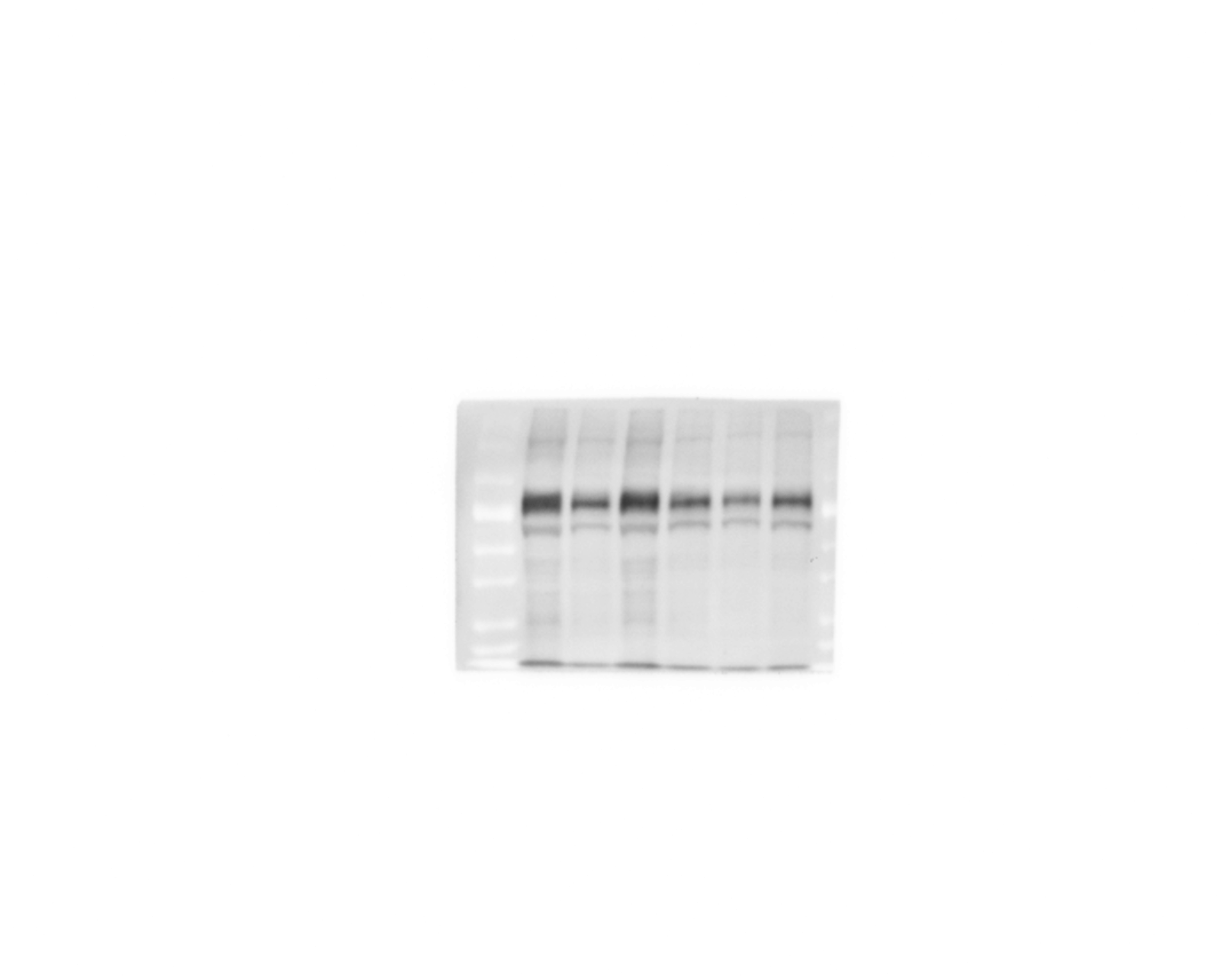

Supplement: Supplementary file 1 [file biomolecules-16-00868-s001.zip › FigureS1 the full, uncropped western blot images/The vivo mice study/HSF1/1-2s.Tif]

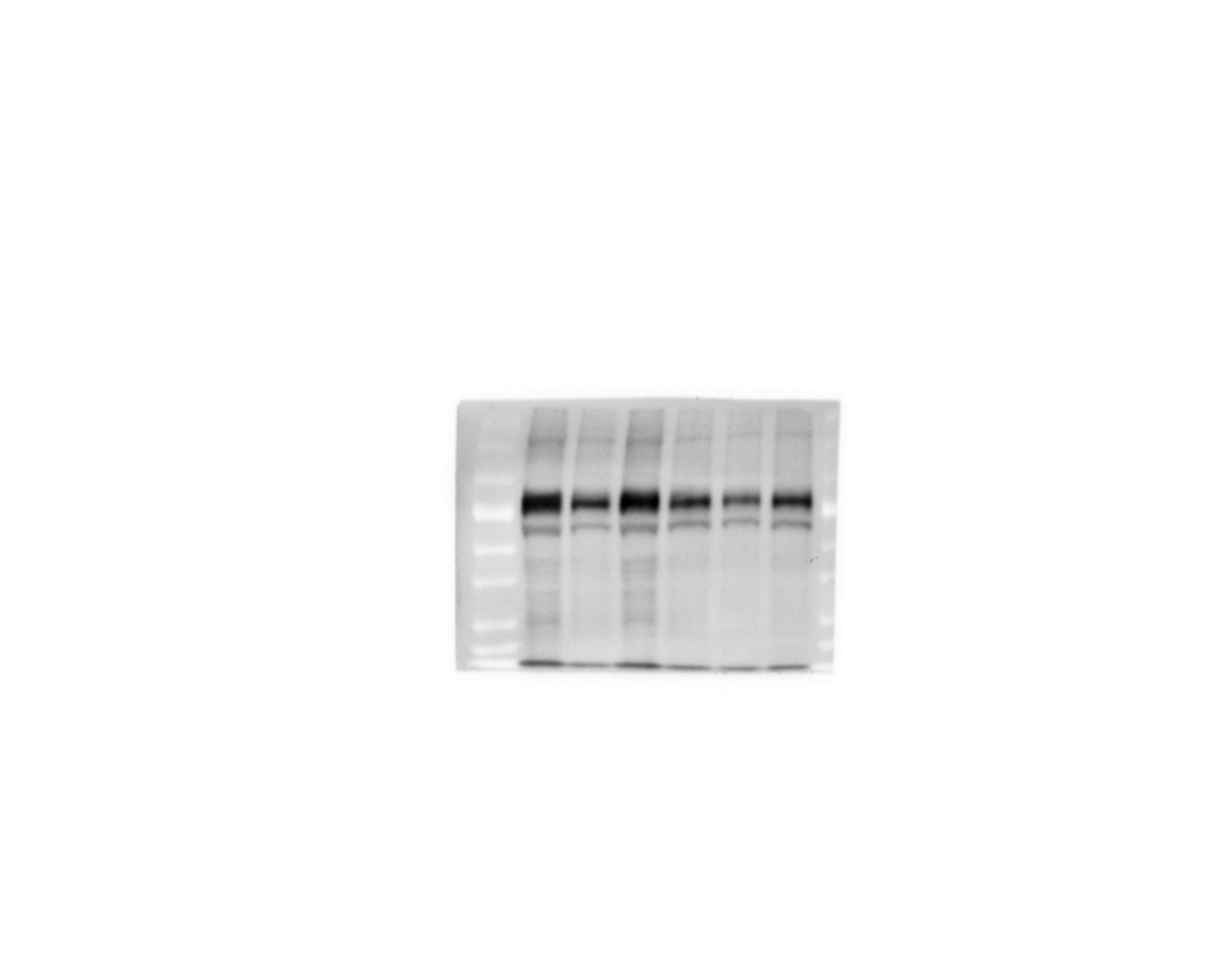

Supplement: Supplementary file 1 [file biomolecules-16-00868-s001.zip › FigureS1 the full, uncropped western blot images/The vivo mice study/HSF1/1-5s.Tif]

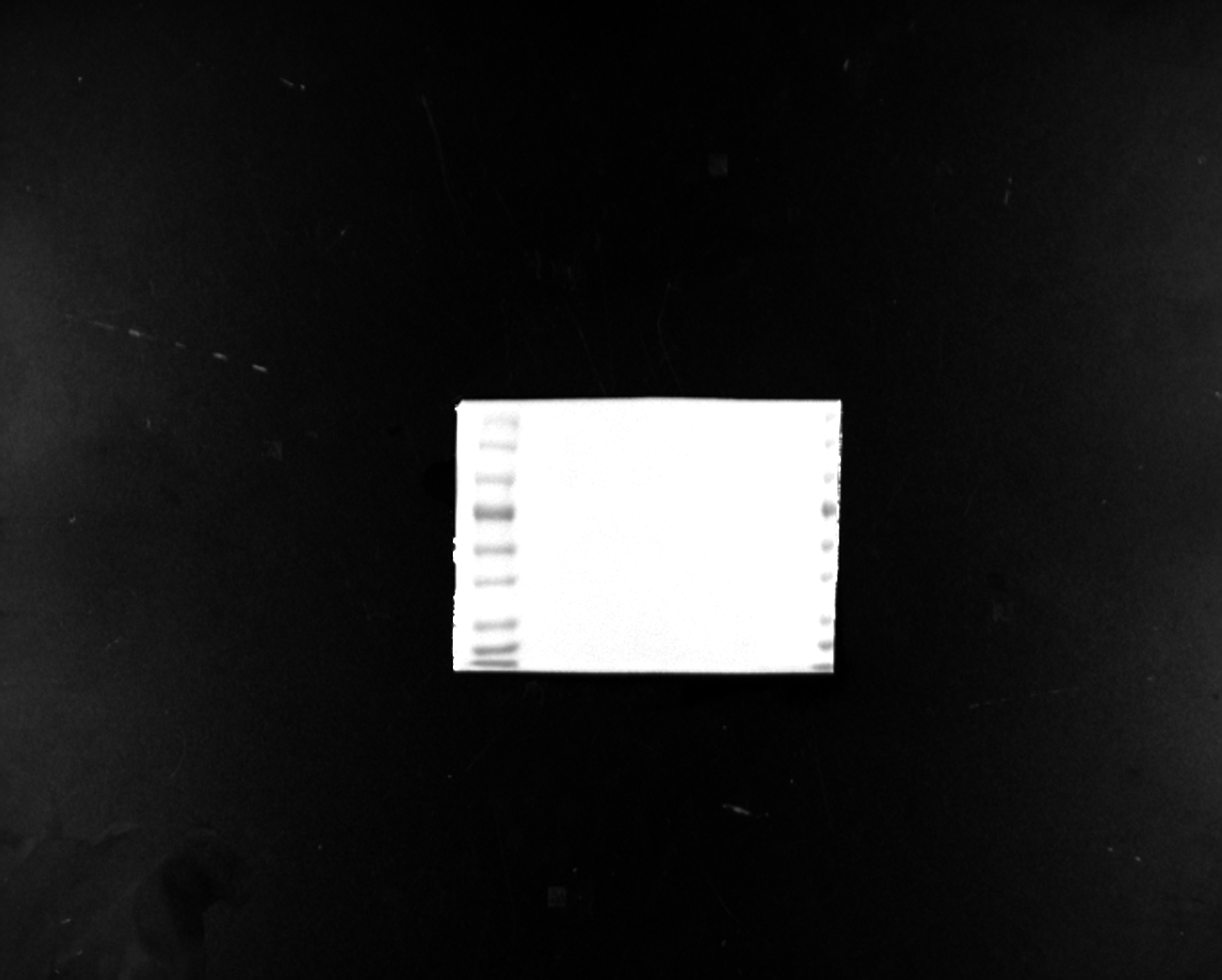

Supplement: Supplementary file 1 [file biomolecules-16-00868-s001.zip › FigureS1 the full, uncropped western blot images/The vivo mice study/HSF1/1-t.Tif]

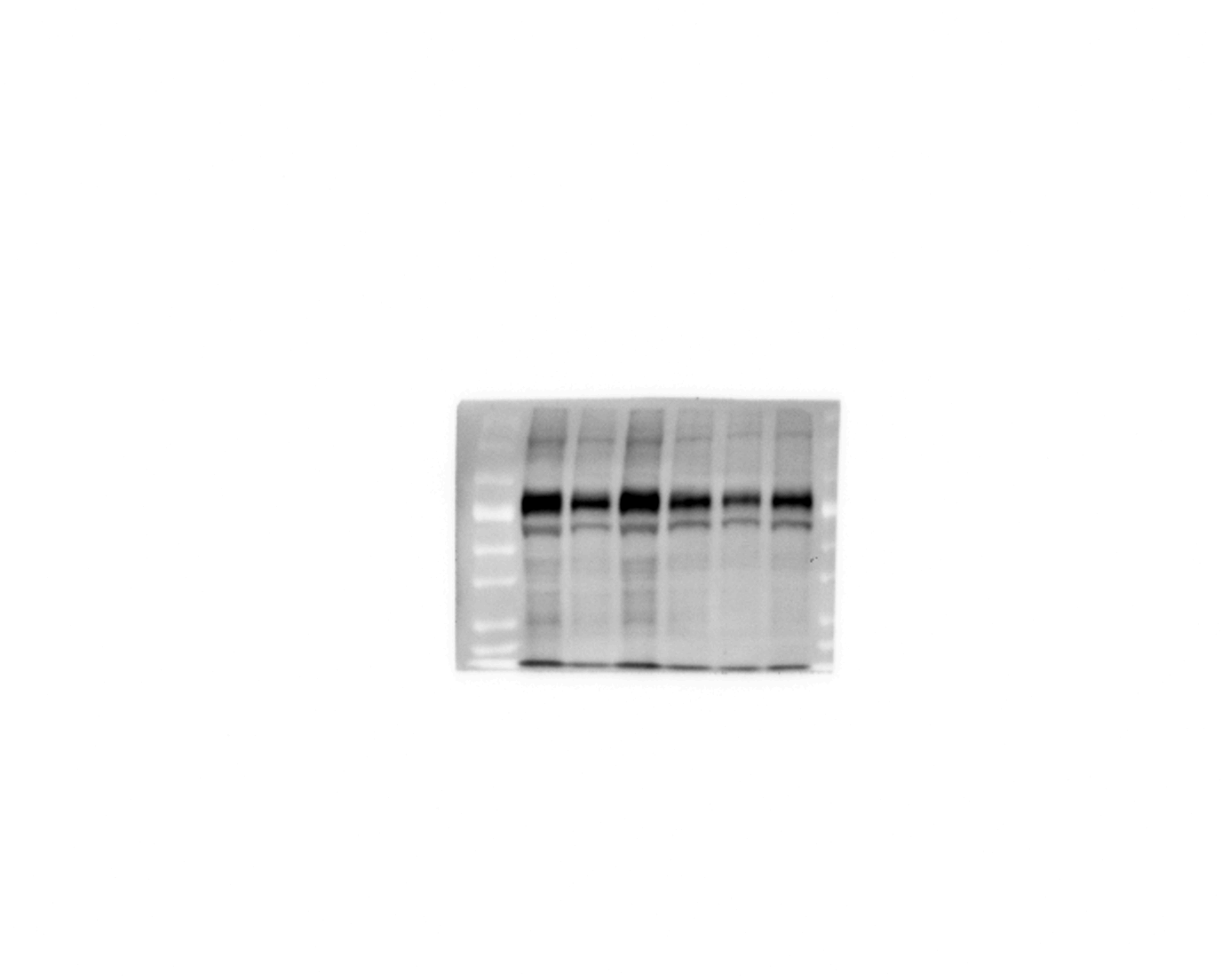

Supplement: Supplementary file 1 [file biomolecules-16-00868-s001.zip › FigureS1 the full, uncropped western blot images/The vivo mice study/HSF1/1.Tif]

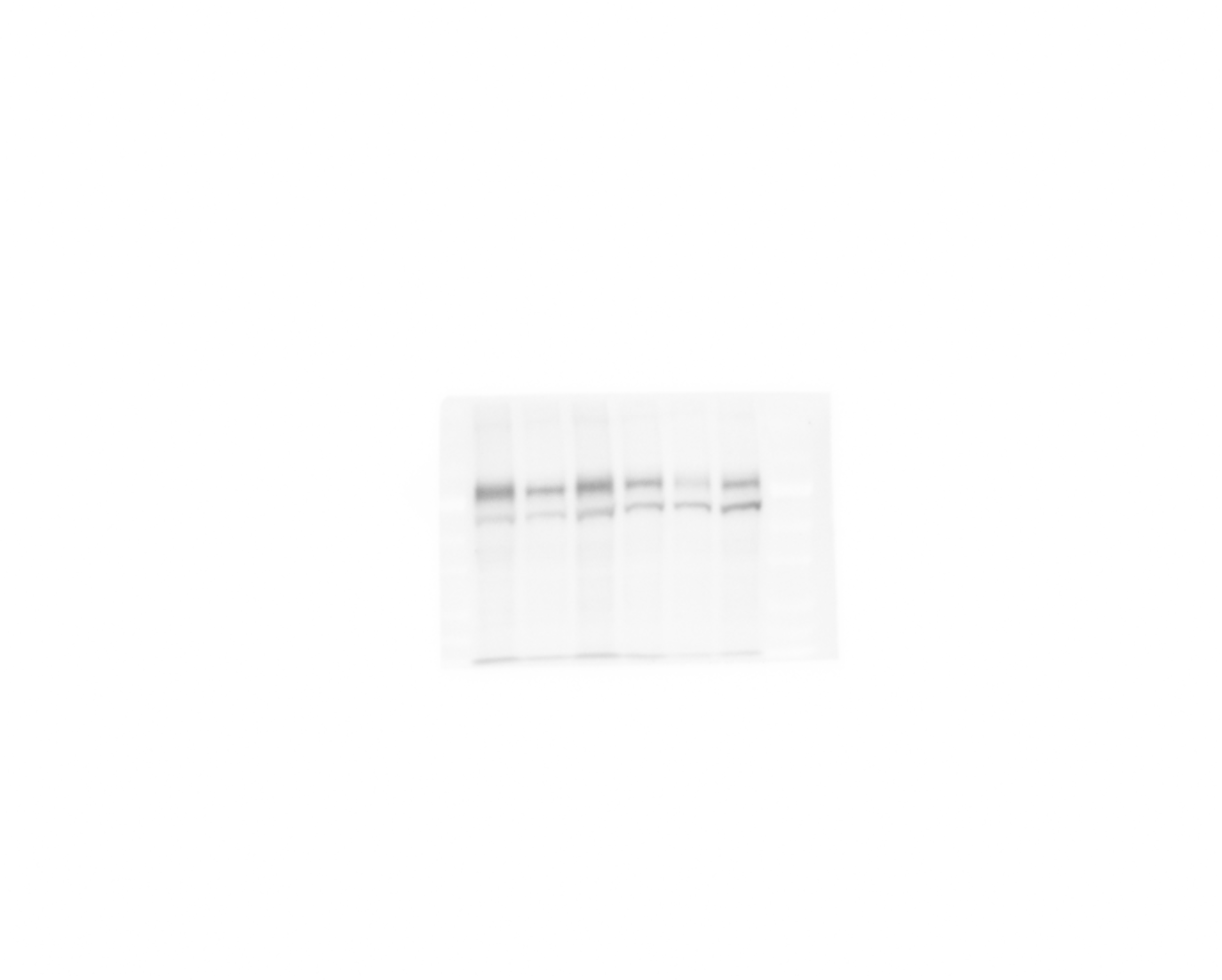

Supplement: Supplementary file 1 [file biomolecules-16-00868-s001.zip › FigureS1 the full, uncropped western blot images/The vivo mice study/HSF1/2-0.3s.Tif]

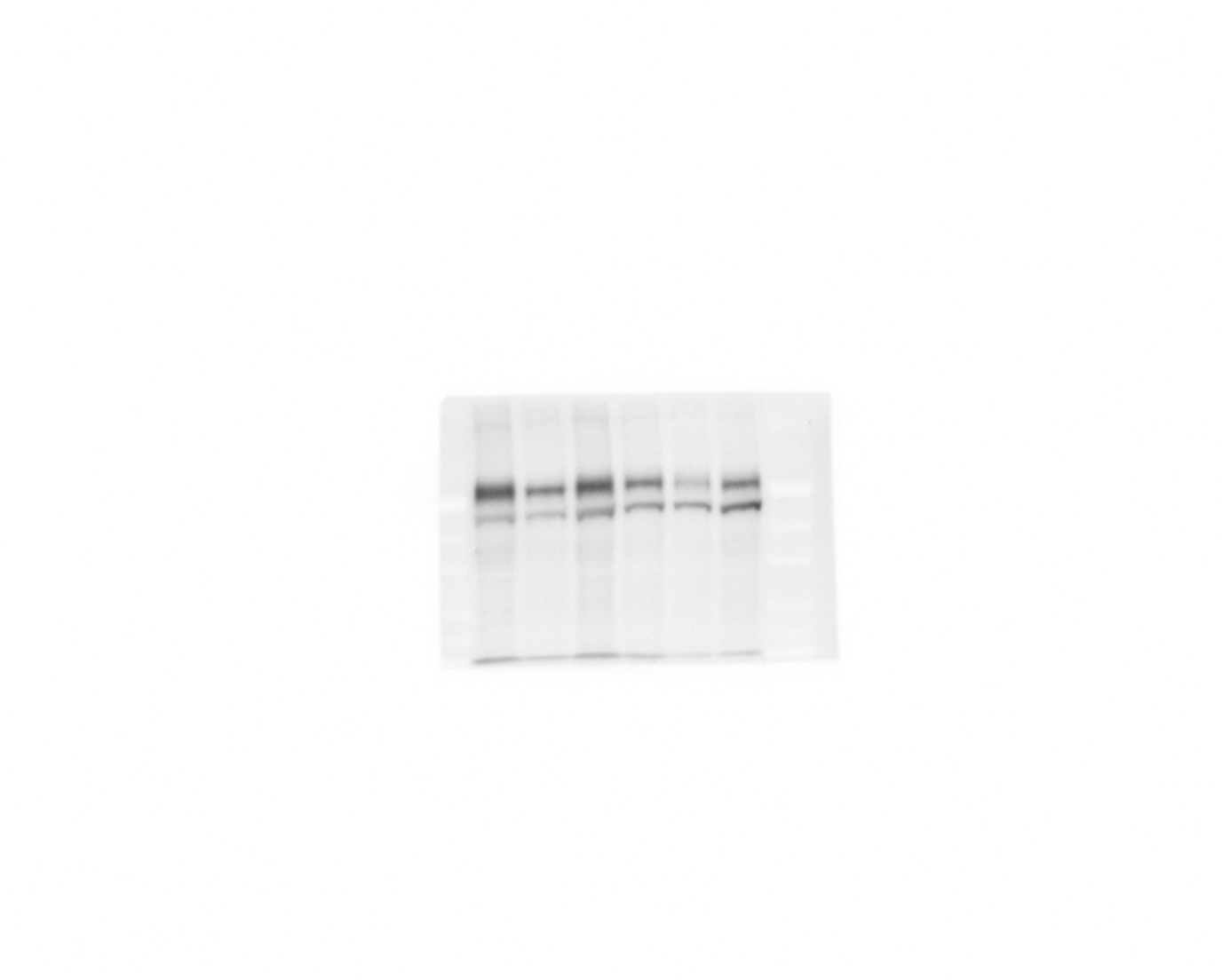

Supplement: Supplementary file 1 [file biomolecules-16-00868-s001.zip › FigureS1 the full, uncropped western blot images/The vivo mice study/HSF1/2-1s.Tif]

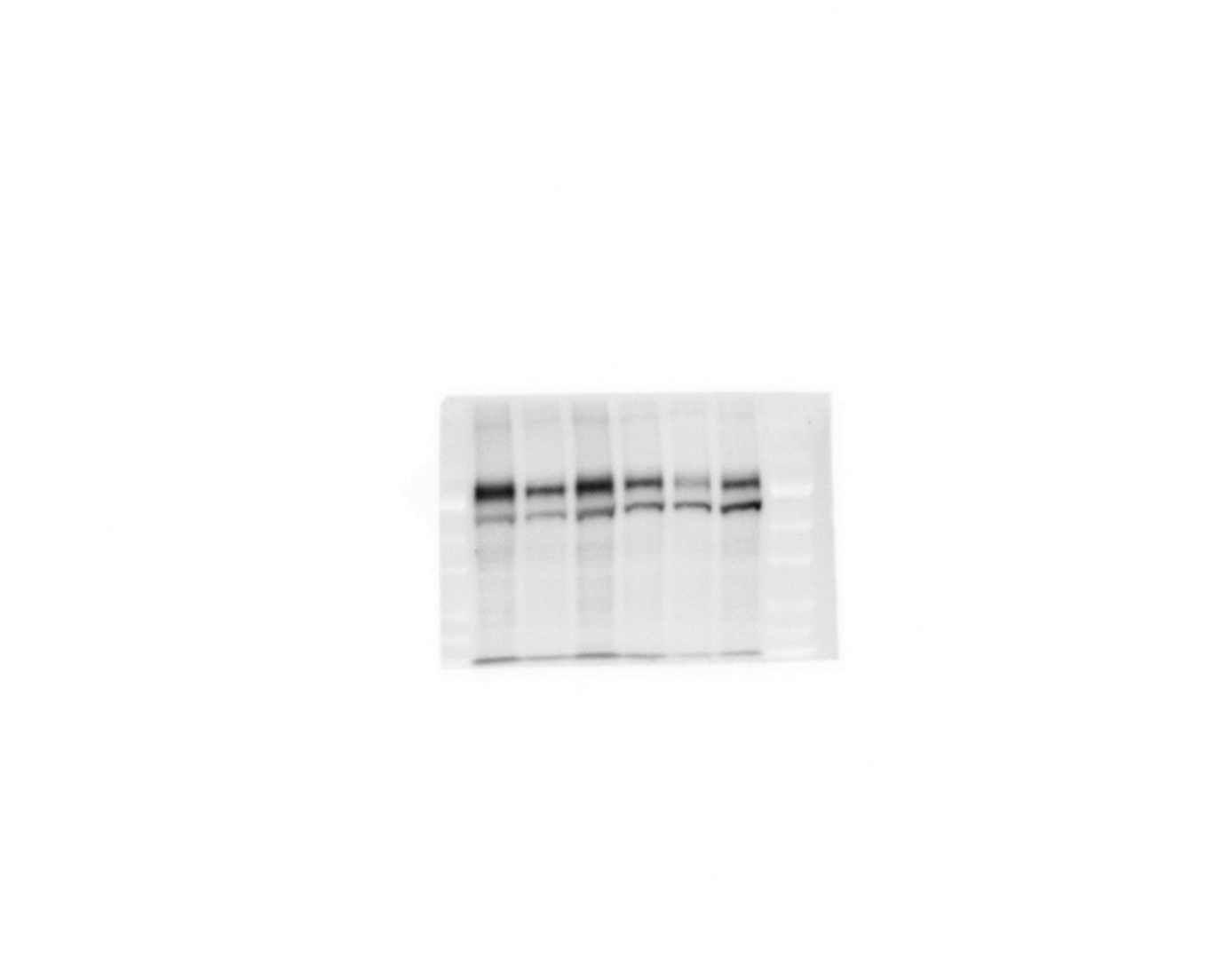

Supplement: Supplementary file 1 [file biomolecules-16-00868-s001.zip › FigureS1 the full, uncropped western blot images/The vivo mice study/HSF1/2-3s.Tif]

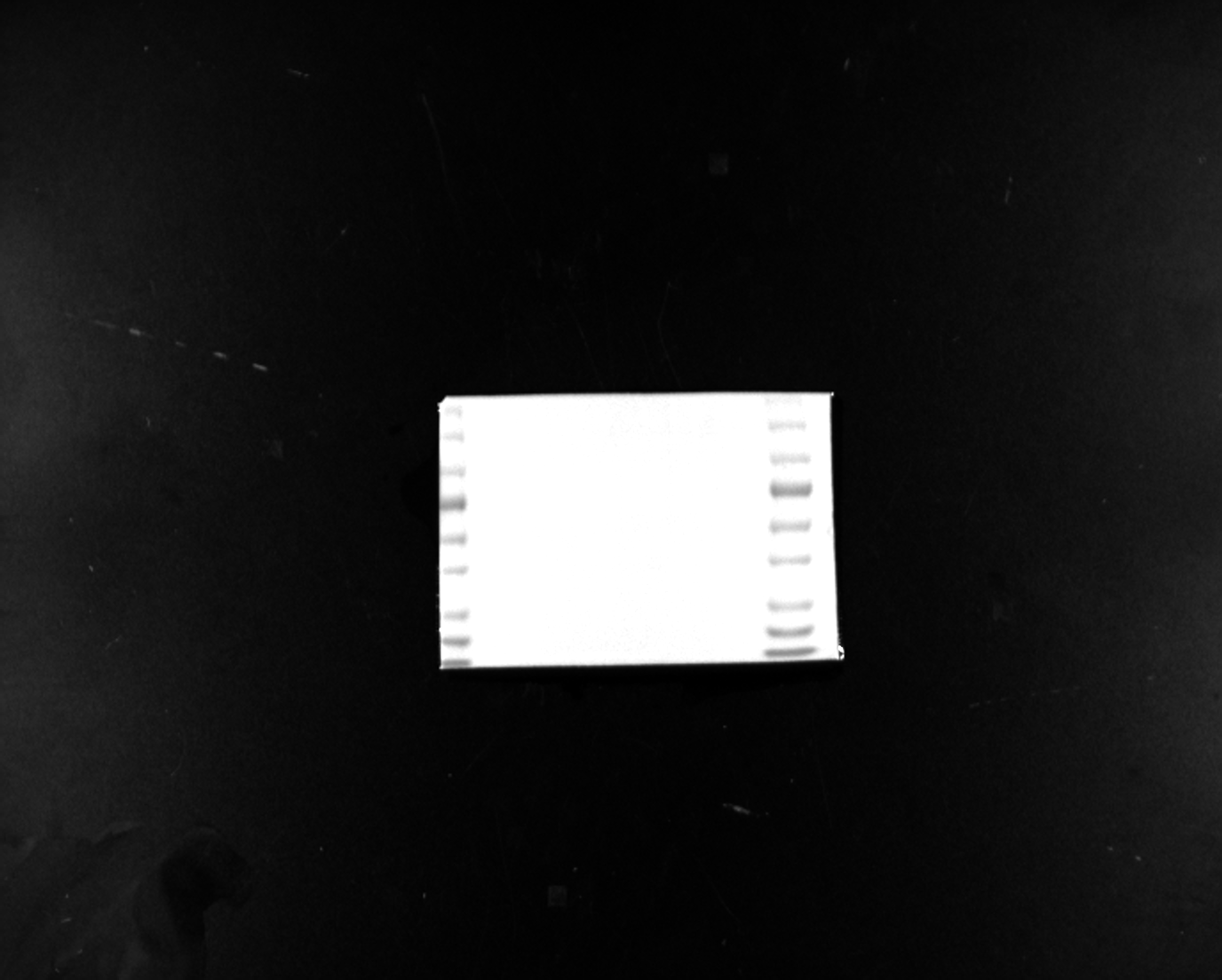

Supplement: Supplementary file 1 [file biomolecules-16-00868-s001.zip › FigureS1 the full, uncropped western blot images/The vivo mice study/HSF1/2-t.Tif]

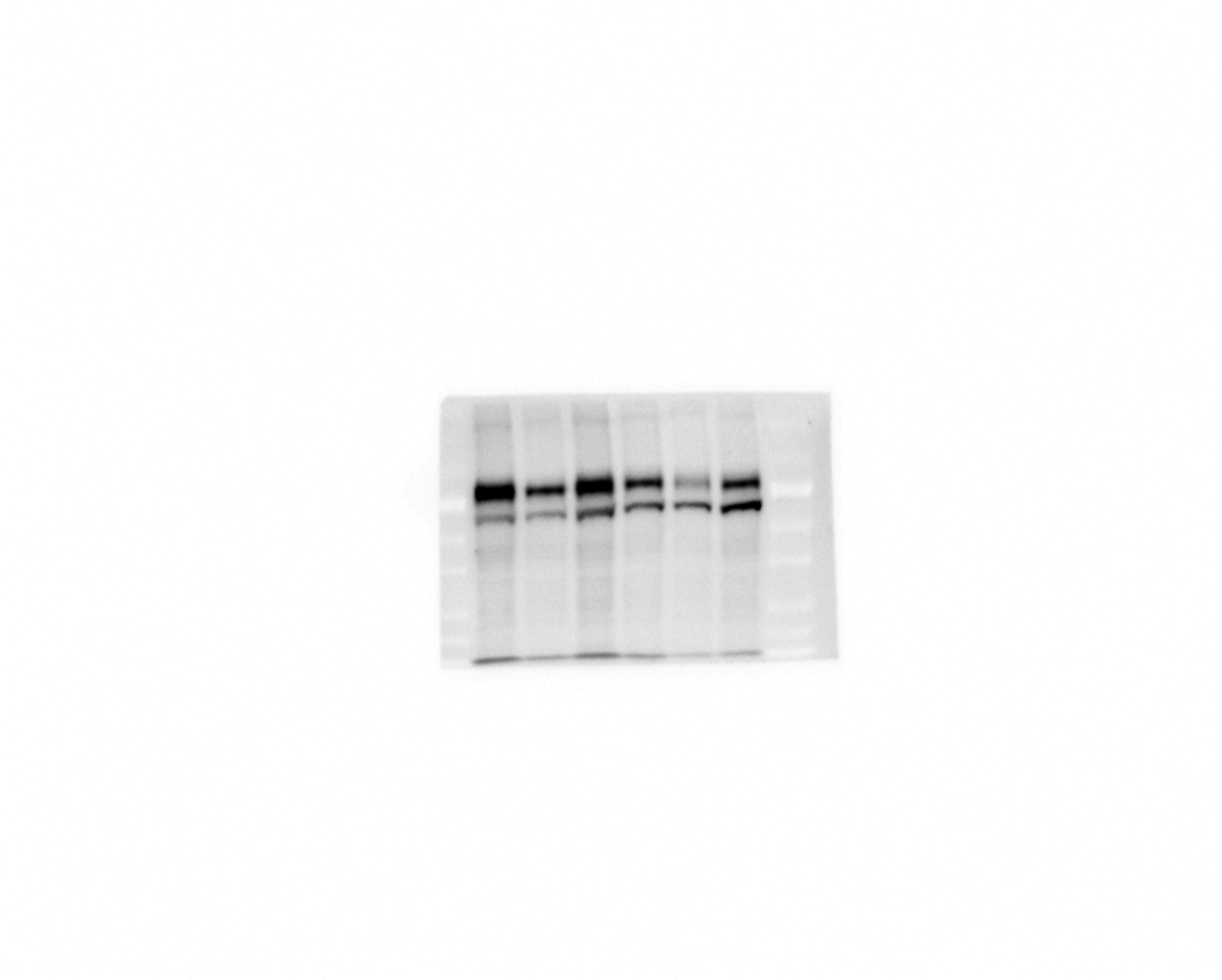

Supplement: Supplementary file 1 [file biomolecules-16-00868-s001.zip › FigureS1 the full, uncropped western blot images/The vivo mice study/HSF1/2.Tif]
