# Supplementary material for: Dysregulation of the HSF1-Mediated UPRmt Pathway in Colonic Smooth Muscle Cells Drives Motility Dysfunction in Functional Constipation
Source: Biomolecules. 2026 Jun 12;16(6):868. doi: 10.3390/biom16060868 (PMC13296460; doi:10.3390/biom16060868)
Supplement: Supplementary file 1 [file biomolecules-16-00868-s001.zip › Supplementary figures.pdf]

Supplementary figures

Figure S1. The original full-blot images.
(See a separate attachments).

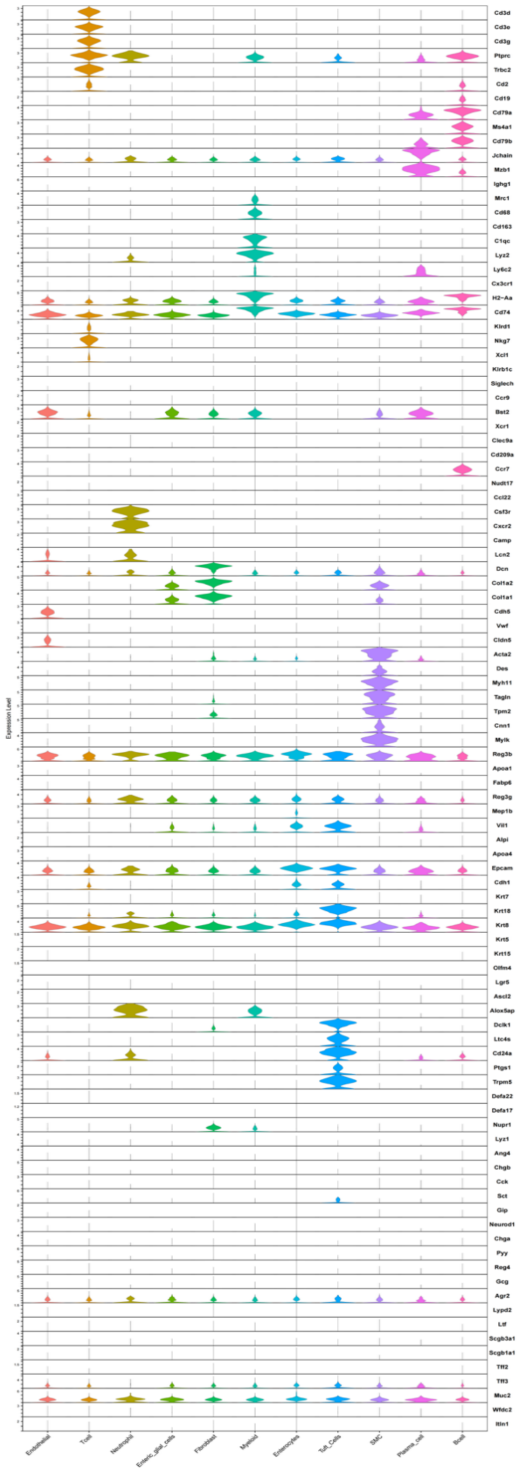

Figure S2. Violin plots showing expression levels of specific markers across all identified cell types.

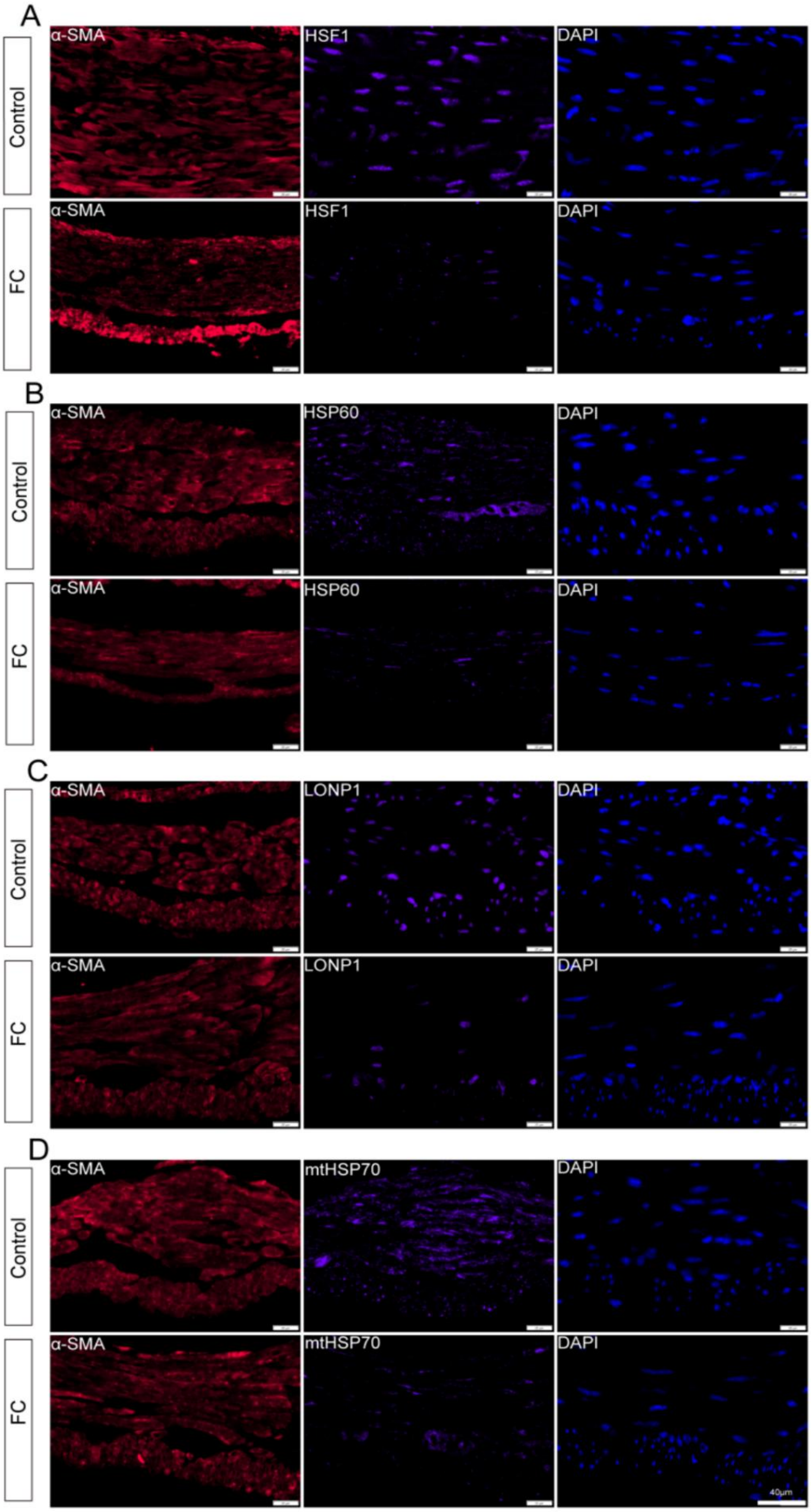

Figure S3. Representative IF images of individual staining channels for UPR^{mt}-related proteins and α-SMA in the control and FC mice. (A) HSF1, (B) HSP60, (C) LONP1, (D) mtHSP70.

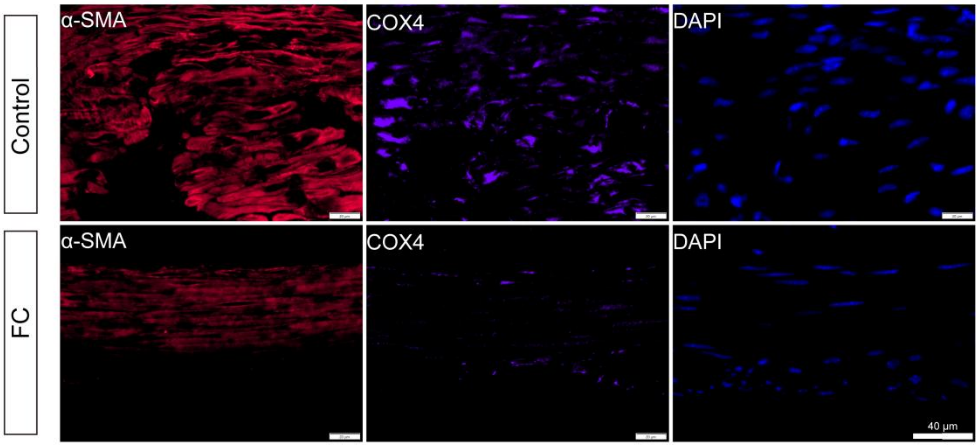

Figure S4. Representative IF images of individual staining channels for COX IV in the control and FC mice

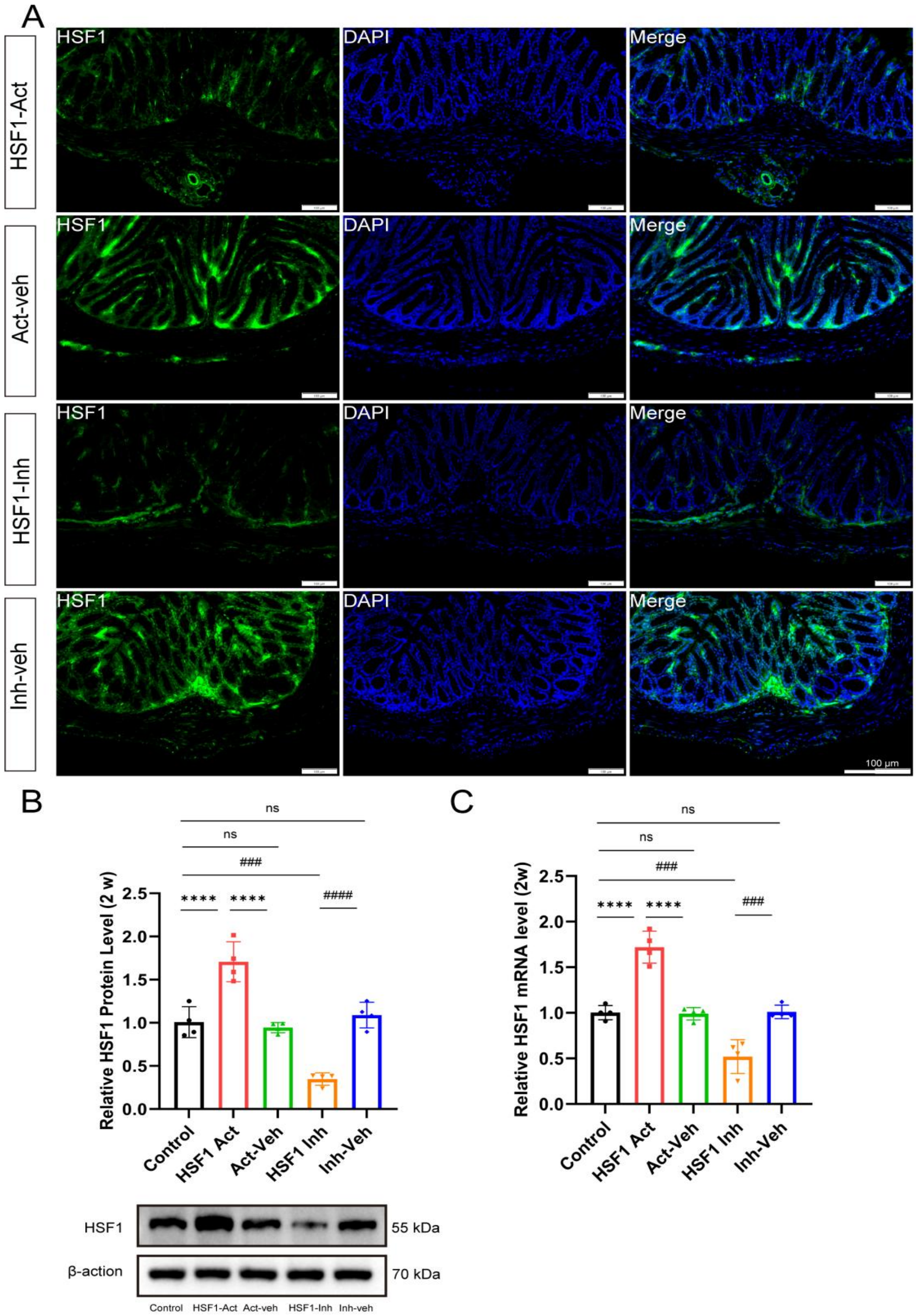

Figure S5. The confirmation of successful AAV9-mediated HSF1 modulation in colonic tissue from four groups of FC mice subjected to AAV9 vectors. (A) Representative IF images showing HSF1 (green) and DAPI (blue) in the smooth muscle layer of the colon from each group, confirming efficient transduction. (B) RT-qPCR analysis of HSF1 mRNA expression in colon tissues from the four groups. (C) Representative Western blot bands for HSF1 protein levels. (C1) Quantification of the western blot band intensities for HSF1, normalized to β-actin (n = 4). Data are presented as mean ± SD (n = 4 per group). $^{**}p < 0.01$ for HSF1+Act vs. Act-Veh or $^{##}p < 0.01$ for HSF1+Inh vs. Inh-Veh (one-way ANOVA with Tukey's post hoc test).

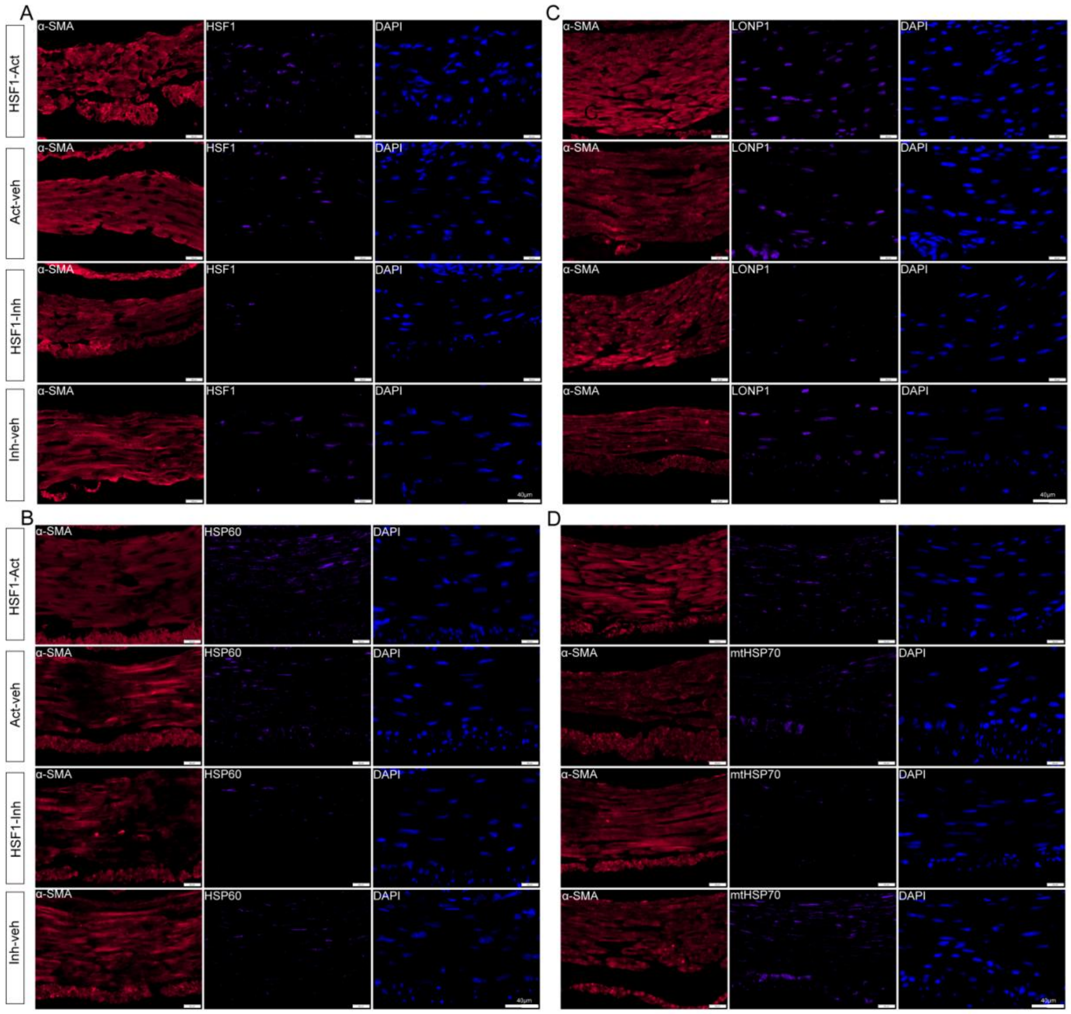

Figure S6. Representative IF images of individual staining channels for UPR^{mt}-related proteins in the AAV9-treated groups. (A) HSF1, (B) LONP1, (C) HSP60, (D) mtHSP70.

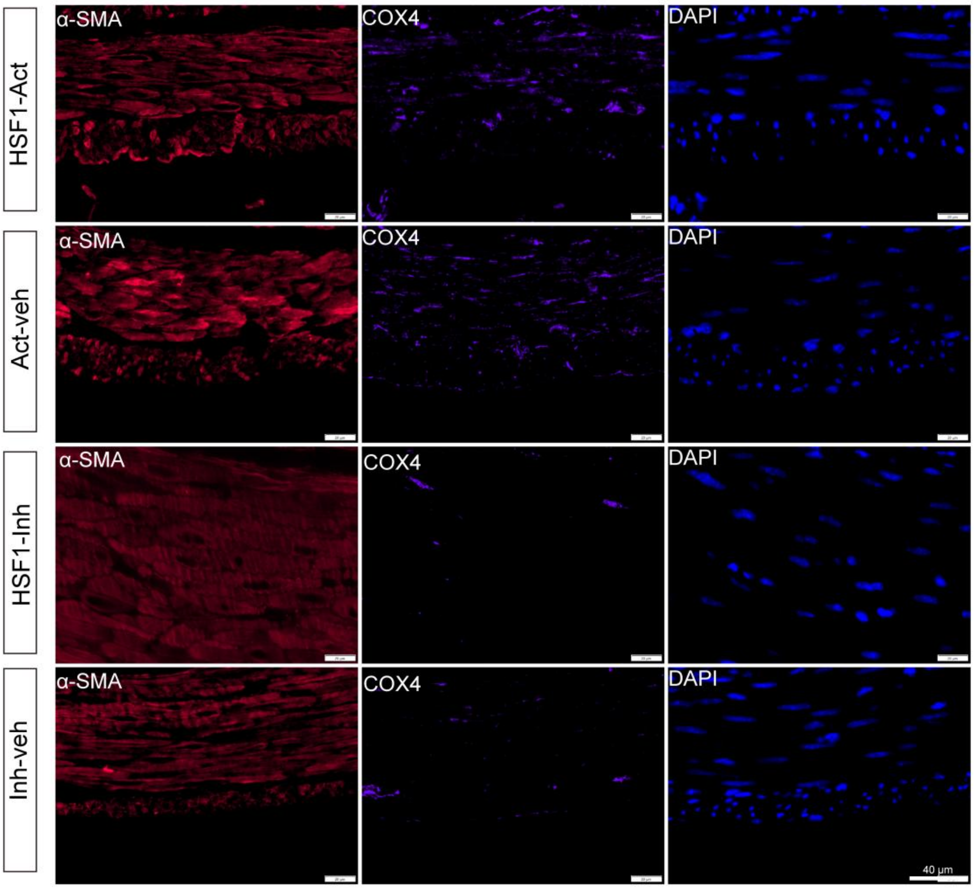

Figure S7. Representative IF images of individual staining channels for COX IV and α-SMA in the four groups subjected to AAV9 vectors.

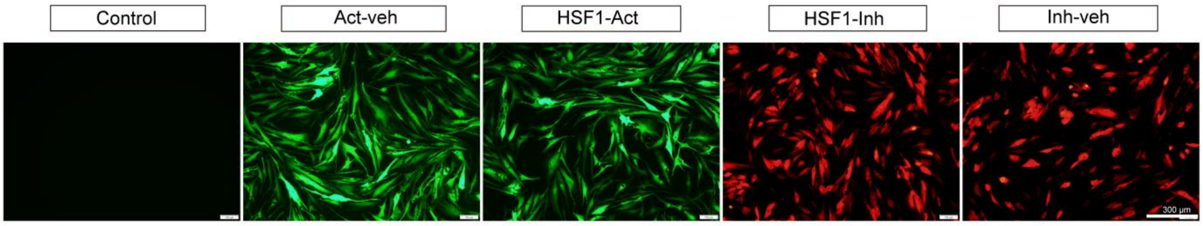

Figure S8. Lentiviral transfection efficiency images in primary SMCs

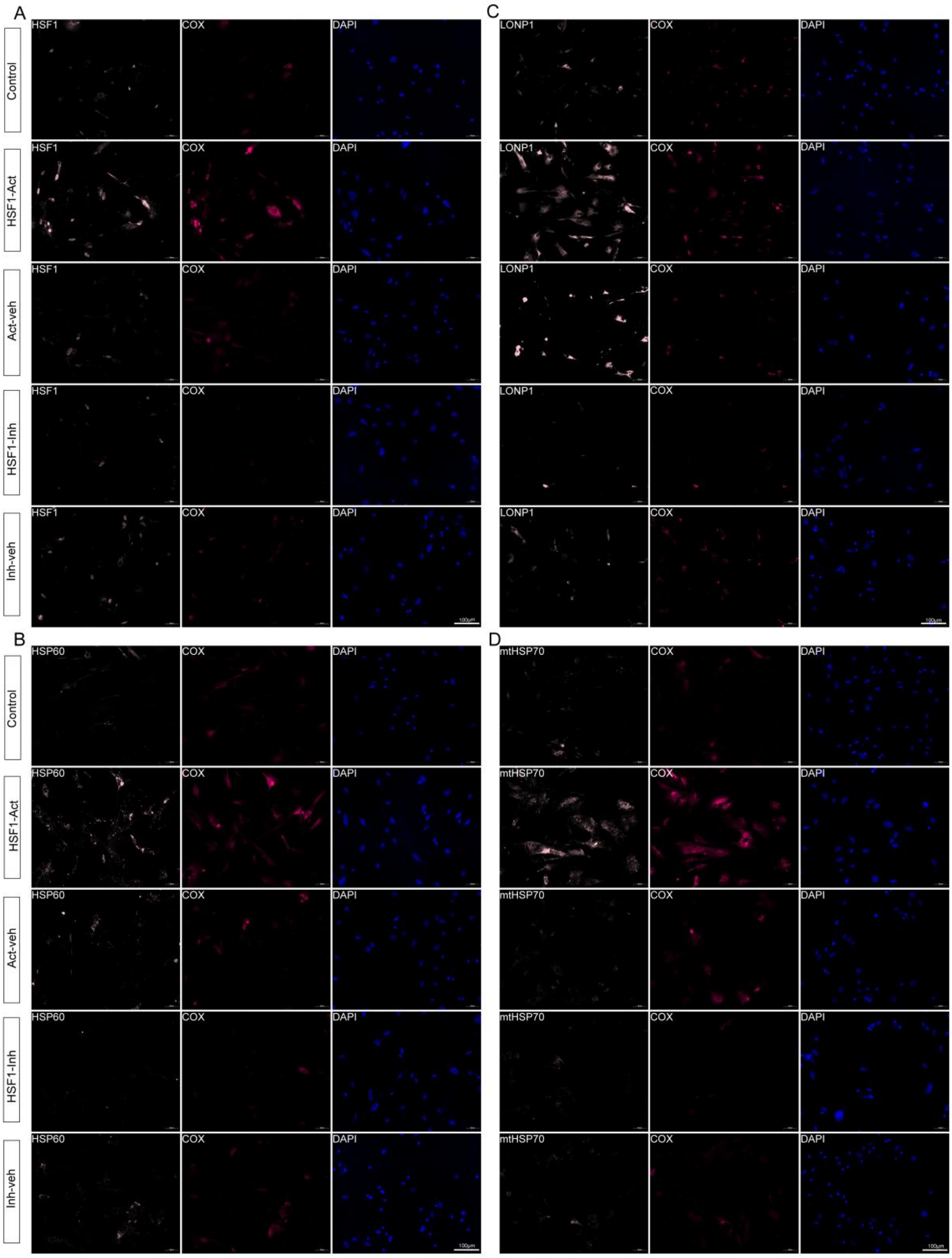

Figure S9. Representative IF images of individual staining channels for UPRmt-related proteins in the LV-transfected primary SMCs. (A) HSF1, (B) LONP1, (C) HSP60, (D) mtHSP70.
