# Supplementary material for: Dysregulation of the HSF1-Mediated UPRmt Pathway in Colonic Smooth Muscle Cells Drives Motility Dysfunction in Functional Constipation
Source: Biomolecules. 2026 Jun 12;16(6):868. doi: 10.3390/biom16060868 (PMC13296460; doi:10.3390/biom16060868)
Supplement: Supplementary file 1 [file biomolecules-16-00868-s001.zip › Supplementary tables.pdf]

Supplementary tables

Table S1. AAV9 vectors used in this study

Virus Name	Target/Gene	Vector Details	Titer (vg/mL)	Volume
AAV9-HSF1-act	Mouse HSF1 (NM_008296.3)	Overexpression construct; C-terminal 3×Flag tag; driven by SMC-specific promoter (Sm22a); co-expressing GFP reporter	6.57×10^{13}	200 µL × 5
AAV9-act-Veh	None (empty vector)	Control vector expressing GFP only; driven by Sm22a promoter	9.54×10^{13}	200 µL
AAV9-HSF1-inh	Mouse HSF1 (NM_008296.3)	shRNA-mediated knockdown; miR30-based shRNA cassette (target sequence: GCACTCTGGTCCGTGTCAAGCTAGTGAAAGCCACAGATGTAGCTTGACACGGACCAGAGTGT); driven by Sm22a promoter; co-expressing GFP reporter	6.25×10^{13}	200 µL × 5
AAV9-inh-Veh	None (scramble control)	Control vector expressing scrambled shRNA (NC sequence: CAGGCAGAAGTATGCAAGACAT) and GFP; driven by Sm22a promoter	8.32×10^{13}	200 µL

Table S2. List of primary and secondary antibodies

REAGENT OR RESOURCE	SOURCE	IDENTIFIER
**IMMUNOFLUORESCENCE (MOUSE TISSUE)**		
**MOUSE MONOCLONAL ANTI-A-SMA**	proteintech	Cat#67735-1-Ig; 1:200
**RABBIT MONOCLONAL ANTI-COX IV**	huabio	Cat#ET1701-63; 1:200
**RABBIT MONOCLONAL ANTI-HSF1**	ABclonal	Cat#A23895; 1:200
**RABBIT MONOCLONAL ANTI-HSP60**	huabio	Cat#ET1609-45; 1:400
**RABBIT MONOCLONAL ANTI-LONP1**	starter	Cat#SOB6032; 1:200
**RABBIT MONOCLONAL ANTI-MTHSP70**	huabio	Cat#R1510-39; 1:100
**CY3-CONJUGATED GOAT ANTI-MOUSE IGG**	Servicebio	Cat#GB21301; 1:200
**CY5-CONJUGATED GOAT ANTI-RABBIT IGG**	Servicebio	Cat#GB27303; 1:200
**IMMUNOFLUORESCENCE (CELL SAMPLE)**		
**RABBIT MONOCLONAL ANTI-COX**	huabio	Cat#ET1610-98; 1:200
**RABBIT MONOCLONAL ANTI-HSF1**	ABclonal	Cat#A23895; 1:200
**RABBIT MONOCLONAL ANTI-HSP60**	huabio	Cat#ET1609-45; 1:500
**RABBIT MONOCLONAL ANTI-LONP1**	starter	Cat#SOB6032; 1:200
**RABBIT MONOCLONAL ANTI-MTHSP70**	huabio	Cat#R1510-39; 1:400
**HRP-CONJUGATED GOAT ANTI-RABBIT IGG**	Servicebio	Cat#GB23303; 1:100
**IF647-TYRAMIDE**	Servicebio	Cat#G1232; 1:500
**IF750-TYRAMIDE**	Servicebio	Cat#G1258-25UL; 1:500

**WESTERN BLOTTING (MOUSE TISSUE)**		
**RABBIT POLYCLONAL ANTI-HSF1**	cusabio	Cat#CSB-PA002974; 1:2000
**RABBIT MONOCLONAL ANTI-HSP60**	huabio	Cat#ET1609-45; 1:5000
**RABBIT POLYCLONAL ANTI-LONP1**	cusabio	Cat#CSB-PA897125; 1:2000
**RABBIT POLYCLONAL ANTI-MTHSP70**	Proteintech	Cat#14887-1-AP; 1:5000
**RABBIT MONOCLONAL ANTI-B-ACTIN**	Abclonal	Cat#AC026; 1:50000
**HRP-CONJUGATED GOAT ANTI-RABBIT IGG**	Abclonal	Cat#AS014; 1:8000
**WESTERN BLOTTING (CELL SAMPLE)**		
**RABBIT POLYCLONAL ANTI-HSF1**	cusabio	Cat#CSB-PA002974; 1:2000
**RABBIT MONOCLONAL ANTI-HSP60**	huabio	Cat#ET1609-45; 1:5000
**RABBIT POLYCLONAL ANTI-LONP1**	cusabio	Cat#CSB-PA897125; 1:2000
**RABBIT POLYCLONAL ANTI-MTHSP70**	Proteintech	Cat#14887-1-AP; 1:5000
**RABBIT POLYCLONAL ANTI-ATP5A**	Proteintech	Cat#14676-1-AP; 1:5000
**RABBIT POLYCLONAL ANTI-NDUFA9**	Abclonal	Cat#A3196; 1:2000
**RABBIT MONOCLONAL ANTI-COX1**	huabio	Cat#ET1610-98; 1:5000
**RABBIT MONOCLONAL ANTI-SDHA**	huabio	Cat#ET1703-40; 1:1000
**RABBIT MONOCLONAL ANTI-UQCRC1**	huabio	Cat#HA721640; 1:5000
**RABBIT MONOCLONAL ANTI-B-ACTIN**	Abclonal	Cat#AC026; 1:50000
**HRP-CONJUGATED GOAT ANTI-RABBIT IGG**	Abclonal	Cat#AS014; 1:8000

Table S3. PCR primers.

**Gene names**	**Primers**
**Mouse colon tissue**	
HSF1	Forward: 5’-CTAGACAATTTGGCTCGCCC-3’<br>Reverse: 5’-AGGTTGGAGTCCATGGCAT-3’
HSP60	Forward: 5’-TGGGGTCACTGTGCAAAGTA-3’<br>Reverse: 5’-TCCCACGCTCTTCGTGTT-3’
LONP1	Forward: 5’-AGAACTTGGACTTGGCTCGG-3’<br>Reverse: 5’-TCCAGGACACGCTTCTTCAC-3’
mtHSP70	Forward: 5’-TGTTCGGCACATTGTCAAGC-3’<br>Reverse: 5’-TTCTCAGCAGCTTCCCGAA-3’
β-actin	Forward: 5’-CTACCTCATGAAGATCCTGACC-3’<br>Reverse: 5’-CACAGCTTCTCTTTGATGTCAC-3’
**Cell sample**	
HSF1	Forward: 5’-CTAGACAATTTGGCTCGCGC-3’<br>Reverse: 5’-CAGGTTGGAGTCCATGGCAT-3’
HSP60	Forward: 5’-TGGGGTCACTGTTGCAAAGT-3’<br>Reverse: 5’-ATCCCCAGCCTCTTCGTTTG-3’
LONP1	Forward: 5’-AGAACTTGGACTTGGCTCGG-3’<br>Reverse: 5’-TCCAGGACACGCTTCTTCAC-3’

mtHSP70	Forward: 5'-TGTTGCGGCACATTGTCAAG-3'<br>Reverse: 5'-CTTCTCAGCAGCTTCCCGAA-3'
β-actin	Forward: 5'-CTACCTCATGAAGATCCTGACC-3'<br>Reverse: 5'-CACAGCTTCTCTTTGATGTCAC-3'

Table S4. The statistical methods and data in Figure 1.

Figure	Statistical Test	Indicator	F value (df)	Post-hoc Comparison	p value
Fig. A	T-test	TGITT	t (18) = 7.076	Control vs. FC	p<0.0001
Fig. B	T-test	6-h fecal pallets	t (12) = 2.400	Control vs. FC	p<0.0001
Fig. C	T-test	Fecal water content	t (18) = 2.898	Control vs. FC	p=0.0096
Fig.D	T-test	small intestinal propulsive rate	t (18) = 2.572	Control vs. FC	p=0.0192
Fig.E	T-test	5-HT	t (8) = 5.461	Control vs. FC	p=0.0006
	T-test	VIP	t (8) = 4.996	Control vs. FC	p=0.0011
	T-test	TPH-1	t (8) = 7.213	Control vs. FC	p<0.0001

Note: The 6-week time point was chosen for detailed mechanistic investigations because the behavioral deficits and the proposed molecular pathways were most prominently manifested and modulated at this endpoint.

Table S5. The statistical methods and data in Figure 3.

Figure	Statistical Test	Indicator	F value (df)	Post-hoc Comparison	Post-hoc p value
Fig.A1	T-test	HSF1/IF	t (8) = 5.694	Control vs. FC	p=0.0005
Fig.A2	T-test	HSP60/IF	t (8) = 10.10	Control vs. FC	p<0.0001
Fig.A3	T-test	mtHSP70/IF	t (8) = 7.535	Control vs. FC	p<0.0001
Fig.A4	T-test	LONP1/IF	t (8) = 11.09	Control vs. FC	p<0.0001
Fig.B1	T-test	HSF1/WB	t (8) = 12.12	Control vs. FC	p<0.0001

Figure	Statistical Test	Indicator	F value (df)	Post-hoc Comparison	Post-hoc p value
Fig.B2	T-test	HSP60/WB	t (8) = 6.444	Control vs. FC	p=0.0002
Fig.B3	T-test	mtHSP70/WB	t (8) = 5.378	Control vs. FC	p=0.0007
Fig.B4	T-test	LONP1/WB	t (8) = 5.106	Control vs. FC	p=0.0009
Fig.C	T-test	HSF1/RT-qPCR	t (6) = 2.919	Control vs. FC	p=0.0267
Fig.D	T-test	HSP60/RT-qPCR	t (8) = 5.035	Control vs. FC	p=0.0010
Fig.E	T-test	mtHSP70/RT-qPCR	t (8) = 3.568	Control vs. FC	p=0.0073
Fig.F	T-test	LONP1/RT-qPCR	t (4.525) = 4.64	Control vs. FC	p=0.0072

Table S6. The statistical methods and data in Figure 4.

Figure	Statistical Test	Indicator	F value (df)	Post-hoc Comparison	Post-hoc p value
Fig. A1	T-test	COX/IV	t (8) = 6.450	Control vs. FC	p=0.0002
Fig. B1	T-test	mitochondrial density	t (4.209) = 4.066	Control vs. FC	P=0.0138
Fig. C1	T-test	mitochondrial membrane potential	t (8) = 28.66	Control vs. FC	p<0.0001
Fig. D1	T-test	mtROS	t (4) = 5.542	Control vs. FC	p=0.0052
Fig. E	T-test	ATP	t (8) = 3.869	Control vs. FC	p=0.0047
Fig. F	T-test	activities of complexes I	t (8) = 12.25	Control vs. FC	p<0.0001

Figure	Statistical Test	Indicator	F value (df)	Post-hoc Comparison	Post-hoc p value
	T-test	activities of complexes II	t (8) = 7.485	Control vs. FC	p<0.0001
	T-test	activities of complexes III	t (8) = 5.604	Control vs. FC	p=0.0005
	T-test	activities of complexes IV	t (8) = 6.426	Control vs. FC	p=0.0002
	T-test	activities of complexes V	t (8) = 8.157	Control vs. FC	p<0.0001

Table S7. The statistical methods and data in Figure 5.

Figure	Statistical Test	indicator/detection method	F value (df)	p value	Post-hoc Comparison	Post-hoc p value
Fig. B1	One-way ANOVA	HSF1/IF	F (3, 16) = 18.36	p<0.0001	HSF1+Act vs. Act-Veh	P=0.0082
					HSF1+Inh vs. Inh-Veh	P=0.0341
Fig. B2	One-way ANOVA	HSP60/IF	F (3, 12) = 57.24	p<0.0001	HSF1+Act vs. Act-Veh	P=0.0002
					HSF1+Inh vs. Inh-Veh	p<0.0001
Fig. B3	One-way ANOVA	mtHSP70/IF	F (3, 16) = 19.24	p<0.0001	HSF1+Act vs. Act-Veh	P=0.0047
					HSF1+Inh vs. Inh-Veh	P=0.0010
Fig. B4	One-way ANOVA	LONP1/IF	F (3, 16) = 58.59	p<0.0001	HSF1+Act vs. Act-Veh	P=0.0002
					HSF1+Inh vs. Inh-Veh	P=0.0003
Fig. C	One-way	HSF1/WB	F (3,	p<0.0001	HSF1+Act	p<0.0001

Figure	Statistical Test	indicator/detection method	F value (df)	p value	Post-hoc Comparison	Post-hoc p value
	ANOVA		12) = 205.1		vs. Act-Veh	
					HSF1+Inh vs. Inh-Veh	p=0.0001
	One-way ANOVA	HSP60/WB	F (3, 16) = 73.11	p<0.0001	HSF1+Act vs. Act-Veh	p<0.0001
					HSF1+Inh vs. Inh-Veh	P=0.0015
	One-way ANOVA	mtHSP70/WB	F (3, 12) = 50.97	p<0.0001	HSF1+Act vs. Act-Veh	p<0.0001
					HSF1+Inh vs. Inh-Veh	P=0.0003
	One-way ANOVA	LONP1/WB	F (3, 16) = 54.03	p<0.0001	HSF1+Act vs. Act-Veh	p<0.0001
					HSF1+Inh vs. Inh-Veh	p<0.0001
Fig. D	One-way ANOVA	HSF1/RT-qPCR	F (3, 12) = 15.85	P=0.0002	HSF1+Act vs. Act-Veh	p=0.0033
					HSF1+Inh vs. Inh-Veh	p=0.0318
	One-way ANOVA	HSP60/RT-qPCR	F (3, 12) = 31.66	p<0.0001	HSF1+Act vs. Act-Veh	p=0.0001
					HSF1+Inh vs. Inh-Veh	p=0.0050
	One-way ANOVA	mtHSP70/RT-qPCR	F (3, 12) = 14.68	P=0.0003	HSF1+Act vs. Act-Veh	p=0.0161
					HSF1+Inh vs. Inh-Veh	p=0.0260

Figure	Statistical Test	indicator/detection method	F value (df)	p value	Post-hoc Comparison	Post-hoc p value
	One-way ANOVA	LONP1/RT-qPCR	F (3, 8) = 30.87	p<0.0001	HSF1+Act vs. Act-Veh	p=0.0005
					HSF1+Inh vs. Inh-Veh	p=0.0094

Table S8. The statistical methods and data in Figure 6.

Figure	Statistical Test	indicator/detection method	F value (df)	p value	Post-hoc Comparison	Post-hoc p value
Fig. A1	One-way ANOVA	COX IV/IF	F (3, 12) = 30.45	p<0.0001	HSF1+Act vs. Act-Veh	p=0.0002
					HSF1+Inh vs. Inh-Veh	p=0.0066
Fig. B1	One-way ANOVA	mitochondrial density	F (3, 16) = 29.18	p<0.0001	HSF1+Act vs. Act-Veh	p=0.0001
					HSF1+Inh vs. Inh-Veh	p=0.0016
Fig. C1	One-way ANOVA	mitochondrial membrane potential	F (3, 12) = 628.7	p<0.0001	HSF1+Act vs. Act-Veh	p<0.0001
					HSF1+Inh vs. Inh-Veh	p<0.0001
Fig. D1	One-way ANOVA	mtROS	F (3, 12) = 429.9	p<0.0001	HSF1+Act vs. Act-Veh	p<0.0001
					HSF1+Inh vs. Inh-Veh	p=0.0314
Fig. E	One-way ANOVA	ATP	F (3, 12) = 14.56	p=0.0003	HSF1+Act vs. Act-Veh	p=0.0039
					HSF1+Inh vs.	p=0.0164

Figure	Statistical Test	indicator/detection method	F value (df)	p value	Post-hoc Comparison	Post-hoc p value
					Inh-Veh	
Fig. F	One-way ANOVA	activities of complexes I	F (3, 8) = 32.57	p<0.0001	HSF1+Act vs. Act-Veh	p=0.0003
					HSF1+Inh vs. Inh-Veh	p=0.0249
	One-way ANOVA	activities of complexesII	F (3, 16) = 16.72	p<0.0001	HSF1+Act vs. Act-Veh	p=0.0126
					HSF1+Inh vs. Inh-Veh	p=0.0037
	One-way ANOVA	activities of complexes III	F (3, 16) = 14.66	p<0.0001	HSF1+Act vs. Act-Veh	p=0.0381
					HSF1+Inh vs. Inh-Veh	p=0.0151
	One-way ANOVA	activities of complexes IV	F (3, 8) = 22.28	p=0.0003	HSF1+Act vs. Act-Veh	p=0.0047
					HSF1+Inh vs. Inh-Veh	p=0.0128
	One-way ANOVA	activities of complexes V	F (3, 16) = 21.94	p<0.0001	HSF1+Act vs. Act-Veh	p=0.0002
					HSF1+Inh vs. Inh-Veh	p=0.0346

Table S9. The statistical methods and data in Figure 7.

Figure	Statistical Test	indicator/detection method	F value (df)	p value	Post-hoc Comparison	Post-hoc p value
Fig. A	One-way ANOVA	TGITT	F (3, 36) = 41.79	p<0.0001	HSF1+Act vs. Act-Veh	p<0.0001
					HSF1+Inh vs.	p=0.0014

**Figure**	**Statistical Test**	**indicator/detection method**	**F value (df)**	**p value**	**Post-hoc Comparison**	**Post-hoc p value**
					Inh-Veh	
Fig. B	One-way ANOVA	6-h fecal pallets	F (3, 36) = 26.32	p<0.0001	HSF1+Act vs. Act-Veh	p<0.0001
					HSF1+Inh vs. Inh-Veh	p=0.0455
Fig. C	One-way ANOVA	fecal water content	F (3, 16) = 15.75	p<0.0001	HSF1+Act vs. Act-Veh	p=0.0003
					HSF1+Inh vs. Inh-Veh	p=0.0270
Fig. D	One-way ANOVA	small intestinal propulsive rate	F (3, 32) = 9.797	p<0.0001	HSF1+Act vs. Act-Veh	p=0.0069
					HSF1+Inh vs. Inh-Veh	p=0.0302
Fig. G	One-way ANOVA	5-HT	F (3, 12) = 23.54	p<0.0001	HSF1+Act vs. Act-Veh	p=0.0016
					HSF1+Inh vs. Inh-Veh	p=0.0075
	One-way ANOVA	VIP	F (3, 12) = 18.76	p<0.0001	HSF1+Act vs. Act-Veh	p=0.0110
					HSF1+Inh vs. Inh-Veh	p=0.0038
	One-way ANOVA	TPH-1	F (3, 12) = 14.46	p=0.0003	HSF1+Act vs. Act-Veh	p=0.0176
					HSF1+Inh vs. Inh-Veh	p=0.0246

Table S10. The statistical methods and data in Figure 8.

Figure	Statistical Test	indicator/detection method	F value (df)	p value	Post-hoc Comparison	Post-hoc p value
Fig. C1	One-way ANOVA	HSF1/WB	F (4, 10) = 163.6	p<0.0001	LV-HSF1-OE vs. LV-veh	p<0.0001
					LV-shHSF1 vs. LV-shVeh	p=0.0008
	One-way ANOVA	HSP60/WB	F (4, 10) = 108.0	p<0.0001	LV-HSF1-OE vs. LV-veh	p<0.0001
					LV-shHSF1 vs. LV-shVeh	P=0.0013
	One-way ANOVA	mtHSP70/WB	F (4, 10) = 28.83	p<0.0001	LV-HSF1-OE vs. LV-veh	p=0.0007
					LV-shHSF1 vs. LV-shVeh	P=0.0046
	One-way ANOVA	LONP1/WB	F (4, 10) = 38.44	p<0.0001	LV-HSF1-OE vs. LV-veh	p=0.0004
					LV-shHSF1 vs. LV-shVeh	p=0.0006
Fig. D	One-way ANOVA	HSF1/PCR	F (4, 10) = 949.6	p<0.0001	LV-HSF1-OE vs. LV-veh	p<0.0001
					LV-shHSF1 vs. LV-shVeh	p=0.0812
	One-way ANOVA	HSP60/PCR	F (4, 10) = 8.574	P=0.0029	LV-HSF1-OE vs. LV-veh	p=0.0405
					LV-shHSF1 vs. LV-shVeh	p=0.1717
	One-way ANOVA	mtHSP70/PCR	F (4, 10) = 6.824	P=0.0065	LV-HSF1-OE vs. LV-veh	p=0.1496
					LV-shHSF1	p=0.2271

Figure	Statistical Test	indicator/detection method	F value (df)	p value	Post-hoc Comparison	Post-hoc p value
					vs. LV-shVeh	
	One-way ANOVA	LONP1/PCR	F (4, 10) = 7.173	P=0.0054	LV-HSF1-OE vs. LV-veh	p=0.1352
					LV-shHSF1 vs. LV-shVeh	p=0.1748

Table S11. The statistical methods and data in Figure 9.

Figure	Statistical Test	indicator/detection method	F value (df)	p value	Post-hoc Comparison	Post-hoc p value
Fig. A1	One-way ANOVA	mitochondrial density	F (4, 10) = 55.30	p<0.0001	LV-HSF1-OE vs. LV-veh	p<0.0001
					LV-shHSF1 vs. LV-shVeh	P=0.0108
Fig. B	One-way ANOVA	ATP5A	F (4, 10) = 98.80	p<0.0001	LV-HSF1-OE vs. LV-veh	p<0.0001
					LV-shHSF1 vs. LV-shVeh	p=0.0006
	One-way ANOVA	NDUFA9	F (4, 10) = 58.35	p<0.0001	LV-HSF1-OE vs. LV-veh	p<0.0001
					LV-shHSF1 vs. LV-shVeh	p=0.0013
	One-way ANOVA	COX1	F (4, 10) = 19.19	p=0.0001	LV-HSF1-OE vs. LV-veh	p=0.0029
					LV-shHSF1 vs. LV-shVeh	p=0.0681
	One-way ANOVA	SDHA	F (4, 10) = 293.9	p<0.0001	LV-HSF1-OE vs. LV-veh	p<0.0001
					LV-shHSF1 vs.	p<0.0001

Figure	Statistical Test	indicator/detection method	F value (df)	p value	Post-hoc Comparison	Post-hoc p value
					LV-shVeh	
	One-way ANOVA	UQCRC1	F (4, 10) = 54.98	p<0.0001	LV-HSF1-OE vs. LV-veh	p<0.0001
					LV-shHSF1 vs. LV-shVeh	p=0.0011
Fig. C	One-way ANOVA	ATP	F (4, 10) = 21.58	p<0.0001	LV-HSF1-OE vs. LV-veh	p=0.0085
					LV-shHSF1 vs. LV-shVeh	p=0.0040
